# Free-radical cyclization approach to polyheterocycles containing pyrrole and pyridine rings

**DOI:** 10.3762/bjoc.17.105

**Published:** 2021-06-23

**Authors:** Ivan P Mosiagin, Olesya A Tomashenko, Dar’ya V Spiridonova, Mikhail S Novikov, Sergey P Tunik, Alexander F Khlebnikov

**Affiliations:** 1 Saint Petersburg State University, Institute of Chemistry, 7/9 Universitetskaya nab., St. Petersburg 199034, Russia

**Keywords:** arylation, pyridine, pyrrole, radical cyclization, tris(trimethylsilyl)silane

## Abstract

A wide range of derivatives with new pyrido[2,1-*a*]pyrrolo[3,4-*c*]isoquinoline skeleton was synthesized by free-radical intramolecular cyclization of *o*-bromophenyl-substituted pyrrolylpyridinium salts using the (TMS)_3_SiH/AIBN system. The cyclization provides generally good yields of pyrido[2,1-*a*]pyrrolo[3,4-*c*]isoquinoline hydrobromides having no additional radical-sensitive substituents. The free bases can be obtained from the synthesized hydrobromides in quantitative yield by basification at room temperature. The selectivity control of intramolecular arylation was achieved by replacing the halogen: the use of 1-(2-(*ortho*-bromophenyl)-4-(*ortho*-iodophenyl)pyrrol-3-yl)pyridinium bromide makes it possible to obtain a monocyclization product, and the bicyclization product from the dibromo derivative. The procedure is also applicable to obtain 3-arylpyrido[2,1-*a*]pyrrolo[3,2-*c*]isoquinoline derivatives including 2-unsubstituted skeletons that are inaccessible via Pd-catalyzed cyclization.

## Introduction

**Scheme 1:**
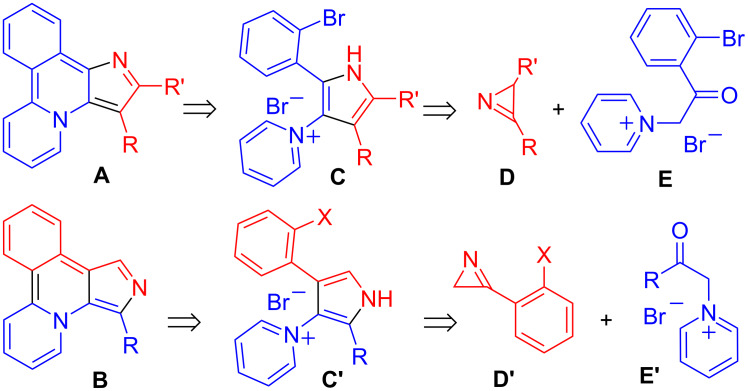
Retrosynthetic analysis of heterocycles **A** and **B**.

Polycyclic heteroaromatic molecules, which have a tunable electronic structure and excellent self-assembling properties, are highly desirable in materials science, especially in post-silicon electronics [1–4]. Though the remarkable progress in the synthesis of the polycyclic heteroarenes during the last two decades, further development of the existing methodologies is still required for broadening of the scope of polycyclic heteroaromatics available for various applications. Fused polyheteroarenes, containing a pyrrole moiety, are widely present in natural products and pharmacologically important agents and have a significant importance in the development of new materials useful for bioimaging applications and chemosensor systems [3–13]. Pyrroles with vicinal *o*-bromophenyl and heteroaryl substituents, which are readily accessible via reactions of the corresponding 2*H*-azirines and phenacylcycloiminium ylides [14–19], are excellent precursors for various fused aza-heteroaromatics via metal-catalyzed arylation [17–19]. We have recently successfully applied this approach to synthesize the new luminescent heterocyclic system pyrido[2,1-*a*]pyrrolo[3,2-*c*]isoquinoline **A** [17], which turned out to be useful for bioimaging [13] and as a ligand for the preparation of luminescent metal complexes, Au(I) [20], Ir(III) [21] and Eu(III) [22] (Scheme 1). According to the calculations, the isomeric pyrido[2,1-*a*]pyrrolo[3,4-*c*]isoquinoline system **B** (Scheme 1) should have no less interesting photophysical properties [17], than skeleton **A** but its synthesis is still a challenge. In particular, attempts to assemble this framework using Pd-catalyzed intramolecular arylation of compounds **C'** (X = Br, I) failed [17]. In this work, we report an effective method for the assembly of pyrido[2,1-*a*]pyrrolo[3,4-*c*]isoquinoline and related frameworks via a free radical cyclization of pyrrolylpyridinium salts.

## Results and Discussion

**Figure 1:**
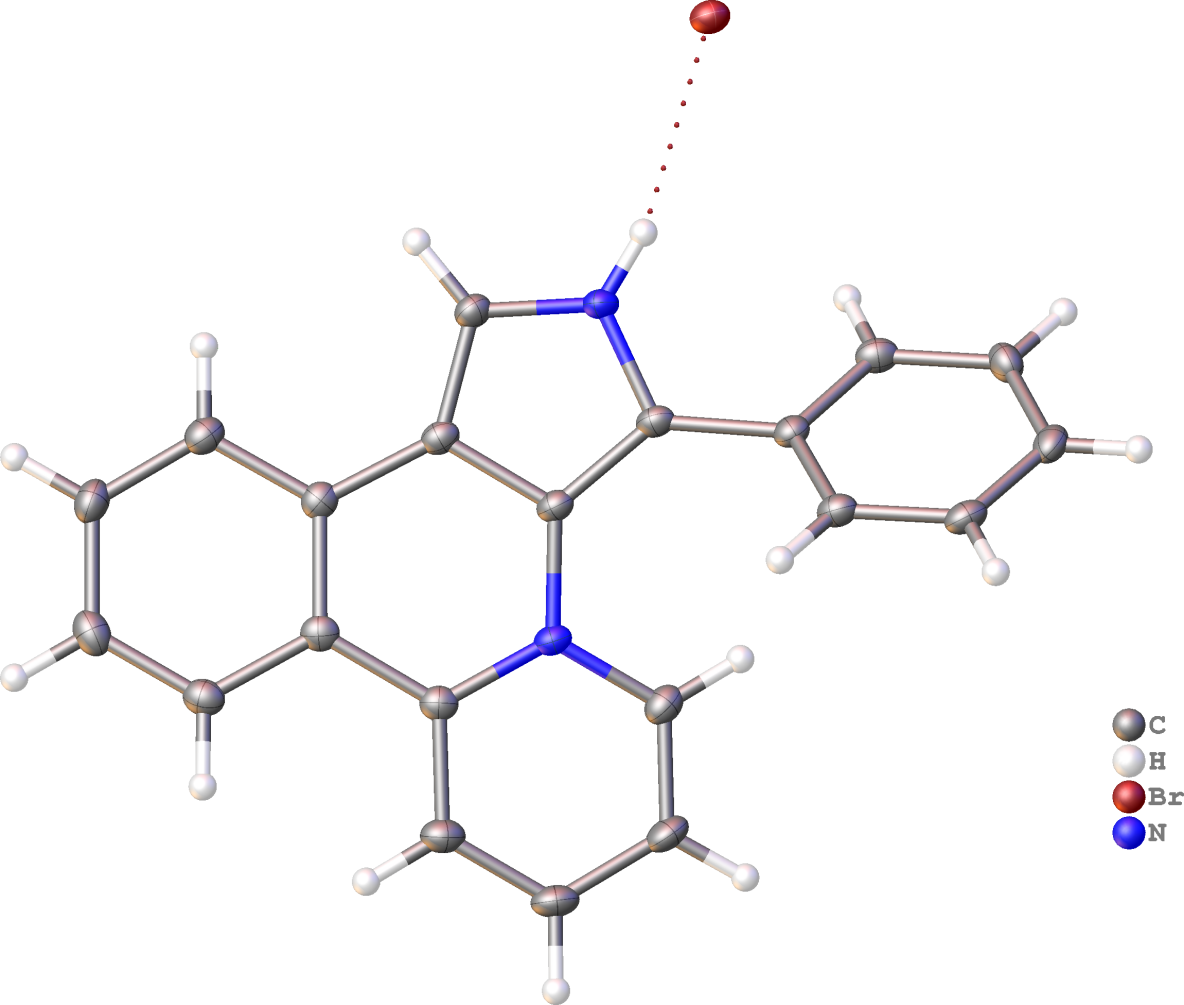
Molecular structure of compound **3a**, displacement parameters are drawn at 50%.

**Scheme 2:**
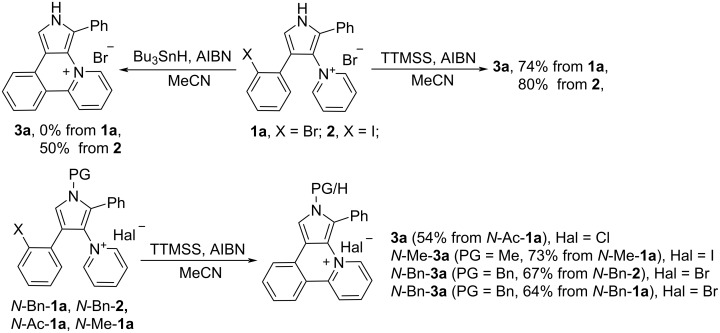
Free-radical cyclization of *N*-protected and *N*-unprotected pyrroles **1a** and **2**.

Although the intramolecular free-radical cyclization is widely used for the synthesis of heteroaromatic compounds [23–26], only a few examples of the successful use of radical cyclization for the synthesis of pyrrole-containing fused heteroaromatics are known in the literature [23–30]. We initiated our study with the attempts to carry out the cyclization of 4-(2-bromophenyl)pyrrole **1a** under standard radical cyclization conditions (azobisisobutyronitrile (AIBN)/Bu_3_SnH), which previously had been successfully used for the cyclization of 2-bromophenyl-substituted pyrroles into 7-oxa-2a^1^-azabenzo[*b*]-cyclopenta[*pq*]pleiadenes [30]. However, an attempt to use AIBN/Bu_3_SnH for the cyclization of pyrrole **1a** to compound **3a** only led to a tarring of the reaction mixture, regardless of the temperature (70–110 °C, MeCN) and the protocol for mixing the reagents (Scheme 2). Luckily, 4-(2-iodophenyl)pyrrole **2** gave under these conditions target heterocyclic skeleton **3a** in 50% yield (Scheme 2). Salt **3a** was characterized by ^1^H, ^13^C NMR and HRMS, its structure was also confirmed by single-crystal X-ray diffraction analysis (Figure 1).

Since iodo-substituted compounds are more expensive and less accessible than bromo-substituted analogs, we tried to accomplish the cyclization of pyridinium salt **1a** using another radical mediator, tris(trimethylsilyl)silane (TTMSS) [31–32], which, moreover, is much less toxic than tributylstannane. Fortunately, free-radical cyclization with TTMSS gave salt **3a** in 74% yield from 4-(2-bromophenyl)pyrrole **1a**, that is comparable with the yield for the 4-(2-iodophenyl)pyrrole **2** cyclization. Protection of pyrrole nitrogen with benzyl, methyl or acetyl PG did not improve the yield of the cyclization product **3a** (Scheme 2). Acetylated compound *N*-Ac-**1a** gives deacetylated product **3a**. The use of 1,1'-azobis(cyclohexanecarbonitrile) or Et_3_B as radical reaction initiators instead of AIBN was unsuccessful.

Therefore, the reaction conditions for the free-radical cyclization of salt **1a** were further optimized by variation of the mediator/initiator ratio, temperature, reaction time, concentration of **1a**, and reagent mixing procedure (Table 1). The optimal conditions found (entry 8, Table 1), characterized by the use of minimum amounts of reagents, were used to evaluate the substrate scope for the synthesis of compounds **3** (Table 2). The developed protocol doesn’t require column chromatography for purification of target compounds **3** and can be performed in a gram scale.

**Table 1:** Optimization of the reaction conditions for the cyclization of **1a** with TTMSS/AIBN in MeCN.

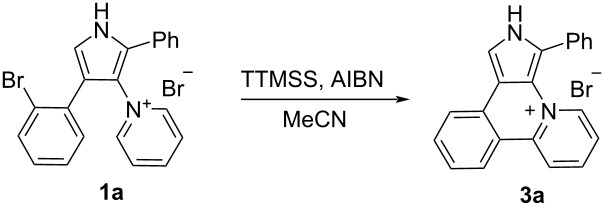					

Entry	TTMSS/AIBN, equiv	*T*, °C, (time, h)	Concentration of **1a**, mg/mL	Reagent mixing procedure^a^	Conversion^b^ of **1a** to **3a**, %

1	3/3	100 (12)	0.7	A	20
2	3/3	100 (12)	10	A	100
3	3/3	100 (12)	7	A	100
4	2/3	100 (12)	7	B	100
5	2/2	100 (6)	10	C	100
6	1.5/3	100 (5)	10	C	100
7	1.7/2.2	85 (5)	10	C	100
8	1.5/2	75 (25)	10	C	100

^a^Method A: slow addition of the TTMSS/AIBN mixture through a syringe pump during all the reaction time; method B: slow addition of AIBN to the **1a**/TTMSS mixture; method C: heating of premixed mixture. ^b^Conversion was determined by ^1^H NMR.

Pyridinium bromides **1a–l,n–w** and iodide **1m** were prepared by the reaction of 3-(2-bromophenyl)-2*H*-azirine (**4a**) with substituted *N*-phenacyl pyridinium salts **5a–w** according to the published method (Table 2) [14]. The free-radical cyclization tolerates 4-F and 4-Cl substituents in the 2-phenyl ring (Table 2, entries 2,3), whereas the reaction of compound **1d**, with a 4-Br substituent in the 2-phenyl ring, led to the formation of a complex mixture of products because of competitive radical reactions. Methyl and methoxy substituents in the 2-phenyl ring (Table 2, entries 5–9) did not affect the cyclization, whereas the presence of 3- or 4-NO_2_-substituents (Table 2, entries 10 and 11) led to tarring of the reaction mixtures probably due to participation of the NO_2_ group in side radical reactions. The fact that this result is not related to the electron-withdrawing nature of this substituent follows from the fact that another strong EWG at the position 4, such as cyano, did not interfere with the reaction, and the product was obtained in good yield. (Table 2, entry 12). It is important that the reaction of iodide **1m** with 2-pyridyl substituent gave a good yield of the product **3m**, which could be used for the preparation of bidentate ligand for metal complexes. Reaction of salts **1** with electron-donating *para*-substituents (Me, MeO, Me_2_N) in the pyridine ring (**1n–p**,**s**) and aryl substituents (**1q**,**r**) afforded the corresponding compounds **3** in 51–82% yield (Table 2, entries 14–19). Heating of isoquinolinium salt **1t** with the TTMSS/AIBN system, disappointingly, led to intensive tarring without the formation of the cyclization product in even trace amounts. Salts with electron-withdrawing *para*-substituents in the pyridine ring (**1u–w**) do not cyclize under used reaction conditions. The reaction of 2*H*-pyrido[2,1-*a*]pyrrolo[3,4-*c*]isoquinolin-4-ium bromides **3** with aq KOH at room temperature gave quantitatively the corresponding bases **6** (Table 2).

**Table 2:** Synthesis of compounds **1**, **3** and **6**.

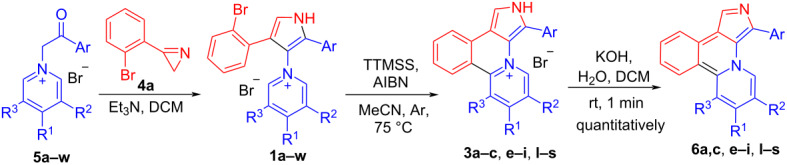					

Entry	Ar	R^1^	R^2^ = R^3^	Yield of **1**, %	Yield of **3**, %

1	Ph	H	H	**a**, 81	**a**, 74
2	4-FC_6_H_4_	H	H	**b**, 72	**b**, 60
3	4-ClC_6_H_4_	H	H	**c**, 80	**c**, 71
4	4-BrC_6_H_4_	H	H	**d**, 79	–^a^
5	4-MeC_6_H_4_	H	H	**e**, 76	**e**, 58
6	4-MeOC_6_H_4_	H	H	**f**, 83	**f**, 70
7	2-MeOC_6_H_4_	H	H	**g**, 63	**g**, 63
8	3-MeOC_6_H_4_	H	H	**h**, 68	**h**, 74
9	2,4-(MeO)_2_C_6_H_3_	H	H	**i**, 56	**i**, 93
10	4-NO_2_C_6_H_4_	H	H	**j**, 72	–^a^
11	3-NO_2_C_6_H_4_	H	H	**k**, 65	–^a^
12	4-NCC_6_H_4_	H	H	**l,** 50	**l**, 73
13	2-Pyridyl^b^	H	H	**m**, 89	**m**, 92
14	Ph	Me	H	**n**, 82	**n**, 77
15	Ph	MeO	H	**o**, 55	**o**, 72
16	Ph	Me_2_N	H	**p**, 51	**p**, 85
17	Ph	Ph	H	**q**, 80	**q**, 53
18	Ph	4-MeOС_6_H_4_	H	**r**, 61	**r**, 71
19	Ph	H	Me	**s**, 73	**s**, 56
20	Ph	R^1^ + R^2^ = (CH=CH)_2_; R^3^ = H		**t**, 40	–^a^
21	Ph	CO_2_Me	H	**u**, 52	–^a^
22	Ph	CN	H	**v**, 64*^c^*	–^a^
23	Ph	Bz	H	**w**, 42^c^	–^a^

^a^Complex mixture of unidentified products.^b^**1m**, **3m**, **5m** - iodides. ^c^NiBr_2_·3H_2_O was used as a base instead of Et_3_N.

**Scheme 3:**
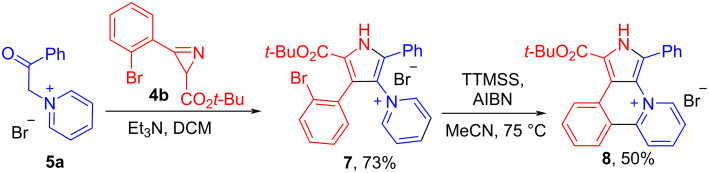
Synthesis of 2*H*-pyrido[2,1-*a*]pyrrolo[3,4-*c*]isoquinolin-4-ium bromide **8**.

Pyridopyrroloisoquinolinium bromide **8**, functionalized with an alkoxycarbonyl group at the C1 position, was also synthesized by the developed approach, starting from azirine **4b** (Scheme 3).

**Scheme 4:**
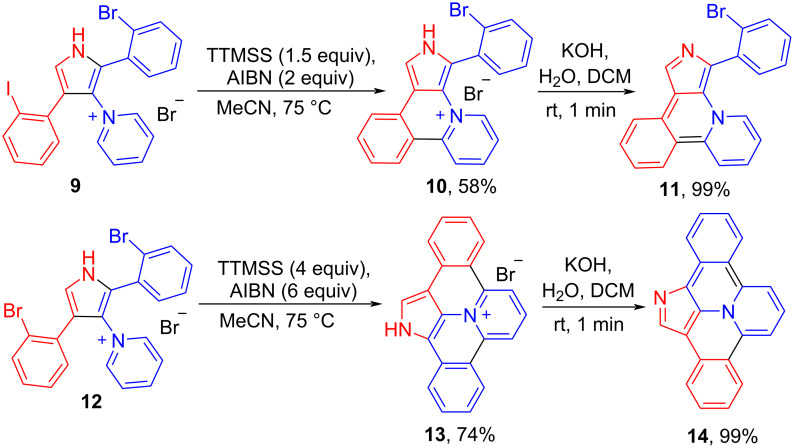
Free-radical cyclization of dihalogeno-substituted salts **9** and **12**.

**Figure 2:**
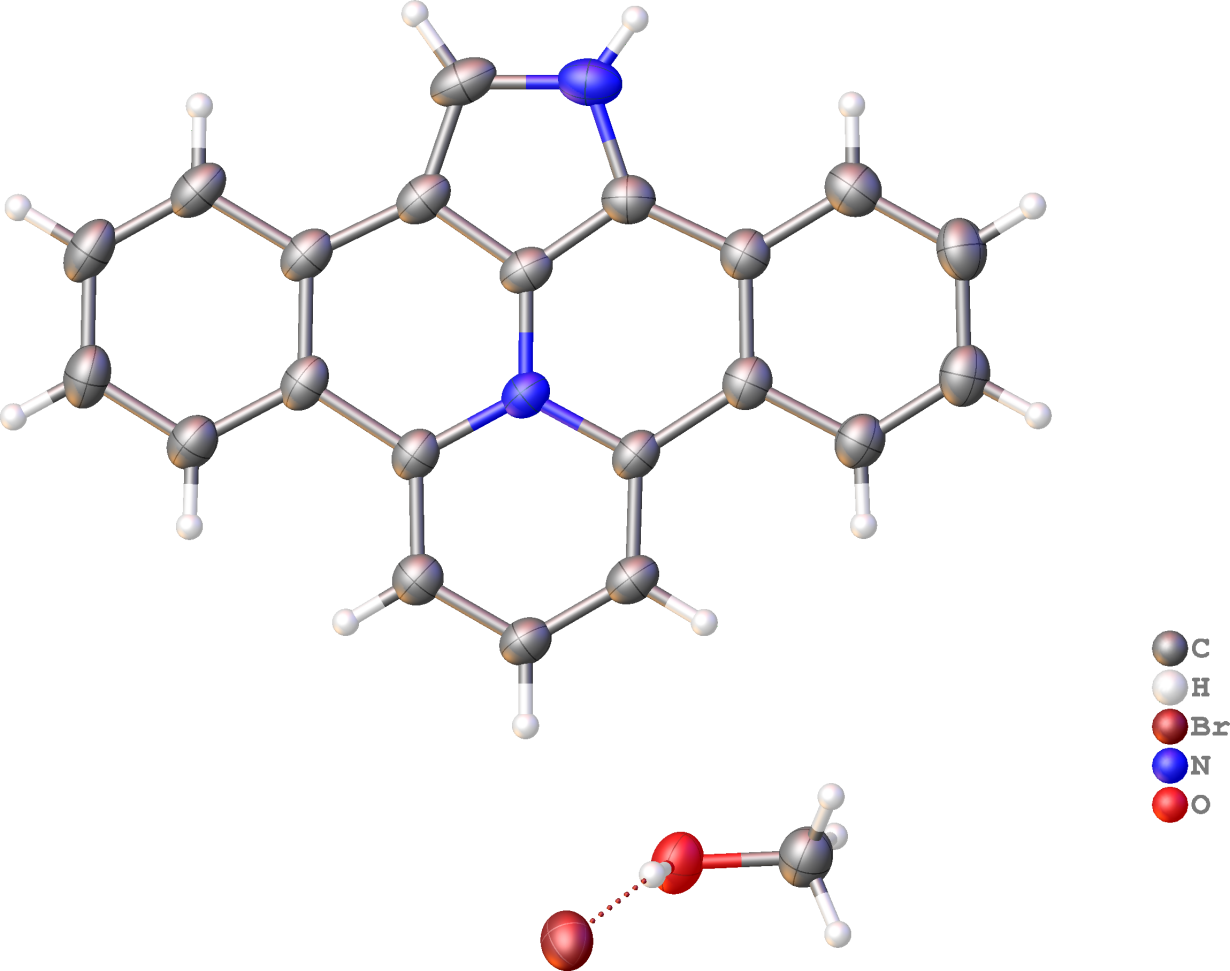
Molecular structure of compound **13**, displacement parameters are drawn at 50% probability level.

Iodo-substituted salt **2** should be more reactive towards cyclization as compared to its bromo-analogue, and this can be used to control the selectivity of intramolecular arylation. Thus compound **10** was obtained from iodo/bromo-substituted salt **9** using 1.5 equiv of TTMSS. Dibromo-substituted salt **12** under the same conditions with 4 equiv of TTMSS gave the product of the double arylation, new heterocyclic skeleton **13**, 1*H*-dibenzo[*b*,*g*]pyrido[2,1,6-*de*]pyrrolo[2,3,4-*ij*]quinolizin-14-ium bromide (Scheme 4). The structure of **13** was confirmed by XRD-analysis (Figure 2). The reaction of bromide **13** with aq KOH at room temperature gave quantitatively the corresponding base **14**. This new 24π-electron hexacyclic phenalenoid doped with two nitrogens is potentially useful material for organic field-effect transistors [1].

**Scheme 5:**
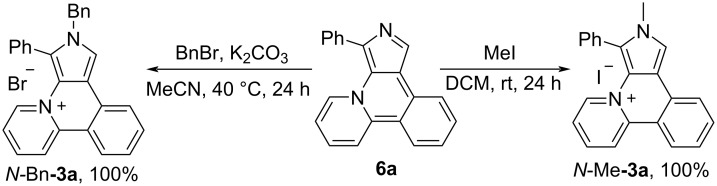
*N*-alkylation of the base **6a**.

Bases **6** can be easily methylated and benzylated on the pyrrole nitrogen with formation of the corresponding salts *N***-**Me**-**and *N***-**Bn**-3a** in quantitative yield (Scheme 5).

Encouraged by the above mentioned results we decided to apply the developed methodology for the preparation of pyrido[2,1-*a*]pyrrolo[3,2-*c*]isoquinoline derivatives **A** (Scheme 1). The previously developed Pd-catalyzed protocol for the preparation of these compounds could be used only for arylation of pyrrole *N*-benzyl protected starting materials **C** [17]. The deprotection of the product could be performed only with AlCl_3_ under harsh conditions and, therefore, cannot be used for the preparation of compounds with acid-sensitive substituents. In addition, the debenzylation of 1-benzyl-3-arylpyrido[2,1-*a*]pyrrolo[3,2-c]isoquinolines **A** was found to accompany by isomerization to 2-aryl derivatives, and therefore 2-unsubstituted bases **A** could not be prepared by a Pd-catalyzed procedure [17].

Optimal reaction conditions, found for the preparation of compounds **3** afforded reasonable results for the synthesis of 3-aryl-substituted heteroaromatics **17** (Table 3). The reaction tolerates various substituents (H, Me, MeO, Ph, 4-MeOC_6_H_4_) at the pyridine moiety of **16**, with the exception of electron-withdrawing groups (CN, Bz). The reaction of 1*H*-pyrido[2,1-*a*]pyrrolo[3,2-*c*]isoquinolin-4-ium bromides **17a,c**–**h** with KOH at room temperature gave quantitatively bases **18a**–**h**.

**Table 3:** Synthesis of compounds **16**–**18**.

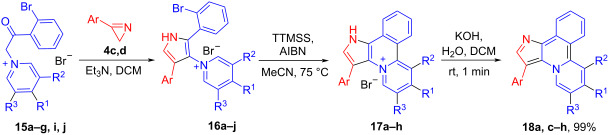					

entry	Ar, azirine	R^1^	R^2^ = R^3^	Yield of **16**, %	Yield of **17**, %
1	Ph**, 4c**	H	H	**a**, 68	**a**, 77
2	Ph**, 4c**	Me	H	**b**, 64	**b**, 46
3	Ph**, 4c**	MeO	H	**c**, 48	**c**, 60
4	Ph**, 4c**	Ph	H	**d**, 53	**d**, 69
5	Ph**, 4c**	4-MeOС_6_H_4_	H	**e**, 95	**e**, 46
6	Ph**, 4c**	H	Me	**f**, 35	**f**, 64
7	Ph**, 4c**	R^1^ + R^2^= (CH=CH)_2_; R^3^ = H		**g**, 44	**g**, 49
8	4-MeOC_6_H_4_**, 4d**	H	H	**h**, 50	**h**, 67
9	Ph**, 4c**	CN	H	**i**, 18^a^	–^b^
10	Ph**, 4c**	Bz	H	**j**, 45^a^	–^b^

^a^NiBr_2_·3H_2_O was used as a base instead of Et_3_N (see Experimental section). ^b^Complex mixture of unidentified products.

**Figure 3:**
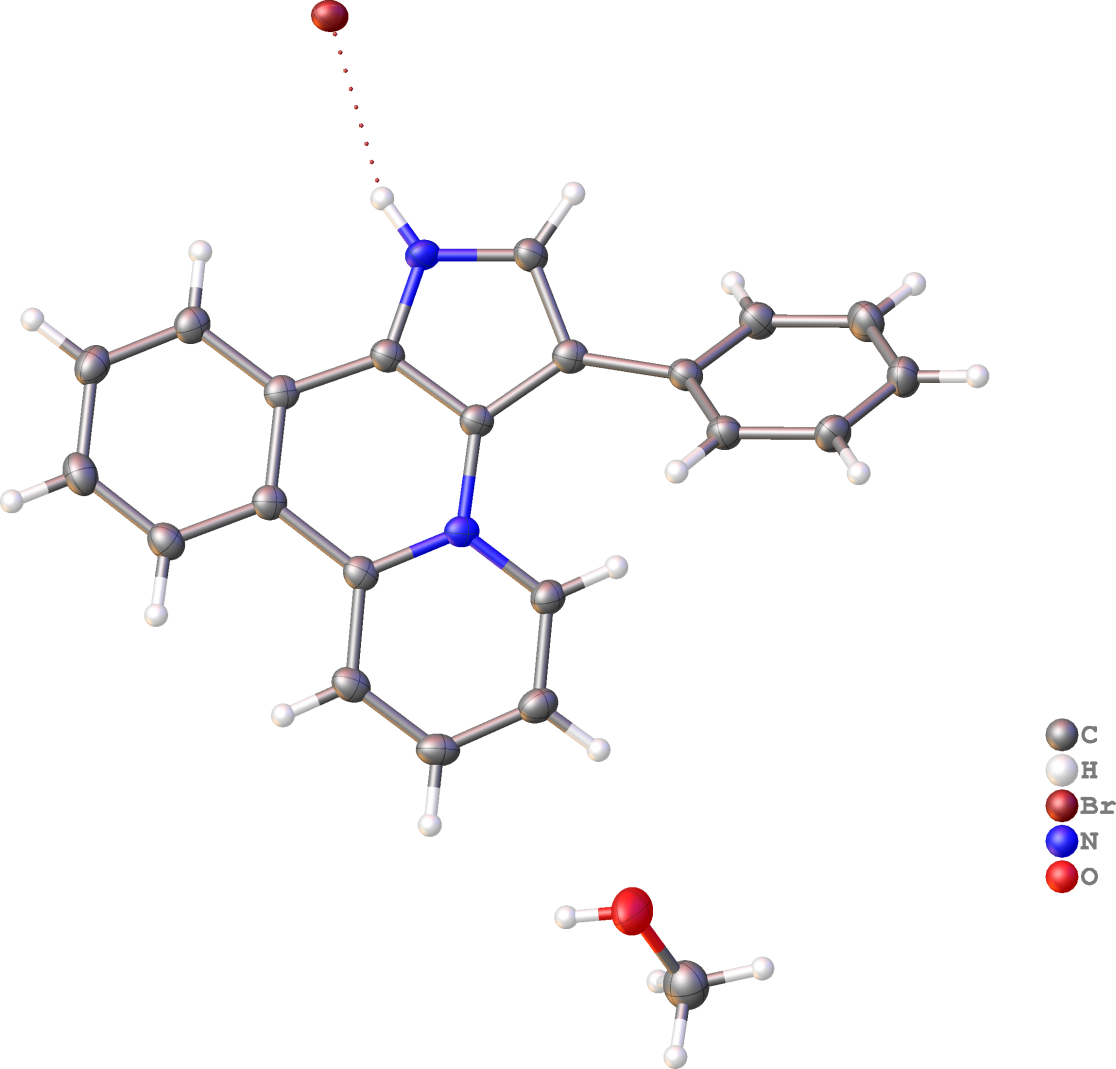
Molecular structure of compound **17a**, displacement parameters are drawn at 50% probability level.

The structure of **17a** was confirmed by XRD-analysis (Figure 3).

**Scheme 6:**
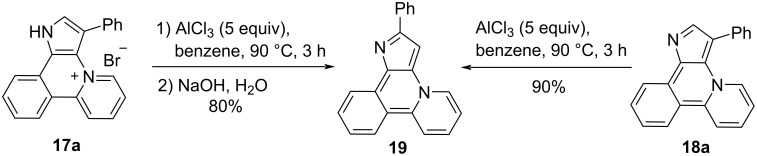
Isomerization of compounds **17a** and **18a**.

It was found that both salt **17a** and base **18a** can be isomerized into isomer **19** under heating in the presence of AlCl_3_ via migration of the 3-Ph-group into the position 2 (Scheme 6).

**Scheme 7:**
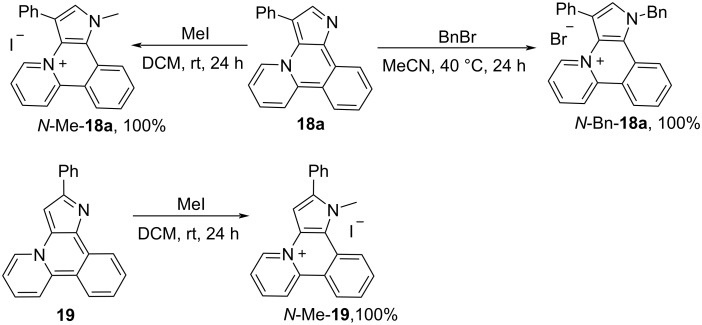
*N*-Alkylation of compounds **18a** and **19**.

Compounds **18a**, **19** can be quantitatively *N*-alkylated by MeI at room temperature and by BnBr at 40 °C (Scheme 7).

A study of photophysical and electrochemical properties of compounds **3** is in progress and will be published elsewhere.

## Conclusion

A wide range of derivatives with new pyrido[2,1-*a*]pyrrolo[3,4-*c*]isoquinoline skeleton was synthesized by free-radical intramolecular cyclization of *o*-bromophenyl-substituted pyrrolylpyridinium salts using the TTMSS/AIBN system. The cyclization provides generally good yields of pyrido[2,1-*a*]pyrrolo[3,4-*c*]isoquinoline hydrobromides having no additional radical-sensitive substituents. The developed protocol does not require column chromatography to purify target compounds and can be performed on a gram scale. The free bases can be obtained from the synthesized hydrobromides in quantitative yield by basification at rt. The use 2/4-(*ortho*-bromo/iodophenyl)-substituted pyrrolylpyridinium bromides allows control over the chemoselectivity of the intramolecular arylation to give the monocyclization product starting from bromo/iodo-substituents or biscyclization product starting from dibromo-derivatives to give the new 24π-electron hexacyclic phenalenoid doped with two nitrogens. The protocol is also applicable for the construction of the pyrido[2,1-*a*]pyrrolo[3,2-*c*]isoquinoline backbone from *N*-unprotected pyrrolylpyridinium salts and, unlike the Pd-catalyzed version cyclization, avoids the deprotection step that is accompanied by isomerization.

## Supporting Information

File 1 Experimental procedures, compound characterization data, X-ray diffraction experiment, and copies of NMR spectra of new compounds.
