# Supplementary material for: Free-radical cyclization approach to polyheterocycles containing pyrrole and pyridine rings
Source: Beilstein J Org Chem. 2021 Jun 23;17:1490–8. doi: 10.3762/bjoc.17.105 (PMC8239278; doi:10.3762/bjoc.17.105)
Supplement: File 1 — Experimental procedures, compound characterization data, X-ray diffraction experiment, and copies of NMR spectra of new compounds. [file Beilstein_J_Org_Chem-17-1490-s001.pdf]

BEILSTEIN JOURNAL OF ORGANIC CHEMISTRY

Supporting Information

for

Free-radical cyclization approach to polyheterocycles containing pyrrole and pyridine rings

Ivan P. Mosiagin, Olesya A. Tomashenko, Dar'ya V. Spiridonova, Mikhail S. Novikov, Sergey P. Tunik and Alexander F. Khlebnikov

Beilstein J. Org. Chem. **2021,** *17,* 1490–1498. doi:10.3762/bjoc.17.105

Experimental procedures, compound characterization data, X-ray diffraction experiment, and copies of NMR spectra of new compounds

License and Terms: This is a supporting information file under the terms of the Creative Commons Attribution License (https://creativecommons.org/licenses/by/4.0). Please note that the reuse, redistribution and reproduction in particular requires that the author(s) and source are credited and that individual graphics may be subject to special legal provisions.
The license is subject to the *Beilstein Journal of Organic Chemistry* terms and conditions: (https://www.beilstein-journals.org/bjoc/terms)

Table of contents

Experimental section ……………………………………………. …..S2

References ………………………………………………….…….S38

Copies of NMR spectra ……………………………………………..S40

X-ray-diffraction experiments……………………………..….…S242

Experimental section

General Information and Methods.

^{1}H (400 or 500 MHz) and ^{13}C (101 or 126 MHz) NMR spectra were recorded on the Bruker AVANCE 400 spectrometer, chemical shift values are reported in ppm on the δ scale relative to TMS (δ = 0.00). ^{1}H NMR spectra were calibrated according to the residual peak of DMSO-d_6 (2.50 ppm). For all new compounds ^{13}C{^{1}H} and ^{13}C DEPT135 were recorded and calibrated according to the peak of DMSO-d_6 (39.52 ppm). Mass spectra were recorded on the mass spectrometer Bruker maXis HRMS-ESI-QTOF, electrospray ionization, positive mode. Single-crystal X-ray data were collected by means of the Rigaku Oxford Diffraction «XtaLAB Supernova» with HyPix 3000 type detector. Crystallographic data for the structures **3a** (CCDC 1974298), **13** (CCDC 2049334), and **17a** (CCDC 2049337) have been deposited with the Cambridge Crystallographic Data Centre. Melting points were determined on melting point apparatus Stuart® SMP30. Thin-layer chromatography (TLC) was conducted on aluminum sheets ALUGRAM SIL G/UV254 with 0.2 mm silica gel (fluorescent indicator). 3-(2-Iodophenyl)-2*H*-azirine [1], azirines **4a,c,d** [1] and azirine **4b**[2] were obtained according to the published procedures. Pyridinium salts **5k,l,n–w** were obtained from the corresponding 2-bromoacetophenones and pyridines in acetone or diethyl ether, salt **5m** was obtained from 2-acetylpyridine and I_2 in pyridine [3]. Salts **1a**, *N*-Me-**1a, 16a,d** were obtained according to the reported procedure [4].

Synthesis of starting materials.

Synthesis of 3-(2-iodophenyl)-2H-azirine

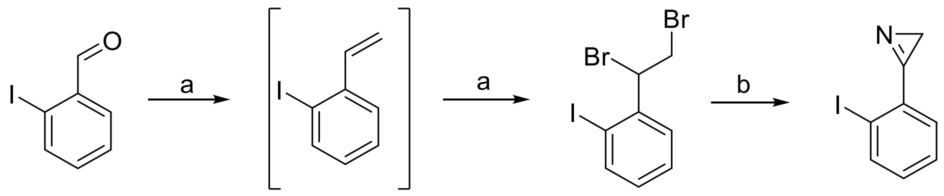

(a) Potassium *tert*-butoxide (3.48 g, 32.0 mmol, 1.75 equiv) was added in one portion to a suspension of methyltriphenylphosphonium bromide (10.4 g, 29.0 mmol, 1.6 equiv) in THF (40 mL). The reaction mixture was stirred for 30 min, cooled to 0 °C, a solution of 2-iodobenzaldehyde (4.25 g, 18.0 mmol) in THF (10 mL) was added from a dropping funnel for 30 min and the reaction mixture was refluxed for 2 h. The completion of the reaction was monitored by TLC analysis. The reaction mixture was cooled, THF was partially evaporated,

dichloromethane was added and the organic layer was washed with saturated aq NH_4Cl and water. The solvents were evaporated and the residue was suspended in petroleum ether, filtrated, the solvent was evaporated and the residue was resolved in DCM (70 mL). The solution was cooled and a cold solution of bromine (3.02 g, 18.9 mmol, 1.05 equiv) in DCM (50 mL) was added dropwise. The reaction mixture was stirred for an additional 30 min at rt, washed with water, 5% aq $Na_2S_2O_3$ and brine. The organic fraction was dried over Na_2SO_4 and the solvent was removed. 1-(1,2-dibromoethyl)-2-iodobenzene was isolated by chromatography on silica gel (petroleum ether). Yield 3.34 g (50%).

White solid, mp 45 °C. ^1H NMR (400 MHz, $CDCl_3$): δ 7.87 (dd, J = 8.0, 1.1 Hz, 1H), 7.52 (dd, J = 7.9, 1.5 Hz, 1H), 7.48–7.36 (m, 1H), 7.03 (td, J = 7.7, 1.6 Hz, 1H), 5.55 (dd, J = 9.6, 6.5 Hz, 1H), 4.18–3.99 (m, 2H). ^{13}C NMR (101 MHz, $CDCl_3$): δ 140.7 (C), 140.0 (CH), 130.6 (CH), 129.1 (CH), 127.7 (CH), 100.8 (C), 53.8 (CH), 34.0 (CH_2). HRMS (ESI) m/z: 388.8032 calcd for $C_8H_8Br_2I^+$ $[M + H]^+$, found 388.8036.

(b) To a solution of 1-(1,2-dibromoethyl)-2-iodobenzene (2.81 g, 7.21 mmol) in DMSO (200 mL) sodium azide (702 mg, 10.8 mmol) was added in several portions. The reaction mixture was stirred at rt for 24 h, then a solution of NaOH (290 mg, 7.21 mmol, 1 equiv) in water (10 mL) was added and stirring was continued for another 24 h. The reaction mixture was poured in 2% aq $NaHCO_3$, the water layer was extracted with DCM, the combined organic layers were washed with brine, dried over Na_2SO_4 and the product was purified by chromatography on Al_2O_3 (petroleum ether). The solvent was the removed, the residue dissolved in toluene (60 mL) and the solution was refluxed for 3 h. After evaporation of toluene the product was used without further purification.

Yield 826 mg (47 %). Yellowish solid, mp 92–94 °C. ^1H NMR (400 MHz, $CDCl_3$): δ 8.03 (dd, J = 8.1, 1.2 Hz, 1H), 7.81 (dd, J = 7.7, 1.7 Hz, 1H), 7.54 (td, J = 7.6, 1.2 Hz, 1H), 7.27 (td, J = 7.7, 1.8 Hz, 1H), 1.95 (s, 2H). ^{13}C NMR (101 MHz, $CDCl_3$): δ 168.6 (C), 140.6 (CH), 133.6 (CH), 132.8 (CH), 128.4 (CH), 128.2 (C), 98.2 (C), 22.3 (CH_2). HRMS (ESI) m/z: 243.9618 calcd for $C_8H_7IN^+$ $[M + H]^+$, found 243.9621.

*1-(1-Benzyl-4-(2-bromophenyl)-2-phenyl-1H-pyrrol-3-yl)pyridin-1-ium bromide (N-Bn-**1a**)*. To a stirred suspension of 4-(2-bromophenyl)-2-phenyl-3-(pyridin-1-ium-1-yl)pyrrol-1-ide [4] (220 mg, 0.59 mmol) and K_2CO_3 (162 mg, 1.17 mmol, 2 equiv) in acetonitrile (10 mL) benzyl bromide (281 mg, 2.05 mmol, 3.5 equiv) was added in one portion, and the reaction mixture was vigorously stirred at rt for 12 h. After the reaction was completed acetonitrile was evaporated

and the residue was suspended in diethyl ether. The precipitate was collected, washed with diethyl ether (3×10 mL) and water (3×5 mL) and dried. Yield 180 mg, 56%. Red solid, mp 193–194 °C (MeCN). ^{1}H NMR (400 MHz, DMSO-d_6): δ 8.86–8.81 (m, 2H), 8.62–8.54 (m, 1H), 8.04 (t, J = 7.1 Hz, 2H), 7.62 (d, J = 8.0 Hz, 1H), 7.53 (s, 1H), 7.47–7.24 (m, 13H), 7.09–7.02 (m, 2H), 5.28 (s, 2H). ^{13}C{1H} NMR (101 MHz, DMSO-d_6): δ 146.74 (CH), 146.68 (CH), 137.1 (C), 132.8 (CH), 131.6 (CH), 130.3 (CH), 130.1 (CH), 129.5 (CH), 129.1 (CH), 128.9 (C), 128.6 (CH), 128.2 (CH), 128.1 (CH), 127.7 (CH), 126.9 (CH), 126.8 (C), 126.4 (C), 124.4 (C), 123.4 (C), 122.2 (CH), 117.7 (C), 50.8 (CH_2). HRMS (ESI) m/z: 465.0961 calcd for $C_{28}H_{22}{}^{79}BrN_2^+$ [M – Br]$^+$, found 465.0971.

*1-(1-Acetyl-4-(2-bromophenyl)-2-phenyl-1H-pyrrol-3-yl)pyridin-1-ium chloride (N-Ac-**1a**)*. To a stirred suspension of 4-(2-bromophenyl)-2-phenyl-3-(pyridin-1-ium-1-yl)pyrrol-1-ide [4] (115 mg, 0.307 mmol) and K_2CO_3 (127 mg, 0.920 mmol, 3 equiv) in dry DCM (6 mL) acetyl chloride (36 mg, 0.46 mmol, 1.5 equiv) was added in one portion, and the reaction mixture was vigorously stirred at rt for 10 min. Then reaction mixture was washed with brine (3×15 mL), dried under Na_2SO_4 and evaporated to dryness. Yield 130 mg, 94%. Yellow-orange solid, mp 285–286 °C (dec., DCM). ^{1}H NMR (400 MHz, DMSO-d_6): δ 8.86–8.73 (m, 2H), 8.60 (tt, J = 7.9, 1.4 Hz, 1H), 8.11–8.01 (m, 2H), 7.60 (d, J = 7.9 Hz, 1H), 7.48–7.35 (m, 8H), 7.28 (ddd, J = 8.0, 5.6, 3.6 Hz, 1H), 3.71 (s, 3H). ^{13}C{1H} NMR (101 MHz, DMSO-d_6): δ 146.7 (CH), 146.6 (CH), 132.9 (CH), 132.8 (CH), 131.6 (C), 130.08 (CH), 130.07 (CH), 130.0 (CH), 129.3 (CH), 129.2 (CH), 128.7 (C), 128.2 (CH), 126.9 (C), 123.9 (C), 123.4 (C), 122.7 (CH), 117.2 (C), 35.2 (CH_3). HRMS (ESI) m/z: 417.0597 calcd for $C_{23}H_{18}{}^{79}BrN_2O^+$ [M – Cl]$^+$, found 417.0602.

General procedure A for the synthesis of 1-(4-(2-bromophenyl)-2-phenyl-1*H*-pyrrol-3-yl)pyridin-1-ium bromides 1, 7, 9, 12 and 16. To a suspension of 2*H*-azirine **4** (1.5 equiv) and pyridinium salt **5** (1 equiv) in DCM triethylamine (1.5 equiv) was added in one portion. The reaction mixture was stirred at room temperature for 24 h (progress was monitored by TLC). The precipitate was filtrated off, washed with small amount of cold DCM and dried. In the case of high solubility of a product in DCM it was isolated by column chromatography on silica gel (DCM/MeOH from 20:1 to 10:1).

General procedure B for the synthesis of bromides 1 and 16 with strong EWD substituent.

To a suspension of 2*H*-azirine (1.5 equiv) and pyridinium salt (1 equiv) in acetone fresh $NiBr_2{\cdot}3H_2O$ (0.2 equiv) was added in one portion. The reaction mixture was heated at 40 °C for

6 h (progress was monitored by TLC). The precipitate was filtrated off, washed with acetone and dried.

*1-(4-(2-Bromophenyl)-2-(4-fluorophenyl)-1H-pyrrol-3-yl)pyridin-1-ium bromide (**1b**).* Compound **1b** (347 mg, 72%) was obtained from 1-(2-(4-fluorophenyl)-2-oxoethyl)pyridin-1-ium bromide [5] (**5b**) (291 mg, 0.98 mmol, 1 equiv), triethylamine (149 mg, 1.48 mmol, 1.5 equiv) and 3-(2-bromophenyl)-*2H*-azirine (**4a**) (289 mg, 1.48 mmol, 1.5 equiv) in DCM (4 mL) according to the general procedure A. Colorless solid, mp 340–341 °C (dec., DCM). ^{1}H NMR (400 MHz, DMSO-d_6): δ 12.52 (s, 1H), 8.91 (d, J = 5.9 Hz, 2H), 8.73–8.67 (m, 1H), 8.18–8.13 (m, 2H), 7.59 (d, J = 7.9 Hz, 1H), 7.45–7.40 (m, 2H), 7.35–7.21 (m, 6H). ^{13}C{1H} NMR (101 MHz, DMSO-d_6): δ 161.8 (d, J = 246.4 Hz, C), 147.0 (CH), 147.0 (CH), 132.8 (d, J = 34.2 Hz, CH), 132.0 (C), 130.0 (CH), 129.3 (CH), 129.2 (CH), 128.6 (CH), 128.1 (CH), 125.5 (C), 124.9 (d, J = 3.2 Hz, C), 123.7 (C), 122.9 (C), 119.2 (C) , 118.8 (CH), 116.3 (d, J = 21.8 Hz, CH). HRMS (ESI) m/z: 393.0397 calcd for $C_{21}H_{15}{}^{79}BrFN_2^+$ [M – Br]$^+$, found 393.0387.

*1-(4-(2-Bromophenyl)-2-(4-chlorophenyl)-1H-pyrrol-3-yl)pyridin-1-ium bromide (**1c**).* Compound **1c** (385 mg, 80%) was obtained from 1-(2-(4-chlorophenyl)-2-oxoethyl)pyridin-1-ium bromide [6] (**5c**) (307 mg, 0.98 mmol, 1 equiv), triethylamine (145 mg, 1.47 mmol, 1.5 equiv) and 3-(2-bromophenyl)-*2H*-azirine (**4a**) (289 mg, 1.47 mmol, 1.5 equiv) in DCM (4 mL) according to the general procedure A. Yellow solid, mp 330–332 °C (dec., DCM). ^{1}H NMR (400 MHz, DMSO-d_6): δ 12.56 (s, 1H), 8.92 (d, J = 5.5 Hz, 2H), 8.70 (t, J = 7.8 Hz, 1H), 8.21–8.11 (m, 2H), 7.60 (d, J = 7.9 Hz, 1H), 7.52–7.38 (m, 4H), 7.35 (s, 1H), 7.32–7.21 (m, 3H). ^{13}C{1H} NMR (101 MHz, DMSO-d_6): δ 147.0 (CH), 147.0 (CH), 133.0 (CH), 132.6 (CH), 131.9 (C), 130.1 (CH), 129.3 (CH), 128.7 (CH), 128.6 (CH), 128.1 (CH), 127.2 (C), 125.1 (C), 123.7 (C), 123.2 (C), 119.4 (C), 119.3 (CH). HRMS (ESI) m/z: 409.0107 calcd for $C_{21}H_{15}{}^{79}BrClN_2^+$ [M – Br]$^+$, found 409.0114.

*1-(4-(2-Bromophenyl)-2-(4-bromophenyl)-1H-pyrrol-3-yl)pyridin-1-ium bromide (**1d**).* Compound **1d** (383 mg, 79%) was obtained from 1-(2-(4-bromophenyl)-2-oxoethyl)pyridin-1-ium bromide [6] (**5d**) (323 mg, 0.90 mmol, 1 equiv), triethylamine (137 mg, 1.36 mmol, 1.5 equiv) and 3-(2-bromophenyl)-*2H*-azirine (**4a**) (266 mg, 1.36 mmol, 1.5 equiv) in DCM (4 mL) according to the general procedure A. Slightly-yellow solid, mp 338–339 °C (dec., DCM). ^{1}H NMR (400 MHz, DMSO-d_6): δ 12.57 (s, 1H), 8.91 (d, J = 5.7 Hz, 2H), 8.70 (t, J = 7.8 Hz, 1H), 8.16 (t, J = 7.1 Hz, 2H), 7.62–7.58 (m, 3H), 7.45–7.38 (m, 2H), 7.35 (s, 1H), 7.28 (ddd, J = 8.8, 5.9, 3.4 Hz, 1H), 7.17 (d, J = 8.5 Hz, 2H). ^{13}C{1H} NMR (101 MHz, DMSO-d_6): δ 147.0 (CH), 146.97 (CH), 133.0 (CH), 132.6 (CH), 132.2 (CH), 131.9 (C), 130.1 (CH), 128.8 (CH), 128.7

(CH), 128.1 (CH), 127.6 (C), 125.2 (C), 123.7 (C), 123.2 (C), 121.6 (C), 119.5 (C), 119.3 (CH). HRMS (ESI) m/z: 454.9577 calcd for $C_{21}H_{16}{}^{79}Br^{81}BrN_2^+$ [M – Br]$^+$, found 454.9591.

*1-(4-(2-Bromophenyl)-2-(p-tolyl)-1H-pyrrol-3-yl)pyridin-1-ium bromide (**1e**).* Compound **1e** (381 mg, 76%) was obtained from 1-(2-oxo-2-(4-methylphenyl)ethyl)pyridin-1-ium bromide [6] (**5e**) (310 mg, 1.06 mmol, 1 equiv), triethylamine (160 mg, 1.59 mmol, 1.5 equiv) and 3-(2-bromophenyl)-*2H*-azirine (**4a**) (311 mg, 1.59 mmol, 1.5 equiv) in DCM (4 mL) according to the general procedure A. Slightly-yellow solid, mp 330–331 °C (dec., DCM). ^{1}H NMR (400 MHz, DMSO-d_6): δ 12.42 (s, 1H), 8.91 (d, J = 5.5 Hz, 2H), 8.69 (t, J = 7.9 Hz, 1H), 8.20–8.11 (m, 2H), 7.59 (d, J = 7.8 Hz, 1H), 7.45–7.39 (m, 2H), 7.30–7.24 (m, 2H), 7.20 (d, J = 8.0 Hz, 2H), 7.10 (d, J = 8.2 Hz, 2H), 2.30 (s, 3H). ^{13}C{1H} NMR (101 MHz, DMSO-d_6): δ 147.1 (C), 146.9 (CH), 137.9 (C), 133.0 (CH), 132.6 (CH), 132.1 (C), 130.0 (CH), 129.8 (CH), 128.6 (CH), 128.1 (CH), 126.7 (CH), 126.5 (C), 125.5 (C), 123.7 (C), 122.7 (C), 119.2 (C), 118.6 (CH), 20.7 (CH_3). HRMS (ESI) m/z: 389.0648 calcd for $C_{22}H_{18}{}^{79}BrN_2^+$ [M – Br]$^+$, found 389.0652.

*1-(4-(2-Bromophenyl)-2-(4-methoxyphenyl)-1H-pyrrol-3-yl)pyridin-1-ium bromide (**1f**).* Compound **1f** (400 mg, 83%) was obtained from 1-(2-(4-methoxyphenyl)-2-oxoethyl)pyridin-1-ium bromide [6] (**5f**) (305 mg, 0.99 mmol, 1 equiv), triethylamine (151 mg, 1.50 mmol, 1.5 equiv) and 3-(2-bromophenyl)-*2H*-azirine (**4a**) (292 mg, 1.49 mmol, 1.5 equiv) in DCM (4 mL) according to the general procedure A. Slightly-yellow solid, mp 294 °C (dec., DCM). ^{1}H NMR (400 MHz, DMSO-d_6): δ 12.35 (s, 1H), 8.90 (d, J = 5.8 Hz, 2H), 8.69 (t, J = 7.7 Hz, 1H), 8.19–8.07 (m, 2H), 7.59 (d, J = 7.8 Hz, 1H), 7.46–7.37 (m, 2H), 7.31–7.25 (m, 2H), 7.16 (d, J = 8.7 Hz, 2H), 6.96 (d, J = 8.8 Hz, 2H), 3.76 (s, 3H). ^{13}C{1H} NMR (101 MHz, DMSO-d_6): δ 159.2 (C), 147.1 (CH), 146.8 (CH), 133.0 (CH), 132.6 (CH), 132.2 (C), 129.9 (CH), 128.5 (CH), 128.4 (CH), 128.1 (CH), 126.4 (C), 123.7 (C), 122.4 (C), 120.6 (C), 119.0 (C), 118.2 (CH), 114.7 (CH), 55.2 (CH_3). HRMS (ESI) m/z: 405.0597 calcd for $C_{22}H_{18}{}^{79}BrN_2O^+$ [M – Br]$^+$, found 405.0593.

*1-(4-(2-Bromophenyl)-2-(2-methoxyphenyl)-1H-pyrrol-3-yl)pyridin-1-ium bromide (**1g**).* Compound **1g** (306 mg, 63%) was obtained from 1-(2-(2-methoxyphenyl)-2-oxoethyl)pyridin-1-ium bromide [6] (**5g**) (305 mg, 0.99 mmol, 1 equiv), triethylamine (151 mg, 1.45 mmol, 1.5 equiv) and 3-(2-bromophenyl)-*2H*-azirine (**4a**) (292 mg, 1.49 mmol, 1.5 equiv) in DCM (4 mL) according to the general procedure A. Yellow solid, mp 264 °C (dec., DCM). ^{1}H NMR (400 MHz, DMSO-d_6): δ 12.30 (br. s, 1H), 8.71 (d, J = 5.7 Hz, 2H), 8.63 (t, J = 7.8 Hz, 1H), 8.08 (t, J = 7.0 Hz, 2H), 7.60 (d, J = 7.9 Hz, 1H), 7.54 (d, J = 7.4 Hz, 1H), 7.51–7.37 (m, 3H), 7.35–7.24 (m, 2H), 7.15–7.09 (m, 1H), 6.96 (d, J = 8.3 Hz, 1H), 3.24 (s, 3H). ^{13}C{1H} NMR (101 MHz,

DMSO-d_6): δ 155.1 (C), 146.1 (CH), 146.0 (CH), 133.1 (CH), 132.7 (CH), 132.2 (C), 130.6 (CH), 130.5 (CH), 129.9 (CH), 128.2 (CH), 128.1 (CH), 124.0 (C), 123.6 (C), 123.5 (C), 121.1 (CH), 119.1 (CH), 118.2 (C), 116.8 (C), 111.4 (CH), 54.8 (CH_3). HRMS (ESI) m/z: 405.0597 calcd for $C_{22}H_{18}{}^{79}BrN_2O^+$ [M – Br]$^+$, found 405.0611.

1-(4-(2-Bromophenyl)-2-(3-methoxyphenyl)-1H-pyrrol-3-yl)pyridin-1-ium bromide ***(1h)***. Compound **1h** (235 mg, 68%) was obtained from 1-(2-(3-methoxyphenyl)-2-oxoethyl)pyridin-1-ium bromide [6] (**5h**) (215 mg, 0.99 mmol, 1 equiv), triethylamine (150 mg, 1.49 mmol, 1.5 equiv) and 3-(2-bromophenyl)-*2H*-azirine (**4a**) (205 mg, 1.49 mmol, 1.5 equiv) in DCM (4 mL) according to the general procedure A. Yellow solid, mp 283–285 °C (dec., DCM). ^{1}H NMR (400 MHz, DMSO-d_6): δ 12.49 (s, 1H), 8.94 (d, J = 5.5 Hz, 2H), 8.70 (t, J = 7.8 Hz, 1H), 8.25–8.04 (m, 2H), 7.60 (d, J = 7.9 Hz, 1H), 7.46–7.38 (m, 2H), 7.33 (s, 1H), 7.32–7.25 (m, 2H), 6.93 (dd, J = 8.2, 2.3 Hz, 1H), 6.85–6.82 (m, 1H), 6.67 (d, J = 7.8 Hz, 1H), 3.70 (s, 3H). ^{13}C{1H} NMR (101 MHz, DMSO-d_6): δ 159.7 (C), 147.2 (CH), 147.0 (CH), 133.0 (CH), 132.6 (CH), 132.0 (C), 130.4 (CH), 130.0 (CH), 129.6 (C), 128.5 (CH), 128.1 (CH), 126.1 (C), 123.7 (C), 123.1 (C), 119.3 (C), 118.9 (CH), 118.7 (CH), 114.0 (CH), 112.2 (CH), 55.1 (CH_3). HRMS (ESI) m/z: 405.0597 calcd for $C_{22}H_{18}{}^{79}BrN_2O^+$ [M – Br]$^+$, found 405.0601.

1-(4-(2-Bromophenyl)-2-(2,4-dimethoxyphenyl)-1H-pyrrol-3-yl)pyridin-1-ium bromide ***(1i)***. Compound **1i** (279 mg, 56%) was obtained from 1-(2-(2,4-dimethoxyphenyl)-2-oxoethyl)pyridin-1-ium bromide (**5i**) (327 mg, 0.97 mmol, 1 equiv), triethylamine (147 mg, 1.45 mmol, 1.5 equiv) and 3-(2-bromophenyl)-*2H*-azirine (**4a**) (283 mg, 1.45 mmol, 1.5 equiv) in DCM (4 mL) according to the general procedure A. Bright-yellow solid, mp 250–251 °C (dec., DCM). ^{1}H NMR (400 MHz, DMSO-d_6): δ 12.16 (br. s, 1H), 8.67 (d, J = 5.5 Hz, 2H), 8.62(t, J = 7.8 Hz, 1H), 8.07 (t, J = 7.8 Hz, 2H), 7.59 (d, J = 7.9 Hz, 1H), 7.54–7.37 (m, 3H), 6.71 (dd, J = 8.5, 2.4 Hz, 1H), 6.49 (d, J = 2.4 Hz, 1H), 3.79 (s, 3H), 3.24 (s, 3H). ^{13}C{1H} NMR (101 MHz, DMSO-d_6): δ 161.3 (C), 156.4 (C), 146.1 (CH), 145.8 (CH), 133.1 (CH), 132.7 (CH), 132.3 (C), 131.4 (CH), 129.8 (CH), 128.2 (CH), 128.0 (CH), 123.6 (C), 123.46 (C), 118.5 (CH), 118.0 (C), 109.3 (C), 106.1 (CH), 98.4 (CH), 55.4 (CH_3), 54.9 (CH_3). HRMS (ESI) m/z: 435.0703 calcd for $C_{23}H_{20}{}^{79}BrN_2O_2^+$ [M – Br]$^+$, found 435.0693.

1-(4-(2-Bromophenyl)-2-(4-nitrophenyl)-1H-pyrrol-3-yl)pyridin-1-ium bromide ***(1j)***. Compound **1j** (350 mg, 72%) was obtained from 1-(2-(4-nitrophenyl)-2-oxoethyl)pyridin-1-ium bromide [6] (**5j**) (311 mg, 0.96 mmol, 1 equiv), triethylamine (146 mg, 1.45 mmol, 1.5 equiv) and 3-(2-bromophenyl)-*2H*-azirine (**4a**) (284 mg, 1.45 mmol, 1.5 equiv) in DCM (4 mL) according to the general procedure A. Orange-yellow solid, mp > 400 °C (DCM). ^{1}H NMR (400 MHz, DMSO-

d_6): δ 12.86 (s, 1H), 8.99 (d, J = 5.5 Hz, 2H), 8.75 (t, J = 7.9 Hz, 1H), 8.28–8.14 (m, 4H), 7.61 (d, J = 7.8 Hz, 1H), 7.51–7.39 (m, 5H), 7.35–7.23 (m, 1H). $^{13}C\{1H\}$ NMR (101 MHz, DMSO-d_6): δ 147.4 (C), 147.0 (CH), 146.4 (C), 134.8 (C), 133.0 (CH), 132.7 (CH), 131.5 (C), 130.2 (CH), 128.8 (CH), 128.1 (CH), 127.5 (CH), 124.5 (CH), 124.0 (C), 123.7 (C), 120.8 (CH), 120.3 (C). HRMS (ESI) m/z: 420.0342 calcd for $C_{21}H_{15}{}^{79}BrN_3O_2^+$ [M – Br]$^+$, found 420.0349.

*1-(4-(2-Bromophenyl)-2-(3-nitrophenyl)-1H-pyrrol-3-yl)pyridin-1-ium bromide **(1k)***. Compound **1k** (315 mg, 65%) was obtained from 1-(2-(3-nitrophenyl)-2-oxoethyl)pyridin-1-ium bromide [7] (**5k**) (311 mg, 0.96 mmol, 1 equiv), triethylamine (146 mg, 1.45 mmol, 1.5 equiv) and 3-(2-bromophenyl)-*2H*-azirine (**4a**) (283 mg, 1.45 mmol, 1.5 equiv) in DCM (4 mL) according to the general procedure A. Colorless solid, mp 319–320 °C (dec., DCM). ^1H NMR (400 MHz, DMSO-d_6): δ 12.80 (s, 1H), 8.98 (d, J = 5.6 Hz, 2H), 8.73 (t, J = 7.8 Hz, 1H), 8.18 (t, J = 7.1 Hz, 3H), 8.10 (s, 1H), 7.71–7.54 (m, 3H), 7.48–7.39 (m, 3H), 7.35–7.26 (m, 1H). $^{13}C\{1H\}$ NMR (101 MHz, DMSO-d_6): δ 148.2 (CH), 147.3 (C), 147.0 (CH), 133.0 (CH), 132.9 (CH), 132.7 (CH), 130.9 (CH), 130.2 (CH), 130.0 (C), 128.8 (CH), 128.2 (CH), 124.0 (C), 123.9 (C), 123.7 (C), 122.7 (CH), 121.4 (CH), 120.0 (CH). HRMS (ESI) m/z: 420.0342 calcd for $C_{21}H_{15}{}^{79}BrN_3O_2^+$ [M – Br]$^+$, found 420.0352.

*1-(4-(2-Bromophenyl)-2-(4-cyanophenyl)-1H-pyrrol-3-yl)pyridin-1-ium bromide **(1l)***. Compound **1l** (252 mg, 50%) was obtained from 1-(2-(4-cyanophenyl)-2-oxoethyl)pyridin-1-ium bromide [6] (**5l**) (315 mg, 1.04 mmol, 1 equiv), triethylamine (136 mg, 1.56 mmol, 1.5 equiv) and 3-(2-bromophenyl)-*2H*-azirine (**4a**) (264 mg, 1.56 mmol, 1.5 equiv) in DCM (10 mL) according to the general procedure A. Beige solid, mp 332–333 °C (dec., DCM). ^1H NMR (400 MHz, DMSO-d_6): δ 12.76 (br. s, 1H), 9.00–8.91 (m, 2H), 8.73 (td, J = 7.9, 1.6 Hz, 1H), 8.22–8.13 (m, 2H), 7.90–7.81 (m, 2H), 7.60 (dd, J = 8.3, 3.5 Hz, 1H), 7.49–7.34 (m, 5H), 7.33–7.23 (m, 1H). $^{13}C\{1H\}$ NMR (101 MHz, DMSO-d_6): δ 147.4 (CH), 147.0 (CH), 133.1 (CH), 133.0 (CH), 132.9 (C), 132.7 (CH), 131.6 (C), 130.2 (CH), 128.8 (CH), 128.1 (CH), 127.2 (CH), 124.4 (C), 124.1 (C), 123.7 (C), 120.4 (CH), 120.1 (C), 118.4 (C), 110.3 (C). HRMS (ESI) m/z: 400.0444 calcd for $C_{22}H_{25}{}^{79}BrN_3^+$ [M – Br]$^+$, found 400.0432.

*1-(4-(2-Bromophenyl)-2-(pyridin-2-yl)-1H-pyrrol-3-yl)pyridin-1-ium iodide **(1m)***. Compound **1m** (503 mg, 89%) was obtained from 1-(2-oxo-2-(pyridin-2-yl)ethyl)pyridin-1-ium iodide [3] (**5m**) (411 mg, 1.26 mmol, 1 equiv), triethylamine (190 mg, 1.90 mmol, 1.5 equiv) and 3-(2-bromophenyl)-*2H*-azirine (**4a**) (372 mg, 1.90 mmol, 1.5 equiv) in DCM (6 mL) according to the general procedure A. Slightly-yellow solid, mp 287–288 °C (DCM). ^1H NMR (400 MHz, DMSO-d_6): δ 12.74 (s, 1H), 9.12–9.03 (m, 2H), 8.72–8.67 (m, 1H), 8.22–8.20 (m, 1H), 8.17–

8.09 (m, 2H), 7.90 (td, J = 7.8, 1.9 Hz, 1H), 7.77–7.73 (m, 1H), 7.60 (dd, J = 8.0, 1.2 Hz, 1H), 7.48–7.36 (m, 3H), 7.31–7.21 (m, 2H). ^{13}C{1H} NMR (101 MHz, DMSO-d_6): δ 149.2 (CH), 148.1 (C), 147.7 (CH), 146.9 (CH), 137.6 (CH), 132.9 (CH), 132.6 (CH), 131.7 (C), 130.1 (CH), 127.9 (CH), 127.5 (CH), 124.6 (C), 123.8 (C), 122.6 (CH), 120.4 (C), 119.8 (CH), 119.4 (CH). HRMS (ESI) m/z: 376.0449 calcd for $C_{20}H_{15}{}^{79}BrN_3{}^+$ [M – Br]$^+$, found 376.0445.

1-(4-(2-Bromophenyl)-2-phenyl-1H-pyrrol-3-yl)-4-methylpyridin-1-ium bromide ***(1n)***. Compound **1n** (397 mg, 82%) was obtained from 4-methyl-1-(2-oxo-2-phenylethyl)pyridin-1-ium bromide [8] (**5n**) (300 mg, 1.03 mmol, 1 equiv), triethylamine (155 mg, 1.52 mmol, 1.5 equiv) and 3-(2-bromophenyl)-*2H*-azirine (**4a**) (302 mg, 1.54 mmol, 1.5 equiv) in DCM (4 mL) according to the general procedure A. Grey solid, mp 256–257 °C (dec., DCM). ^{1}H NMR (400 MHz, DMSO-d_6): δ 12.44 (s, 1H), 8.78 (d, J = 6.7 Hz, 2H), 7.97 (d, J = 6.4 Hz, 2H), 7.60 (d, J = 7.8 Hz, 1H), 7.45–7.24 (m, 7H), 7.23–7.18 (m, 2H), 2.63 (s, 3H). ^{13}C{1H} NMR (101 MHz, DMSO-d_6): δ 160.8 (C), 146.0 (CH), 132.9 (CH), 132.6 (CH), 132.2 (C), 130.0 (CH), 129.3 (CH), 128.8 (CH), 128.5 (C), 128.2 (CH), 128.0 (CH), 126.6 (CH), 126.2 (C), 123.8 (C), 122.6 (C), 119.4 (C), 118.7 (CH), 21.7 (CH_3). HRMS (ESI) m/z: 389.0648 calcd for $C_{22}H_{18}{}^{79}BrN_2{}^+$ [M – Br]$^+$, found 389.0654.

1-(4-(2-Bromophenyl)-2-phenyl-1H-pyrrol-3-yl)-4-methoxypyridin-1-ium bromide ***(1o)***. Compound **1o** (277 mg, 55%) was obtained from 4-methoxy-1-(2-oxo-2-phenylethyl)pyridin-1-ium bromide [8] (**5o**) (317 mg, 1.03 mmol, 1 equiv), triethylamine (155 mg, 1.54 mmol, 1.5 equiv) and 3-(2-bromophenyl)-*2H*-azirine (**4a**) (302 mg, 1.54 mmol, 1.5 equiv) in DCM (5 mL) according to the general procedure A. Yellow solid, mp 328–330 °C (dec., DCM). ^{1}H NMR (400 MHz, DMSO-d_6): δ 12.32 (br. s, 1H), 8.73 (d, J = 7.0 Hz, 2H), 7.67–7.66 (m, 3H), 7.45–7.32 (m, 5H), 7.30–7.25 (m, 2H), 7.24–7.17 (m, 2H), 4.11 (s, 3H). ^{13}C{1H} NMR (101 MHz, DMSO-d_6): δ 171.41 (C), 148.38 (CH), 132.9 (CH), 132.6 (CH), 132.4 (C), 129.9 (CH), 129.3 (CH), 128.7 (C), 128.04 (CH), 127.95 (CH), 126.4 (CH), 126.1 (C), 123.9 (C), 122.2 (C), 119.6 (C), 118.5 (CH), 113.9 (CH), 58.4 (CH_3). HRMS (ESI) m/z: 405.0597 calcd for $C_{22}H_{18}{}^{79}BrN_2O^+$ [M – Br]$^+$, found 405.0617.

1-(4-(2-Bromophenyl)-2-phenyl-1H-pyrrol-3-yl)-4-(dimethylamino)pyridin-1-ium bromide ***(1p)***. Compound **1p** (1.109 g, 51%) was obtained from 4-(dimethylamino)-1-(2-oxo-2-phenylethyl)pyridin-1-ium bromide [6] (**5p**) (680 mg, 2.12 mmol, 1 equiv), triethylamine (320 mg, 3.18 mmol, 1.5 equiv) and 3-(2-bromophenyl)-*2H*-azirine (**4a**) (624 mg, 3.18 mmol, 1.5 equiv) in DCM (10 mL) according to the general procedure A. Colorless solid, mp 313 °C (DCM). ^{1}H NMR (400 MHz, DMSO-d_6): δ 12.16 (s, 1H), 8.08 (d, J = 7.6 Hz, 2H), 7.64 (d, J =

8.0 Hz, 1H), 7.44–7.19 (m, 8H), 7.18 (s, 1H), 6.97 (d, *J* = 7.6 Hz, 2H), 3.18 (s, 6H). ^{13}C{1H} NMR (101 MHz, DMSO-d_6): δ 155.6 (C), 144.0 (CH), 133.1 (C), 132.7 (CH), 132.6 (CH), 129.7 (CH), 129.3 (C), 129.1 (CH), 127.8 (CH), 127.7 (CH), 126.1 (CH), 125.7 (C), 124.0 (C), 122.3 (C), 119.9 (C), 118.1 (CH), 107.8 (CH), 39.9 (CH_3). HRMS (ESI) m/z: вычислено 418.0913, $C_{23}H_{21}{}^{79}BrN_3^+$ [M – Br]$^+$, найдено 418.0907.

*1-(4-(2-Bromophenyl)-2-phenyl-1H-pyrrol-3-yl)-4-phenylpyridin-1-ium bromide (**1q**).* Compound **1q** (480 mg, 80%) was obtained from 1-1-(2-oxo-2-phenylethyl)-4-phenylpyridin-1-ium bromide [8] (**5q**) (400 mg, 1.13 mmol, 1 equiv), triethylamine (172 mg, 1.70 mmol, 1.5 equiv) and 3-(2-bromophenyl)-*2H*-azirine (**4a**) (332 mg, 1.70 mmol, 1.5 equiv) in DCM (10 mL) according to the general procedure A. Orange solid, mp 335 °C (dec., DCM). ^{1}H NMR (400 MHz, DMSO-d_6): δ 12.50 (s, 1H), 8.92 (d, *J* = 7.0 Hz, 2H), 8.53 (d, *J* = 7.0 Hz, 2H), 8.13 (d, *J* = 7.2 Hz, 2H), 7.71–7.55 (m, 4H), 7.50–7.24 (m, 9H). ^{13}C{1H} NMR (101 MHz, DMSO-d_6): δ 155.3 (C), 146.9 (CH), 133.0 (C), 132.8 (CH), 132.72 (C), 132.70 (CH), 132.2 (C), 130.0 (CH), 129.7 (CH), 129.3 (CH), 128.52 (C), 128.41 (CH), 128.2 (CH), 128.1 (CH), 126.8 (CH), 126.4 (C), 124.5 (CH), 123.8 (C), 119.4 (C), 119.0 (CH). HRMS (ESI) m/z: 451.0804 calcd for $C_{27}H_{20}{}^{79}BrN_2^+$ [M – Br]$^+$, found 451.0817.

*1-(4-(2-Bromophenyl)-2-phenyl-1H-pyrrol-3-yl)-4-(4-methoxyphenyl)pyridin-1-ium bromide (**1r**).* Compound **1r** (306 mg, 61%) was obtained from 4-(4-methoxyphenyl)-1-(2-oxo-2-phenylethyl)pyridin-1-ium bromide (**5r**) (342 mg, 0.89 mmol, 1 equiv), triethylamine (135 mg, 1.34 mmol, 1.5 equiv) and 3-(2-bromophenyl)-*2H*-azirine (**4a**) (261 mg, 1.34 mmol, 1.5 equiv) in DCM (10 mL) according to the general procedure A. Yellow solid, mp 192–193 °C (DCM). ^{1}H NMR (400 MHz, DMSO-d_6): δ 12.42 (br. s, 1H), 8.86–8.70 (m, 2H), 8.51–8.38 (m, 2H), 8.20–8.08 (m, 2H), 7.62 (dd, *J* = 8.1, 1.2 Hz, 1H), 7.50–7.24 (m, 9H), 7.21–7.13 (m, 2H), 3.88 (s, 3H). ^{13}C{1H} NMR (101 MHz, DMSO-d_6): δ 163.3 (C), 154.6 (C), 146.4 (CH), 132.9 (CH), 132.7 (CH), 132.3 (C), 130.5 (CH), 130.0 (CH), 129.3 (CH), 128.6 (C), 128.2 (CH), 128.0 (C), 126.7 (CH), 126.3 (C), 124.6 (C), 123.8 (C), 123.0 (CH), 122.5 (C), 119.4 (C), 118.8 (CH), 115.3 (CH), 55.8 (CH_3). HRMS (ESI) m/z: 481.0910 calcd for $C_{28}H_{22}{}^{79}BrN_2O^+$ [M – Br]$^+$, found 481.0898.

*1-(4-(2-Bromophenyl)-2-phenyl-1H-pyrrol-3-yl)-3,5-dimethylpyridin-1-ium bromide (**1s**).* Compound **1s** (367 mg, 73%) was obtained from 3,5-dimethyl-1-(2-oxo-2-phenylethyl)pyridin-1-ium bromide [9] (**5s**) (316 mg, 1.03 mmol, 1 equiv), triethylamine (156 mg, 1.55 mmol, 1.5 equiv) and 3-(2-bromophenyl)-*2H*-azirine (**4a**) (304 mg, 1.55 mmol, 1.5 equiv) in DCM (6 mL) according to the general procedure A. Bright yellow solid, mp 317–318 °C (DCM). ^{1}H NMR

(400 MHz, DMSO-d_6): δ 12.44 (br. s, 1H), 8.74 (s, 2H), 8.42 (s, 1H), 7.60 (d, J = 7.9 Hz, 1H), 7.45–7.32 (m, 5H), 7.32–7.23 (m, 2H), 7.20 (d, J = 6.9 Hz, 2H), 2.34 (s, 6H). ^{13}C{1H} NMR (101 MHz, DMSO-d_6): δ 147.9 (CH), 143.9 (CH), 138.4 (C), 132.8 (CH), 132.6 (C), 132.1 (C), 129.9 (CH), 129.2 (CH), 128.5 (C), 128.2 (CH), 127.9 (CH), 126.3 (CH), 126.0 (C), 123.8 (C), 122.9 (C), 119.6 (C), 118.7 (CH), 17.4 (CH_3). HRMS (ESI) m/z: 403.0804 calcd for $C_{23}H_{20}{}^{79}BrN_2^+$ [M – Br]$^+$, found 403.0818.

*2-(4-(2-Bromophenyl)-2-phenyl-1H-pyrrol-3-yl)isoquinolin-2-iumbromide (**1t**).* Compound **1t** (240 mg, 40%) was obtained from 2-(2-oxo-2-phenylethyl)isoquinolin-2-ium bromide [8] (**5t**) (389 mg, 1.19 mmol, 1 equiv), triethylamine (180 mg, 1.78 mmol, 1.5 equiv) and 3-(2-bromophenyl)-*2H*-azirine (**4a**) (349 mg, 1.78 mmol, 1.5 equiv) in DCM (6 mL) according to the general procedure A. Orange solid, mp 266–268 °C (DCM). ^{1}H NMR (400 MHz, DMSO-d_6):δ 12.48 (d, J = 3.4 Hz, 1H), 10.02 (d, J = 1.4 Hz, 1H), 8.63–8.53 (m, 2H), 8.40–8.35 (m, 2H), 8.35–8.25 (m, 1H), 8.07–8.05 (m, 1H), 7.55–7.47 (m, 2H), 7.42–7.30 (m, 5H), 7.26–7.18 (m, 3H). ^{13}C{1H} NMR (101 MHz, DMSO-d_6):δ 152.0 (CH), 138.1 (CH), 137.2 (CH), 137.0 (C), 133.0 (CH), 132.6 (CH), 132.1 (C), 131.7 (CH), 130.7 (CH), 130.0 (CH), 129.3 (CH), 128.5 (C), 128.3 (CH), 128.1 (CH), 127.5 (CH), 127.3 (C), 126.7 (CH), 126.5 (C), 126.2 (CH), 123.6 (C), 123.1 (C), 119.7 (C), 119.0 (CH). HRMS (ESI) m/z: 425.0648 calcd for $C_{25}H_{18}{}^{79}BrN_2^+$ [M – Br]$^+$, found 425.0660.

*1-(4-(2-Bromophenyl)-2-phenyl-1H-pyrrol-3-yl)-4-(methoxycarbonyl)pyridin-1-ium bromide (**1u**).* Compound **1u** (246 mg, 52%) was obtained from 4-(methoxycarbonyl)-1-(2-oxo-2-phenylethyl)pyridin-1-ium bromide (**5u**) (309 mg, 0.920 mmol, 1 equiv), triethylamine (147 mg, 1.46 mmol, 1.5 equiv) and 3-(2-bromophenyl)-*2H*-azirine (**4a**) (286 mg, 1.46 mmol, 1.5 equiv) in DCM (4 mL) according to the general procedure A. Red solid, mp 290–291 °C (dec., DCM). ^{1}H NMR (400 MHz, DMSO-d_6): δ 12.52 (s, 1H), 9.06–8.97 (m, 2H), 8.47–8.39 (m, 2H), 7.60 (d, J = 8.1 Hz, 1H), 7.47–7.33 (m, 6H), 7.31–7.23 (m, 3H), 3.95 (s, 3H). ^{13}C{1H} NMR (101 MHz, DMSO-d_6): δ 162.2 (C), 148.1 (CH), 144.5 (C), 133.1 (CH), 132.7 (CH), 131.7 (C), 130.1 (CH), 129.4 (CH), 128.5 (CH), 128.3 (CH), 128.0 (C), 127.6 (CH), 127.0 (CH), 126.7 (C), 123.6 (C), 122.7 (C), 119.43 (CH), 119.17 (C), 53.80 (CH_3). HRMS (ESI) m/z: 433.0546 calcd for $C_{23}H_{18}{}^{79}BrN_2O_2^+$ [M – Br]$^+$, found 433.0564.

*1-(4-(2-Bromophenyl)-2-phenyl-1H-pyrrol-3-yl)-4-cyanopyridin-1-ium bromide (**1v**).* Compound **1v** (152 mg, 64%) was obtained from 4-cyano-1-(2-oxo-2-phenylethyl)pyridin-1-ium bromide [10] (**5v**) (150 mg, 0.495 mmol, 1 equiv), $NiBr_2{\cdot}3H_2O$ (27 mg, 0.10 mmol, 0.2 equiv) and 3-(2-bromophenyl)-*2H*-azirine (**4a**) (145 mg, 0.74 mmol, 1.5 equiv) in acetone (10 mL) according to

the general procedure B. Red solid, mp 301–302 °C (dec., acetone). ^{1}H NMR (400 MHz, DMSO-d_6): δ 12.61 (s, 1H), 9.17 (d, J = 6.2 Hz, 2H), 8.66 (d, J = 6.3 Hz, 2H), 7.61 (d, J = 8.0 Hz, 1H), 7.51–7.21 (m, 9H). ^{13}C{1H} NMR (101 MHz, DMSO-d_6): δ 147.8 (CH), 133.1 (CH), 132.7 (CH), 131.5 (CH), 130.0 (CH), 129.3 (CH), 128.5 (CH), 128.2 (CH), 127.83 (C), 127.78 (C), 127.1 (CH), 126.9 (C), 123.5 (C), 122.6 (C), 119.5 (CH), 119.1 (C), 114.6 (C). HRMS (ESI) m/z: 400.0444 calcd for $C_{22}H_{15}{}^{79}BrN_3^+$ [M – Br]$^+$, found 400.0449.

*4-Benzoyl-1-(4-(2-bromophenyl)-2-phenyl-1H-pyrrol-3-yl)pyridin-1-ium bromide (**1w**).* Compound **1w** (155 mg, 42%) was obtained from 4-benzoyl-1-(2-oxo-2-phenylethyl)pyridin-1-ium bromide (**5w**) (250 mg, 0.654 mmol, 1 equiv), $NiBr_2 \cdot 3H_2O$ (43 mg, 0.13 mmol, 0.2 equiv) and 3-(2-bromophenyl)-*2H*-azirine (**4a**) (192 mg, 0.98 mmol, 1.5 equiv) in acetone (20 mL) according to the general procedure B. Bright red solid, mp 280–282 °C (acetone). ^{1}H NMR (400 MHz, DMSO-d_6): δ 12.55 (br. s, 1H), 9.08 (d, J = 6.6 Hz, 2H), 8.34 (d, J = 6.6 Hz, 2H), 7.84–7.77 (m, 3H), 7.68–7.62 (m, 3H), 7.51–7.34 (m, 8H), 7.34–7.28 (m, 1H). ^{13}C{1H} NMR (101 MHz, DMSO-d_6): δ 192.6 (C), 152.1 (C), 147.7 (CH), 134.8 (CH), 134.0 (C), 133.2 (CH), 132.8 (CH), 131.8 (C), 130.12 (CH), 130.08 (CH), 129.4 (CH), 129.1 (CH), 128.5 (CH), 128.3 (CH) 128.2 (C), 127.5 (CH), 127.1 (CH), 126.7 (C), 123.6 (C), 122.7 (C), 119.38 (CH), 119.29 (C). HRMS (ESI) m/z: 479.0754 calcd for $C_{28}H_{20}{}^{79}BrN_2O^+$ [M – Br]$^+$, found 479.0774.

*1-(4-(2-Iodophenyl)-2-phenyl-1H-pyrrol-3-yl)pyridin-1-ium bromide (**2**).* Compound **2** (272 mg, 75%) was obtained from 1-(2-oxo-2-phenylethyl)pyridin-1-ium bromide (**5a**) (200 mg, 0.72 mmol, 1 equiv), triethylamine (109 mg, 1.08 mmol, 1.5 equiv) and 3-(2-iodophenyl)-*2H*-azirine (262 mg, 1.08 mmol, 1.5 equiv) in DCM (4 mL) according to the general procedure A. Yellow solid, mp 318–320 °C (DCM). ^{1}H NMR (400 MHz, DMSO-d_6): δ 12.41 (s, 1H), 8.96–8.84 (m, 2H), 8.67 (tt, J = 7.9, 1.4 Hz, 1H), 8.20–8.09 (m, 2H), 7.93–7.78 (m, 1H), 7.43–7.32 (m, 5H), 7.28 (d, J = 2.9 Hz, 1H), 7.23–7.19 (m, 2H), 7.08 (ddd, J = 7.9, 6.5, 2.5 Hz, 1H). ^{13}C{1H} NMR (101 MHz, DMSO-d_6): δ 147.1 (CH), 147.0 (CH), 139.0 (CH), 136.2 (C), 131.9 (CH), 130.0 (CH), 129.3 (CH), 128.6 (CH), 128.5 (CH), 128.4 (C), 128.3 (CH), 126.6 (CH), 126.1 (C), 122.8 (C), 122.7 (C), 118.7 (CH), 101.6 (C). HRMS (ESI) m/z: 423.0353 calc for $C_{21}H_{16}IN_2^+$ [M – Br] $^+$, found 423.0368.

*1-(1-Benzyl-4-(2-iodophenyl)-2-phenyl-1H-pyrrol-3-yl)pyridin-1-ium bromide (N-Bn-**2**).* Compound *N-Bn-**2*** (339 mg, 73%) was obtained from 4-(2-iodophenyl)-2-phenyl-3-(pyridin-1-ium-1-yl)pyrrol-1-ide (331 mg, 0.78 mmol), benzyl bromide (469 mg, 2.74 mmol, 3.5 equiv) and K_2CO_3 (216 mg, 1.57 mmol, 2 equiv) in acetonitrile (12 mL) likewise *N*-Bn-**1**. Yellow solid, mp 220–222 °C (MeCN). ^{1}H NMR (400 MHz, DMSO-d_6): δ 8.79 (d, J = 6.0 Hz, 2H), 8.56 (t, J =

7.9 Hz, 1H), 8.04 (t, *J* = 7.0 Hz, 2H), 7.89 (d, *J* = 7.9 Hz, 1H), 7.49 (s, 1H), 7.46–7.22 (m, 10H), 7.14–7.02 (m, 3H), 5.30 (s, 2H). ^{13}C{1H} NMR (101 MHz, DMSO-d_6): δ 146.73 (CH), 146.70 (CH), 139.1 (CH), 137.2 (C), 135.8 (C), 131.7 (CH), 130.2 (CH), 130.0 (CH), 129.4 (CH), 129.1 (CH), 128.61 (CH), 128.59 (CH), 128.1 (CH), 127.6 (CH), 126.8 (C), 126.7 (CH), 124.2 (CH), 122.1 (C), 121.0 (C), 101.3 (C), 50.7 (CH_2). HRMS (ESI) m/z: 513.0822 calcd for $C_{28}H_{22}IN_2^+$ $[M – Br]^+$, found 513.0839.

General procedure C for the radical cyclization with TTMSS/AIBN. To a suspension of pyrrolylpyridinium bromide (1 equiv) and AIBN (2 equiv) in dry MeCN (C_{salt} = 10 mg/mL) in a screw top vial TTMSS (1.5 equiv) was added. Then argon was bubbled through the reaction mixture for 15 min. Then vial was screwed and reaction mixture was vigorously stirred at 75 °C for 25 h (the completion of the reaction was checked by NMR of the reaction mixture). Then it was cooled down, ethyl acetate (MeCN/EtOAc, v/v 1:6) was added. The solution was kept in a refrigerator for 2–3 h, the precipitate was collected, washed with ethyl acetate and hexane and dried.

*3-Phenylpyrido[2,1-a]pyrrolo[3,4-c]isoquinolin-2-ium bromide (**3a**).* Compound **3a** (122 mg, 74%) was obtained from 1-(4-(2-bromophenyl)-2-phenyl-1*H*-pyrrol-3-yl)pyridin-1-ium bromide (**1a**) (201 mg, 0.44 mmol, 1 equiv), TTMSS (163 mg, 0.65 mmol, 1.5 equiv) and AIBN (144 mg, 0.87 mmol, 2 equiv) according to the general procedure C. Orange solid, mp > 400 °C (MeCN/EtOAc). ^{1}H NMR (400 MHz, DMSO-d_6): δ 13.14 (br. s, 1H), 9.39 (d, *J* = 8.5 Hz, 1H), 9.13 (d, *J* = 6.6 Hz, 1H), 8.92 (d, *J* = 8.5 Hz, 1H), 8.53–8.47 (m, 1H), 8.46 (d, *J* = 7.9 Hz, 1H), 8.34 (d, *J* = 3.1 Hz, 1H), 8.02–7.97 (m, 2H), 7.76–7.61 (m, 6H). ^{13}C{1H} NMR (101 MHz, DMSO-d_6): δ 142.7 (C), 140.0 (CH), 135.0 (CH),133.9 (CH), 130.7 (C), 130.2 (CH), 129.6 (CH), 129.51 (C), 129.45 (CH), 127.1 (CH), 126.7 (CH), 124.4 (CH), 124.1 (CH), 123.1 (CH), 121.6 (C), 120.9 (C), 117.5 (C), 113.9 (C), 112.5 (CH). HRMS (ESI) m/z: 295.1230 calcd for $C_{21}H_{15}N_2^+$ $[M – Br]^+$, found 295.1242.

Gram scale experiment. Compound **3a** (736 mg, 88%) was obtained from 1-(4-(2-bromophenyl)-2-phenyl-1*H*-pyrrol-3-yl)pyridin-1-ium bromide (**1a**) (1 g, 2.19 mmol, 1 equiv), TTMSS (819 mg, 3.29 mmol, 1.5 equiv) and AIBN (718 mg, 4.38 mmol, 2 equiv) according to the general procedure C.

2-Methyl-3-phenylpyrido[2,1-a]pyrrolo[3,4-c]isoquinolin-4-ium iodide (*N*-Me-**3a**). Compound *N*-Me-**3a** (62 mg, 73%) was obtained from 1-(4-(2-bromophenyl)-1-methyl-2-phenyl-1*H*-pyrrol-3-yl)pyridin-1-ium iodide (*N*-Me-**1a**) (100 mg, 0.19 mmol, 1 equiv), TTMSS (71 mg, 0.28

mmol, 1.5 equiv) and AIBN (62 mg, 0.38 mmol, 2 equiv) according to the general procedure C. Compound *N*-Me-**3a** (15 mg, 100%) was obtained from 3-phenylpyrido[2,1-a]pyrrolo[3,4-c]isoquinoline (**6a**) (10 mg, 0.035 mmol) and methyl iodide (5 mg, 0.035 mmol, 1 equiv) in dry DCM (2 mL). The reaction mixture was stirred for 24 h and evaporated. The product was dried under vacuum. Orange solid, mp 279–281 °C (MeCN/EtOAc). ^{1}H NMR (400 MHz, DMSO-d_6): δ 9.38 (d, J = 8.7 Hz, 1H), 8.92 (d, J = 8.5 Hz, 1H), 8.81 (d, J = 6.7 Hz, 1H), 8.50–8.45 (m, 1H), 8.40 (s, 1H), 8.38 (d, J = 11.5 Hz, 1H), 8.04–7.97 (m, 1H), 7.95–7.88 (m, 1H), 7.78–7.63 (m, 6H), 3.71 (s, 3H). ^{13}C{1H} NMR (101 MHz, DMSO-d_6): δ 142.6 (C), 139.8 (CH), 134.3 (CH), 134.1 (CH), 131.3 (CH), 130.5 (CH), 130.0 (CH), 129.3 (C), 128.9 (C), 127.2 (CH), 126.8 (CH), 124.4 (CH), 124.1 (CH), 122.8 (CH), 122.7 (C), 120.8 (C), 118.3 (C), 116.1 (CH), 112.3 (C), 36.2 (CH_3). HRMS (ESI) m/z: 309.1387 calcd for $C_{22}H_{17}N_2^+$ [M – I]$^+$, found 309.1383.

*2-Benzyl-3-phenylpyrido[2,1-a]pyrrolo[3,4-c]isoquinolin-4-ium bromide (N-Bn-**3a**).* Compound N-Bn-**3a** (54 mg, 64% from N-Bn-**1a**; 56 mg, 67% from N-Bn-**2**) was obtained from 1-(1-benzyl-4-(2-bromophenyl)-2-phenyl-1*H*-pyrrol-3-yl)pyridin-1-ium bromide (N-Bn-**1a**) (100 mg, 0.18 mmol, 1 equiv) or 1-(1-benzyl-4-(2-iodophenyl)-2-phenyl-1*H*-pyrrol-3-yl)pyridin-1-ium bromide (N-Bn-**2**) (107 mg, 0.18 mmol, 1 equiv), TTMSS (68 mg, 0.27 mmol, 1.5 equiv) and AIBN (60 mg, 0.37 mmol, 2 equiv) according to the general procedure C.

3-Phenylpyrido[2,1-a]pyrrolo[3,4-c]isoquinoline (**6a**) (14.5 mg, 0.05 mmol), benzyl bromide (9 mg, 0.05 mmol, 1 equiv) and K_2CO_3 (7 mg, 0.10 mmol, 2 equiv) was mixed in dry acetonitrile (4 mL). The reaction mixture was heated at 40 °C for 24 h, evaporated. The residue was washed with water and Et_2O and dried under vacuum to obtain compound N-Bn-**3a** (23 mg, 100%). Yellow to brown solid, mp 249–250 °C (dec., MeCN/EtOAc). ^{1}H NMR (400 MHz, $CDCl_3$): δ 9.47 (d, J = 8.8 Hz, 1H), 8.83–8.74 (m, 2H), 8.68 (d, J = 1.4 Hz, 1H), 8.12 (dd, J = 8.0, 1.3 Hz, 1H), 7.99 (s, 1H), 7.83–7.73 (m, 2H), 7.70–7.60 (m, 4H), 7.48–7.42 (m, 2H), 7.32–7.27 (m, 3H), 7.02–6.96 (m, 2H), 5.23 (s, 2H). ^{13}C{1H} NMR (126 MHz, $CDCl_3$): δ 143.1 (C), 140.9 (CH), 135.8 (C), 134.3 (CH), 133.8 (CH), 131.7 (CH), 131.4 (CH), 130.6 (CH), 129.14 (C), 129.1 (CH), 129.0 (C), 128.5 (CH), 128.0 (C), 127.5 (CH), 127.1 (CH), 125.7 (CH), 124.6 (CH), 123.3 (CH), 123.2 (C), 120.8 (C), 118.8 (C), 115.2 (CH), 113.6 (C), 53.0 (CH_2). HRMS (ESI) m/z: 385.1699 calcd for $C_{28}H_{21}N_2^+$ [M – Br]$^+$, found 385.1711.

*3-(4-Fluorophenyl)pyrido[2,1-a]pyrrolo[3,4-c]isoquinolin-2-ium bromide (**3b**).* Compound **3b** (80 mg, 60%) was obtained from 1-(4-(2-bromophenyl)-2-(4-fluorophenyl)-1*H*-pyrrol-3-yl)pyridin-1-ium bromide (**1b**) (160 mg, 0.34 mmol, 1 equiv), TTMSS (126 mg, 0.506 mmol, 1.5 equiv) and AIBN (111 mg, 0.676 mmol, 2 equiv) according to the general procedure C. Orange

solid, mp 321–322 °C (MeCN/EtOAc). ^{1}H NMR (400 MHz, DMSO-d_6): δ 13.13 (s, 1H), 9.39 (d, J = 8.8 Hz, 1H), 9.08 (d, J = 6.8 Hz, 1H), 8.91 (d, J = 8.5 Hz, 1H), 8.57–8.41 (m, 2H), 8.33 (d, J = 3.2 Hz, 1H), 8.03–7.94 (m, 2H), 7.82–7.69 (m, 3H), 7.55–7.47 (m, 2H). ^{13}C{1H} NMR (126 MHz, DMSO-d_6): δ 162.8 (d, J = 247.2 Hz, C), 142.8 (C), 140.0 (CH), 135.3 (CH), 134.0 (CH), 132.7 (CH), 132.6 (CH), 129.6 (C), 127.1 (d, J = 3.1 Hz, C), 127.0 (d, J = 59.2 Hz, CH), 124.4 (CH), 124.3 (CH), 123.1 (CH), 121.0 (C), 120.6 (C), 117.8 (C), 116.5 (d, J = 21.7 Hz, CH), 113.9 (C), 112.4 (CH). HRMS (ESI) m/z: 313.1136 calcd for $C_{21}H_{14}FN_2^+$ [M – Br]$^+$, found 313.1148.

*3-(4-Chlorophenyl)pyrido[2,1-a]pyrrolo[3,4-c]isoquinolin-2-ium bromide (**3c**).* Compound **3c** (118 mg, 71%) was obtained from 1-(4-(2-bromophenyl)-2-(4-chlorophenyl)-1*H*-pyrrol-3-yl)pyridin-1-ium bromide (**1c**) (200 mg, 0.41 mmol, 1 equiv), TTMSS (152 mg, 0.61 mmol, 1.5 equiv) and AIBN (133 mg, 0.82 mmol, 2 equiv) according to the general procedure C. Orange solid, mp > 400 °C (MeCN/EtOAc). ^{1}H NMR (400 MHz, DMSO-d_6): δ 13.16 (s, 1H), 9.39 (d, J = 8.8 Hz, 1H), 9.11 (d, J = 6.6 Hz, 1H), 8.92 (d, J = 8.5 Hz, 1H), 8.56–8.50 (m, 1H), 8.46 (d, J = 7.9 Hz, 1H), 8.36 (d, J = 3.3 Hz, 1H), 8.05–7.95 (m, 2H), 7.74 (s, 5H). ^{13}C{1H} NMR (101 MHz, DMSO-d_6): δ 142.8 (CH), 140.1 (C), 135.5 (CH), 134.3 (C), 134.0 (CH), 132.0 (CH), 129.52 (CH), 129.48 (C), 127.3 (CH), 126.8 (CH), 124.33 (CH), 124.31 (CH), 123.1 (CH), 121.0 (C), 120.4 (C), 117.9 (C), 114.1 (C), 112.8 (CH). HRMS (ESI) m/z: 329.0845 calcd for $C_{21}H_{14}ClN_2^+$ [M – Br]$^+$, found 329.0849.

*3-(4-Methylphenyl)pyrido[2,1-a]pyrrolo[3,4-c]isoquinolin-2-ium bromide (**3e**).* Compound **3e** (96 mg, 58%) was obtained from 1-(4-(2-bromophenyl)-2-(4-methylphenyl)-1*H*-pyrrol-3-yl)pyridin-1-ium bromide (**1e**) (200 mg, 0.42 mmol, 1 equiv), TTMSS (158 mg, 0.64 mmol, 1.5 equiv) and AIBN (139 mg, 0.85 mmol, 2 equiv) according to the general procedure C. Pale yellow solid, mp > 400 °C (MeCN/EtOAc). ^{1}H NMR (400 MHz, DMSO-d_6): δ 13.13–13.01 (m, 1H), 9.41–9.32 (m, 1H), 9.16 (dd, J = 6.8, 1.3 Hz, 1H), 8.89 (d, J = 8.4 Hz, 1H), 8.53–8.47 (m, 1H), 8.43 (d, J = 8.7Hz, 1H), 8.47–8.41 (m, 1H), 8.30 (d, J = 3.2 Hz, 1H), 8.02–7.93 (m, 2H), 7.75–7.68 (m, 1H), 7.63–7.57 (m, 2H), 7.47 (d, J = 7.9 Hz, 2H), 2.47 (s, 3H). ^{13}C{1H} NMR (101 MHz, DMSO-d_6): δ 142.7 (C), 140.0 (CH), 135.0 (CH), 133.9 (CH), 130.7 (C), 130.2 (CH), 129.6 (CH), 129.51 (C), 129.5 (CH), 127.1 (CH), 126.7 (CH), 124.4 (CH), 124.1 (CH), 123.1 (CH), 121.6 (C), 120.9 (C), 117.5 (C), 113.9 (C), 112.5 (CH), 21.0 (CH_3). HRMS (ESI) m/z: 309.1386 calc for $C_{22}H_{17}N_2^+$ [M – Br]$^+$, found 309.1382.

*3-(4-Methoxyphenyl)pyrido[2,1-a]pyrrolo[3,4-c]isoquinolin-2-ium bromide (**3f**).* Compound **3f** (116 mg, 70%) was obtained from 1-(4-(2-bromophenyl)-2-(4-methoxyphenyl)-1*H*-pyrrol-3-

yl)pyridin-1-ium bromide (**1f**) (200 mg, 0.41 mmol, 1 equiv), TTMSS (153 mg, 0.62 mmol, 1.5 equiv) and AIBN (135 mg, 0.82 mmol, 2 equiv) according to the general procedure C. Red solid, mp > 400 °C (MeCN/EtOAc). ^{1}H NMR (400 MHz, DMSO-d_6): δ 13.03 (s, 1H), 9.37 (d, J = 8.7 Hz, 1H), 9.18 (d, J = 6.7 Hz, 1H), 8.90 (d, J = 8.5 Hz, 1H), 8.53–8.41 (m, 2H), 8.28 (d, J = 3.2 Hz, 1H), 8.01–7.94 (m, 2H), 7.76–7.69 (m, 1H), 7.66–7.59 (m, 2H), 7.25–7.18 (m, 2H), 3.89 (s, 3H). ^{13}C{1H} NMR (101 MHz, DMSO-d_6): δ 160.0 (C), 142.5 (C), 139.7 (CH), 134.7 (CH), 133.8 (CH), 131.6 (CH), 129.5 (C), 127.0 (CH), 126.6 (CH), 124.3 (CH), 124.1 (CH), 123.0 (CH), 122.5 (C), 121.5 (C), 120.8 (C), 117.2 (C), 114.9 (CH), 113.6 (C), 111.9 (CH), 55.4 (CH_3). HRMS (ESI) m/z: 325.1340 calcd for $C_{22}H_{17}N_2O^+$ [M – Br]$^+$, found 325.1347.

*3-(2-Methoxyphenyl)pyrido[2,1-a]pyrrolo[3,4-c]isoquinolin-2-ium bromide (**3g**)*. Compound **3g** (42 mg, 63%) was obtained from 1-(4-(2-bromophenyl)-2-(2-methoxyphenyl)-1*H*-pyrrol-3-yl)pyridin-1-ium bromide (**1g**) (80 mg, 0.16 mmol, 1 equiv), TTMSS (61 mg, 0.25 mmol, 1.5 equiv) and AIBN (54 mg, 0.33 mmol, 2 equiv) according to the general procedure C. Orange solid, mp 240 °C (dec., MeCN/EtOAc). ^{1}H NMR (400 MHz, DMSO-d_6): δ 13.06 (br. s, 1H), 9.38 (d, J = 8.7 Hz, 1H), 8.91 (d, J = 8.4 Hz, 1H), 8.83–8.77 (m, 1H), 8.56–8.48 (m, 1H), 8.44 (d, J = 7.9 Hz, 1H), 8.34 (d, J = 3.3 Hz, 1H), 8.05–7.96 (m, 2H), 7.76–7.68 (m, 1H), 7.67–7.60 (m, 2H), 7.32 (d, J = 8.2 Hz, 1H), 7.28–7.23 (m, 1H), 3.65 (s, 3H). ^{13}C{1H} NMR (126 MHz, DMSO-d_6): δ 156.6 (C), 142.6 (C), 139.7 (CH), 135.9 (CH), 134.0 (CH), 131.8 (CH), 131.6 (CH), 129.6 (C), 127.2 (CH), 126.8 (CH), 123.9 (CH), 123.8 (CH), 123.1 (CH), 121.3 (CH), 120.9 (C), 119.0 (C), 118.5 (C), 117.8 (C), 113.7 (C), 112.6 (CH), 112.3 (CH), 55.4 (CH_3). HRMS (ESI) m/z: 325.1335 calcd for $C_{22}H_{16}N_2O^+$ [M – Br]$^+$, found 325.1331.

*3-(3-Methoxyphenyl)pyrido[2,1-a]pyrrolo[3,4-c]isoquinolin-2-ium bromide (**3h**)*. Compound **3h** (122 mg, 74%) was obtained from 1-(4-(2-bromophenyl)-2-(3-methoxyphenyl)-1*H*-pyrrol-3-yl)pyridin-1-ium bromide (**1h**) (200 mg, 0.412 mmol, 1 equiv), TTMSS (154 mg, 0.617 mmol, 1.5 equiv) and AIBN (135 mg, 0.823 mmol, 2 equiv) according to the general procedure C. Pale orange solid, mp 230–231 °C (dec., MeCN/EtOAc). ^{1}H NMR (400 MHz, DMSO-d_6): δ 13.14 (s, 1H), 9.38 (d, J = 8.8 Hz, 1H), 9.18 (d, J = 6.7 Hz, 1H), 8.90 (d, J = 8.4 Hz, 1H), 8.54–8.49 (m, 1H), 8.44 (d, J = 7.9 Hz, 1H), 8.32 (d, J = 3.3 Hz, 1H), 8.04–7.91 (m, 2H), 7.74–7.69 (m, 1H), 7.60–7.55 (m, 1H), 7.34–7.24 (m, 2H), 7.19 (dd, J = 8.4, 2.6 Hz, 1H), 3.84 (s, 3H). ^{13}C{1H} NMR (126 MHz, DMSO-d_6): δ 159.8 (C), 142.8 (C), 140.0 (CH), 135.4 (CH), 133.9 (CH), 131.9 (C), 130.7 (CH), 129.5 (C), 127.2 (CH), 126.7 (CH), 124.3 (CH), 124.1 (CH), 123.1 (CH), 122.2 (CH), 121.5 (C), 121.0 (C), 117.5 (C), 115.5 (CH), 115.4 (CH), 114.0, 112.4 (CH), 55.4 (CH_3). HRMS (ESI) m/z: 325.1335 calcd for $C_{22}H_{16}N_2O^+$ [M – Br]$^+$, found 325.1339.

*3-(2,4-Dimethoxyphenyl)pyrido[2,1-a]pyrrolo[3,4-c]isoquinolin-2-ium bromide (**3i**).* Compound **3i** (160 mg, 93%) was obtained from 1-(4-(2-bromophenyl)-2-(2,4-dimethoxyphenyl)-1*H*-pyrrol-3-yl)pyridin-1-ium bromide (**1i**) (200 mg, 0.388 mmol, 1 equiv), TTMSS (145 mg, 0.581 mmol, 1.5 equiv) and AIBN (127 mg, 0.775 mmol, 2 equiv) according to the general procedure C. Orange solid, mp 212–213 °C (MeCN/EtOAc). ^{1}H NMR (400 MHz, DMSO-d_6): δ 12.96 (s, 1H), 9.36 (d, J = 8.7 Hz, 1H), 8.94–8.81 (m, 2H), 8.54–8.48 (m, 1H), 8.42 (dd, J = 8.1, 1.2 Hz, 1H), 8.28 (d, J = 3.2 Hz, 1H), 8.08–7.99 (m, 1H), 8.00–7.93 (m, 1H), 7.74–7.67 (m, 1H), 7.57–7.50 (m, 1H), 6.88–6.80 (m, 2H), 3.91 (s, 3H), 3.65 (s, 3H). ^{13}C{1H} NMR (101 MHz, DMSO-d_6): δ 162.2 (C), 157.9 (C), 142.5 (C), 139.6 (CH), 135.6 (CH), 133.9 (CH), 132.6 (CH), 129.7 (C), 127.1 (CH), 126.8 (CH), 123.9 (CH), 123.1 (CH), 120.9 (C), 118.2 (C), 117.9 (C), 113.5 (C), 112.1 (CH), 111.3 (C), 106.2 (CH), 99.3 (CH), 55.6 (CH_3), 55.5 (CH_3). HRMS (ESI) m/z: 355.1441 calcd for $C_{23}H_{18}N_2O_2^+$ [M – Br]$^+$, found 355.1458.

*3-(4-Cyanophenyl)pyrido[2,1-a]pyrrolo[3,4-c]isoquinolin-2-ium bromide (**3l**).* Compound **3l** (122 mg, 73%) was obtained from 1-(4-(2-bromophenyl)-2-(4-cyanophenyl)-1*H*-pyrrol-3-yl)pyridin-1-ium bromide (**1l**) (200 mg, 0.416 mmol, 1 equiv), TTMSS (155 mg, 0.624 mmol, 1.5 equiv) and AIBN (137 mg, 0.832 mmol, 2 equiv) according to the general procedure C. Orange solid, mp > 400 °C (MeCN/EtOAc). ^{1}H NMR (500 MHz, DMSO-d_6): δ 13.38–13.26 (m, 1H), 9.41 (d, J = 8.7 Hz, 1H), 9.05 (d, J = 6.6 Hz, 1H), 8.92 (d, J = 8.5 Hz, 1H), 8.59–8.52 (m, 1H), 8.51–8.40 (m, 2H), 8.13 (d, J = 8.2 Hz, 2H), 8.01–7.90 (m, 4H), 7.78–7.73 (m, 1H). ^{13}C{1H} NMR (126 MHz, DMSO-d_6): δ 143.0 (C), 140.4 (CH), 136.0 (CH), 135.2 (C), 134.0 (CH), 133.2 (CH), 130.8 (CH), 129.3 (C), 127.4 (CH), 126.7 (CH), 124.38 (C), 124.35 (CH), 123.1 (CH), 121.1 (C), 119.9 (C), 118.6 (C), 118.4 (C), 114.5 (C), 113.8 (CH), 111.7 (C). HRMS (ESI) m/z: 320.1182 calcd for $C_{22}H_{14}N_3^+$ [M – Br]$^+$, found 320.1178.

*3-(Pyridin-2-yl)pyrido[2,1-a]pyrrolo[3,4-c]isoquinolin-2-ium iodide (**3m**).* Compound **3m** (160 mg, 92%) was obtained from 1-(4-(2-bromophenyl)-2-(pyridin-2-yl)-1*H*-pyrrol-3-yl)pyridin-1-ium iodide (**1m**) (200 mg, 0.49 mmol, 1 equiv), TTMSS (153 mg, 0.62 mmol, 1.5 equiv) and AIBN (135 mg, 0.82 mmol, 2 equiv) according to the general procedure C. Orange solid, mp > 400 °C (MeCN/EtOAc). ^{1}H NMR (400 MHz, DMSO-d_6): δ 11.57 (d, J = 6.7 Hz, 1H), 9.18 (dd, J = 8.8, 1.5 Hz, 1H), 8.73 (d, J = 8.4 Hz, 1H), 8.57 (dd, J = 5.0, 1.8 Hz, 1H), 8.29–8.19 (m, 3H), 8.06 (s, 1H), 7.94–7.88 (m, 1H), 7.83–7.77 (m, 2H), 7.51–7.45 (m, 1H), 7.22–7.17 (m, 1H). ^{13}C{1H} NMR (101 MHz, DMSO-d_6): δ 156.8 (C), 147.2 (CH), 139.5 (C), 138.6 (CH), 136.4 (CH), 135.0 (CH), 132.8 (CH), 131.0 (C), 127.9(C), 125.9 (CH), 124.5 (CH), 123.4 (CH), 123.0

(CH), 122.66 (CH), 122.20 (CH), 122.08 (CH), 120.9 (C), 119.8 (CH), 118.9 (C), 116.7 (C). HRMS (ESI) m/z: 296.1182 calcd for $C_{20}H_{14}N_3^+$ [M – I]$^+$, found 296.1186.

*7-Methyl-3-phenylpyrido[2,1-a]pyrrolo[3,4-c]isoquinolin-2-ium bromide (**3n**).* Compound **3n** (128 mg, 77%) was obtained from 1-(4-(2-bromophenyl)-2-phenyl-1*H*-pyrrol-3-yl)-4-methylpyridin-1-ium bromide (**1n**) (200 mg, 0.425 mmol, 1 equiv), TTMSS (158 mg, 0.64 mmol, 1.5 equiv) and AIBN (140 mg, 0.85 mmol, 2 equiv) according to the general procedure C. Orange solid, mp > 400 °C (MeCN/EtOAc). ^{1}H NMR (400 MHz, DMSO-d_6): δ 13.03 (s, 1H), 9.28–9.24 (m, 1H), 8.95 (d, J = 6.9 Hz, 1H), 8.90 (d, J = 8.4 Hz, 1H), 8.42 (dd, J = 8.0, 1.3 Hz, 1H), 8.29 (d, J = 3.2 Hz, 1H), 7.99–7.92 (m, 1H), 7.84 (dd, J = 7.0, 1.8 Hz, 1H), 7.75–7.61 (m, 6H), 2.69 (s, 3H). ^{13}C{1H} NMR (101 MHz, DMSO-d_6): δ 152.9 (C), 142.1 (C), 134.1 (CH), 133.7 (CH), 130.7 (C), 130.1 (CH), 129.5 (CH), 129.4 (C), 127.0 (CH), 126.6 (CH), 125.2 (CH), 123.9 (CH), 123.4 (C), 123.1 (CH), 121.2 (C), 120.7 (C), 117.2 (C), 113.6 (C), 112.5 (CH), 21.4 (CH_3). HRMS (ESI) m/z: 309.1386 calcd for $C_{22}H_{17}N_2^+$ [M – Br]$^+$, found 309.1390.

*7-Methoxy-3-phenylpyrido[2,1-a]pyrrolo[3,4-c]isoquinolin-2-ium bromide (**3o**).* Compound **3o** (119 mg, 72%) was obtained from 1-(4-(2-bromophenyl)-2-phenyl-1*H*-pyrrol-3-yl)-4-methoxypyridin-1-ium bromide (**1o**) (200 mg, 0.41 mmol, 1 equiv), TTMSS (153 mg, 0.616 mmol, 1.5 equiv) and AIBN (134 mg, 0.82 mmol, 2 equiv) according to the general procedure C. Yellow-green solid, mp 213–214 °C (MeCN/EtOAc). ^{1}H NMR (400 MHz, DMSO-d_6): δ 12.86 (s, 1H), 8.95–8.88 (m, 2H), 8.63 (d, J = 2.9 Hz, 1H), 8.37 (d, J = 7.9 Hz, 1H), 8.22 (d, J = 3.2 Hz, 1H), 7.96–7.90 (m, 1H), 7.71–7.59 (m, 7H), 4.21 (s, 3H). ^{13}C{1H} NMR (101 MHz, DMSO-d_6): δ 166.9 (C), 145.1 (C), 136.7 (CH), 133.6 (CH), 130.8 (C), 130.2 (CH), 129.4 (CH), 129.3 (CH), 129.3 (C), 127.0 (CH), 126.7 (CH), 122.9 (CH), 120.6 (C), 120.3 (C), 117.0 (C), 112.9 (CH), 112.5 (CH), 106.8 (CH), 58.0 (CH_3). HRMS (ESI) m/z: 325.1335 calcd for $C_{22}H_{17}N_2O^+$ [M – Br]$^+$, found 325.1348.

*7-(Dimethylamino)-3-phenylpyrido[2,1-a]pyrrolo[3,4-c]isoquinolin-2-ium bromide (**3p**).* Compound **3p** (215 mg, 85%) was obtained from 1-(4-(2-bromophenyl)-2-phenyl-1*H*-pyrrol-3-yl)-4-(dimethylamino)pyridin-1-ium bromide (**1p**) (300 mg, 0.40 mmol, 1 equiv), TTMSS (223 mg, 0.60 mmol, 1.5 equiv) and AIBN (198 mg, 0.80 mmol, 2 equiv) according to the general procedure C. Yellow solid, mp 355–357 °C (dec., MeCN/EtOAc). ^{1}H NMR (400 MHz, DMSO-d_6): δ 12.47 (d, J = 3.2 Hz, 1H), 8.68 (d, J = 8.4 Hz, 1H), 8.31 (d, J = 7.9 Hz, 1H), 8.19–8.14 (m, 1H), 7.99 (d, J = 3.2 Hz, 1H), 7.78–7.70 (m, 2H), 7.64–7.56 (m, 5H), 7.53–7.47 (m, 1H), 7.14 (dd, J = 7.9, 3.0 Hz, 1H), 3.36–3.18 (m, 6H). ^{13}C{1H} NMR (101 MHz, DMSO-d_6): δ 153.6 (C), 142.1 (C), 134.0 (CH), 132.5 (CH), 131.2 (C), 130.1 (CH), 129.2 (CH), 120.0 (CH), 126.4 (CH),

126.3 (CH), 122.8 (CH), 120.9 (C), 118.8 (C), 116.8 (C), 112.4 (CH), 111.9 (C), 107.7 (CH), 101.1 (CH), 39.9 (CH_3). HRMS (ESI) m/z: 338.1652 calcd for $C_{23}H_{20}N_3^+$ [M – Br]$^+$, found 338.1656.

*3,7-Diphenylpyrido[2,1-a]pyrrolo[3,4-c]isoquinolin-2-ium bromide (**3q**)*. Compound **3q** (68 mg, 53%) was obtained from 1-(4-(2-bromophenyl)-2-phenyl-1*H*-pyrrol-3-yl)-4-phenylpyridin-1-ium bromide (**1q**) (150 mg, 0.282 mmol, 1 equiv), TTMSS (105 mg, 0.56 mmol, 1.5 equiv) and AIBN (92 mg, 0.75 mmol, 2 equiv) according to the general procedure C. Orange solid, mp 220 °C (dec., MeCN/EtOAc). ^{1}H NMR (400 MHz, DMSO-d_6): δ 13.11 (s, 1H), 9.51 (d, J = 2.2 Hz, 1H), 9.22 (d, J = 8.5 Hz, 1H), 9.11 (d, J = 7.2 Hz, 1H), 8.49–8.39 (m, 2H), 8.32 (d, J = 3.2 Hz, 1H), 8.28–8.23 (m, 2H), 8.02–7.96 (m, 1H), 7.81–7.61 (m, 9H). ^{13}C{1H} NMR (101 MHz, DMSO-d_6): δ 112.5 (CH), 149.5 (C), 143.0 (C), 135.0 (CH), 134.0 (C), 133.8 (CH), 131.6 (CH), 130.8 (C), 130.3 (CH), 129.6 (CH), 129.5 (CH), 128.1 (CH), 127.4 (CH), 127.0 (CH), 123.3 (C), 123.0 (CH), 121.4 (C), 121.19 (CH), 121.14 (C), 120.0 (CH), 117.3 (C), 113.8 (C). HRMS (ESI) m/z: 371.1543 calcd for $C_{27}H_{19}N_2^+$ [M – Br]$^+$, found 371.1547.

*7-(4-Methoxyphenyl)-3-phenylpyrido[2,1-a]pyrrolo[3,4-c]isoquinolin-2-ium bromide (**3r**).* Compound **3r** (73 mg, 71%) was obtained from 1-(4-(2-bromophenyl)-2-phenyl-1*H*-pyrrol-3-yl)-4-(4-methoxyphenyl)pyridin-1-ium bromide (**1r**) (120 mg, 0.214 mmol, 1 equiv), TTMSS (80 mg, 0.32 mmol, 1.5 equiv) and AIBN (70 mg, 0.43 mmol, 2 equiv) according to the general procedure C. Dark orange solid, mp 230–233 °C (MeCN/EtOAc). ^{1}H NMR (500 MHz, DMSO-d_6): δ 13.04 (d, J = 3.3 Hz, 1H), 9.33 (d, J = 2.3 Hz, 1H), 9.15 (d, J = 8.5 Hz, 1H), 8.96 (d, J = 7.2 Hz, 1H), 8.38 (d, J = 7.6 Hz, 1H), 8.34 (d, J = 7.4 Hz, 1H), 8.28–8.22 (m, 3H), 7.95–7.90 (m, 1H), 7.75–7.63 (m, 6H), 7.18–7.12 (m, 2H), 3.88 (s, 3H). ^{13}C{1H} NMR (126 MHz, DMSO-d_6): δ 162.3 (C), 149.0 (C), 142.8 (C), 134.7 (CH), 133.7 (CH), 130.8 (C), 130.2 (CH), 129.9 (CH), 129.50 (CH), 129.46 (CH), 127.3 (CH), 126.9 (CH), 125.8 (C), 123.0 (CH), 121.14 (C), 121.12 (C), 120.2 (CH), 118.4 (CH), 117.2 (C), 114.9 (CH), 113.6 (C), 112.5 (CH), 55.6 (CH_3). HRMS (ESI) m/z: 401.1648 calcd for $C_{28}H_{21}N_2O^+$ [M – Br]$^+$, found 401.1663.

*6,8-Dimethyl-3-phenylpyrido[2,1-a]pyrrolo[3,4-c]isoquinolin-2-ium bromide (**3s**).* Compound **3s** (93 mg, 56%) was obtained from 1-(4-(2-bromophenyl)-2-phenyl-1*H*-pyrrol-3-yl)-3,5-dimethylpyridin-1-ium bromide (**1s**) (200 mg, 0.41 mmol, 1 equiv), TTMSS (154 mg, 0.62 mmol, 1.5 equiv) and AIBN (135 mg, 0.83 mmol, 2 equiv) according to the general procedure C. Orange solid, mp > 400 °C (MeCN/EtOAc). ^{1}H NMR (400 MHz, DMSO-d_6): δ 13.08 (s, 1H), 8.92–8.87 (m, 1H), 8.68 (d, J = 8.5 Hz, 1H), 8.43 (dd, J = 7.9, 1.4 Hz, 1H), 8.38 (d, J = 1.8 Hz, 1H), 8.26 (d, J = 3.2 Hz, 1H), 7.94–7.89 (m, 1H), 7.69–7.60 (m, 6H), 3.08 (s, 3H), 2.24 (s, 3H).

$^{13}C\{1H\}$ NMR (101 MHz, DMSO-d_6): δ 145.1 (CH), 136.5 (C), 133.2 (CH), 132.7 (CH), 132.6 (C), 130.8 (C), 130.1 (C), 130.0 (CH), 129.5 (CH), 129.3 (CH), 128.5 (CH), 125.8 (CH), 122.9 (CH), 121.6 (C), 121.5 (C), 117.9 (C), 114.0 (C), 112.1 (CH), 25.6 (CH_3), 17.3 (CH_3). HRMS (ESI) m/z: 323.1543 calcd for $C_{23}H_{19}N_2^+$ $[M – Br]^+$, found 323.1538.

1-(2-(2,4-Dimethoxyphenyl)-2-oxoethyl)pyridin-1-ium bromide ***(5i)***. Compound **5i** (391 mg, 75%) was obtained from pyridine (122 mg, 1.54 mmol, 1 equiv) and 2-bromo-1-(2,4-dimethoxyphenyl)ethan-1-one (400 mg, 1.54 mmol, 1 equiv) in acetone (10 mL). Pale yellow solid, mp 287–288 °C (acetone). ^1H NMR (400 MHz, DMSO-d_6): δ 8.99 (d, J = 5.6 Hz, 2H), 8.69 (t, J = 7.5 Hz, 1H), 8.22 (t, J = 6.6 Hz, 2H), 7.86 (d, J = 8.7 Hz, 1H), 6.80 (d, J = 2.1 Hz, 1H), 6.73 (dd, J = 8.9, 2.1 Hz, 1H), 6.16 (s, 2H), 4.04 (s, 3H), 3.90 (s, 3H). $^{13}C\{1H\}$ NMR (101 MHz, DMSO-d_6): δ 187.9 (C), 165.8 (C), 162.0 (C), 146.0 (CH), 145.8 (CH), 132.1 (CH), 127.2 (CH), 115.9 (C), 107.0 (CH), 98.2 (CH), 69.4 (CH_2), 56.2 (CH_3), 55.8 (CH_3). HRMS (ESI) m/z: 258.1125 calcd for $C_{15}H_{16}NO_3^+$ $[M – Br]^+$, found 258.1129.

4-(4-Methoxyphenyl)-1-(2-oxo-2-phenylethyl)pyridin-1-ium bromide ***(5r)***. Compound **5r** (541 mg, 87%) was obtained from 4-(4-methoxyphenyl)pyridine (300 mg, 1.62 mmol, 1 equiv) and 2-bromo-1-phenylethan-1-one (323 mg, 1.62 mmol, 1 equiv) in acetone (10 mL). White solid, mp 256–257 °C (acetone). ^1H NMR (400 MHz, DMSO-d_6): δ 8.97 (d, J = 6.8 Hz, 2H), 8.60 (d, J = 6.9 Hz, 2H), 8.17 (d, J = 8.8 Hz, 2H), 8.09 (d, J = 7.5 Hz, 2H), 7.80 (t, J = 7.4 Hz, 1H), 7.67 (t, J = 7.7 Hz, 2H), 7.21 (d, J = 8.8 Hz, 2H), 6.50 (s, 2H), 3.90 (s, 3H). $^{13}C\{1H\}$ NMR (101 MHz, DMSO-d_6): δ 191.0 (C), 162.9 (C), 154.6 (C), 145.8 (CH), 134.6 (CH), 133.6 (C), 130.2 (CH), 129.1 (CH), 128.2 (CH), 125.3 (C), 122.8 (CH), 115.2 (CH), 65.1 (CH_2), 55.7 (CH_3). HRMS (ESI) m/z: 304.1332 calcd for $C_{20}H_{18}NO_2^+$ $[M – Br]^+$, found 304.1328.

4-(Methoxycarbonyl)-1-(2-oxo-2-phenylethyl)pyridin-1-ium bromide ***(5u)***. Compound (842 mg, 80%) **5u** was obtained from methyl isonicotinate (400 mg, 2.92 mmol, 1 equiv) and 2-bromo-1-phenylethan-1-one (581 mg, 2.92 mmol, 1 equiv) in acetone (5 mL). White solid, mp 178 °C (dec., acetone). ^1H NMR (400 MHz, DMSO-d_6): δ 9.26 (d, J = 6.5 Hz, 2H), 8.66 (d, J = 6.5 Hz, 2H), 8.08 (d, J = 7.5 Hz, 2H), 7.80 (t, J = 7.4 Hz, 1H), 7.67 (t, J = 7.7 Hz, 2H), 6.69 (s, 2H), 4.01 (s, 3H). $^{13}C\{1H\}$ NMR (101 MHz, DMSO-d_6): δ 190.3 (C), 162.5 (C), 147.8 (CH), 144.4 (C), 134.7 (CH), 133.5 (C), 129.1 (CH), 128.3 (CH), 127.0 (CH), 66.7 (CH_2), 53.8 (CH_3). HRMS (ESI) m/z: 256.0968 calcd for $C_{15}H_{14}NO_3^+$ $[M – Br]^+$, found 256.0973.

4-Benzoyl-1-(2-oxo-2-phenylethyl)pyridin-1-ium bromide ***(5w)***. Compound **5w** (291 mg, 84%) was obtained from phenyl(pyridin-4-yl)methanone (166 mg, 0.905 mmol, 1 equiv) and 2-bromo-

1-phenylethan-1-one (180 mg, 0.905 mmol, 1 equiv) in acetone (8 mL). White solid, mp 191–192 °C (acetone). ^{1}H NMR (400 MHz, DMSO-d_6): δ 9.25 (d, J = 6.7 Hz, 1H), 8.51 (d, J = 6.7 Hz, 1H), 8.15–8.08 (m, 1H), 7.93–7.86 (m, 1H), 7.85–7.80 (m, 1H), 7.71–7.65 (m, 2H), 6.65 (s, 1H). ^{13}C NMR{1H} (101 MHz, DMSO-d_6): δ 192.0 (C), 190.4 (C), 152.1 (C), 147.3 (CH), 134.82 (CH), 134.78 (CH), 134.1 (C), 133.5 (C), 130.3 (CH), 129.1 (CH), 128.3 (CH), 126.8 (CH), 66.4 (CH_2). HRMS (ESI) m/z: 302.1176 calcd for $C_{20}H_{16}NO_2^+$ [M – Br]$^+$, found 302.1189.

General procedure D for the synthesis of pyrido[2,1-*a*]pyrroloisoquinolines 6 and 17. The corresponding salt was dissolved in water (about 1 mg in 1 ml) and then aq 10% KOH (about 0.3 ml per 1 mg of salt) and DCM, in a volume sufficient to completely dissolve the product, were added. After stirring for 1 min the organic layer was separated. The water layer was twice extracted with DCM and the combined DCM solution was dried under Na_2SO_4, filtered and evaporated to dryness to give the product.

3-Phenylpyrido[2,1-a]pyrrolo[3,4-c]isoquinoline (**6a**). Compound **6a** (78 mg, 99%) was obtained from bromide (**3a**) (100 mg, 0.27 mmol) according to the general procedure D. Dark purple solid, mp 195–197 °C (DCM). ^{1}H NMR (400 MHz, DMSO-d_6): δ 9.26 (dd, J = 6.8, 1.2 Hz, 1H), 9.17 (dd, J = 9.0, 1.5 Hz, 1H), 8.73 (d, J = 8.5 Hz, 1H), 8.25 (dd, J = 8.2, 1.2 Hz, 1H), 8.11 (ddd, J = 8.7, 7.2, 1.4 Hz, 1H), 7.94 (s, 1H), 7.82–7.73 (m, 2H), 7.63–7.58 (m, 2H), 7.49–7.42 (m, 3H), 7.38–7.30 (m, 1H). ^{13}C{1H} NMR (101 MHz, DMSO-d_6): δ 139.2 (C), 137.9 (C), 134.8 (CH), 133.4 (CH), 132.9 (CH), 131.2 (C), 129.6 (CH), 128.5 (CH), 127.8 (C), 126.5 (CH), 126.1 (CH), 124.3 (CH), 123.7 (CH), 122.6 (CH), 122.2 (CH), 119.9 (CH), 118.8 (C), 117.6 (C), 114.9 (C). HRMS (ESI) m/z: 295.1230 calcd for $C_{21}H_{15}N_2^+$ [M + H]$^+$, found 295.1235.

3-(4-Chlorophenyl)pyrido[2,1-a]pyrrolo[3,4-c]isoquinoline (**6c**). Compound **6c** (22 mg, 99%) was obtained from bromide (**3c**) (27 mg, 0.066 mmol) according to the general procedure D. Dark red solid, mp 207–209 °C (dec., DCM). ^{1}H NMR (400 MHz, DMSO-d_6): δ 9.25–9.14 (m, 2H), 8.75 (d, J = 8.5 Hz, 1H), 8.26 (d, J = 8.1 Hz, 1H), 8.19–8.14 (m, 1H), 7.97 (s, 1H), 7.85–7.77 (m, 2H), 7.67–7.60 (m, 2H), 7.53–7.44 (m, 3H). ^{13}C{1H} NMR (101 MHz, DMSO-d_6): δ 138.9 (C), 137.7 (C), 134.3 (CH), 133.6 (CH), 132.8 (CH), 131.3 (C), 131.0 (CH), 130.5 (C), 128.3 (CH), 127.4 (C), 126.0 (CH), 124.0 (CH), 123.6 (CH), 122.6 (CH), 122.1 (CH), 121.4 (CH), 118.6 (C), 117.9 (C), 115.3 (C). HRMS (ESI) m/z: 329.0840 calcd for $C_{21}H_{14}ClN_2^+$ [M + H]$^+$, found 329.0850.

3-(3-Methyl)pyrido[2,1-a]pyrrolo[3,4-c]isoquinoline (**6e**). Compound **6e** (31 mg, 98%) was obtained from bromide (**3e**) (40 mg, 0.10 mmol) according to the general procedure D. Dark

purple solid, mp 89–90 °C (DCM). ^{1}H NMR (400 MHz, DMSO-d_6): δ 9.28 (d, J = 6.8 Hz, 1H), 9.18 (d, J = 8.8 Hz, 1H), 8.74 (d, J = 8.4 Hz, 1H), 8.25 (d, J = 8.1 Hz, 1H), 8.16–8.09 (m, 1H), 7.94 (s, 1H), 7.83–7.75 (m, 2H), 7.51–7.43 (m, 3H), 7.29 (d, J = 7.5 Hz, 2H), 2.40 (s, 3H). ^{13}C{1H} NMR (101 MHz, DMSO-d_6): δ 138.9 (C), 135.4 (C), 134.3 (CH), 133.1 (CH), 132.8 (CH), 131.3 (C), 129.5 (CH), 129.1 (CH), 128.3 (C), 126.0 (CH), 124.0 (CH), 123.6 (CH), 122.5 (CH), 122.1 (CH), 120.2 (CH), 118.6 (C), 117.5 (C), 114.8 (C), 20.9 (CH_3). HRMS (ESI) m/z: 309.1386 calcd for $C_{22}H_{17}N_2^+$ [M + H]$^+$, found 309.1377.

3-(4-Methoxyphenyl)pyrido[2,1-a]pyrrolo[3,4-c]isoquinoline (**6f**). Compound **6f** (41 mg, 98%) was obtained from bromide (**3f**) (69 mg, 0.17 mmol) according to the general procedure D. Dark red solid, mp 150–151 °C (dec., DCM). ^{1}H NMR (400 MHz, DMSO-d_6): δ 9.23 (dd, J = 18.4, 7.7 Hz, 2H), 8.76 (d, J = 8.4 Hz, 1H), 8.28 (d, J = 8.0 Hz, 1H), 8.25–8.17 (m, 1H), 7.99 (s, 1H), 7.88–7.78 (m, 2H), 7.53 (dd, J = 8.5, 6.4 Hz, 3H), 7.12–7.06 (m, 2H), 3.85 (s, 3H). ^{13}C{1H} NMR (101 MHz, DMSO-d_6): δ 160.0 (C), 142.5 (C), 139.7 (CH), 134.7 (CH), 133.8 (CH), 131.6 (CH), 129.5 (C), 127.0 (CH), 126.6 (CH), 124.3 (CH), 124.1 (CH), 123.0 (CH), 122.5 (C), 121.5 (C), 120.7 (C), 117.2 (C), 114.9 (CH), 113.6 (C), 111.9 (CH), 55.4 (CH_3). HRMS (ESI) m/z: 325.1335 calcd for $C_{22}H_{17}N_2O^+$ [M + H]$^+$, found 325.1349.

3-(2-Methoxyphenyl)pyrido[2,1-a]pyrrolo[3,4-c]isoquinoline (**6g**). Compound **6g** (40 mg, 98%) was obtained from bromide (**3g**) (51 mg, 0.125 mmol) according to the general procedure D. Dark red solid, mp 176–177 °C (dec., DCM). ^{1}H NMR (400 MHz, DMSO-d_6): δ 9.23 (d, J = 8.7 Hz, 1H), 8.80 (d, J = 8.5 Hz, 1H), 8.74 (dd, J = 6.8, 1.3 Hz, 1H), 8.34–8.25 (m, 2H), 8.10 (s, 1H), 7.90–7.83 (m, 2H), 7.66 (dd, J = 7.6, 1.9 Hz, 1H), 7.59–7.48 (m, 2H), 7.23–7.14 (m, 2H), 3.58 (s, 3H). ^{13}C{1H} NMR (101 MHz, DMSO-d_6): δ 155.9 (C), 138.9 (C), 134.4 (CH),134.3 (CH), 132.8 (CH), 132.0 (CH), 131.2 (C), 128.5 (CH), 126.1 (CH), 124.2 (CH), 123.8 (C), 122.8 (CH), 122.2 (CH), 121.8 (CH), 120.8 (CH), 119.8 (CH), 119.2 (C), 118.7 (C), 114.3 (C), 111.3 (C), 54.8 (CH_3). HRMS (ESI) m/z: 325.1335 calcd for $C_{22}H_{17}N_2O^+$ [M + H]$^+$, found 325.1331.

3-(3-Methoxyphenyl)pyrido[2,1-a]pyrrolo[3,4-c]isoquinoline (**6h**). Compound **6h** (57 mg, 99%) was obtained from bromide (**3h**) (72 mg, 0.18 mmol) according to the general procedure D. Dark brown solid, mp 153–155 °C (DCM). ^{1}H NMR (400 MHz, DMSO-d_6): δ 9.30 (d, J = 6.7 Hz, 1H), 9.19 (d, J = 8.7 Hz, 1H), 8.76 (d, J = 8.5 Hz, 1H), 8.27 (d, J = 8.1 Hz, 1H), 8.19–8.12 (m, 1H), 7.96 (s, 1H), 7.84–7.77 (m, 2H), 7.48 (ddd, J = 8.4, 6.9, 1.3 Hz, 1H), 7.42–7.36 (m, 1H), 7.22–7.14 (m, 2H), 6.92 (dd, J = 8.2, 2.6 Hz, 1H), 3.80 (s, 3H). ^{13}C{1H} NMR (126 MHz, DMSO-d_6): δ 159.3 (C), 139.8 (C), 139.0 (C), 134.4 (CH), 133.6 (CH), 132.8 (CH), 131.3 (C), 129.4 (CH), 128.3 (C), 126.0 (CH), 124.1 (CH), 123.6 (CH), 122.4 (CH), 122.2 (CH), 121.8

(CH), 120.4 (CH), 118.7 (C), 117.7 (C), 115.0 (C), 114.7 (CH), 112.3 (CH), 55.1 (CH_3). HRMS (ESI) m/z: 325.1335 calcd for $C_{22}H_{17}N_2O^+$ $[M + H]^+$, found 325.1339.

3-(2,4-Dimethoxyphenyl)pyrido[2,1-a]pyrrolo[3,4-c]isoquinoline (**6i**). Compound **6i** (46 mg, 99%) was obtained from bromide (**3i**) (59 mg, 0.13 mmol) according to the general procedure D. Dark brown solid, mp 202–205 °C (DCM). ^1H NMR (400 MHz, DMSO-d_6): δ 9.15 (d, J = 8.7 Hz, 1H), 8.80–8.68 (m, 2H), 8.24 (d, J = 8.0 Hz, 1H), 8.21–8.13 (m, 1H), 7.97 (s, 1H), 7.84–7.76 (m, 2H), 7.58–7.51 (m, 1H), 7.51–7.44 (m, 1H), 6.76–6.70 (m, 2H), 3.87 (s, 3H), 3.56 (s, 3H). $^{13}C\{1H\}$ NMR (101 MHz, DMSO-d_6): δ 160.4 (C), 157.1 (C), 139.3 (C), 134.8 (CH), 134.2 (CH), 132.9 (CH), 132.6 (CH), 131.1 (C), 126.1 (CH), 124.5 (CH), 123.0, 122.9 (CH), 122.23 (CH), 122.17 (CH), 118.9 (C), 118.7 (C), 118.3 (CH), 118.1 (C), 114.0 (C), 105.6 (CH), 98.7 (CH), 55.3 (CH_3), 55.0 (CH_3). HRMS (ESI) m/z: 355.1441 calcd for $C_{23}H_{19}N_2O_2^+$ $[M + H]^+$, found 355.1448.

4-(Pyrido[2,1-a]pyrrolo[3,4-c]isoquinolin-3-yl)benzonitrile (**6l**). Compound **6l** (37 mg, 99%) was obtained from bromide (**3l**) (46 mg, 0.115 mmol) according to the general procedure D. Dark red solid, mp 225–227 °C (DCM). ^1H NMR (400 MHz, DMSO-d_6): δ 9.23 (d, J = 8.7 Hz, 1H), 9.17 (d, J = 6.8 Hz, 1H), 8.79 (d, J = 8.5 Hz, 1H), 8.29 (d, J = 8.0 Hz, 1H), 8.21 (ddd, J = 8.7, 7.2, 1.4 Hz, 1H), 8.03 (s, 1H), 7.87–7.79 (m, 6H), 7.51 (ddd, J = 8.3, 6.9, 1.3 Hz, 1H). $^{13}C\{1H\}$ NMR (101 MHz, DMSO-d_6): δ 143.4 (C), 139.2 (C), 134.8 (CH), 134.4 (CH), 132.9 (CH), 132.2 (CH), 131.1 (C), 129.2 (CH), 127.2 (C), 126.0 (CH), 124.3 (CH), 123.7 (CH), 123.1 (CH), 122.6 (CH), 122.2 (CH), 119.5 (C), 119.0 (C), 118.9 (C), 116.4 (C), 107.2 (C). HRMS (ESI) m/z: 320.1182 calcd for $C_{22}H_{14}N_3^+$$[M + H]^+$, found 320.1186.

3-(Pyridin-2-yl)pyrido[2,1-a]pyrrolo[3,4-c]isoquinoline (**6m**). Compound **6m** (59 mg, 99%) was obtained from iodide (**3m**) (85 mg, 0.20 mmol) according to the general procedure D. Dark purple solid, mp 92–93 °C (DCM). ^1H NMR (400 MHz, DMSO-d_6): δ 11.57 (d, J = 6.7 Hz, 1H), 9.18 (dd, J = 8.8, 1.5 Hz, 1H), 8.73 (d, J = 8.4 Hz, 1H), 8.57 (dd, J = 5.0, 1.8 Hz, 1H), 8.32–8.18 (m, 3H), 8.06 (s, 1H), 7.95–7.90 (m, 1H), 7.83–7.76 (m, 2H), 7.48 (ddd, J = 8.3, 6.9, 1.3 Hz, 1H), 7.19 (ddd, J = 7.3, 4.9, 1.2 Hz, 1H). $^{13}C\{1H\}$ NMR (101 MHz, DMSO-d_6): δ 156.8 (C), 147.2 (CH), 139.5 (C), 138.6 (CH), 136.4 (CH), 135.0 (CH), 132.8 (CH), 131.0 (C), 127.9 (C), 125.9 (CH), 124.5 (CH), 123.5 (CH), 123.0 (CH), 122.7 (C), 122.21 (CH), 122.1 (CH), 120.9 (C), 119.8 (CH), 118.9 (C), 116.7 (C). HRMS (ESI) m/z: 296.1182 calcd for $C_{20}H_{14}N_3^+$ $[M + H]^+$, found 296.1193.

7-Methyl-3-phenylpyrido[2,1-a]pyrrolo[3,4-c]isoquinoline (**6n**). Compound **6n** (54.5 mg, 99%) was obtained from bromide (**3n**) (69 mg, 0.18 mmol) according to the general procedure D. Dark brown solid, mp > 400 °C (DCM). ^{1}H NMR (400 MHz, DMSO-d_6): δ 9.11–9.05 (m, 2H), 8.76 (d, J = 8.4 Hz, 1H), 8.25 (dd, J = 8.2, 1.2 Hz, 1H), 7.95 (s, 1H), 7.83–7.77 (m, 1H), 7.68 (dd, J = 7.0, 1.9 Hz, 1H), 7.62–7.58 (m, 2H), 7.51–7.45 (m, 3H), 7.39–7.34 (m, 1H), 2.63 (s, 3H). ^{13}C{1H} NMR (126 MHz, DMSO-d_6): δ 147.5 (C), 139.2 (C), 137.5 (C), 132.7 (CH), 131.2 (C), 129.5 (CH), 128.5 (CH), 127.0 (C), 126.6 (CH), 126.1 (CH), 124.2 (CH), 123.9 (CH), 123.1 (CH), 122.2 (CH), 119.4 (CH), 118.6 (C), 117.3 (C), 114.5 (C), 21.0 (CH_3). HRMS (ESI) m/z: 309.1386 calcd for $C_{22}H_{17}N_2^+$ [M + H]$^+$, found 309.1400.

7-Methoxy-3-phenylpyrido[2,1-a]pyrrolo[3,4-c]isoquinoline (**6o**). Compound **6o** (45 mg, 99%) was obtained from bromide (**3o**) (57 mg, 0.14 mmol) according to the general procedure D. Dark orange solid, mp 149–151 °C (dec., DCM). ^{1}H NMR (400 MHz, DMSO-d_6): δ 9.07 (d, J = 7.6 Hz, 1H), 8.77 (d, J = 8.4 Hz, 1H), 8.46 (d, J = 3.0 Hz, 1H), 8.18 (dd, J = 8.1, 1.2 Hz, 1H), 7.86 (s, 1H), 7.76 (ddd, J = 8.0, 6.9, 1.0 Hz, 1H), 7.59–7.52 (m, 3H), 7.48–7.39 (m, 3H), 7.36–7.31 (m, 1H), 4.15 (s, 3H). ^{13}C{1H} NMR (101 MHz, DMSO-d_6): δ 163.3 (C), 142.0 (C), 138.5 (C), 135.2 (CH), 132.8 (CH), 131.6 (C), 129.5 (CH), 128.4 (CH), 127.0 (C), 126.4 (CH), 126.0 (CH), 123.5 (CH), 122.0 (CH), 120.5 (CH), 118.2 (C), 117.0 (C), 113.8 (C), 112.2 (CH), 105.3 (CH), 57.2 (CH_3). HRMS (ESI) m/z: 325.1335 calcd for $C_{22}H_{17}N_2O^+$ [M + H]$^+$, found 325.1333.

N,N-Dimethyl-3-phenylpyrido[2,1-a]pyrrolo[3,4-c]isoquinolin-7-amine (**6p**). Compound **6p** (127 mg, 99%) was obtained from bromide (**3p**) (159 mg, 0.38 mmol) according to the general procedure D. Orange solid, mp 199–200 °C (DCM). ^{1}H NMR (400 MHz, DMSO-d_6): δ 8.69–8.61 (m, 2H), 8.09 (d, J = 7.9 Hz, 1H), 7.82–7.76 (m, 2H), 7.72–7.66 (m, 1H), 7.56 (d, J = 7.5 Hz, 2H), 7.50–7.45 (m, 2H), 7.41–7.34 (m, 2H), 7.22 (dd, J = 7.8, 3.0 Hz, 1H), 3.27 (s, 6H). ^{13}C{1H} NMR (101 MHz, DMSO-d_6): δ 153.0 (C), 141.7 (C), 134.0 (CH), 132.4 (CH), 130.0 (C), 129.9 (C), 128.9 (CH), 128.1 (CH), 127.8 (CH), 127.4 (CH), 126.2 (CH), 125.3 (CH), 122.5 (CH), 121.2 (C), 120.2 (C), 116.9 (C), 112.1 (C), 107.6 (CH), 100.9 (CH), 39.5 (CH_3). HRMS (ESI) m/z: 338.1652 calcd for $C_{23}H_{20}N_3^+$ [M + H]$^+$, found 338.1656.

3,7-Diphenylpyrido[2,1-a]pyrrolo[3,4-c]isoquinoline (**6q**). Compound **6q** (49 mg, 99%) was obtained from bromide (**3q**) (60 mg, 0.135 mmol) according to the general procedure D. Dark brown solid, mp 195–198 °C (DCM). ^{1}H NMR (400 MHz, DMSO-d_6): δ 9.32 (d, J = 2.2 Hz, 1H), 9.21 (d, J = 7.2 Hz, 1H), 9.02 (d, J = 8.5 Hz, 1H), 8.27–8.14 (m, 4H), 7.97 (s, 1H), 7.84–7.78 (m, 1H), 7.66–7.46 (m, 8H), 7.43–7.38 (m, 1H). ^{13}C{1H} NMR (101 MHz, DMSO-d_6): δ 144.7 (C), 139.5 (C), 137.8 (C), 134.7 (C), 133.4 (CH), 132.9 (CH), 131.2 (C), 130.6 (CH),

129.6 (CH), 129.3 (CH), 128.6 (CH), 127.7 (C), 127.6 (CH), 126.7 (CH), 126.6 (CH), 124.3 (CH), 122.1 (CH), 120.0 (CH), 119.8 (CH), 119.5 (CH), 119.1 (C), 117.4 (C), 114.8 (C). HRMS (ESI) m/z: 371.1543 calcd for $C_{27}H_{19}N_2^+$ [M + H]$^+$, found 371.1531.

7-(4-Methoxyphenyl)-3-phenylpyrido[2,1-a]pyrrolo[3,4-c]isoquinoline (**6r**). Compound **6r** (31.5 mg, 99%) was obtained from bromide (**3r**) (38 mg, 0.079 mmol) according to the general procedure D. Dark brown solid, mp 149–151 °C (DCM). ^{1}H NMR (400 MHz, DMSO-d_6): δ 9.25 (d, J = 2.3 Hz, 1H), 9.11 (d, J = 7.2 Hz, 1H), 9.03 (d, J = 8.5 Hz, 1H), 8.27–8.16 (m, 4H), 7.99 (s, 1H), 7.84–7.78 (m, 1H), 7.69–7.62 (m, 2H), 7.56–7.48 (m, 3H), 7.46–7.41 (m, 1H), 7.16–7.11 (m, 2H), 3.87 (s, 3H). ^{13}C{1H} NMR (101 MHz, DMSO-d_6): δ 161.6 (C), 145.2 (C), 140.1 (C), 136.6 (C), 133.6 (CH), 132.9 (CH), 130.9 (C), 129.7 (CH), 129.3 (CH), 128.7 (CH), 127.0 (CH), 126.8 (CH), 126.6 (C), 126.3 (C), 124.6 (CH), 122.2 (CH), 119.5 (C), 119.4 (CH), 118.5 (CH), 118.1 (CH), 117.4 (C), 114.8 (CH), 114.4 (C), 55.5 (CH_3). HRMS (ESI) m/z: 401.1648 calcd for $C_{28}H_{21}N_2O^+$ [M + H]$^+$, found 401.1644.

6,8-Dimethyl-3-phenylpyrido[2,1-a]pyrrolo[3,4-c]isoquinoline (**6s**). Compound **6s** (32 mg, 99%) was obtained from bromide (**3s**) (40 mg, 0.1 mmol) according to the general procedure D. Brown solid, mp 208 °C (DCM). ^{1}H NMR (400 MHz, DMSO-d_6): δ 9.04 (s, 1H), 8.61 (d, J = 8.6 Hz, 1H), 8.27 (d, J = 8.0 Hz, 1H), 8.05 (s, 1H), 7.93 (s, 1H), 7.81–7.75 (m, 1H), 7.64–7.58 (m, 2H), 7.51–7.36 (m, 4H), 3.06 (s, 3H), 2.23 (s, 3H). ^{13}C{1H} NMR (101 MHz, DMSO-d_6): δ 142.33 (CH), 142.31 (CH), 139.1 (C), 135.9 (C), 132.3 (CH), 132.2 (CH), 131.6 (C), 131.2 (C), 129.7 (CH), 129.6 (CH), 128.7 (CH), 127.6 (CH), 124.0 (CH), 122.4 (CH), 120.3 (C), 118.0 (C), 116.5 (C), 114.7 (C), 25.7 (CH_3), 17.3 (CH_3). HRMS (ESI) m/z: 323.1543 calcd for $C_{23}H_{19}N_2^+$ [M + H]$^+$, found 323.1547.

1-(4-(2-Bromophenyl)-5-(tert-butoxycarbonyl)-2-phenyl-1H-pyrrol-3-yl)pyridin-1-ium bromide (7). Compound **7** (366 mg, 73%) was obtained from 1-(2-oxo-2-phenylethyl)pyridin-1-ium bromide (**5a**) (249 mg, 0.903 mmol, 1 equiv), triethylamine (136 mg, 1.35 mmol, 1.5 equiv) and *tert*-butyl 3-(2-bromophenyl)-*2H*-azirine-2-carboxylate (**4b**) (400 mg, 1.35 mmol, 1.5 equiv) in 10 mL of DCM according to the general procedure A. Colorless solid, mp 260–261 °C (dec., DCM). ^{1}H NMR (400 MHz, DMSO-d_6): δ 13.23 (br. s, 1H), 9.02 (d, J = 5.9 Hz, 2H), 8.72–8.65 (m, 1H), 8.17 (t, J = 7.2 Hz, 2H), 7.63 (dd, J = 8.0, 1.2 Hz, 1H), 7.46 (dd, J = 7.6, 1.8 Hz, 1H), 7.42–7.23 (m, 7H), 1.21 (s, 9H). ^{13}C{1H} NMR (101 MHz, DMSO-d_6): δ 159.1 (C), 147.9 (CH), 147.2 (CH), 132.1 (CH), 132.04 (CH), 131.98 (C), 130.4 (C), 130.2 (CH), 129.2 (CH), 129.0 (CH), 128.6 (CH), 127.8 (CH), 127.4 (C), 127.1 (C), 124.6 (C), 124.17 (C), 124.14 (C), 120.6

(C), 80.8 (C), 27.4 (CH_3). HRMS (ESI) m/z: 475.1016 calcd for $C_{26}H_{24}{}^{79}BrN_2O_2{}^+$ [M – Br]$^+$, found 475.1034.

*1-(Tert-butoxycarbonyl)-3-phenylpyrido[2,1-a]pyrrolo[3,4-c]isoquinolin-2-ium bromide (**8**).* Compound **8** (64 mg, 50%) was obtained from 1-(4-(2-bromophenyl)-5-(*tert*-butoxycarbonyl)-2-phenyl-1*H*-pyrrol-3-yl)pyridin-1-ium bromide (**7**) (150 mg, 0.271 mmol, 1 equiv), TTMSS (101 mg, 0.406 mmol, 1.5 equiv) and AIBN (89 mg, 0.54 mmol, 2 equiv) according to the general procedure C. Pale orange solid, mp > 400 °C (MeCN/EtOAc). ^{1}H NMR (400 MHz, DMSO-d_6): δ 13.61 (s, 1H), 9.70 (d, J = 8.3 Hz, 1H), 9.47 (d, J = 8.8 Hz, 1H), 9.06 (d, J = 8.4 Hz, 1H), 8.97 (d, J = 6.8 Hz, 1H), 8.60–8.52 (m, 1H), 8.16–8.07 (m, 1H), 8.01–7.88 (m, 2H), 7.80–7.68 (m, 5H), 1.67 (s, 9H). ^{13}C{1H} NMR (101 MHz, DMSO-d_6): δ 159.7 (C), 142.8 (C), 140.6 (CH), 134.9 (CH), 133.8 (CH), 130.6 (CH), 130.5 (CH), 129.8 (C), 129.6 (CH), 128.8 (CH), 127.9 (C), 126.9 (CH), 125.5 (C), 124.3 (CH), 124.1 (CH), 122.8 (C), 119.6 (C), 116.4 (C), 116.0 (C), 82.9 (C), 28.0 (CH_3). HRMS (ESI) m/z: 395.1755 calcd for $C_{26}H_{23}N_2O_2{}^+$ [M – Br]$^+$, found 395.1750.

*1-(2-(2-Bromophenyl)-4-(2-iodophenyl)-1H-pyrrol-3-yl)pyridin-1-ium bromide (**9**).* Compound **9** (240 mg, 49%) was obtained from 1-(2-(2-bromophenyl)-2-oxoethyl)pyridin-1-ium bromide (**15a**) (300 mg, 0.84 mmol, 1 equiv), triethylamine (106 mg, 1.05 mmol, 1.5 equiv) and 3-(2-iodophenyl)-*2H*-azirine (250 mg, 1.05 mmol, 1.5 equiv) in DCM (5 mL) according to the general procedure **A**. Isolated using column chromatography. Beige solid, mp 340 °C (DCM/MeOH). ^{1}H NMR (400 MHz, DMSO-d_6): δ 12.45–12.39 (m, 1H), 8.65–8.55 (m, 3H), 8.09 (t, J = 7.1 Hz, 2H), 7.88 (d, J = 7.9 Hz, 1H), 7.71 (dd, J = 8.1, 1.2 Hz, 1H), 7.62 (dd, J = 7.6, 1.8 Hz, 1H), 7.56–7.51 (m, 1H), 7.48–7.37 (m, 3H), 7.34 (d, J = 3.1 Hz, 1H), 7.13–7.09 (m, 1H). ^{13}C{1H} NMR (101 MHz, DMSO-d_6): δ 146.4 (CH), 146.1 (CH), 139.1 (CH), 136.1 (C), 133.3 (CH), 133.1 (CH), 132.1 (C), 131.5 (CH), 130.0 (CH), 129.3 (C), 128.7 (CH), 128.6 (CH), 128.4 (CH), 125.2 (C), 123.8 (C), 123.3 (C), 121.1 (C), 119.2 (CH), 101.4 (C). HRMS (ESI) m/z: 500.9458 calcd for $C_{21}H_{16}{}^{79}BrIN_2{}^+$ [M – Br]$^+$, found 500.9454.

*3-(2-Bromophenyl)pyrido[2,1-a]pyrrolo[3,4-c]isoquinolin-2-ium bromide (**10**).* Compound **10** (36 mg, 58%) was obtained from 1-(2-(2-bromophenyl)-4-(2-iodophenyl)-1*H*-pyrrol-3-yl)pyridin-1-ium bromide (**9**) (80 mg, 0.14 mmol, 1 equiv), TTMSS (51 mg, 0.21 mmol, 1.5 equiv) and AIBN (45 mg, 0.28 mmol, 2 equiv) according to the general procedure C. Orange solid, mp > 400 °C (MeCN/EtOAc).^{1}H NMR (400 MHz, DMSO-d_6): δ 13.14 (s, 1H), 9.39 (d, J = 8.7 Hz, 1H), 9.13 (d, J = 6.6 Hz, 1H), 8.92 (d, J = 8.4 Hz, 1H), 8.56–8.43 (m, 2H), 8.34 (d, J = 3.2 Hz, 1H), 8.02–7.95 (m, 2H), 7.77–7.62 (m, 6H). ^{13}C{1H} NMR (101 MHz, DMSO-d_6): δ 142.8 (C), 140.0 (CH), 135.0 (CH), 133.9 (CH), 130.7 (C), 130.2 (CH), 129.58 (CH), 129.47

(CH), 127.2 (CH), 126.7 (CH), 124.4 (CH), 124.1 (CH), 123.1 (CH), 121.7 (C), 121.0 (C), 117.5 (C), 113.9 (C), 112.5 (CH). HRMS (ESI) m/z: 375.0314 calcd for $C_{21}H_{14}{}^{81}BrN_2{}^+$ [M – Br]$^+$, found 375.0333.

*3-(2-Bromophenyl)pyrido[2,1-a]pyrrolo[3,4-c]isoquinoline (**11**).*Compound **11** (28 mg, 99%) was obtained from 3-(2-bromophenyl)pyrido[2,1-*a*]pyrrolo[3,4-*c*]isoquinolin-4-ium bromide (**10**) (35 mg, 0.077 mmol) according to the general procedure D. Dark purple solid, mp 298–300 °C (DCM). ^{1}H NMR (400 MHz, DMSO-d_6): δ 9.27–9.22 (m, 2H), 8.80 (d, J = 8.5 Hz, 1H), 8.32 (d, J = 8.6 Hz, 1H), 8.27–8.20 (m, 1H), 8.06 (s, 1H), 8.02–7.94 (m, 2H), 7.68–7.62 (m, 2H), 7.57–7.51 (m, 3H), 7.47–7.41 (m, 1H). ^{13}C{1H} NMR (101 MHz, DMSO-d_6): δ 139.7 (C), 136.9 (C), 135.5 (CH), 133.6 (CH), 133.13 (C), 133.06 (CH), 131.0 (C), 129.7 (CH), 129.6 (CH), 128.6 (CH), 127.0 (CH), 126.2 (CH), 124.7 (CH), 123.8 (CH), 122.8 (CH), 122.4 (CH), 119.1 (C), 118.9 (C), 117.6 (C), 114.7 (C). HRMS (ESI) m/z: 373.0335 calcd for $C_{21}H_{14}{}^{79}BrN_2{}^+$ [M + H]$^+$, found 373.0349.

*1-(2,4-Bis(2-bromophenyl)-1H-pyrrol-3-yl)pyridin-1-ium bromide (**12**).* Compound **12** (301 mg, 67%) was obtained from 1-(2-(2-bromophenyl)-2-oxoethyl)-pyridin-1-ium bromide (**15a**) (300 mg, 0.84 mmol, 1 equiv), triethylamine (127 mg, 1.26 mmol, 1.5 equiv) and 3-(2-bromophenyl)-*2H*-azirine (**4a**) (247 mg, 1.26 mmol, 1.5 equiv) in DCM (6 mL) according to the general procedure A. Isolated using column chromatography. Slightly-yellow solid, mp 341–342 °C (DCM/MeOH). ^{1}H NMR (400 MHz, DMSO-d_6): δ 12.47 (s, 1H), 8.70–8.55 (m, 3H), 8.08 (t, J = 7.0 Hz, 2H), 7.69 (d, J = 8.0 Hz, 1H), 7.67–7.58 (m, 2H), 7.58–7.37 (m, 5H), 7.33–7.29 (m, 1H). ^{13}C{1H} NMR (101 MHz, DMSO-d_6): δ 146.4 (CH), 146.1 (CH), 133.4 (CH), 133.1 (CH), 133.0 (CH), 132.8 (CH), 131.9 (C), 131.5 (CH), 130.0 (CH), 129.2 (C), 128.6 (CH), 128.4 (CH), 128.3 (CH), 125.4 (C), 124.1 (C), 123.5 (C), 123.3 (C), 119.5 (CH), 117.8 (C). HRMS (ESI) m/z: 454.9577 calcd for $C_{21}H_{16}{}^{79}Br^{81}BrN_2{}^+$[M – Br]$^+$, found 454.9573.

*Dibenzo[b,g]pyrido[2,1,6-de]pyrrolo[2,3,4-ij]quinolizin-1-ium bromide (**13**).* Compound **13** (52 mg, 74%) was obtained from 1-(2,4-bis(2-bromophenyl)-1*H*-pyrrol-3-yl)pyridin-1-ium bromide (**12**) (100 mg, 0.19 mmol, 1 equiv), TTMSS (140 mg, 0.56 mmol, 4 equiv) and AIBN (184 mg, 1.12 mmol, 6 equiv) according to the general procedure C. Orange solid, mp > 400 °C (MeCN/EtOAc). ^{1}H NMR (500 MHz, DMSO-d_6): δ 13.87 (s, 1H), 9.34 (d, J = 8.5 Hz, 1H), 9.19 (d, J = 8.3 Hz, 1H), 9.03 (d, J = 8.5 Hz, 1H), 8.79 (d, J = 8.3 Hz, 1H), 8.60–8.53 (m, 1H), 8.49 (d, J = 2.7 Hz, 1H), 8.44 (d, J = 7.8 Hz, 1H), 8.24 (d, J = 7.7 Hz, 1H), 8.08–8.03 (m, 1H), 7.90–7.85 (m, 1H), 7.83–7.78 (m, 1H), 7.69–7.63 (m, 1H). ^{13}C{1H} NMR (101 MHz, DMSO-d_6): δ 140.0 (C), 139.3 (C), 135.6 (C), 133.7 (CH), 133.4 (CH), 128.4 (C), 127.8 (CH), 127.6 (CH),

127.2 (CH), 127.0 (CH), 124.6 (C), 123.6 (CH), 123.3 (C), 122.5 (C), 121.5 (C), 120.8 (CH), 120.4 (CH), 119.8 (C), 119.4 (CH), 115.5 (CH), 110.3 (C). HRMS (ESI) m/z: 293.1073 calcd for $C_{21}H_{13}N_2^+$ $[M – Br]^+$, found 293.1080.

*Dibenzo[b,g]pyrido[2,1,6-de]pyrrolo[2,3,4-ij]quinoline **(14)***. Compound **14** (41 mg, 99%) was obtained from dibenzo[*b,g*]pyrido[2,1,6-*de*]pyrrolo[2,3,4-*ij*]quinolizin-1-ium bromide (**13**) (53 mg, 0.14 mmol) according to the general procedure D. Dark brown solid, mp 155–157 °C (dec., DCM). ^{1}H NMR (400 MHz, DMSO-d_6): δ 9.03 (d, J = 8.6 Hz, 1H), 8.86 (d, J = 8.2 Hz, 1H), 8.79 (d, J = 8.5 Hz, 1H), 8.62 (d, J = 8.3 Hz, 1H), 8.44 (dd, J = 8.1, 1.3 Hz, 1H), 8.32 (s, 1H), 8.20–8.10 (m, 2H), 7.86–7.80 (m, 1H), 7.74–7.68 (m, 1H), 7.51 (ddd, J = 8.4, 6.9, 1.4 Hz, 1H), 7.43–7.37 (m, 1H). ^{13}C{1H} NMR (101 MHz, DMSO-d_6): δ 138.3 (C), 137.4 (C), 132.6 (CH), 132.3 (CH), 131.2 (C), 131.0 (CH), 130.4 (C), 127.0 (C), 126.5 (CH), 126.4 (CH), 125.5 (CH), 124.9 (CH), 124.5 (CH), 124.1 (C), 122.5 (CH), 121.5 (C), 120.5 (C), 120.3 (CH), 118.5 (CH), 116.9 (CH), 109.4 (C). HRMS (ESI) m/z: 292.9996 calcd for $C_{21}H_{13}N_2^+$ $[M + H]^+$, found 292.9992.

*1-(2-(2-Bromophenyl)-2-oxoethyl)-4-methylpyridin-1-ium bromide **(15b)***. Compound **15b** (1.18 g, 76%) was obtained from 4-methylpyridine (390 mg, 4.19 mmol, 1 equiv) and 2-bromo-1-(2-bromophenyl)ethan-1-one (1.167 g, 4.19 mmol, 1 equiv) in diethyl ether (18 mL). Bright yellow solid, mp 226–227 °C (Et_2O). ^{1}H NMR (400 MHz, DMSO-d_6): δ 8.93 (d, J = 6.5 Hz, 2H), 8.16–8.12 (m, 3H), 7.86 (d, J = 7.8 Hz, 1H), 7.67 (td, J = 10.7, 4.1 Hz, 1H), 7.61 (td, J = 7.8, 1.6 Hz, 1H), 6.45 (s, 2H), 2.68 (s, 3H). ^{13}C{1H} NMR (101 MHz, DMSO-d_6): δ 192.0 (C), 160.1 (C), 145.0 (CH), 134.76 (CH), 134.61 (C), 134.28 (CH), 131.0 (CH), 128.19 (CH), 128.04 (CH), 119.8 (C), 66.6 (CH_2), 21.7 (CH_3). HRMS (ESI) m/z: 290.0175 calcd for $C_{14}H_{13}{}^{79}BrNO^+$ $[M – Br]^+$, found 290.0167.

*1-(2-(2-Bromophenyl)-2-oxoethyl)-4-(4-methoxyphenyl)pyridin-1-ium bromide **(15e)***. Compound **15e** (592 mg, 79%) was obtained from 4-(4-methoxyphenyl)pyridine (300 mg, 1.62 mmol, 1 equiv) and 2-bromo-1-(2-bromophenyl)ethan-1-one (451 mg, 1.62 mmol, 1 equiv) in acetone (10 mL). White solid, mp 240–241 °C (acetone). ^{1}H NMR (400 MHz, DMSO-d_6): δ 8.98 (d, J = 7.0 Hz, 2H), 8.62 (d, J = 7.0 Hz, 2H), 8.21–8.13 (m, 3H), 7.87 (dd, J = 7.9, 1.0 Hz, 1H), 7.68 (td, J = 7.5, 1.1 Hz, 1H), 7.62 (td, J = 7.7, 1.7 Hz, 1H), 7.22 (d, J = 8.9 Hz, 2H), 6.41 (s, 2H), 3.90 (s, 3H). ^{13}C{1H} NMR (101 MHz, DMSO-d_6): δ 192.0 (C), 163.0 (C), 154.8 (C), 145.7 (CH), 134.83 (CH), 134.57 (C), 134.32 (CH), 131.1 (CH), 130.2 (CH), 128.1 (CH), 125.3 (C), 122.9 (CH), 119.9 (C), 115.3 (CH), 66.2 (CH_2), 55.7 (CH_3). HRMS (ESI) m/z: 382.0437 calcd for $C_{20}H_{17}{}^{79}BrNO_2^+$ $[M – Br]^+$, found 382.0443.

*2-(2-(2-Bromophenyl)-2-oxoethyl)isoquinolin-2-ium bromide **(15g)***. Compound **15g** (453 mg, 90%) was obtained from isoquinoline (159 mg, 1.23 mmol, 1 equiv) and 2-bromo-1-(2-bromophenyl)ethan-1-one (342 mg, 1.23 mmol, 1 equiv) in acetone (5 mL). Beige solid, mp 216–218 °C (acetone). ^{1}H NMR (400 MHz, DMSO-d_6): δ 10.12 (s, 1H), 8.79 (dd, J = 6.9, 1.4 Hz, 1H), 8.72 (d, J = 6.8 Hz, 1H), 8.59 (d, J = 8.3 Hz, 1H), 8.44 (d, J = 8.2 Hz, 1H), 8.34 (ddd, J = 8.3, 6.8, 1.2 Hz, 1H), 8.20 (dd, J = 7.7, 1.8 Hz, 1H), 8.13 (ddd, J = 8.2, 6.9, 1.1 Hz, 1H), 7.88 (dd, J = 7.9, 1.2 Hz, 1H), 7.74–7.59 (m, 2H), 6.62 (s, 2H). ^{13}C NMR (101 MHz, DMSO-d_6): δ 192.0 (C), 151.6 (CH), 137.6 (CH), 137.3 (C), 136.2 (CH), 134.8 (CH), 134.6 (C), 134.4 (CH), 131.4 (CH), 131.1 (CH), 130.6 (CH), 128.1 (CH), 127.4 (CH), 126.8 (C), 125.6 (CH), 119.8 (C), 67.2 (CH_2). HRMS (ESI) m/z: 326.0175 calcd for $C_{17}H_{13}{}^{79}BrNO^+$ [M – Br]$^+$, found 326.0170.

*4-Benzoyl-1-(2-(2-bromophenyl)-2-oxoethyl)pyridin-1-ium bromide **(15j)***. Compound **15j** (613 mg, 61%) was obtained from phenyl(pyridin-4-yl)methanone (400 mg, 2.19 mmol, 1 equiv) and 2-bromo-1-(2-bromophenyl)ethan-1-one (607 mg, 2.19 mmol, 1 equiv) in acetone (10 mL). Pale yellow solid, mp 223 °C (acetone). ^{1}H NMR (400 MHz, DMSO-d_6): δ 9.30 (d, J = 6.6 Hz, 2H), 8.53 (d, J = 6.6 Hz, 2H), 8.21 (dd, J = 7.7, 1.5 Hz, 1H), 7.94–7.86 (m, 3H), 7.83 (t, J = 7.4 Hz, 1H), 7.73–7.60 (m, 4H), 6.63 (s, 2H). ^{13}C NMR (101 MHz, DMSO-d_6): δ 191.9 (C), 191.2 (C), 152.2 (C), 147.2 (CH), 134.9 (CH), 134.8 (CH), 134.5 (CH), 134.2 (C), 134.1 C, 131.3 (CH), 130.3 (CH), 129.1 (CH), 128.1 (CH), 126.9 (CH), 120.1 (C), 67.5 (CH_2). HRMS (ESI) m/z: 380.0281 calcd for $C_{20}H_{15}{}^{79}BrNO_2^+$ [M – Br]$^+$, found 380.0296.

*1-(2-(2-Bromophenyl)-4-phenyl-1H-pyrrol-3-yl)-4-methylpyridin-1-ium bromide **(16b)***. Compound **16b** (319 mg, 64%) was obtained from 1-(2-(2-bromophenyl)-2-oxoethyl)-4-methylpyridin-1-ium bromide (**15b**) (395 mg, 1.06 mmol, 1 equiv), triethylamine (161 mg, 1.60 mmol, 1.5 equiv) and 3-phenyl-*2H*-azirine (**4c**) (187 mg, 1.60 mmol, 1.5 equiv) in DCM (5 mL) according to the general procedure A. The product was isolated by column chromatography on silica gel (DCM/MeOH from v/v 20:1 to 10:1). Dark red solid, mp 240 °C (dec., DCM/MeOH). ^{1}H NMR (400 MHz, DMSO-d_6): δ 12.38–12.23 (m, 1H), 8.79–8.71 (m, 2H), 7.96 (d, J = 6.4 Hz, 2H), 7.68 (dd, J = 8.1, 1.3 Hz, 1H), 7.57 (dd, J = 7.6, 1.8 Hz, 1H), 7.52–7.46 (m, 2H), 7.40 (dd, J = 7.8, 1.9 Hz, 1H), 7.35–7.29 (m, 2H), 7.27–7.23 (m, 1H), 7.10–7.06 (m, 2H), 2.63 (s, 3H). ^{13}C{1H} NMR (101 MHz, DMSO-d_6): δ 160.8 (C), 145.8 (CH), 133.3 (CH), 132.9 (CH), 131.49 (C), 131.46 (CH), 129.6 (C), 129.2 (CH), 129.1 (CH), 128.8 (CH), 128.1 (CH), 126.9 (CH), 126.5 (C), 123.6 (C), 122.8 (C), 118.8 (C), 117.6 (CH), 21.7 (CH_3). HRMS (ESI) m/z: 389.0648 calcd for $C_{22}H_{18}{}^{79}BrN_2^+$ [M – Br]$^+$, found 389.0661.

*1-(2-(2-Bromophenyl)-4-phenyl-1H-pyrrol-3-yl)-4-methoxypyridin-1-ium bromide (**16c**).* Compound **16c** (240 mg, 48%) was obtained from 1-(2-(2-bromophenyl)-2-oxoethyl)-4-methoxypyridin-1-ium bromide[11] (**15c**) (398 mg, 1.03 mmol, 1 equiv), triethylamine (156 mg, 1.54 mmol, 1.5 equiv) and 3-phenyl-*2H*-azirine (**4c**) (180 mg, 1.54 mmol, 1.5 equiv) in DCM (10 mL) according to the general procedure A. The product was isolated by column chromatography on silica gel (DCM/MeOH from v/v 20:1 to 10:1). Pale brown solid, mp 106 °C (dec., DCM/MeOH). ^{1}H NMR (500 MHz, DMSO-d_6): δ 12.21 (d, J = 3.0 Hz, 1H), 8.75–8.69 (m, 2H), 7.70 (dd, J = 8.1, 1.2 Hz, 1H), 7.62–7.58 (m, 2H), 7.56 (dd, J = 7.6, 1.8 Hz, 1H), 7.52–7.45 (m, 1H), 7.45 (d, J = 3.1 Hz, 1H), 7.40 (dd, J = 7.8, 1.9 Hz, 1H), 7.34–7.30 (m, 2H), 7.27–7.22 (m, 1H), 7.13–7.07 (m, 2H), 4.11 (s, 3H). ^{13}C{1H} NMR (126 MHz, DMSO-d_6): δ 171.3 (C), 148.2 (CH), 133.2 (CH), 132.8 (CH), 131.7 (C), 131.4 (CH), 129.8 (C), 129.0 (CH), 128.0 (CH), 126.73 (C), 126.71 (CH), 126.5 (C), 123.7 (C), 122.4 (C), 118.8 (C), 117.2 (CH), 113.8 (CH), 58.4 (CH_3). HRMS (ESI) m/z: 405.0597 calcd for $C_{22}H_{18}{}^{79}BrN_2O^+$ [M – Br]$^+$, found 405.0588.

*1-(2-(2-Bromophenyl)-4-phenyl-1H-pyrrol-3-yl)-4-(4-methoxyphenyl)pyridin-1-ium bromide (**16e**).* Compound **16e** (462 mg, 95%) was obtained from 1-(2-(2-bromophenyl)-2-oxoethyl)-4-(4-methoxyphenyl)pyridin-1-ium bromide (**15e**) (400 mg, 0.864 mmol, 1 equiv), triethylamine (131 mg, 1.30 mmol, 1.5 equiv) and 3-phenyl-*2H*-azirine (**4c**) (152 mg, 1.30 mmol, 1.5 equiv) in DCM (10 mL) according to the general procedure A. The product was isolated by column chromatography on silica gel (DCM/MeOH from v/v 20:1 to 10:1). Yellow solid, mp 209–210 °C (DCM/MeOH). ^{1}H NMR (400 MHz, DMSO-d_6): δ 12.29 (d, J = 2.8 Hz, 1H), 8.79–8.73 (m, 2H), 8.46–8.42 (m, 2H), 8.17–8.11 (m, 2H), 7.70 (dd, J = 8.0, 1.2 Hz, 1H), 7.60 (dd, J = 7.7, 1.8 Hz, 1H), 7.54–7.48 (m, 2H), 7.43–7.36 (m, 1H), 7.36–7.30 (m, 2H), 7.28–7.22 (m, 1H), 7.19–7.13 (m, 4H), 3.88 (s, 3H). ^{13}C{1H} NMR (101 MHz, DMSO-d_6): δ 163.3 (C), 154.6 (C), 146.2 (CH), 133.3 (CH), 132.9 (CH), 131.6 (C), 131.4 (CH), 130.5 (CH), 129.7 (C), 129.1 (CH), 128.2 (CH), 126.94 (CH), 126.86 (CH), 126.5 (C), 124.6 (C), 123.6 (C), 123.0 (CH), 122.7 (C), 118.8 (C), 117.7 (CH), 115.3 (CH), 55.8 (CH_3). HRMS (ESI) m/z: 481.0910 calcd for $C_{28}H_{22}{}^{79}BrN_2O^+$ [M – Br]$^+$, found 481.0895.

*1-(2-(2-Bromophenyl)-4-phenyl-1H-pyrrol-3-yl)-3,5-dimethylpyridin-1-ium bromide (**16f**).* Compound **16f** (177 mg, 35%) was obtained from 1-(2-(2-bromophenyl)-2-oxoethyl)-3,5-dimethylpyridin-1-ium bromide[11] (**15f**) (398 mg, 1.03 mmol, 1 equiv), triethylamine (156 mg, 1.55 mmol, 1.5 equiv) and 3-phenyl-*2H*-azirine (**4c**) (181 mg, 1.55 mmol, 1.5 equiv) in DCM (6 mL) according to the general procedure A. The product was isolated by column chromatography on silica gel (DCM/MeOH from v/v 20:1 to 10:1). Pale yellow solid, mp 339–340 °C

(DCM/MeOH). ^{1}H NMR (400 MHz, DMSO-d_6): δ 12.31 (br. s, 1H), 8.74 (s, 2H), 8.41 (s, 1H), 7.68 (d, J = 7.5 Hz, 1H), 7.57 (dd, J = 7.6, 1.5 Hz, 1H), 7.52–7.45 (m, 2H), 7.42–7.34 (m, 1H), 7.35–7.27 (m, 2H), 7.27–7.21 (m, 1H), 7.08 (d, J = 7.1 Hz, 2H), 2.35 (s, 6H). ^{13}C{1H} NMR (101 MHz, DMSO-d_6): δ 147.9 (CH), 143.7 (CH), 138.4 (C), 133.2 (CH), 132.8 (CH), 131.48 (C), 131.45 (CH), 129.5 (C), 129.0 (CH), 128.0 (CH), 126.9 (CH), 126.7 (C), 126.6 (CH), 123.6 (C), 123.0 (C), 118.7 (C), 117.4 (CH), 17.5 (CH_3). HRMS (ESI) m/z: 403.0804 calcd for $C_{23}H_{20}{}^{79}BrN_2^+$ [M – Br]$^+$, found 403.0819.

*2-(2-(2-Bromophenyl)-4-phenyl-1H-pyrrol-3-yl)isoquinolin-2-ium bromide (**16g**).* Compound **16g** (214 mg, 44%) was obtained from 2-(2-(2-bromophenyl)-2-oxoethyl)isoquinolin-2-ium bromide (**15g**) (388 mg, 0.956 mmol, 1 equiv), triethylamine (145 mg, 1.43 mmol, 1.5 equiv) and 3-phenyl-*2H*-azirine (**4c**) (168 mg, 1.43 mmol, 1.5 equiv) in DCM (6 mL) according to the general procedure A. The product was isolated by column chromatography on silica gel (DCM/MeOH from v/v 20:1 to 10:1). Pale orange solid, mp 182–183 °C (DCM/MeOH). ^{1}H NMR (500 MHz, DMSO-d_6): δ 12.39 (br. s, 1H), 10.02 (s, 1H), 8.64–8.54 (m, 2H), 8.42 (d, J = 8.3 Hz, 1H), 8.38 (d, J = 8.2 Hz, 1H), 8.33–8.27 (m, 1H), 8.08–8.02 (m, 1H), 7.66–7.59 (m, 2H), 7.56 (d, J = 2.9 Hz, 1H), 7.48 (m, J = 7.3 Hz, 1H), 7.46–7.24 (m, 1H), 7.27–7.21 (m, 3H), 7.11 (d, J = 7.1 Hz, 2H). ^{13}C{1H} NMR (126 MHz, DMSO-d_6): δ 151.7 (CH), 138.1 (CH), 137.1 (CH), 136.9 (C), 133.3 (CH), 132.8 (CH), 131.8 (CH), 131.5 (C), 131.4 (CH), 130.7 (CH), 129.5 (C), 129.1 (CH), 128.2 (CH), 127.5 (CH), 127.3 (C), 126.9 (CH), 126.3 (CH), 123.43 (C), 123.28 (C), 119.0 (C), 117.8 (CH). HRMS (ESI) m/z: 425.0648 calcd for $C_{25}H_{18}{}^{79}BrN_2^+$ [M – Br]$^+$, found 425.0658.

*1-(2-(2-Bromophenyl)-4-(4-methoxyphenyl)-1H-pyrrol-3-yl)pyridin-1-ium bromide (**16h**).* Compound **16h** (249 mg, 50%) was obtained from 1-(2-(2-bromophenyl)-2-oxoethyl)pyridin-1-ium bromide (**15h**) (367 mg, 1.02 mmol, 1 equiv), triethylamine (156 mg, 1.54 mmol, 1.5 equiv) and 3-(4-methoxyphenyl)-*2H*-azirine (**4d**) (227 mg, 1.54 mmol, 1.5 equiv) in DCM (5 mL) according to the general procedure **A**. The product was isolated by column chromatography on silica gel (DCM/MeOH from v/v 20:1 to 10:1). Bright yellow solid, mp 254–255 °C (DCM/MeOH). ^{1}H NMR (400 MHz, DMSO-d_6): δ 12.28 (br. s, 1H), 8.87 (d, J = 5.5 Hz, 2H), 8.68 (t, J = 7.9 Hz, 1H), 8.15 (dd, J = 7.6, 6.8 Hz, 2H), 7.67 (dd, J = 8.0, 0.8 Hz, 1H), 7.57 (dd, J = 7.6, 1.6 Hz, 1H), 7.53–7.47 (m, 1H), 7.43–7.34 (m, 2H), 7.06–6.96 (m, 2H), 6.93–6.83 (m, 2H), 3.73 (s, 3H). ^{13}C{1H} NMR (101 MHz, DMSO-d_6): δ 158.3 (C), 146.8 (CH), 133.3 (CH), 132.9 (CH), 131.4 (CH), 128.6 (CH), 128.3 (CH), 128.2 (CH), 126.2 (C), 123.5 (C), 123.4 (C),

123.2 (C), 117.2 (CH), 114.5 (CH), 55.1 (CH_3). HRMS (ESI) m/z: 405.0597 calcd for $C_{22}H_{18}{}^{79}BrN_2O^+$ [M – Br]$^+$, found 405.0614.

*1-(2-(2-Bromophenyl)-4-phenyl-1H-pyrrol-3-yl)-4-cyanopyridin-1-ium bromide (**16i**).* Compound **16i** (46 mg, 18%) was obtained from 1-(2-(2-bromophenyl)-2-oxoethyl)-4-cyanopyridin-1-ium bromide[11] (**15i**) (200 mg, 0.523 mmol, 1 equiv), $NiBr_2{\cdot}3H_2O$ (28.5 mg, 0.105 mmol, 0.2 equiv) and 3-phenyl-*2H*-azirine (**4c**) (92 mg, 0.785 mmol, 1.5 equiv) in acetone (10 mL) according to the general procedure B. Orange solid, mp 276–278 °C (acetone). ^{1}H NMR (400 MHz, DMSO-d_6): δ 12.49 (br. s, 1H), 9.13 (d, J = 6.2 Hz, 2H), 8.66 (d, J = 6.3 Hz, 2H), 7.68 (d, J = 8.1 Hz, 1H), 7.60 (d, J = 7.5 Hz, 1H), 7.55–7.49 (m, 2H), 7.42 (d, J = 7.8 Hz, 1H), 7.35–7.26 (m, 3H), 7.14 (d, J = 7.3 Hz, 2H). ^{13}C{1H} NMR (101 MHz, DMSO-d_6): δ 147.0 (CH), 133.0 (CH), 132.4 (CH), 131.1 (CH), 131.0 (CH), 130.2 (C), 128.6 (CH), 128.2 (C), 127.8 (CH), 127.2 (C), 126.6 (CH), 126.5 (CH), 126.4 (C), 122.7 (C), 122.4 (C), 118.1 (C), 117.9 (CH), 114.1 (C). HRMS (ESI) m/z: 400.0444 calcd for $C_{22}H_{15}{}^{79}BrN_3^+$ [M – Br]$^+$, found 400.0428.

*4-Benzoyl-1-(2-(2-bromophenyl)-4-phenyl-1H-pyrrol-3-yl)pyridin-1-ium bromide (**16j**).* Compound **16j** (219 mg, 45%) was obtained from 4-benzoyl-1-(2-(2-bromophenyl)-2-oxoethyl)pyridin-1-ium bromide (**15j**) (400 mg, 0.868 mmol, 1 equiv), $NiBr_2{\cdot}3H_2O$ (47 mg, 0.17 mmol, 0.2 equiv) and 3-phenyl-*2H*-azirine (**4c**) (203 mg, 1.74 mmol, 1.5 equiv) in acetone (20 mL) according to the general procedure B. Bright orange solid, mp 257–258 °C (acetone). ^{1}H NMR (400 MHz, DMSO-d_6): δ 12.44 (br. s, 1H), 9.04 (d, J = 6.4 Hz, 2H), 8.35 (d, J = 6.4 Hz, 2H), 7.80–7.78 (m, 3H), 7.72 (d, J = 7.8 Hz, 1H), 7.68–7.61 (m, 3H), 7.56–7.54 (m, 2H), 7.47–7.40 (m, 1H), 7.39–7.35 (m, 2H), 7.30–7.35 (m, 1H), 7.21 (d, J = 7.2 Hz, 2H). ^{13}C{1H} NMR (101 MHz, DMSO-d_6): δ 191.6 (C), 152.0 (C), 147.5 (CH), 134.8 (CH), 134.0 (C), 133.4 (CH), 133.0 (CH), 131.6 (CH), 131.1 (C), 130.1 (CH), 129.2 (CH), 129.1 (CH), 128.3 (CH), 127.5 (CH), 127.4 (CH), 127.1 (CH), 126.8 (C), 123.3 (C), 123.0 (C), 118.8 (C), 118.3 (CH). HRMS (ESI) m/z: 479.0754 calcd for $C_{28}H_{20}{}^{79}BrN_2O^+$ [M – Br]$^+$, found 479.0775.

*3-Phenylpyrido[2,1-a]pyrrolo[3,2-c]isoquinolin-1-ium bromide (**17a**).* Compound **17a** (120 mg, 77%) was obtained from 1-(2-(2-bromophenyl)-4-phenyl-1*H*-pyrrol-3-yl)pyridin-1-ium bromide (**16a**) (190 mg, 0.42 mmol, 1 equiv), TTMSS (155 mg, 0.62 mmol, 1.5 equiv) and AIBN (137 mg, 0.83 mmol, 2 equiv) according to the general procedure C. Yellow solid, mp 343–344 °C (dec., MeCN/EtOAc). ^{1}H NMR (400 MHz, DMSO-d_6): δ 13.57 (d, J = 3.1 Hz, 1H), 9.56 (d, J = 8.7 Hz, 1H), 9.18–9.13 (m, 2H), 8.67 (d, J = 8.1 Hz, 1H), 8.48 (ddd, J = 8.7, 7.2, 1.3 Hz, 1H), 8.18–8.12 (m, 1H), 8.07–8.01 (m, 1H), 7.93–7.86 (m, 2H), 7.66–7.53 (m, 5H). ^{13}C{1H} NMR

(101 MHz, DMSO-d_6): δ 139.8 (C), 136.8 (CH), 133.7 (CH), 133.3 (CH), 132.8 (C), 130.2 (CH), 129.1 (CH), 128.2 (CH), 127.8 (CH), 126.5 (CH), 126.4 (CH), 124.5 (C), 124.2 (CH), 124.1 (C), 123.5 (CH), 121.2 (C), 121.0 (CH), 120.5 (C), 115.0 (C). HRMS (ESI) m/z: 295.1230 calcd for $C_{21}H_{15}N_2^+$ [M – Br]$^+$, found 295.1234.

*7-Methyl-3-phenylpyrido[2,1-a]pyrrolo[3,2-c]isoquinolin-1-ium bromide (**17b**)*. Compound **17b** (43 mg, 46%) was obtained from 1-(2-(2-bromophenyl)-4-phenyl-1*H*-pyrrol-3-yl)-4-methylpyridin-1-ium bromide (**16b**) (100 mg, 0.215 mmol, 1 equiv), TTMSS (80 mg, 0.32 mmol, 1.5 equiv) and AIBN (70 mg, 0.43 mmol, 2 equiv) according to the general procedure C. Pale yellow solid, mp > 400 °C (MeCN/EtOAc). ^{1}H NMR (400 MHz, DMSO-d_6): δ 13.45 (d, J = 3.1 Hz, 1H), 9.43 (s, 1H), 9.14 (d, J = 8.5 Hz, 1H), 9.01 (d, J = 7.0 Hz, 1H), 8.62 (d, J = 8.1 Hz, 1H), 8.18–8.11 (m, 1H), 7.94–7.84 (m, 3H), 7.64–7.53 (m, 5H), 2.72 (s, 3H). ^{13}C{1H} NMR (101 MHz, DMSO-d_6): δ 149.6 (C), 139.8 (C), 133.7 (CH), 132.9 (C), 132.7 (CH), 130.2 (CH), 129.1 (CH), 128.2 (CH), 127.7 (CH), 126.6 (CH), 126.1 (CH), 124.9 (CH), 124.4 (C), 124.0 (C), 123.6 (CH), 121.1 (C), 120.9 (CH), 120.3 (C), 114.9 (C), 21.2 (CH_3). HRMS (ESI) m/z: 309.1386 calcd for $C_{22}H_{17}N_2^+$ [M – Br]$^+$, found 309.1399.

*7-Methoxy-3-phenylpyrido[2,1-a]pyrrolo[3,2-c]isoquinolin-1-ium bromide (**17c**)*. Compound **17c** (86 mg, 60%) was obtained from 1-(2-(2-bromophenyl)-4-phenyl-1*H*-pyrrol-3-yl)-4-methoxypyridin-1-ium bromide (**16c**) (173 mg, 0.357 mmol, 1 equiv), TTMSS (133 mg, 0.535 mmol, 1.5 equiv) and AIBN (117 mg, 0.713 mmol, 2 equiv) according to the general procedure C. Pale yellow solid, mp 296–298 °C (MeCN/EtOAc). ^{1}H NMR (400 MHz, DMSO-d_6): δ 13.31 (d, J = 3.1 Hz, 1H), 9.15 (d, J = 8.6 Hz, 1H), 8.95 (d, J = 7.6 Hz, 1H), 8.75 (d, J = 3.0 Hz, 1H), 8.55 (dd, J = 8.2, 1.2 Hz, 1H), 8.13–8.06 (m, 1H), 7.82 (ddd, J = 8.4, 7.1, 1.2 Hz, 1H), 7.78–7.73 (m, 2H), 7.63–7.52 (m, 5H), 4.22 (s, 3H). ^{13}C{1H} NMR (101 MHz, DMSO-d_6): δ 164.9 (C), 142.9 (C), 135.4 (CH), 133.8 (CH), 133.0 (C), 130.2 (CH), 129.1 (CH), 128.1 (CH), 127.3 (CH), 127.0 (CH), 125.5 (CH), 124.5 (C), 122.6 (C), 120.9 (C), 120.8 (CH), 120.0 (C), 114.6 (C), 113.4 (CH), 106.0 (CH), 57.8 (CH_3). HRMS (ESI) m/z: 325.1335 calcd for $C_{22}H_{17}N_2O^+$ [M – Br]$^+$, found 325.1329.

*3,7-Diphenylpyrido[2,1-a]pyrrolo[3,2-c]isoquinolin-1-ium bromide (**17d**)*. Compound **17d** (58 mg, 69%) was obtained from 1-(2-(2-bromophenyl)-4-phenyl-1*H*-pyrrol-3-yl)-4-phenylpyridin-1-ium bromide (**16d**)[4] (100 mg, 0.19 mmol, 1 equiv), TTMSS (71 mg, 0.28 mmol, 1.5 equiv) and AIBN (62 mg, 0.38 mmol, 2 equiv) according to the general procedure C. Bright yellow, mp > 400 °C (MeCN/EtOAc). ^{1}H NMR (400 MHz, DMSO-d_6): δ 13.53 (d, J = 1.4 Hz, 1H), 9.68 (d, J = 1.7 Hz, 1H), 9.47 (d, J = 8.6 Hz, 1H), 9.16 (d, J = 7.2 Hz, 1H), 8.65 (d, J = 8.0 Hz, 1H), 8.50

(dd, J = 7.3, 2.0 Hz, 1H), 8.27 (dd, J = 7.6, 1.9 Hz, 2H), 8.21–8.15 (m, 1H), 7.96–7.88 (m, 2H), 7.70–7.56 (m, 8H). ^{13}C{1H} NMR (101 MHz, DMSO-d_6): δ 146.7 (C), 140.4 (C), 134.3 (C), 133.9 (CH), 133.6 (CH), 131.2 (CH), 130.3 (CH), 129.5 (CH), 129.2 (CH), 128.3 (CH), 128.0 (CH), 127.7 (CH), 127.3 (CH), 126.5 (CH), 124.5 (C), 121.7 (C), 120.9 (CH), 120.5 (C), 119.8 (CH), 115.0 (C). HRMS (ESI) m/z: 371.1543 calcd for $C_{27}H_{19}N_2^+$ [M – Br]$^+$, found 371.1554.

*7-(4-Methoxyphenyl)-3-phenylpyrido[2,1-a]pyrrolo[3,2-c]isoquinolin-1-ium bromide (**17e**).* Compound **17e** (66 mg, 46%) was obtained from 1-(2-(2-bromophenyl)-4-phenyl-1*H*-pyrrol-3-yl)-4-(4-methoxyphenyl)pyridin-1-ium bromide (**16e**) (170 mg, 0.302 mmol, 1 equiv), TTMSS (113 mg, 0.456 mmol, 1.5 equiv) and AIBN (99 mg, 0.60 mmol, 2 equiv) according to the general procedure C. Bright yellow solid, mp 295–296 °C (MeCN/EtOAc). ^{1}H NMR (400 MHz, DMSO-d_6): δ 13.48–13.43 (m, 1H), 9.53 (d, J = 2.2 Hz, 1H), 9.41 (d, J = 8.6 Hz, 1H), 9.05 (d, J = 7.3 Hz, 1H), 8.60 (d, J = 8.1 Hz, 1H), 8.42 (dd, J = 7.3, 2.1 Hz, 1H), 8.30–8.22 (m, 2H), 8.16–8.09 (m, 1H), 7.92–7.83 (m, 2H), 7.70–7.53 (m, 5H), 7.19–7.10 (m, 2H), 3.88 (s, 3H). ^{13}C{1H} NMR (101 MHz, DMSO-d_6): δ 162.0 (C), 146.3 (C), 140.3 (C), 133.7 (CH), 133.3 (CH), 133.0 (C), 130.3 (CH), 129.7 (CH), 129.2 (CH), 128.2 (CH), 127.6 (CH), 127.1 (CH), 126.3 (CH), 126.1 (C), 124.4 (C), 124.1 (C), 121.6 (C), 120.8 (CH), 120.3 (C), 120.0 (CH), 118.2 (CH), 114.91 (CH), 114.86 (C), 55.6 (CH_3). HRMS (ESI) m/z: 401.1648 calcd for $C_{28}H_{21}N_2O^+$ [M – Br]$^+$, found 401.1644.

*6,8-Dimethyl-3-phenylpyrido[2,1-a]pyrrolo[3,2-c]isoquinolin-1-ium bromide (**17f**).* Compound **17f** (52 mg, 64%) was obtained from 1-(2-(2-bromophenyl)-4-phenyl-1*H*-pyrrol-3-yl)-3,5-dimethylpyridin-1-ium bromide (**16f**) (100 mg, 0.207mmol, 1 equiv), TTMSS (77 mg, 0.31 mmol, 1.5 equiv) and AIBN (68 mg, 0.41 mmol, 2 equiv) according to the general procedure C. Bright yellow solid, mp > 400 °C (MeCN/EtOAc). ^{1}H NMR (400 MHz, DMSO-d_6): δ 13.45 (br. s, 1H), 9.00 (d, J = 8.7 Hz, 1H), 8.94 (s, 1H), 8.65 (d, J = 7.9 Hz, 1H), 8.35 (s, 1H), 8.17–8.09 (m, 1H), 7.89 (s, 1H), 7.89–7.83 (m, 1H), 7.66–7.50 (m, 5H), 3.19 (s, 3H), 2.26 (s, 3H). ^{13}C{1H} NMR (101 MHz, DMSO-d_6): δ 142.4 (CH), 138.5 (C), 136.3 (C), 133.0 (C), 132.7 (CH), 131.9 (C), 131.7 (CH), 130.2 (CH), 129.8 (CH), 129.0 (CH), 128.2 (CH), 126.6 (CH), 126.4 (CH), 124.8 (C), 124.6 (C), 120.8 (CH), 120.7 (C), 115.2 (C), 26.0 (CH_3), 17.4 (CH_3). HRMS (ESI) m/z: 323.1543 calcd for $C_{23}H_{19}N_2^+$[M – Br]$^+$, found 323.1548.

*3-Phenyl-1H-isoquinolino[1,2-a]pyrrolo[3,2-c]isoquinolin-4-ium bromide (**17g**).* Compound (40 mg, 49%) **17g** was obtained from 2-(2-(2-bromophenyl)-4-phenyl-1*H*-pyrrol-3-yl)isoquinolin-2-ium bromide (**16g**) (120 mg, 0.237 mmol, 1 equiv), TTMSS (89 mg, 0.36 mmol, 1.5 equiv) and AIBN (75 mg, 0.47 mmol, 2 equiv) according to the general procedure C. Pale orange solid, mp

250–253 °C (dec., MeCN/EtOAc). ^{1}H NMR (400 MHz, DMSO-d_6): δ 13.69 (d, J = 2.9 Hz, 1H), 9.13–9.05 (m, 2H), 8.83–8.76 (m, 2H), 8.33–8.27 (m, 3H), 8.19–8.15 (m, 1H), 8.12–8.07 (m, 2H), 7.99–7.95 (m, 1H), 7.66–7.57 (m, 5H). $^{13}C\{1H\}$ NMR (101 MHz, DMSO-d_6): δ 141.7 (C), 134.4 (CH), 133.2 (CH), 132.8 (C), 132.7 (C), 132.2 (CH), 131.2 (CH), 130.5 (CH), 130.3 (CH), 129.0 (CH), 128.2 (CH), 128.1 (CH), 127.6 (CH), 127.5 (CH), 127.1 (CH), 125.9 (C), 125.5 (C), 124.9 (C), 122.0 (C), 121.4 (CH), 121.09 (C), 121.06 (CH), 114.8 (C). HRMS (ESI) m/z: 345.1386 calcd for $C_{25}H_{17}N_2^+$ [M – Br]$^+$, found 345.1398.

*3-(4-Methoxyphenyl)pyrido[2,1-a]pyrrolo[3,2-c]isoquinolin-1-ium bromide (**17h**).* Compound **17h** (111 mg, 67%) was obtained from 1-(2-(2-bromophenyl)-4-(4-methoxyphenyl)-1*H*-pyrrol-3-yl)pyridin-1-ium bromide (**16h**) (200 mg, 0.412 mmol, 1 equiv), TTMSS (152 mg, 0.617 mmol, 1.5 equiv) and AIBN (135 mg, 0.823 mmol, 2 equiv) according to the general procedure C. Yellow solid, mp 249–251 °C (MeCN/EtOAc). ^{1}H NMR (400 MHz, DMSO-d_6): δ 13.52–13.47 (m, 1H), 9.54 (d, J = 8.8 Hz, 1H), 9.22 (d, J = 6.7 Hz, 1H), 9.13 (d, J = 8.5 Hz, 1H), 8.65 (d, J = 8.1 Hz, 1H), 8.51–8.42 (m, 1H), 8.19–8.11 (m, 1H), 8.08–8.02 (m, 1H), 7.93–7.86 (m, 1H), 7.83 (d, J = 2.9 Hz, 1H), 7.54 (d, J = 8.2 Hz, 2H), 7.16 (d, J = 8.2 Hz, 2H), 3.88 (s, 3H). $^{13}C\{1H\}$ NMR (101 MHz, DMSO-d_6): δ 159.2 (C), 139.9 (C), 136.8 (CH), 133.8 (CH), 133.3 (CH), 131.6 (CH), 127.8 (CH), 126.5 (CH), 126.4 (CH), 124.7 (C), 124.4 (C), 124.3 (C), 124.2 (CH), 123.5 (CH), 121.3 (C), 120.9 (CH), 120.7 (C), 114.7 (C), 114.6 (CH), 55.3 (CH_3). HRMS (ESI) m/z: 325.1335 calcd for $C_{22}H_{17}N_2O^+$[M – Br]$^+$, found 325.1341.

*3-Phenylpyrido[2,1-a]pyrrolo[3,2-c]isoquinoline (**18a**).* Compound **18a** (47 mg, 99%) was obtained from 3-phenylpyrido[2,1-a]pyrrolo[3,2-c]isoquinolin-4-ium bromide (**17a**) (60 mg, 0.16mmol) according to the general procedure D. Orange solid, mp > 400 °C (DCM). ^{1}H NMR (400 MHz, DMSO-d_6): δ 9.41 (d, J = 8.8 Hz, 1H), 9.17 (d, J = 6.8 Hz, 1H), 9.01 (d, J = 8.6 Hz, 1H), 8.70 (d, J = 8.2 Hz, 1H), 8.24–8.14 (m, 1H), 8.04–7.97 (m, 1H), 7.87 (d, J = 6.9 Hz, 1H), 7.73–7.65 (m, 2H), 7.58–7.50 (m, 3H), 7.46–7.39 (m, 1H). $^{13}C\{1H\}$ NMR (101 MHz, DMSO-d_6): δ 140.1 (CH), 136.4 (C), 136.3 (C), 134.0 (C), 131.9 (CH), 131.3 (CH), 131.2 (CH), 129.72 (CH), 129.66 (C), 128.7 (CH), 126.0 (CH), 125.4 (CH), 124.5 (CH), 123.5 (C), 121.9 (C), 121.6 (CH), 121.1 (CH), 118.8 (C), 112.8 (C). HRMS (ESI) m/z: 295.1230 calcd for $C_{21}H_{15}N_2^+$[M + H]$^+$, found 295.1234.

*1-Methyl-3-phenylpyrido[2,1-a]pyrrolo[3,2-c]isoquinolin-4-ium iodide (N-Me-**18a**).*[4] Compound *N*-Me-**18a** (74 mg, 100%) was obtained from phenylpyrido[2,1-*a*]pyrrolo[3,2-*c*]isoquinoline (**18a**) (50 mg, 0.17 mmol) and methyl iodide (24.5 mg, 0.17 mmol, 1 equiv) in dry DCM (8 mL) likewise *N*-Me-**3a**.

*1-Benzyl-3-phenylpyrido[2,1-a]pyrrolo[3,2-c]isoquinolin-4-ium bromide (N-Bn-**18a**).*[4] Compound *N*-Bn-**18a** (55 mg, 100%) was obtained from 3-phenylpyrido[2,1-*a*]pyrrolo[3,2-*c*]isoquinoline (**18a**) (35 mg, 0.12 mmol), benzyl bromide (21 mg, 0.12 mmol, 1 equiv) and K_2CO_3 (33 mg, 0.24 mmol, 2 equiv) in dry acetonitrile (5 mL) likewise *N*-Bn-**3a**.

7-Methoxy-3-phenylpyrido[2,1-a]pyrrolo[3,2-c]isoquinoline (**18c**). Compound **18c** (21 mg, 97%) was obtained from 7-methoxy-3-phenylpyrido[2,1-*a*]pyrrolo[3,2-*c*]isoquinolin-4-ium bromide (**17c**) (27 mg, 0.067 mmol) according to the general procedure D. Orange solid, mp 225–227 °C (DCM). ^{1}H NMR (400 MHz, DMSO-d_6): δ 9.02 (d, J = 7.7 Hz, 1H), 8.93 (d, J = 8.6 Hz, 1H), 8.59–8.53 (m, 2H), 7.88–7.82 (m, 1H), 7.58–7.45 (m, 6H), 7.43 (s, 1H), 7.34 (ddd, J = 10.2, 6.1, 4.1 Hz, 1H), 4.16 (s, 3H). ^{13}C{1H} NMR (126 MHz, DMSO-d_6): δ 161.4 (C), 139.9 (C), 136.4 (C), 133.6 (CH), 133.5 (C), 132.2 (CH), 130.1 (CH), 130.0 (C), 129.7 (CH), 128.7 (CH), 126.1 (CH), 126.0 (CH), 124.0 (CH), 121.0 (C), 120.9 (CH), 118.4 (C), 112.6 (CH), 112.5 (C), 104.5 (CH), 57.1 (CH_3). HRMS (ESI) m/z: 325.1335 calcd for $C_{22}H_{17}N_2O^+$[M + H]$^+$, found 325.1350.

3,7-Diphenylpyrido[2,1-a]pyrrolo[3,2-c]isoquinoline (**18d**). Compound **18d** (30 mg, 99%) was obtained from 3,7-diphenylpyrido[2,1-*a*]pyrrolo[3,2-*c*]isoquinolin-4-ium bromide (**17d**) (37 mg, 0.082 mmol) according to the general procedure D. Red solid, mp 224–225 °C (DCM). ^{1}H NMR (400 MHz, DMSO-d_6): δ 9.48 (d, J = 2.4 Hz, 1H), 9.24–9.12 (m, 2H), 8.65 (d, J = 8.2 Hz, 1H), 8.25–8.14 (m, 3H), 7.94–7.85 (m, 1H), 7.64–7.47 (m, 9H), 7.41–7.33 (m, 1H). ^{13}C{1H} NMR (101 MHz, DMSO-d_6): δ 141.2 (CH), 140.9 (C), 136.7 (C), 136.4 (C), 135.2 (C), 134.8 (CH), 131.8 (CH), 131.3 (CH), 130.3 (C), 130.0 (CH), 129.7 (CH), 129.3 (CH), 128.7 (CH), 127.4 (CH), 126.0 (CH), 125.9 (CH), 124.2 (CH), 121.8 (C), 121.0 (CH), 119.3 (CH), 119.20 (CH), 119.06 (C), 112.6 (C). HRMS (ESI) m/z: 371.1543 calcd for $C_{27}H_{19}N_2^+$ [M + H]$^+$, found 371.1534.

7-(4-Methoxyphenyl)-3-phenylpyrido[2,1-a]pyrrolo[3,2-c]isoquinoline (**18e**). Compound **18e** (21 mg, 99%) was obtained from 7-(4-methoxyphenyl)-3-phenylpyrido[2,1-*a*]pyrrolo[3,2-*c*]isoquinolin-4-ium bromide (**17e**) (25 mg, 0.052 mmol) according to the general procedure D. Red solid, mp > 400 °C (DCM). ^{1}H NMR (400 MHz, DMSO-d_6): δ 9.43 (d, J = 2.2 Hz, 1H), 9.21 (d, J = 8.6 Hz, 1H), 9.13 (d, J = 7.3 Hz, 1H), 8.67–8.62 (m, 1H), 8.25–8.18 (m, 3H), 7.93–7.87 (m, 1H), 7.65–7.59 (m, 1H), 7.58–7.49 (m, 5H), 7.41–7.36 (m, 1H), 7.19–7.12 (m, 2H), 3.88 (s, 3H). ^{13}C{1H} NMR (101 MHz, DMSO-d_6): δ 161.0 (C), 141.0 (C), 139.7 (CH), 136.8 (C), 136.5 (C), 133.7 (C), 131.8 (CH), 131.3 (CH), 129.9 (C), 129.8 (CH), 128.9 (CH), 128.7 (CH), 127.2 (C), 126.0 (CH), 124.2 (CH), 121.5 (C), 121.0 (CH), 119.1 (C), 118.6 (CH), 117.9

(CH), 114.7 (CH), 112.7 (C), 55.4 (CH_3). HRMS (ESI) m/z: 401.1648 calcd for $C_{28}H_{21}N_2O^+$[M + H]$^+$, found 401.1662.

6,8-Dimethyl-3-phenylpyrido[2,1-a]pyrrolo[3,2-c]isoquinoline (**18f**). Compound **18f** (28 mg, 99%) was obtained from 6,8-dimethyl-3-phenylpyrido[2,1-*a*]pyrrolo[3,2-*c*]isoquinolin-4-ium bromide (**17f**) (35 mg, 0.087 mmol) according to the general procedure D. Orange solid, mp 220–222 °C (DCM). ^{1}H NMR (400 MHz, DMSO-d_6): δ 8.97 (s, 1H), 8.83 (d, J = 8.8 Hz, 1H), 8.73–8.67 (m, 1H), 7.92 (s, 1H), 7.90–7.84 (m, 1H), 7.60–7.45 (m, 6H), 7.40–7.33 (m, 1H), 3.15 (s, 3H), 2.25 (s, 3H). ^{13}C{1H} NMR (101 MHz, DMSO-d_6): δ 137.1 (CH), 136.6 (C), 135.3 (C), 134.4 (C), 130.8 (CH), 130.6 (C), 129.9 (C), 129.6 (CH), 129.4 (CH), 128.7 (CH), 128.5 (CH), 125.9 (C), 123.1 (CH), 122.2 (C), 121.0 (CH), 119.5 (C), 112.8 (C), 26.2 (CH_3), 17.4 (CH_3). HRMS (ESI) m/z: 323.1543 calcd for $C_{23}H_{19}N_2^+$[M + H]$^+$, found 323.1547.

3-Phenylisoquinolino[1,2-a]pyrrolo[3,2-c]isoquinoline (**18g**).Compound **18g** (25 mg, 99%) was obtained from 3-phenylisoquinolino[1,2-*a*]pyrrolo[3,2-*c*]isoquinolin-4-ium bromide (**17g**) (31 mg, 0.073 mmol) according to the general procedure D. Dark purple solid, mp 176–178 °C (DCM). ^{1}H NMR (400 MHz, DMSO-d_6): δ 9.01–8.92 (m, 2H), 8.84–8.76 (m, 2H), 8.31 (s, 1H), 8.22–8.17 (m, 1H), 8.08–7.96 (m, 3H), 7.85 (s, 1H), 7.74–7.67 (m, 1H), 7.59–7.50 (m, 4H), 7.45–7.39 (m, 1H). ^{13}C{1H} NMR (101 MHz, DMSO-d_6): δ 135.4 (C), 132.49 (C), 132.47 (CH), 131.4 (CH), 131.2 (C), 130.7 (CH), 130.4 (CH), 129.8 (CH), 129.7 (CH), 129.6, 128.7 (CH), 127.3 (CH), 127.0 (CH), 126.6 (CH), 126.0 (C), 125.3 (CH), 125.2 (CH), 123.4 (C), 121.4 (CH), 119.8 (CH), 119.2 (C), 112.9 (C). HRMS (ESI) m/z: 345.1386 calcd for $C_{25}H_{17}N_2^+$[M + H]$^+$, found 345.1391.

3-(4-Methoxyphenyl)pyrido[2,1-a]pyrrolo[3,2-c]isoquinoline (**18h**). Compound **18h** (56 mg, 100%) was obtained from 3-(4-methoxyphenyl)pyrido[2,1-*a*]pyrrolo[3,2-*c*]isoquinolin-4-ium bromide (**17h**) (70 mg, 0.17 mmol) according to the general procedure D. Saturated red solid, mp 169–170 °C (DCM). ^{1}H NMR (400 MHz, DMSO-d_6): δ 9.32 (d, J = 8.9 Hz, 1H), 9.18 (d, J = 6.8 Hz, 1H), 8.92 (d, J = 8.6 Hz, 1H), 8.65 (d, J = 8.2 Hz, 1H), 8.14–8.03 (m, 1H), 7.94–7.87 (m, 1H), 7.83–7.76 (m, 1H), 7.66–7.56 (m, 1H), 7.55 (s, 1H), 7.43 (d, J = 8.1 Hz, 2H), 7.07 (d, J = 8.2 Hz, 2H), 3.84 (s, 3H). ^{13}C{1H} NMR (101 MHz, DMSO-d_6): δ 158.0 (C), 139.3 (CH), 136.4 (C), 133.2 (C), 131.9 (CH), 131.3 (CH), 131.09 (CH), 131.06 (CH), 129.5 (C), 128.3 (C), 125.5 (CH), 124.6 (CH), 123.6 (CH), 121.9 (C), 121.7 (CH), 121.1 (CH), 118.8 (C), 114.2 (CH), 112.4, 55.1 (CH_3). HRMS (ESI) m/z: 325.1335 calcd for $C_{22}H_{17}N_2O^+$[M + H]$^+$, found 325.1346.

2-Phenylpyrido[2,1-a]pyrrolo[3,2-c]isoquinoline (**19**).[4] 3-Phenylpyrido[2,1-*a*]pyrrolo[3,2-*c*]isoquinoline (**18a**) (70 mg, 0.24 mmol) was dissolved in dry benzene (3 mL), argon was bubbled through reaction mixture for 10 minutes and $AlCl_3$ (160 mg, 1.19 mmol, 5 equiv) was added in one portion. The reaction mixture was refluxed for 3 h, then it was quenched with aq 10% NaOH and extracted 3 times with DCM. The combined DCM phases were washed with brine and dried under Na_2SO_4, filtered and evaporated under reduced pressure. The residue was washed with Et_2O to produce **19** (63 mg, 90%), red solid, mp 164–165°C (DCM).

*1-Methyl-2-phenylpyrido[2,1-a]pyrrolo[3,2-c]isoquinolin-4-ium iodide (N-Me-**19**).* Compound *N*-Me-**19** (44 mg, 100%) was obtained from 2-phenylpyrido[2,1-*a*]pyrrolo[3,2-*c*]isoquinoline (**19**) (30 mg, 0.10 mmol) and methyl iodide (14.5 mg, 0.10 mmol, 1 equiv) in dry DCM (5 mL) likewise *N*-Me-**3a**. Orange solid, mp 165–166 °C (DCM). ^{1}H NMR (400 MHz, DMSO-d_6): δ 9.88 (d, J = 6.6 Hz, 1H), 9.59 (d, J = 8.8 Hz, 1H), 9.27 (d, J = 8.5 Hz, 1H), 8.84 (d, J = 8.4 Hz, 1H), 8.59–8.52 (m, 1H), 8.30–8.26 (m, 1H), 8.22–8.18 (m, 1H),7.99–7.93 (m, 1H), 7.86 (s, 1H), 7.73 (d, J = 6.3 Hz, 2H), 7.70–7.57 (m, 3H), 4.31 (s, 3H). ^{13}C{1H} NMR (101 MHz, DMSO-d_6): δ 142.6 (C), 137.2 (CH), 134.4 (CH), 133.7 (CH), 130.1 (C), 129.6 (CH), 129.3 (CH), 129.1 (CH), 127.4 (CH), 127.2 (CH), 125.4 (C), 124.7 (C), 124.3 (C), 124.2 (CH), 123.8 (CH), 121.8 (CH), 121.6 (CH), 97.8 (CH), 36.7 (CH_3). HRMS (ESI) m/z: 309.1388 calcd for $C_{22}H_{17}N_2^+$ [M – I]$^+$, found 309.1397.

REFERENCES

1. Hortmann, A.G.; Robertson, D. A.; Gillard, B. K. *J. Org. Chem.*, **1972**, *37*, 322–324. doi:10.1021/jo00967a033
2. Funt, L. D.; Tomashenko, O. A.; Novikov, M. S.; Khlebnikov, A. F. *Synthesis* **2018**, 50, 4809–4822. doi:10.1055/s-0037-1610840
3. Basnet, A.; Thapa, P.; Karki, R.; Na, Y.; Jahng, Y.; Jeong, B.; Jeong, T.; Lee, J.; Lee, E. *Bioorg. Med. Chem.* **2007**, *15*, 4351–4359. doi:10.1016/j.bmc.2007.04.047
4. Tomashenko, O. A.; Khlebnikov, A. F.; Mosiagin, I. P.; Novikov, M. S.; Grachova, E. V.; Shakirova, J. R.; Tunik, S. P. *RSC Adv.* **2015**, *5,* 94551–94561. doi:10.1039/C5RA17755C

5. Porras, J. A.; Mills, I. N.; Transue, Wesley J.; Bernhard, S. *J. Am. Chem. Soc.* **2016**, *138*, 9460–9472. doi:10.1021/jacs.6b03246
6. He, X.; Li, R.; Choy, P. Y.; Liu, T.; Wang, J.; Yuen, O. Y.; Leung, M. P.; Shang, Y.; Kwong, F. Y. *Org. Lett*. **2020**, *22*, 9444–9449. doi:10.1021/acs.orglett.0c03374
7. Ferlin, M. G.; Chiarelotto, G.; Malesani, G. *J. Heterocyc. Chem.* **1989**, *26*, 245–249. doi:10.1002/jhet.5570260144
8. Hu, R.-B.; Sun, S.; Su, Y. *Angew. Chem. Int. Ed.* **2017**, *56*, 10877–10880. doi:10.1002/anie.201704385
9. Szafran, M.; Szwajca, A. *Polish J. Chem.*; **2004**, *78*, 2131–2140.
10. Szwajca, A.; Katrusiak, A.; Szafran, M. *J. Mol. Str.* **2004**, 705, 159–165. doi:10.1016/j.molstruc.2004.06.015
11. Castillo, R. R.; Burgos, C.; Vaquero, J. J.; Alvarez-Builla, J. *Eur. J. Org. Chem.*, **2011**, 619–628. doi.org:10.1002/ejoc.201001071

^{1}H NMR (400 MHz, DMSO-d_6) of 1-(1-benzyl-4-(2-bromophenyl)-2-phenyl-1*H*-pyrrol-3-yl)pyridin-1-ium bromide (N-Bn-**1a***)*

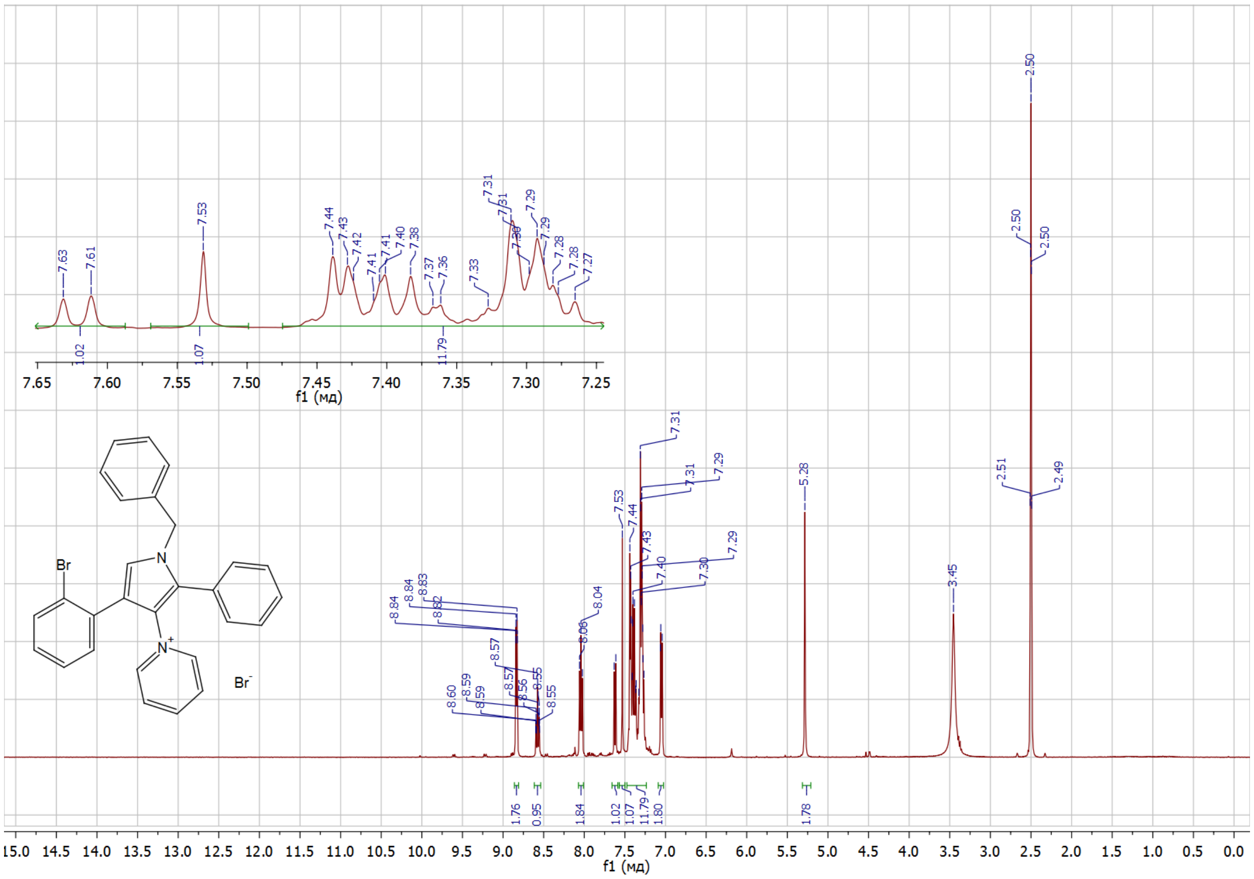

^{13}C{1H} NMR (101 MHz, DMSO-d_6) of 1-(1-benzyl-4-(2-bromophenyl)-2-phenyl-1*H*-pyrrol-3-yl)pyridin-1-ium bromide *(*N-Bn-**1a**)

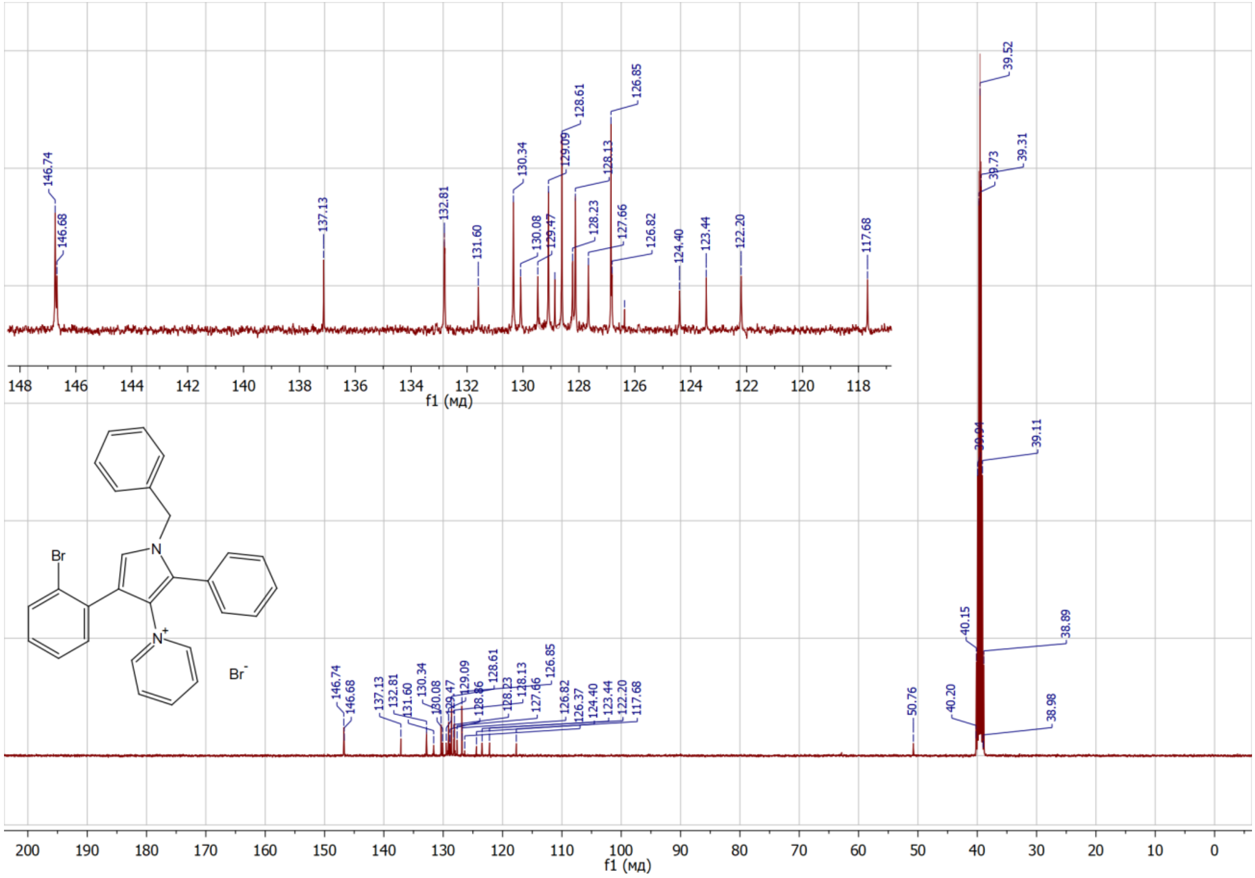

^{13}C DEPT135 NMR (101 MHz, DMSO-d_6) of 1-(1-benzyl-4-(2-bromophenyl)-2-phenyl-1*H*-pyrrol-3-yl)pyridin-1-ium bromide (N-Bn-**1a**)

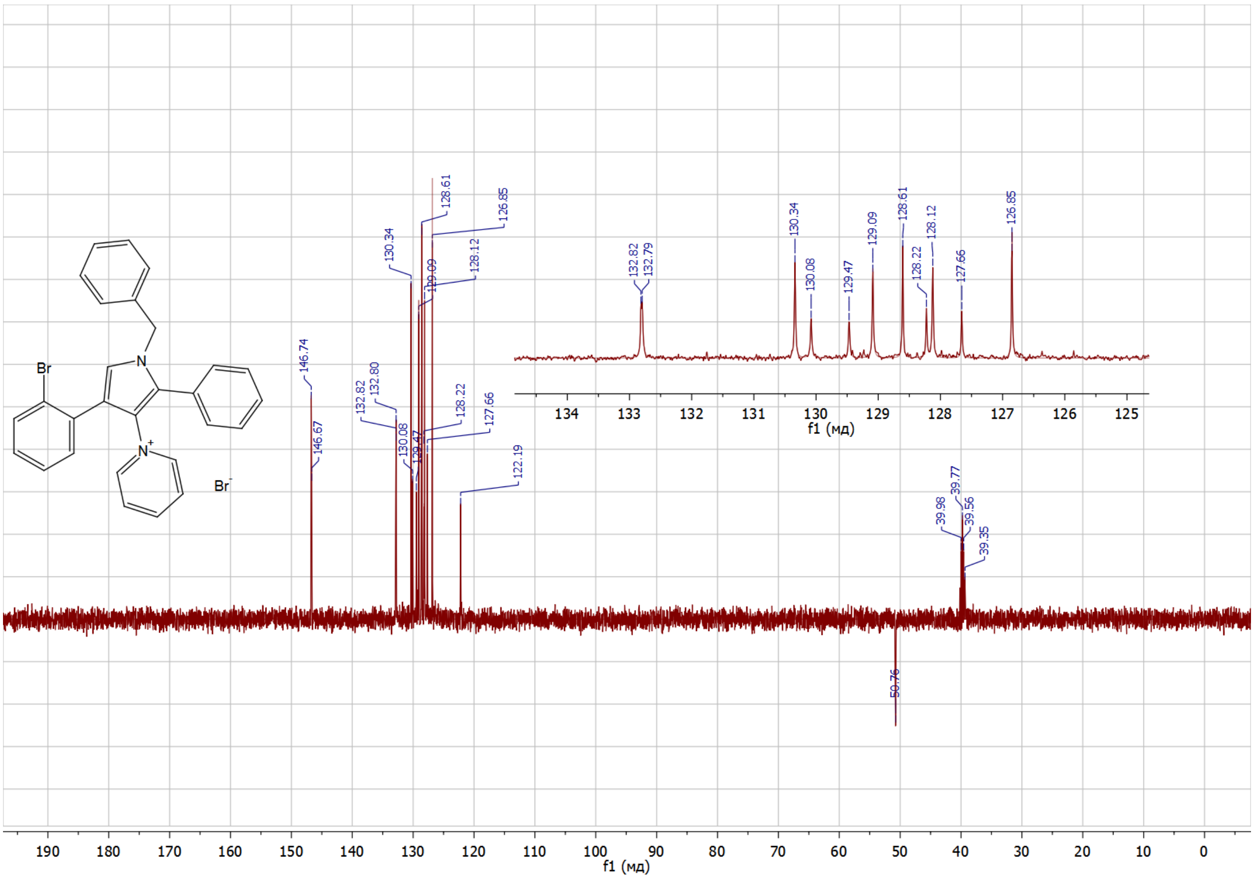

^{1}H NMR (400 MHz, DMSO-d_6) of 1-(1-acetyl-4-(2-bromophenyl)-2-phenyl-1*H*-pyrrol-3-yl)pyridin-1-ium chloride (N-Ac-**1a**)

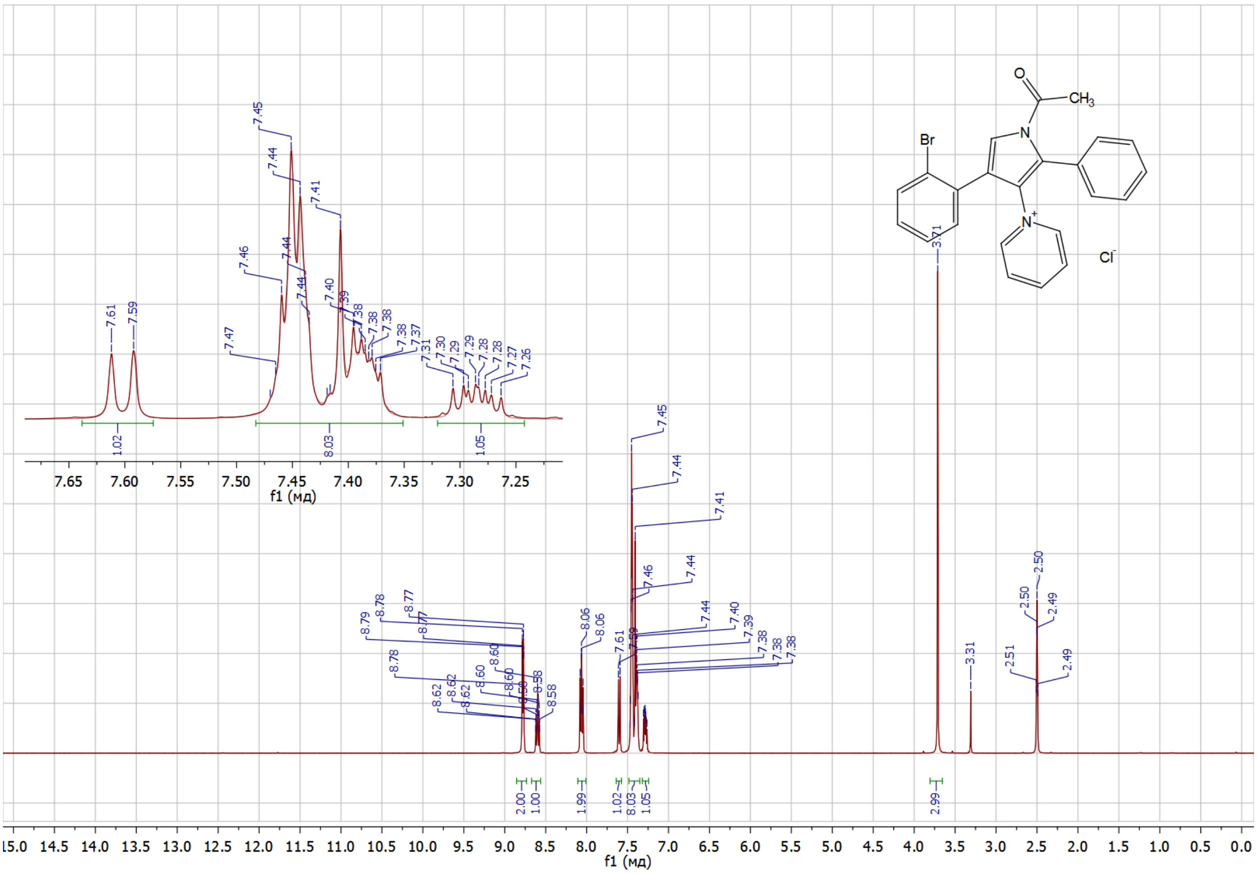

$^{13}C\{^{1}H\}$ NMR (101 MHz, DMSO-d_6) of 1-(1-acetyl-4-(2-bromophenyl)-2-phenyl-1*H*-pyrrol-3-yl)pyridin-1-ium chloride (N-Ac-**1a**)

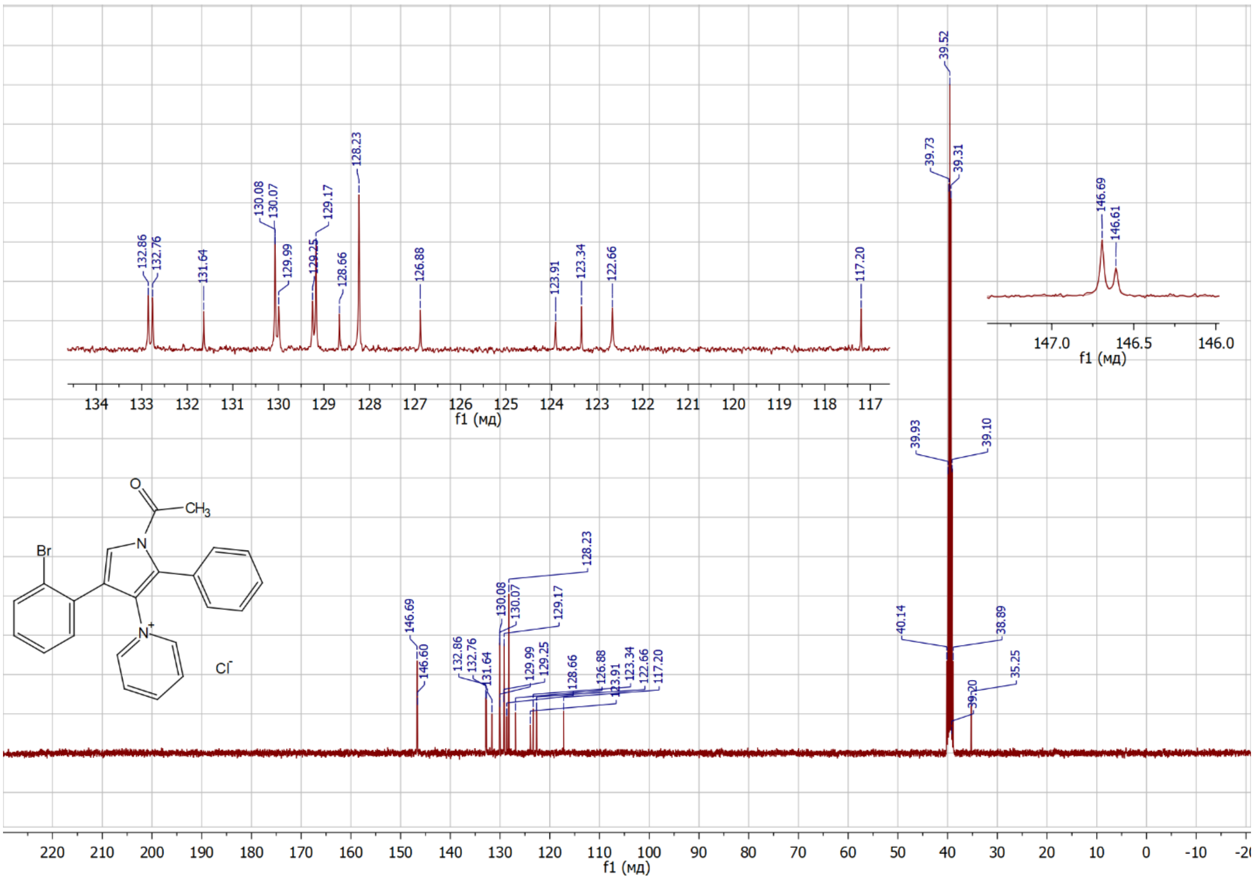

^{13}C DEPT135 NMR (101 MHz, DMSO-d_6) of 1-(1-acetyl-4-(2-bromophenyl)-2-phenyl-1*H*-pyrrol-3-yl)pyridin-1-ium chloride (N-Ac-**1a**)

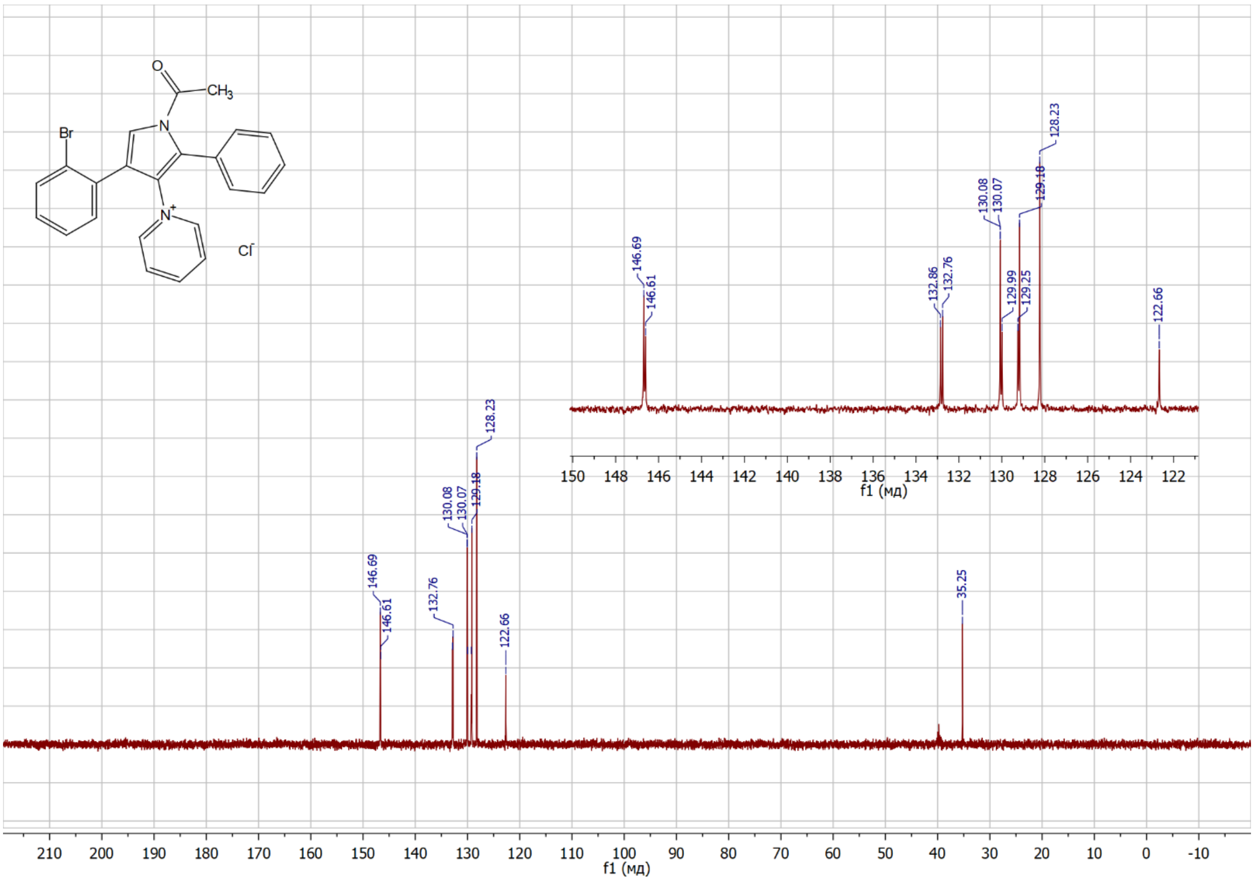

^{1}H NMR (400 MHz, DMSO-d_6) of 1-(4-(2-bromophenyl)-2-(4-fluorophenyl)-1*H*-pyrrol-3-yl)pyridin-1-ium bromide (**1b**)

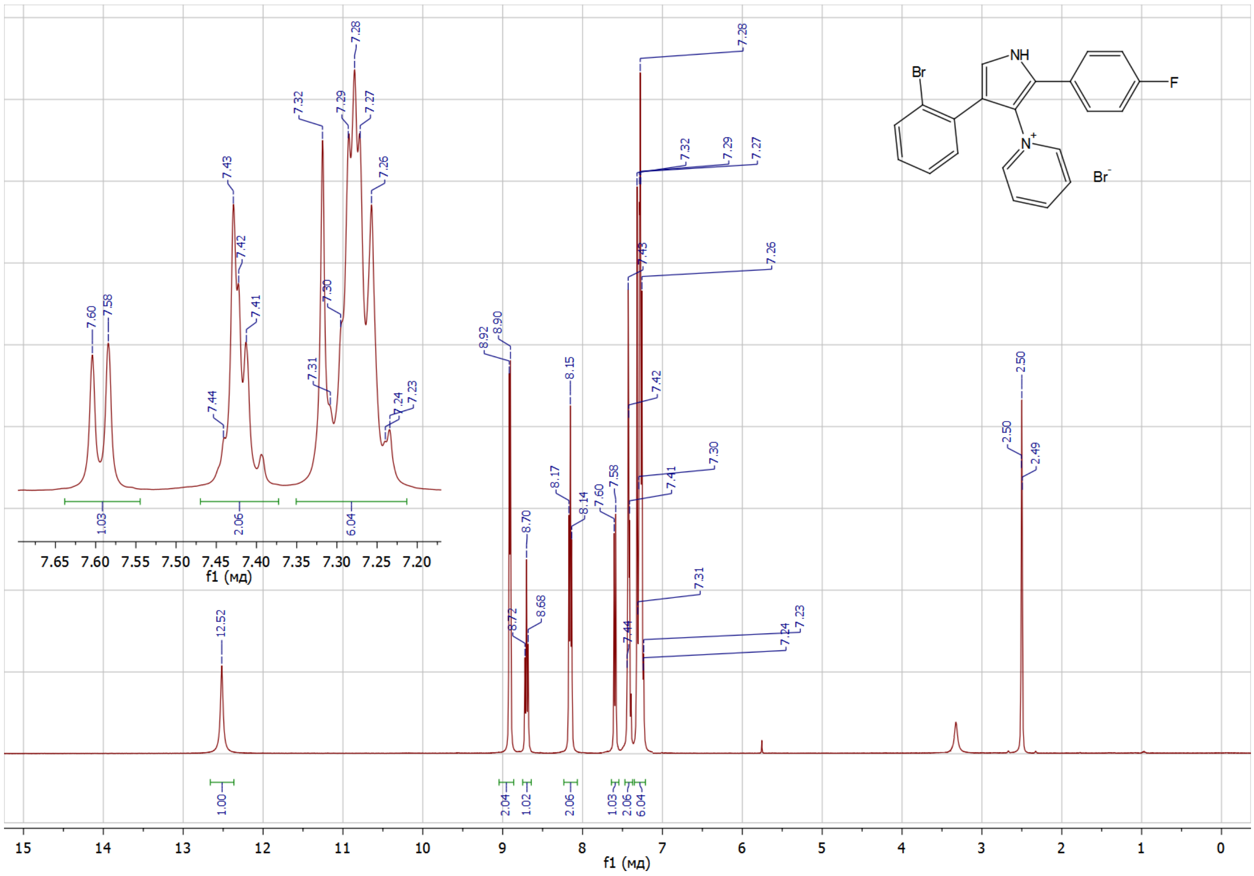

^{13}C\{1H\} NMR (101 MHz, DMSO-d_6) of 1-(4-(2-bromophenyl)-2-(4-fluorophenyl)-1*H*-pyrrol-3-yl)pyridin-1-ium bromide (**1b**)

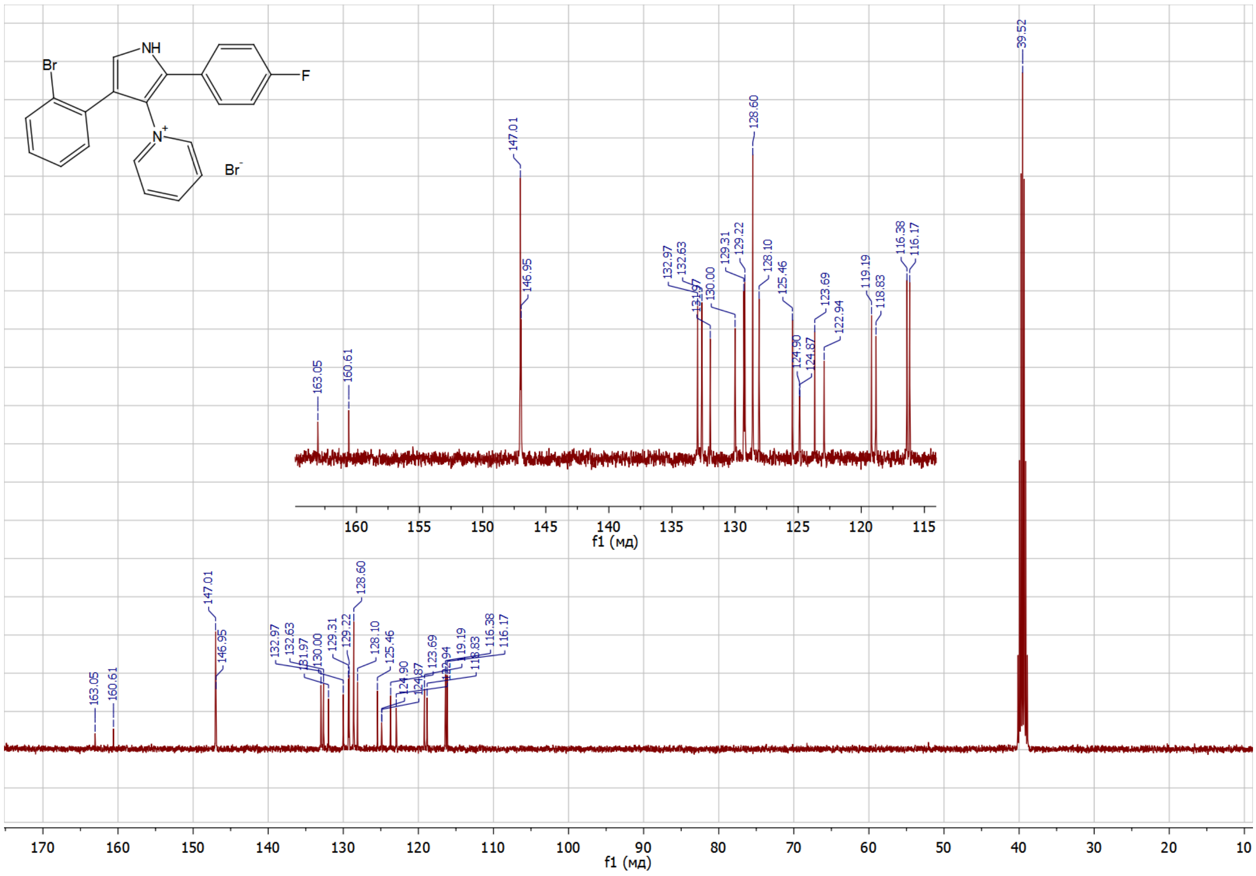

^{13}C DEPT135 NMR (101 MHz, DMSO-d_6) of 1-(4-(2-bromophenyl)-2-(4-fluorophenyl)-1*H*-pyrrol-3-yl)pyridin-1-ium bromide (**1b**)

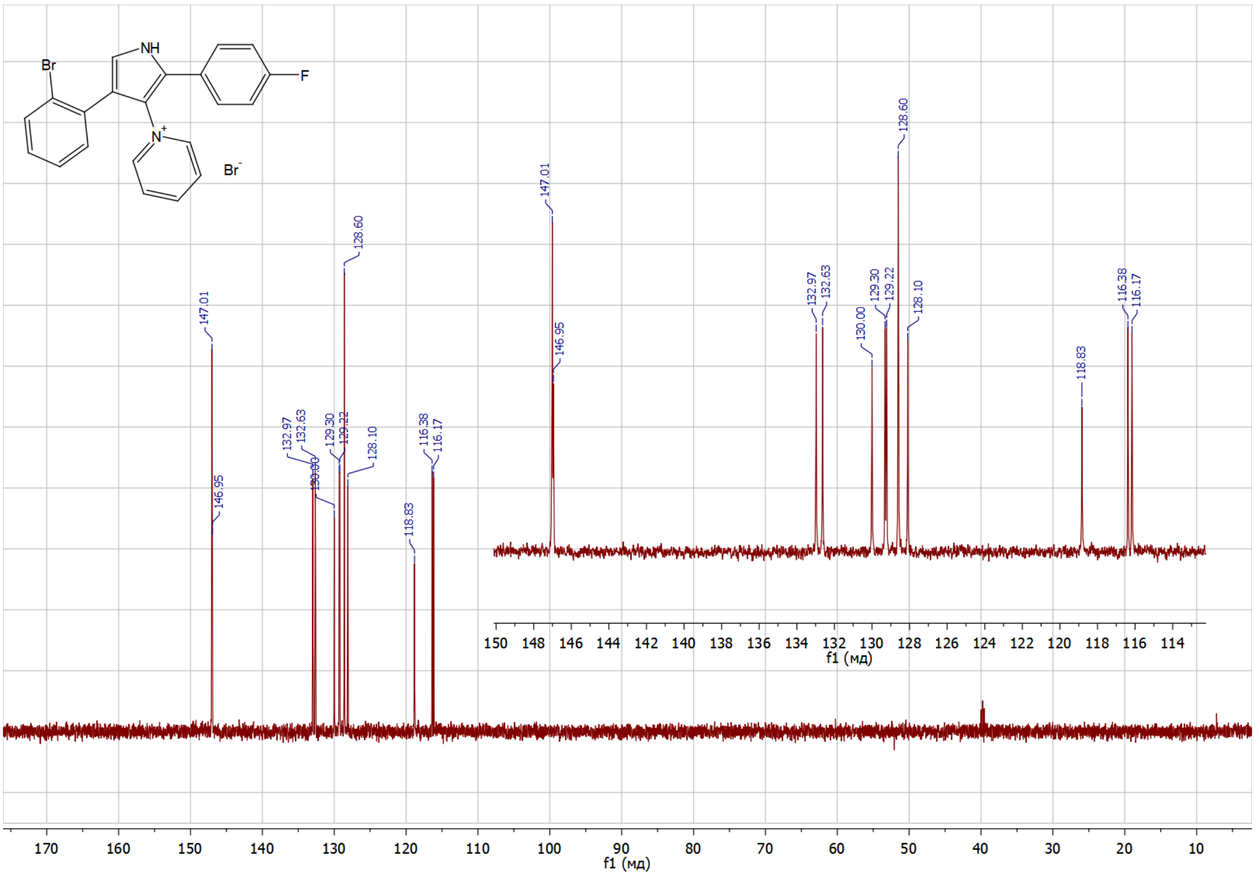

^{1}H NMR (400 MHz, DMSO-d_6) of 1-(4-(2-bromophenyl)-2-(4-chlorophenyl)-1*H*-pyrrol-3-yl)pyridin-1-ium bromide (**1c**)

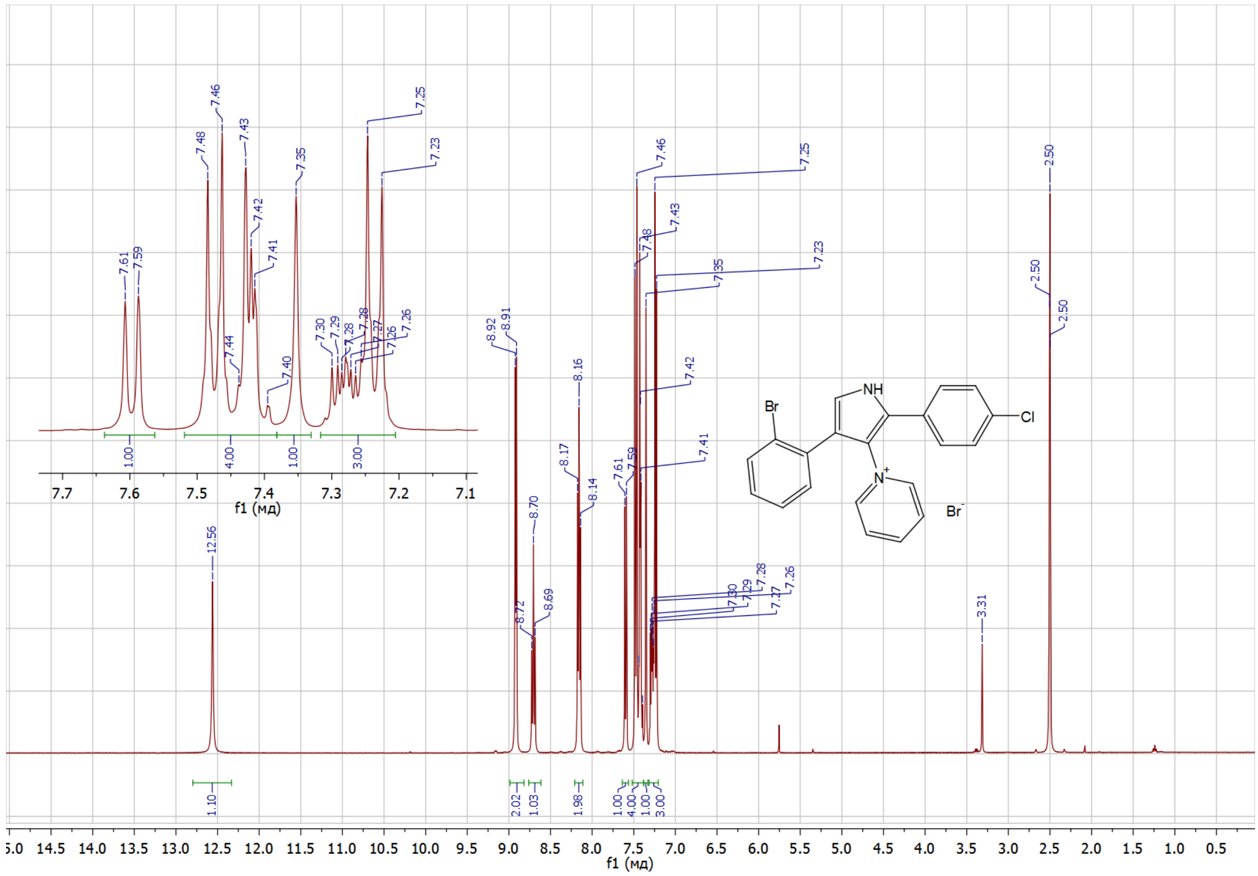

$^{13}C\{1H\}$ NMR (101 MHz, DMSO-d_6) of 1-(4-(2-bromophenyl)-2-(4-chlorophenyl)-1*H*-pyrrol-3-yl)pyridin-1-ium bromide (**1c**)

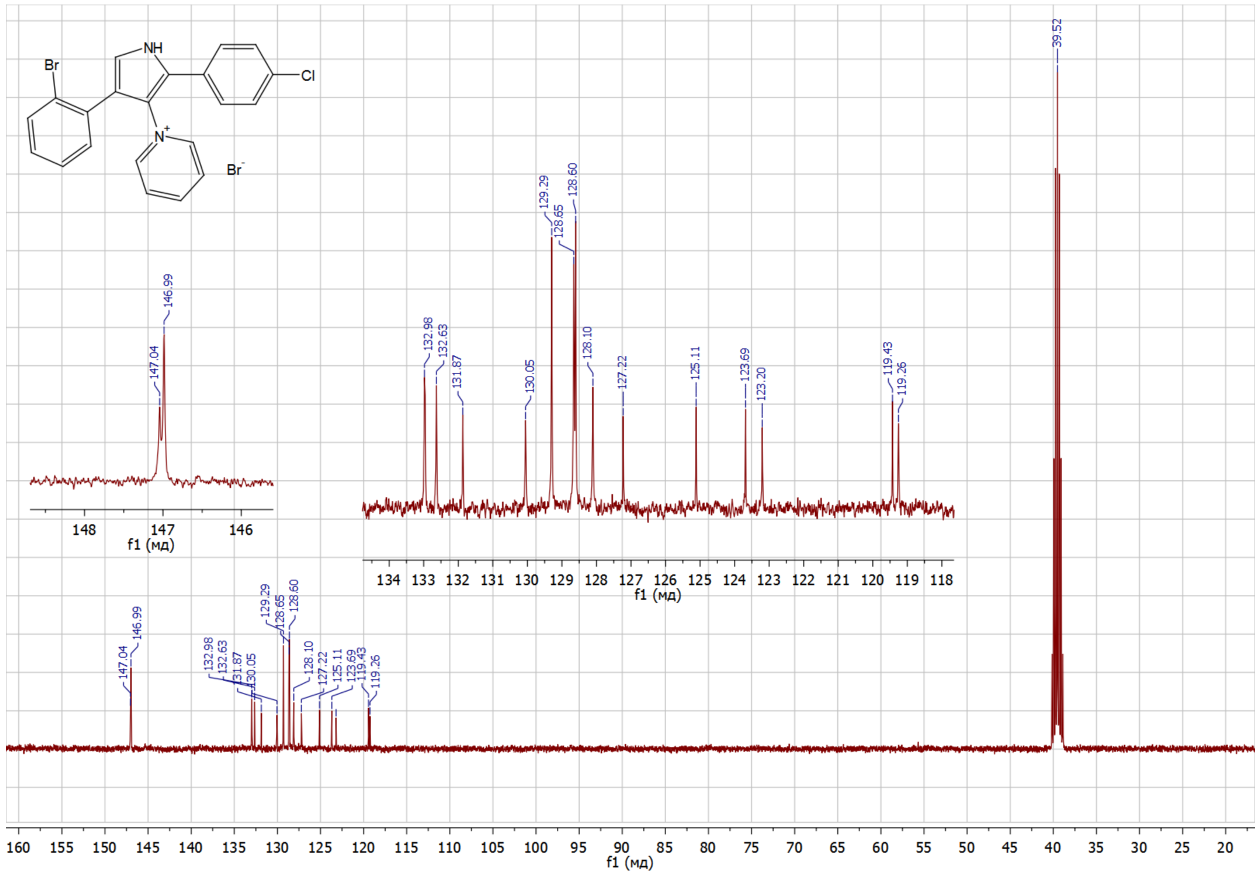

^{13}C DEPT135 NMR (101 MHz, DMSO-d_6) of 1-(4-(2-bromophenyl)-2-(4-chlorophenyl)-1*H*-pyrrol-3-yl)pyridin-1-ium bromide (**1c**)

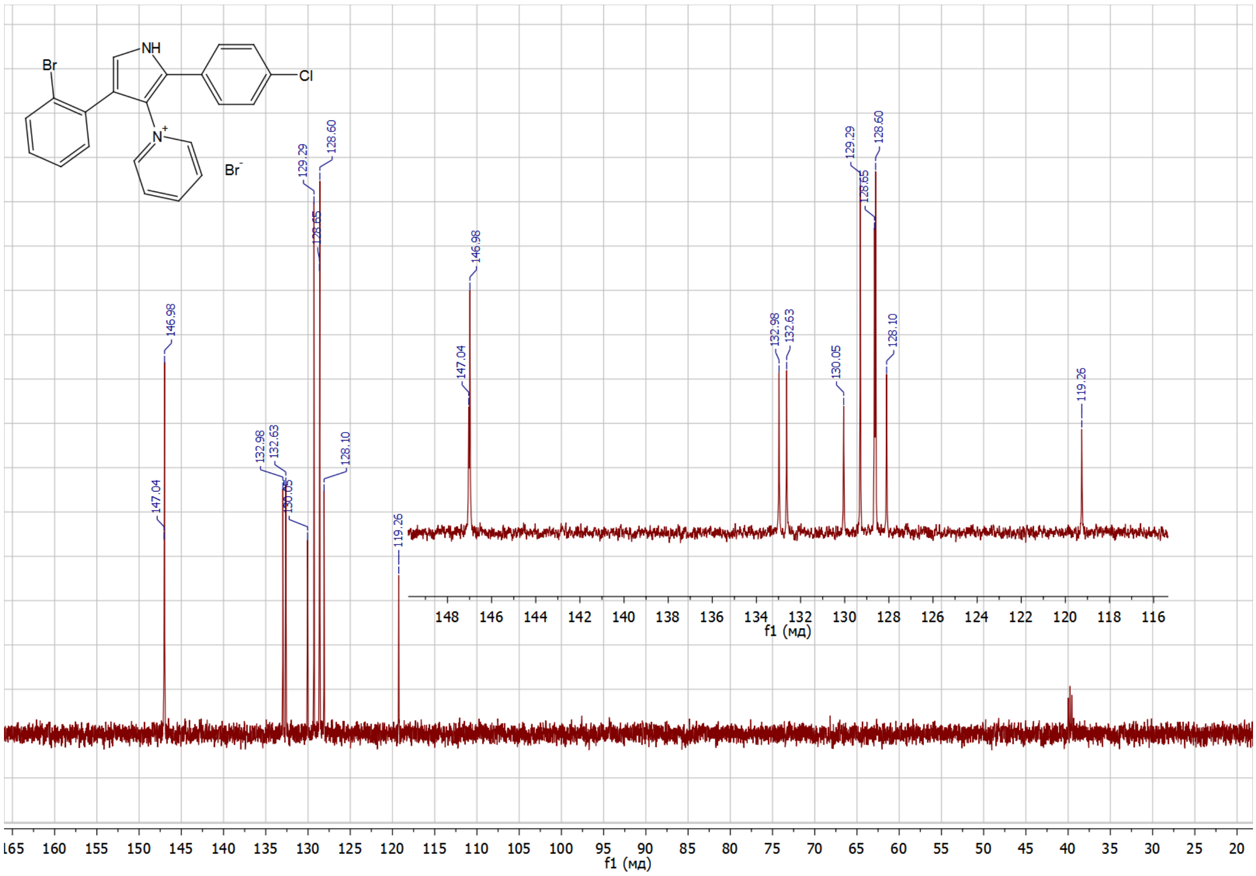

^{1}H NMR (400 MHz, DMSO-d_6) of 1-(4-(2-bromophenyl)-2-(4-bromophenyl)-1*H*-pyrrol-3-yl)pyridin-1-ium bromide (**1d**)

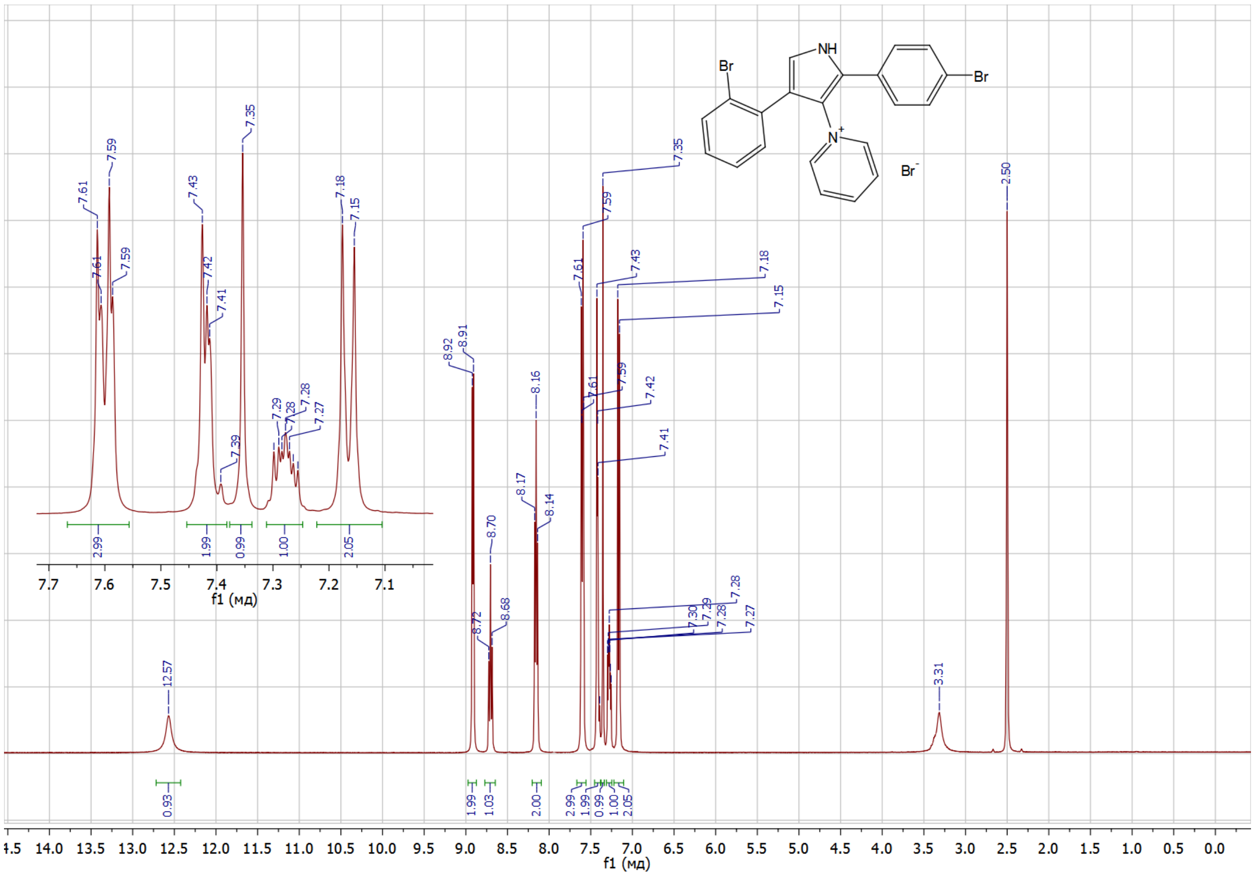

$^{13}C\{^{1}H\}$ NMR (101 MHz, DMSO-d_6) of 1-(4-(2-bromophenyl)-2-(4-bromophenyl)-1*H*-pyrrol-3-yl)pyridin-1-ium bromide (**1d**)

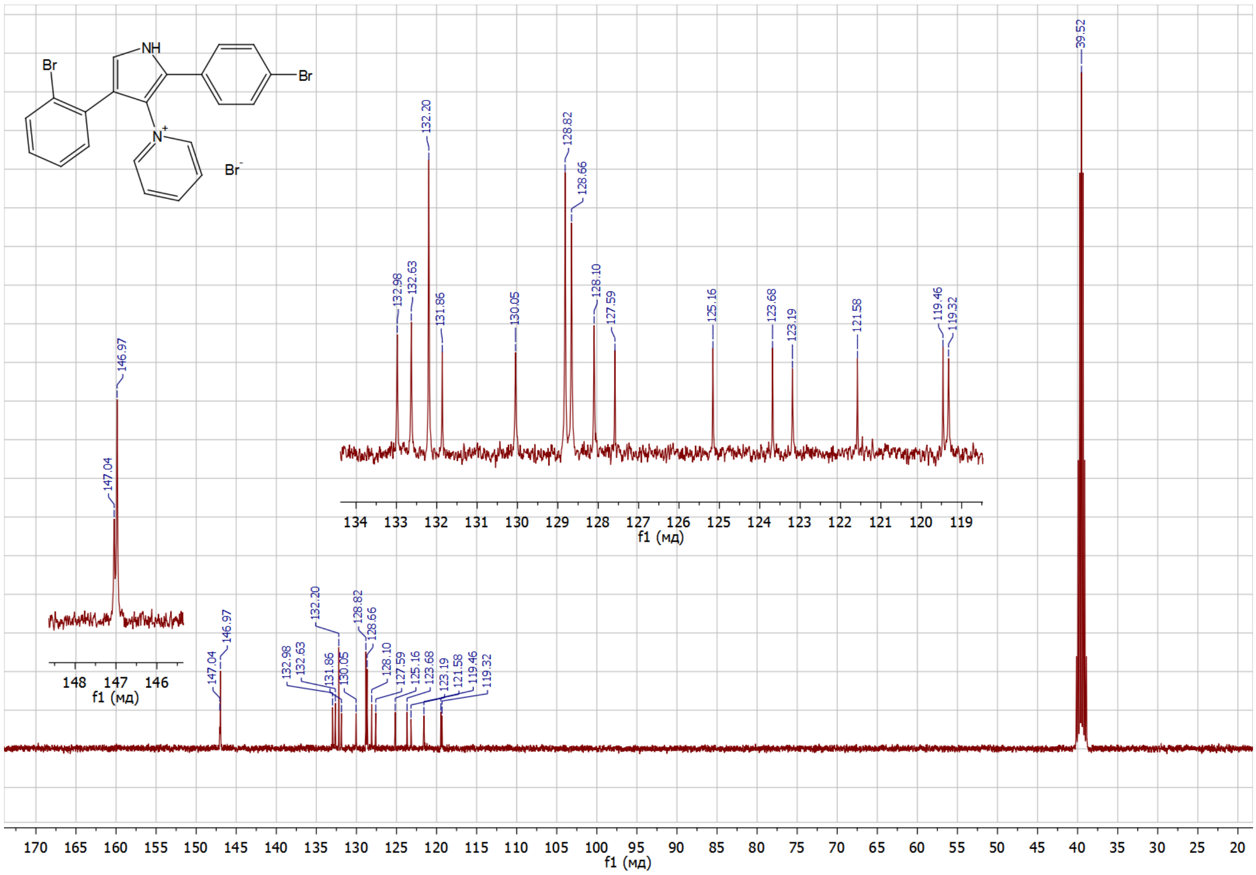

^{13}C DEPT135 NMR (101 MHz, DMSO-d_6) of 1-(4-(2-bromophenyl)-2-(4-bromophenyl)-1*H*-pyrrol-3-yl)pyridin-1-ium bromide (**1d**)

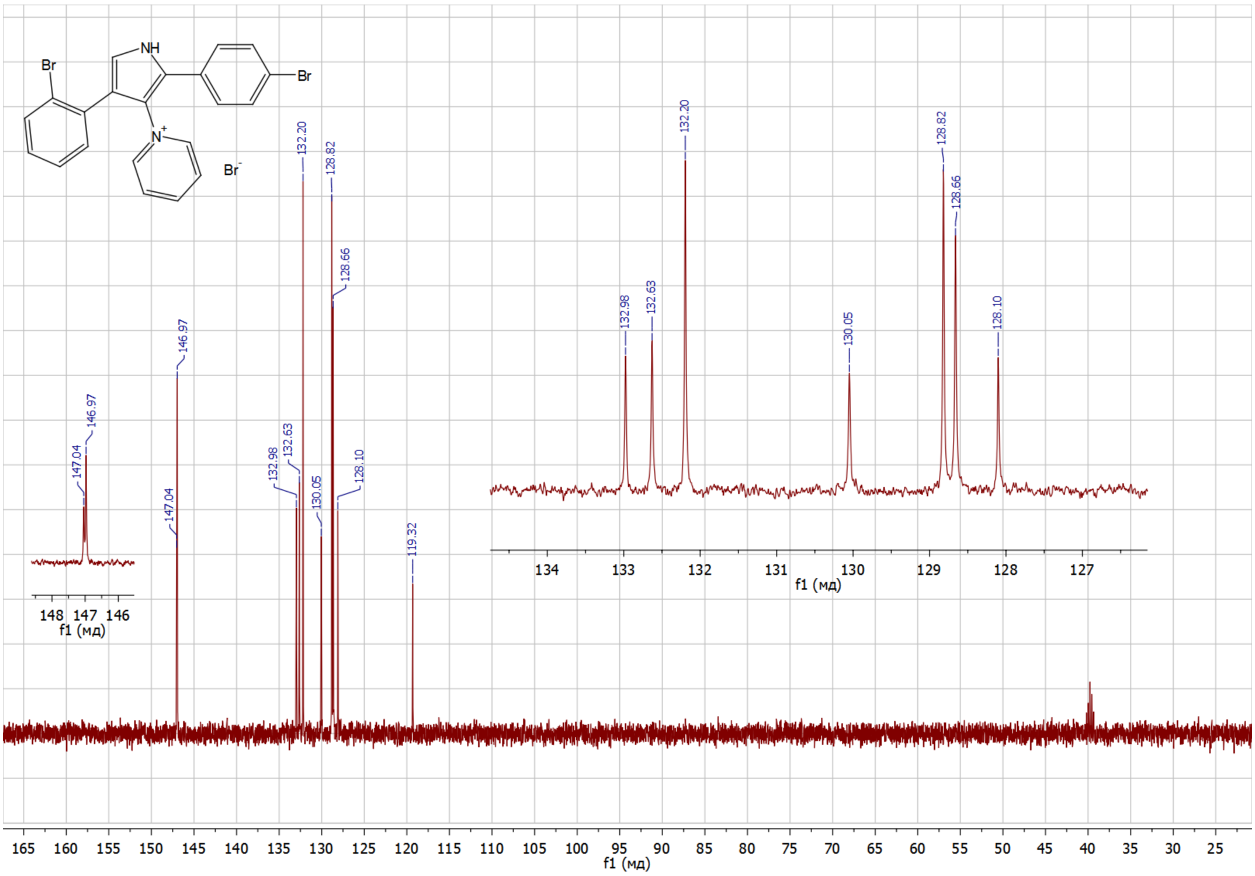

^{1}H NMR (400 MHz, DMSO-d_6) of 1-(4-(2-bromophenyl)-2-(4-methylphenyl)-1*H*-pyrrol-3-yl)pyridin-1-ium bromide (**1e**)

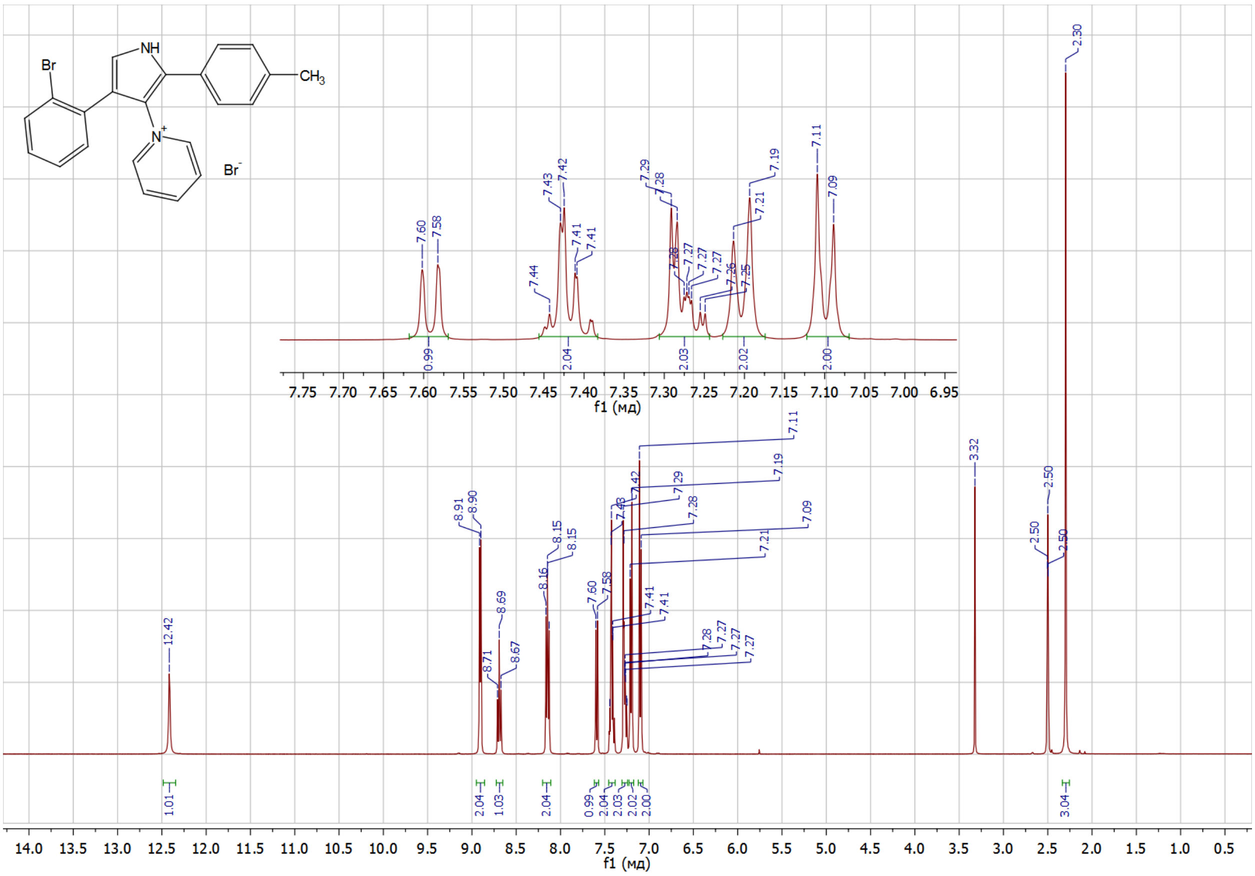

^{13}C{1H} NMR (101 MHz, DMSO-d_6) of 1-(4-(2-bromophenyl)-2-(4-methylphenyl)-1*H*-pyrrol-3-yl)pyridin-1-ium bromide (**1e**)

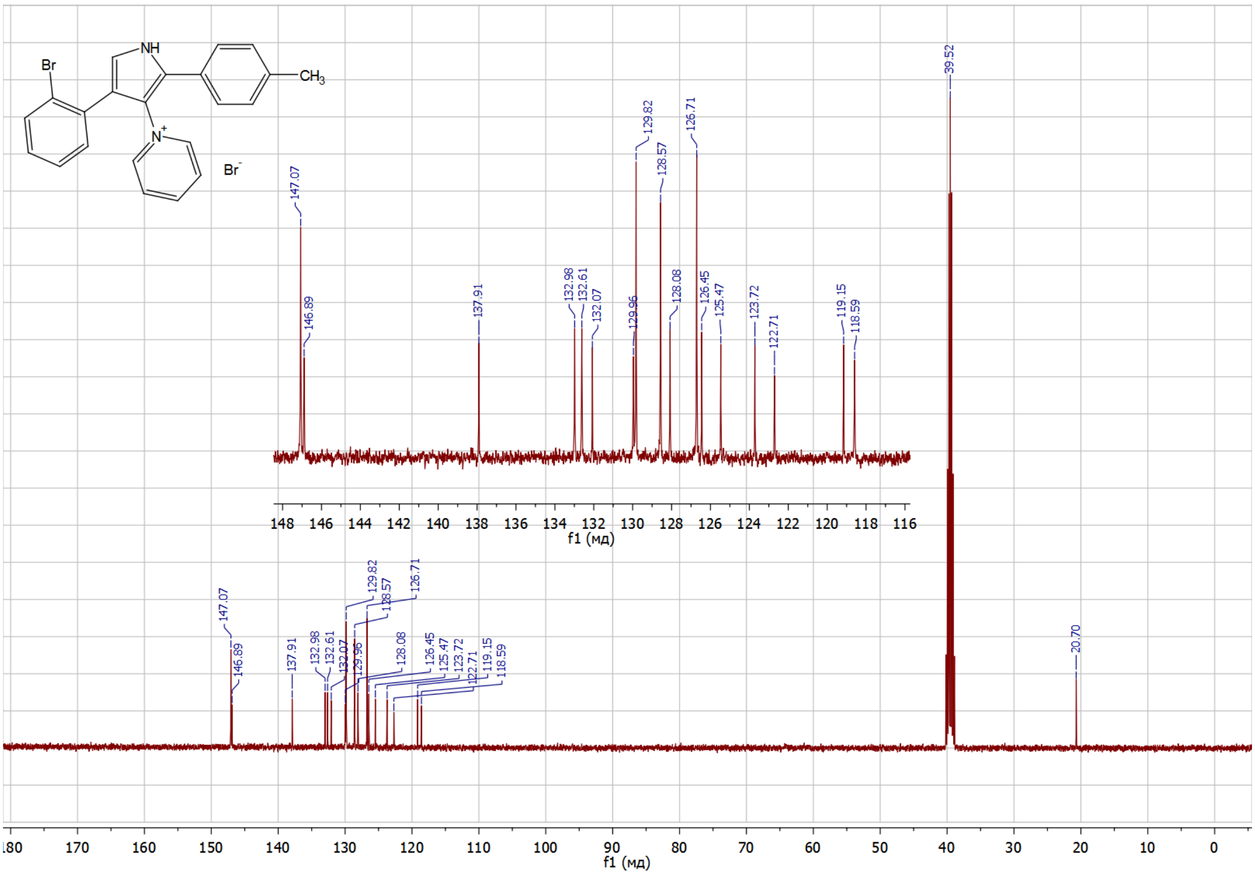

^{13}C DEPT135 NMR (101 MHz, DMSO-d_6) of 1-(4-(2-bromophenyl)-2-(4-methylphenyl)-1*H*-pyrrol-3-yl)pyridin-1-ium bromide (**1e**)

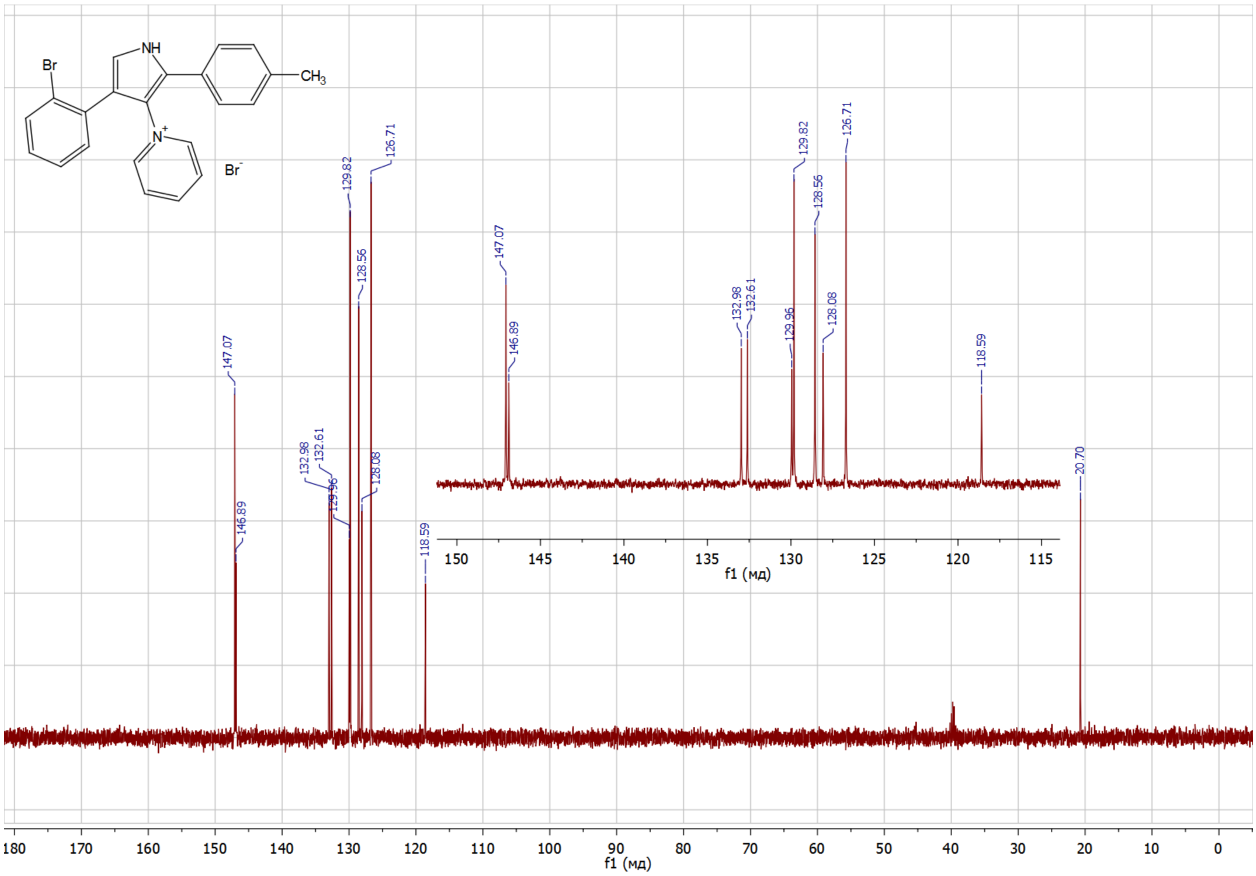

^{1}H NMR (400 MHz, DMSO-d_6) of 1-(4-(2-bromophenyl)-2-(4-methoxyphenyl)-1*H*-pyrrol-3-yl)pyridin-1-ium bromide (**1f**)

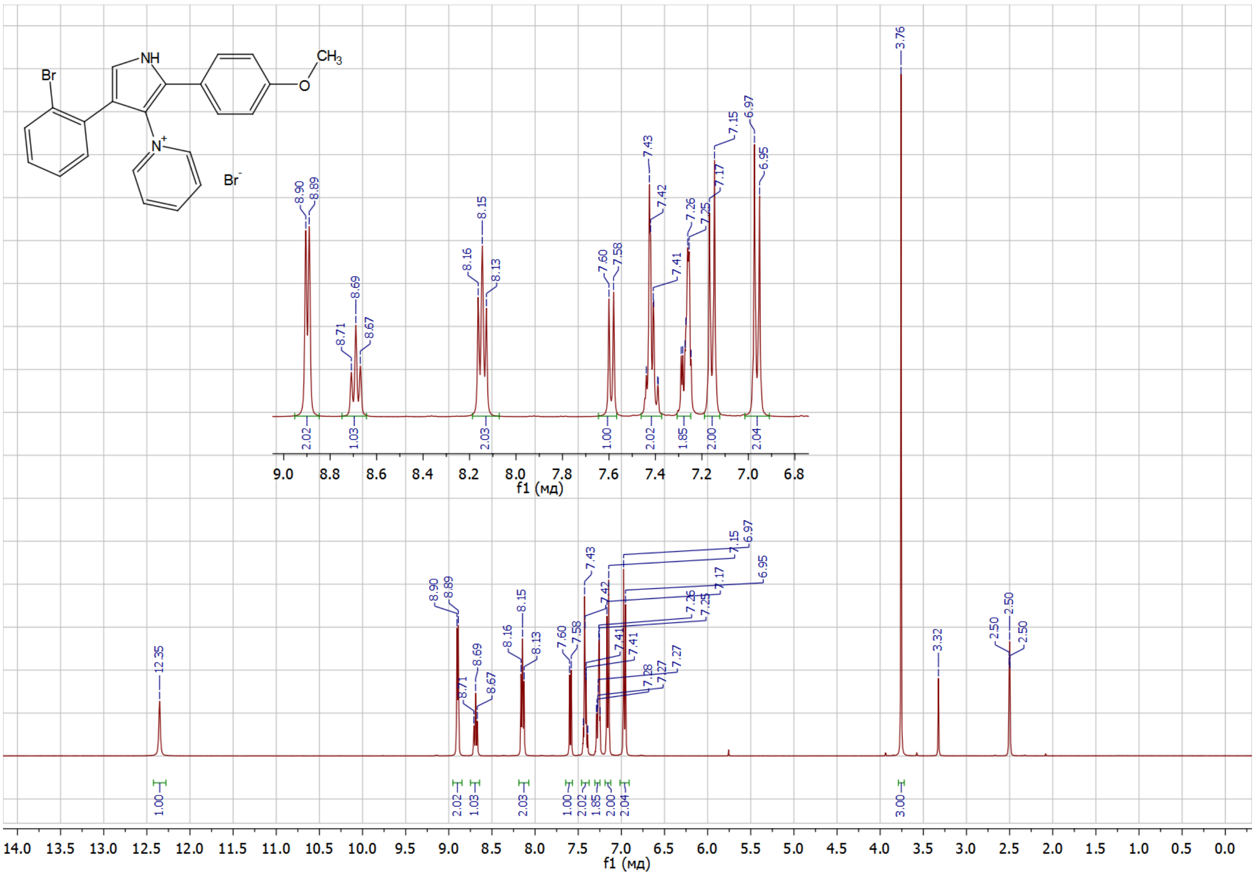

$^{13}C\{1H\}$ NMR (101 MHz, DMSO-d_6) of 1-(4-(2-bromophenyl)-2-(4-methoxyphenyl)-1*H*-pyrrol-3-yl)pyridin-1-ium bromide (**1f**)

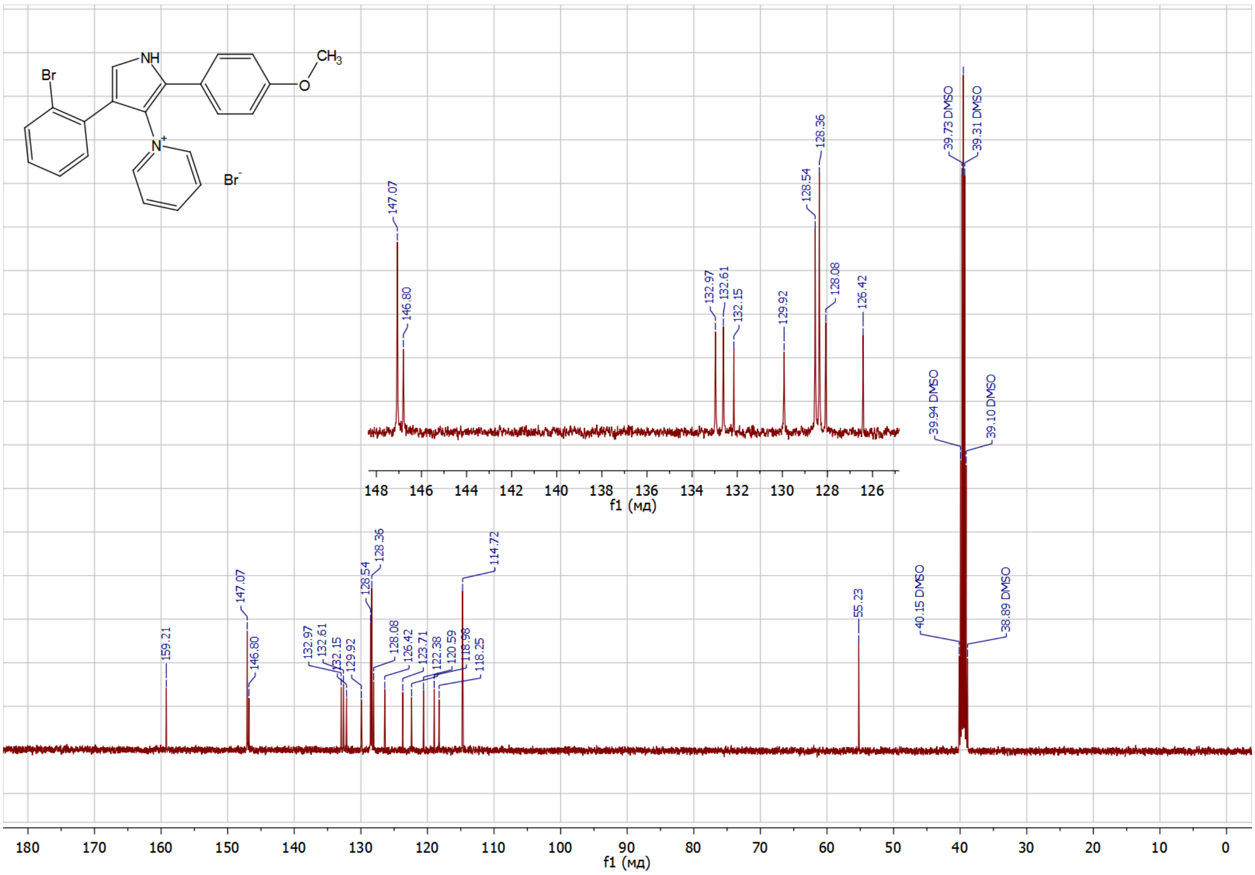

^{13}C DEPT135 NMR (101 MHz, DMSO-d_6) of 1-(4-(2-bromophenyl)-2-(4-methoxyphenyl)-1*H*-pyrrol-3-yl)pyridin-1-ium bromide (**1f**)

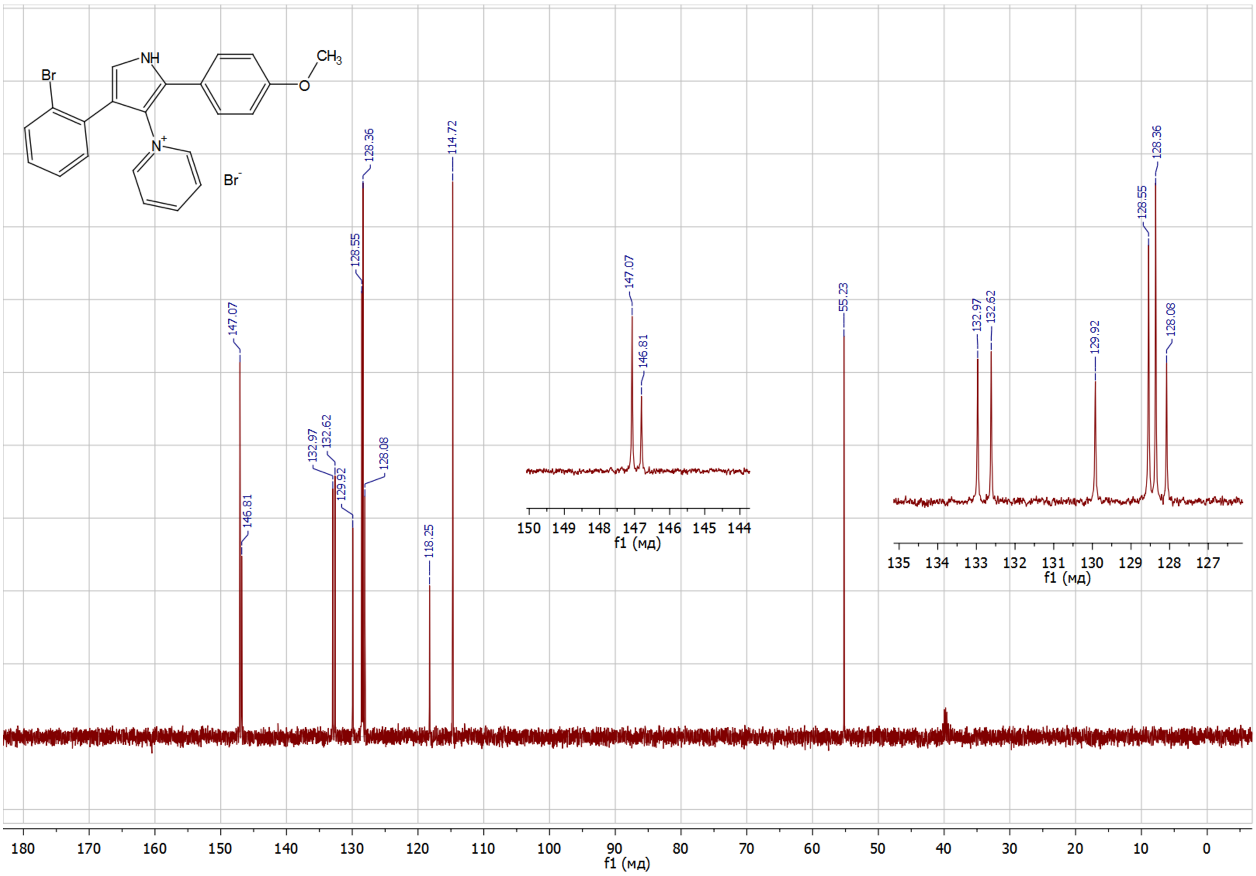

^{1}H NMR (400 MHz, DMSO-d_6) of 1-(4-(2-bromophenyl)-2-(2-methoxyphenyl)-1*H*-pyrrol-3-yl)pyridin-1-ium bromide (**1g**)

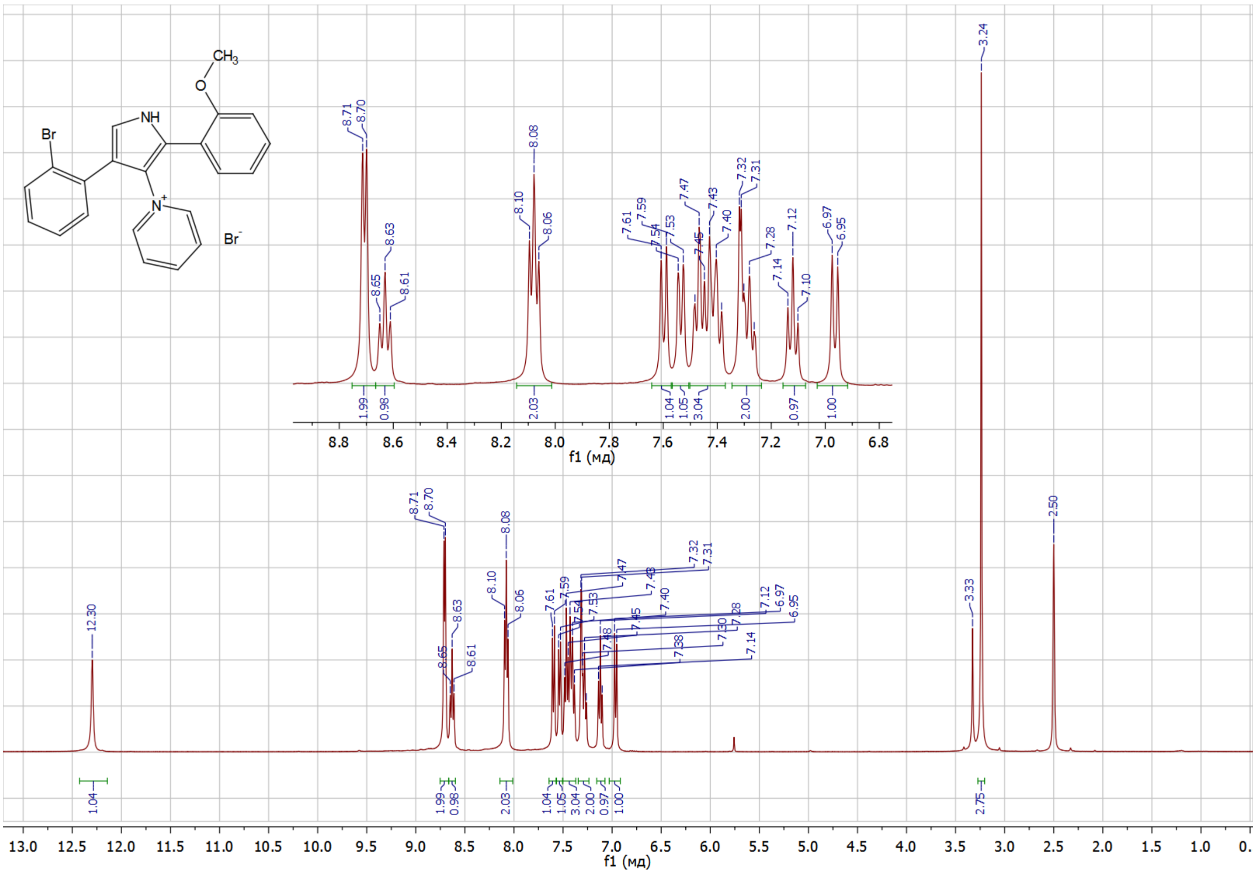

^{13}C{1H} NMR (101 MHz, DMSO-d_6) of 1-(4-(2-bromophenyl)-2-(2-methoxyphenyl)-1*H*-pyrrol-3-yl)pyridin-1-ium bromide (**1g**)

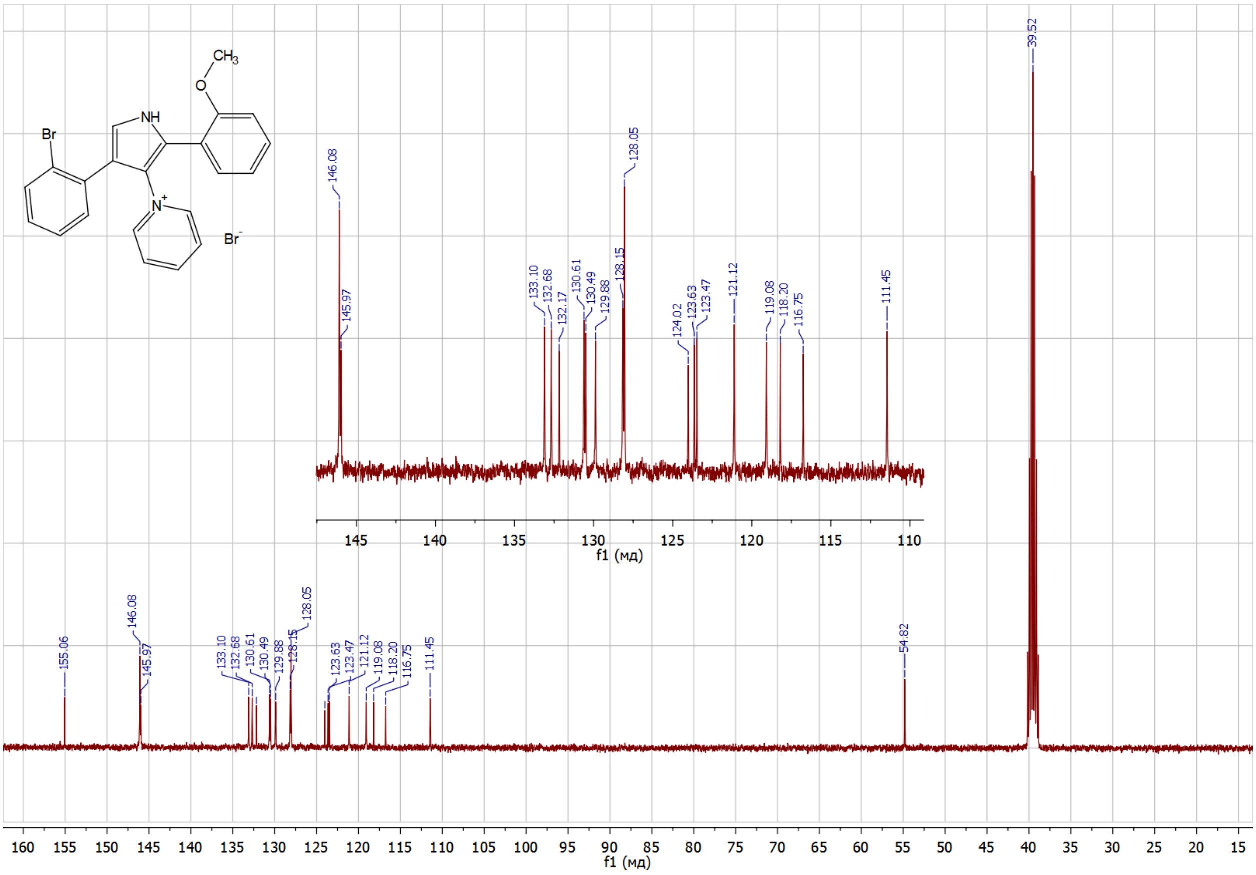

^{13}C DEPT135 NMR (101 MHz, DMSO-d_6) of 1-(4-(2-bromophenyl)-2-(2-methoxyphenyl)-1*H*-pyrrol-3-yl)pyridin-1-ium bromide (**1g**)

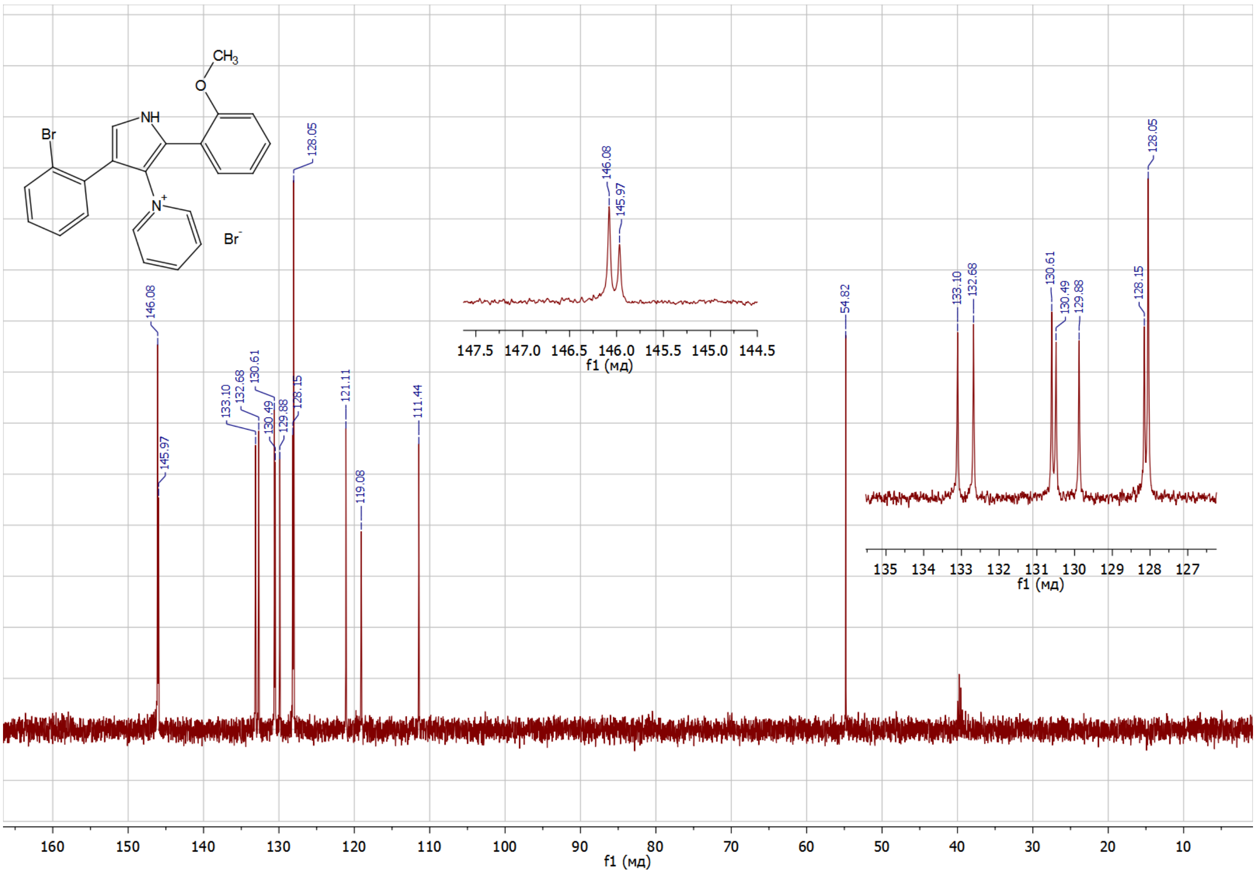

^{1}H NMR (400 MHz, DMSO-d_6) of 1-(4-(2-bromophenyl)-2-(3-methoxyphenyl)-1*H*-pyrrol-3-yl)pyridin-1-ium bromide (**1h**)

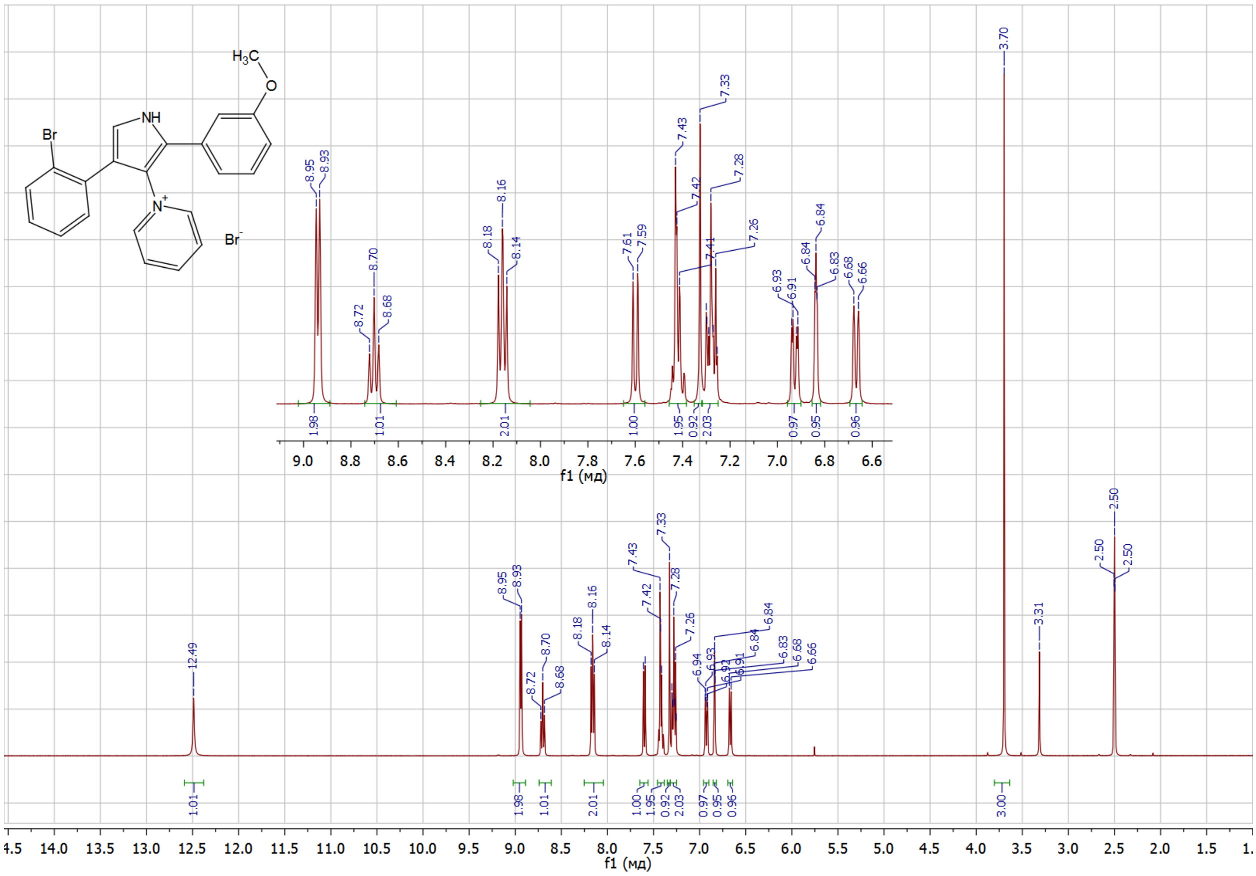

^{13}C{1H} NMR (101 MHz, DMSO-d_6) of 1-(4-(2-bromophenyl)-2-(3-methoxyphenyl)-1*H*-pyrrol-3-yl)pyridin-1-ium bromide (**1h**)

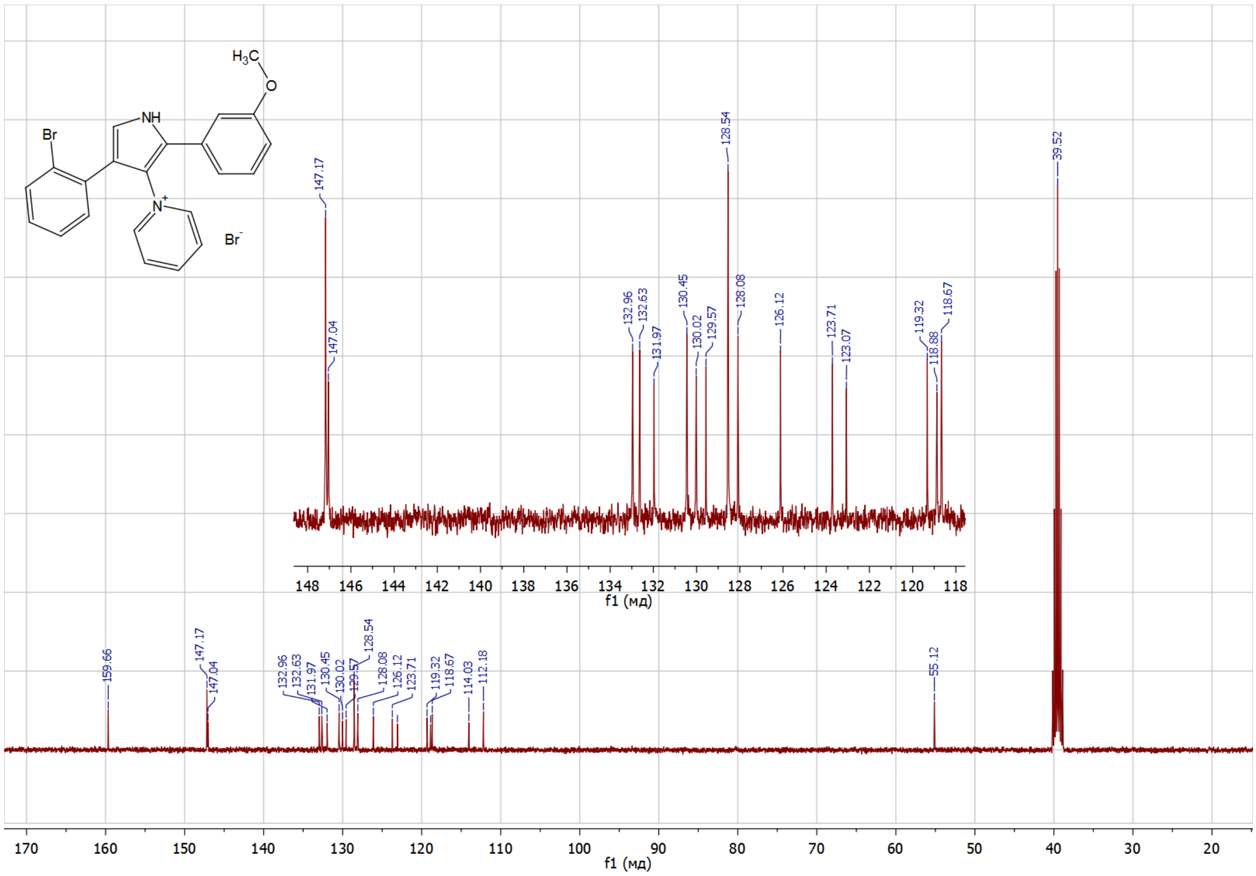

^{13}C DEPT135 NMR (101 MHz, DMSO-d_6) of 1-(4-(2-bromophenyl)-2-(3-methoxyphenyl)-1*H*-pyrrol-3-yl)pyridin-1-ium bromide (**1h**)

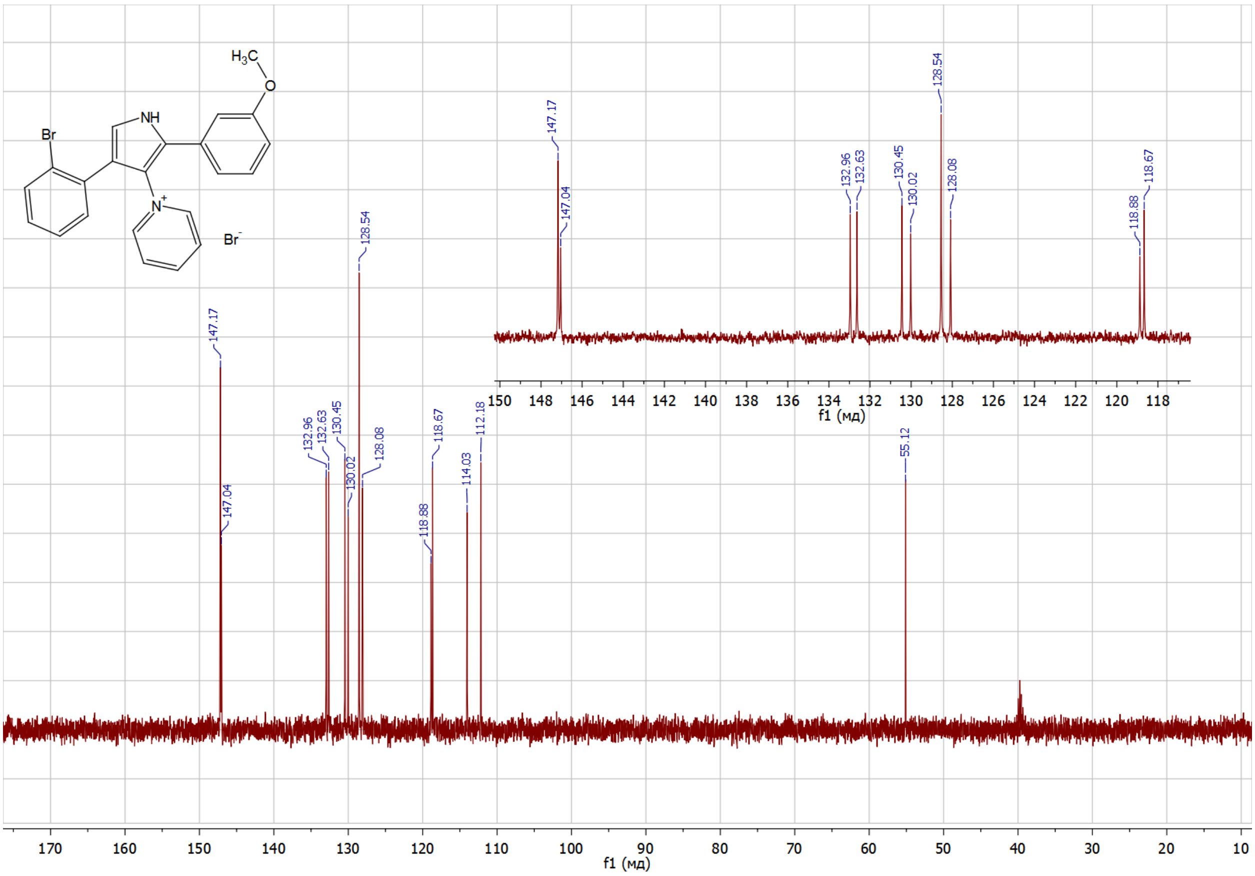

^{1}H NMR (400 MHz, DMSO-d_6) of 1-(4-(2-bromophenyl)-2-(2,4-dimethoxyphenyl)-1*H*-pyrrol-3-yl)pyridin-1-ium bromide (**1i**)

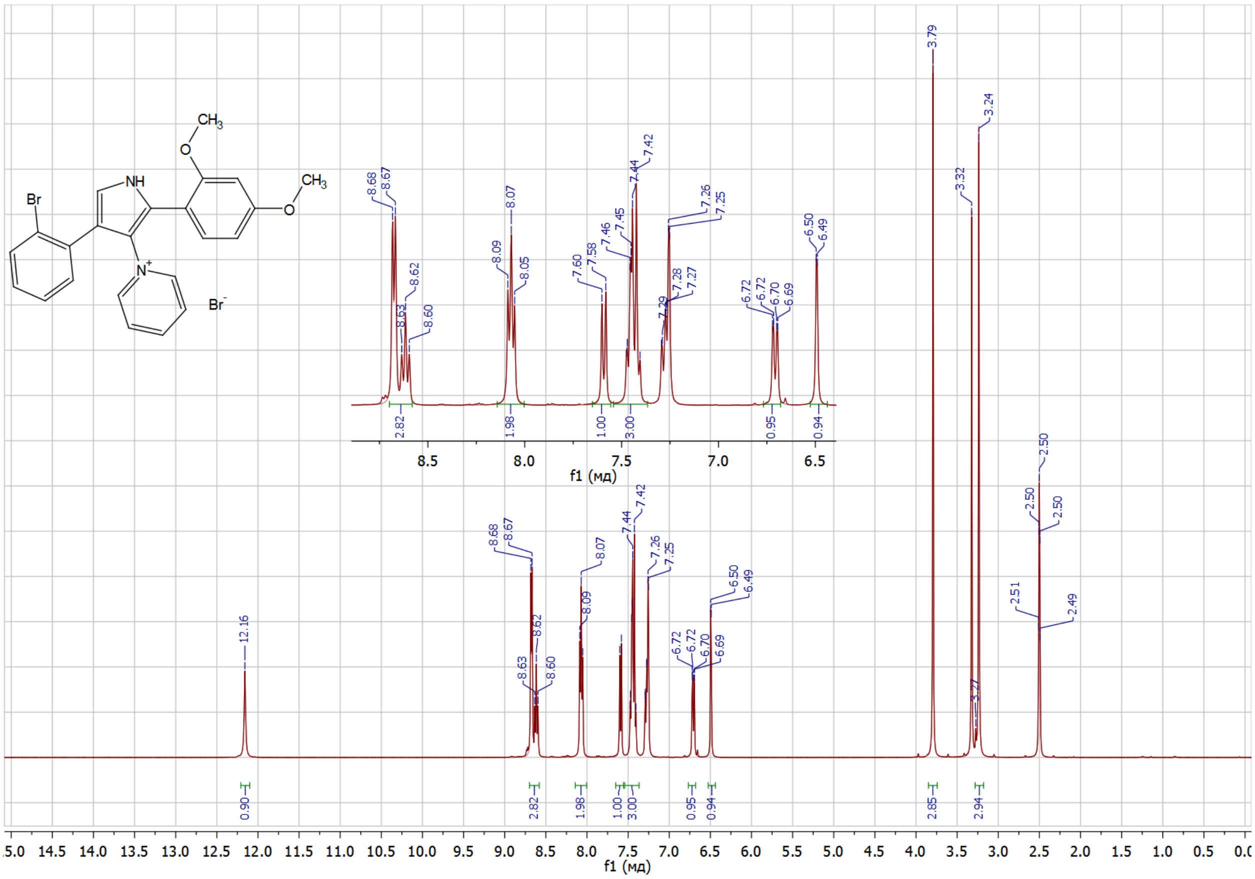

^{13}C{1H} NMR (101 MHz, DMSO-d_6) of 1-(4-(2-bromophenyl)-2-(2,4-dimethoxyphenyl)-1*H*-pyrrol-3-yl)pyridin-1-ium bromide (**1i**)

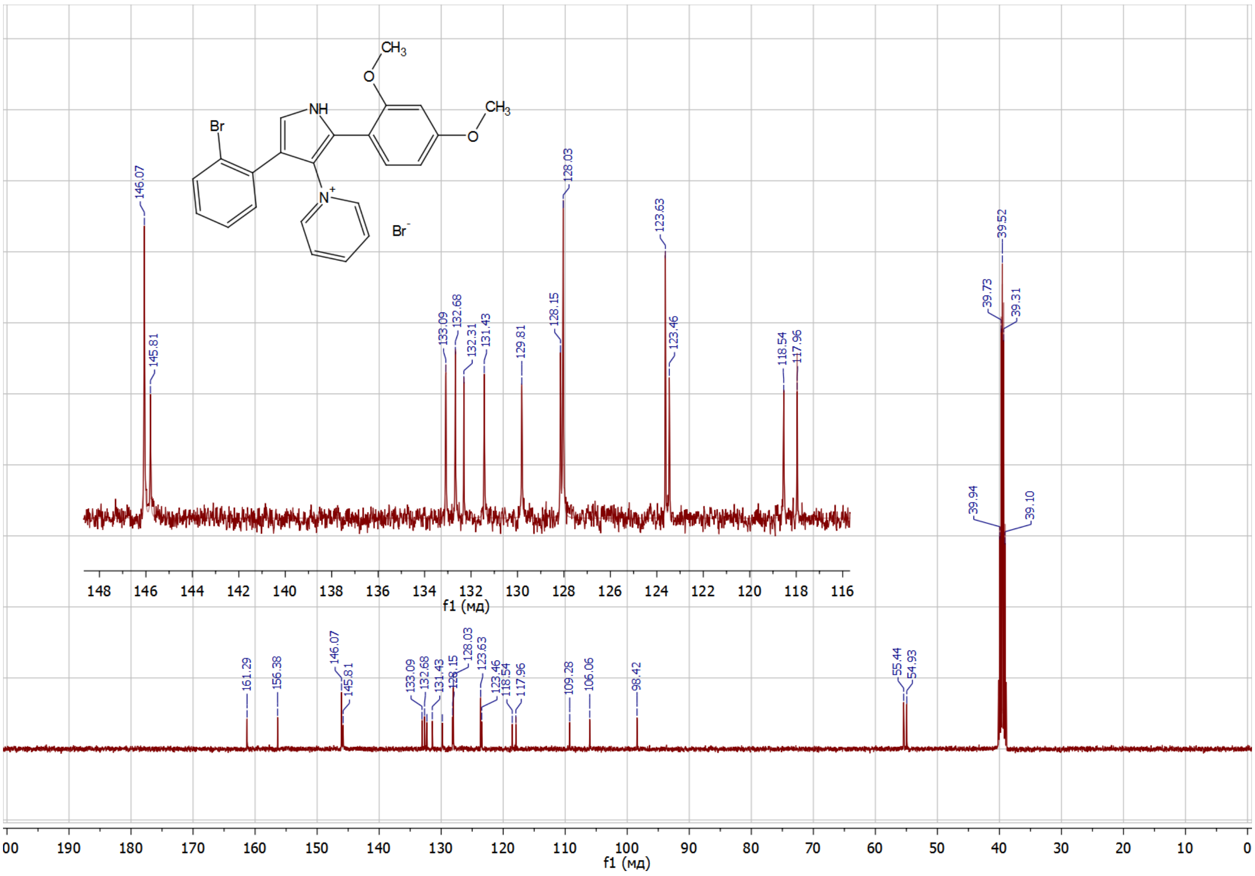

^{13}C DEPT135 NMR (101 MHz, DMSO-d_6) of 1-(4-(2-bromophenyl)-2-(2,4-dimethoxyphenyl)-1*H*-pyrrol-3-yl)pyridin-1-ium bromide (**1i**)

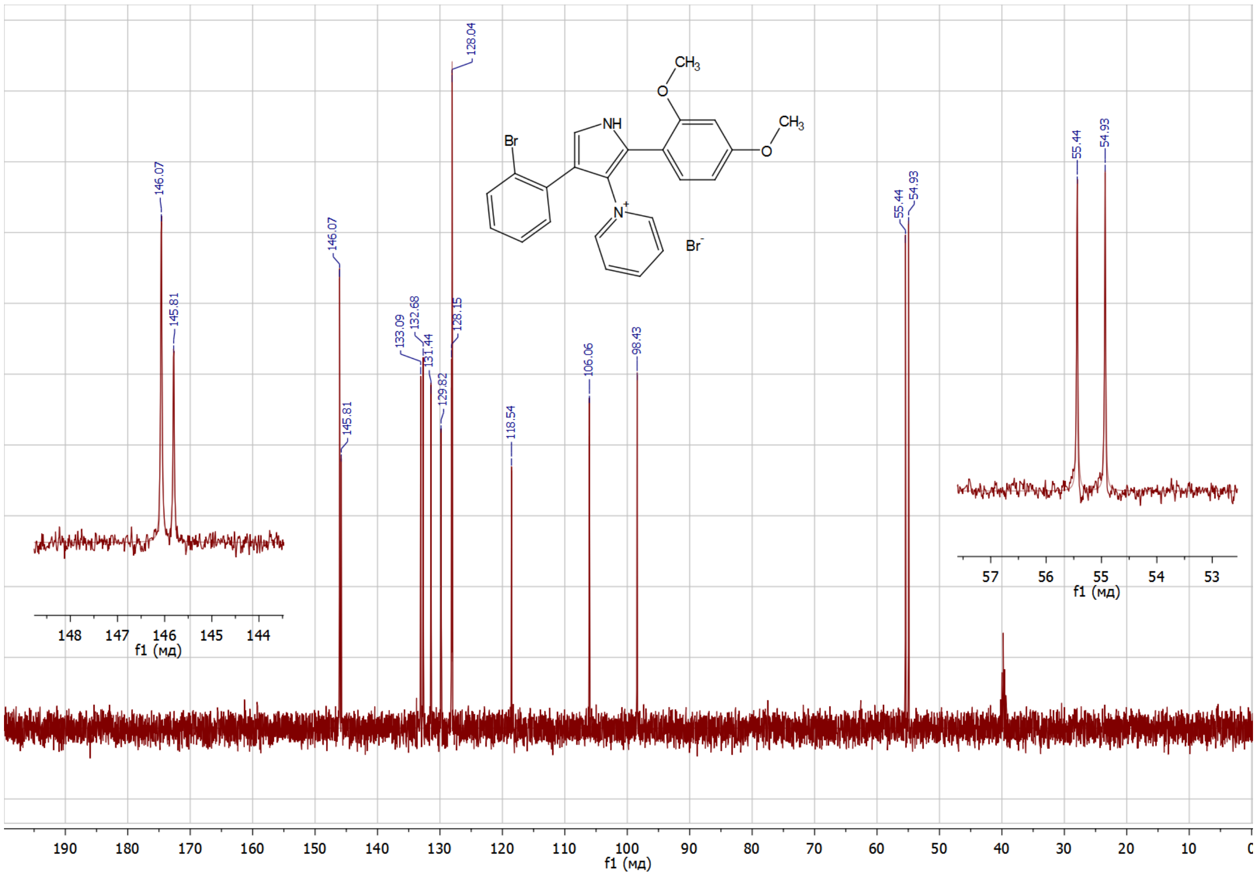

^{13}C{1H} NMR (101 MHz, DMSO-d_6) of ^{1}H NMR (400 MHz, DMSO-d_6) of 1-(4-(2-bromophenyl)-2-(4-nitrophenyl)-1*H*-pyrrol-3-yl)pyridin-1-ium bromide (**1j**)

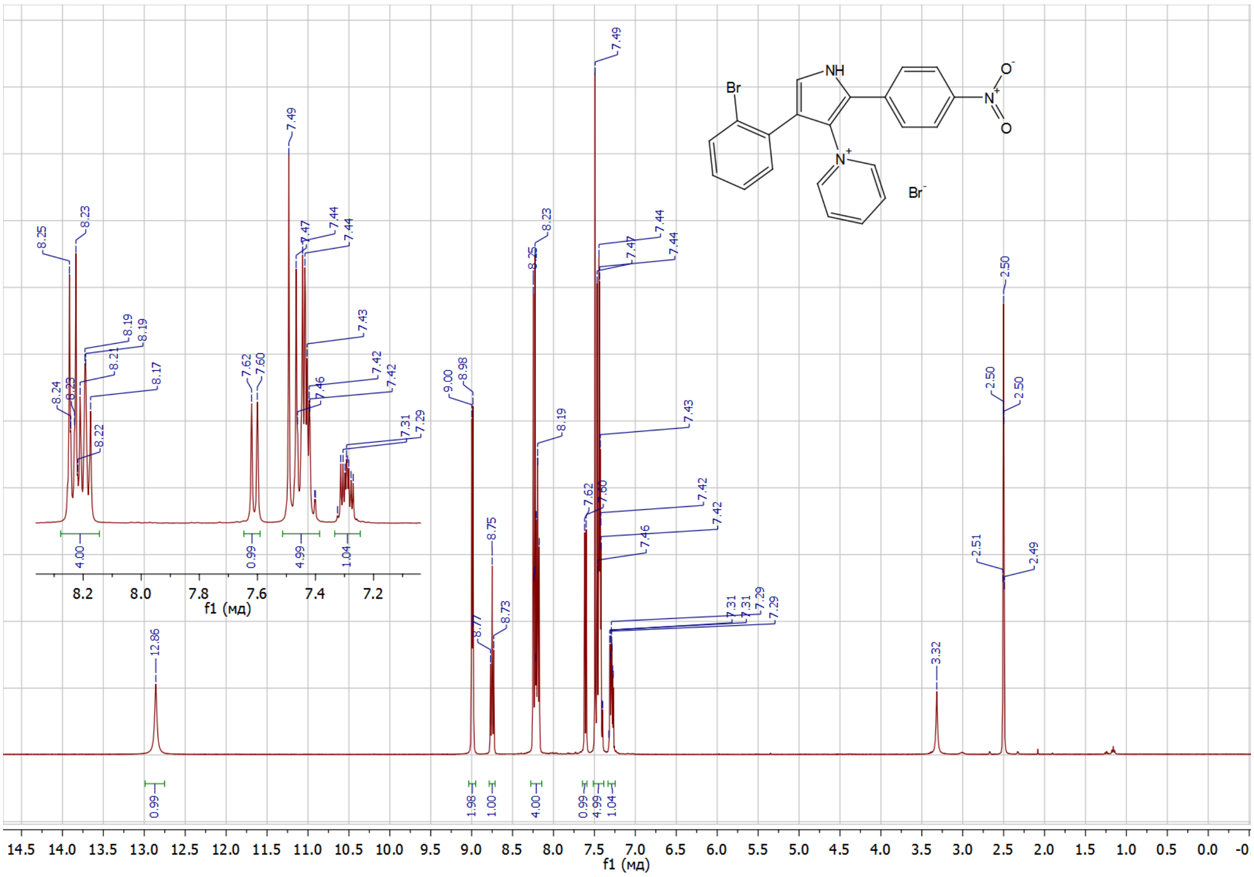

^{1}H NMR (400 MHz, DMSO-d_6) of 1-(4-(2-bromophenyl)-2-(4-nitrophenyl)-1*H*-pyrrol-3-yl)pyridin-1-ium bromide (**1j**)

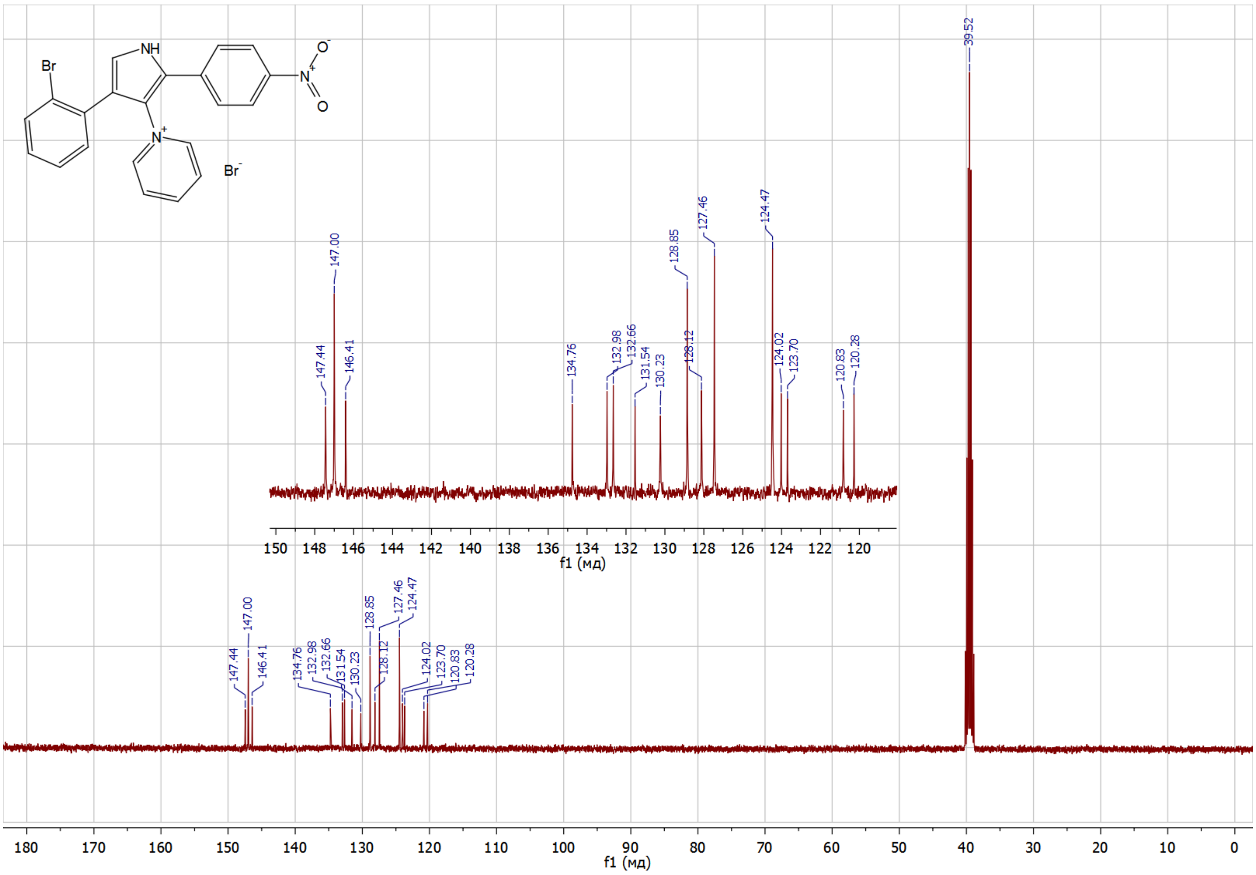

^{13}C DEPT135 NMR (101 MHz, DMSO-d_6) of 1-(4-(2-bromophenyl)-2-(4-nitrophenyl)-1*H*-pyrrol-3-yl)pyridin-1-ium bromide (**1j**)

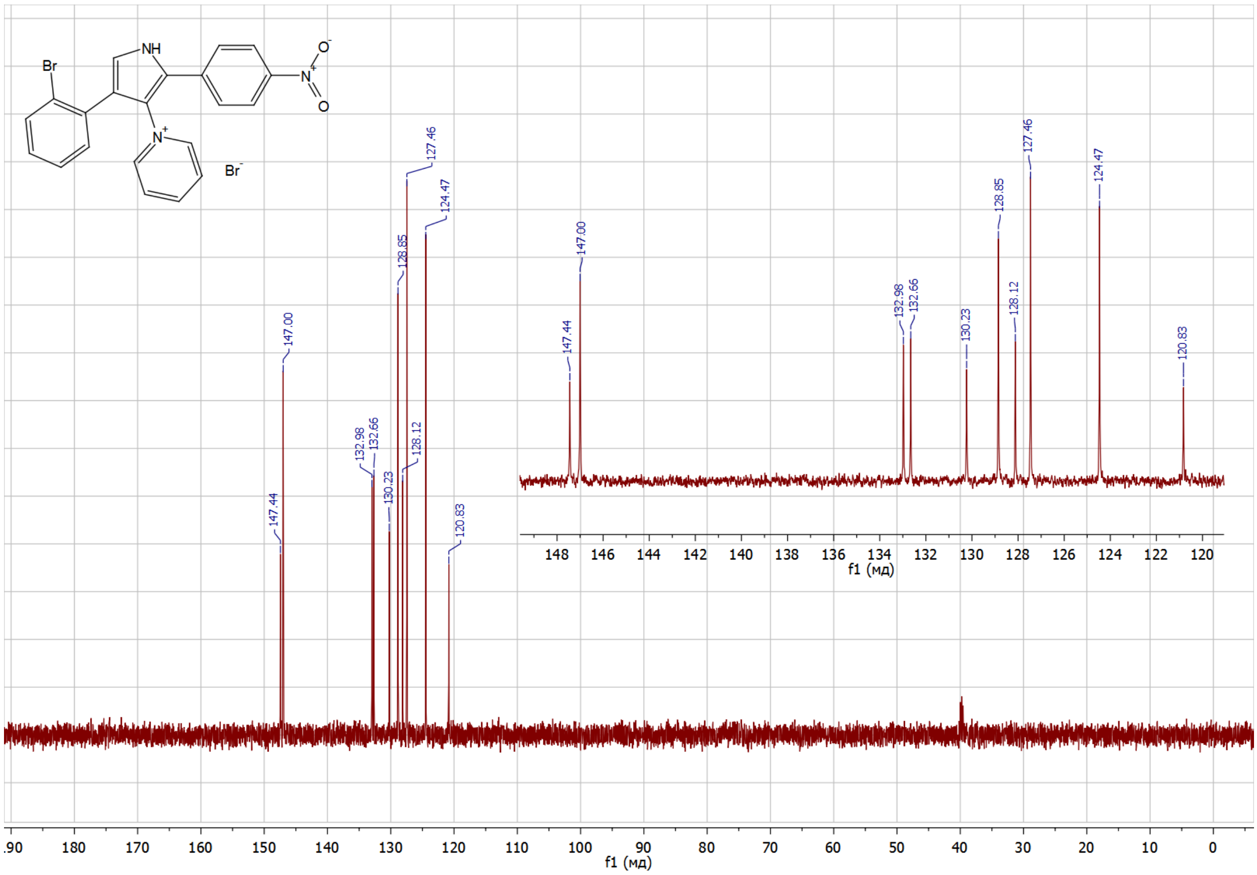

^{1}H NMR (400 MHz, DMSO-d_6) of 1-(4-(2-bromophenyl)-2-(3-nitrophenyl)-1*H*-pyrrol-3-yl)pyridin-1-ium bromide (**1k**)

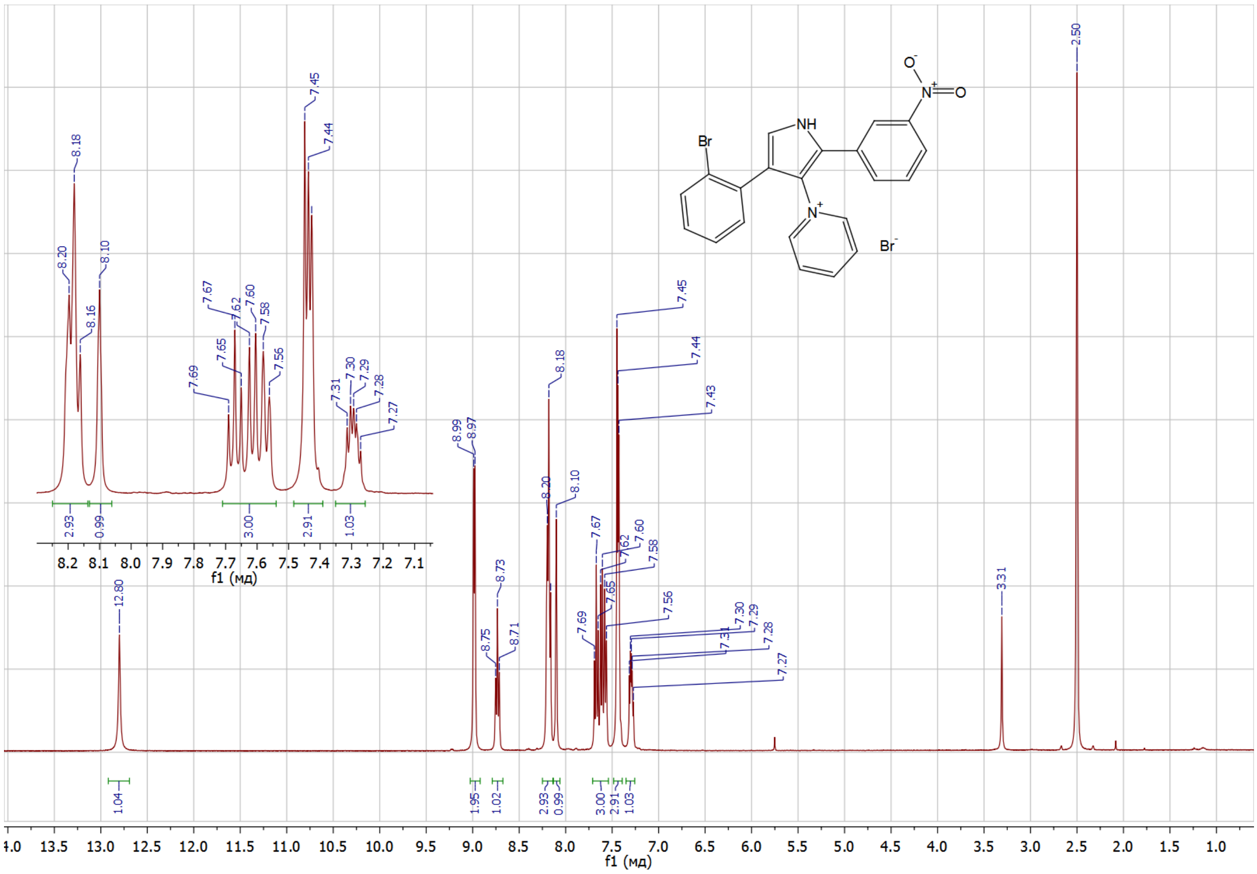

^{13}C{1H} NMR (101 MHz, DMSO-d_6) of 1-(4-(2-bromophenyl)-2-(3-nitrophenyl)-1*H*-pyrrol-3-yl)pyridin-1-ium bromide (**1k**)

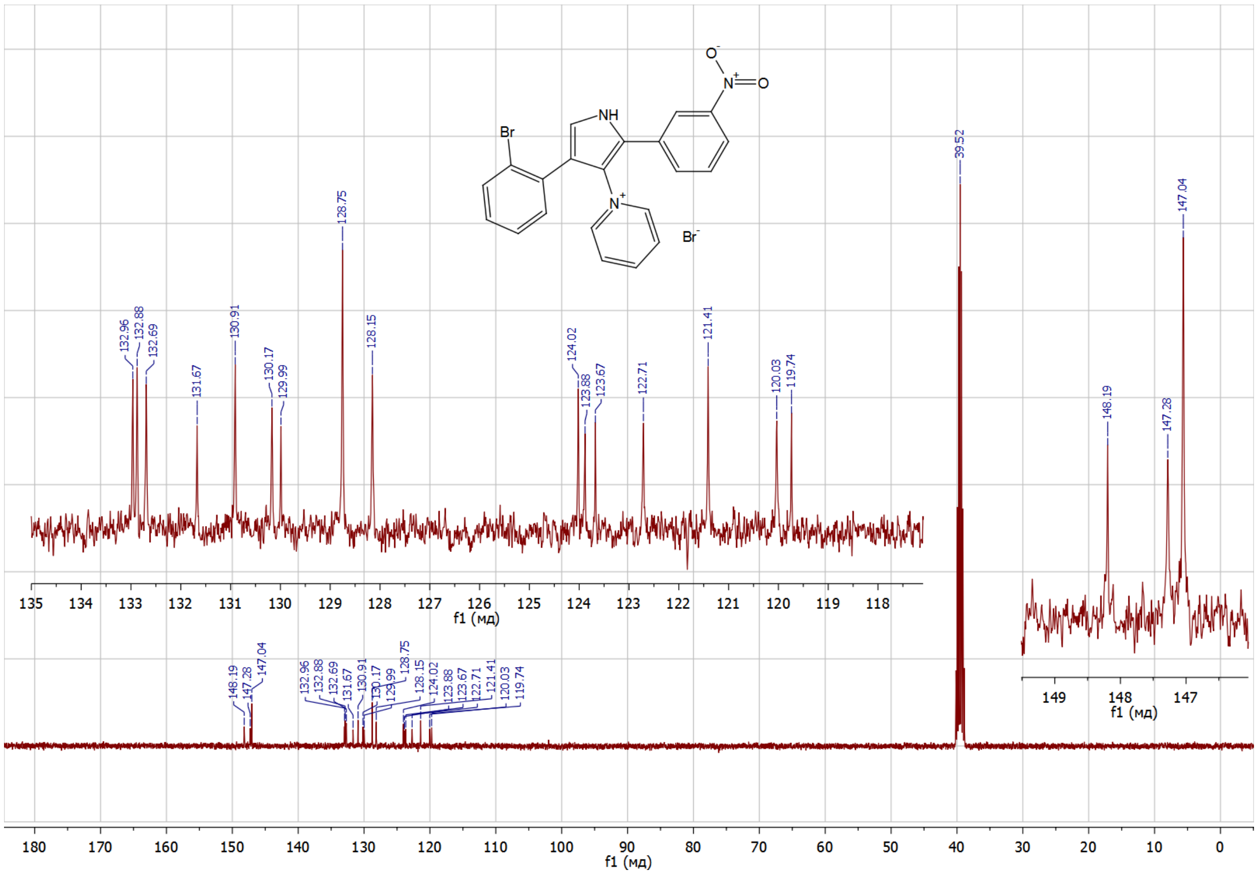

^{13}C DEPT135 NMR (101 MHz, DMSO-d_6) of 1-(4-(2-bromophenyl)-2-(3-nitrophenyl)-1*H*-pyrrol-3-yl)pyridin-1-ium bromide (**1k**)

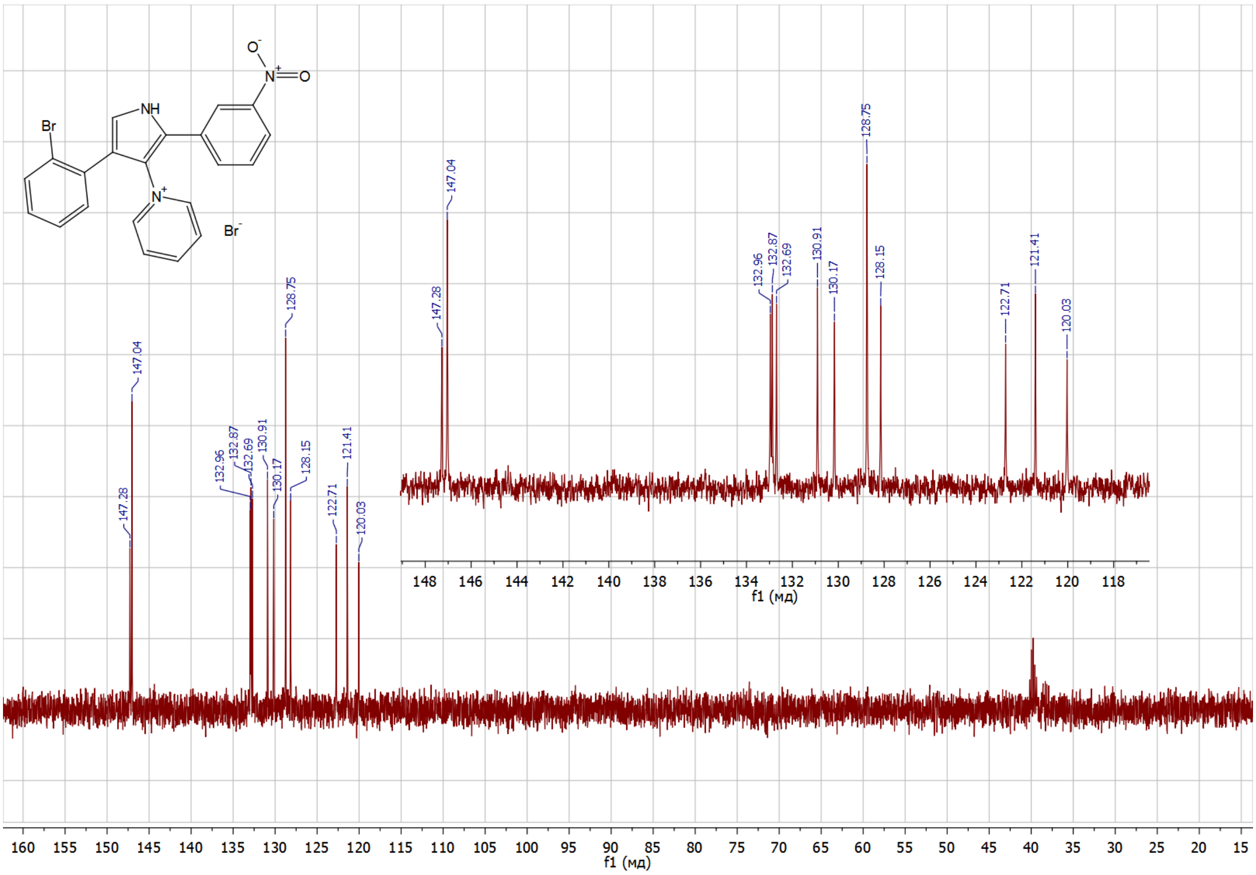

^{1}H NMR (400 MHz, DMSO-d_6) of 1-(4-(2-bromophenyl)-2-(4-cyanophenyl)-1*H*-pyrrol-3-yl)pyridin-1-ium bromide (**1l**)

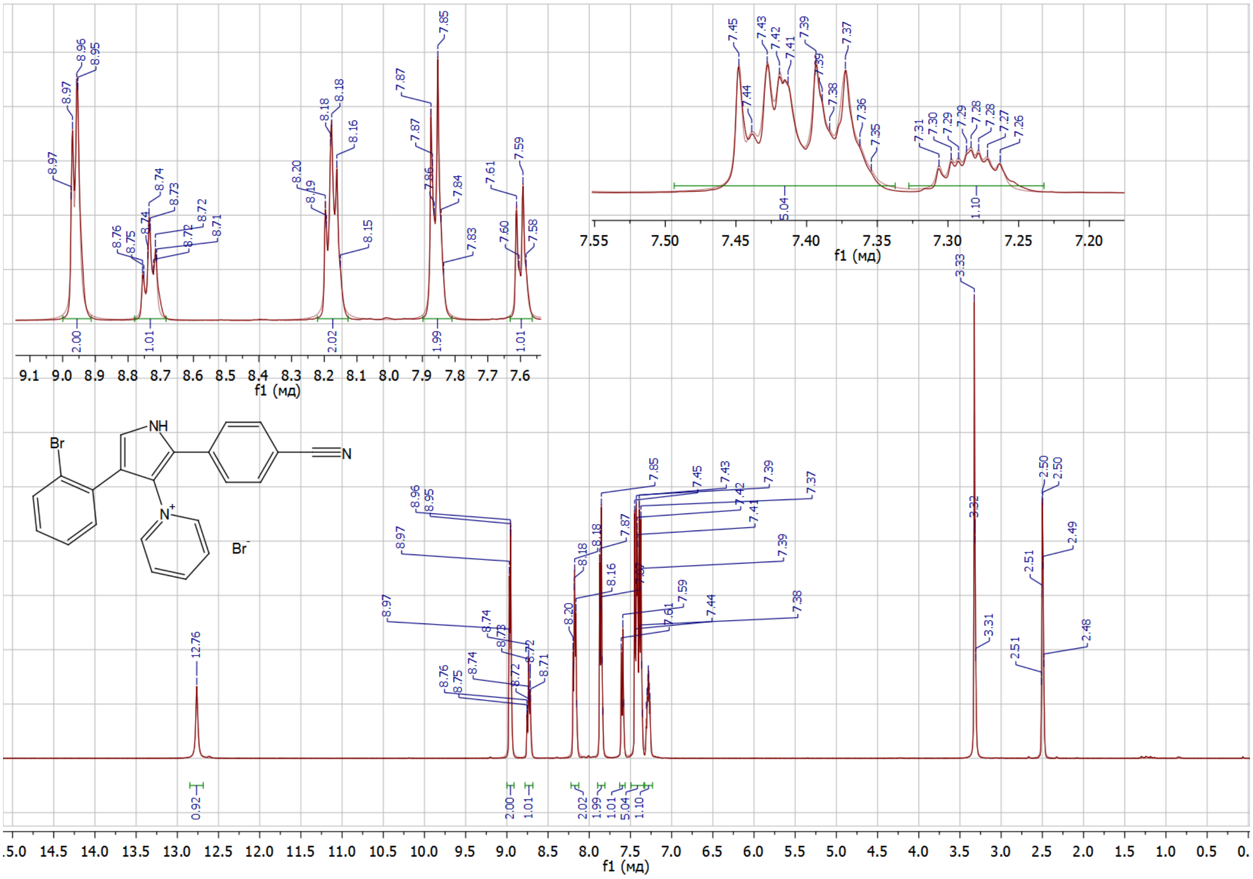

^{13}C{1H} NMR (101 MHz, DMSO-d_6) of 1-(4-(2-bromophenyl)-2-(4-cyanophenyl)-1*H*-pyrrol-3-yl)pyridin-1-ium bromide (**1l**)

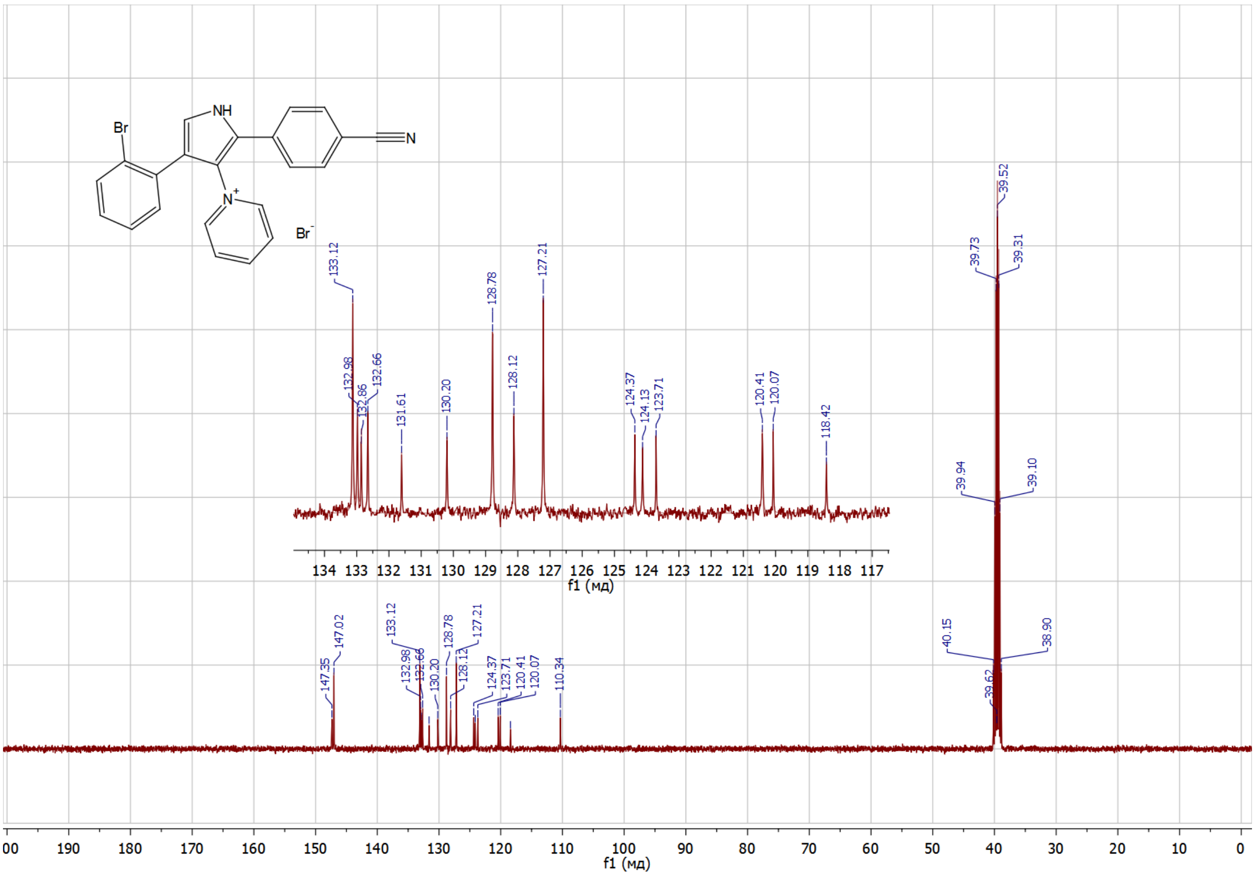

^{13}C DEPT135 NMR (101 MHz, DMSO-d_6) of 1-(4-(2-bromophenyl)-2-(4-cyanophenyl)-1*H*-pyrrol-3-yl)pyridin-1-ium bromide (**1l**)

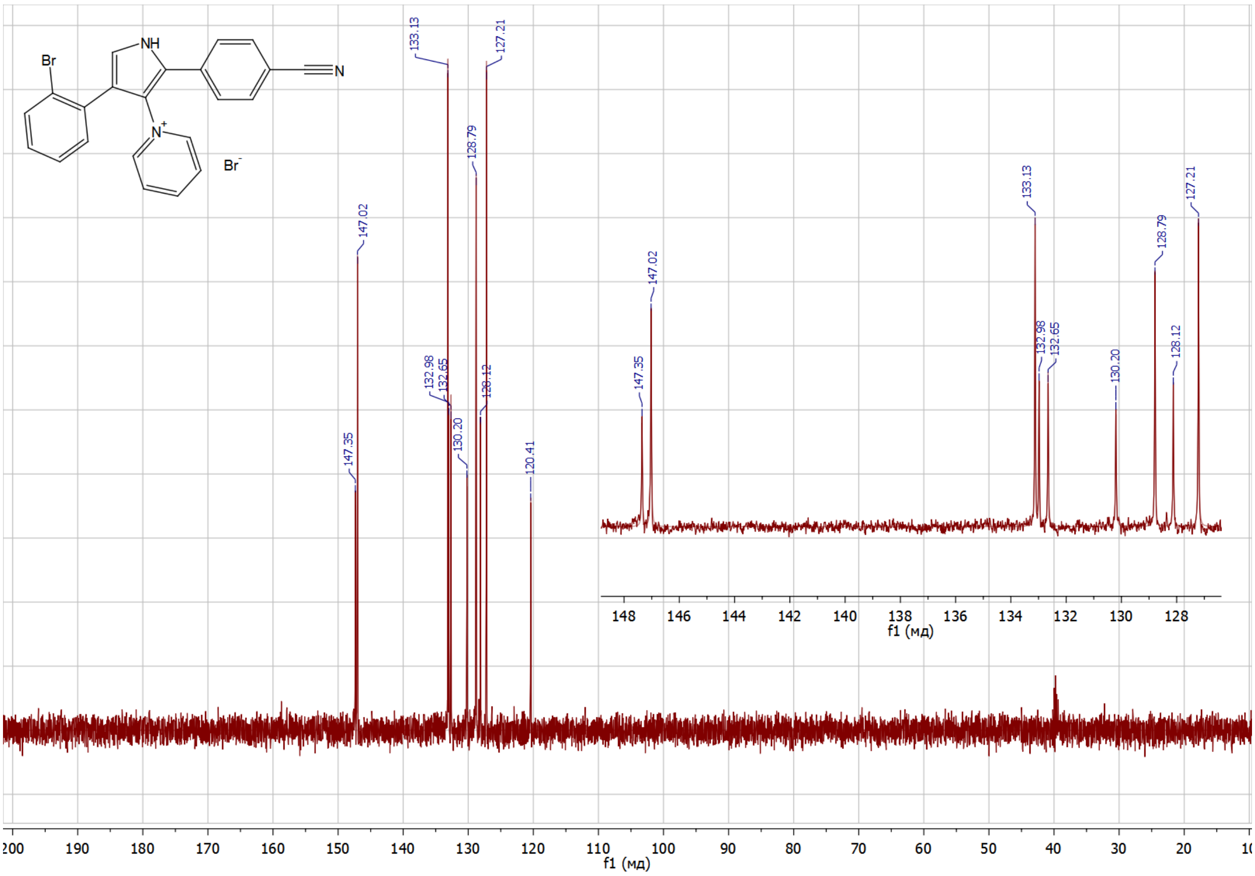

^{1}H NMR (400 MHz, DMSO-d_6) of 1-(4-(2-bromophenyl)-2-(pyridin-2-yl)-1*H*-pyrrol-3-yl)pyridin-1-ium iodide (**1m**)

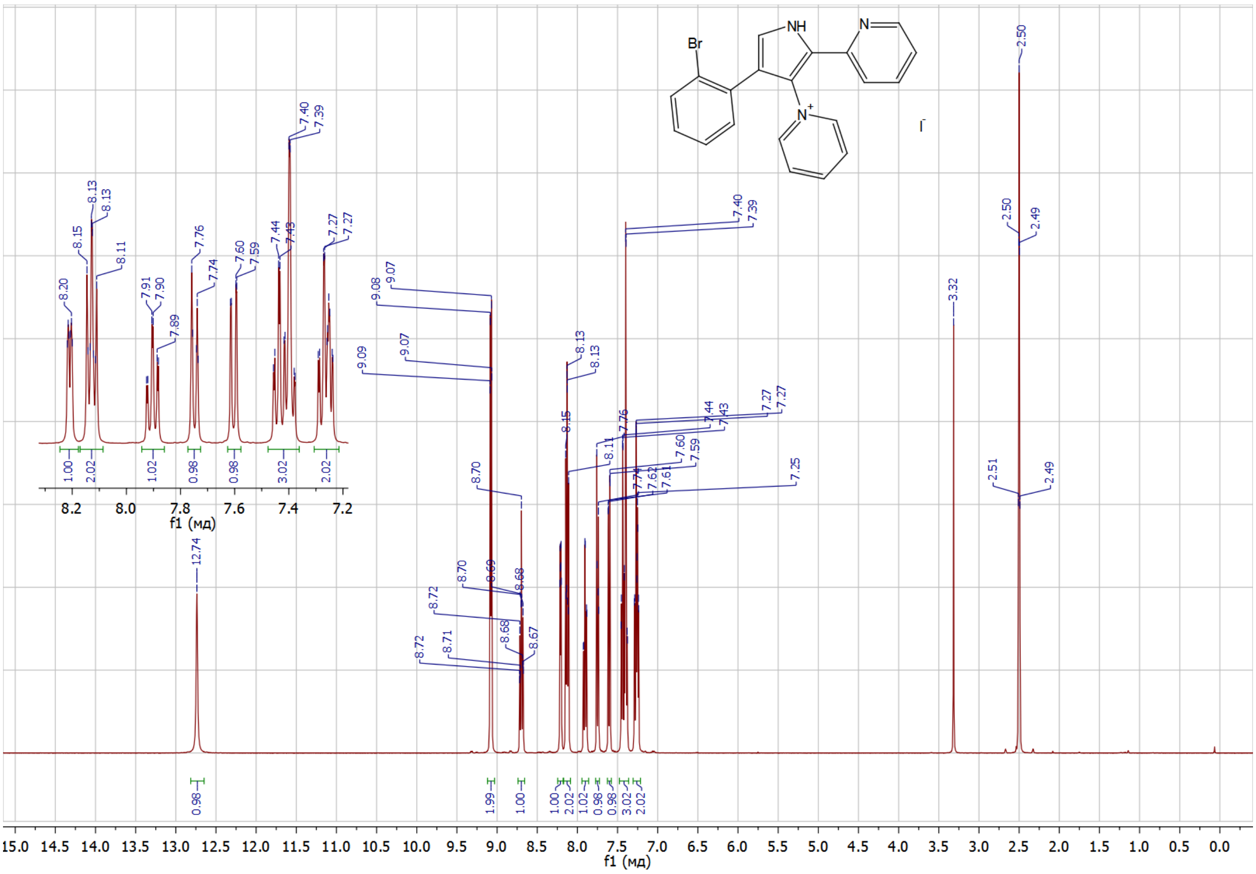

$^{13}C\{^{1}H\}$ NMR (101 MHz, DMSO-d_6) of 1-(4-(2-bromophenyl)-2-(pyridin-2-yl)-1*H*-pyrrol-3-yl)pyridin-1-ium iodide (**1m**)

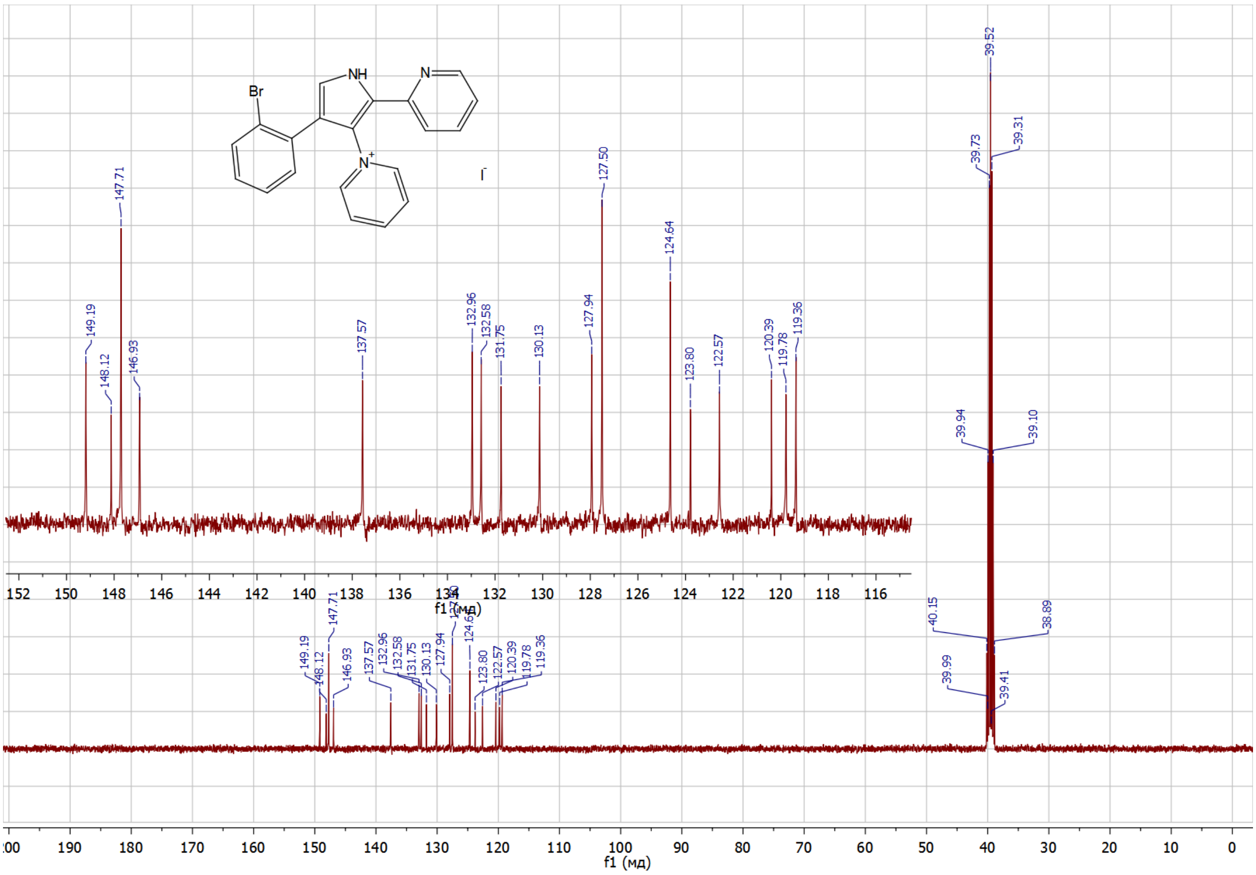

^{13}C DEPT135 NMR (101 MHz, DMSO-d_6) of 1-(4-(2-bromophenyl)-2-(pyridin-2-yl)-1*H*-pyrrol-3-yl)pyridin-1-ium iodide (**1m**)

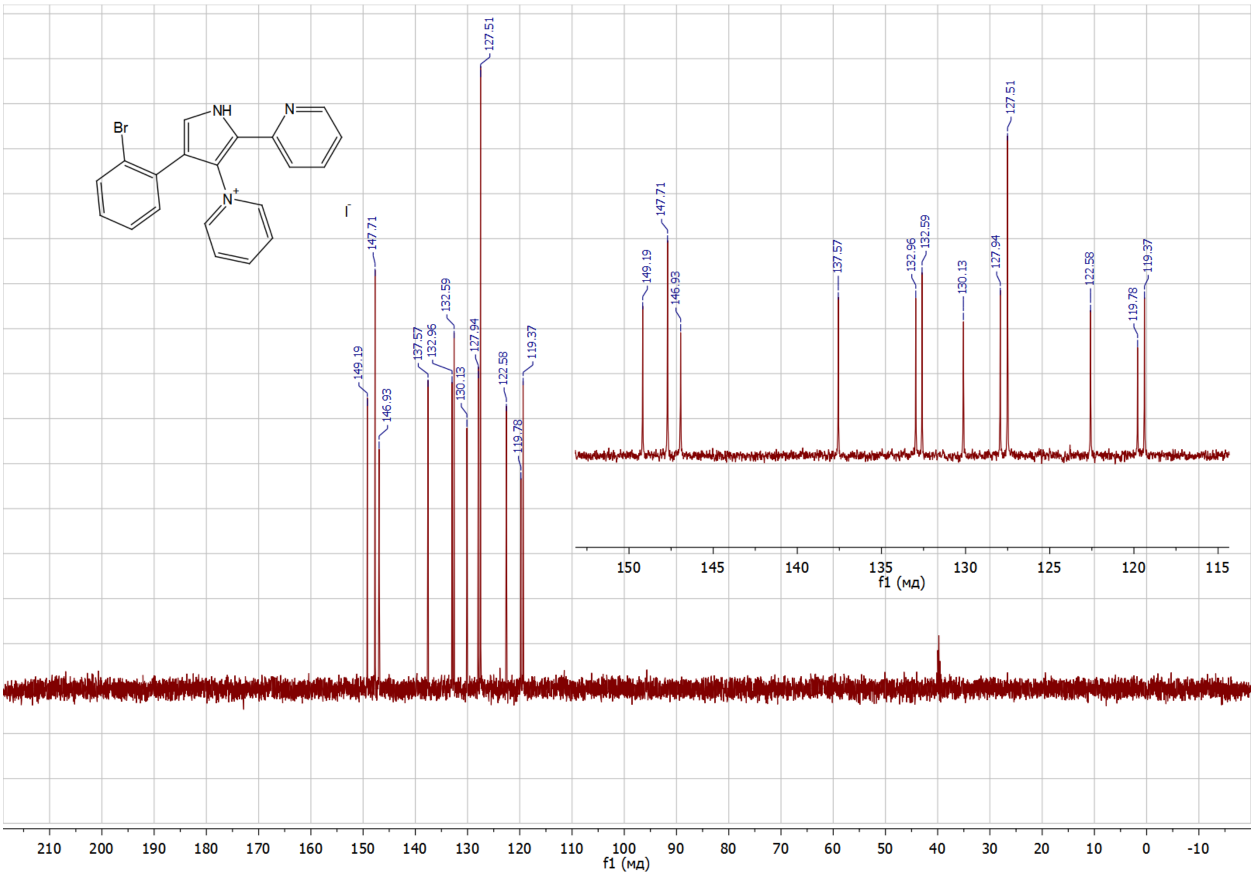

^{1}H NMR (400 MHz, DMSO-d_6) of 1-(4-(2-bromophenyl)-2-phenyl-1*H*-pyrrol-3-yl)-4-methylpyridin-1-ium bromide (**1n**)

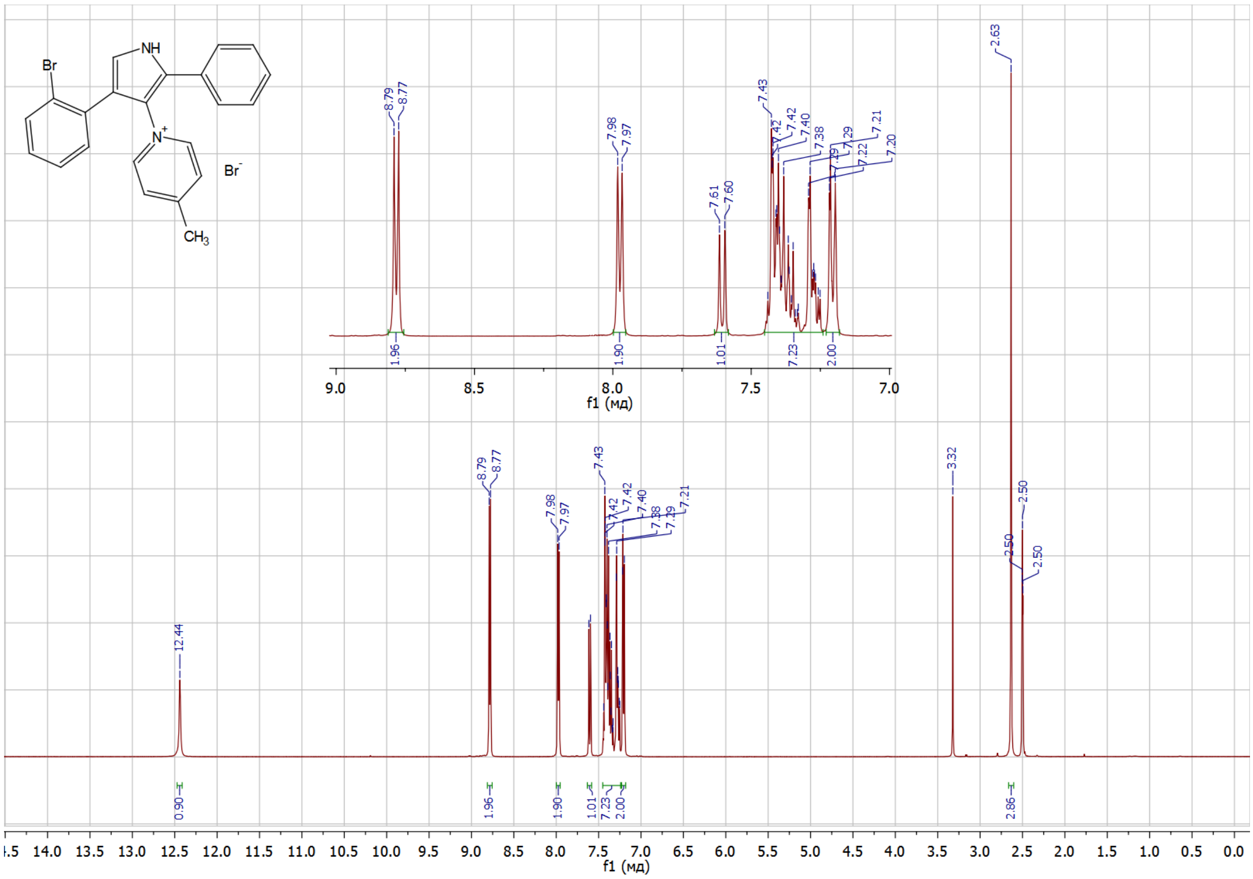

^{13}C{1H} NMR (101 MHz, DMSO-d_6) of 1-(4-(2-bromophenyl)-2-phenyl-1*H*-pyrrol-3-yl)-4-methylpyridin-1-ium bromide (**1n**)

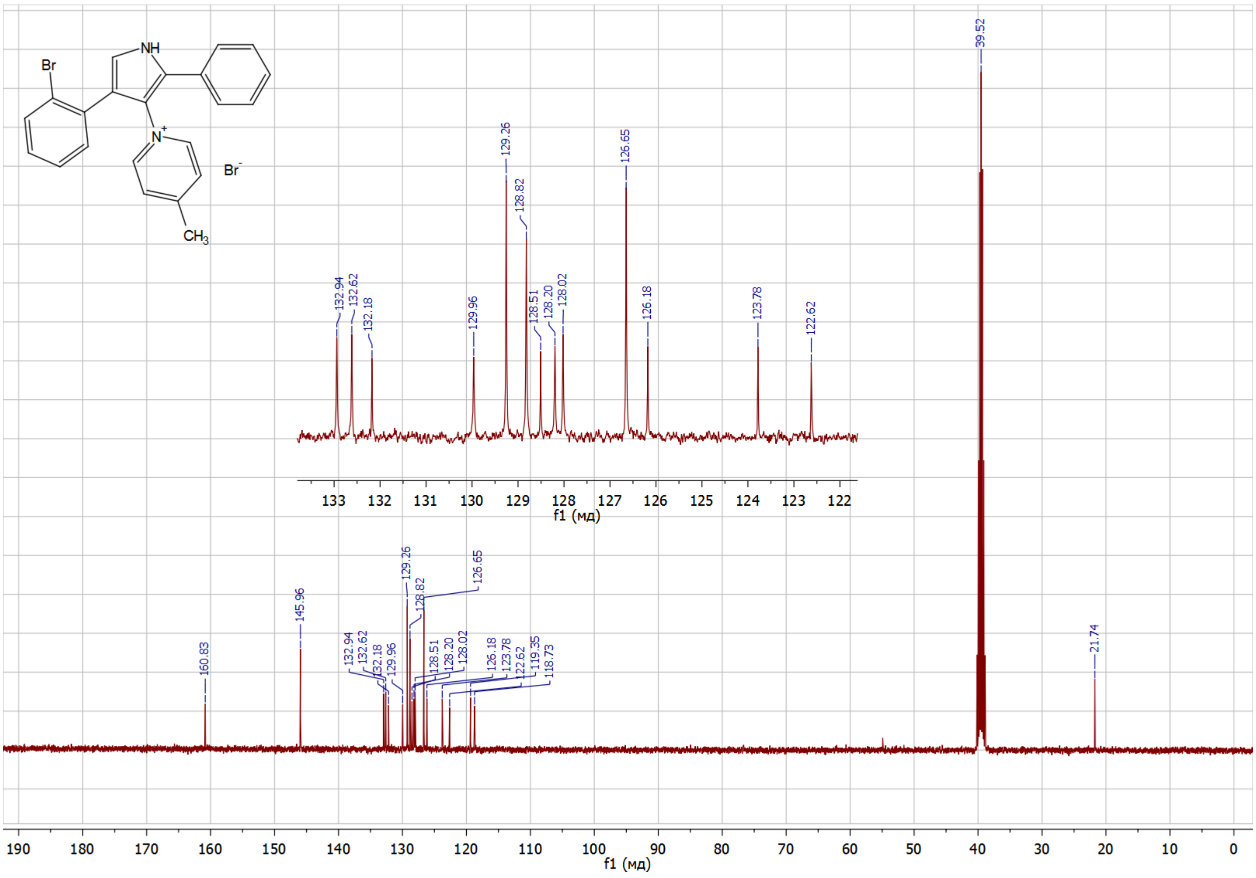

^{13}C DEPT135 NMR (101 MHz, DMSO-d_6) of 1-(4-(2-bromophenyl)-2-phenyl-1*H*-pyrrol-3-yl)-4-methylpyridin-1-ium bromide (**1n**)

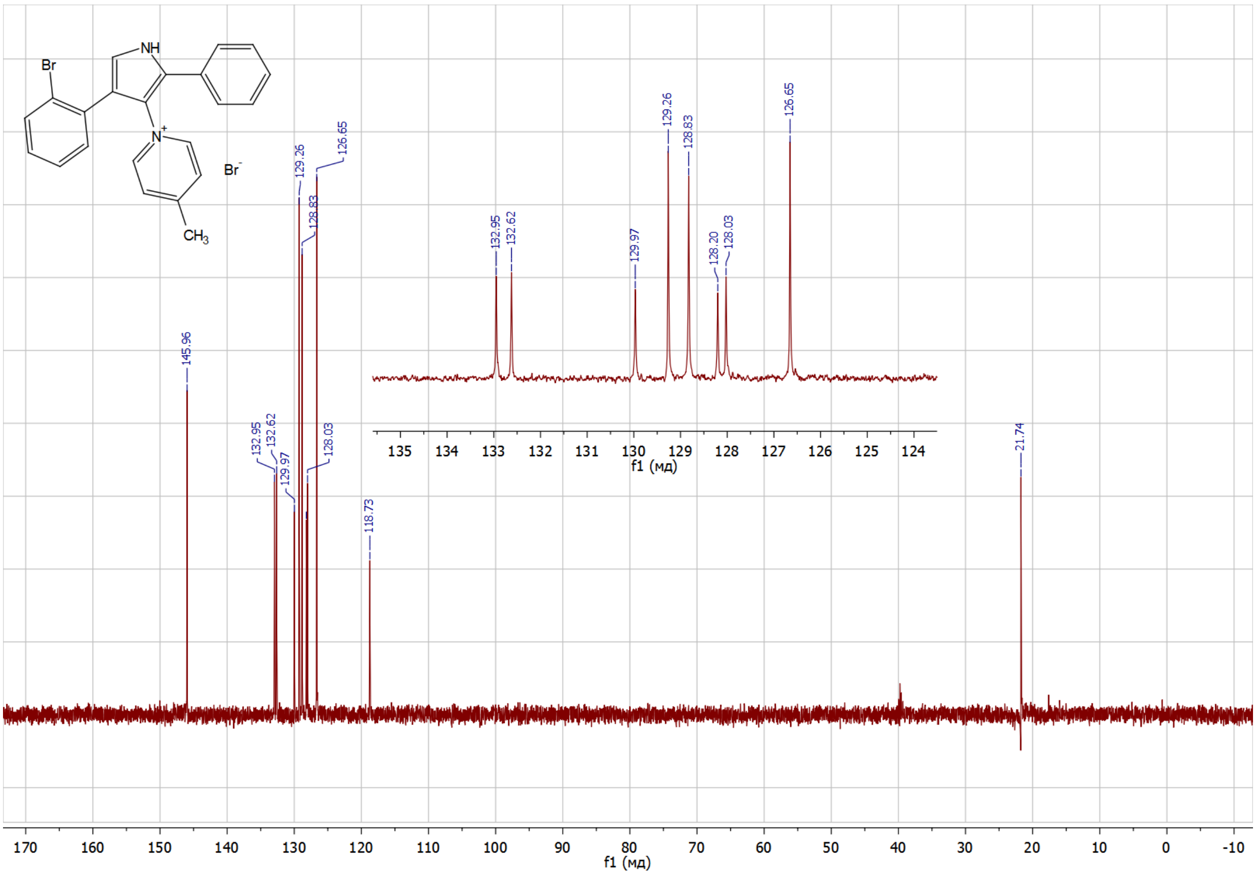

^1H NMR (400 MHz, DMSO-d_6) of 1-(4-(2-bromophenyl)-2-phenyl-1*H*-pyrrol-3-yl)-4-methoxypyridin-1-ium bromide (**1o**)

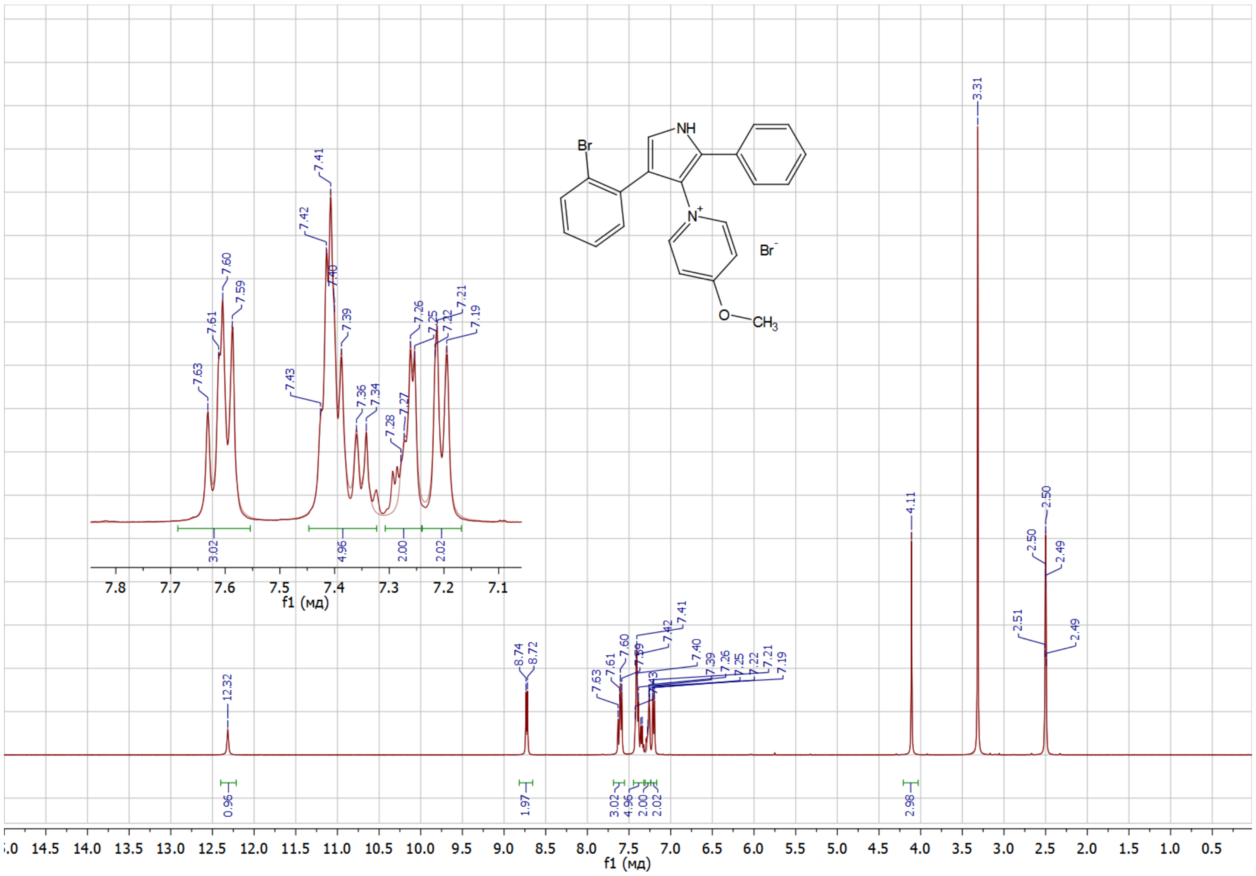

$^{13}C\{1H\}$ NMR (101 MHz, DMSO-d_6) of 1-(4-(2-bromophenyl)-2-phenyl-1*H*-pyrrol-3-yl)-4-methoxypyridin-1-ium bromide (**1o**)

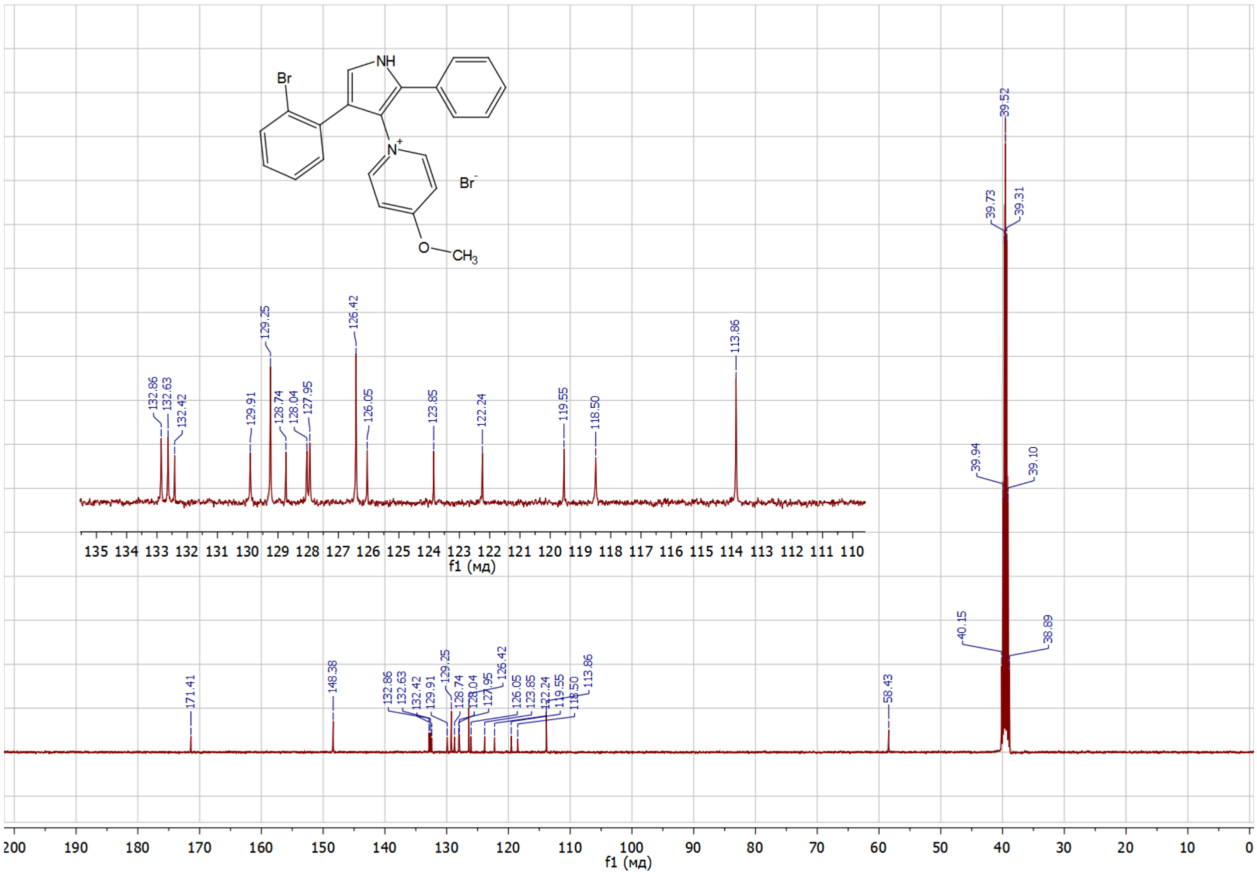

^{13}C DEPT135 NMR (101 MHz, DMSO-d_6) of 1-(4-(2-bromophenyl)-2-phenyl-1*H*-pyrrol-3-yl)-4-methoxypyridin-1-ium bromide (**1o**)

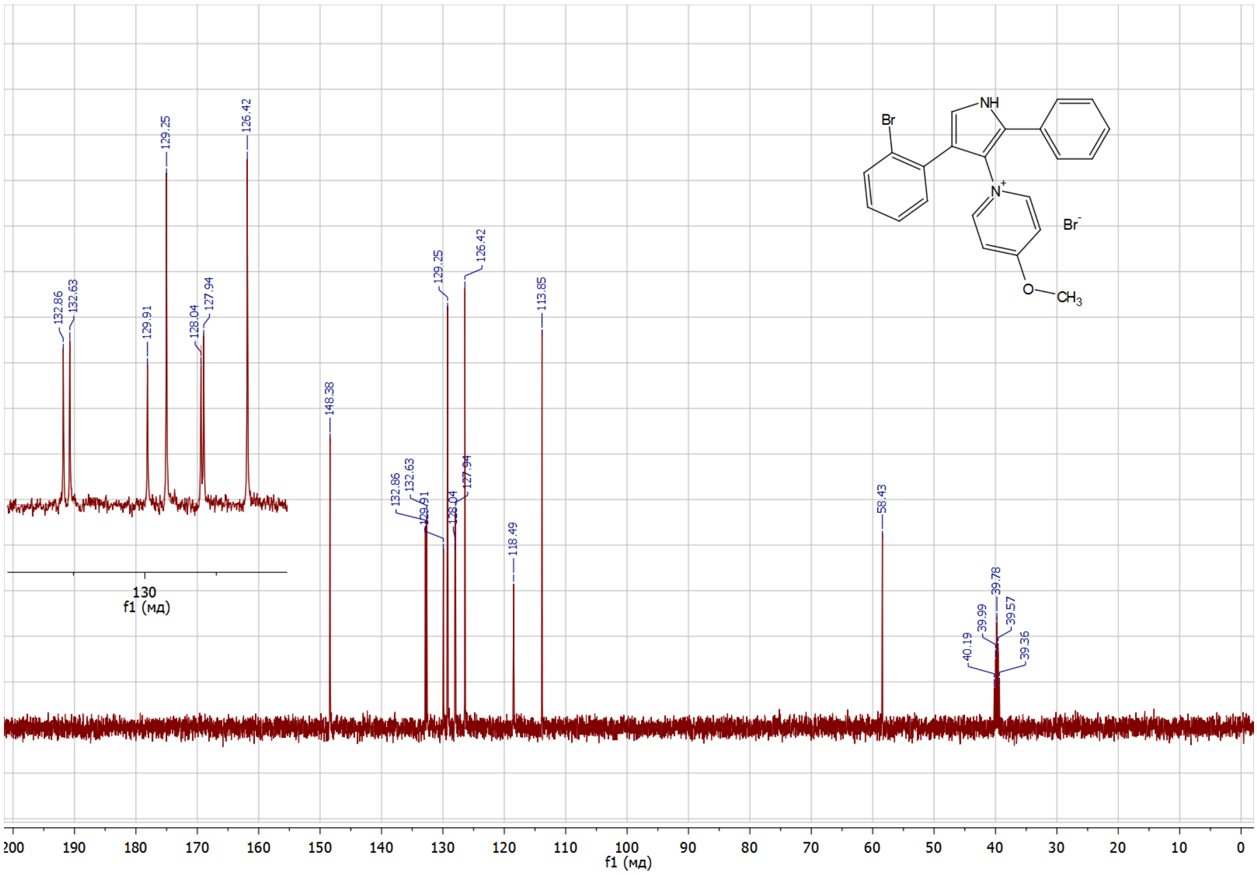

^{1}H NMR (400 MHz, DMSO-d_6) of 1-(4-(2-bromophenyl)-2-phenyl-1*H*-pyrrol-3-yl)-4-(dimethylamino)pyridin-1-ium bromide (**1p**)

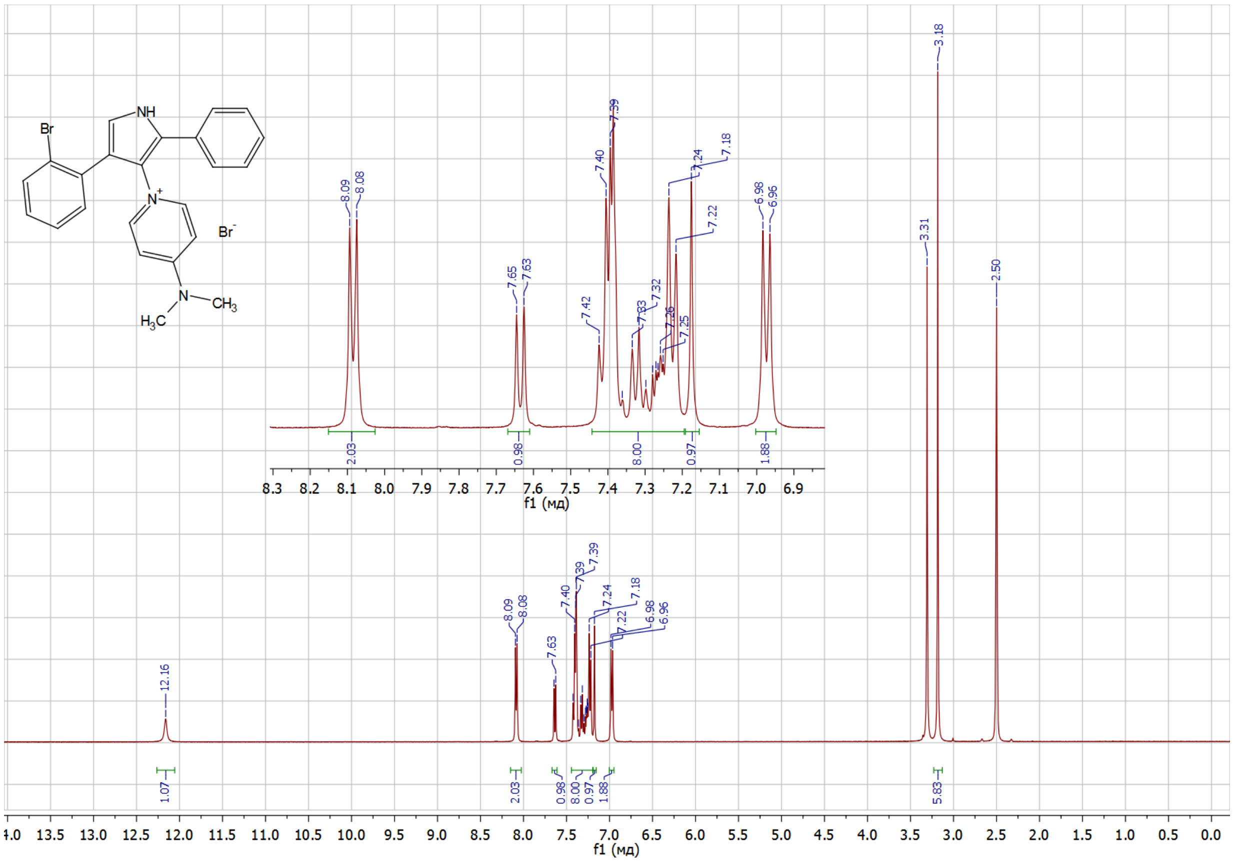

^{13}C{1H} NMR (101 MHz, DMSO-d_6) of 1-(4-(2-bromophenyl)-2-phenyl-1*H*-pyrrol-3-yl)-4-(dimethylamino)pyridin-1-ium bromide (**1p**)

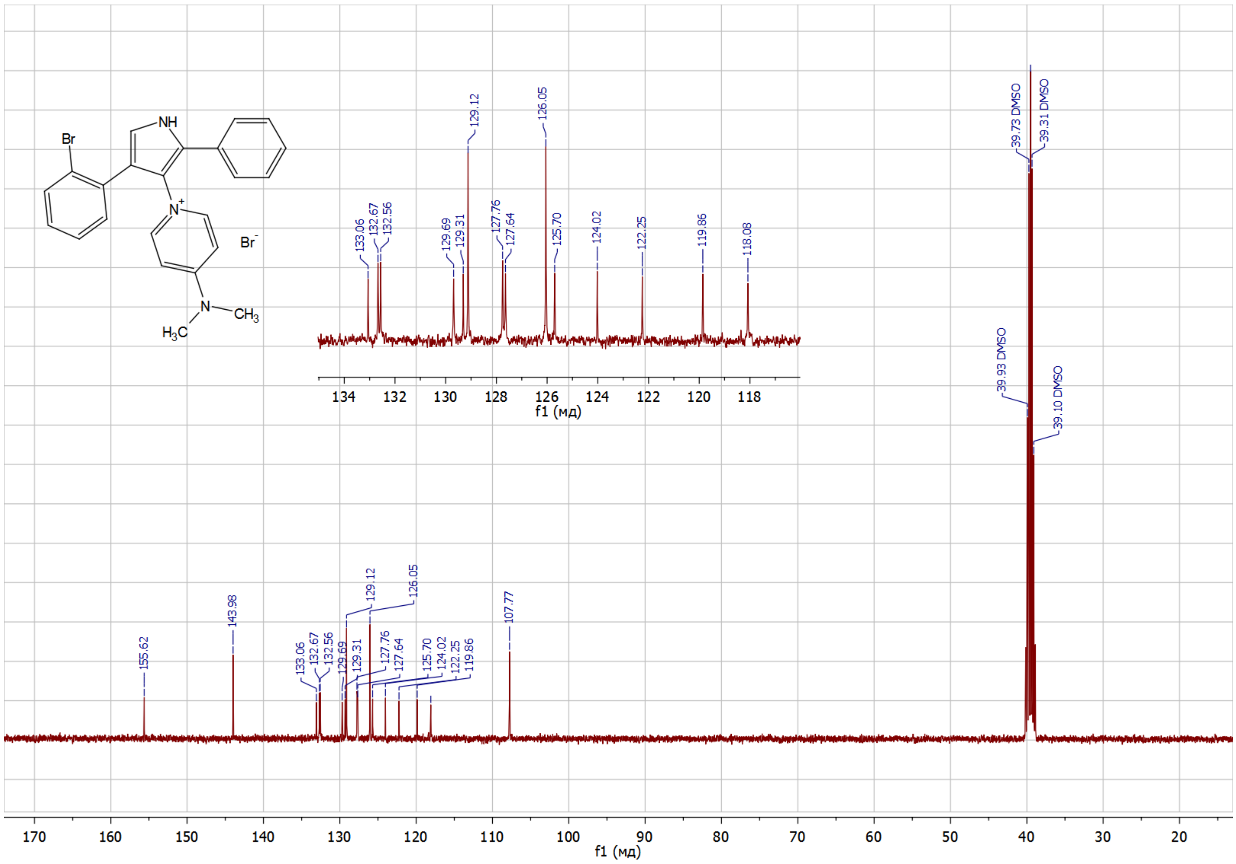

^{13}C DEPT135 NMR (101 MHz, DMSO-d_6) of 1-(4-(2-bromophenyl)-2-phenyl-1*H*-pyrrol-3-yl)-4-(dimethylamino)pyridin-1-ium bromide (**1p**)

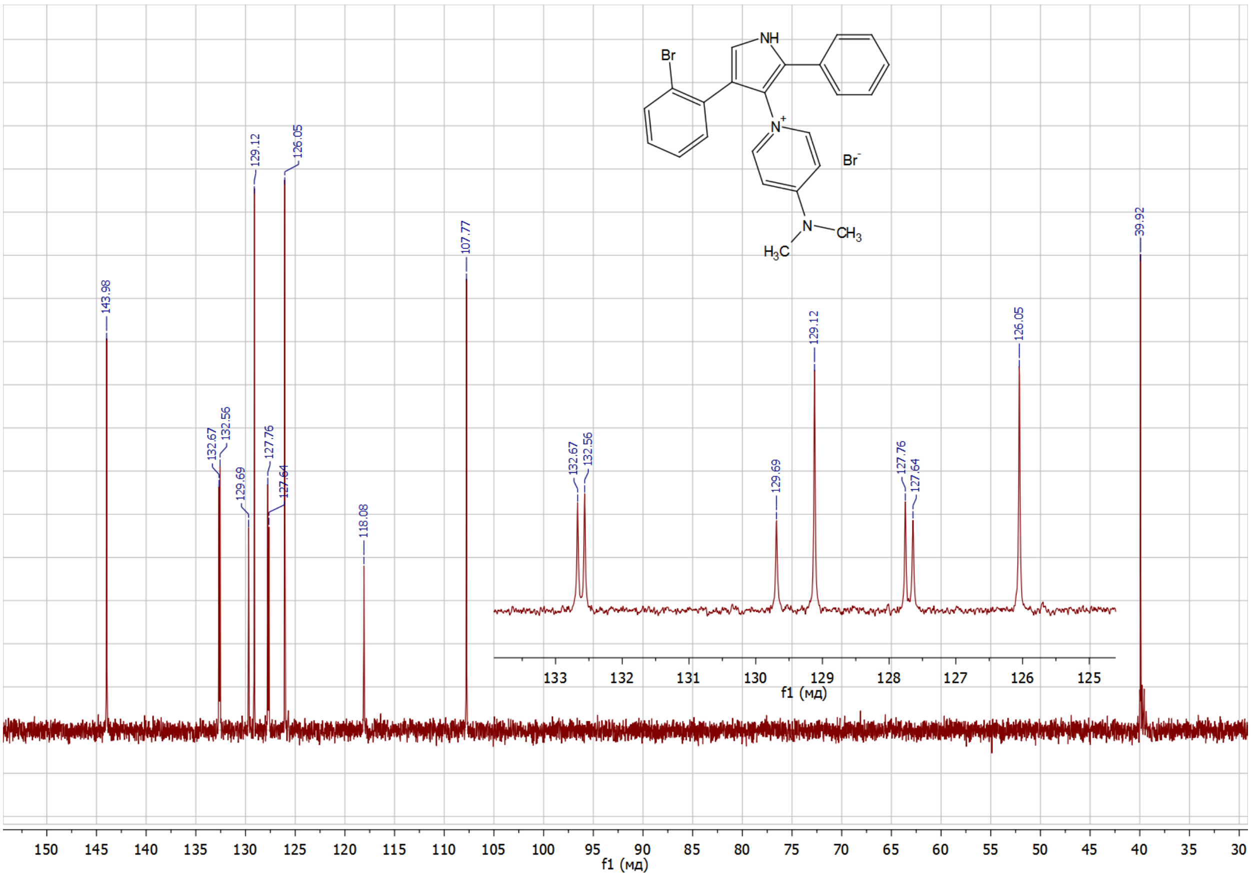

^{1}H NMR (400 MHz, DMSO-d_6) of 1-(4-(2-bromophenyl)-2-phenyl-1*H*-pyrrol-3-yl)-4-phenylpyridin-1-ium bromide (**1q**)

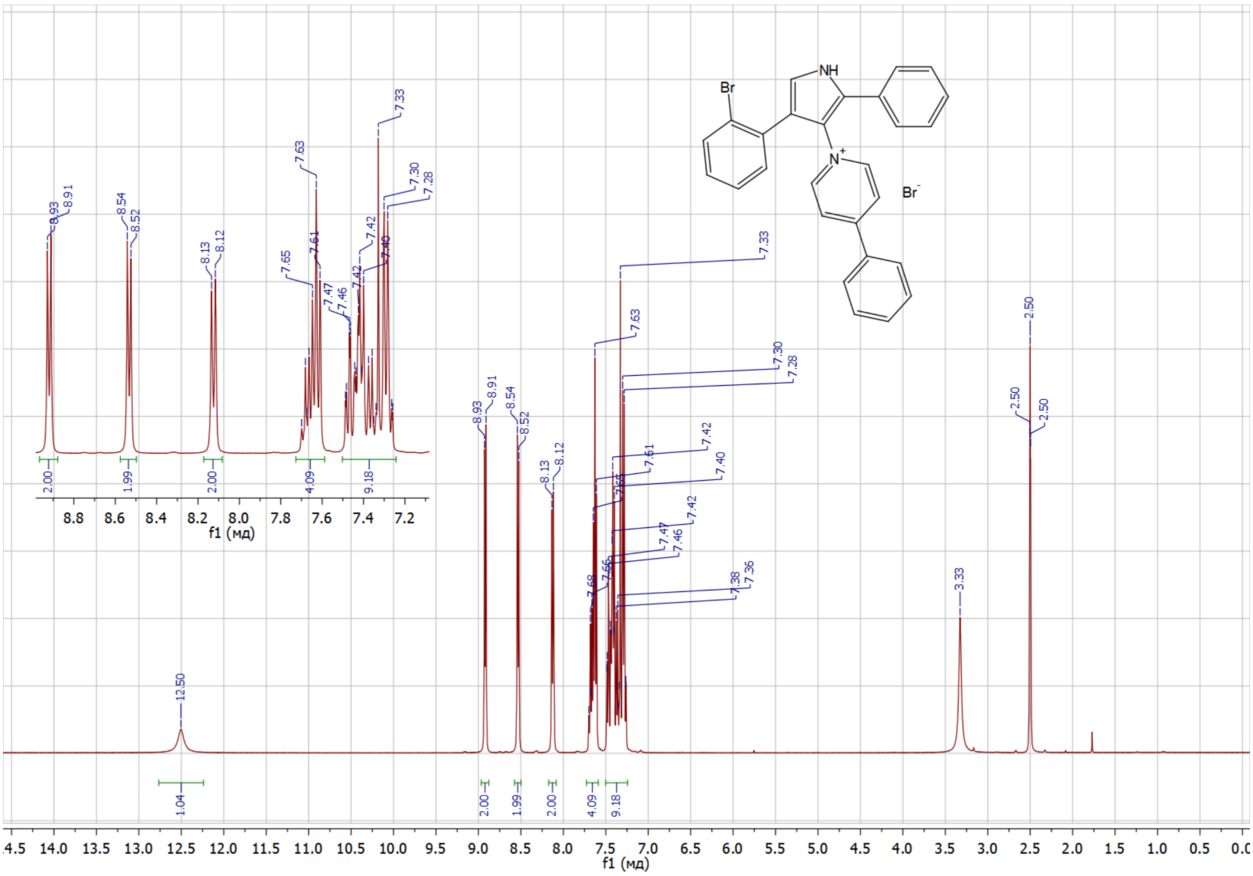

$^{13}C\{1H\}$ NMR (101 MHz, DMSO-d_6) of 1-(4-(2-bromophenyl)-2-phenyl-1*H*-pyrrol-3-yl)-4-phenylpyridin-1-ium bromide (**1q**)

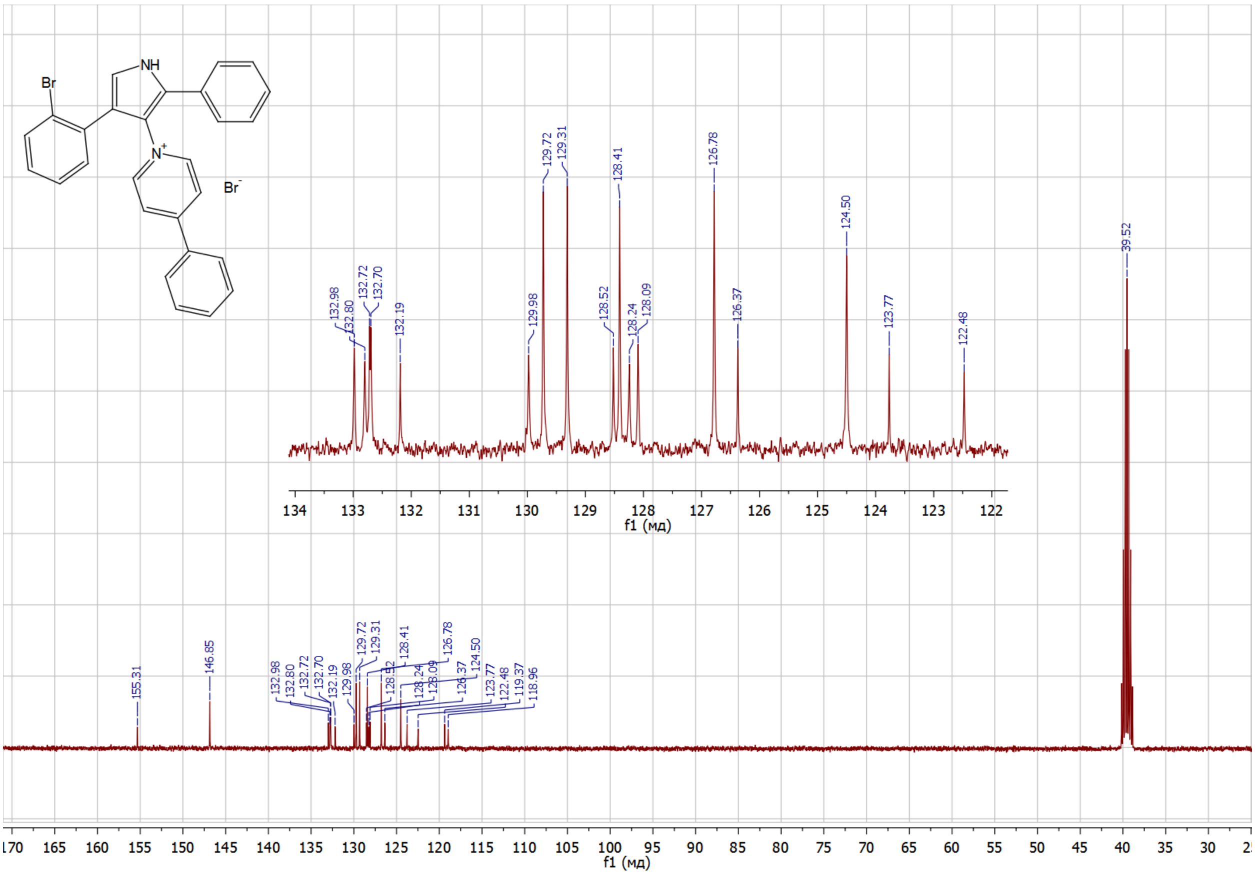

^{13}C DEPT135 NMR (101 MHz, DMSO-d_6) of 1-(4-(2-bromophenyl)-2-phenyl-1*H*-pyrrol-3-yl)-4-phenylpyridin-1-ium bromide (**1q**)

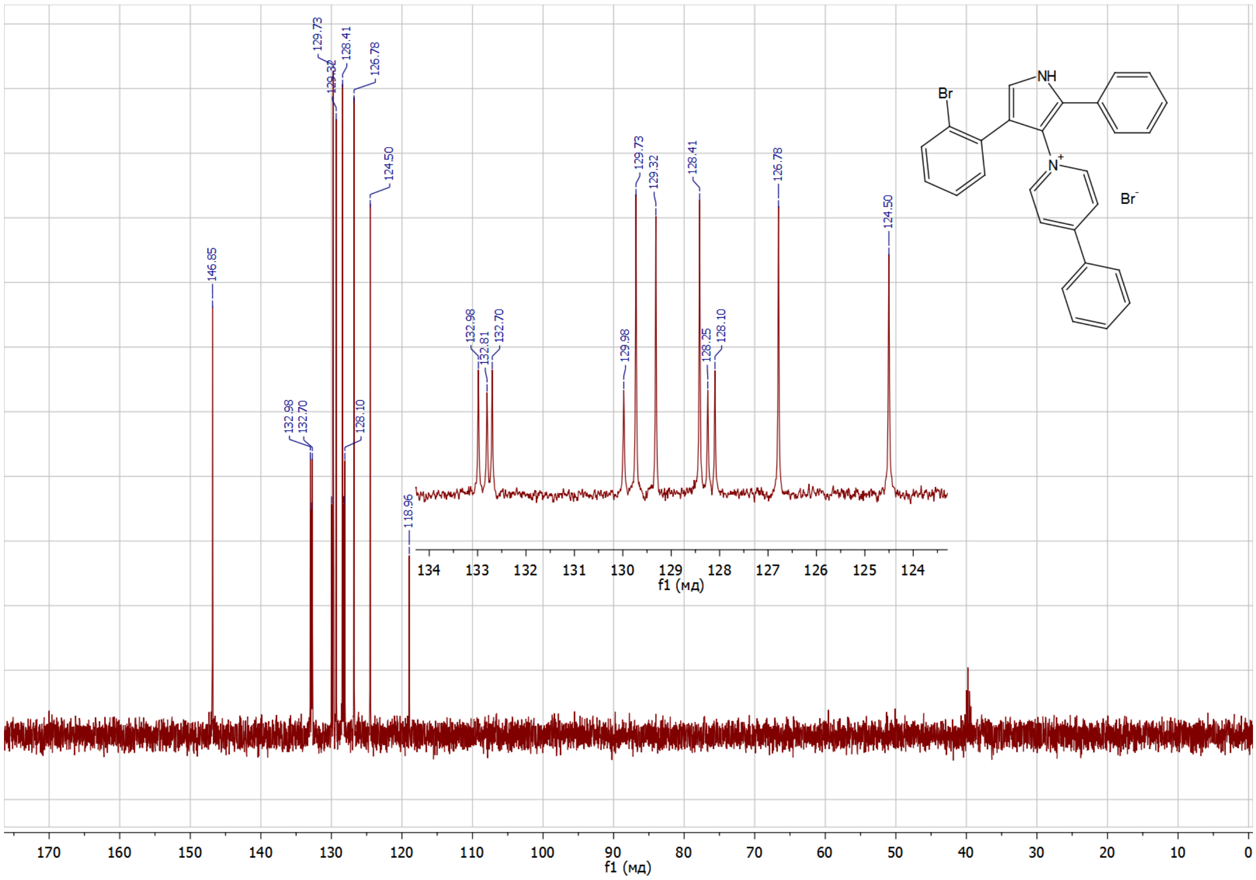

^{1}H NMR (400 MHz, DMSO-d_6) of 1-(4-(2-bromophenyl)-2-phenyl-1H-pyrrol-3-yl)-4-(4-methoxyphenyl)pyridin-1-ium bromide (**1r**)

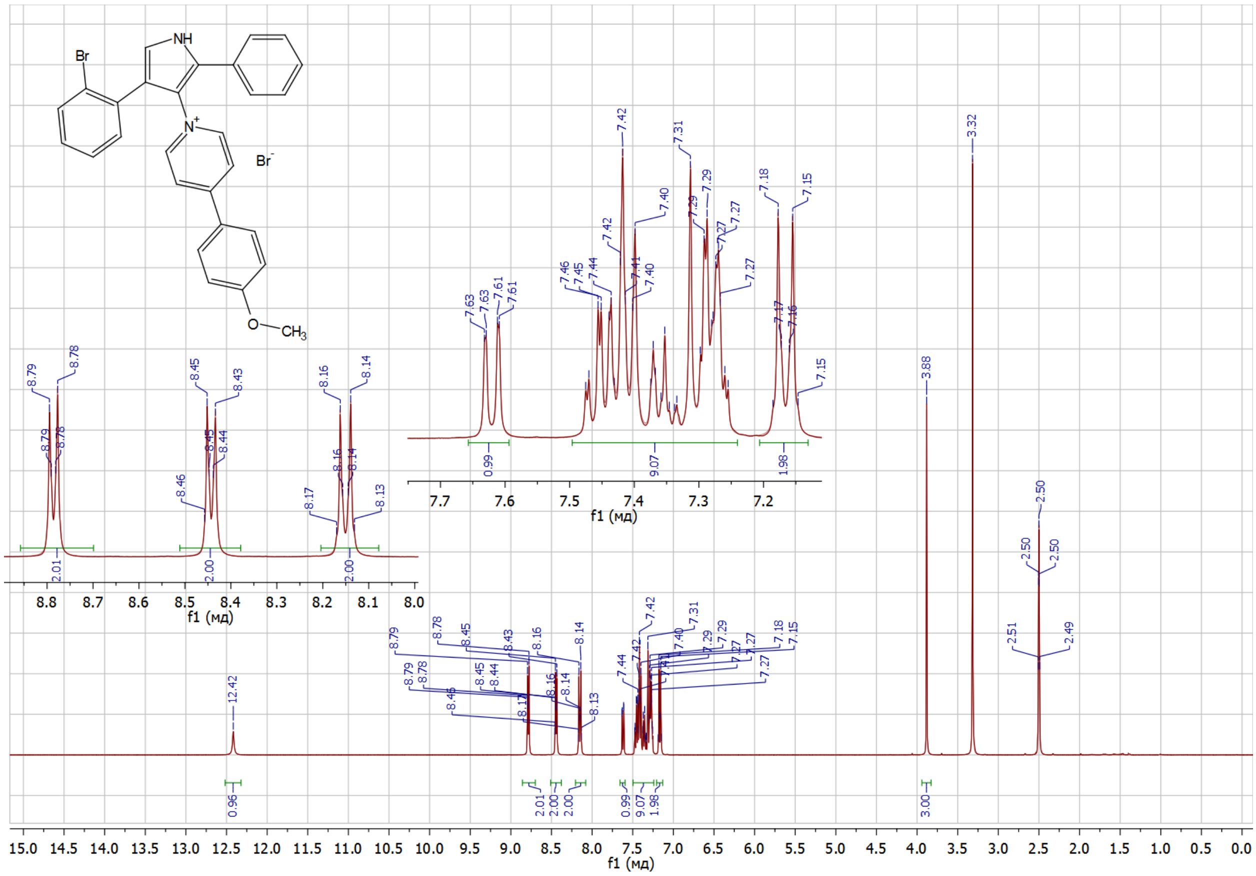

^{13}C{1H} NMR (101 MHz, DMSO-d_6) of 1-(4-(2-bromophenyl)-2-phenyl-1H-pyrrol-3-yl)-4-(4-methoxyphenyl)pyridin-1-ium bromide (**1r**)

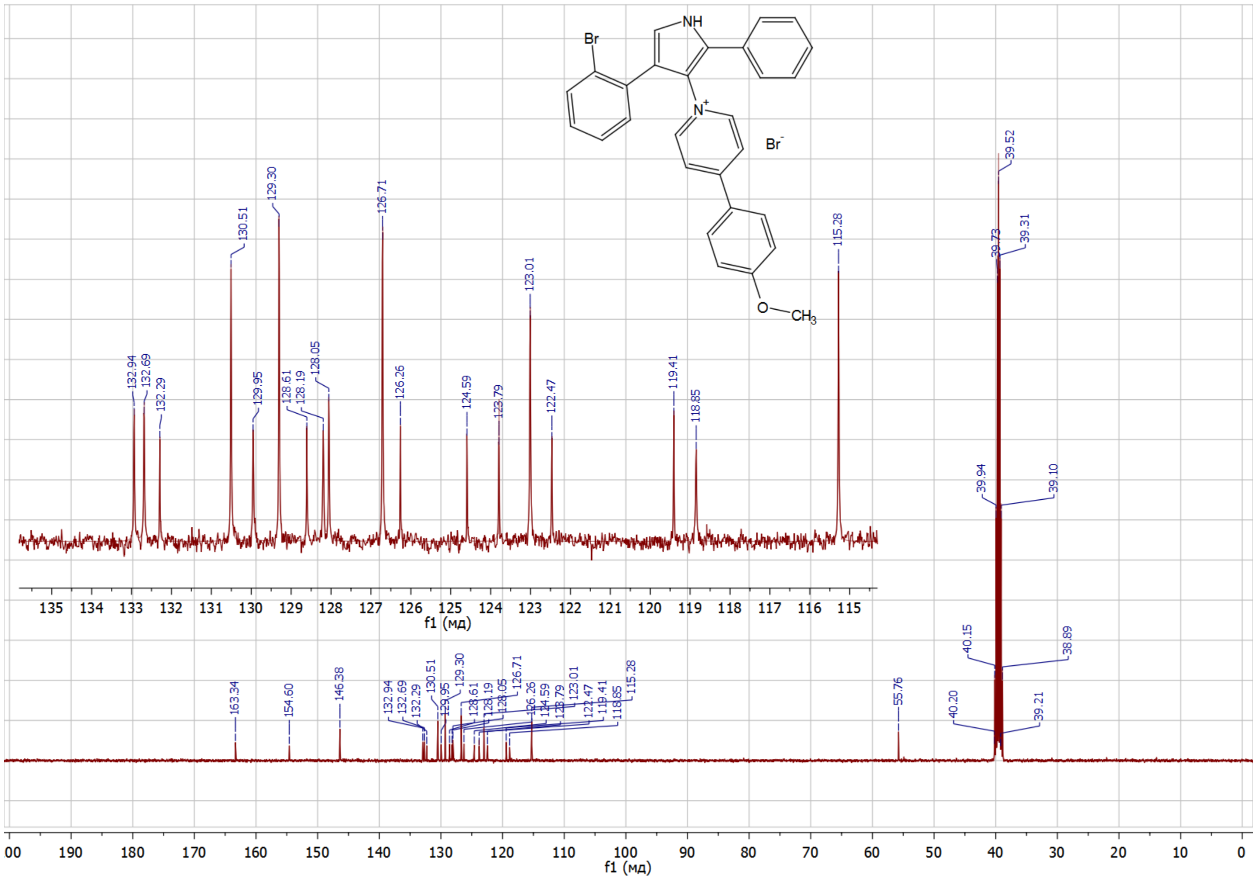

^{13}C DEPT135 NMR (101 MHz, DMSO-d_6) of 1-(4-(2-bromophenyl)-2-phenyl-1*H*-pyrrol-3-yl)-4-(4-methoxyphenyl)pyridin-1-ium bromide (**1r**)

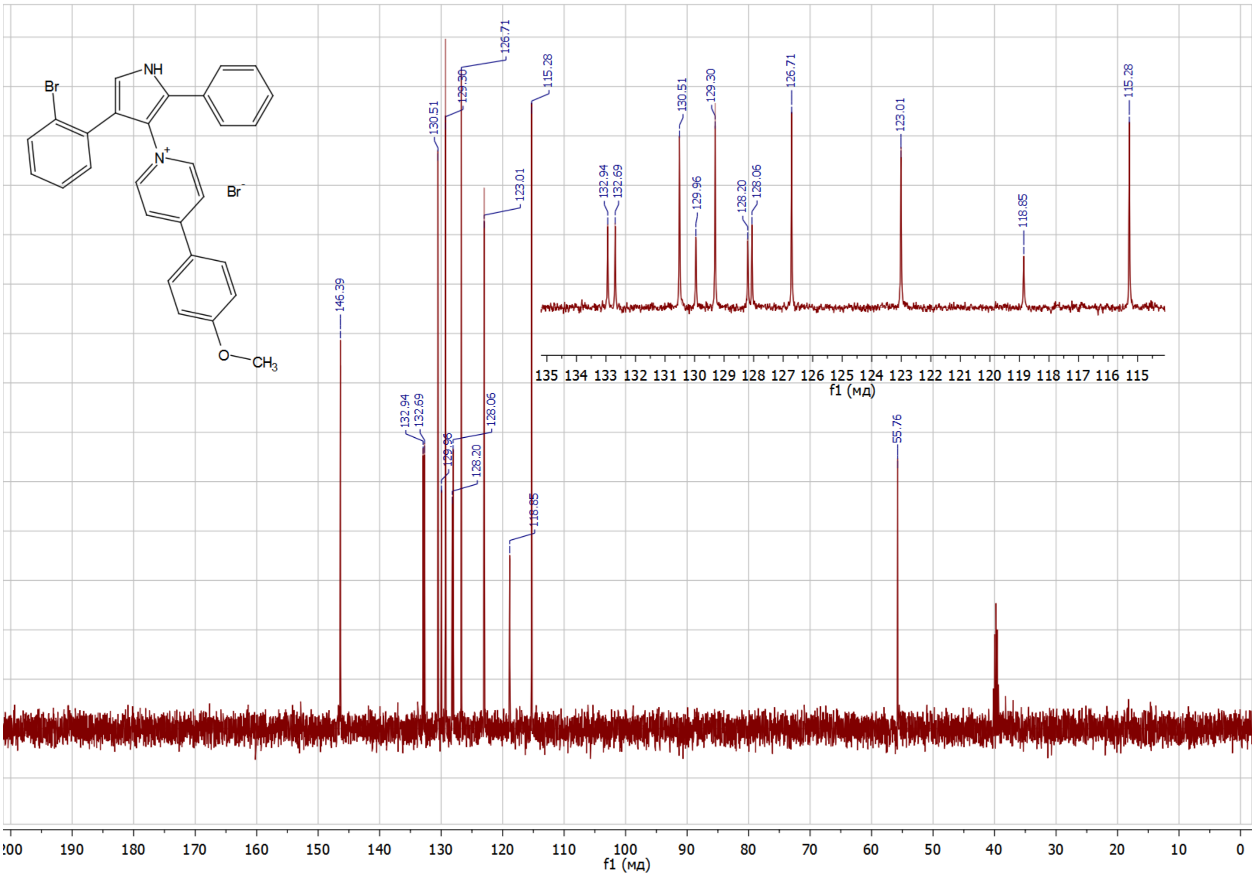

^{1}H NMR (400 MHz, DMSO-d_6) of 1-(4-(2-bromophenyl)-2-phenyl-1*H*-pyrrol-3-yl)-3,5-dimethylpyridin-1-ium bromide (**1s**)

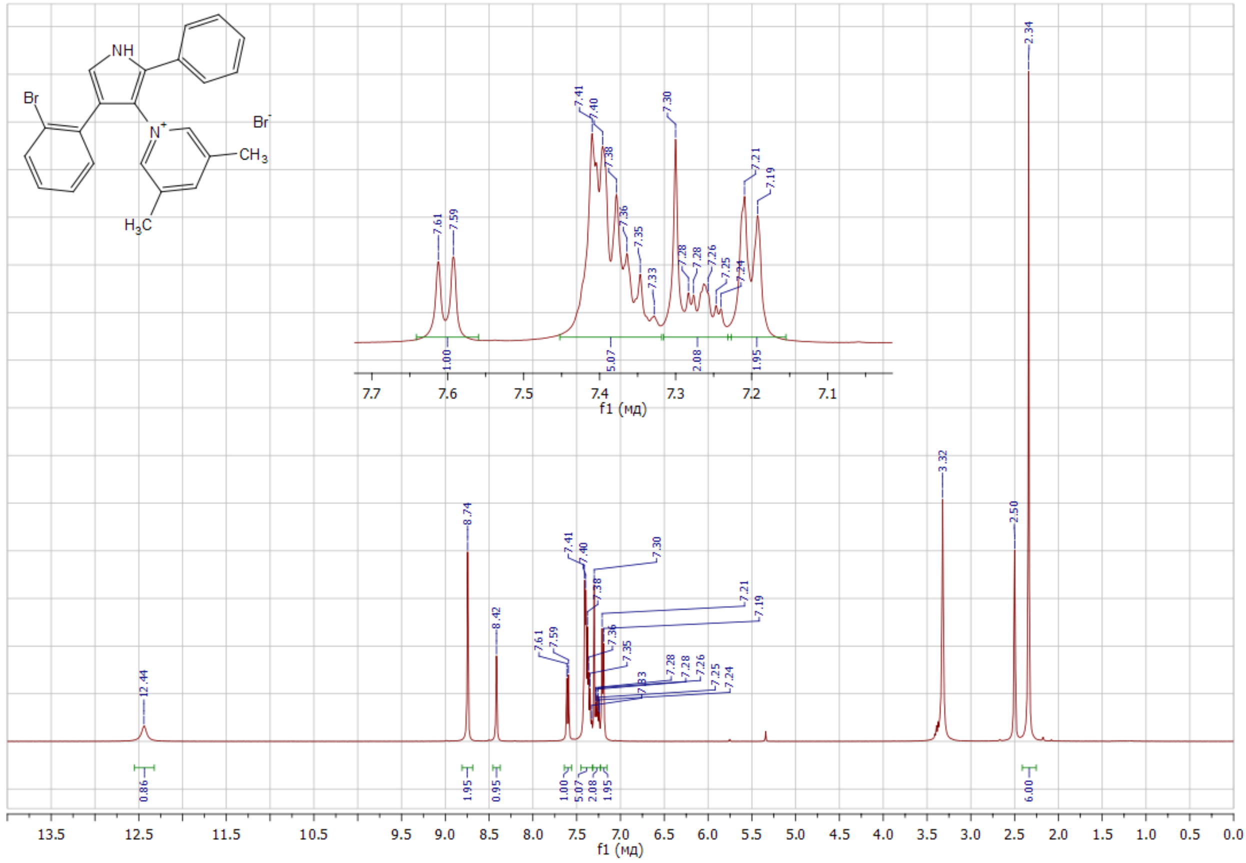

$^{13}C\{1H\}$ NMR (101 MHz, DMSO-d_6) of 1-(4-(2-bromophenyl)-2-phenyl-1*H*-pyrrol-3-yl)-3,5-dimethylpyridin-1-ium bromide (**1s**)

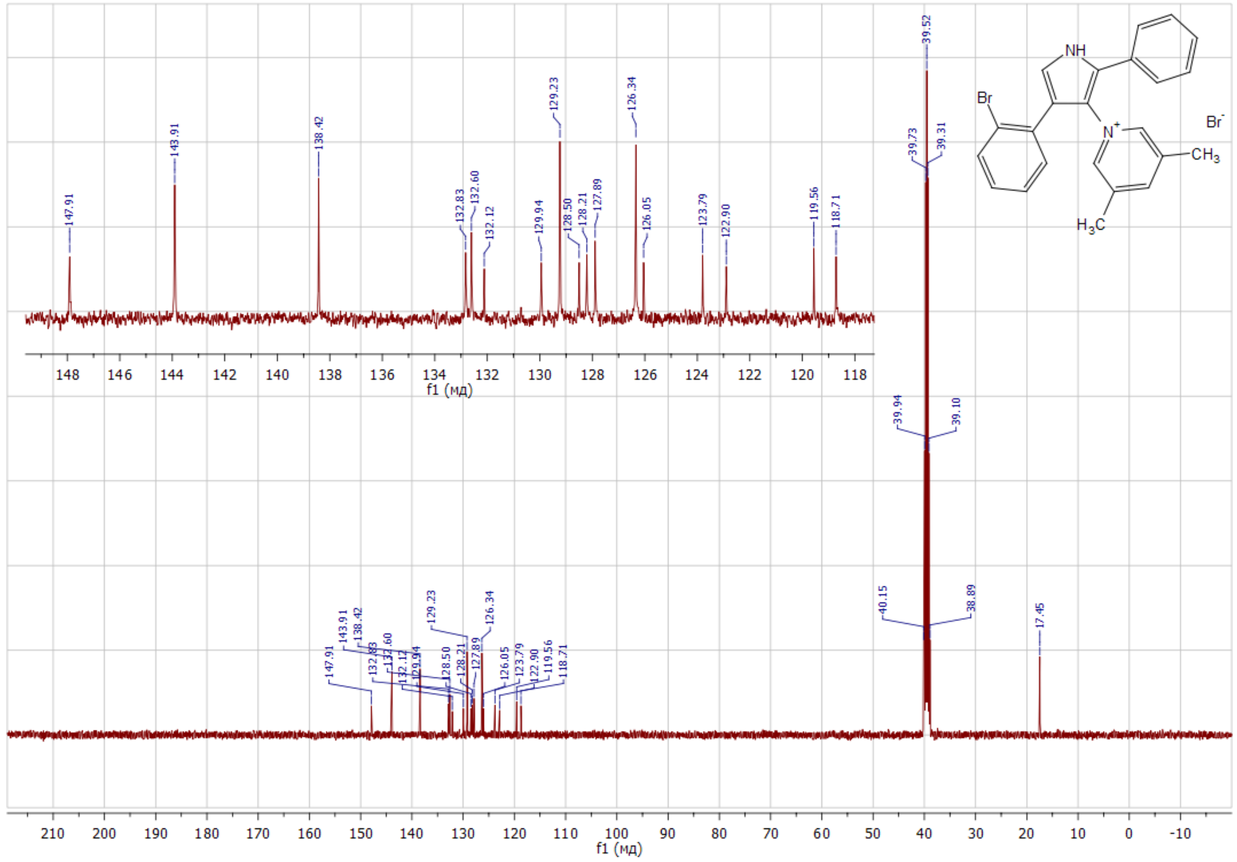

^{13}C DEPT135 NMR (101 MHz, DMSO-d_6) of 1-(4-(2-bromophenyl)-2-phenyl-1*H*-pyrrol-3-yl)-3,5-dimethylpyridin-1-ium bromide (**1s**)

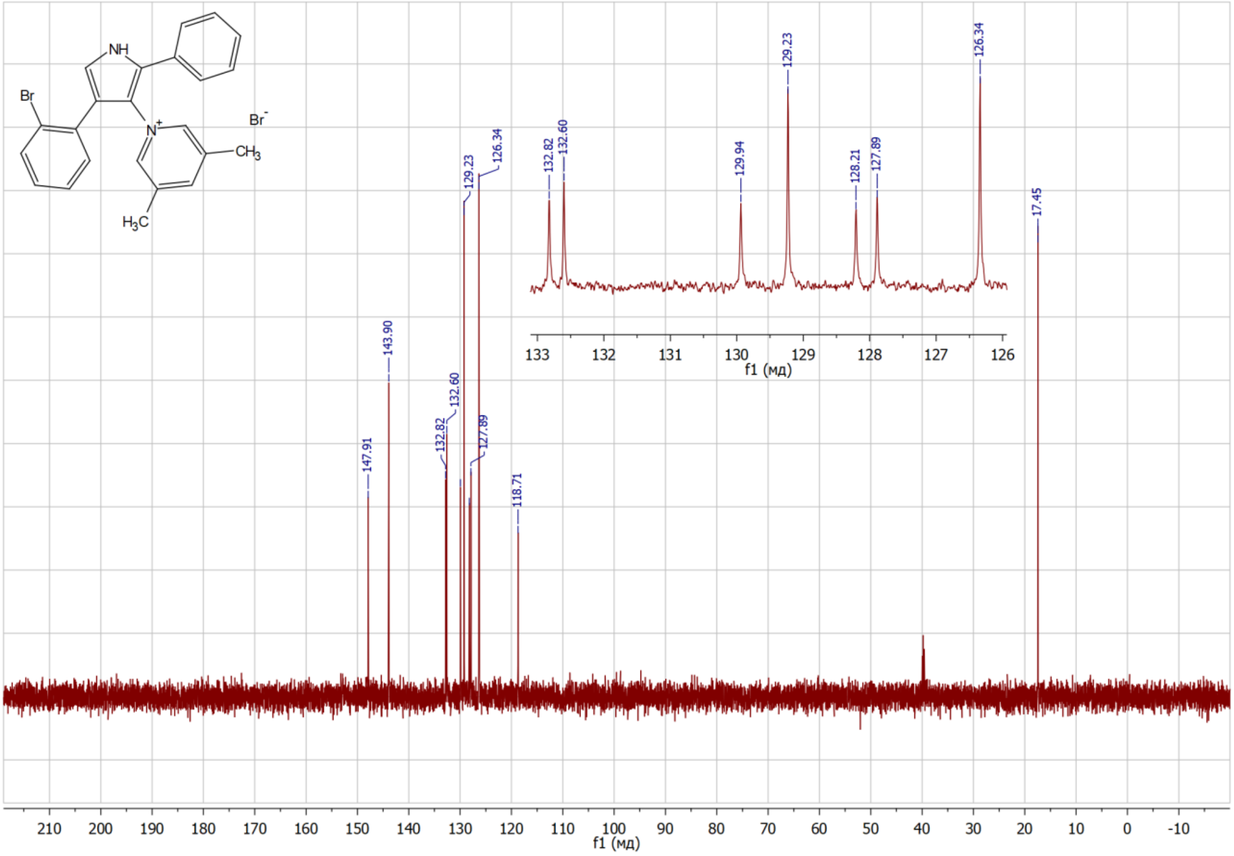

^{1}H NMR (400 MHz, DMSO-d_6) of 2-(4-(2-bromophenyl)-2-phenyl-1*H*-pyrrol-3-yl)isoquinolin-2-iumbromide (**1t**)

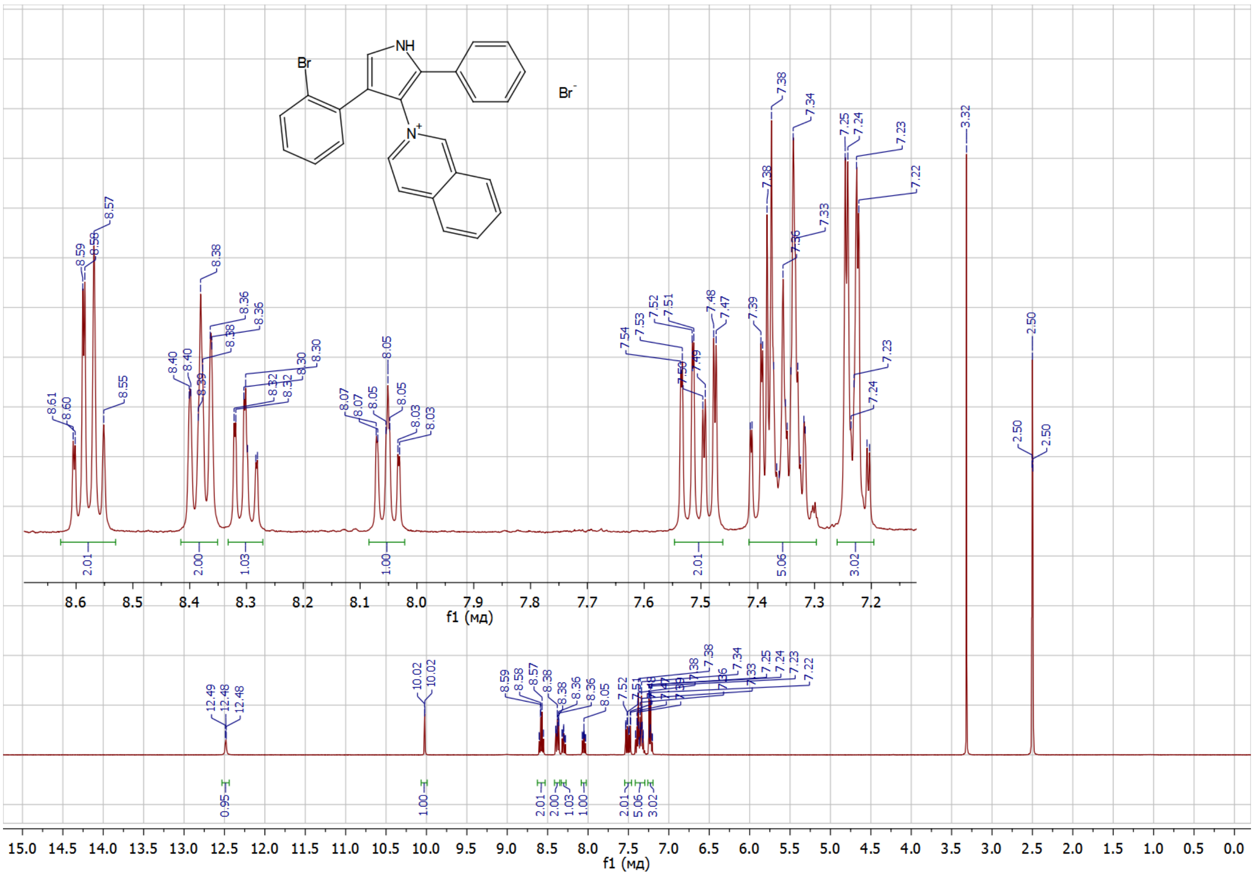

$^{13}C\{^{1}H\}$ NMR (101 MHz, DMSO-d_6) of 2-(4-(2-bromophenyl)-2-phenyl-1*H*-pyrrol-3-yl)isoquinolin-2-iumbromide (**1t**)

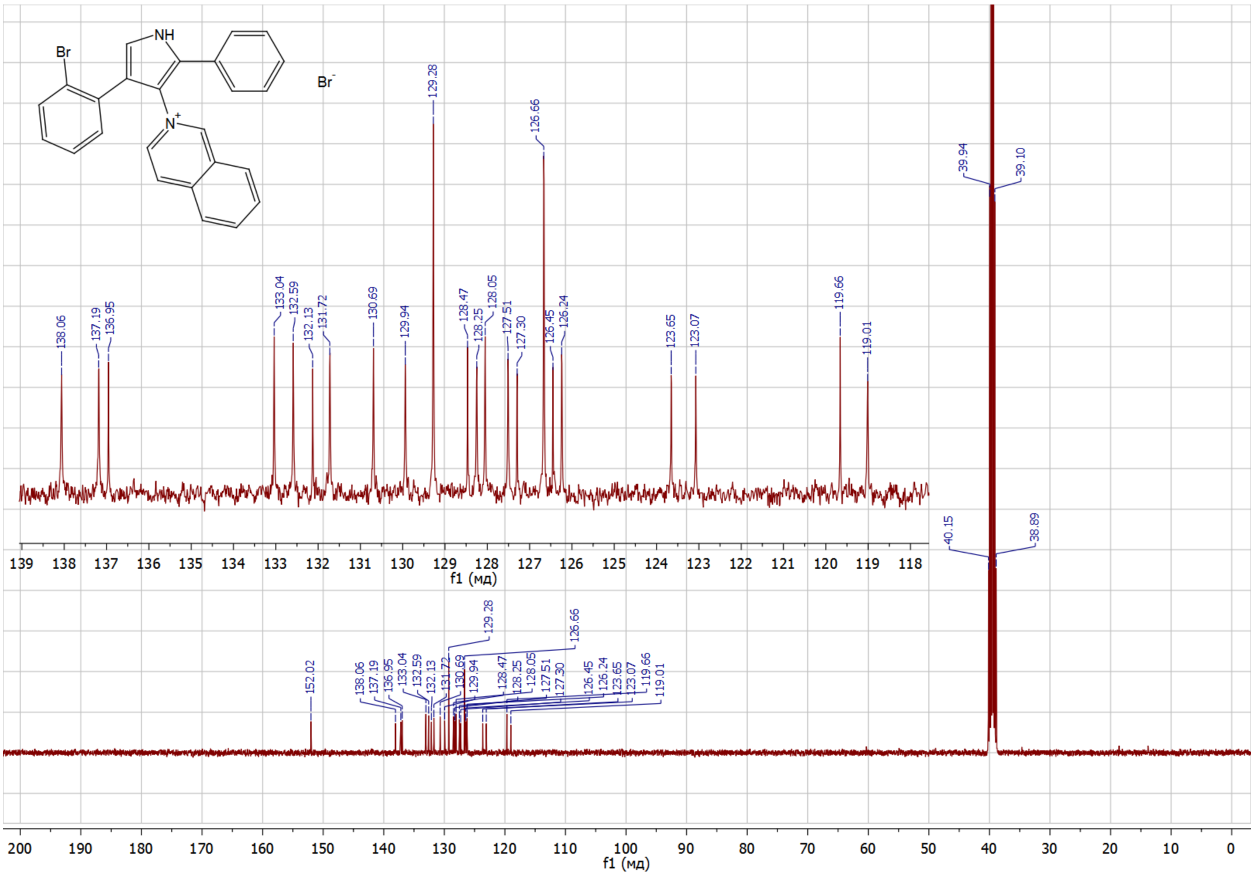

^{13}C DEPT135 NMR (101 MHz, DMSO-d_6) of 2-(4-(2-bromophenyl)-2-phenyl-1*H*-pyrrol-3-yl)isoquinolin-2-iumbromide (**1t**)

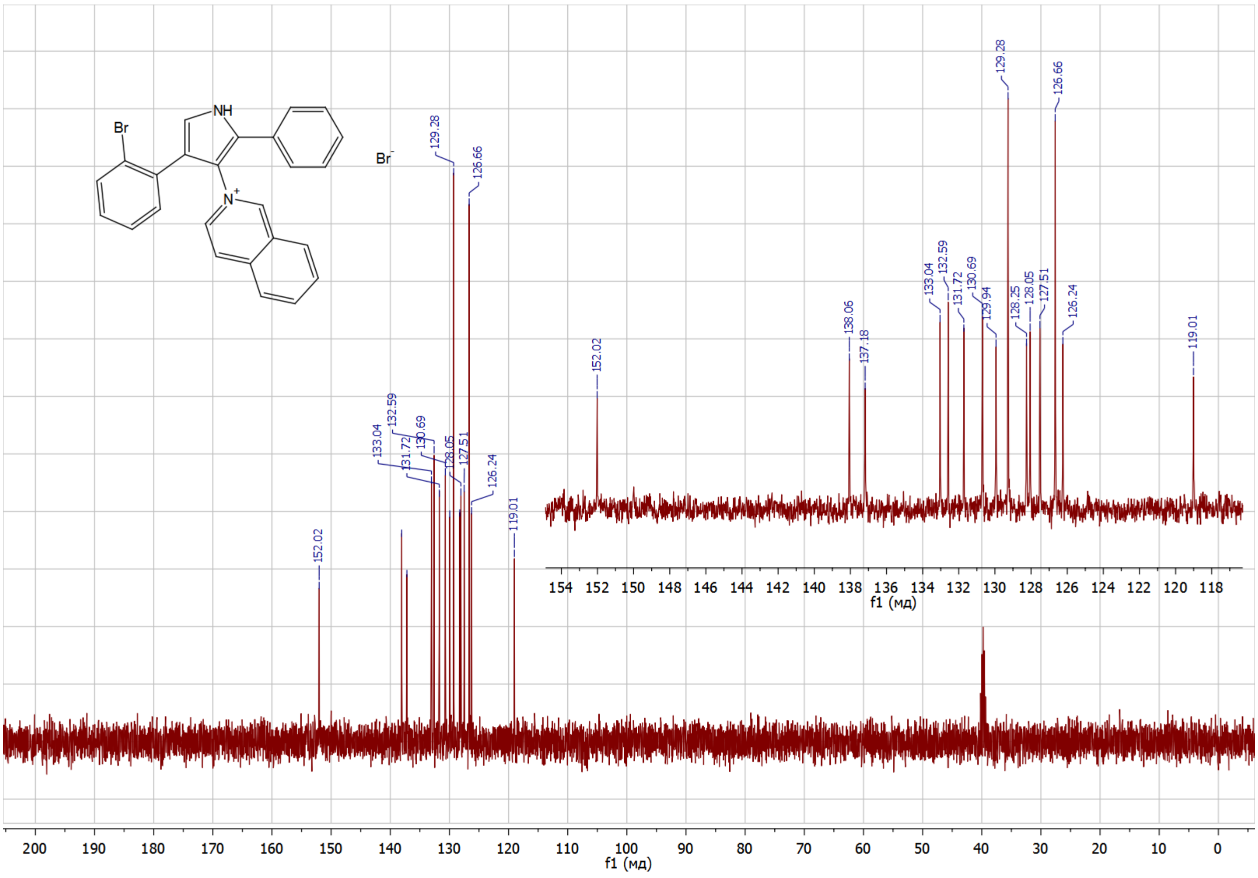

^{1}H NMR (400 MHz, DMSO-d_6) of 1-(4-(2-bromophenyl)-2-phenyl-1*H*-pyrrol-3-yl)-4-(methoxycarbonyl)pyridin-1-ium bromide (**1u**)

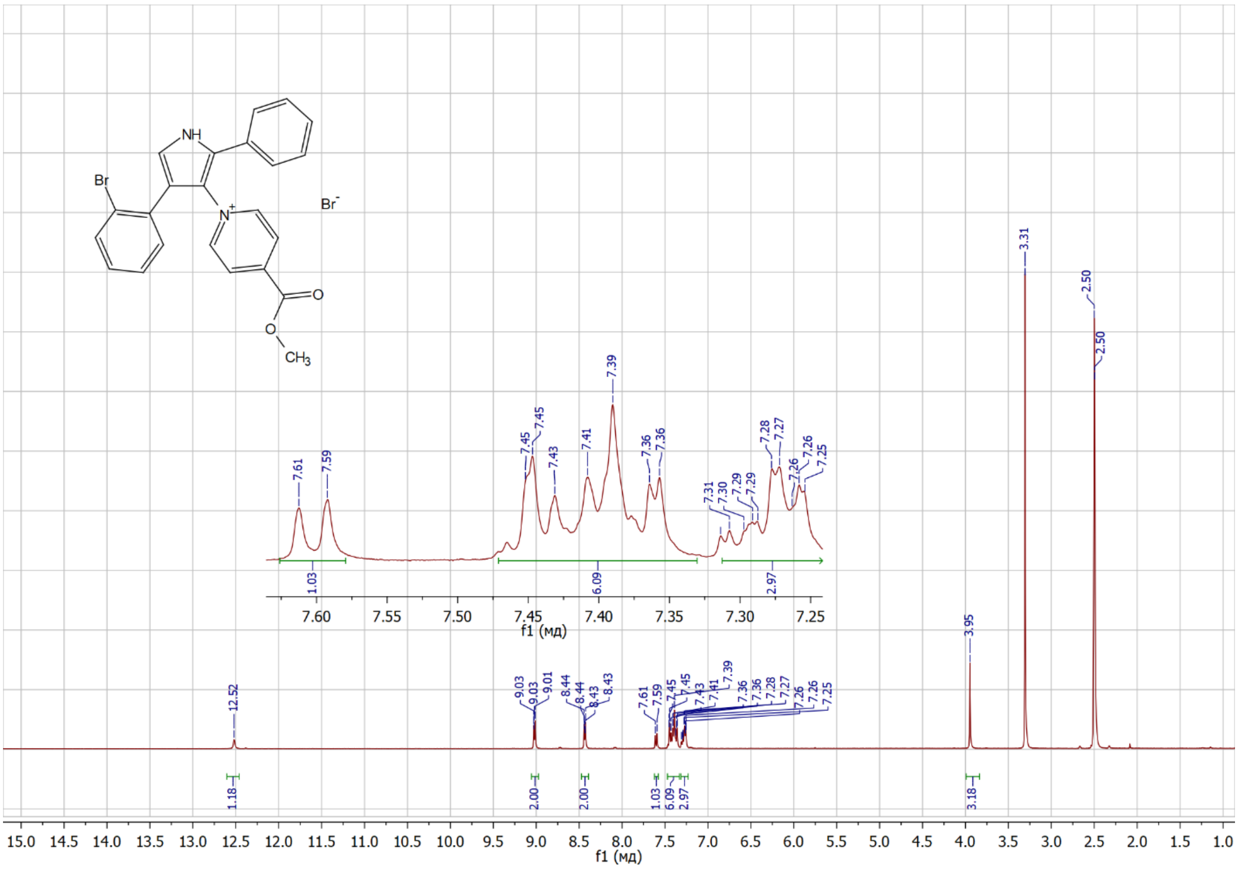

^{13}C{1H} NMR (101 MHz, DMSO-d_6) of 1-(4-(2-bromophenyl)-2-phenyl-1*H*-pyrrol-3-yl)-4-(methoxycarbonyl)pyridin-1-ium bromide (**1u**)

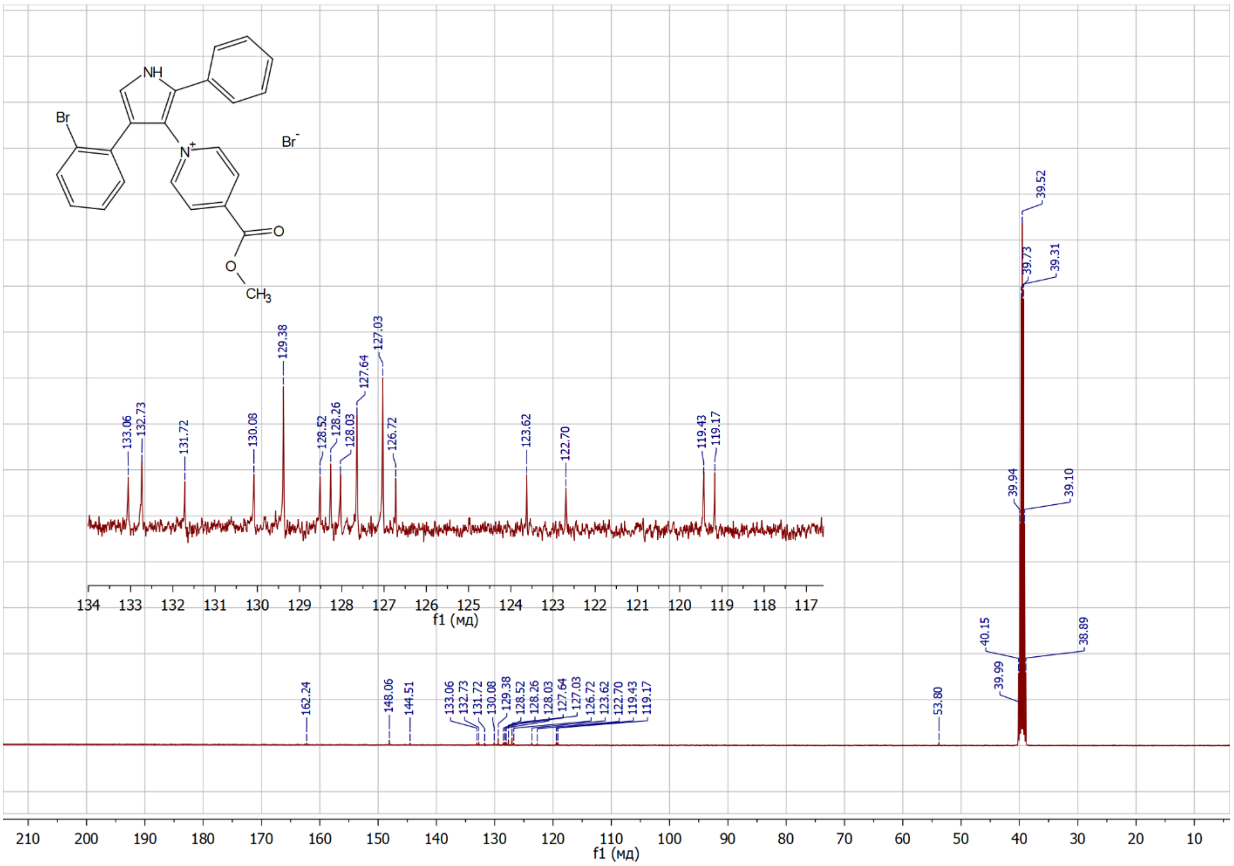

^{13}C DEPT135 NMR (101 MHz, DMSO-d_6) of 1-(4-(2-bromophenyl)-2-phenyl-1*H*-pyrrol-3-yl)-4-(methoxycarbonyl)pyridin-1-ium bromide (**1u**)

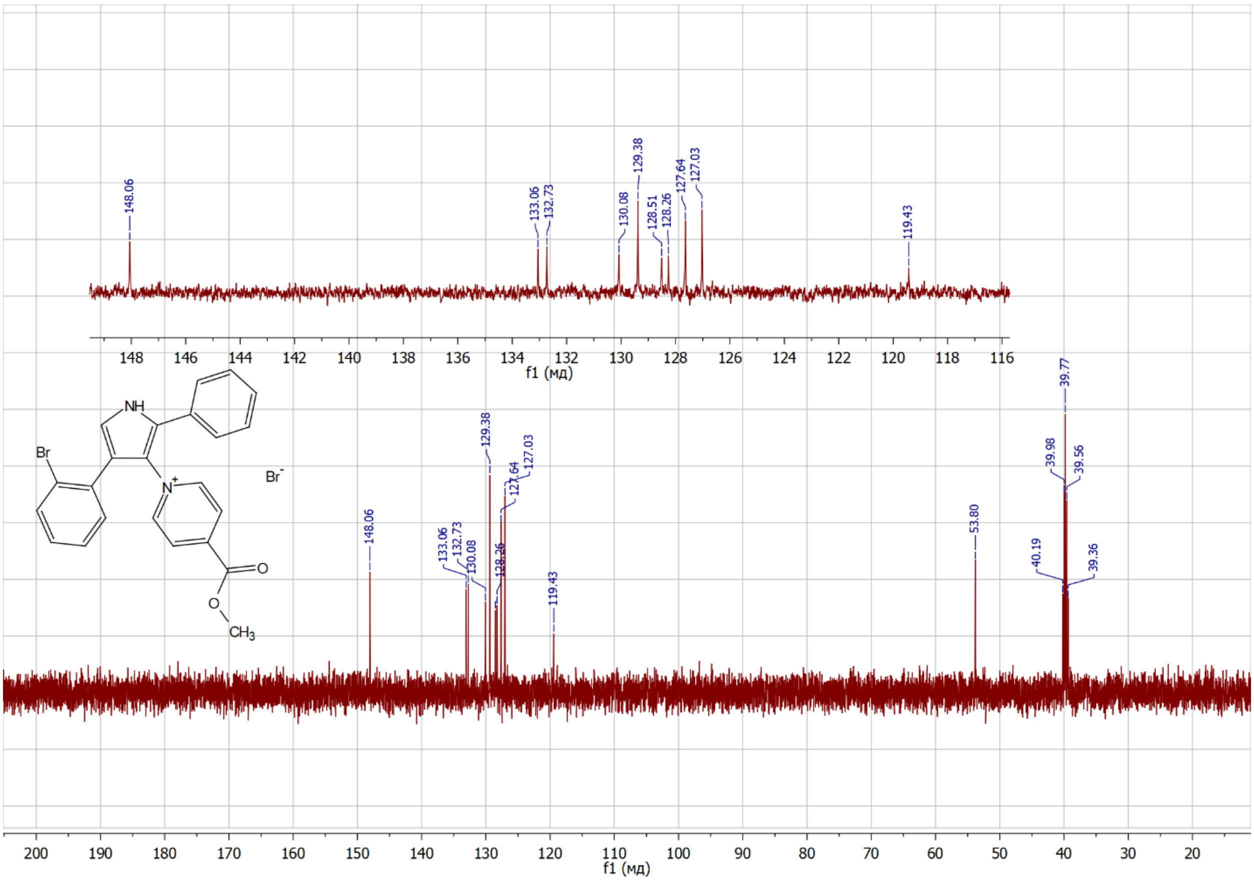

^{1}H NMR (400 MHz, DMSO-d_6) of 1-(4-(2-bromophenyl)-2-phenyl-1*H*-pyrrol-3-yl)-4-cyanopyridin-1-ium bromide (**1v**)

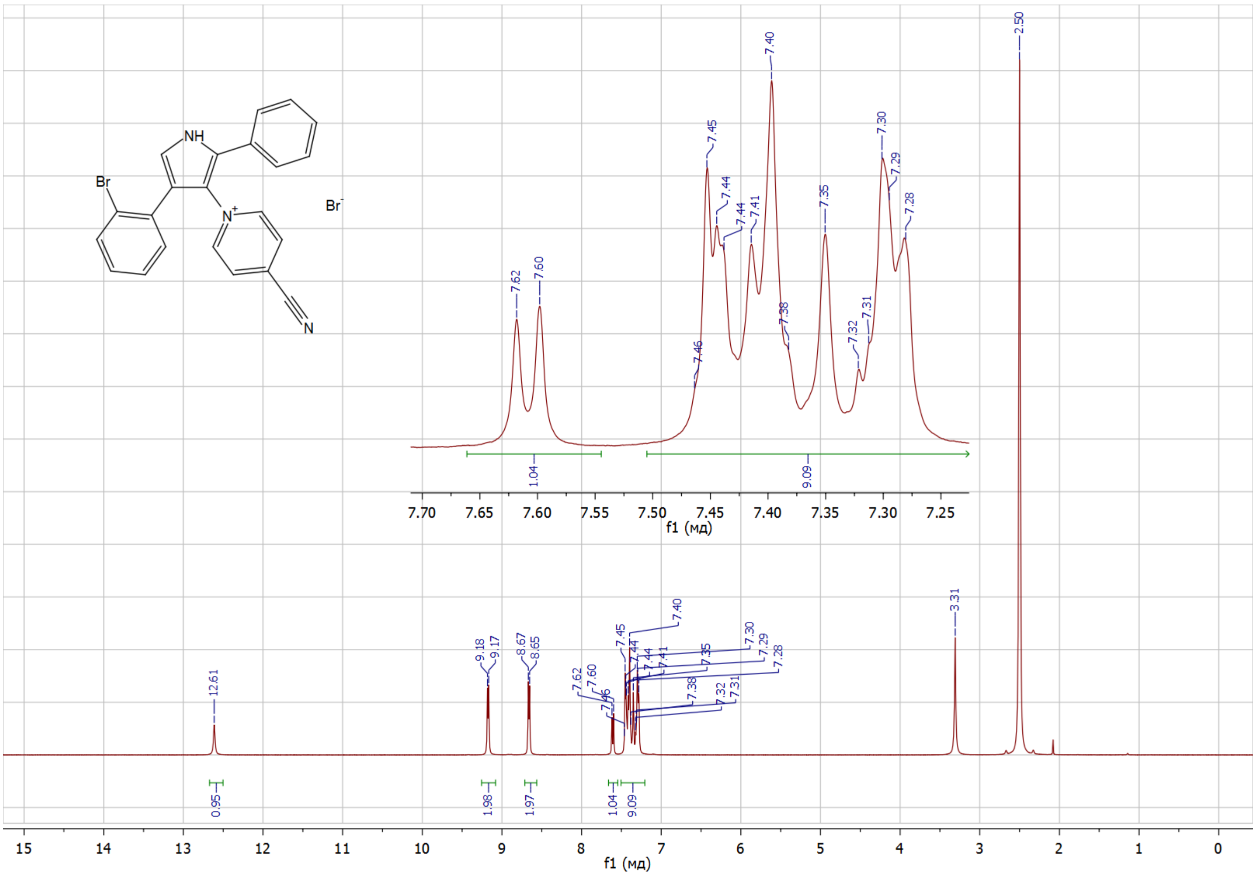

^{13}C{1H} NMR (101 MHz, DMSO-d_6) of 1-(4-(2-bromophenyl)-2-phenyl-1*H*-pyrrol-3-yl)-4-cyanopyridin-1-ium bromide (**1v**)

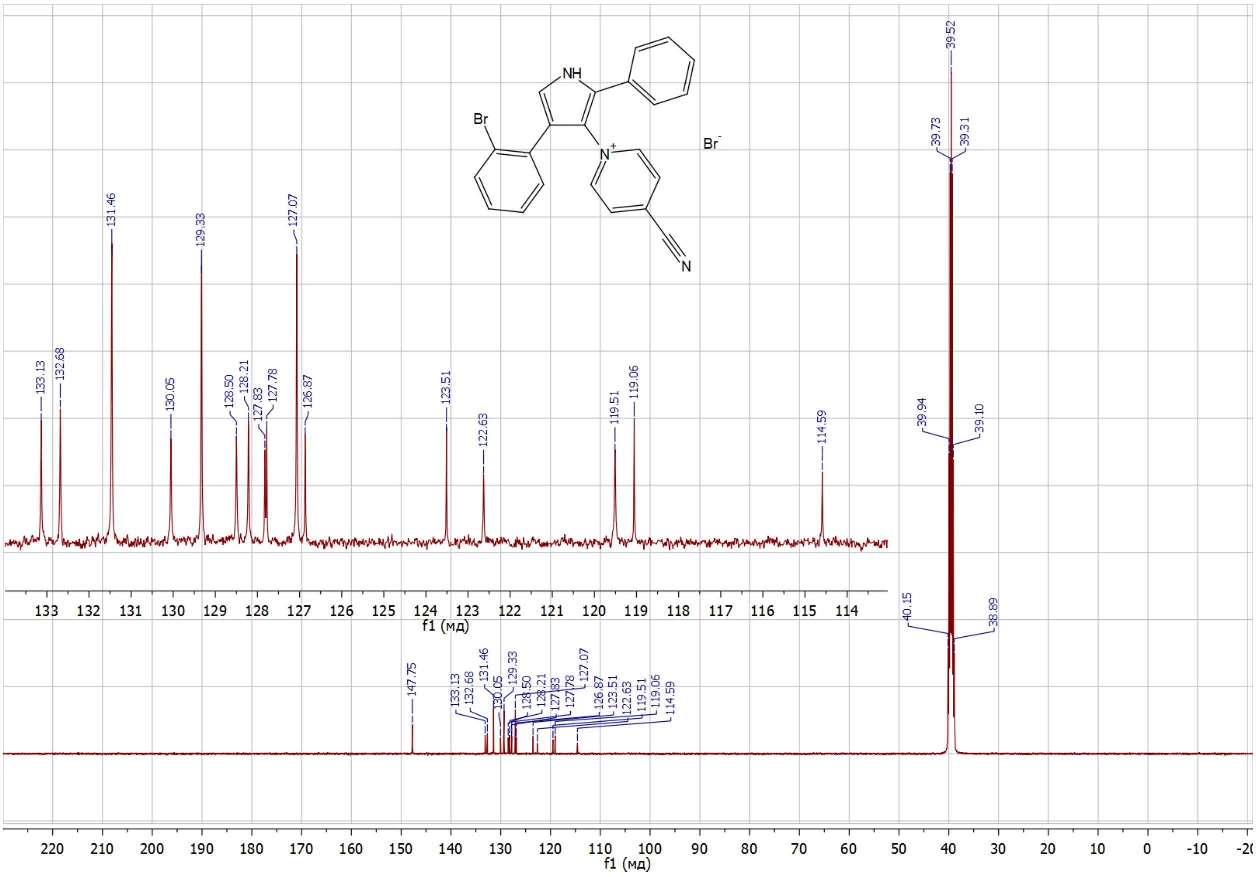

^{13}C DEPT135 NMR (101 MHz, DMSO-d_6) of 1-(4-(2-bromophenyl)-2-phenyl-1*H*-pyrrol-3-yl)-4-cyanopyridin-1-ium bromide (**1v**)

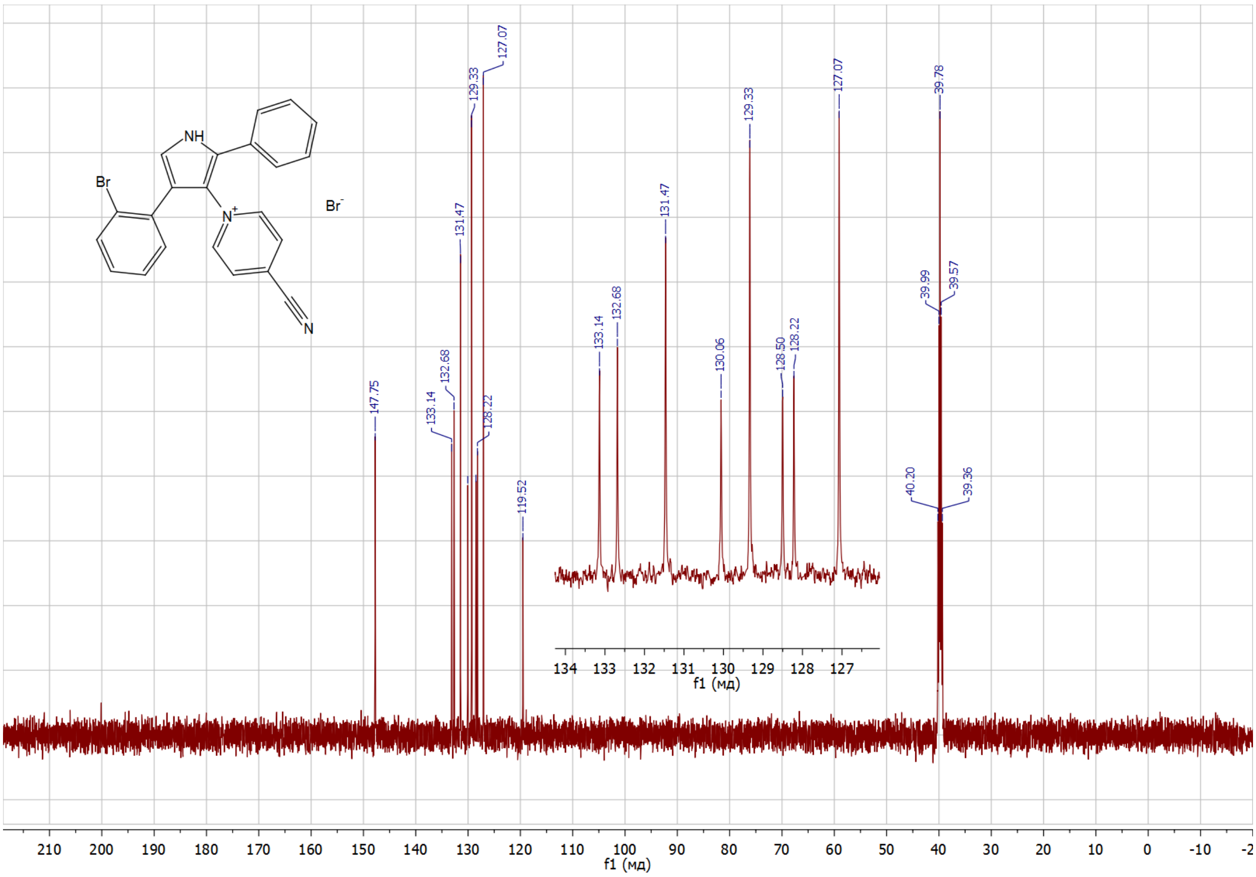

^{1}H NMR (400 MHz, DMSO-d_6) of 4-benzoyl-1-(4-(2-bromophenyl)-2-phenyl-1*H*-pyrrol-3-yl)pyridin-1-ium bromide (**1w**)

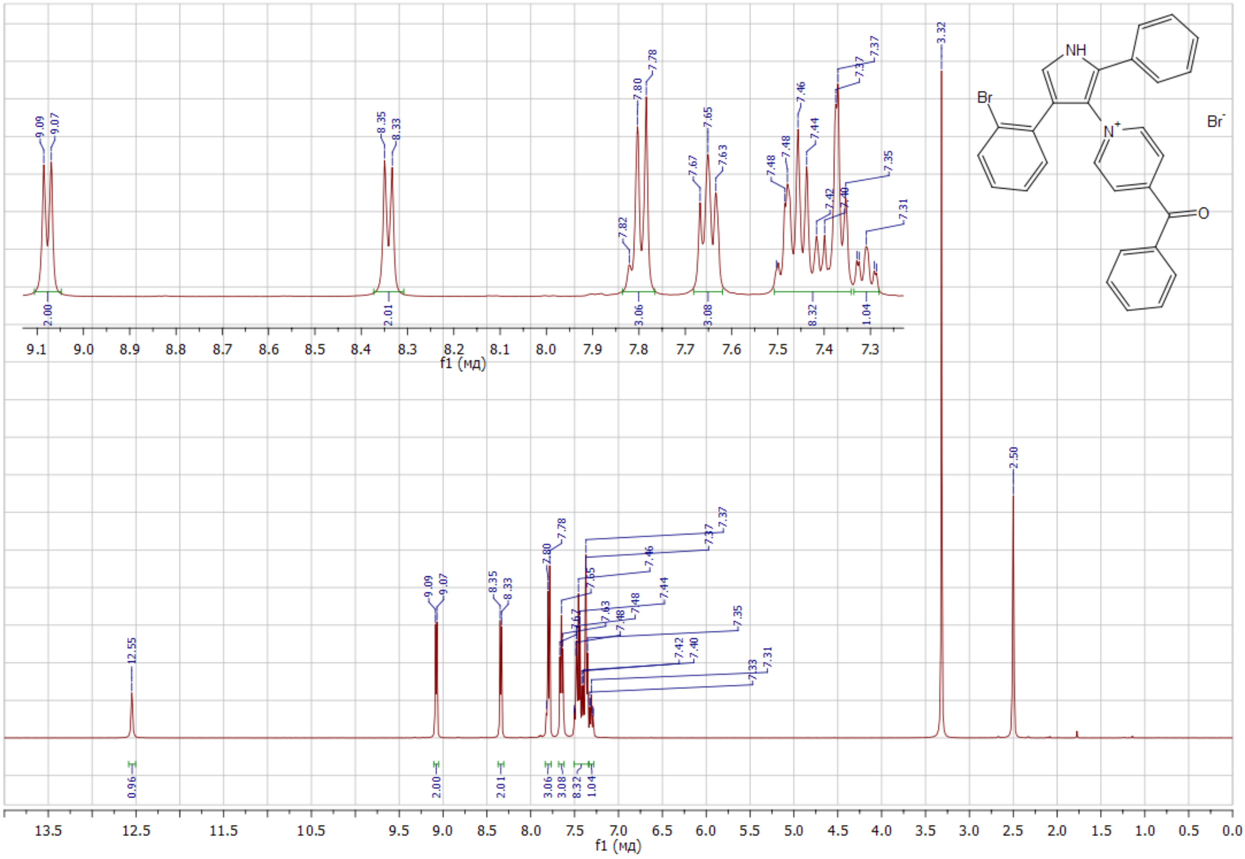

^{13}C{1H} NMR (101 MHz, DMSO-d_6) of 4-benzoyl-1-(4-(2-bromophenyl)-2-phenyl-1*H*-pyrrol-3-yl)pyridin-1-ium bromide (**1w**)

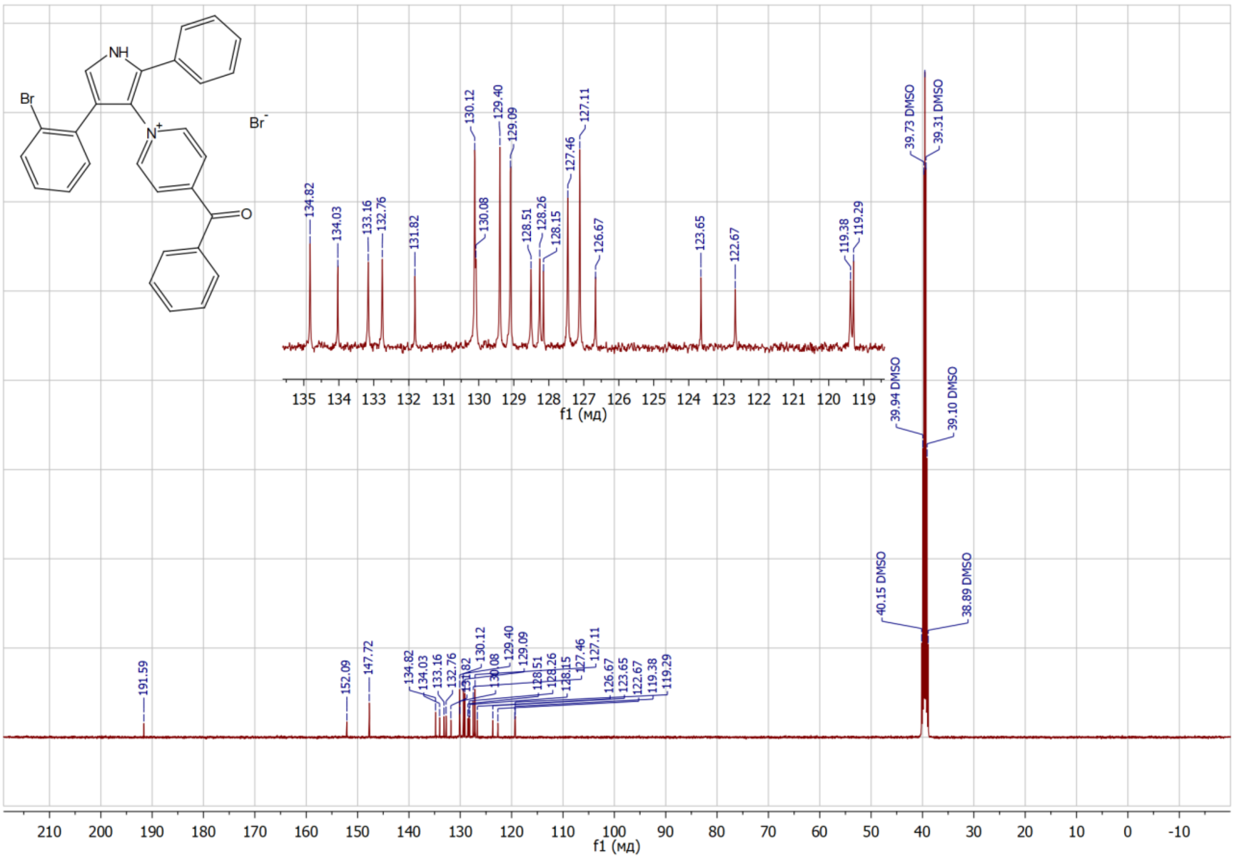

^{13}C DEPT135 NMR (101 MHz, DMSO-d_6) of 4-benzoyl-1-(4-(2-bromophenyl)-2-phenyl-1*H*-pyrrol-3-yl)pyridin-1-ium bromide (**1w**)

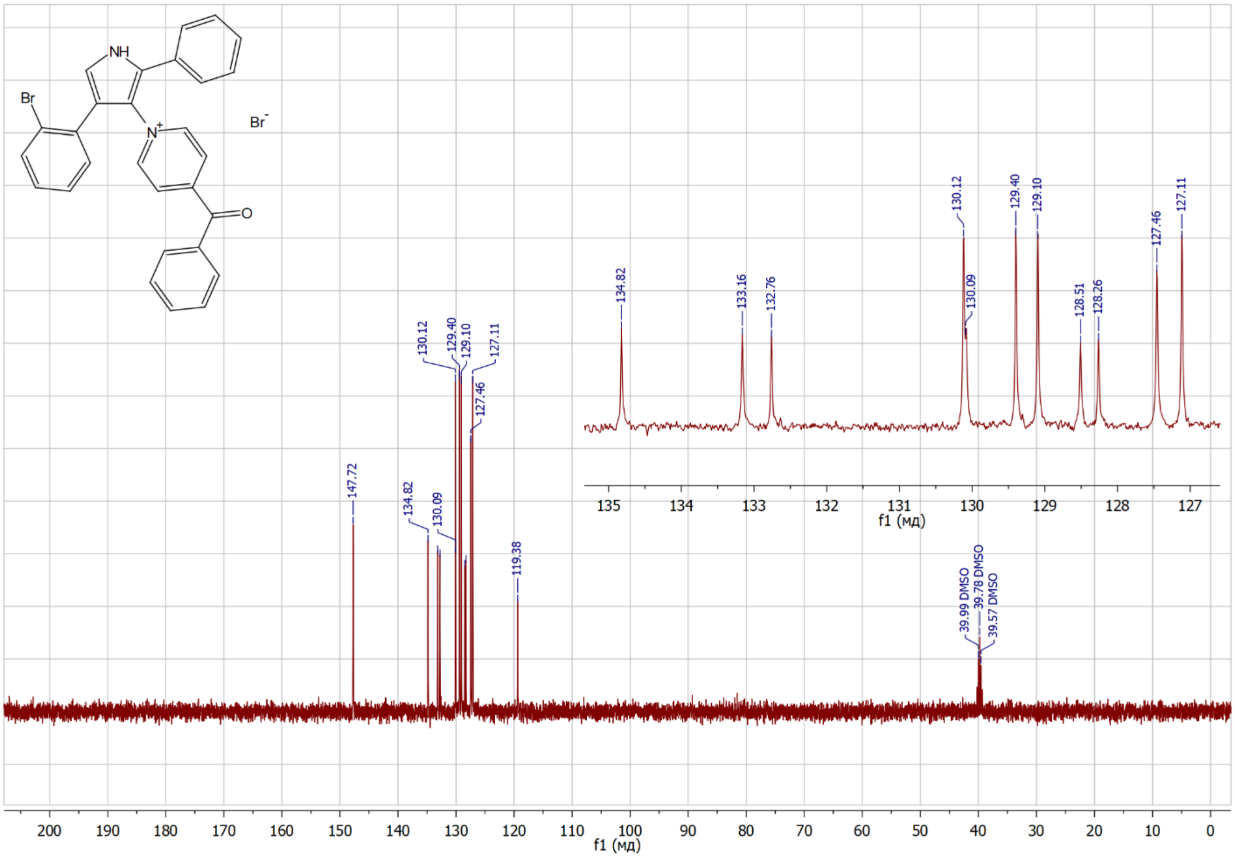

^1H NMR (400 MHz, DMSO-d_6) of 1-(4-(2-iodophenyl)-2-phenyl-1*H*-pyrrol-3-yl)pyridin-1-ium bromide (**2**)

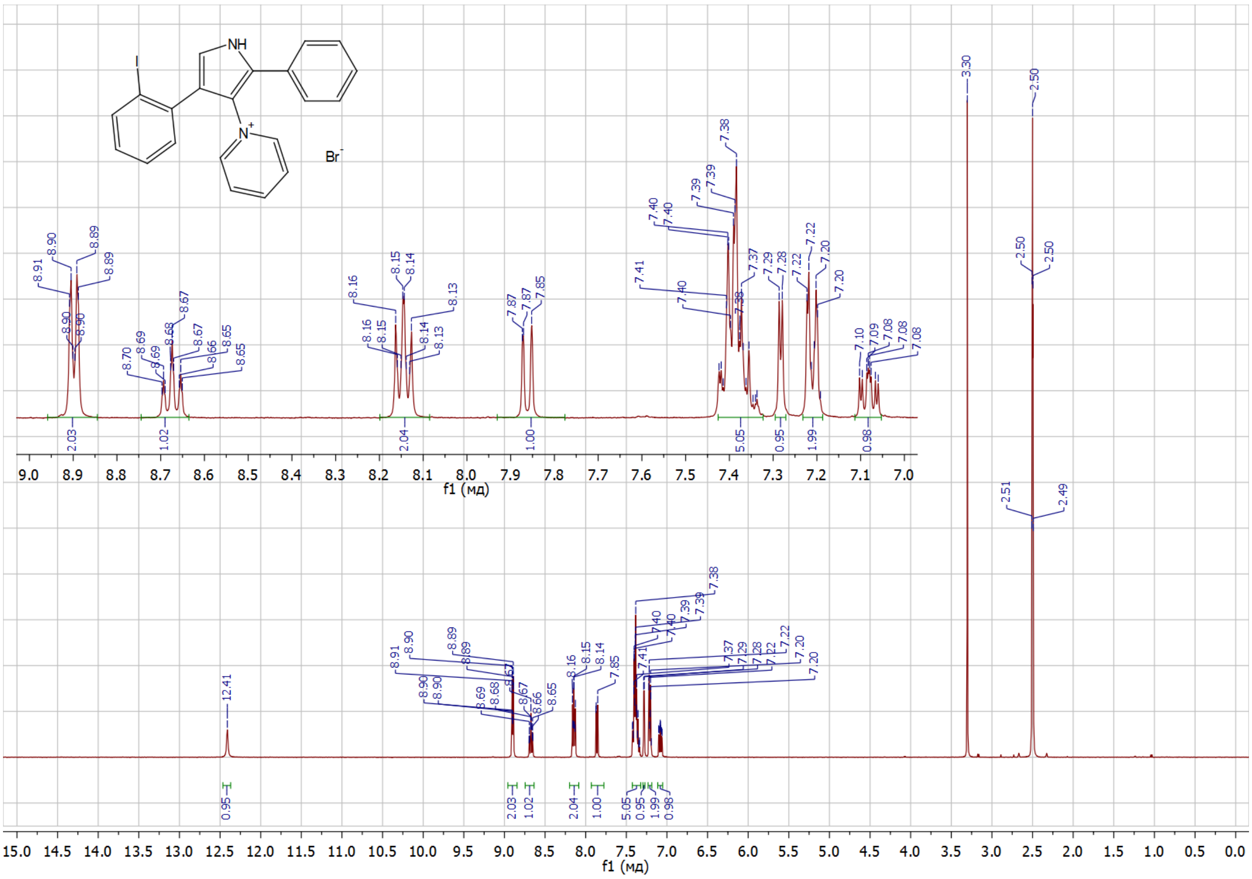

$^{13}C\{1H\}$ NMR (101 MHz, DMSO-d_6) of 1-(4-(2-iodophenyl)-2-phenyl-1*H*-pyrrol-3-yl)pyridin-1-ium bromide (**2**)

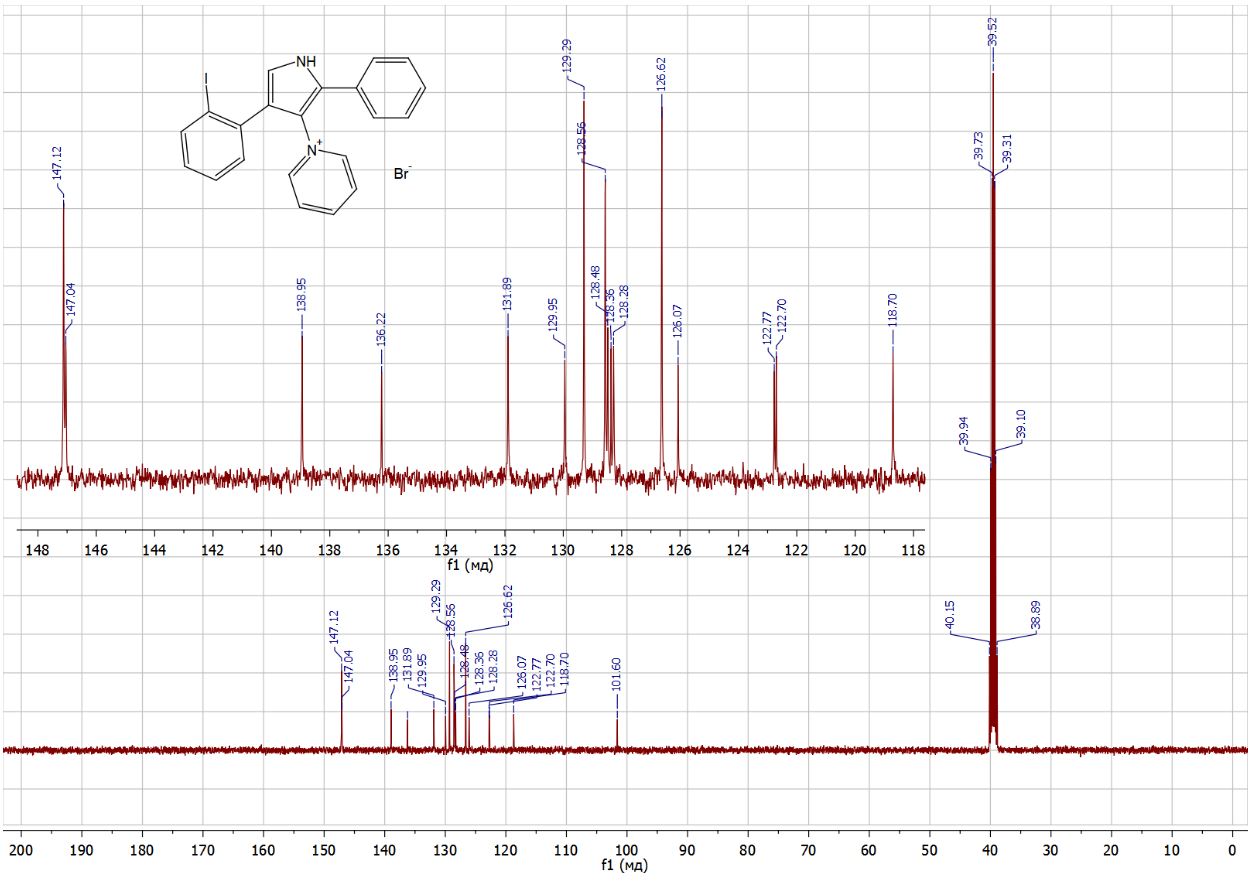

^{13}C DEPT135 NMR (101 MHz, DMSO-d_6) of 1-(4-(2-iodophenyl)-2-phenyl-1*H*-pyrrol-3-yl)pyridin-1-ium bromide (**2**)

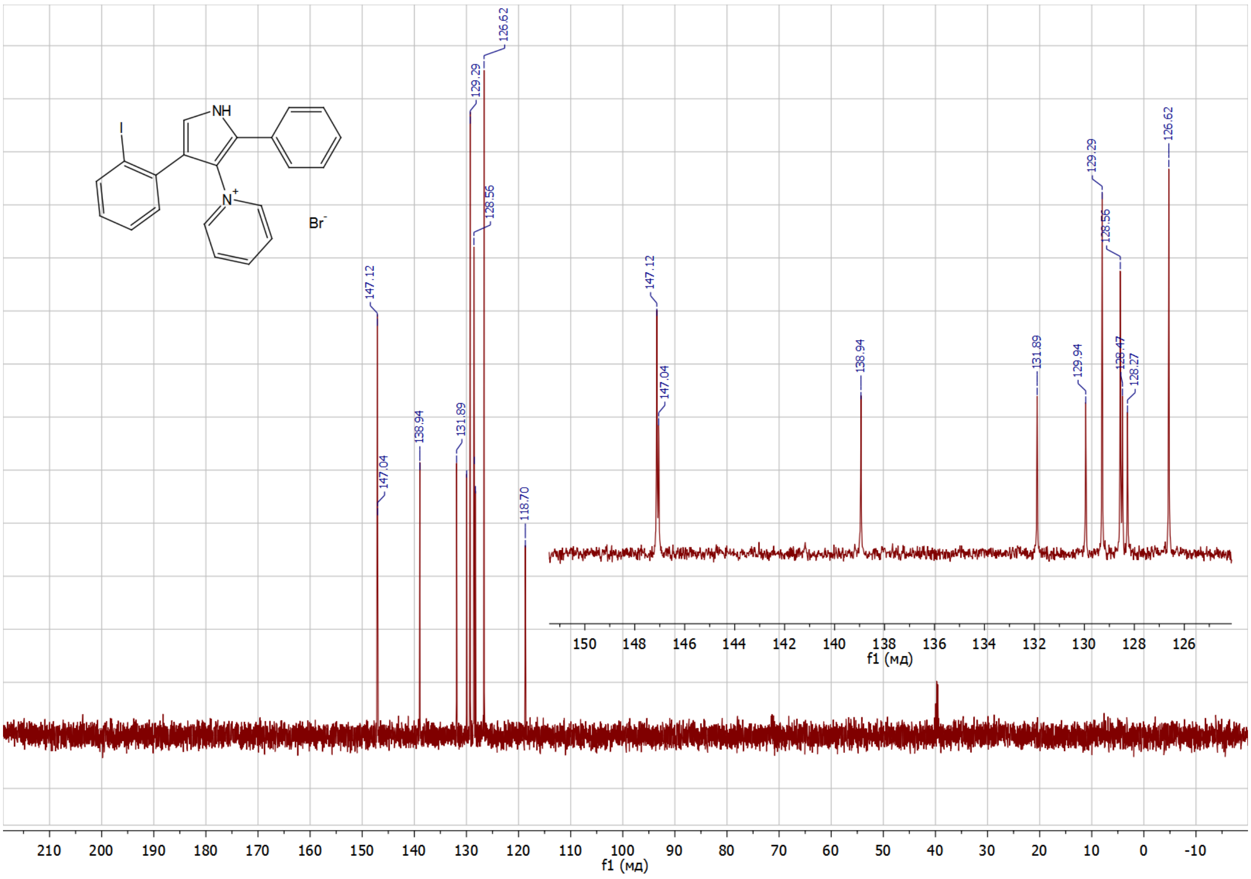

^{1}H NMR (400 MHz, DMSO-d_6) of 1-(1-benzyl-4-(2-iodophenyl)-2-phenyl-1*H*-pyrrol-3-yl)pyridin-1-ium bromide (**N-Bn-2**)

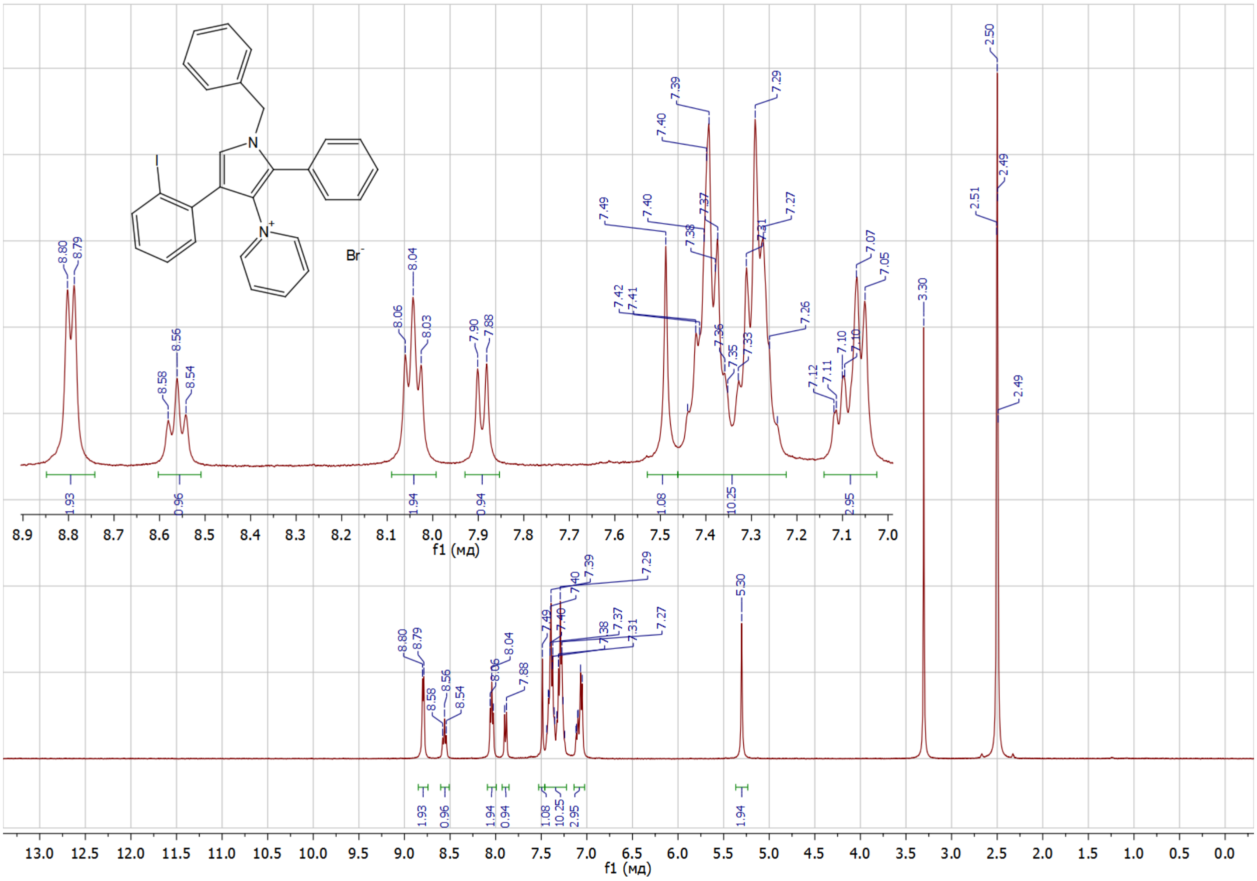

$^{13}C\{^{1}H\}$ NMR (101 MHz, DMSO-d_6) of 1-(1-benzyl-4-(2-iodophenyl)-2-phenyl-1*H*-pyrrol-3-yl)pyridin-1-ium bromide (**N-Bn-2**)

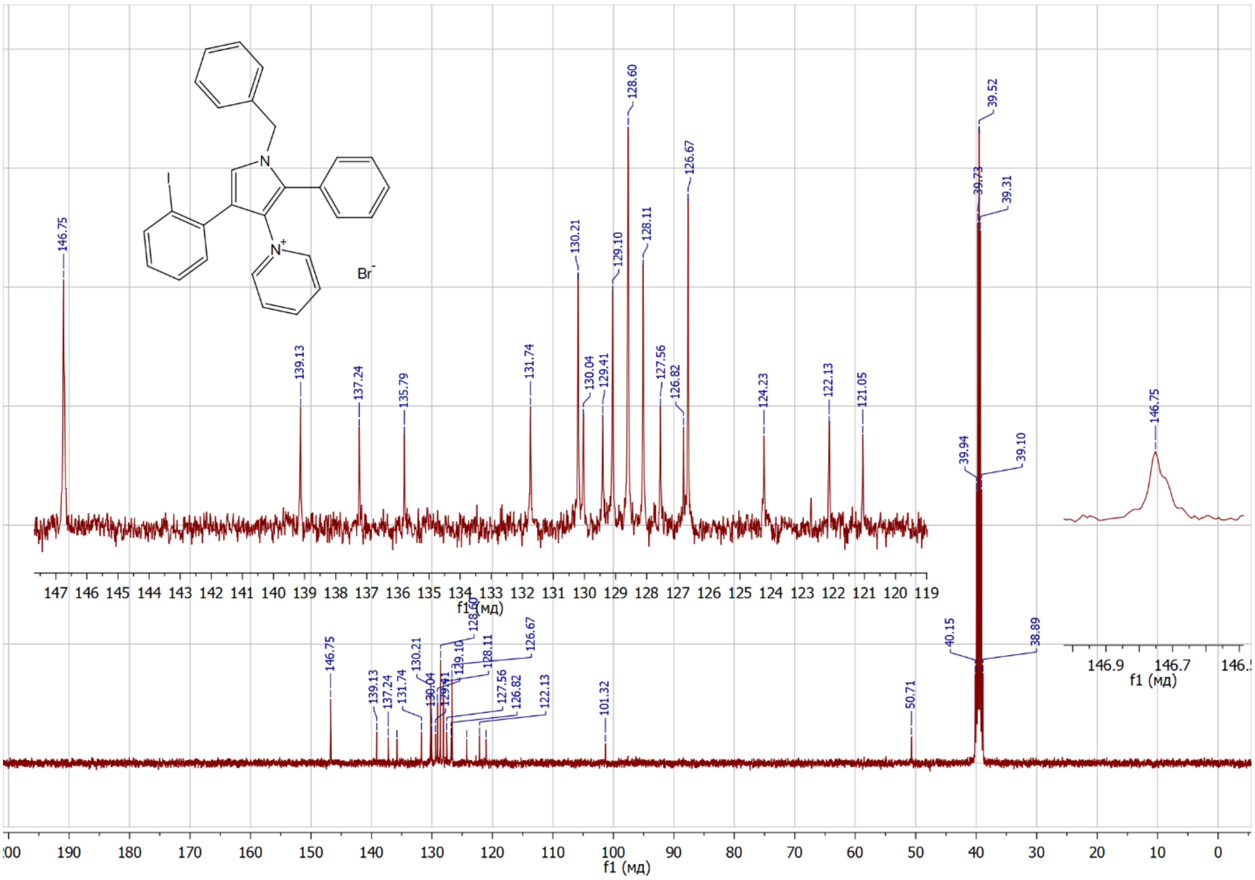

^{13}C DEPT135 NMR (101 MHz, DMSO-d_6) of 1-(1-benzyl-4-(2-iodophenyl)-2-phenyl-1*H*-pyrrol-3-yl)pyridin-1-ium bromide (**N-Bn-2**)

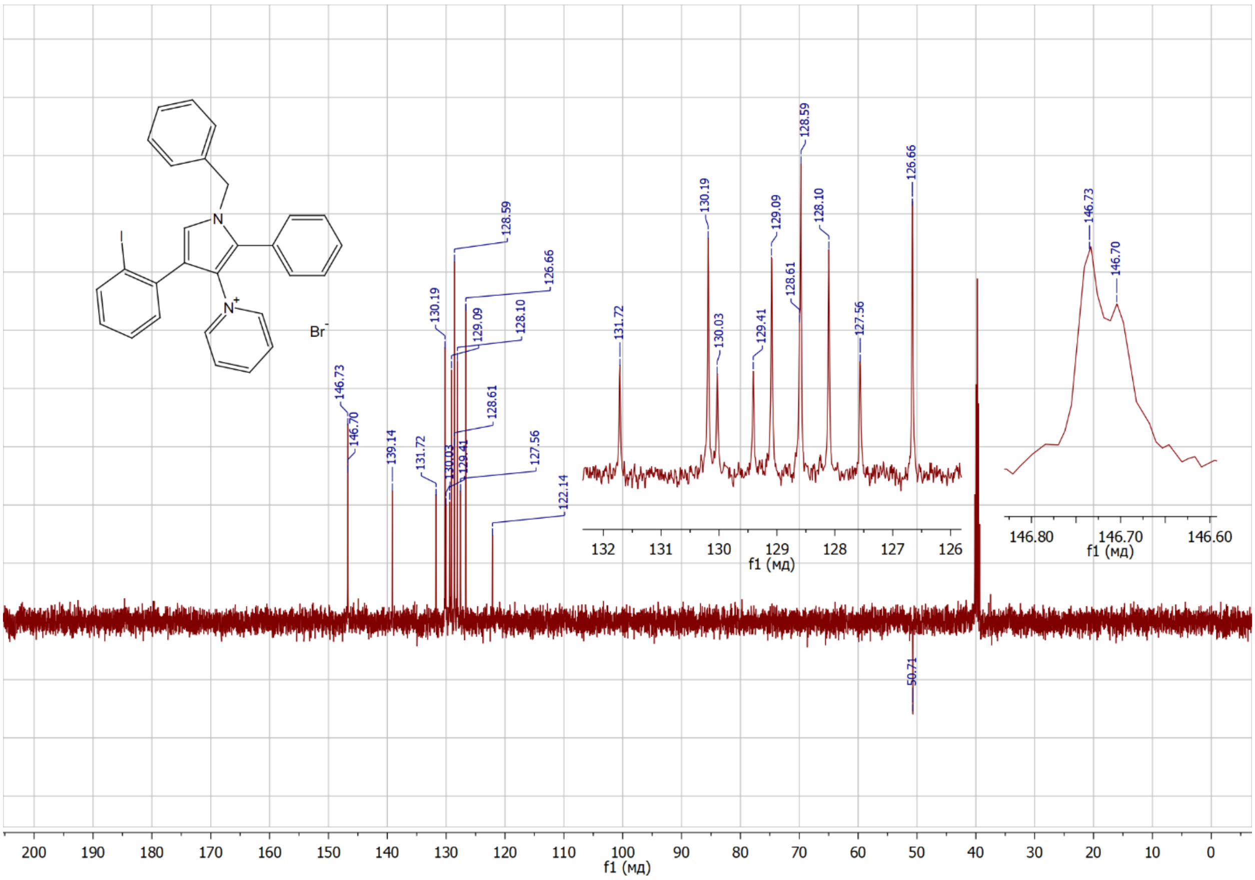

^{1}H NMR (400 MHz, DMSO-d_6) of 3-phenylpyrido[2,1-*a*]pyrrolo[3,4-*c*]isoquinolin-2-iumbromide (**3a**)

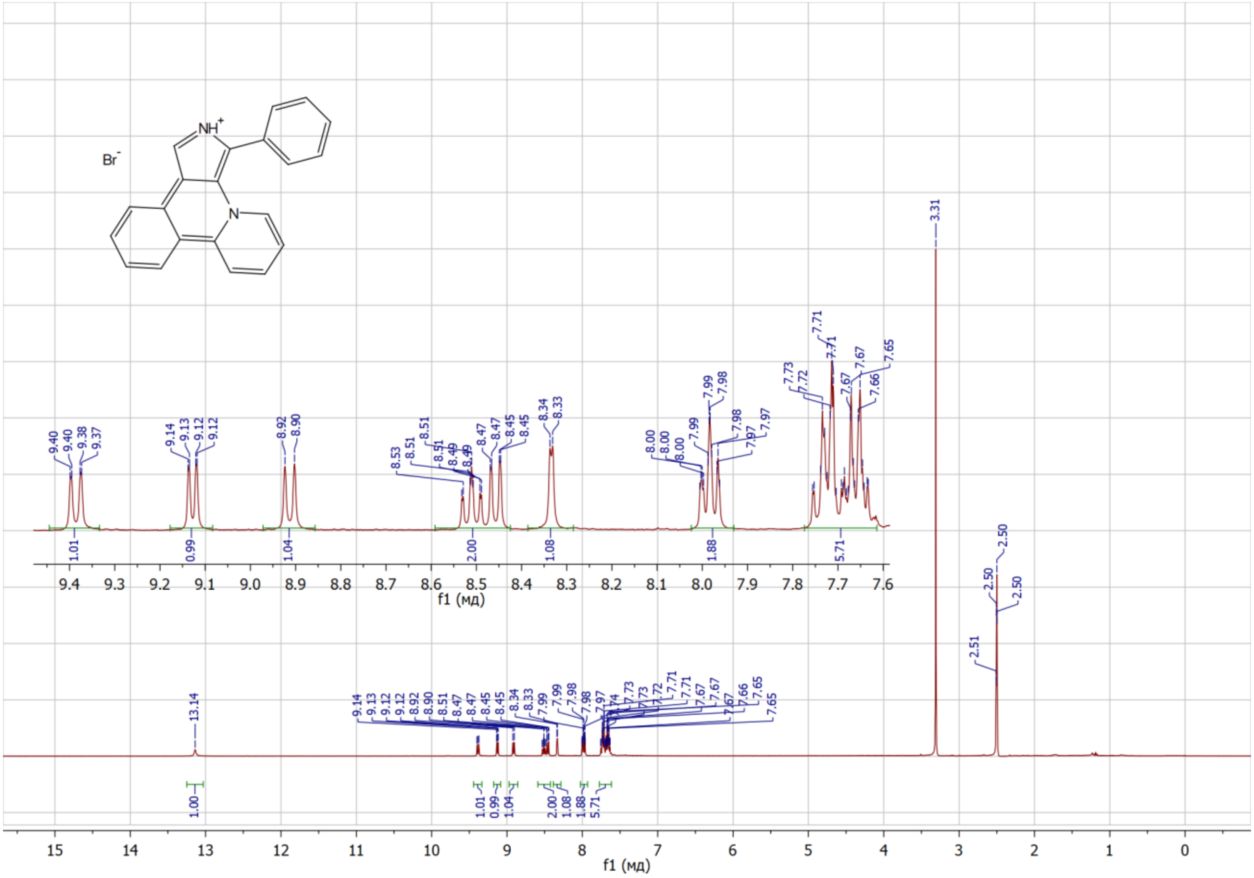

^{13}C{1H} NMR (101 MHz, DMSO-d_6) of 3-phenylpyrido[2,1-*a*]pyrrolo[3,4-*c*]isoquinolin-2-iumbromide (**3a**)

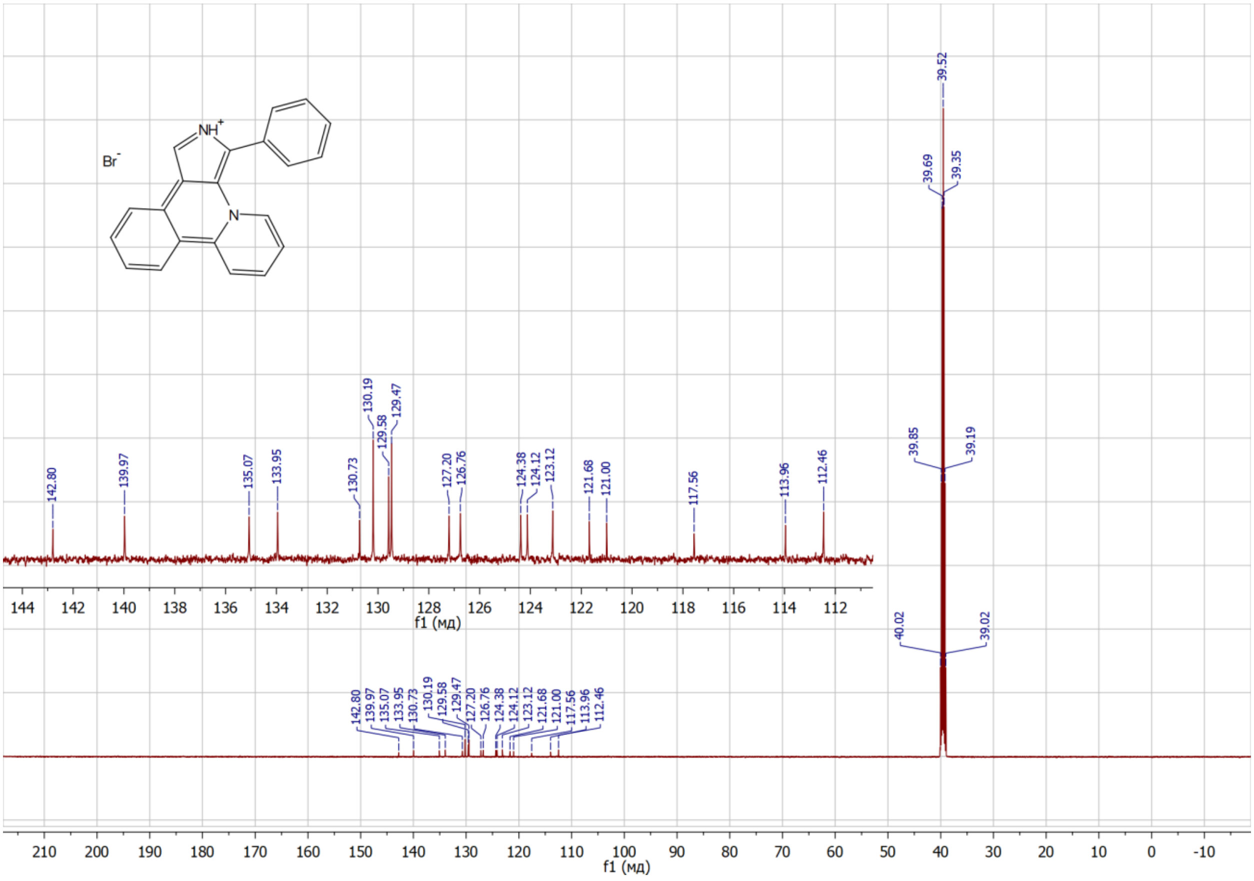

^{13}C DEPT135 NMR (101 MHz, DMSO-d_6) of 3-phenylpyrido[2,1-*a*]pyrrolo[3,4-*c*]isoquinolin-2-iumbromide (**3a**)

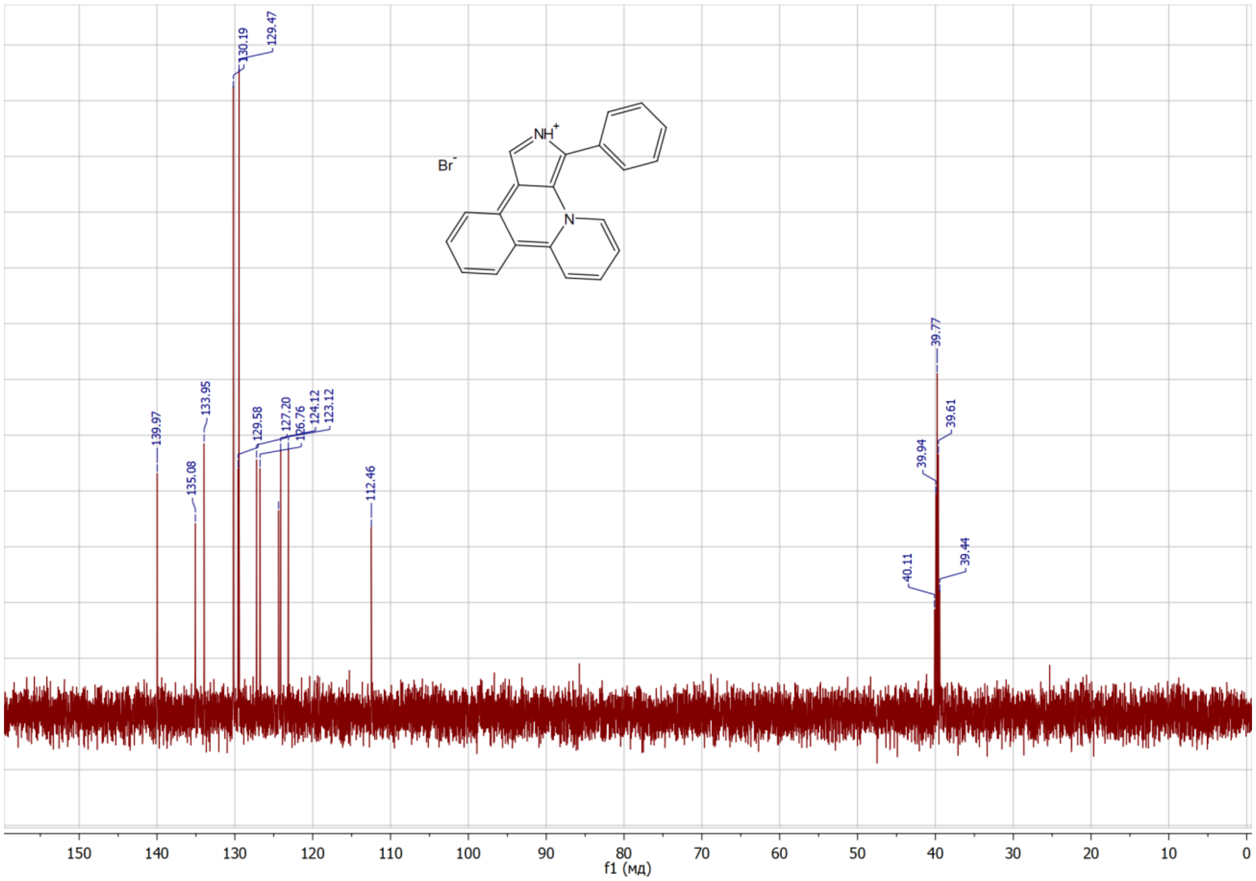

^{1}H NMR (400 MHz, DMSO-d_6) of 2-methyl-3-phenyl-2*H*-pyrido[2,1-*a*]pyrrolo[3,4-*c*]isoquinolin-4-ium iodide (N-Me-**3a**)

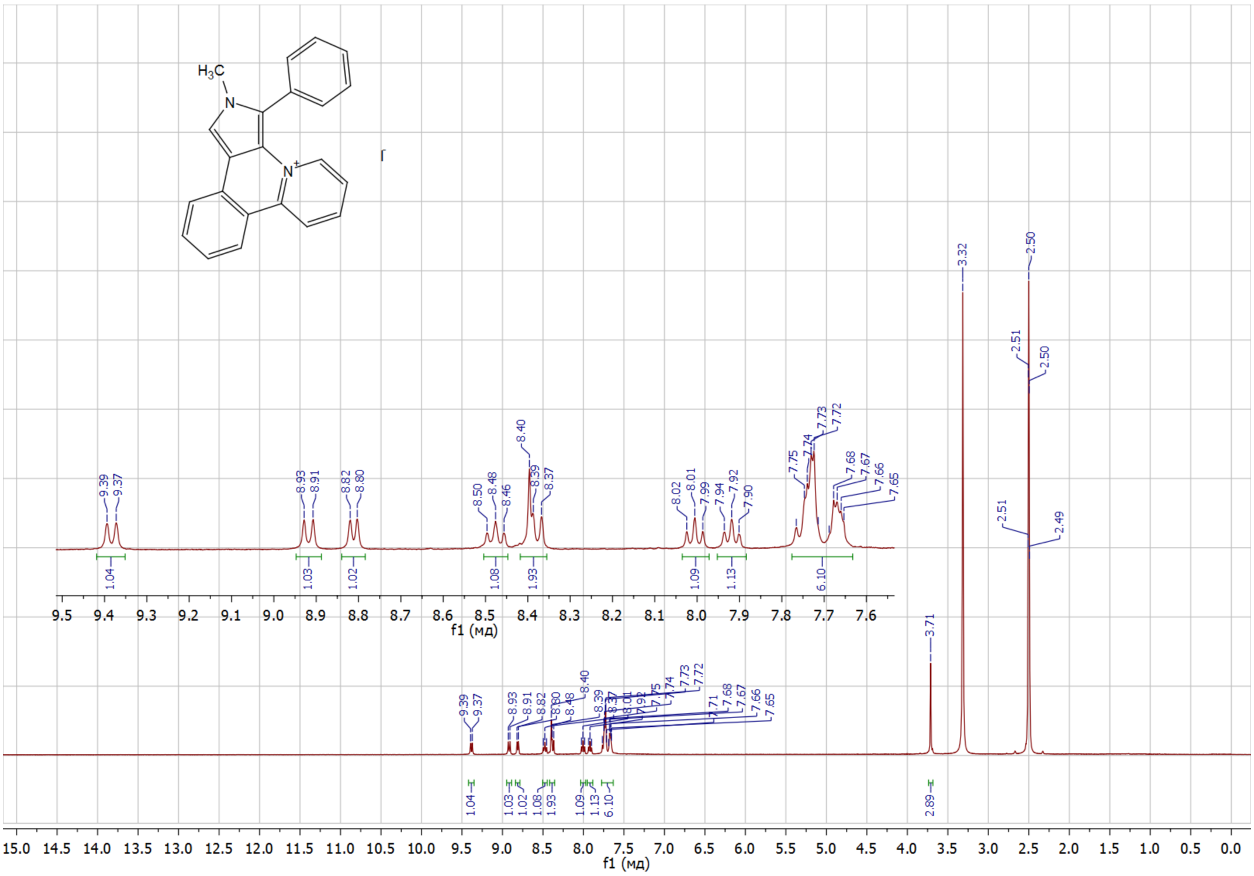

^{13}C{1H} NMR (101 MHz, DMSO-d_6) of 2-methyl-3-phenyl-2*H*-pyrido[2,1-*a*]pyrrolo[3,4-*c*]isoquinolin-4-ium iodide (N-Me-**3a**)

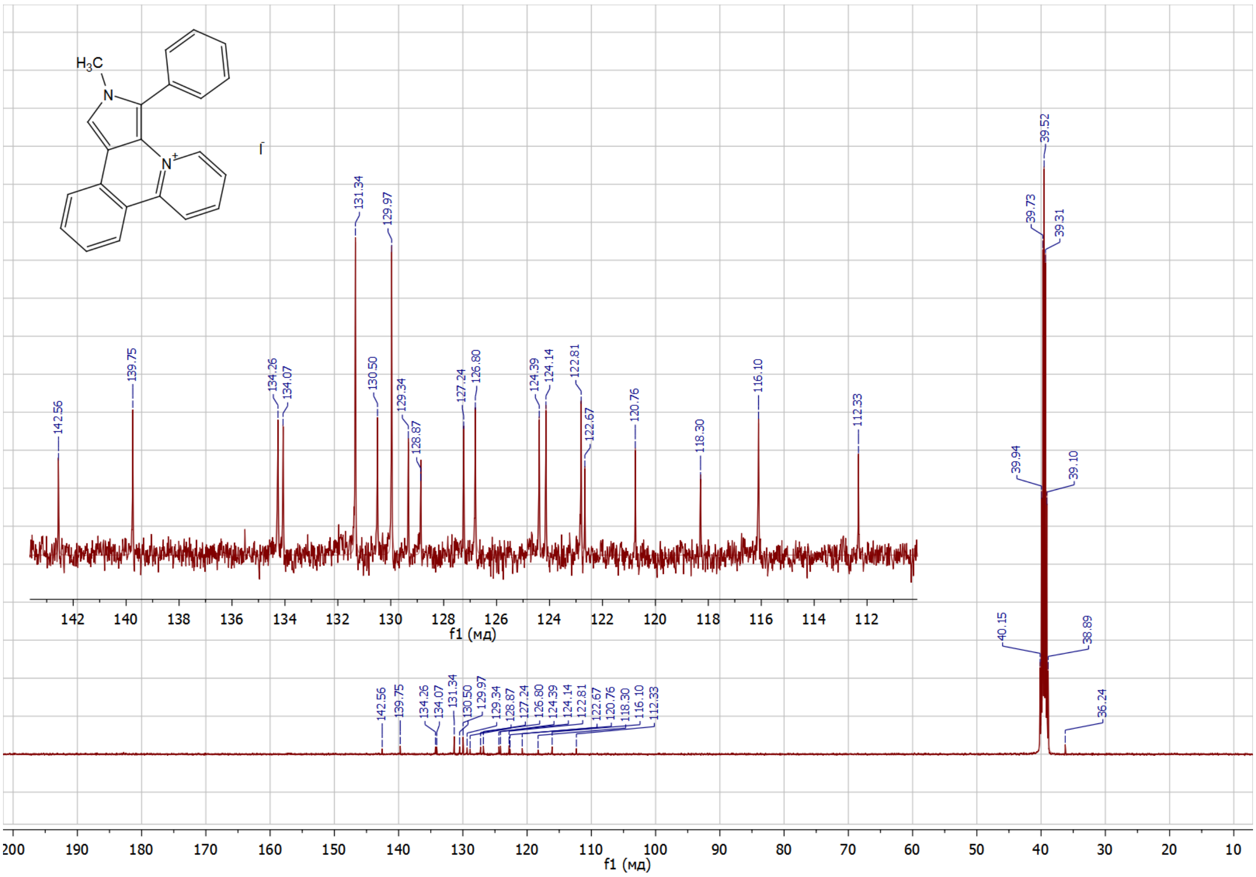

^{13}C DEPT135 NMR (101 MHz, DMSO-d_6) of 2-methyl-3-phenyl-2*H*-pyrido[2,1-*a*]pyrrolo[3,4-*c*]isoquinolin-4-ium iodide (N-Me-**3a**)

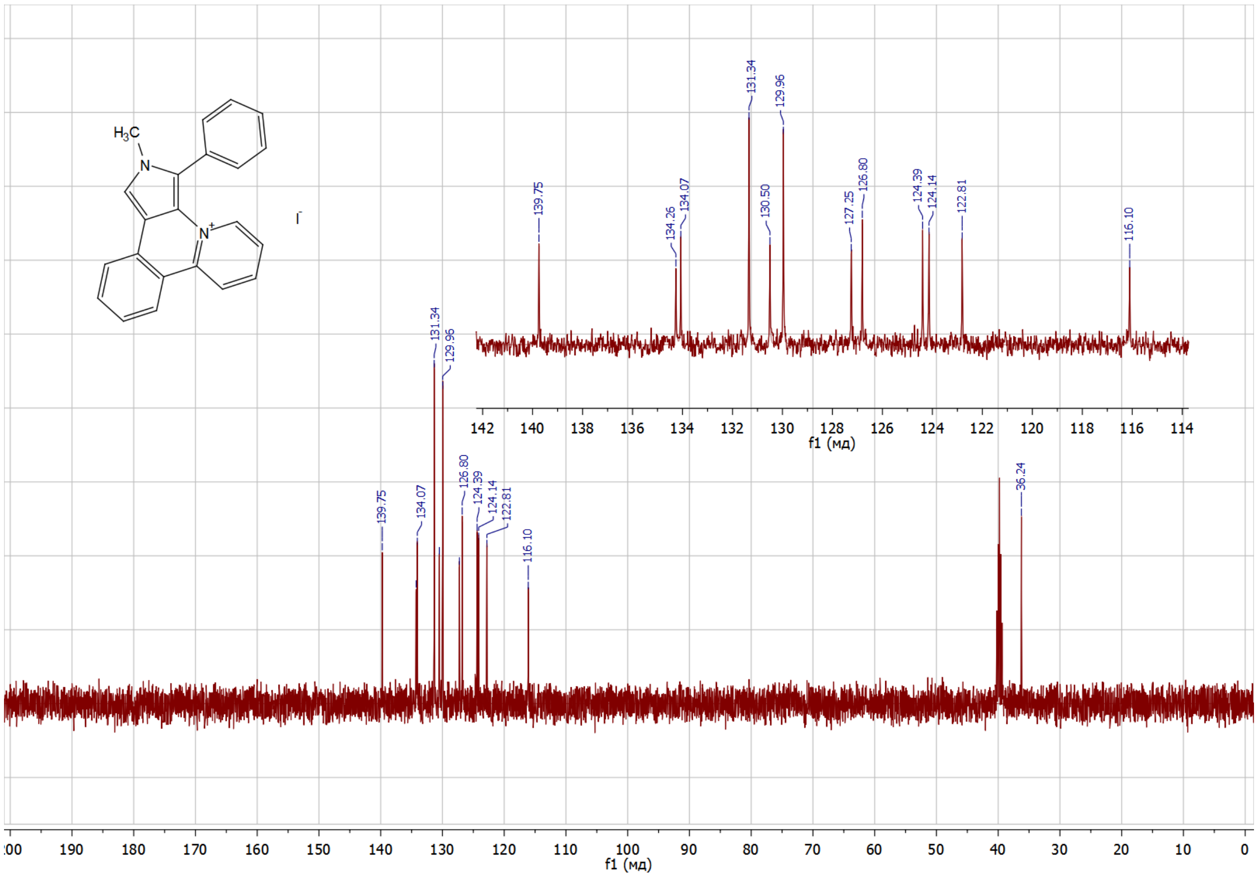

^{1}H NMR (400 MHz, $CDCl_3$) of 2-benzyl-3-phenyl-2*H*-pyrido[2,1-*a*]pyrrolo[3,4-*c*]isoquinolin-4-ium bromide (N-Bn-**3a**)

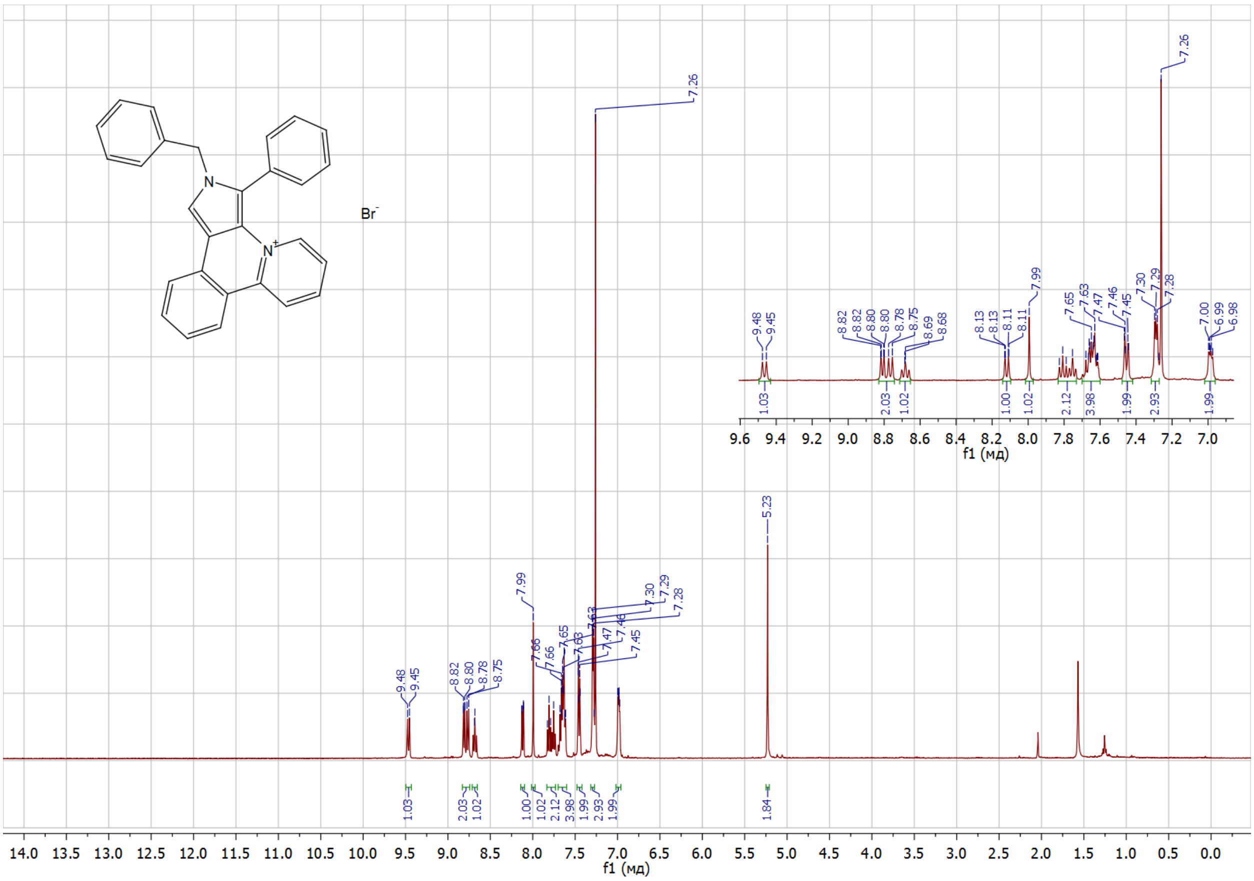

^{13}C{1H} NMR (101 MHz, $CDCl_3$) of 2-benzyl-3-phenyl-2*H*-pyrido[2,1-*a*]pyrrolo[3,4-*c*]isoquinolin-4-ium bromide (N-Bn-**3a**)

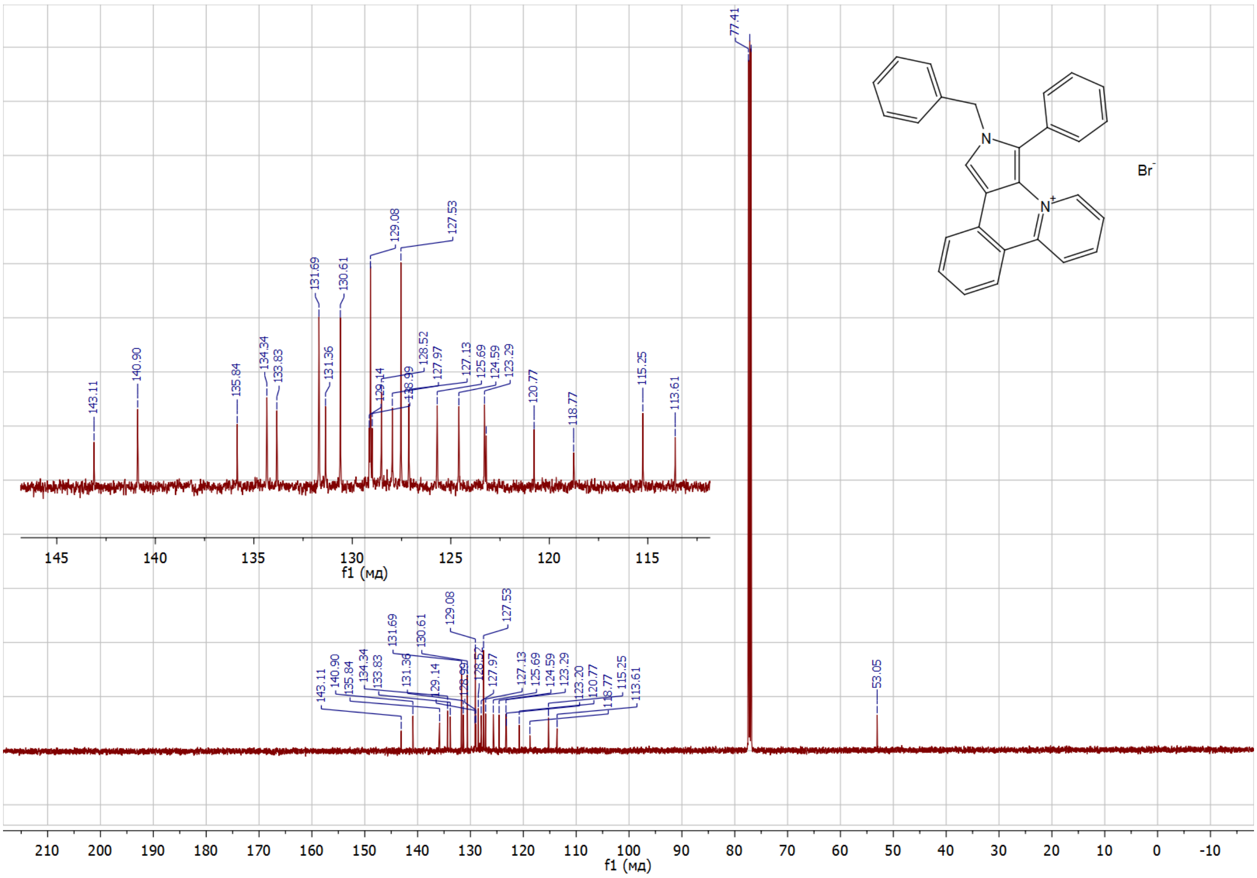

^{13}C DEPT135 NMR (101 MHz, CDCl3) of 2-benzyl-3-phenyl-2*H*-pyrido[2,1-*a*]pyrrolo[3,4-*c*]isoquinolin-4-ium bromide (N-Bn-**3a**)

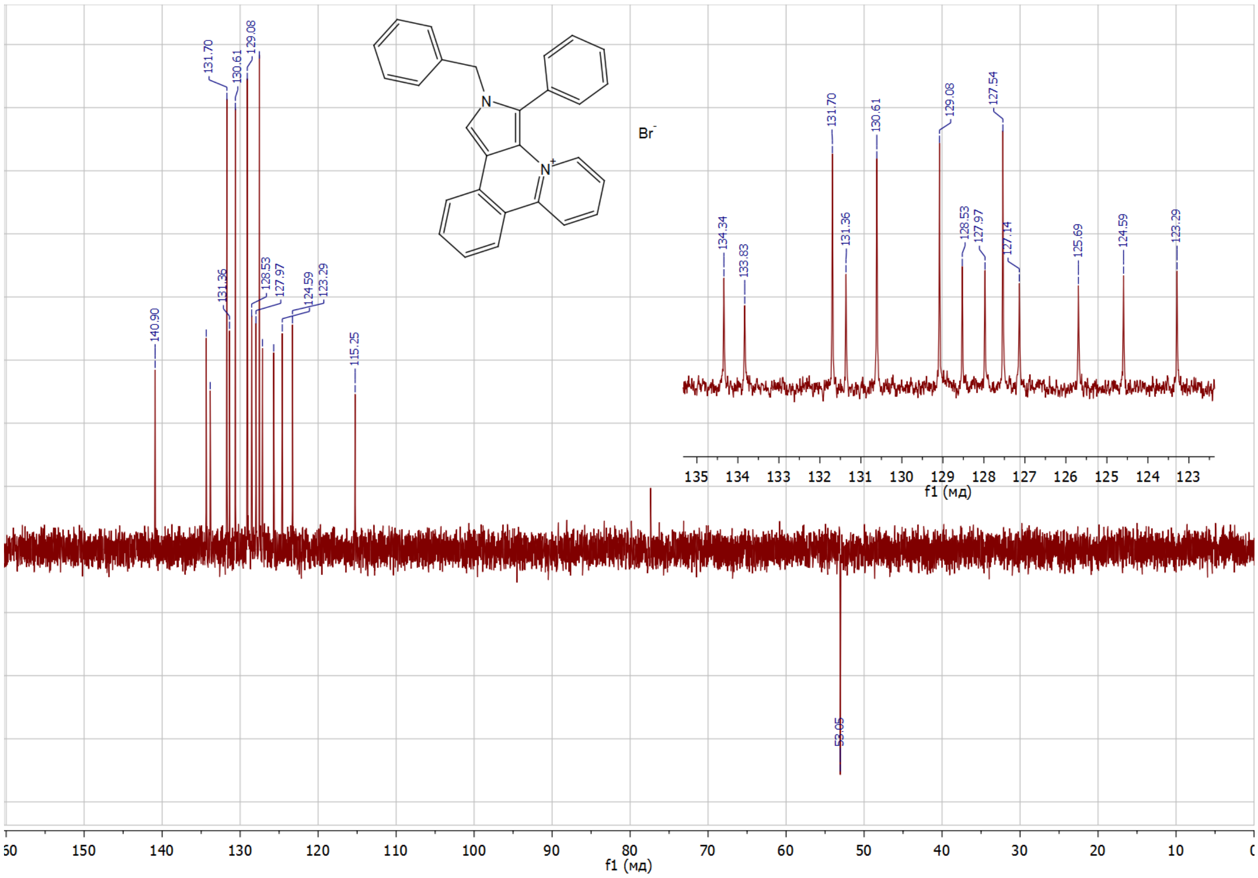

^{1}H NMR (400 MHz, DMSO-d_6) of 3-(4-fluorophenyl)pyrido[2,1-*a*]pyrrolo[3,4-*c*]isoquinolin-2-ium bromide (**3b**)

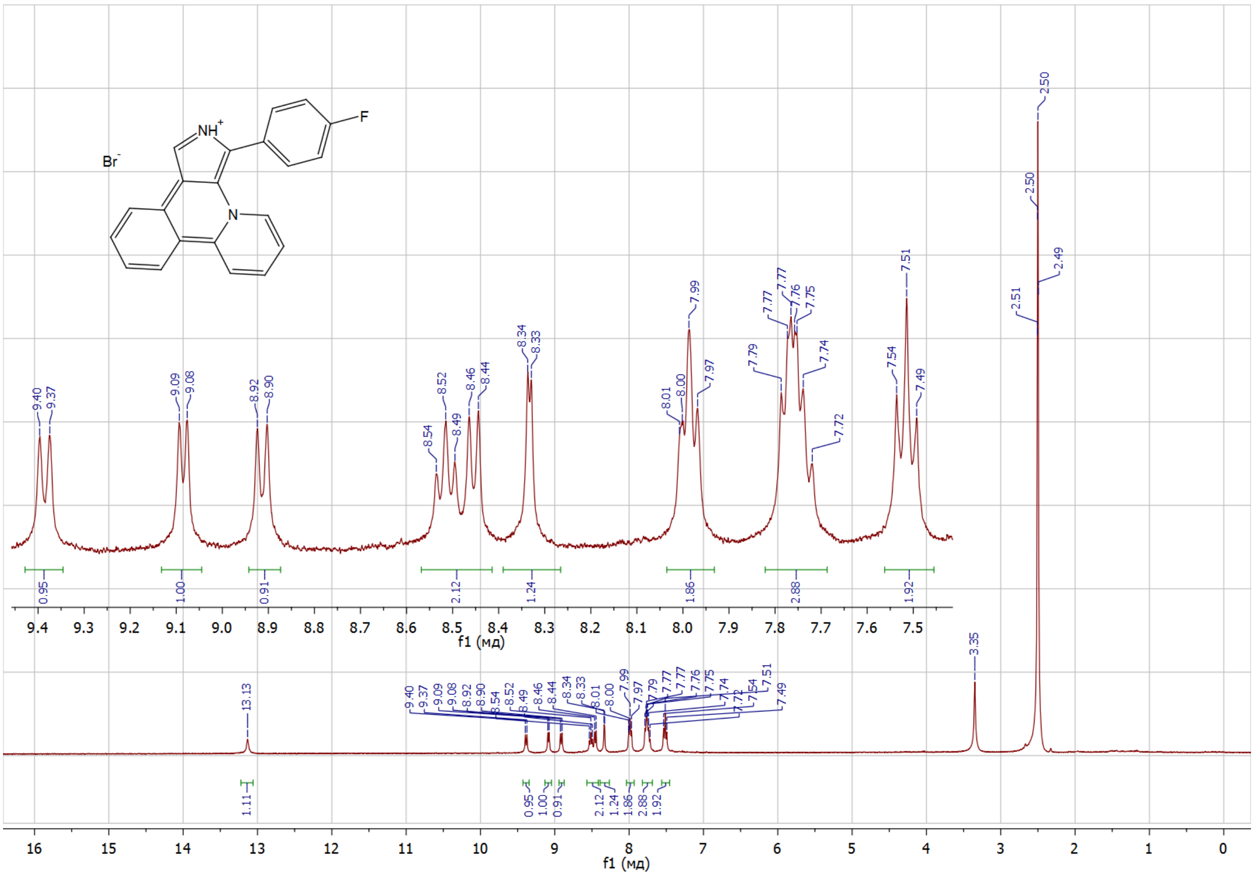

^{13}C{1H} NMR (101 MHz, DMSO-d_6) of 3-(4-fluorophenyl)pyrido[2,1-*a*]pyrrolo[3,4-*c*]isoquinolin-2-ium bromide (**3b**)

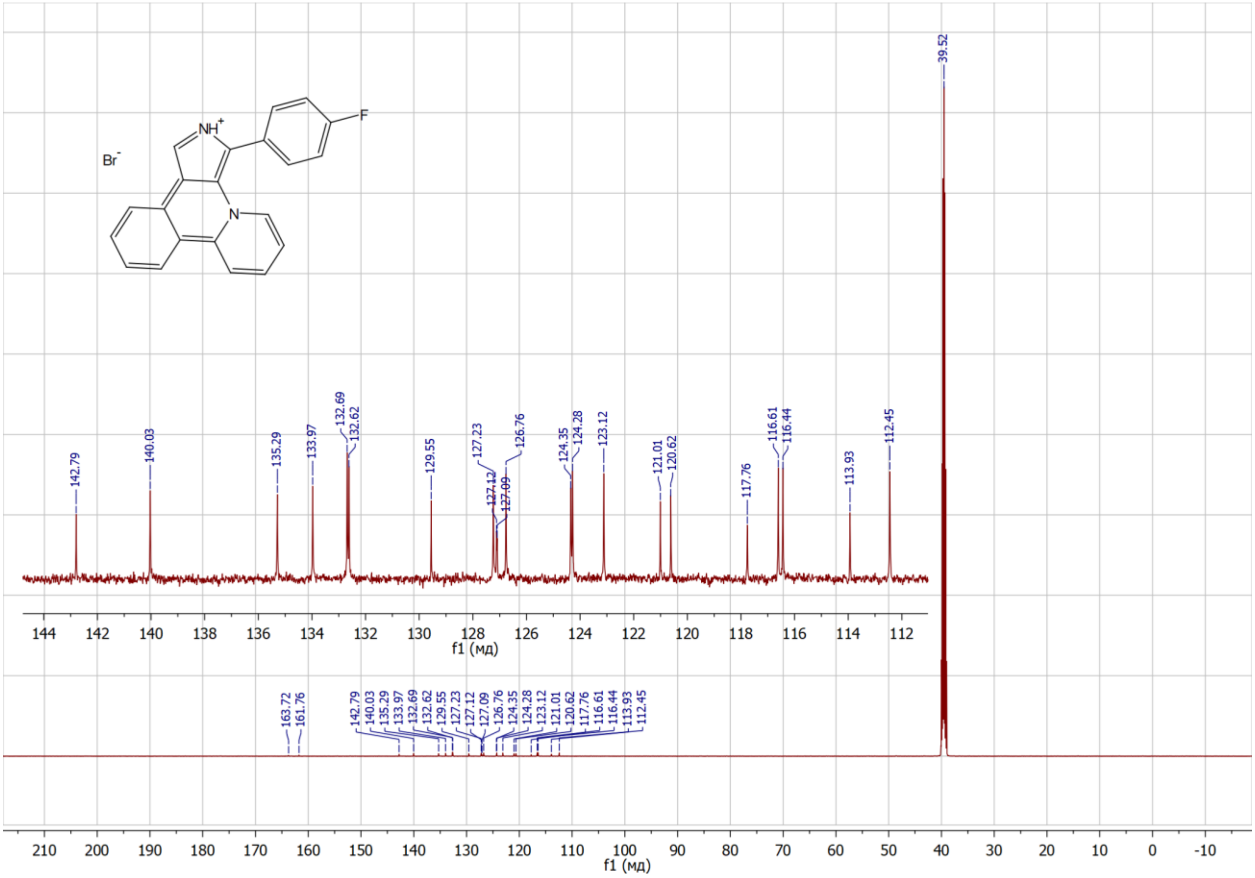

^{13}C DEPT135 NMR (101 MHz, DMSO-d_6) of 3-(4-fluorophenyl)pyrido[2,1-*a*]pyrrolo[3,4-*c*]isoquinolin-2-ium bromide (**3b**)

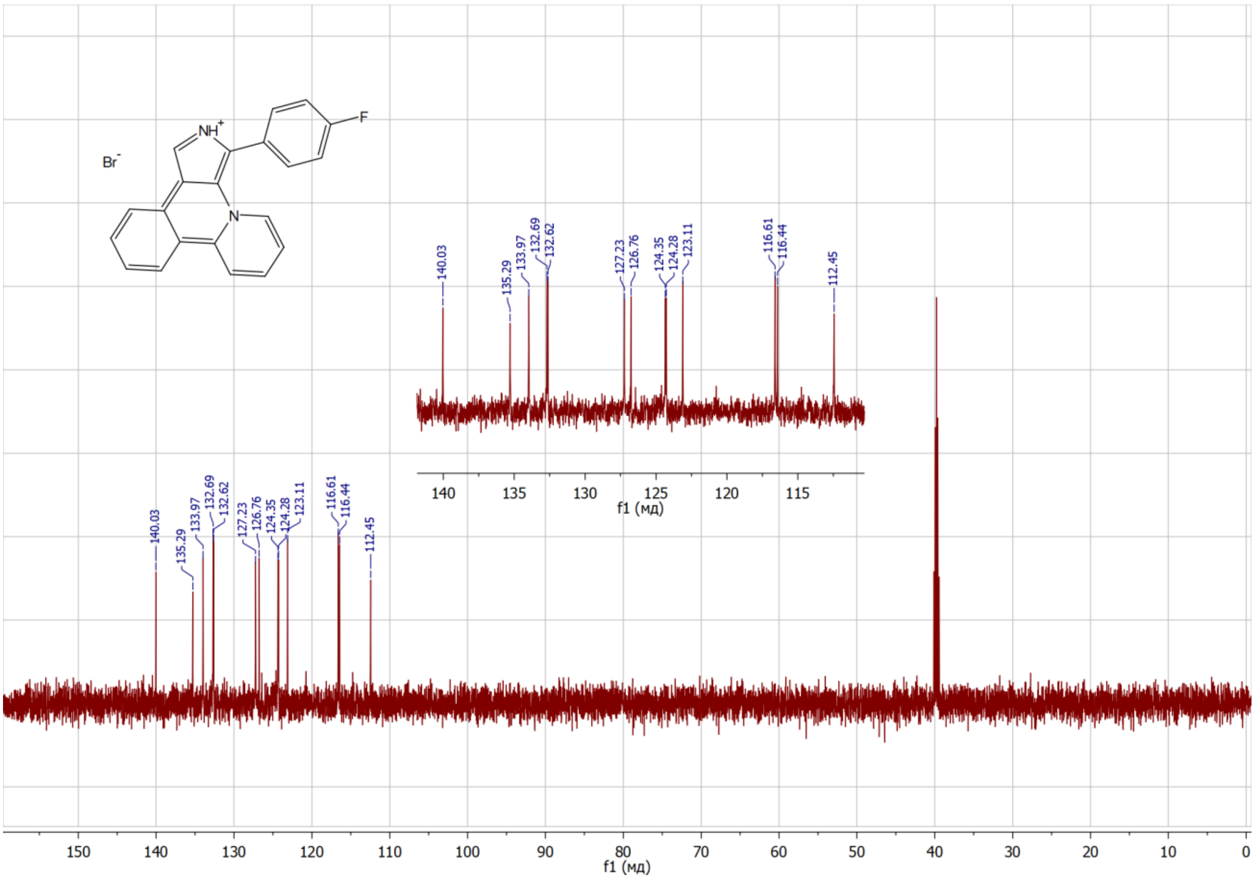

^{1}H NMR (400 MHz, DMSO-d_6) of 3-(4-chlorophenyl)pyrido[2,1-*a*]pyrrolo[3,4-*c*]isoquinolin-2-ium bromide (**3c**)

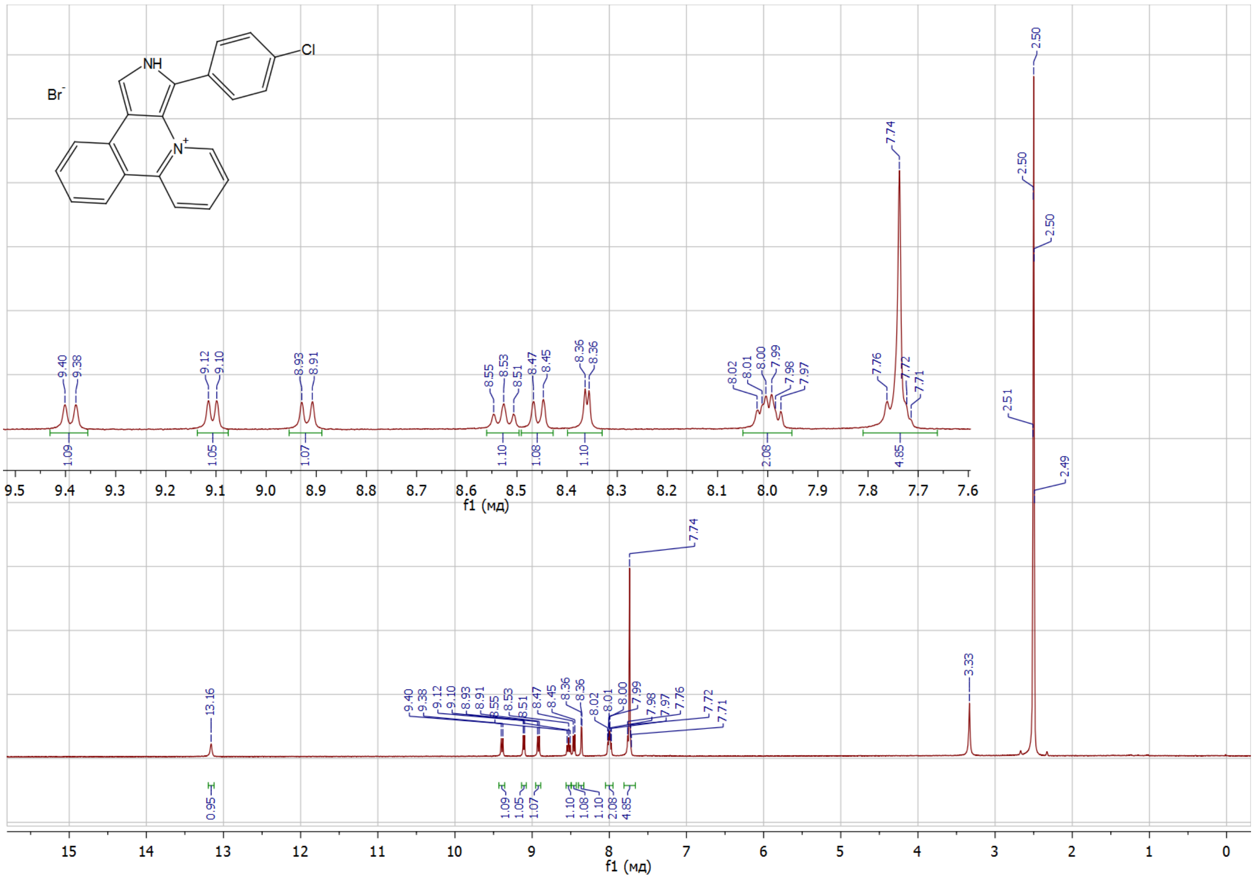

$^{13}C\{1H\}$ NMR (101 MHz, DMSO-d_6) of 3-(4-chlorophenyl)pyrido[2,1-*a*]pyrrolo[3,4-*c*]isoquinolin-2-ium bromide (**3c**)

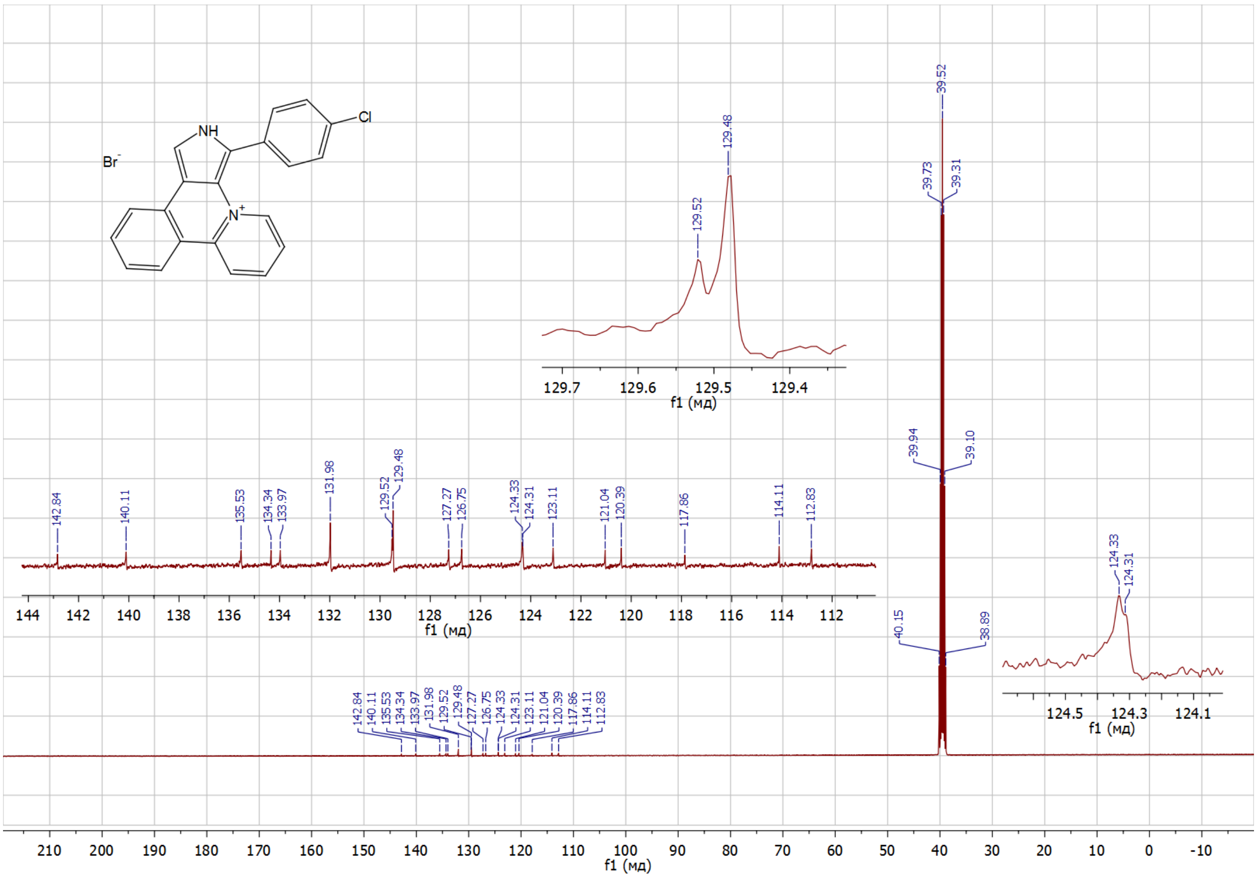

^{13}C DEPT135 NMR (101 MHz, DMSO-d_6) of 3-(4-chlorophenyl)pyrido[2,1-*a*]pyrrolo[3,4-*c*]isoquinolin-2-ium bromide (**3c**)

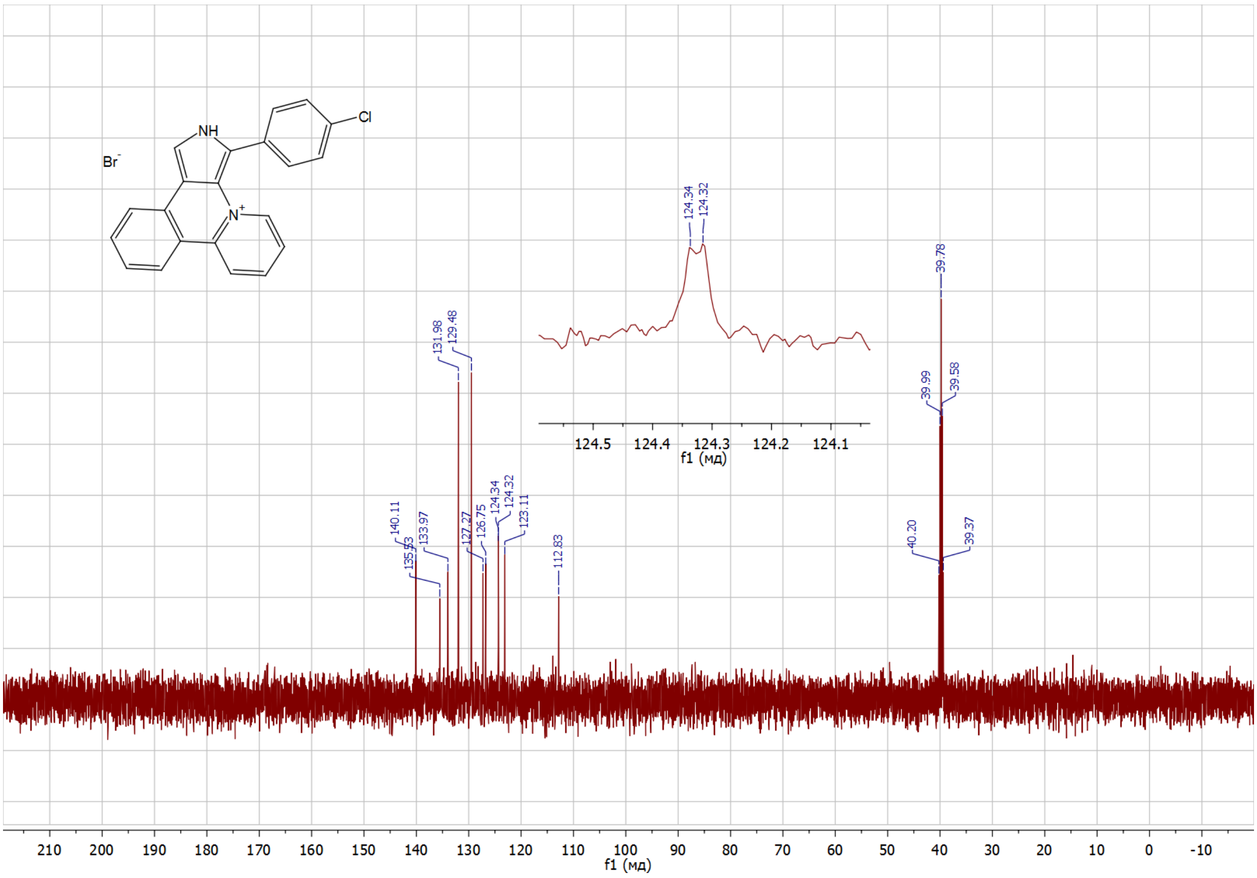

^{1}H NMR (400 MHz, DMSO-d_6) of 3-(4-methylphenyl)pyrido[2,1-*a*]pyrrolo[3,4-*c*]isoquinolin-2-ium bromide (**3e**)

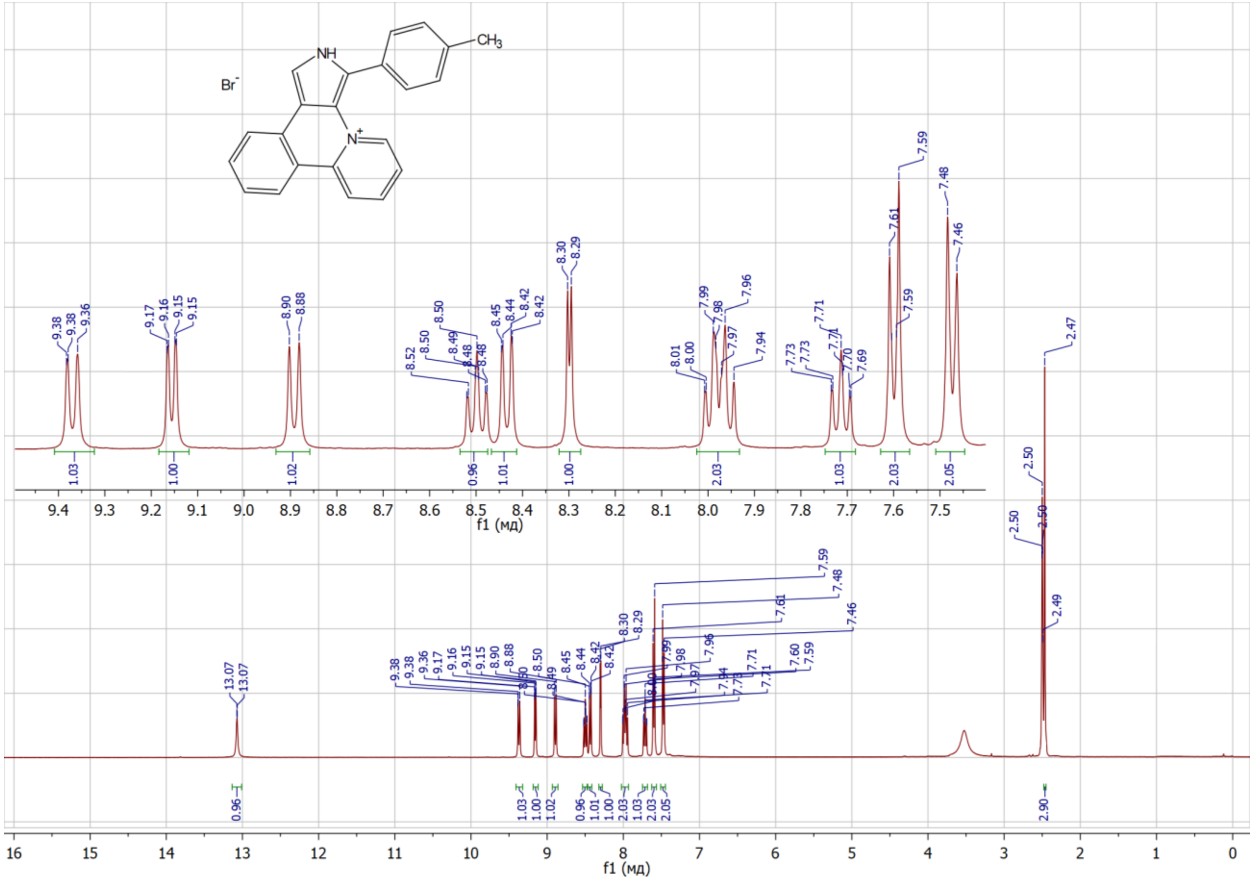

^{13}C{1H} NMR (101 MHz, DMSO-d_6) of 3-(4-methylphenyl)pyrido[2,1-*a*]pyrrolo[3,4-*c*]isoquinolin-2-ium bromide (**3e**)

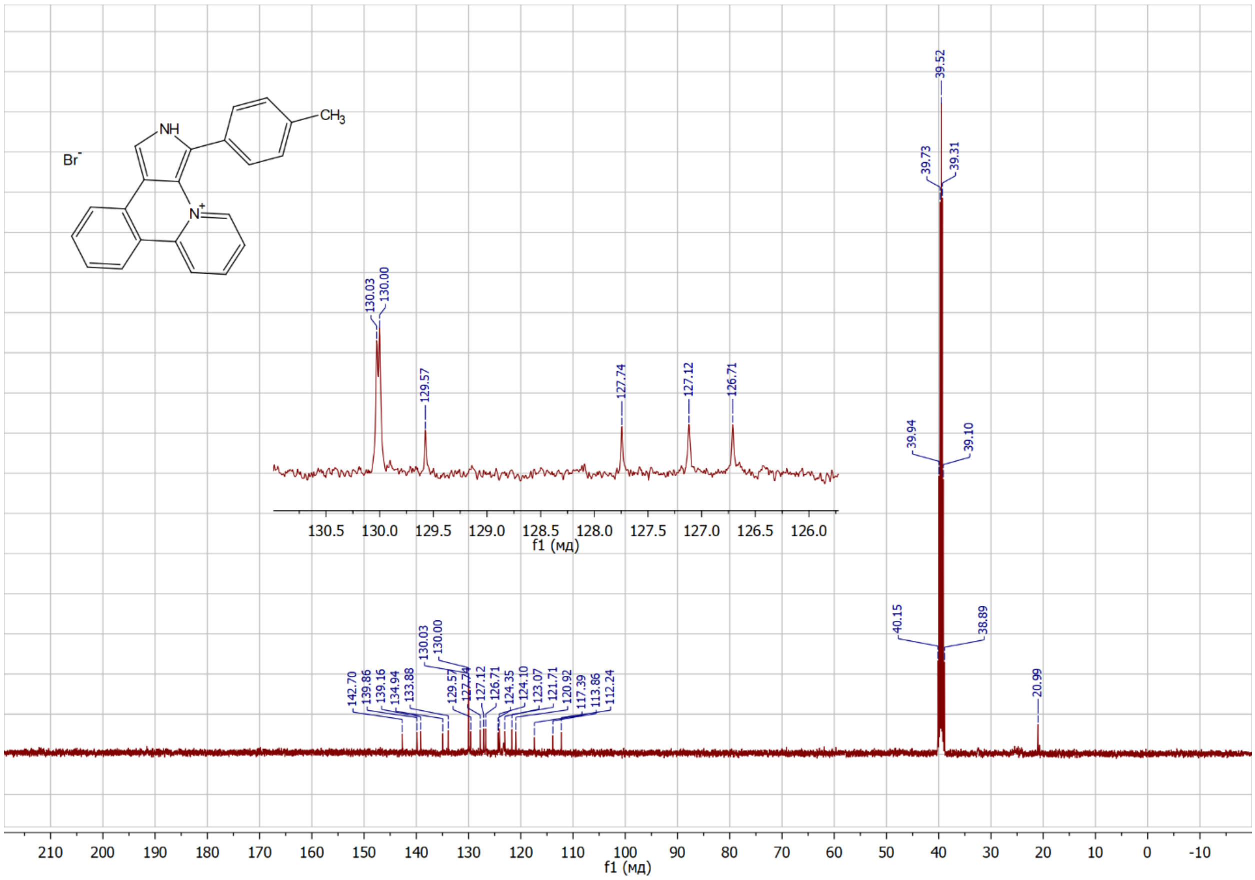

^{13}C DEPT135 NMR (101 MHz, DMSO-d_6) of 3-(4-methylphenyl)pyrido[2,1-*a*]pyrrolo[3,4-*c*]isoquinolin-2-ium bromide (**3e**)

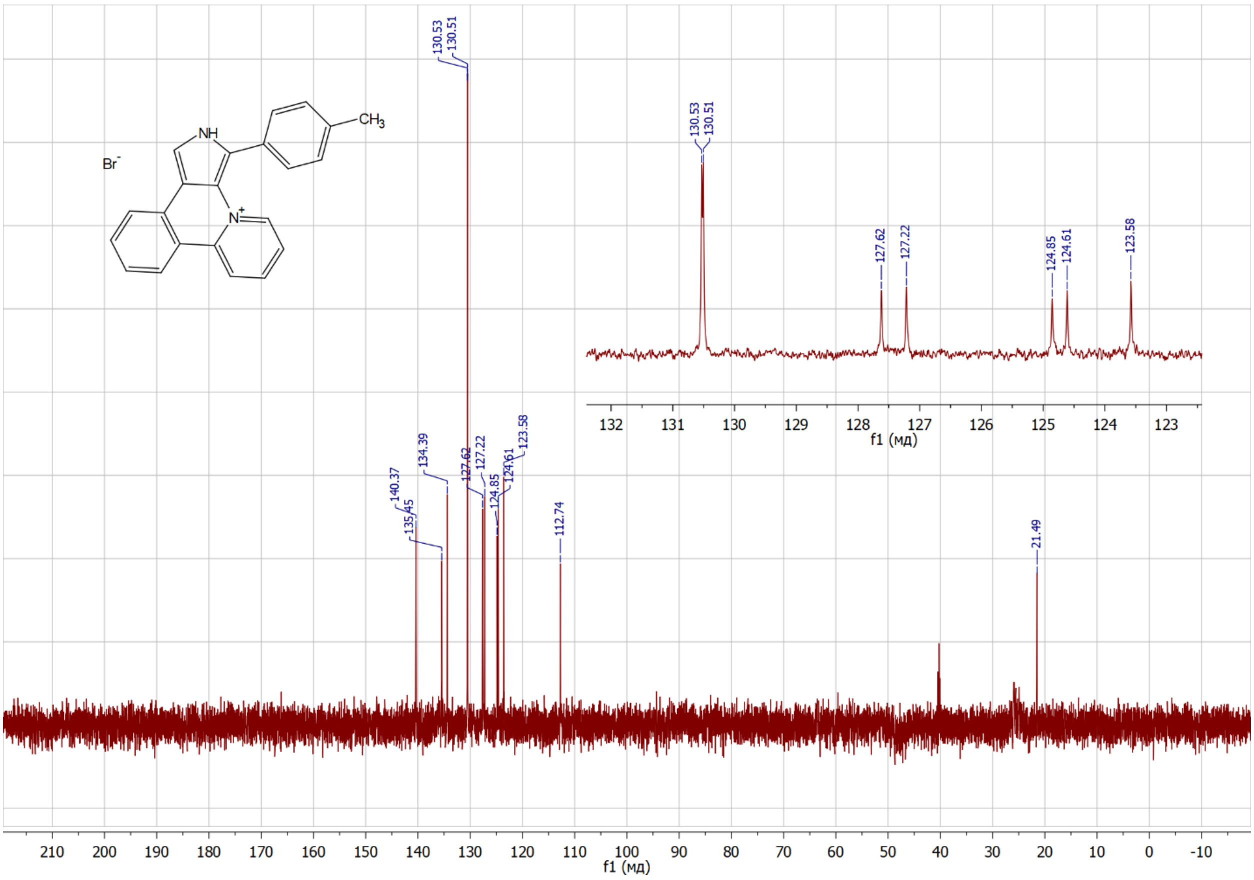

^{1}H NMR (400 MHz, DMSO-d_6) of 3-(4-methoxyphenyl)pyrido[2,1-*a*]pyrrolo[3,4-*c*]isoquinolin-2-ium bromide (**3f**)

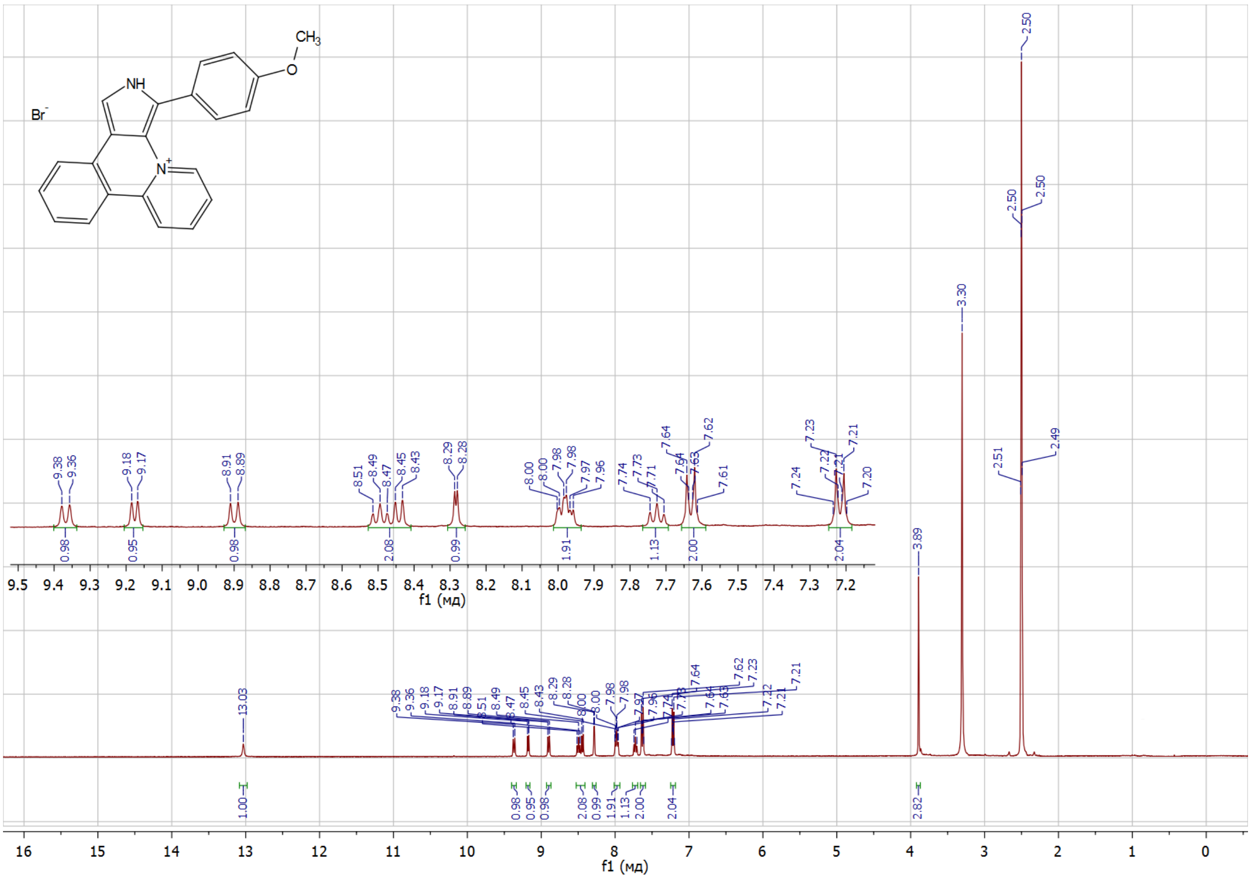

$^{13}C\{1H\}$ NMR (101 MHz, DMSO-d_6) of 3-(4-methoxyphenyl)pyrido[2,1-*a*]pyrrolo[3,4-*c*]isoquinolin-2-ium bromide (**3f**)

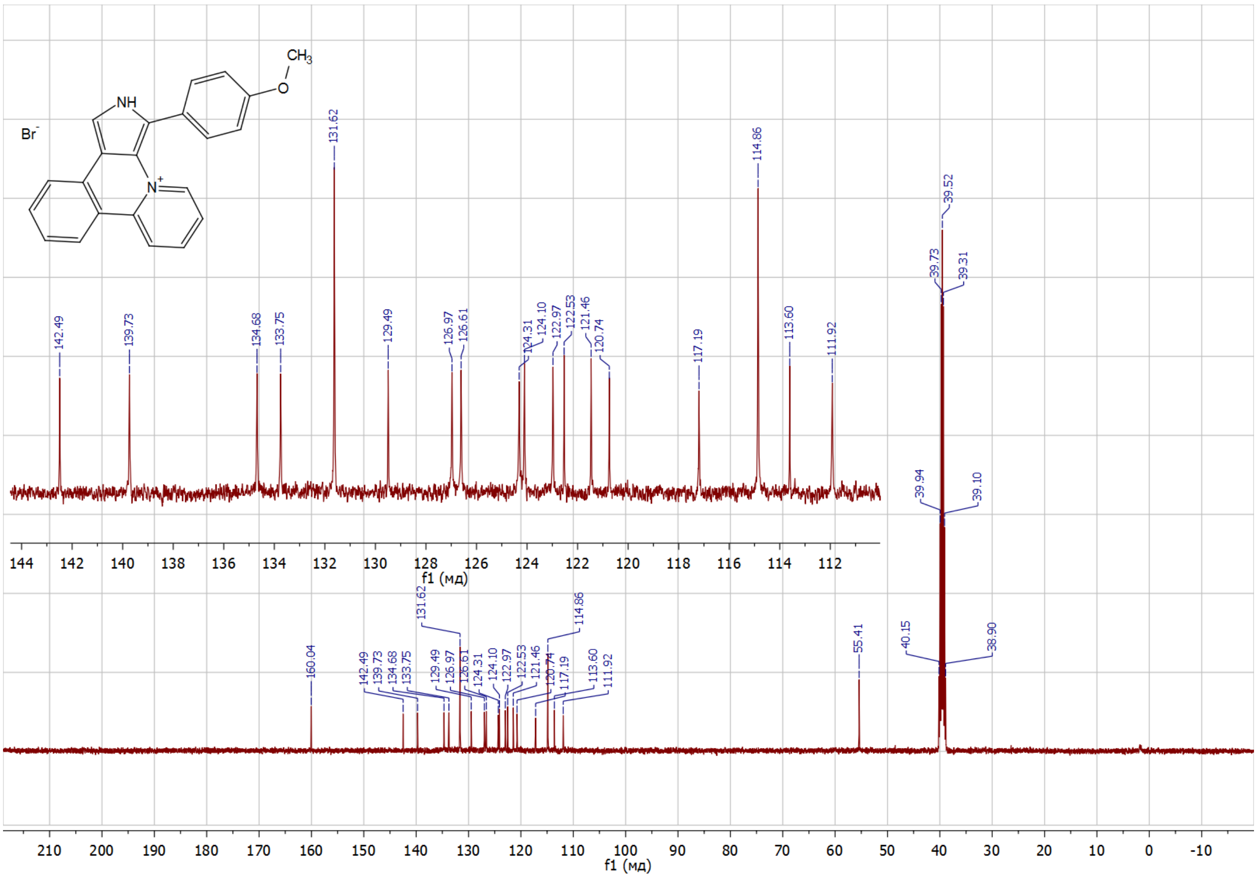

^{13}C DEPT135 NMR (101 MHz, DMSO-d_6) of 3-(4-methoxyphenyl)pyrido[2,1-*a*]pyrrolo[3,4-*c*]isoquinolin-2-ium bromide (**3f**)

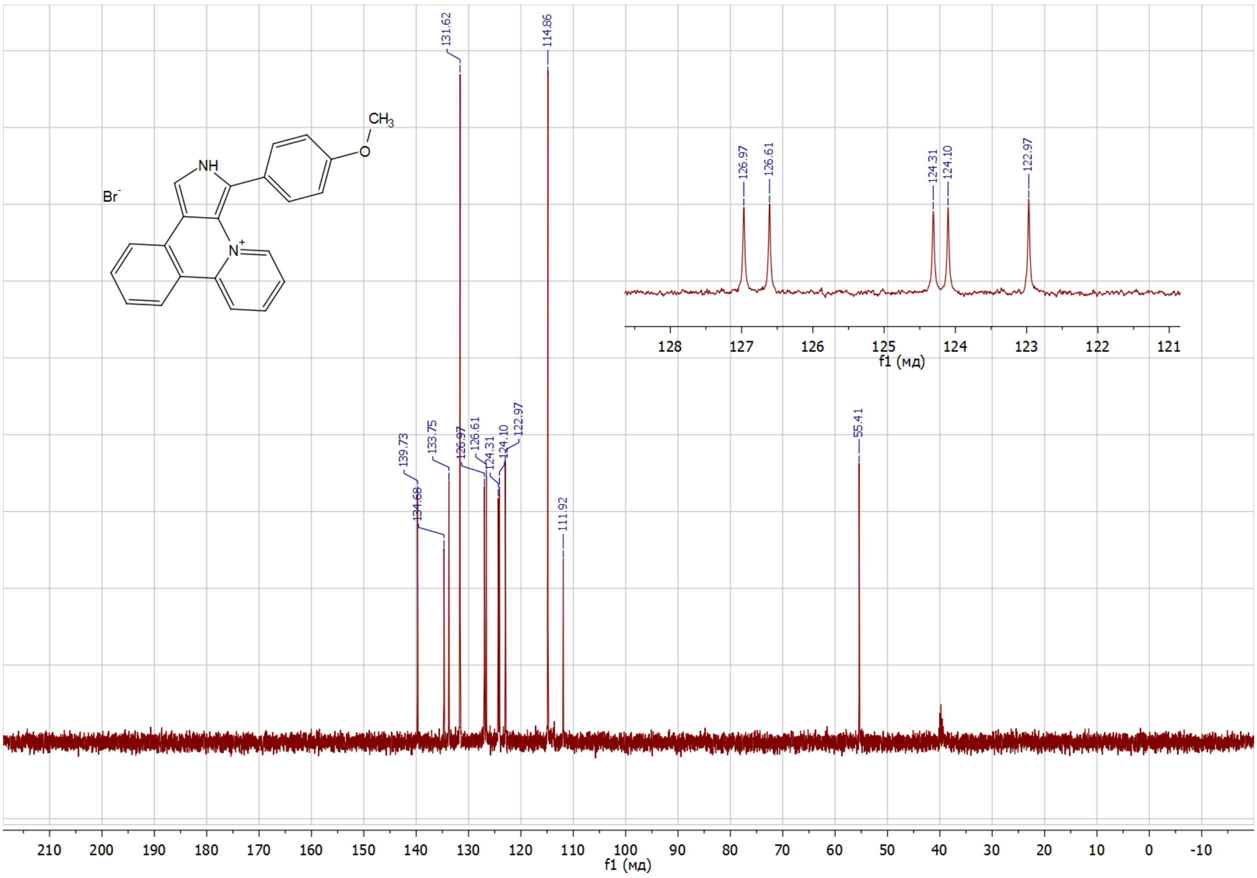

^{1}H NMR (400 MHz, DMSO-d_6) of 3-(2-methoxyphenyl)pyrido[2,1-*a*]pyrrolo[3,4-*c*]isoquinolin-2-ium bromide (**3g**)

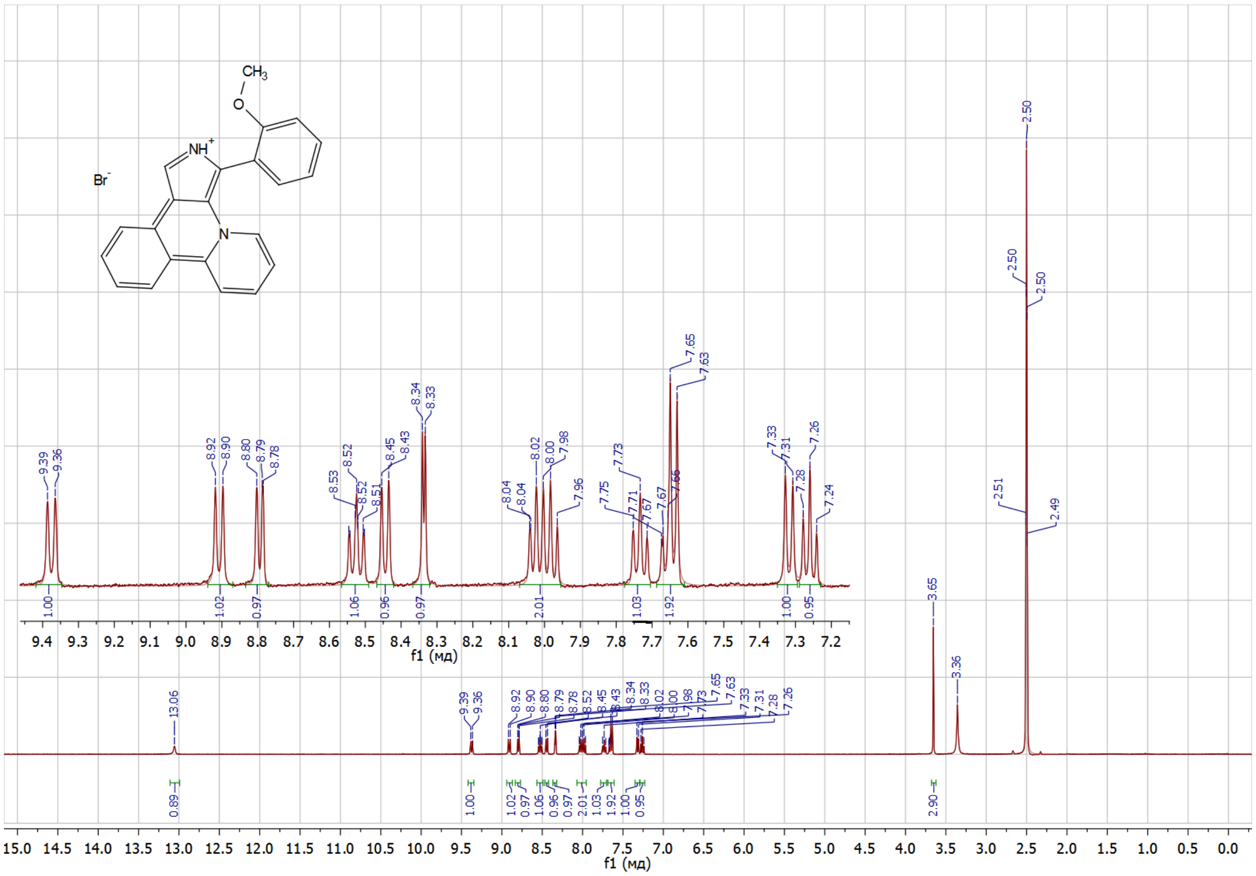

^{13}C{1H} NMR (101 MHz, DMSO-d_6) of 3-(2-methoxyphenyl)pyrido[2,1-*a*]pyrrolo[3,4-*c*]isoquinolin-2-ium bromide (**3g**)

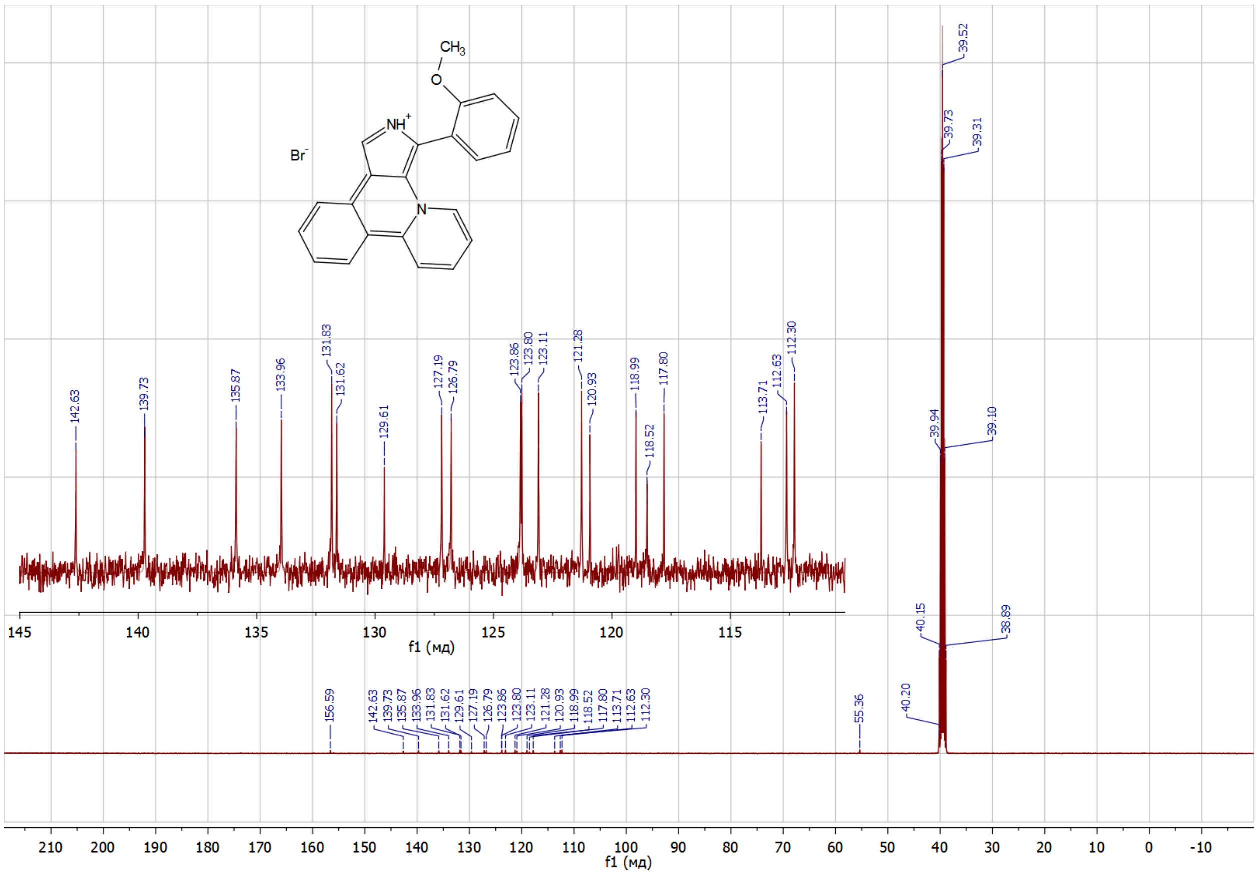

^{13}C DEPT135 NMR (101 MHz, DMSO-d_6) of 3-(2-methoxyphenyl)pyrido[2,1-*a*]pyrrolo[3,4-*c*]isoquinolin-2-ium bromide (**3g**)

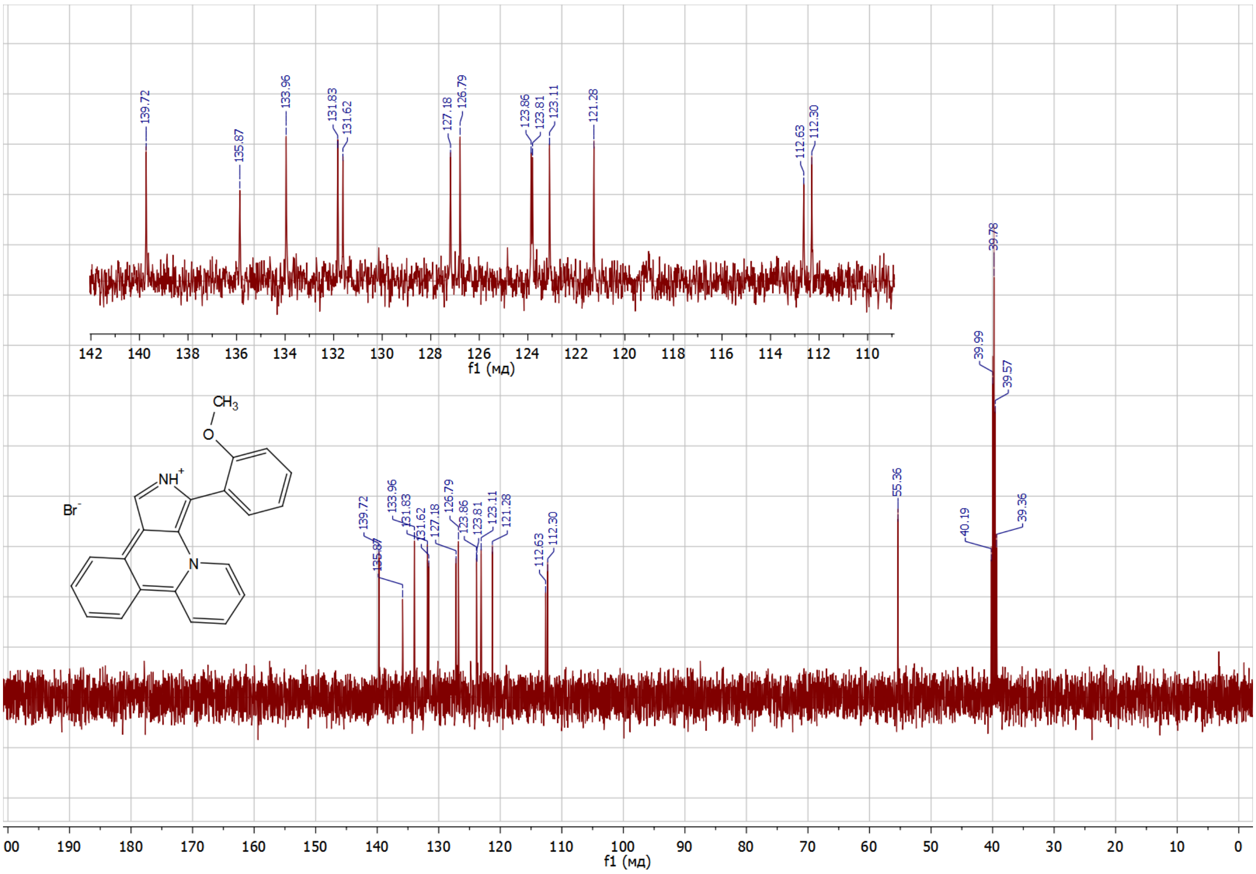

^{1}H NMR (400 MHz, DMSO-d_6) of 3-(3-methoxyphenyl)pyrido[2,1-*a*]pyrrolo[3,4-*c*]isoquinolin-2-ium bromide (**3h**)

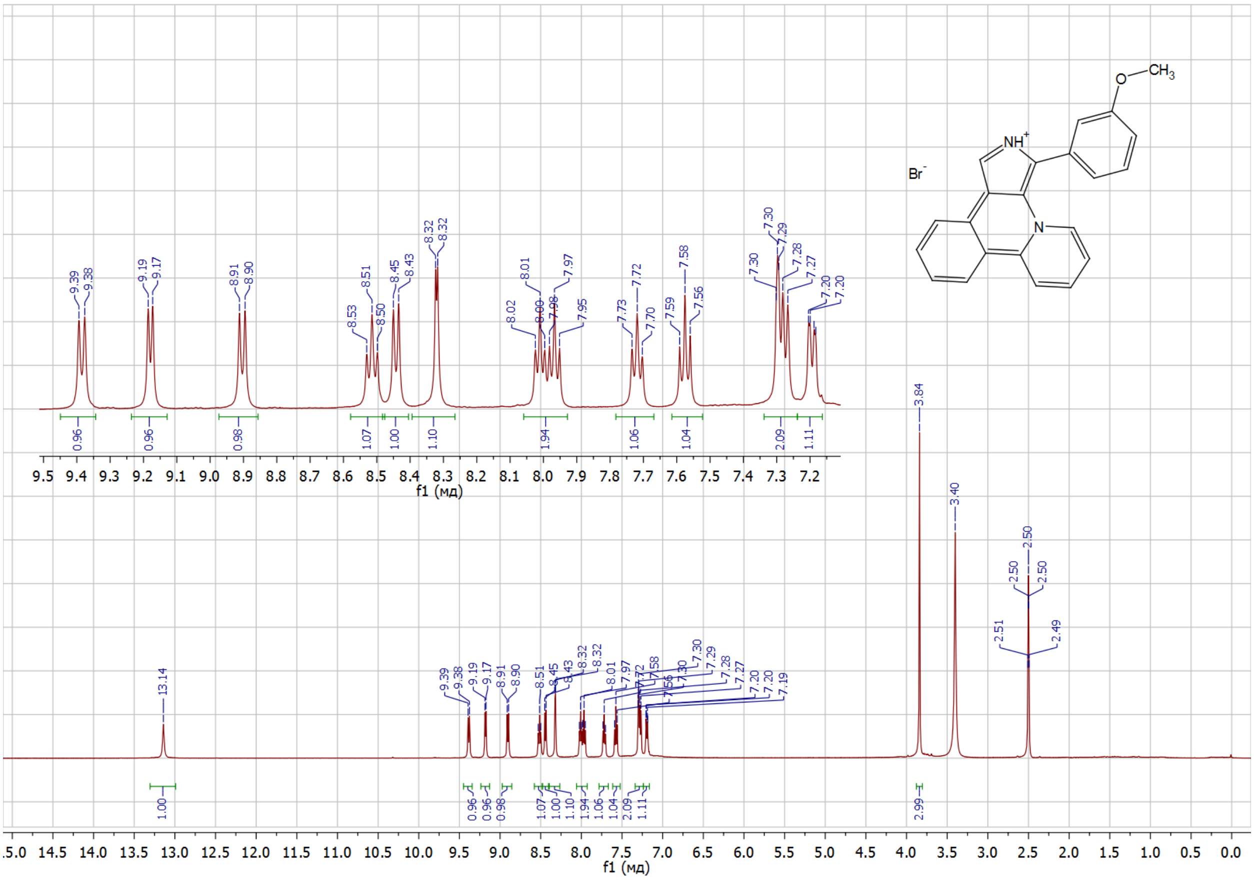

^{13}C{1H} NMR (101 MHz, DMSO-d_6) of 3-(3-methoxyphenyl)pyrido[2,1-*a*]pyrrolo[3,4-*c*]isoquinolin-2-ium bromide (**3h**)

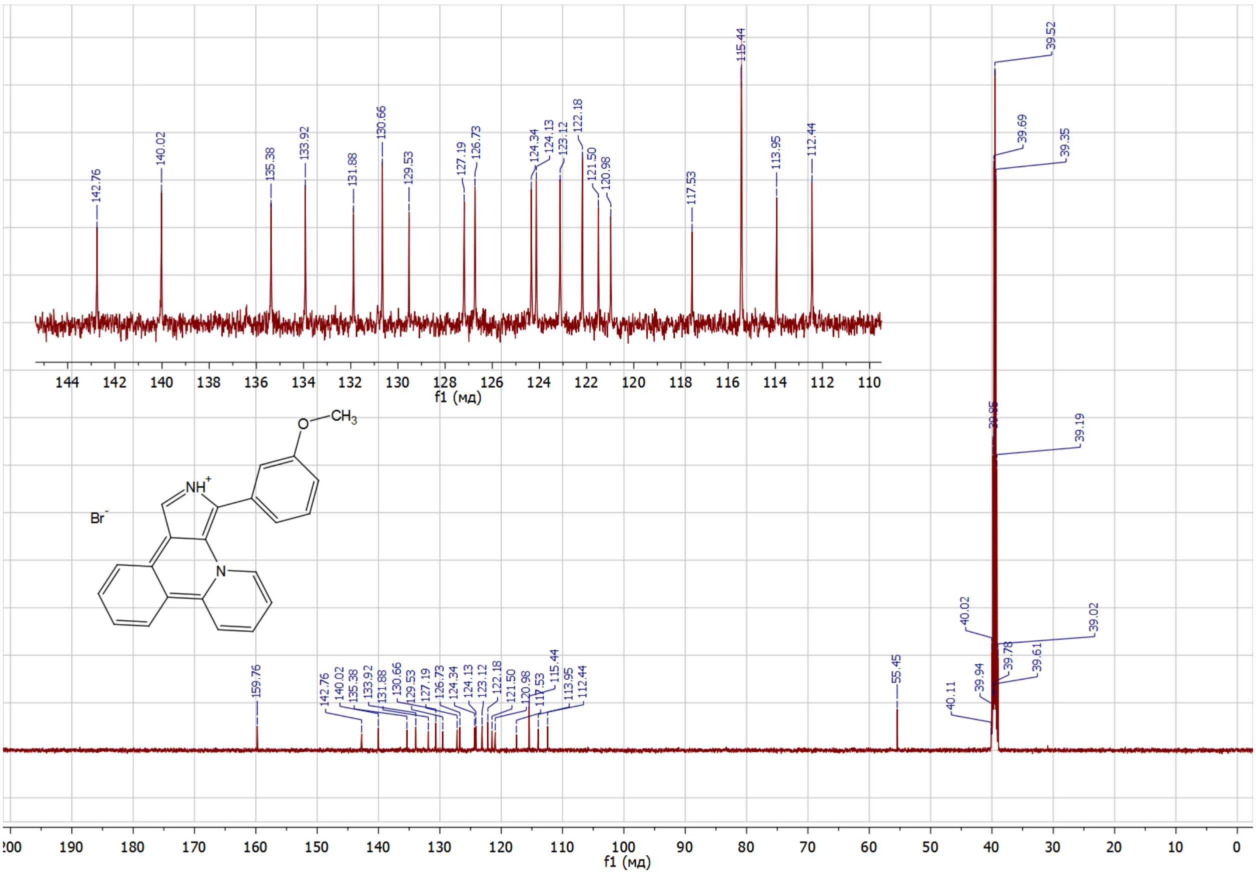

^{13}C DEPT135 NMR (101 MHz, DMSO-d_6) of 3-(3-methoxyphenyl)pyrido[2,1-*a*]pyrrolo[3,4-*c*]isoquinolin-2-ium bromide (**3h**)

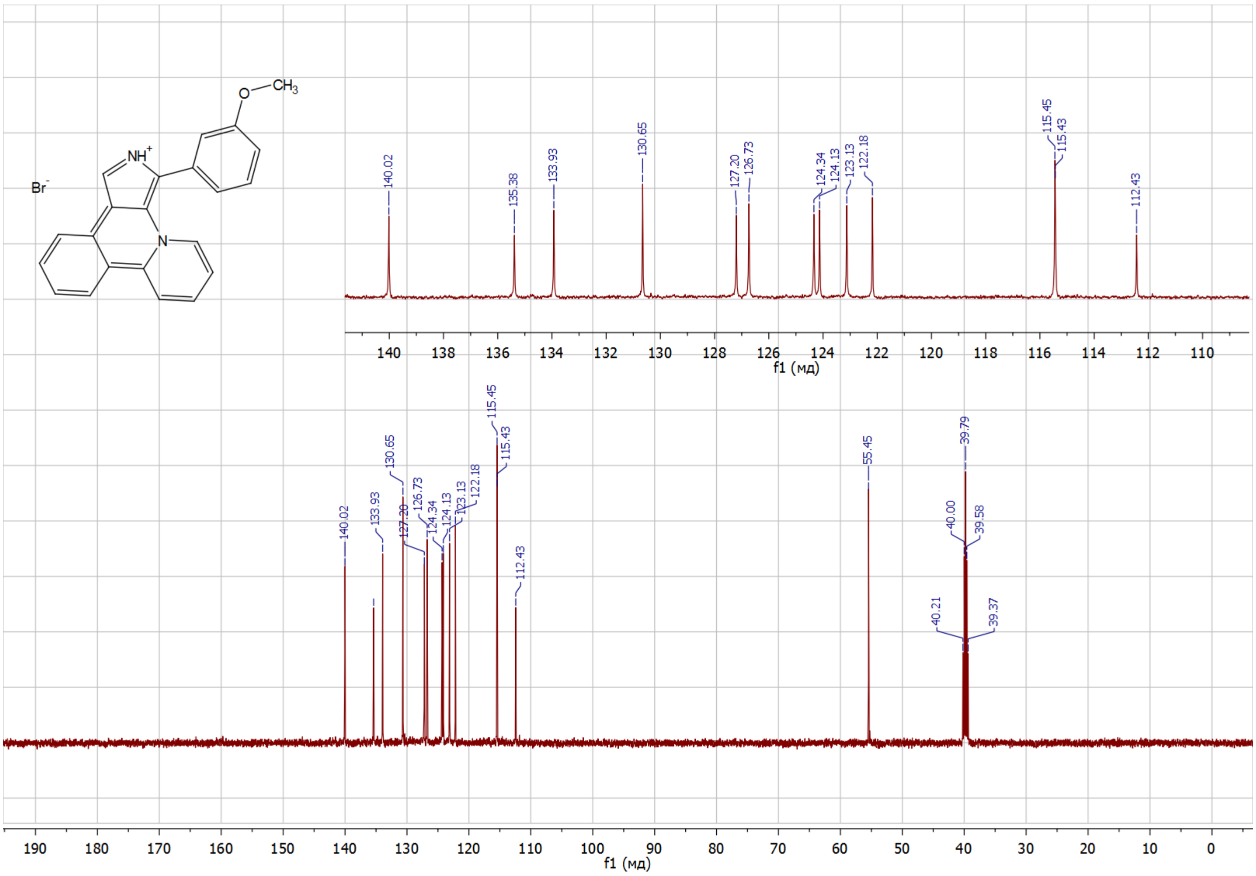

^{1}H NMR (400 MHz, DMSO-d_6) of 3-(2,4-dimethoxyphenyl)pyrido[2,1-*a*]pyrrolo[3,4-*c*]isoquinolin-2-ium bromide (**3i**)

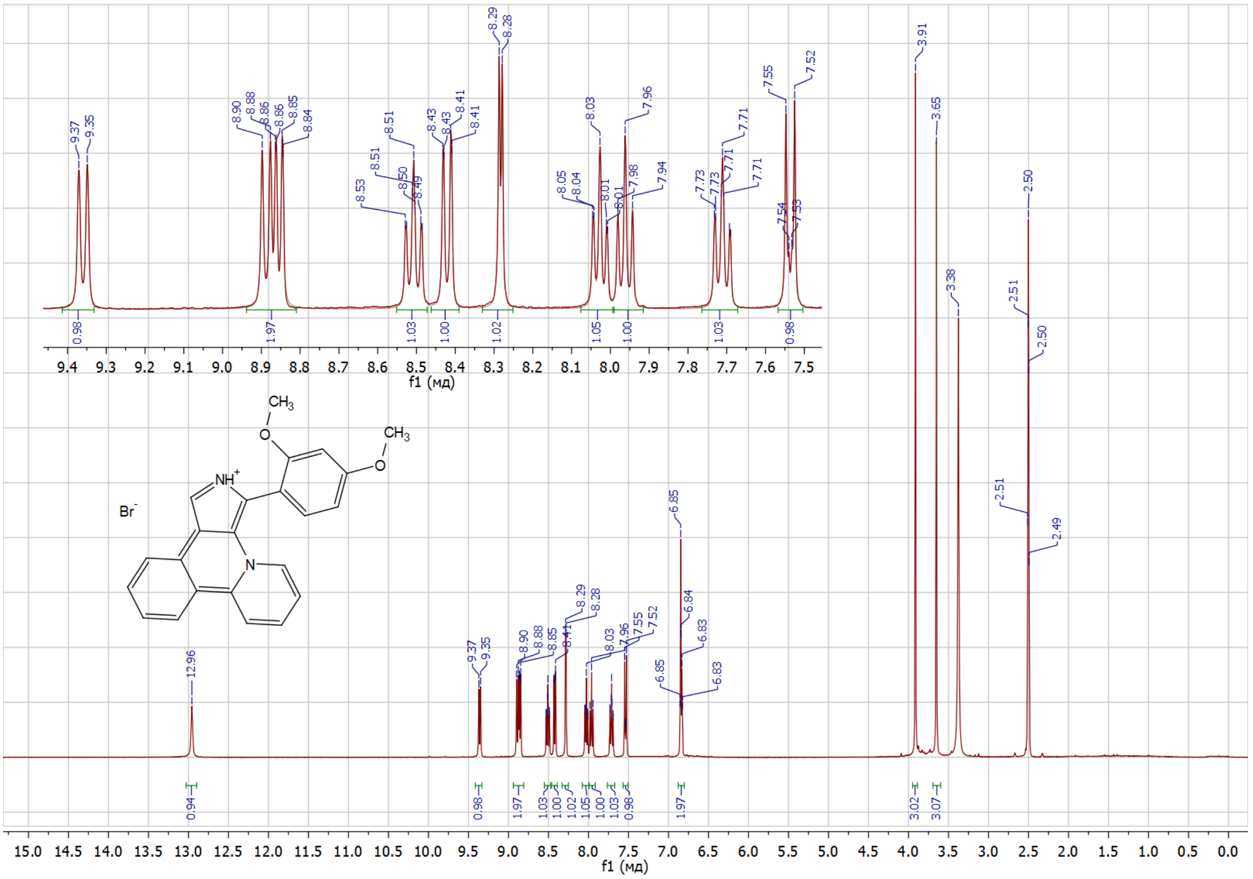

^{13}C{1H} NMR (101 MHz, DMSO-d_6) of 3-(2,4-dimethoxyphenyl)pyrido[2,1-*a*]pyrrolo[3,4-*c*]isoquinolin-2-ium bromide (**3i**)

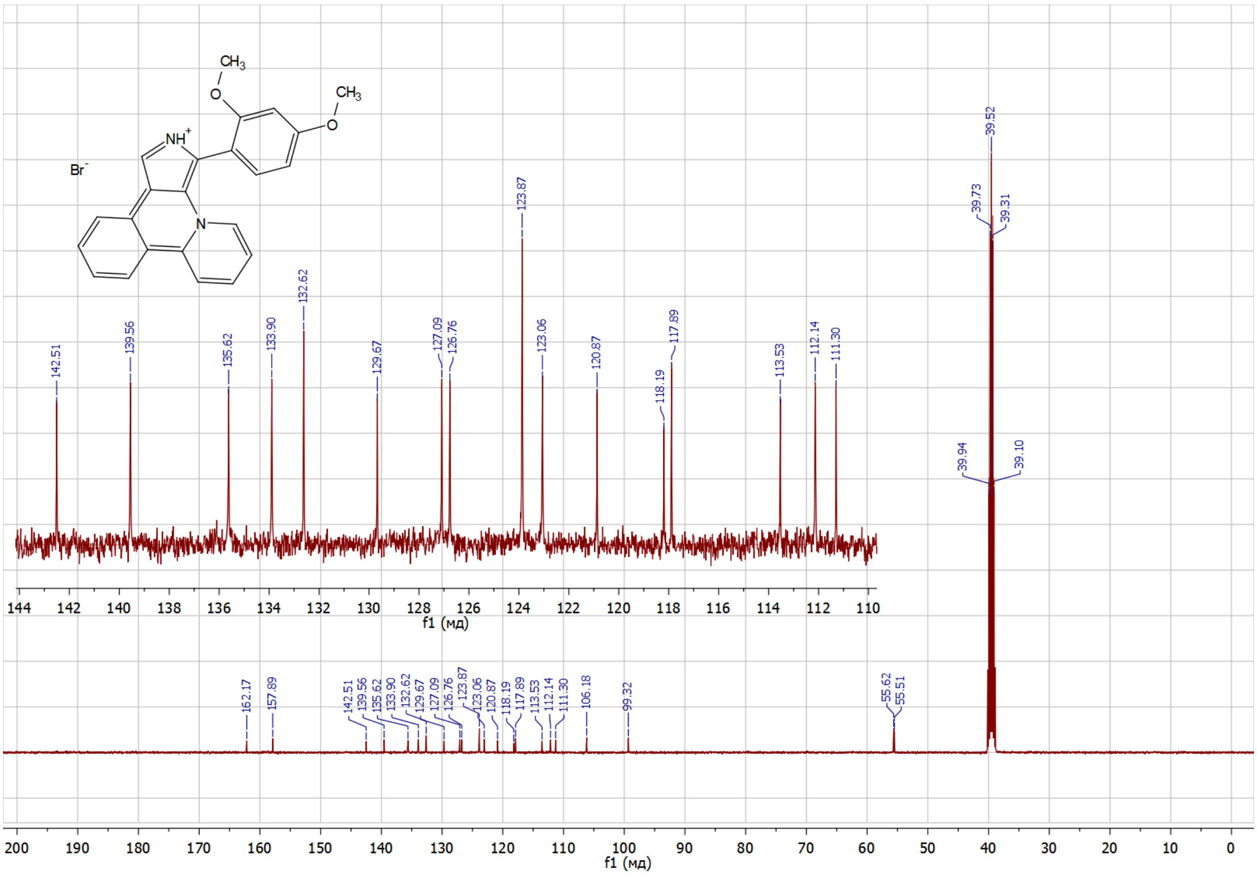

^{13}C DEPT135 NMR (101 MHz, DMSO-d_6) of 3-(2,4-dimethoxyphenyl)pyrido[2,1-*a*]pyrrolo[3,4-*c*]isoquinolin-2-ium bromide (**3i**)

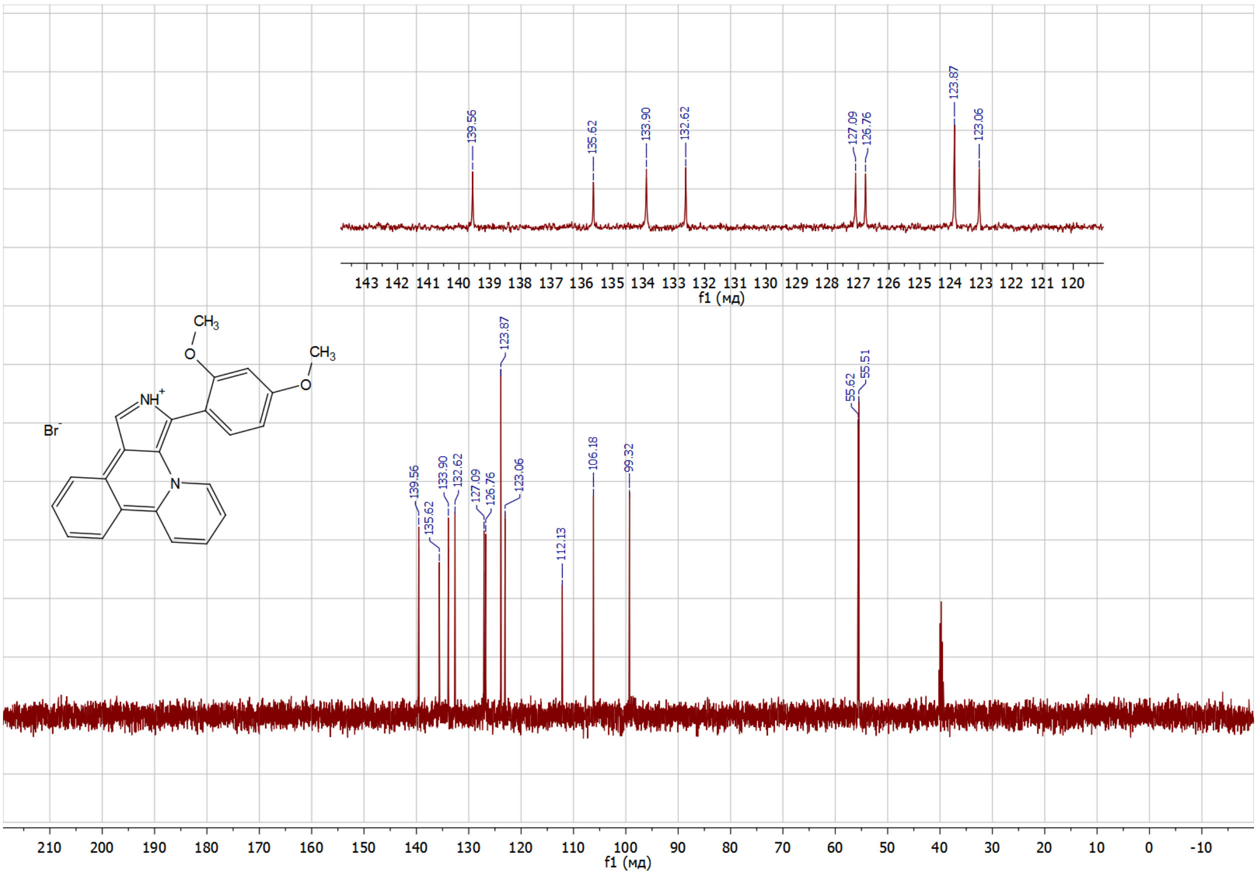

^{1}H NMR (500 MHz, DMSO-d_6) of 3-(4-cyanophenyl)pyrido[2,1-*a*]pyrrolo[3,4-*c*]isoquinolin-2-ium bromide (**3l**)

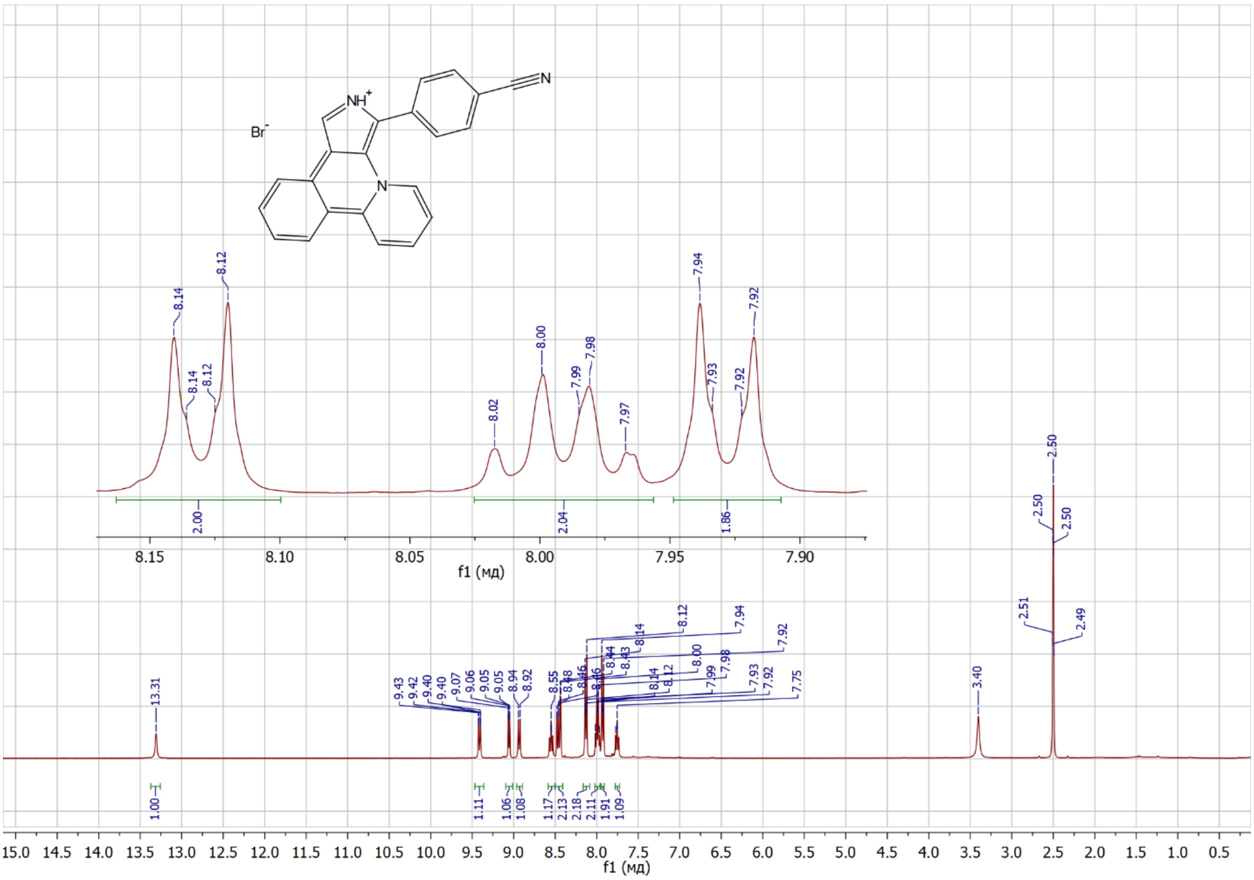

^{13}C{1H} NMR (126 MHz, DMSO-d_6) of 3-(4-cyanophenyl)pyrido[2,1-*a*]pyrrolo[3,4-*c*]isoquinolin-2-ium bromide (**3l**)

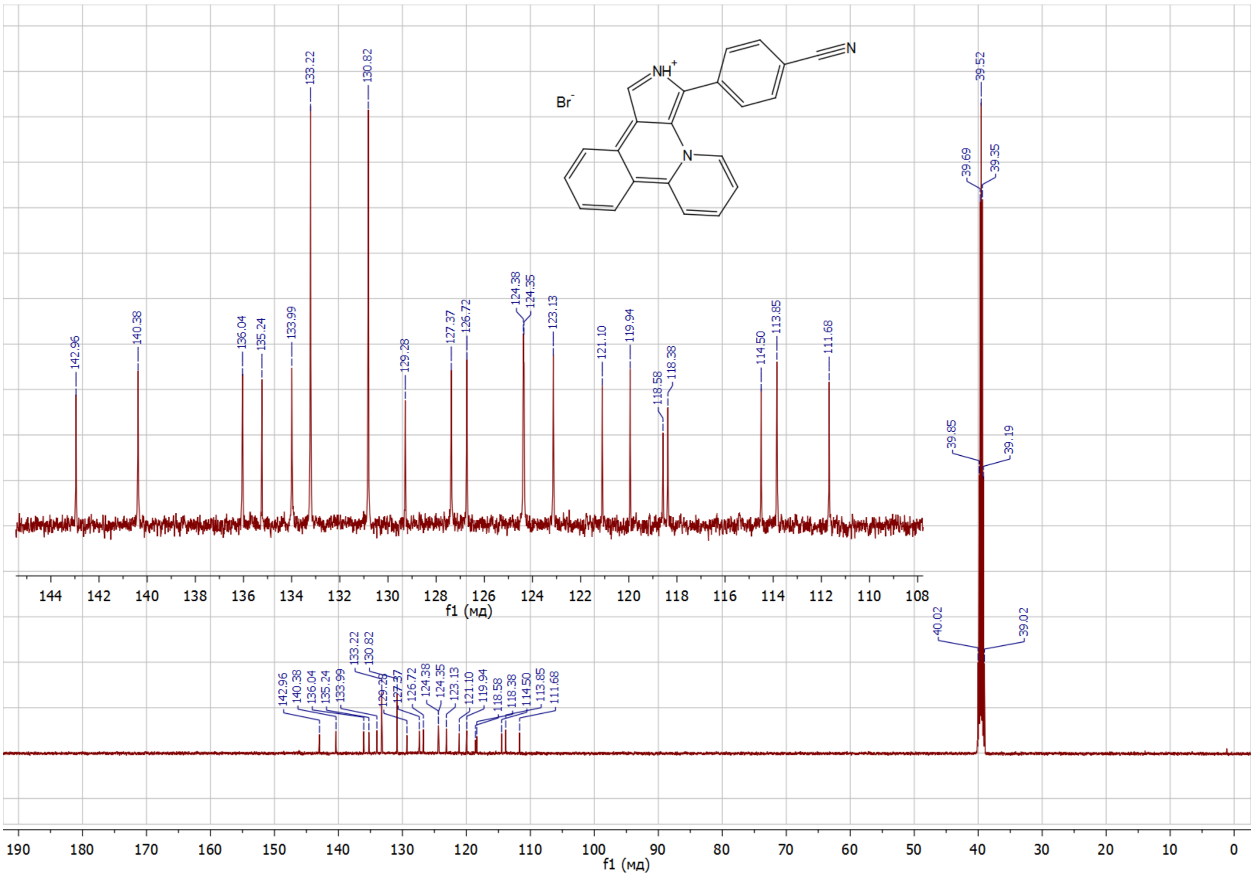

^{13}C DEPT135 NMR (126 MHz, DMSO-d_6) of 3-(4-cyanophenyl)pyrido[2,1-*a*]pyrrolo[3,4-*c*]isoquinolin-2-ium bromide (**3l**)

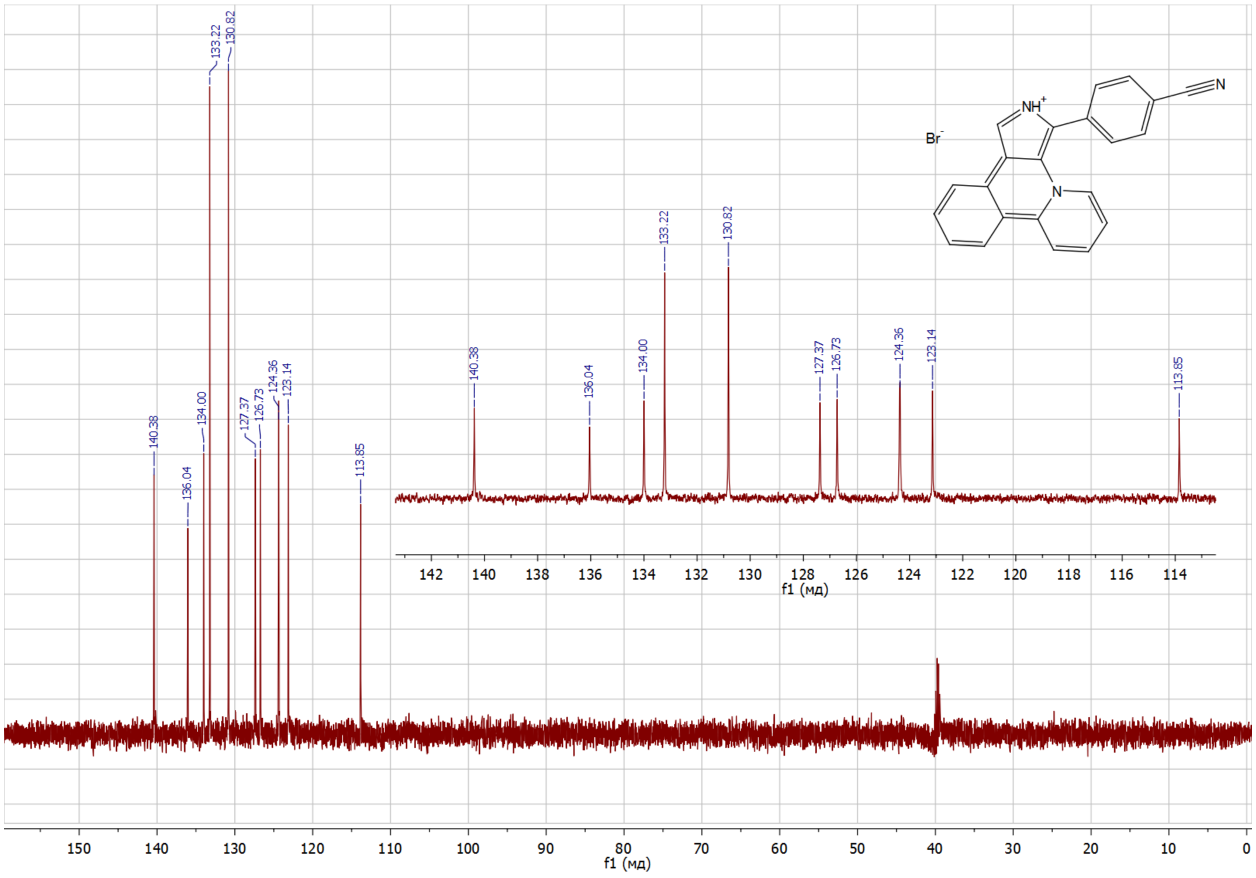

^{1}H NMR (400 MHz, DMSO-d_6) of 3-(pyridin-2-yl)pyrido[2,1-*a*]pyrrolo[3,4-*c*]isoquinolin-2-ium iodide (**3m**)

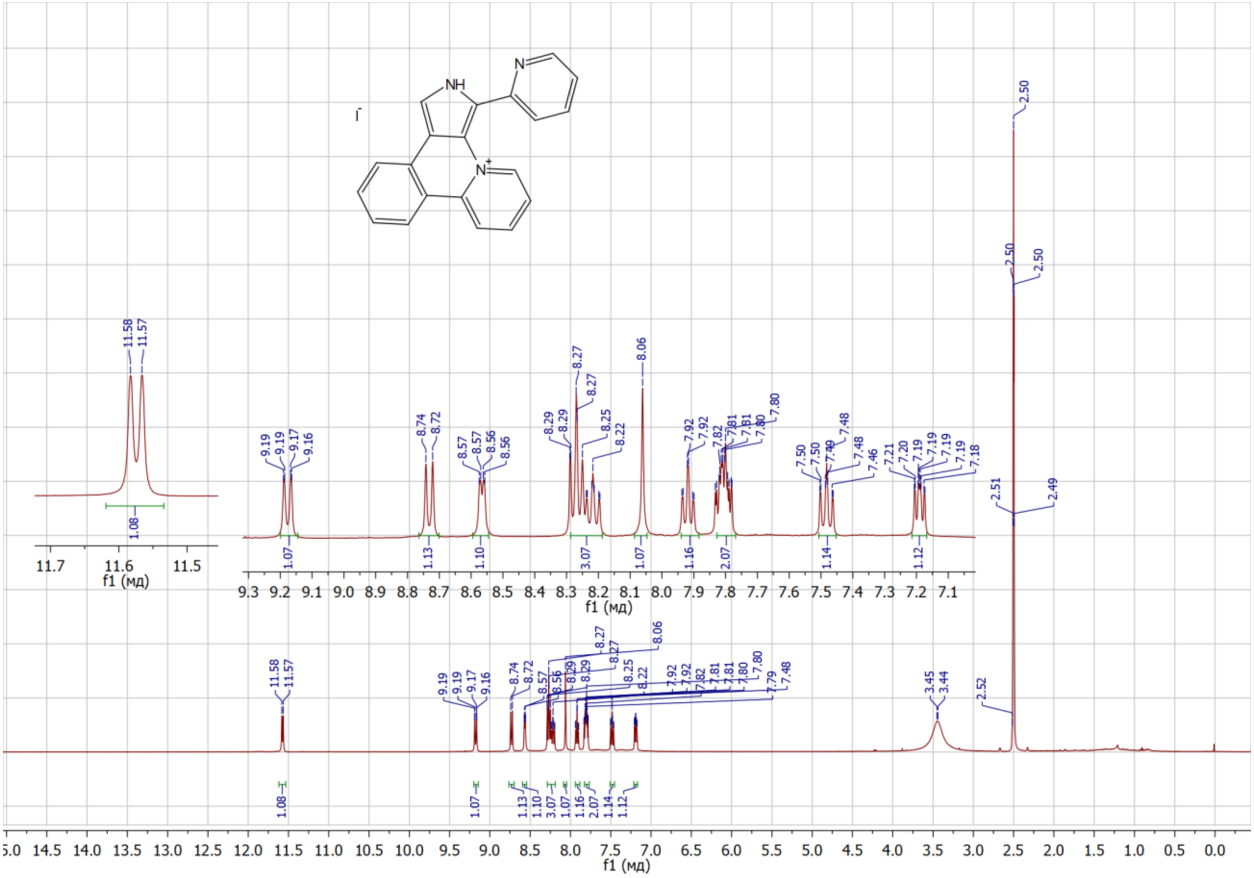

^{13}C{1H} NMR (101 MHz, DMSO-d_6) of 3-(pyridin-2-yl)pyrido[2,1-*a*]pyrrolo[3,4-*c*]isoquinolin-2-ium iodide (**3m**)

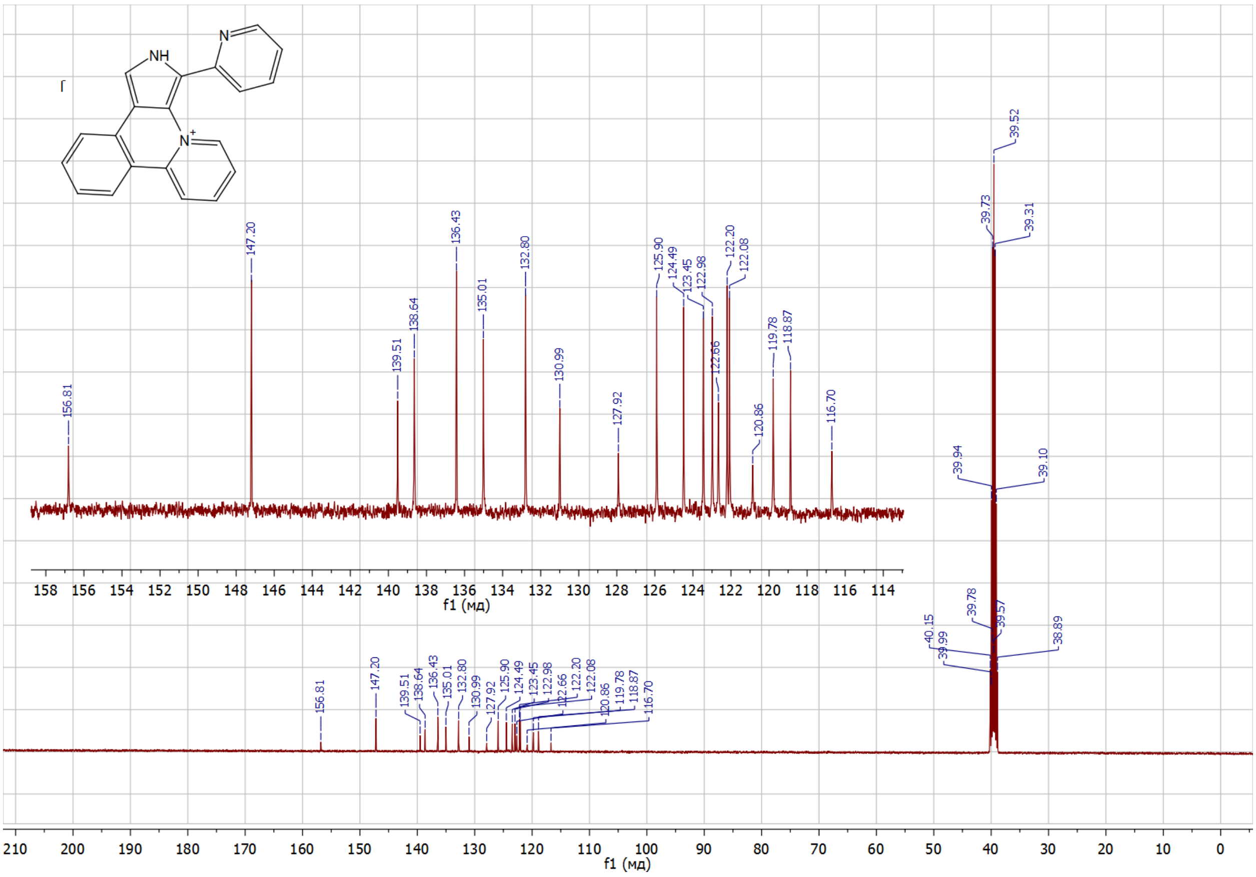

^{13}C DEPT135 NMR (101 MHz, DMSO-d_6) of 3-(pyridin-2-yl)pyrido[2,1-*a*]pyrrolo[3,4-*c*]isoquinolin-2-ium iodide (**3m**)

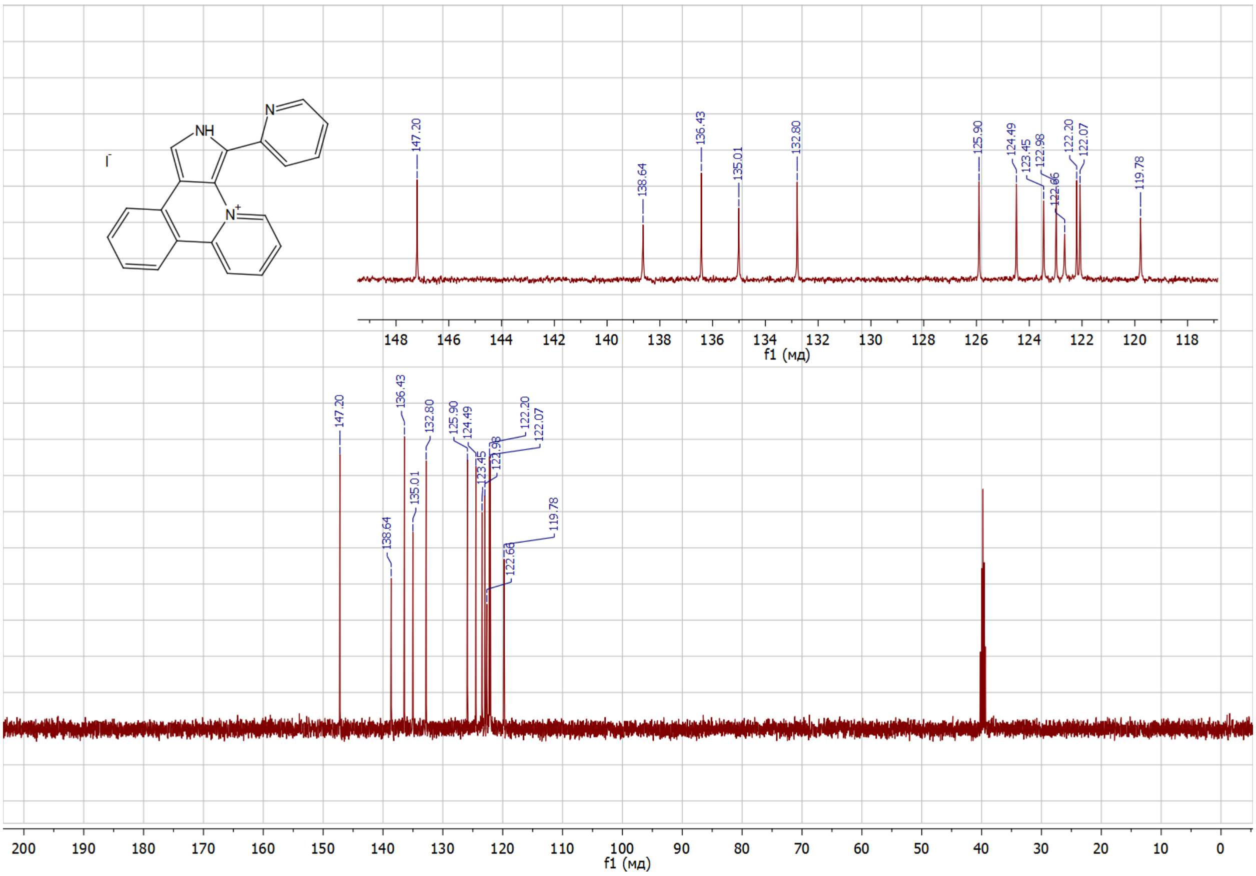

^{1}H NMR (400 MHz, DMSO-d_6) of 7-methyl-3-phenylpyrido[2,1-*a*]pyrrolo[3,4-*c*]isoquinolin-2-ium bromide (**3n**)

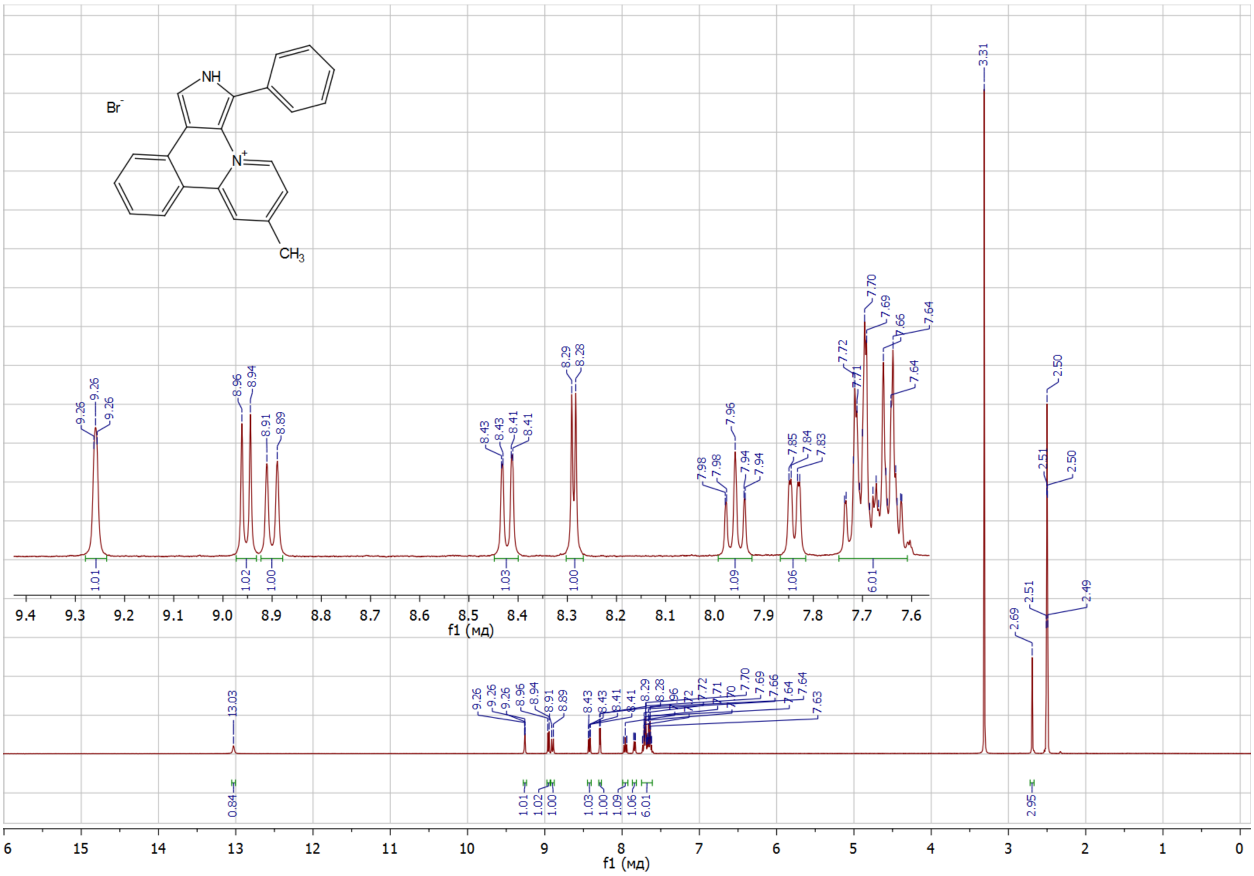

$^{13}C\{1H\}$ NMR (101 MHz, DMSO-d_6) of 7-methyl-3-phenylpyrido[2,1-*a*]pyrrolo[3,4-*c*]isoquinolin-2-ium bromide (**3n**)

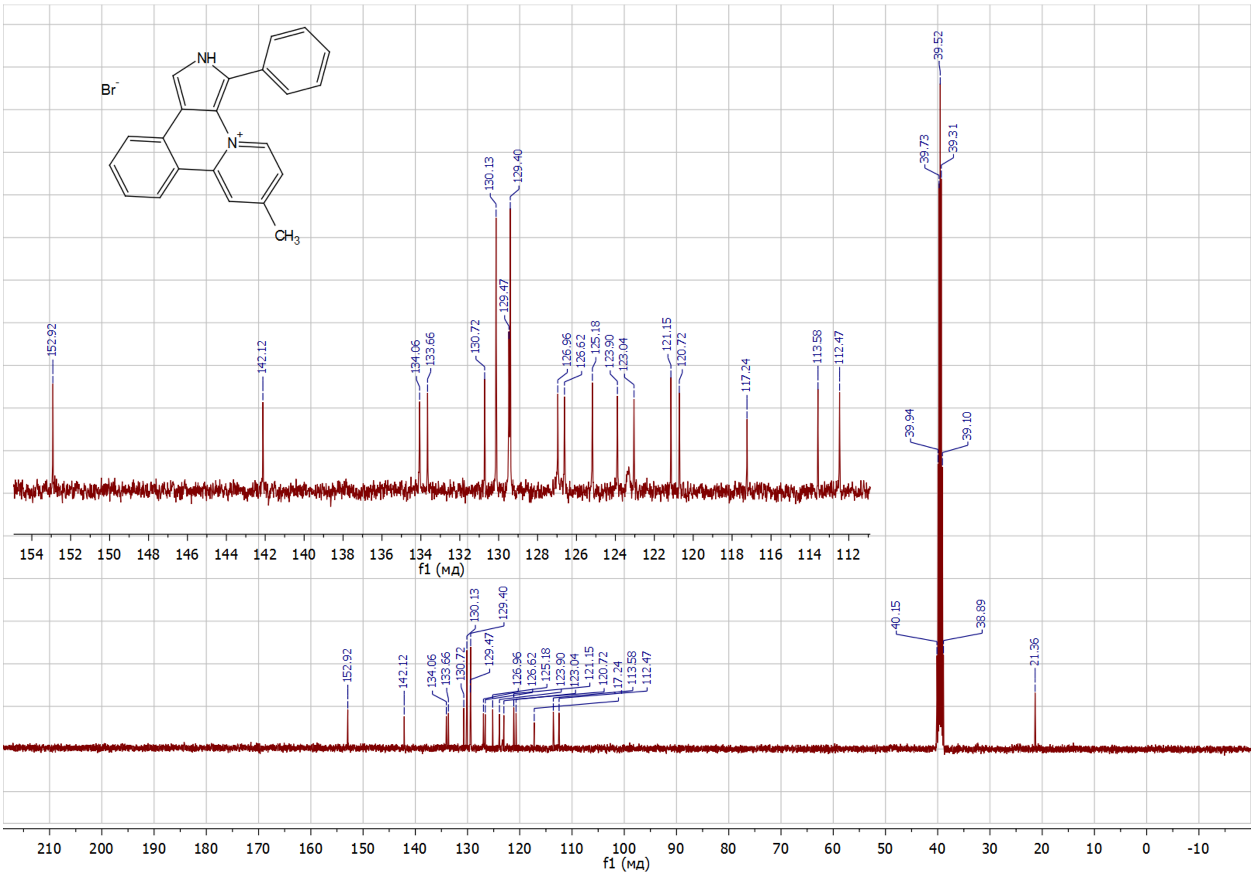

^{13}C DEPT135 NMR (101 MHz, DMSO-d_6) of 7-methyl-3-phenylpyrido[2,1-*a*]pyrrolo[3,4-*c*]isoquinolin-2-ium bromide (**3n**)

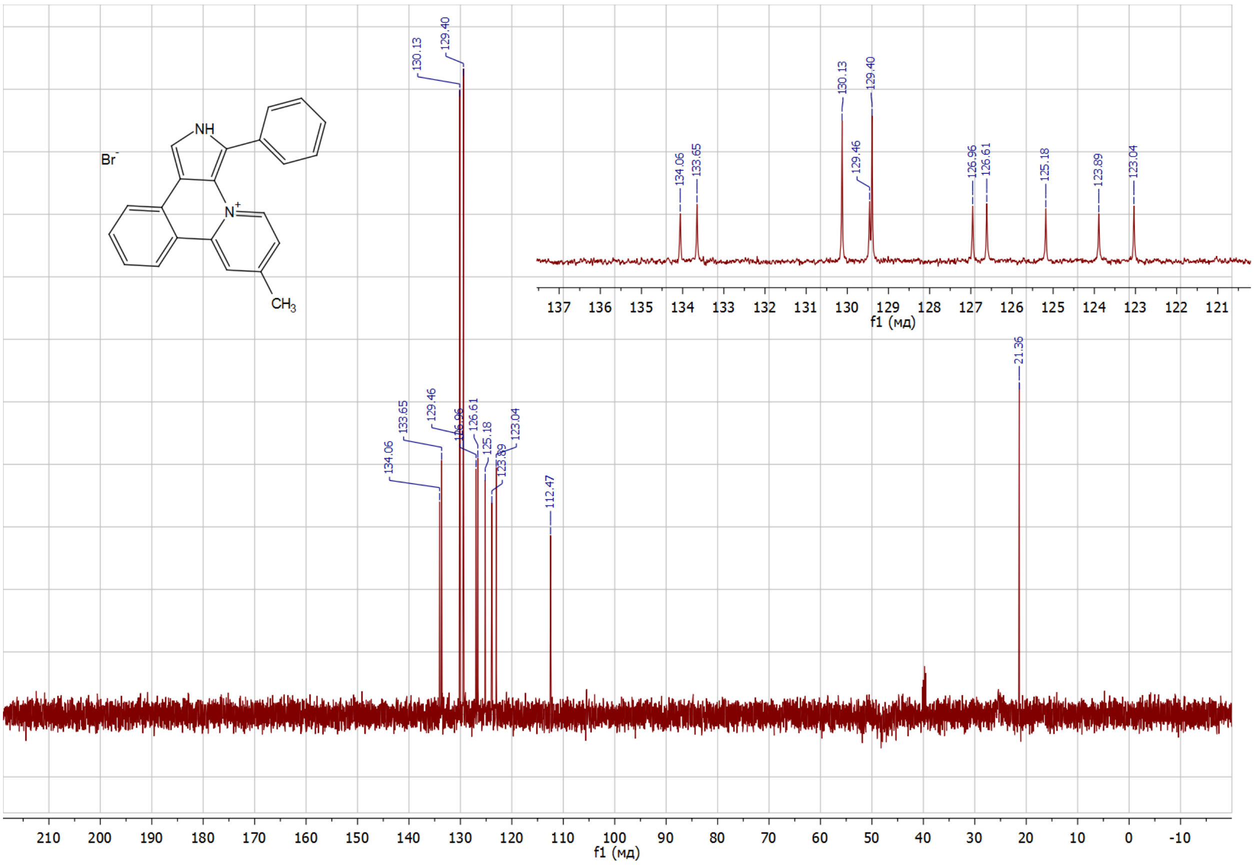

^{1}H NMR (400 MHz, DMSO-d_6) of 7-methoxy-3-phenylpyrido[2,1-*a*]pyrrolo[3,4-*c*]isoquinolin-2-ium bromide (**3o**)

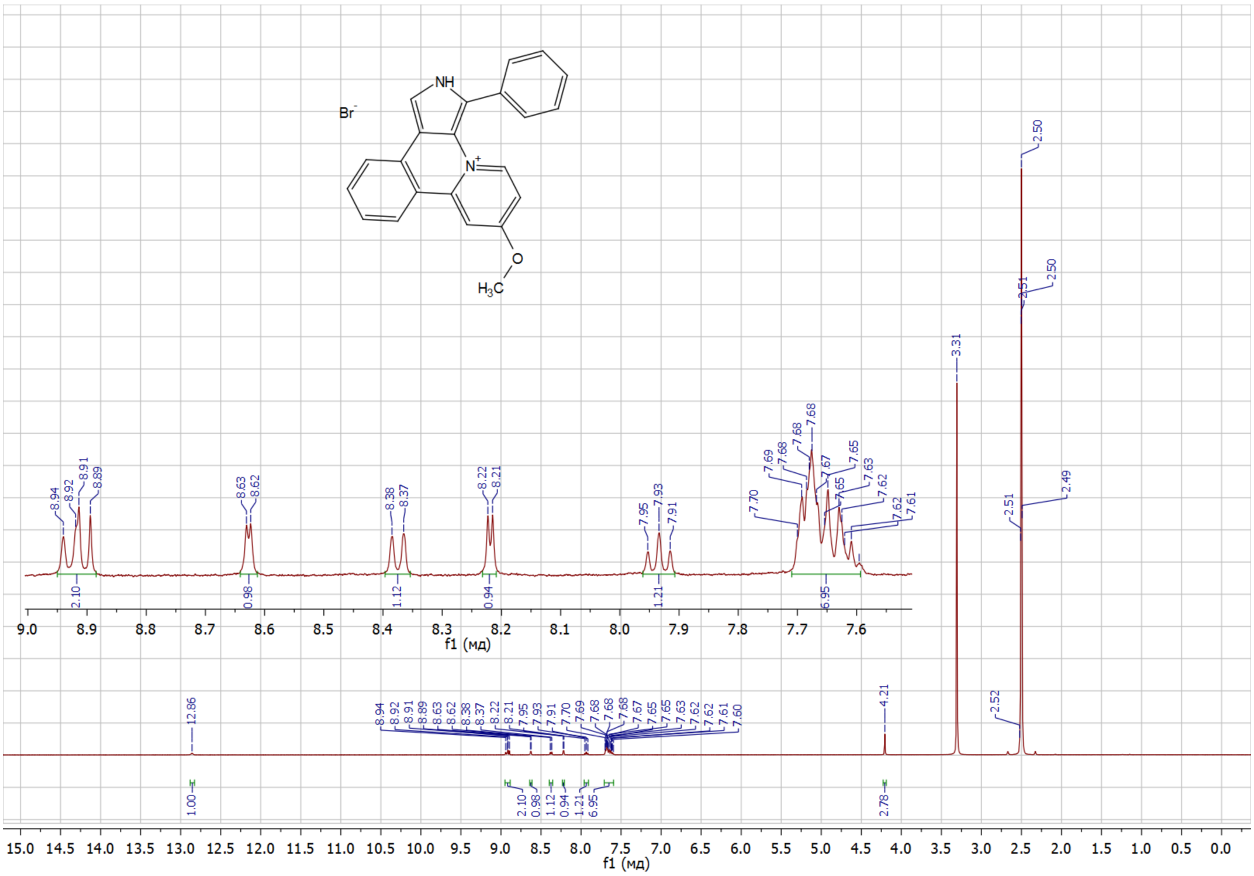

^{13}C{1H} NMR (101 MHz, DMSO-d_6) of 7-methoxy-3-phenylpyrido[2,1-*a*]pyrrolo[3,4-*c*]isoquinolin-2-ium bromide (**3o**)

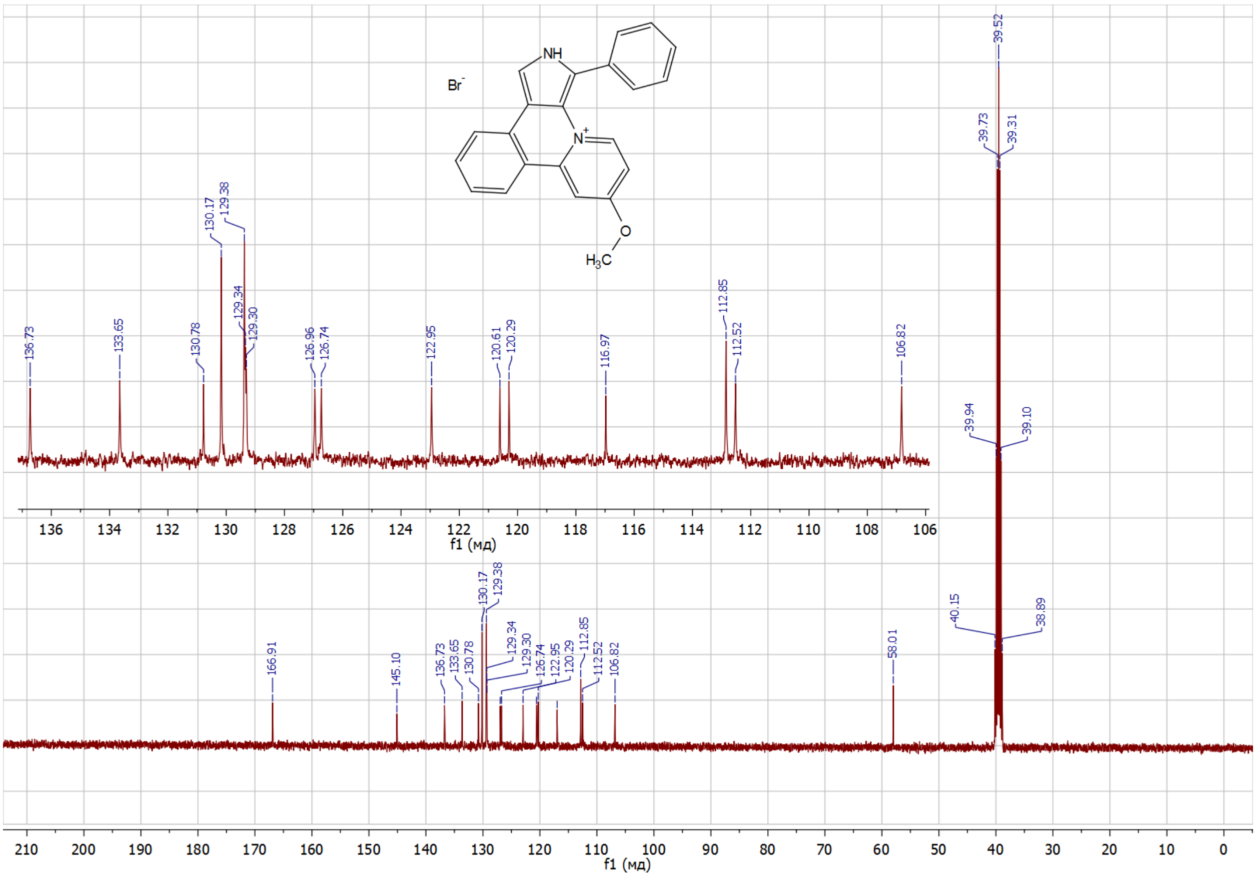

^{13}C DEPT135 NMR (101 MHz, DMSO-d_6) of 7-methoxy-3-phenylpyrido[2,1-*a*]pyrrolo[3,4-*c*]isoquinolin-2-ium bromide (**3o**)

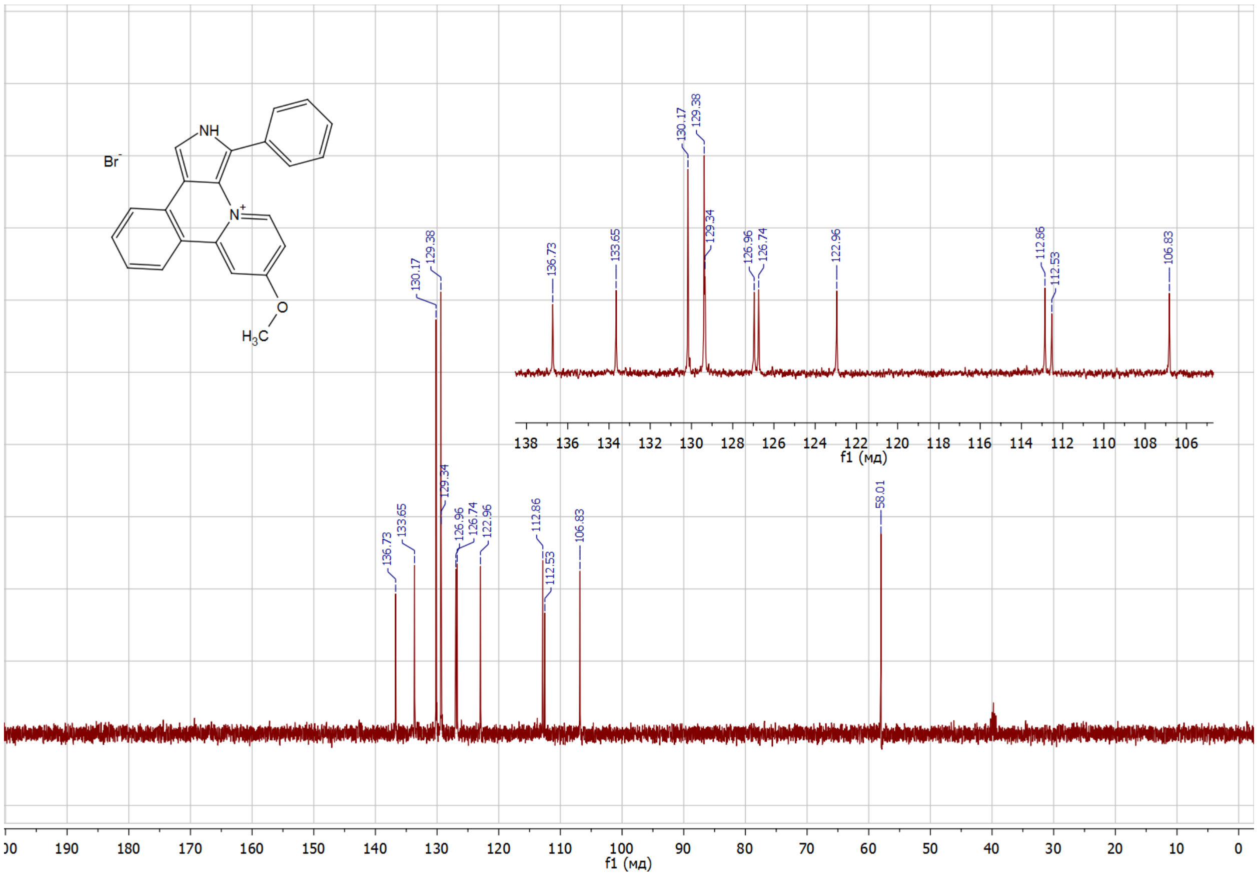

^{1}H NMR (400 MHz, DMSO-d_6) of 7-(dimethylamino)-3-phenylpyrido[2,1-*a*]pyrrolo[3,4-*c*]isoquinolin-2-ium bromide (**3p**)

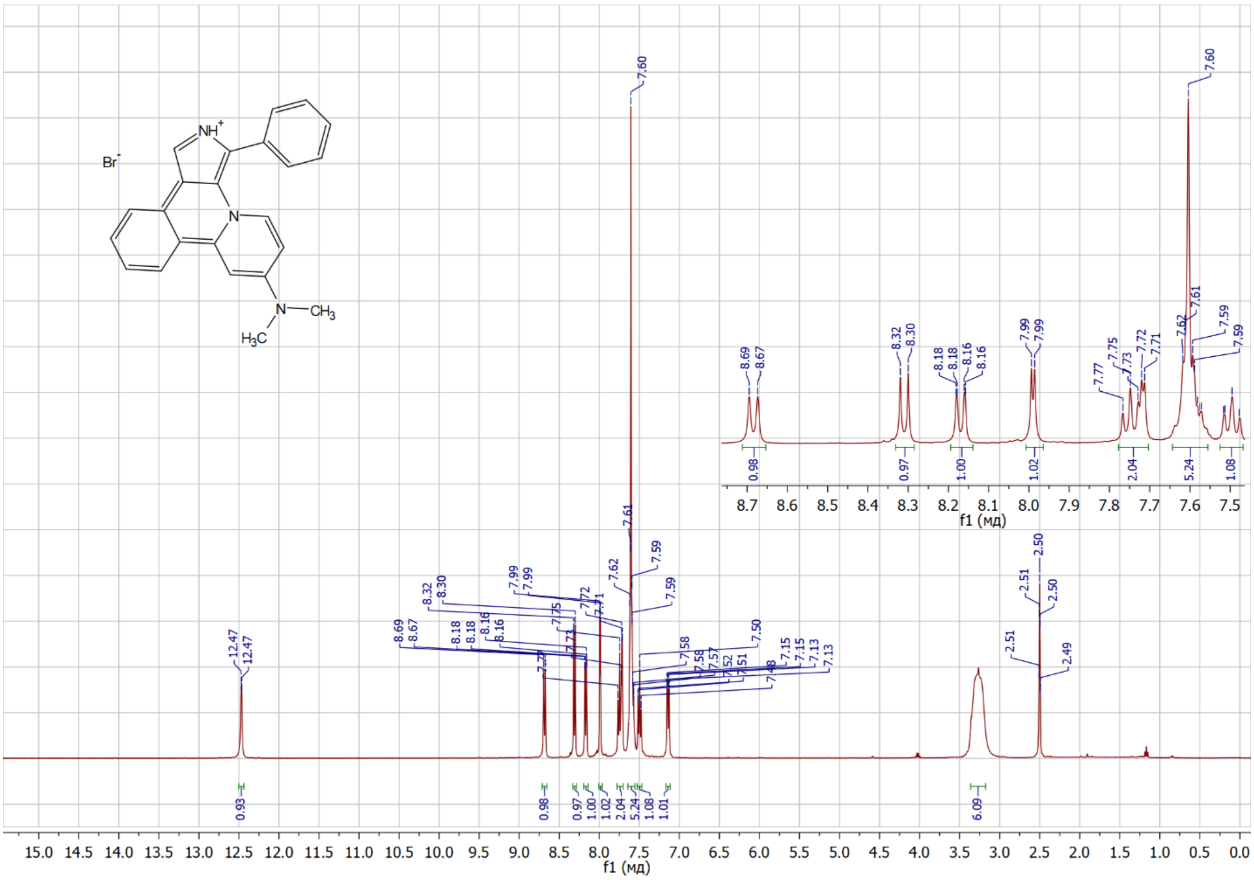

^{13}C{1H} NMR (101 MHz, DMSO-d_6) of 7-(dimethylamino)-3-phenylpyrido[2,1-*a*]pyrrolo[3,4-*c*]isoquinolin-2-ium bromide (**3p**)

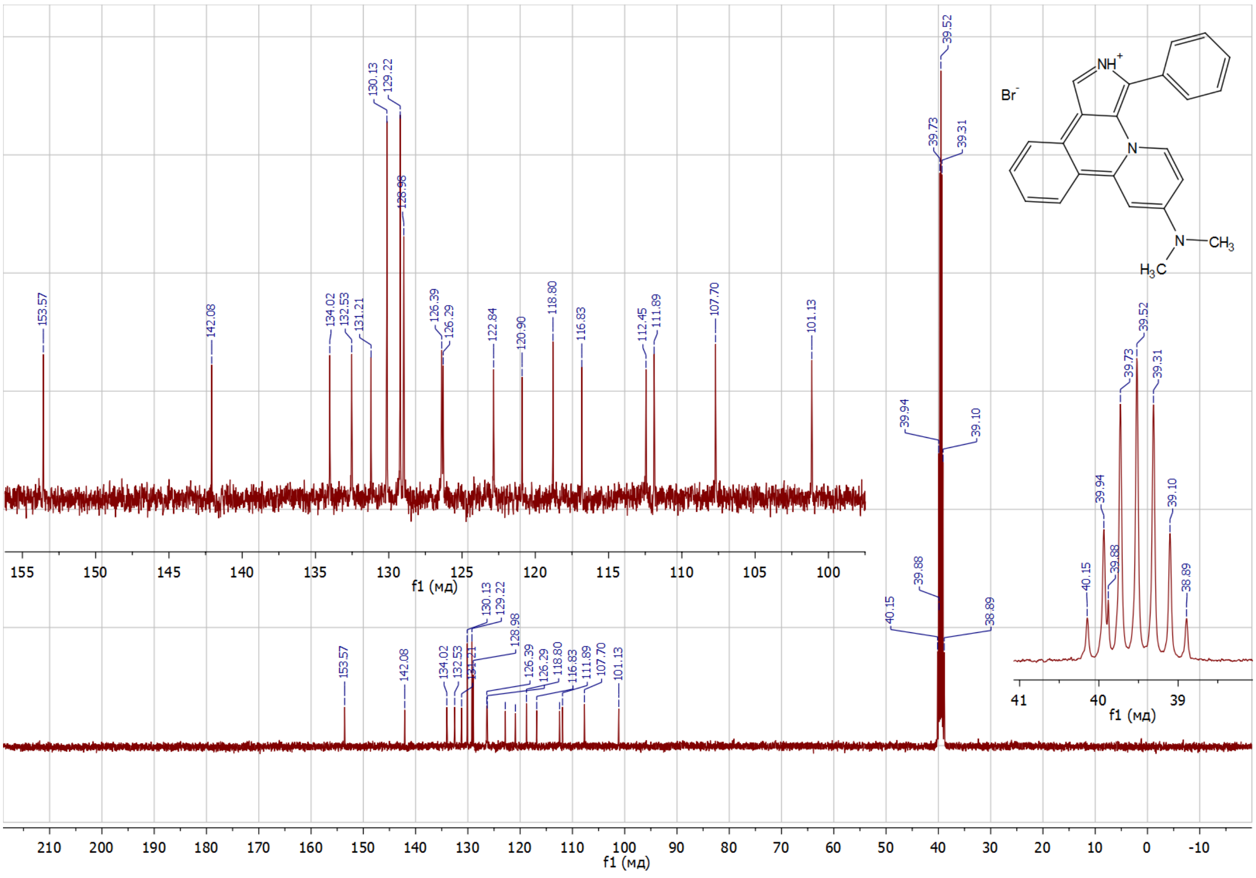

^{13}C DEPT135 NMR (101 MHz, DMSO-d_6) of 7-(dimethylamino)-3-phenylpyrido[2,1-*a*]pyrrolo[3,4-*c*]isoquinolin-2-ium bromide (**3p**)

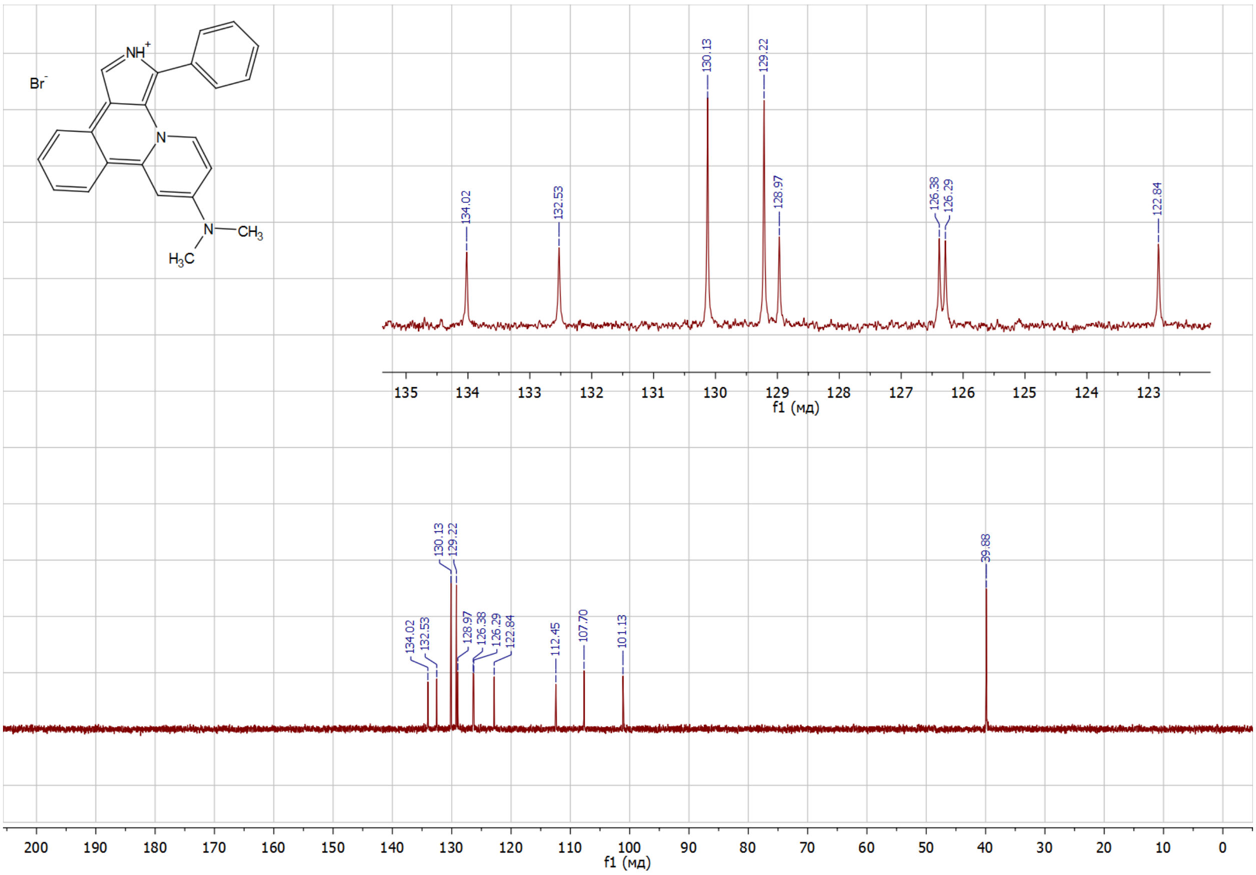

^{1}H NMR (400 MHz, DMSO-d_6) of 3,7-diphenylpyrido[2,1-*a*]pyrrolo[3,4-*c*]isoquinolin-2-ium bromide (**3q**)

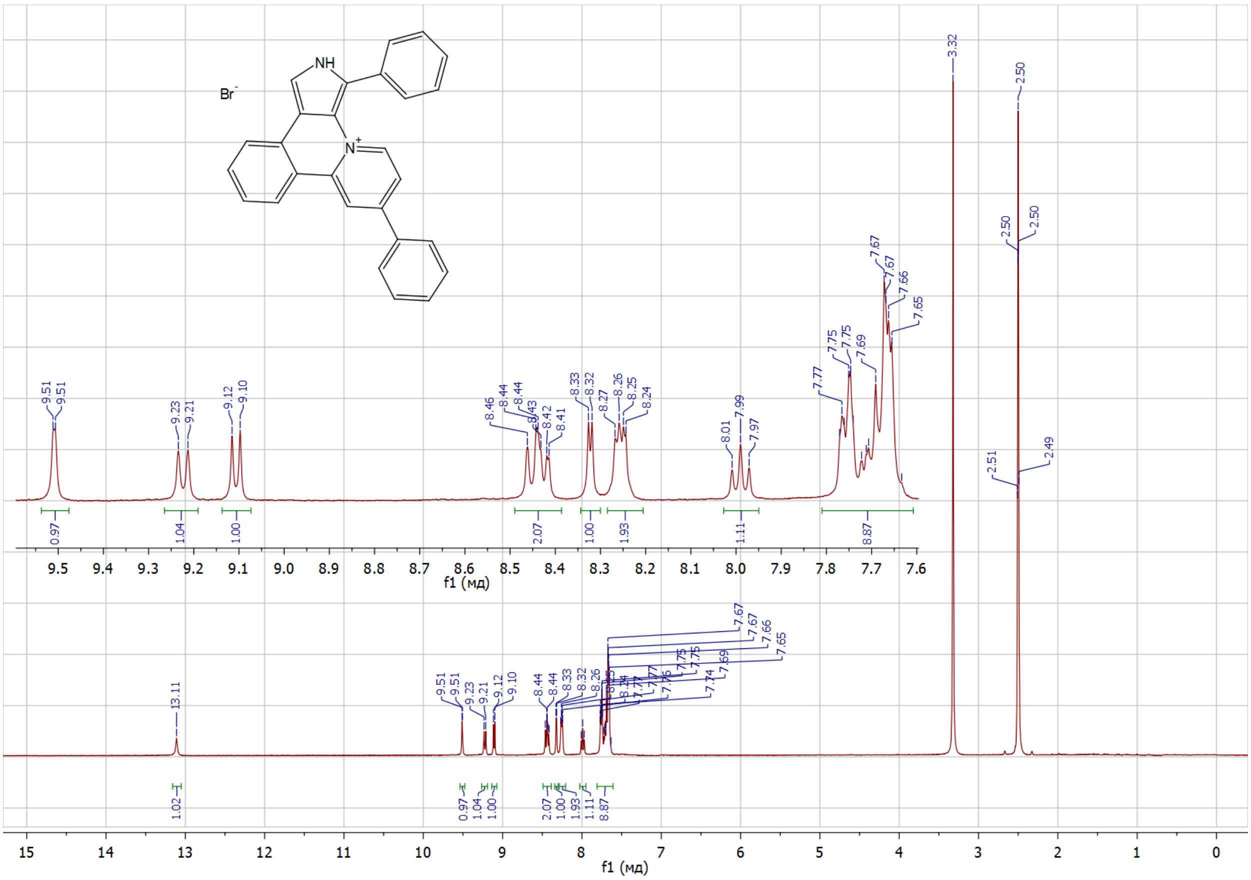

^{13}C{1H} NMR (101 MHz, DMSO-d_6) of 3,7-diphenylpyrido[2,1-*a*]pyrrolo[3,4-*c*]isoquinolin-2-ium bromide (**3q**)

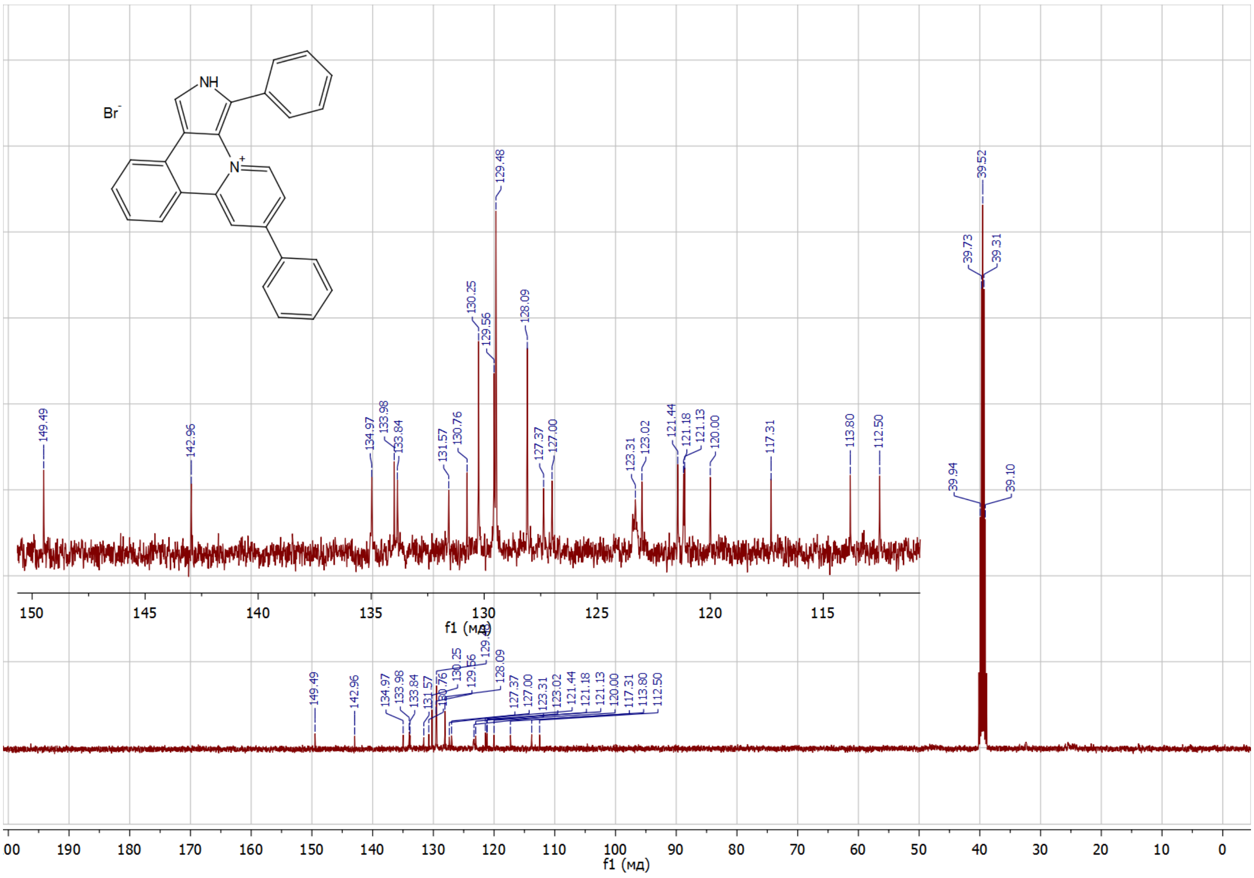

^{13}C DEPT135 NMR (101 MHz, DMSO-d_6) of 3,7-diphenylpyrido[2,1-*a*]pyrrolo[3,4-*c*]isoquinolin-2-ium bromide (**3q**)

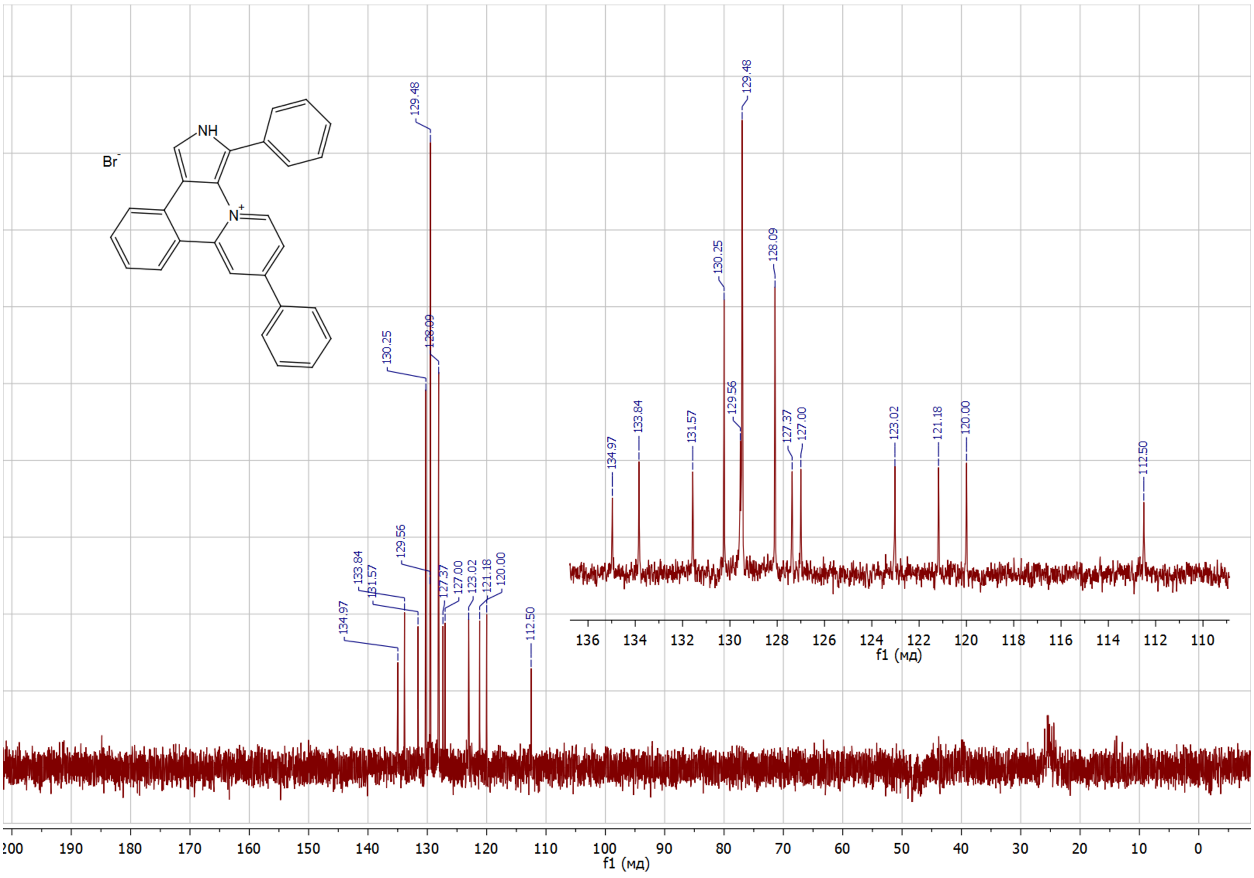

^{1}H NMR (500 MHz, DMSO-d_6) of 7-(4-methoxyphenyl)-3-phenylpyrido[2,1-*a*]pyrrolo[3,4-*c*]isoquinolin-2-ium bromide (**3r**)

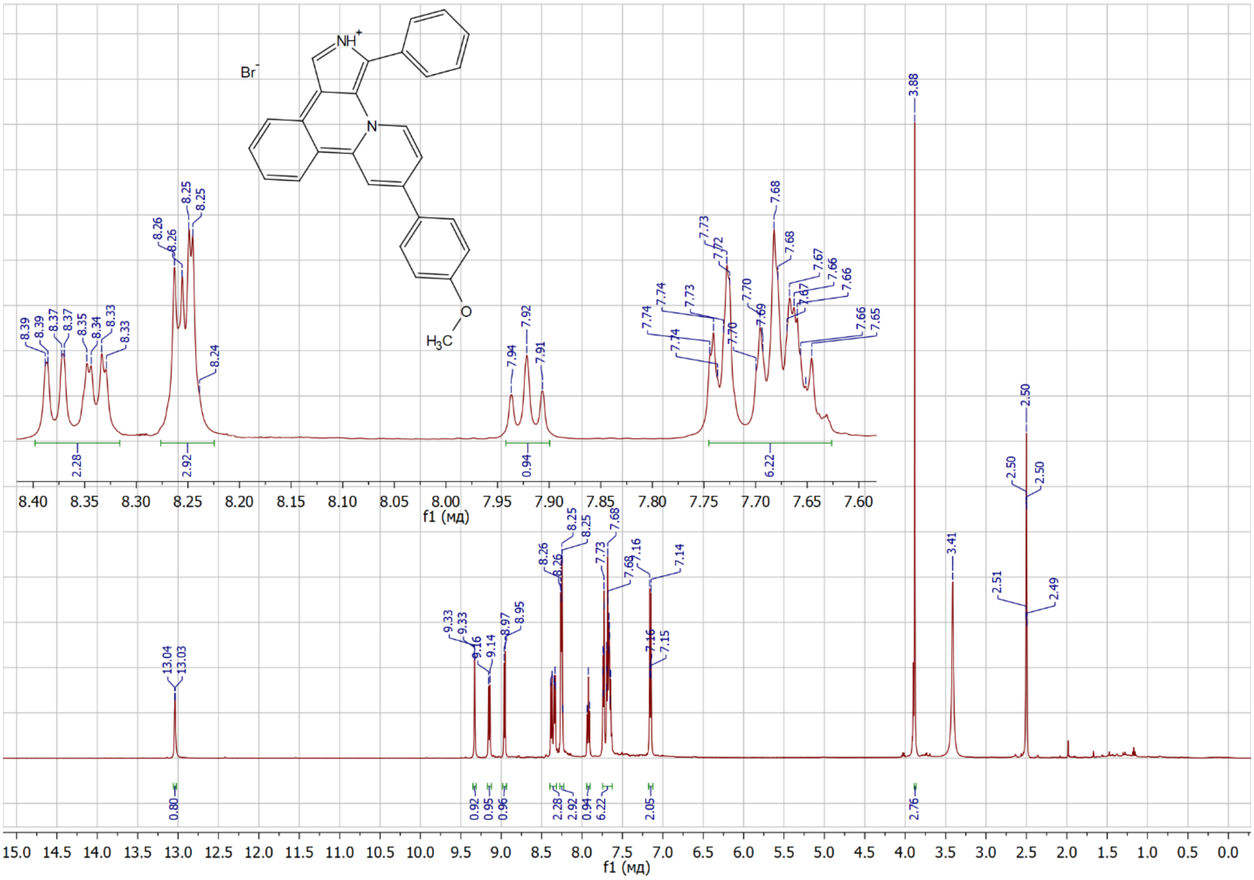

^{13}C{1H} NMR (126 MHz, DMSO-d_6) of 7-(4-methoxyphenyl)-3-phenylpyrido[2,1-*a*]pyrrolo[3,4-*c*]isoquinolin-2-ium bromide (**3r**)

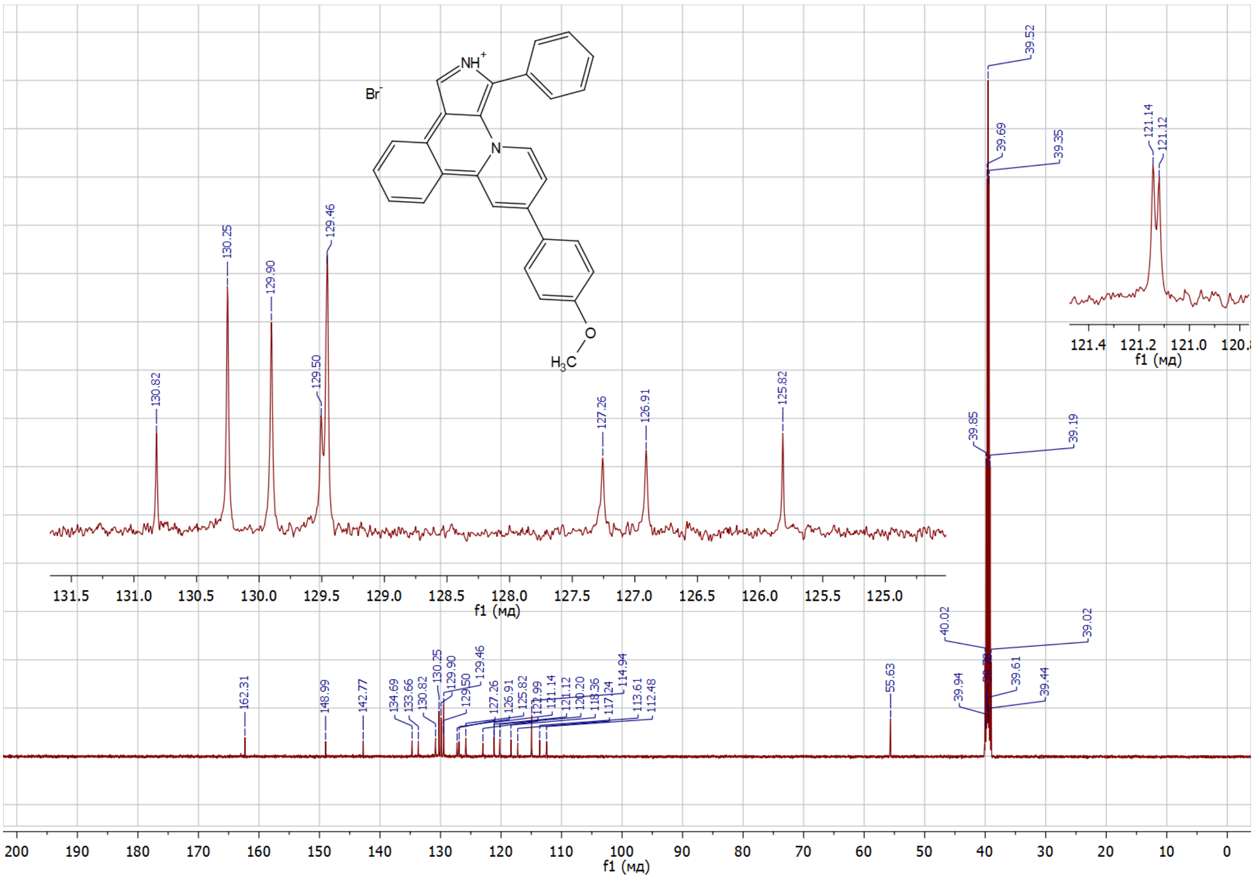

^{13}C DEPT135 NMR (126 MHz, DMSO-d_6) of 7-(4-methoxyphenyl)-3-phenylpyrido[2,1-*a*]pyrrolo[3,4-*c*]isoquinolin-2-ium bromide (**3r**)

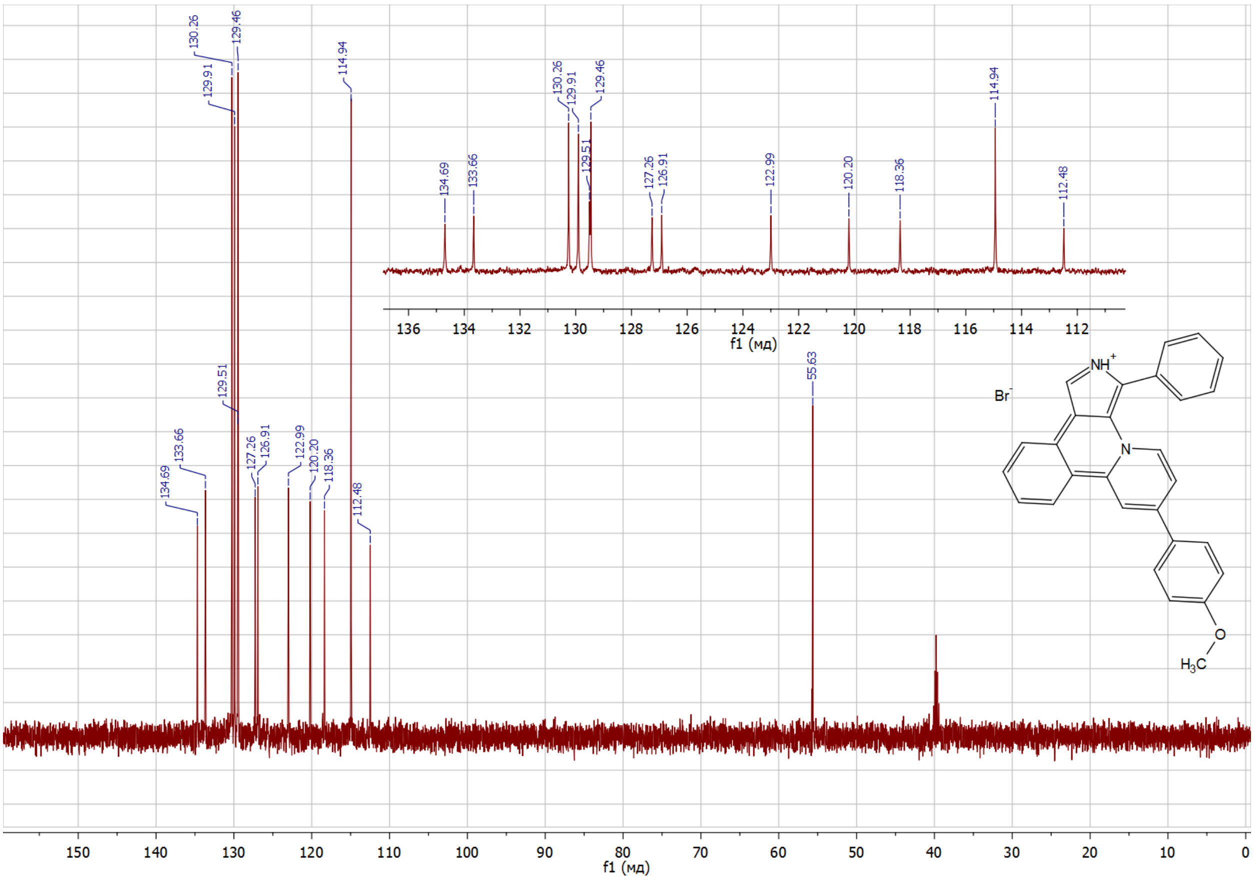

^{1}H NMR (400 MHz, DMSO-d_6) of 6,8-dimethyl-3-phenylpyrido[2,1-*a*]pyrrolo[3,4-*c*]isoquinolin-2-ium bromide (**3s**)

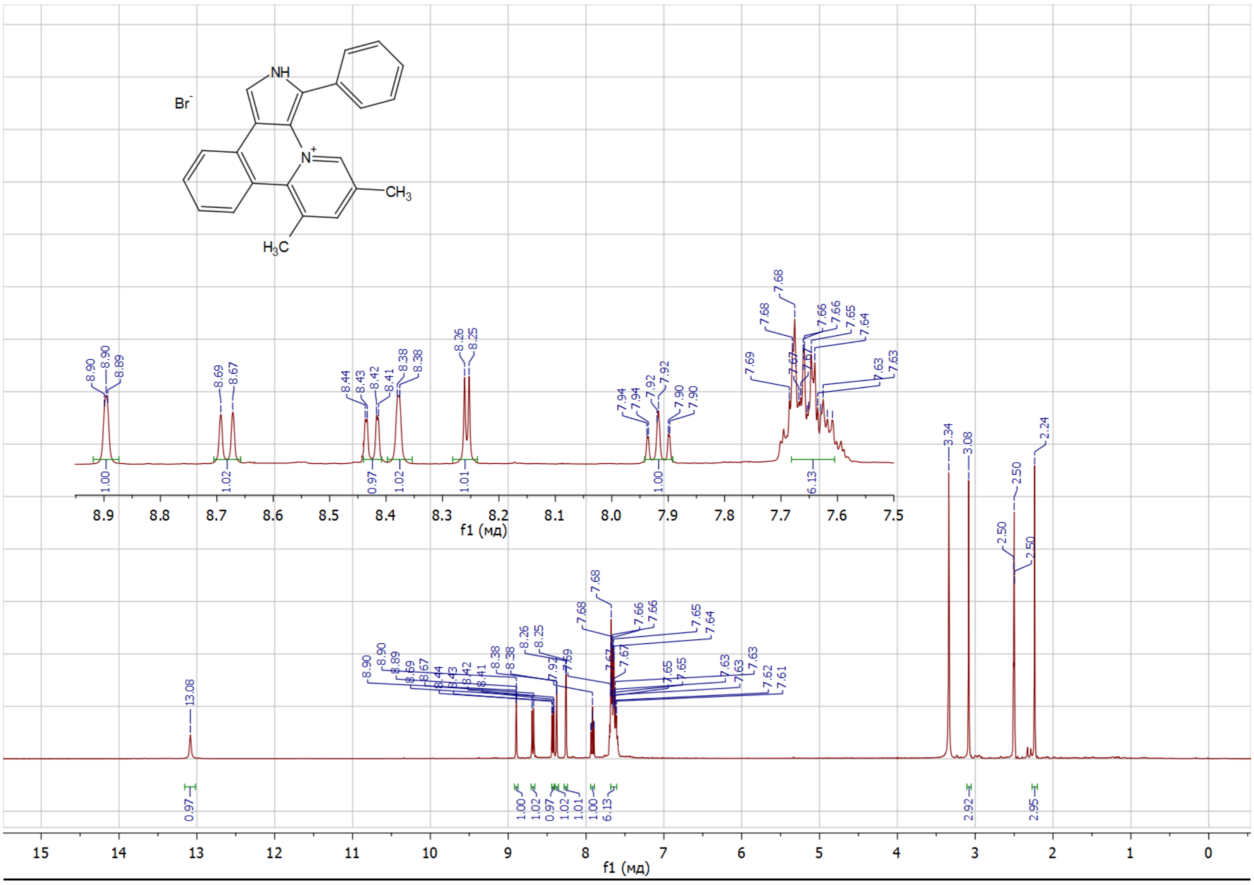

^{13}C{1H} NMR (101 MHz, DMSO-d_6) of 6,8-dimethyl-3-phenylpyrido[2,1-*a*]pyrrolo[3,4-*c*]isoquinolin-2-ium bromide (**3s**)

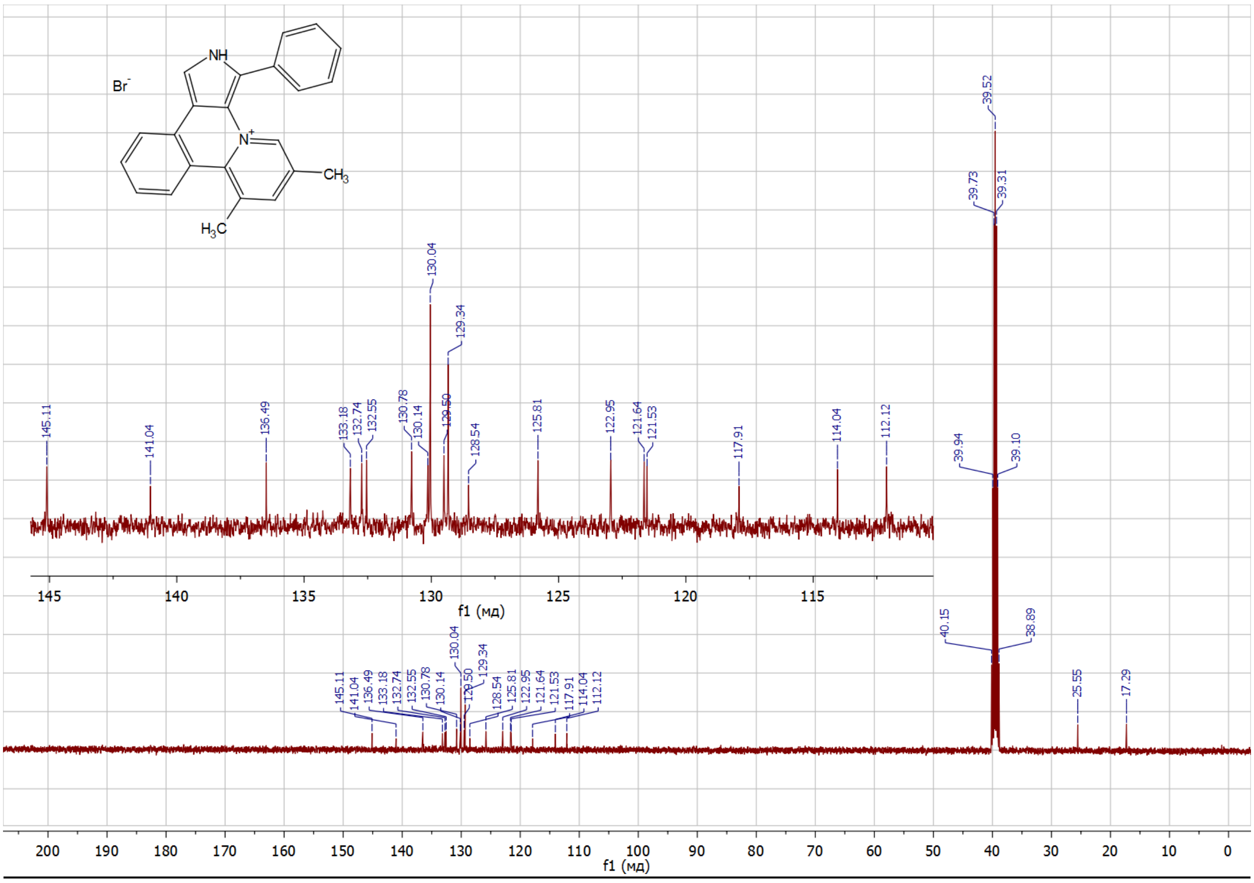

^{13}C DEPT135 NMR (101 MHz, DMSO-d_6) of 6,8-dimethyl-3-phenylpyrido[2,1-*a*]pyrrolo[3,4-*c*]isoquinolin-2-ium bromide (**3s**)

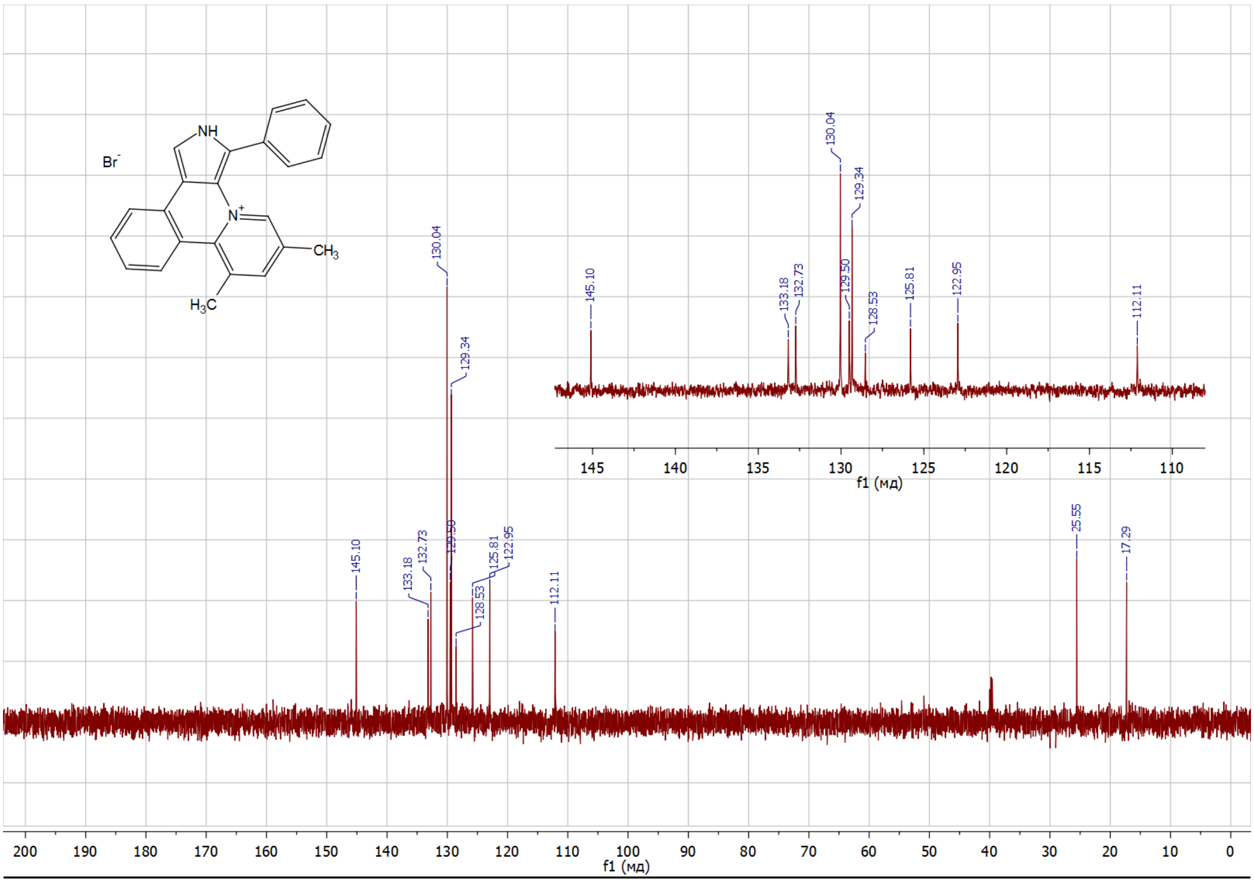

^{1}H NMR (400 MHz, DMSO-d_6) of 1-(2-(2,4-dimethoxyphenyl)-2-oxoethyl)pyridin-1-ium bromide **(5i)**

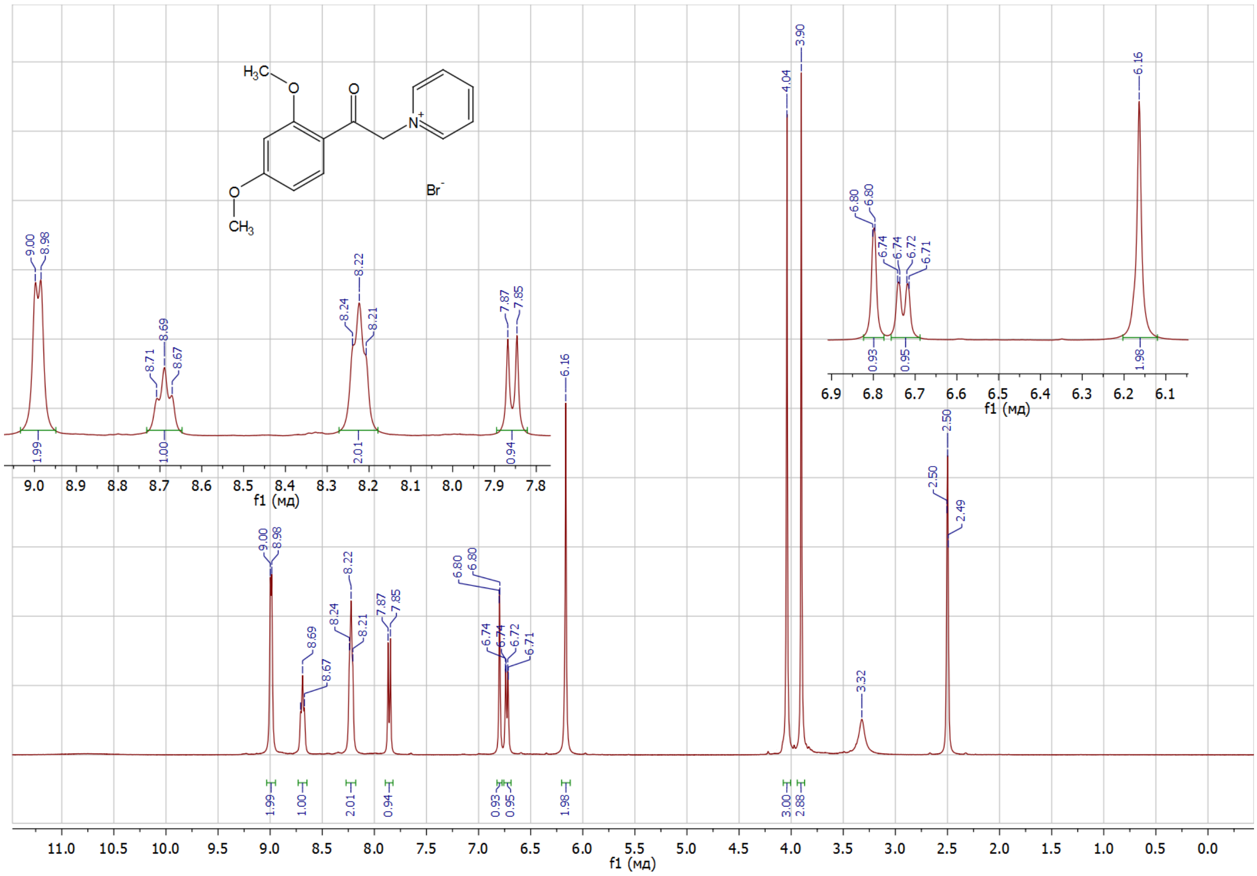

$^{13}C\{1H\}$ NMR (101 MHz, DMSO-d_6) of 1-(2-(2,4-dimethoxyphenyl)-2-oxoethyl)pyridin-1-ium bromide **(5i)**

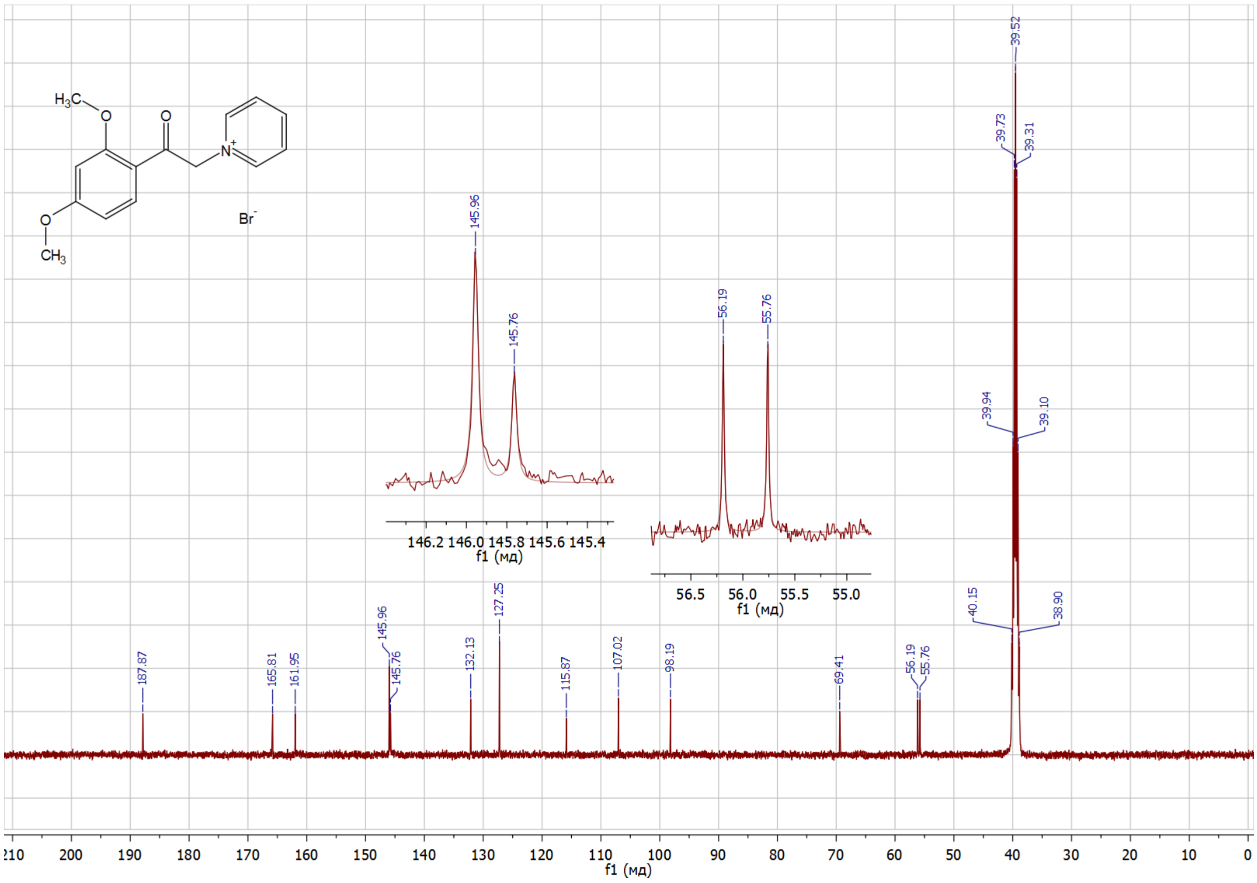

^{13}C DEPT135 NMR (101 MHz, DMSO-d_6) of 1-(2-(2,4-dimethoxyphenyl)-2-oxoethyl)pyridin-1-ium bromide **(5i)**

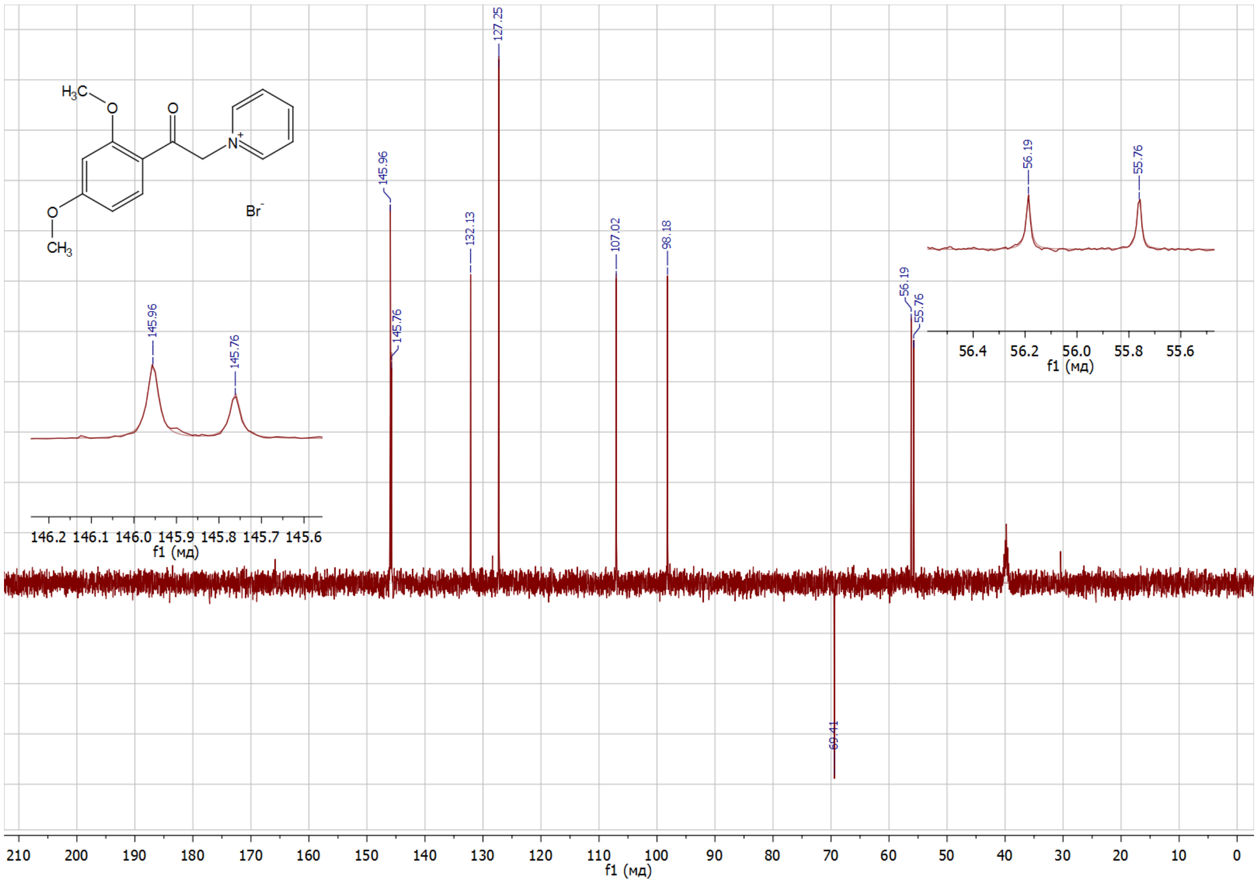

^{1}H NMR (400 MHz, DMSO-d$_6$) of 4-(4-methoxyphenyl)-1-(2-oxo-2-phenylethyl)pyridin-1-ium bromide **(5r)**.

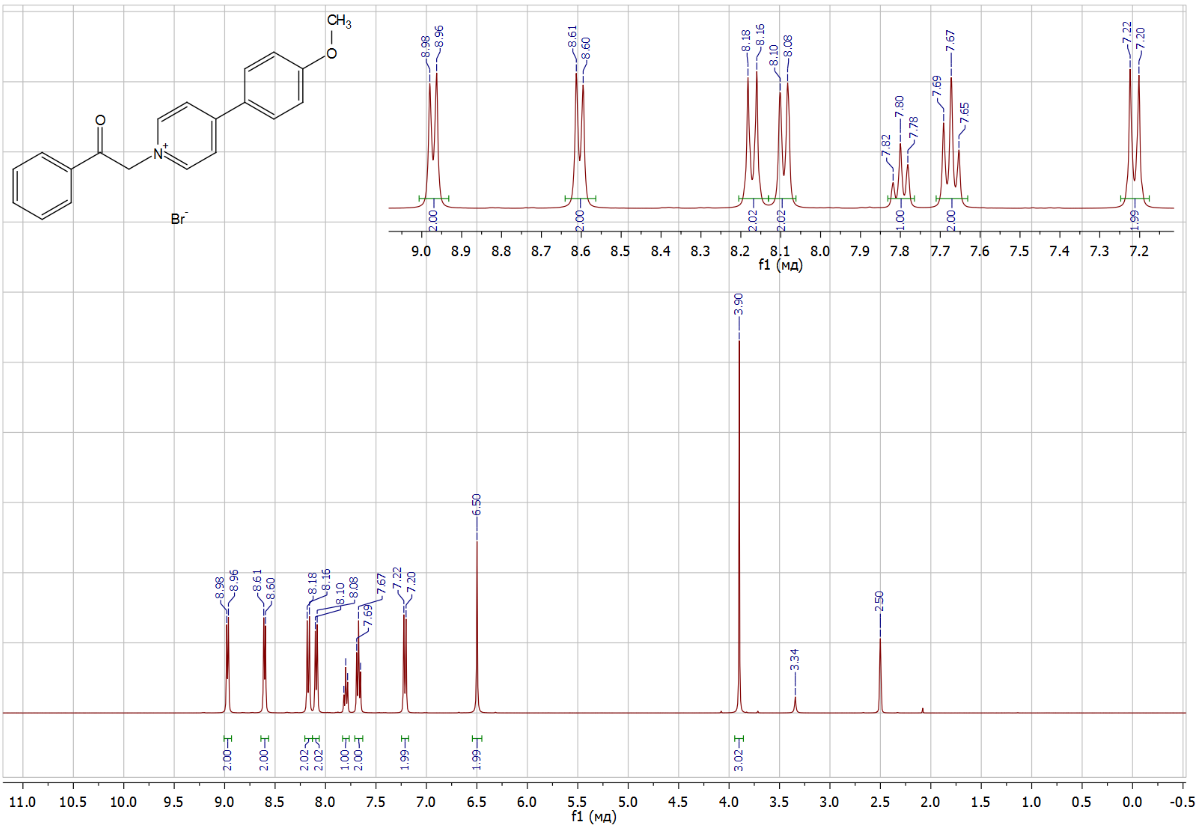

$^{13}C\{^{1}H\}$ NMR (101 MHz, DMSO-d$_6$) of 4-(4-methoxyphenyl)-1-(2-oxo-2-phenylethyl)pyridin-1-ium bromide **(5r)**.

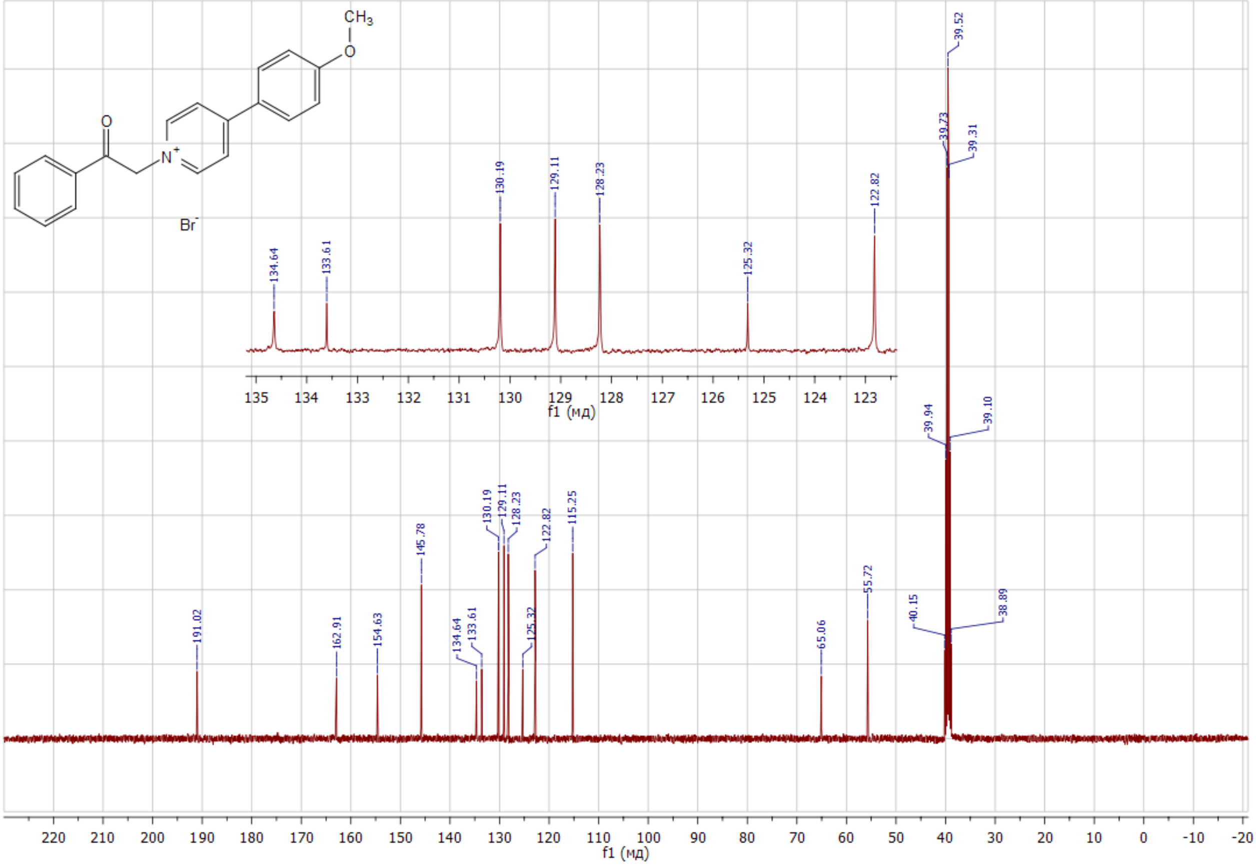

^{13}C DEPT135 NMR (101 MHz, DMSO-d_6) of 4-(4-methoxyphenyl)-1-(2-oxo-2-phenylethyl)pyridin-1-ium bromide **(5r)**.

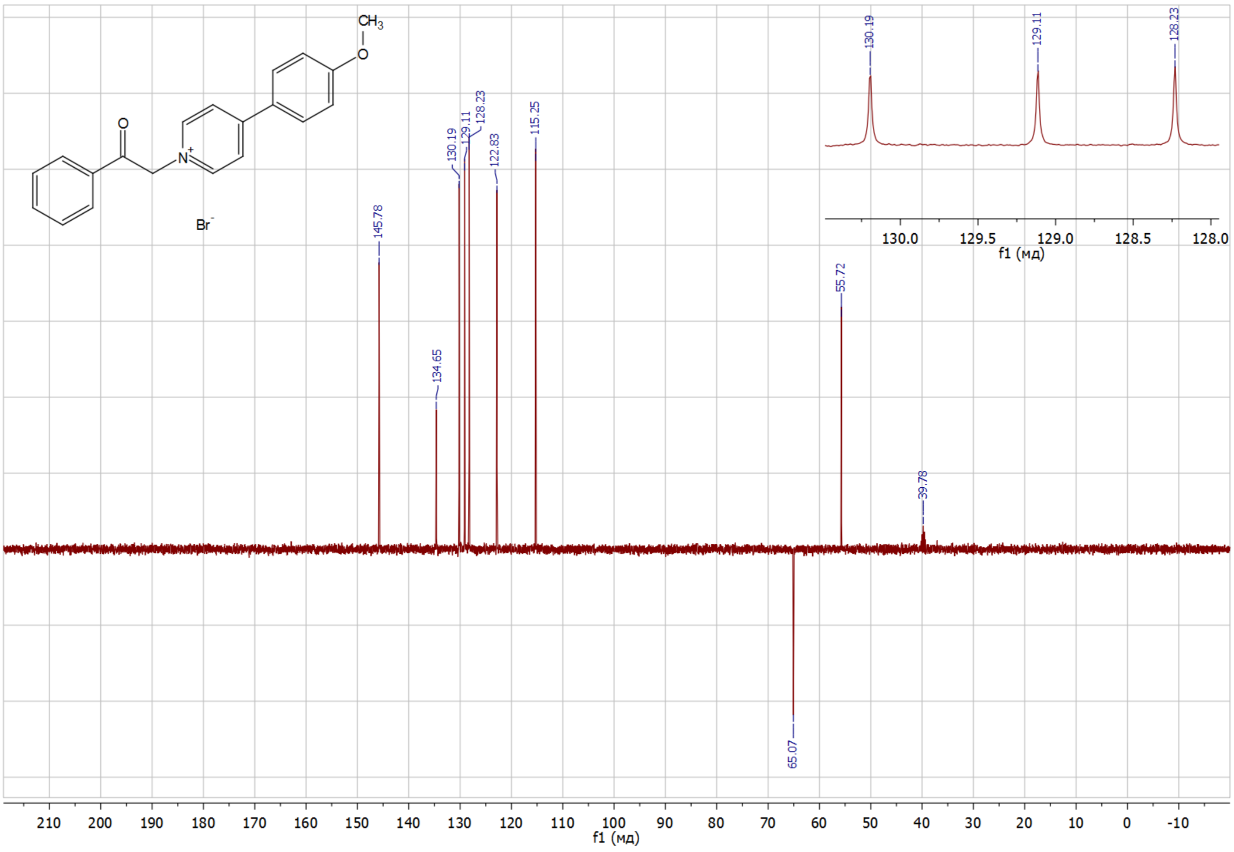

^{1}H NMR (400 MHz, DMSO-d_6) of 4-(methoxycarbonyl)-1-(2-oxo-2-phenylethyl)pyridin-1-ium bromide **(5u)**

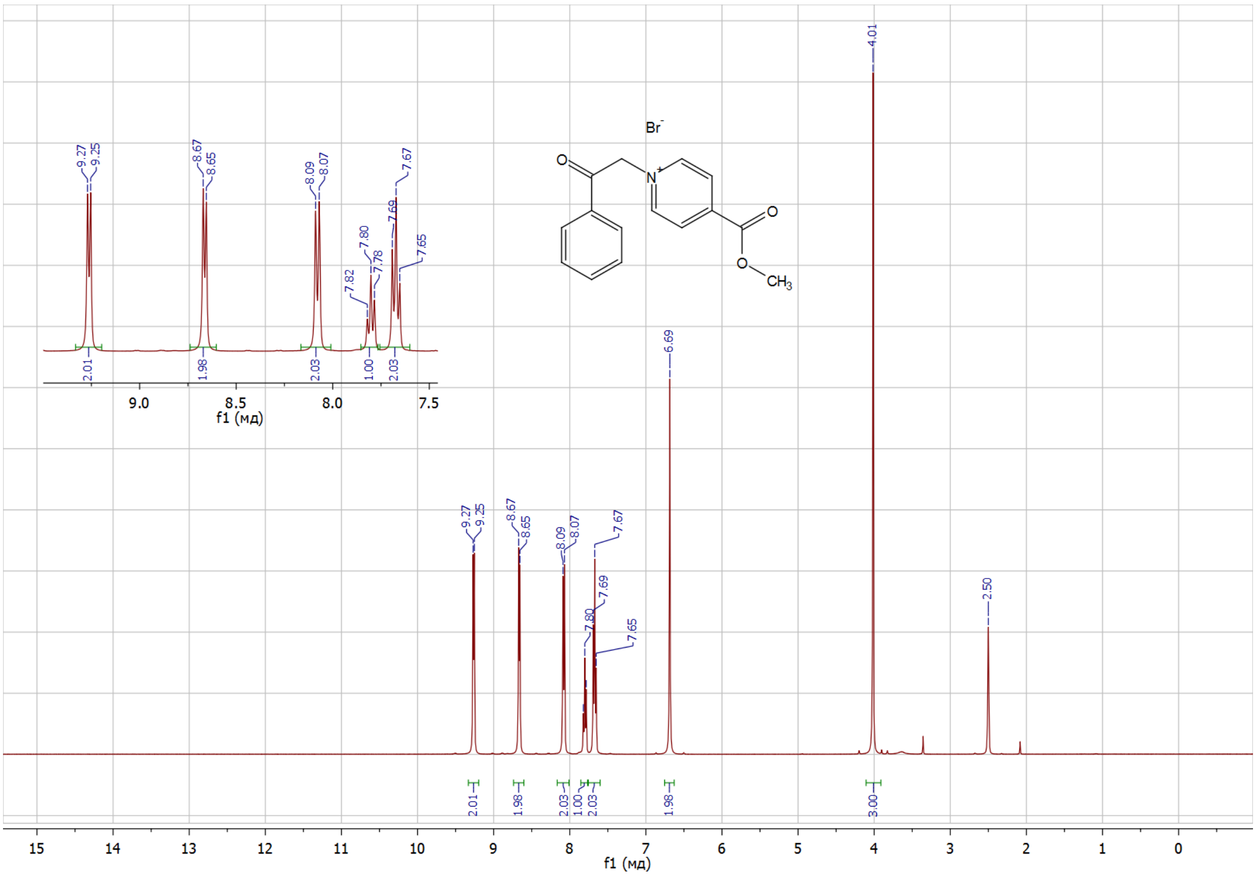

$^{13}C\{1H\}$ NMR (101 MHz, DMSO-d_6) of 4-(methoxycarbonyl)-1-(2-oxo-2-phenylethyl)pyridin-1-ium bromide **(5u)**

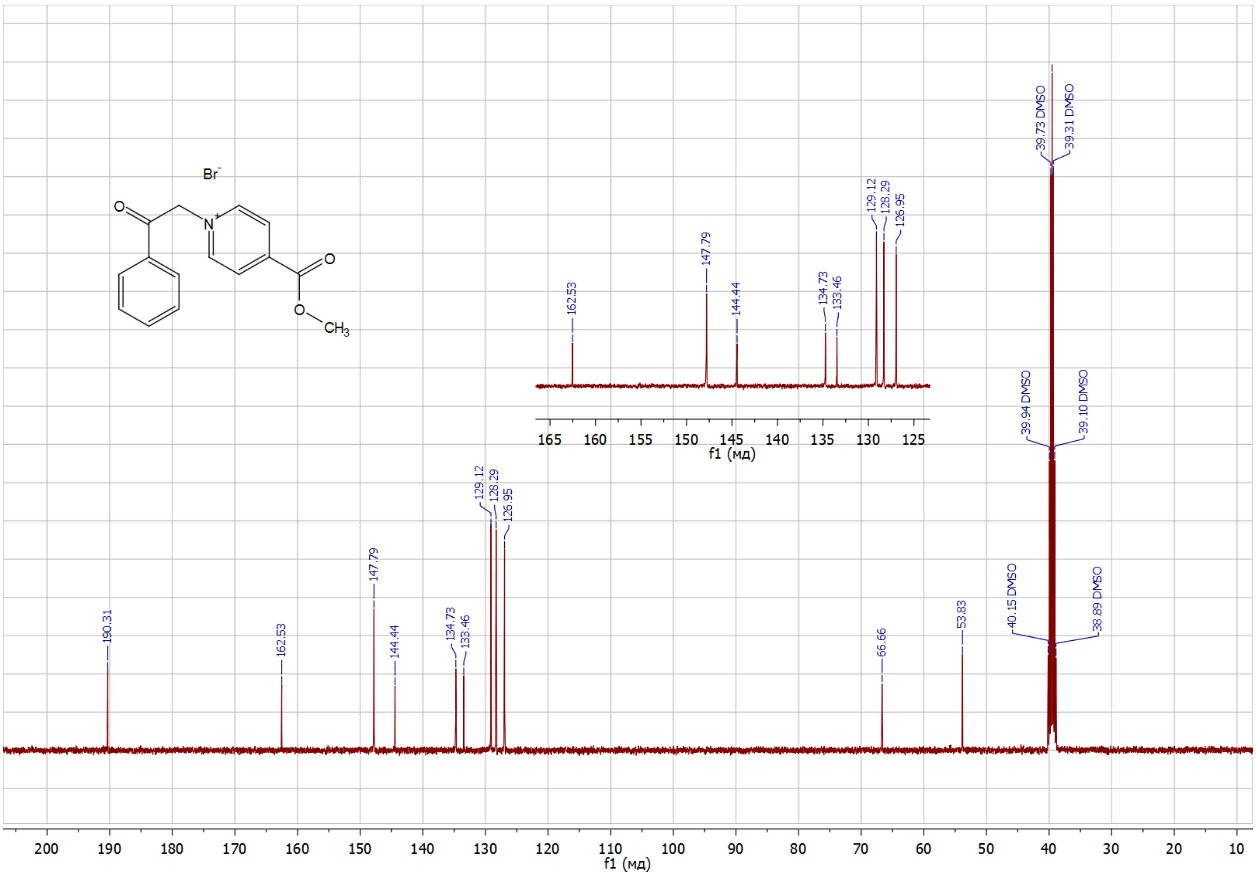

^{13}C DEPT135 NMR (101 MHz, DMSO-d_6) of 4-(methoxycarbonyl)-1-(2-oxo-2-phenylethyl)pyridin-1-ium bromide **(5u)**

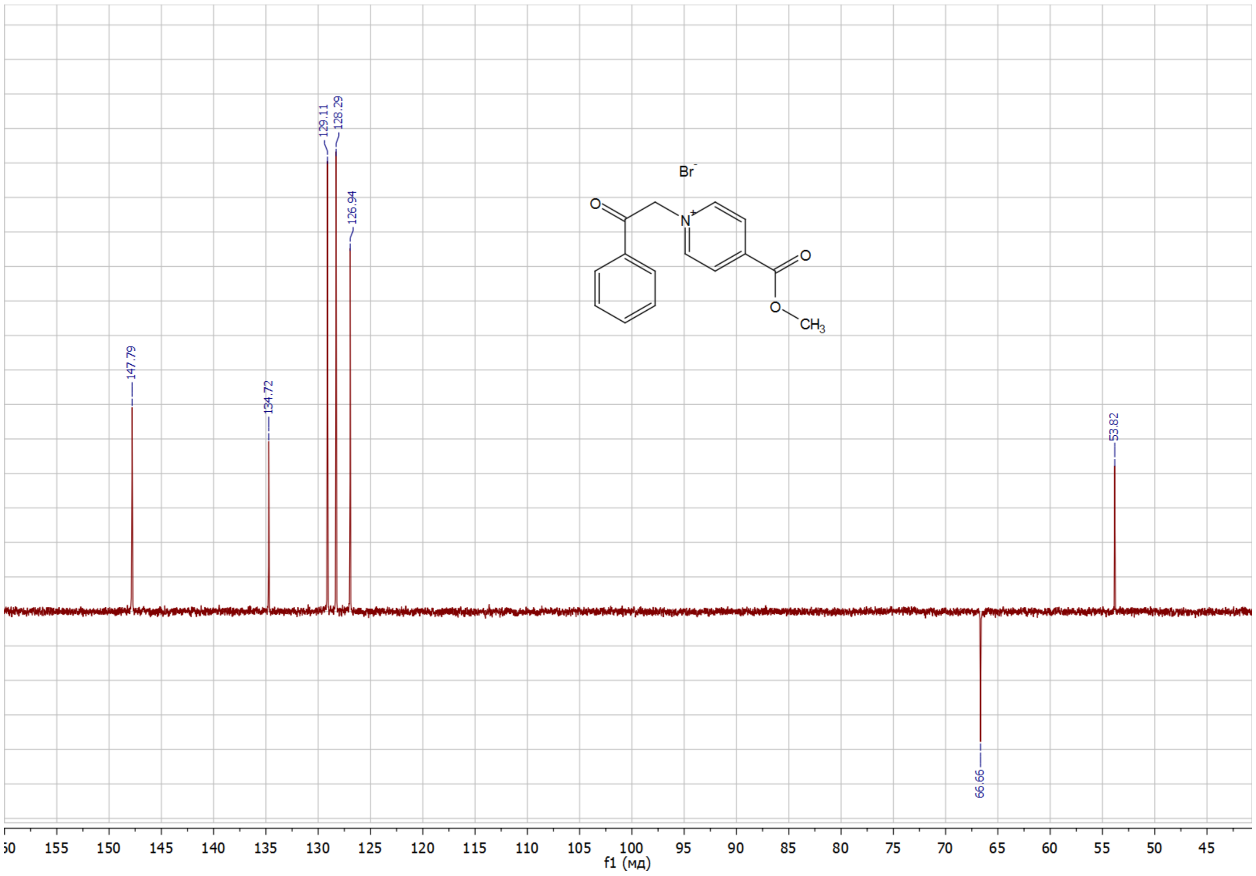

147.79
134.72
129.11
128.29
126.94
53.82
66.66
Br
N
O
O
O
CH3
50
155
150
145
140
135
130
125
120
115
110
105
100
95
90
85
80
75
70
65
60
55
50
45
f1 (мд)

^{1}H NMR (400 MHz, DMSO-d_6) of 4-benzoyl-1-(2-oxo-2-phenylethyl)pyridin-1-ium bromide **(5w)**

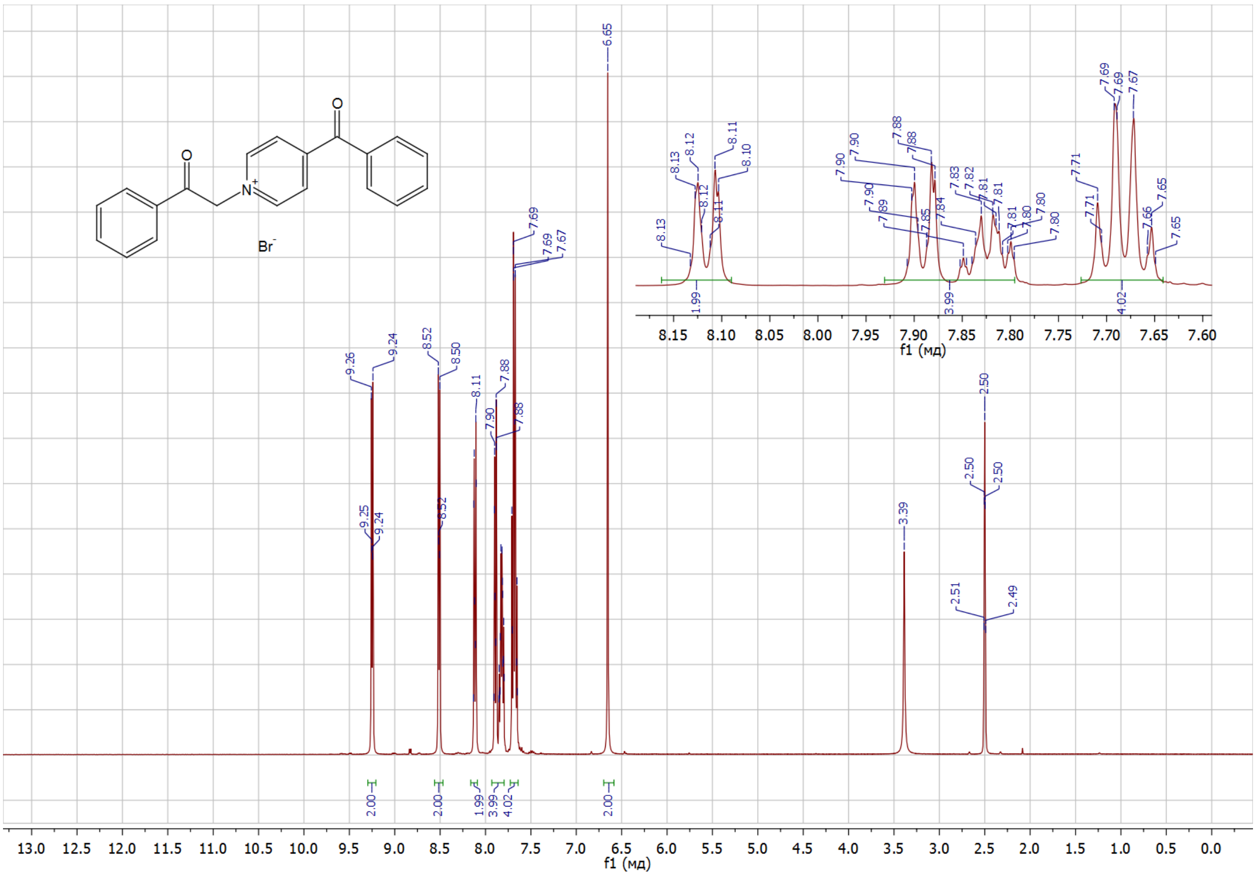

^{13}C{1H} NMR (101 MHz, DMSO-d_6) of 4-benzoyl-1-(2-oxo-2-phenylethyl)pyridin-1-ium bromide **(5w)**

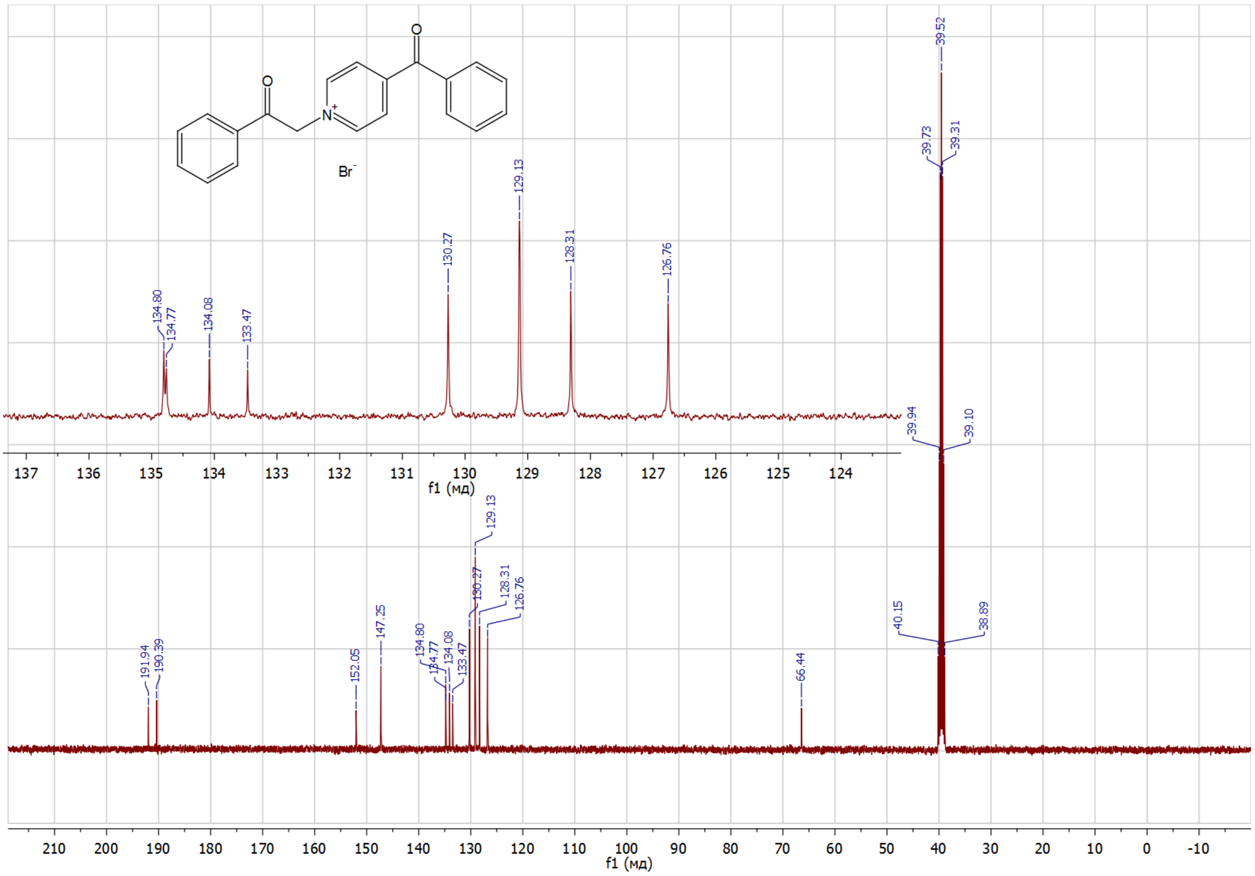

^{13}C DEPT135 NMR (101 MHz, DMSO-d_6) of 4-benzoyl-1-(2-oxo-2-phenylethyl)pyridin-1-ium bromide **(5w)**

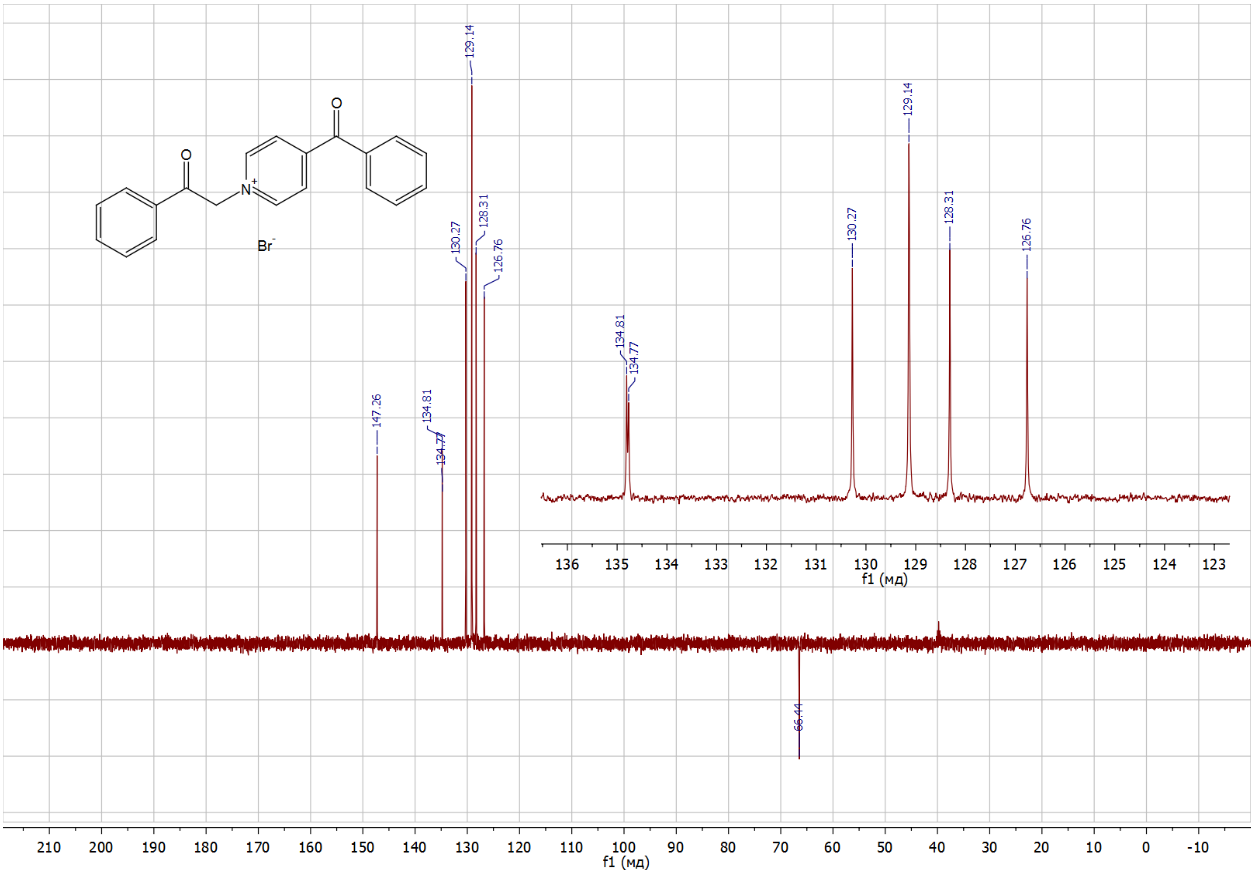

^{1}H NMR (400 MHz, DMSO-d_6) of 3-phenylpyrido[2,1-*a*]pyrrolo[3,4-*c*]isoquinoline (**6a**)

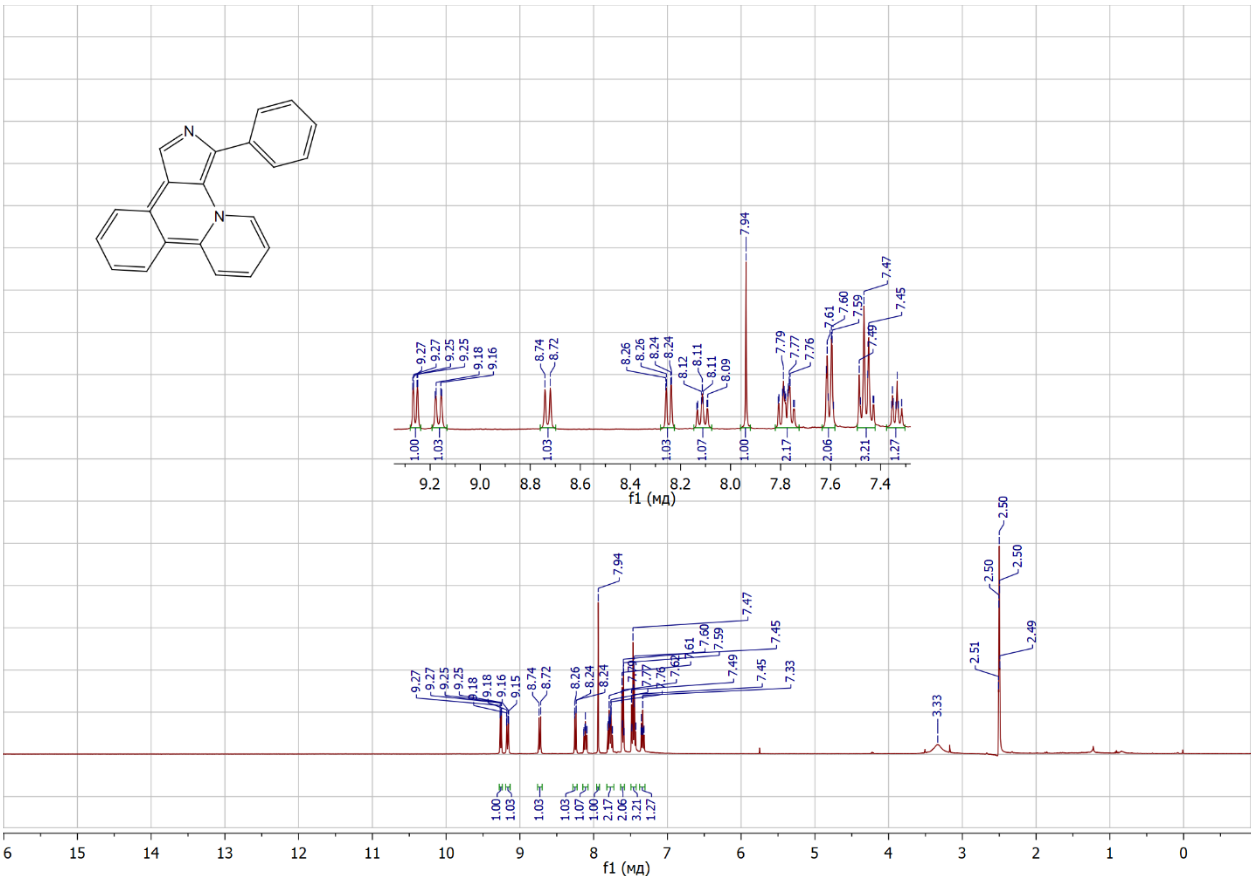

^{13}C{1H} NMR (101 MHz, DMSO-d_6) of 3-phenylpyrido[2,1-*a*]pyrrolo[3,4-*c*]isoquinoline (**6a**)

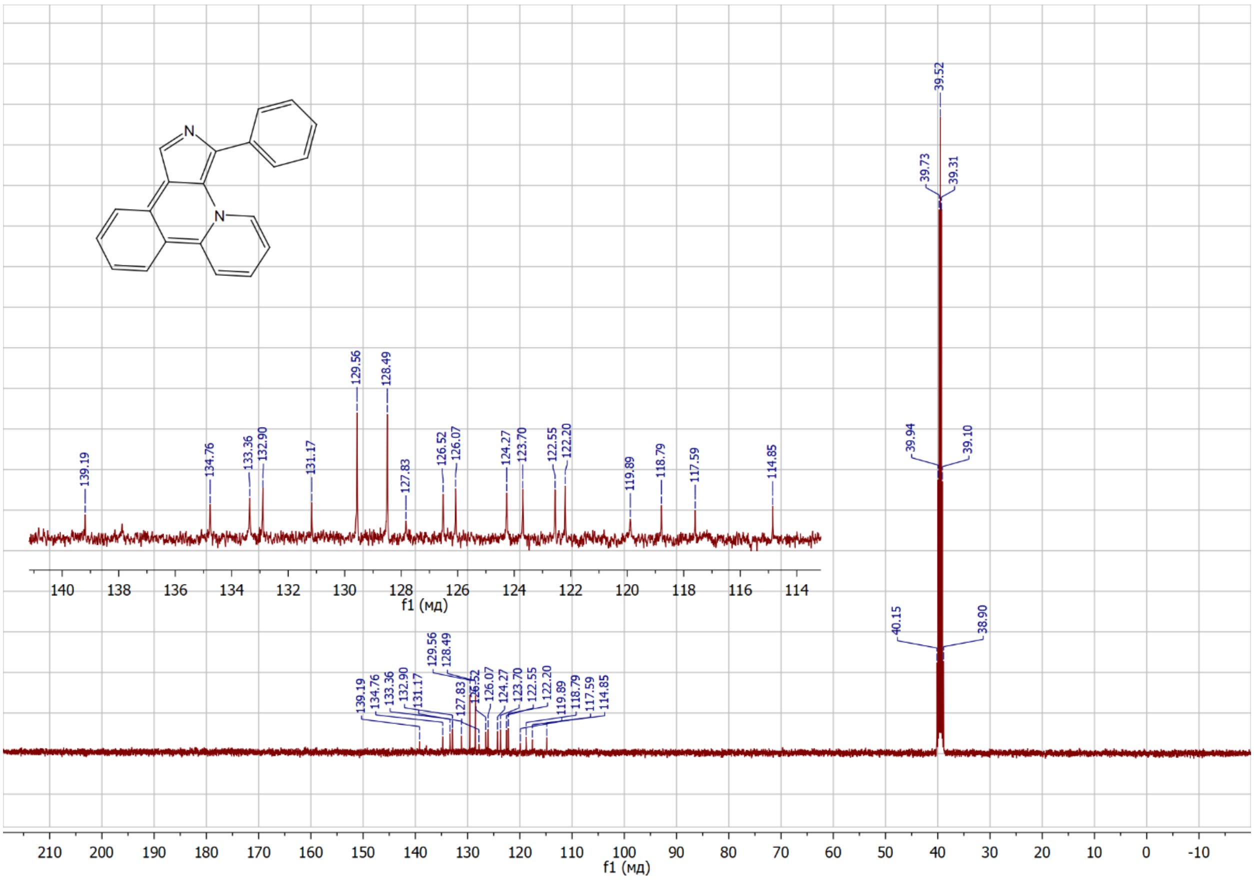

^{13}C DEPT135 NMR (101 MHz, DMSO-d_6) of 3-phenylpyrido[2,1-*a*]pyrrolo[3,4-*c*]isoquinoline (**6a**)

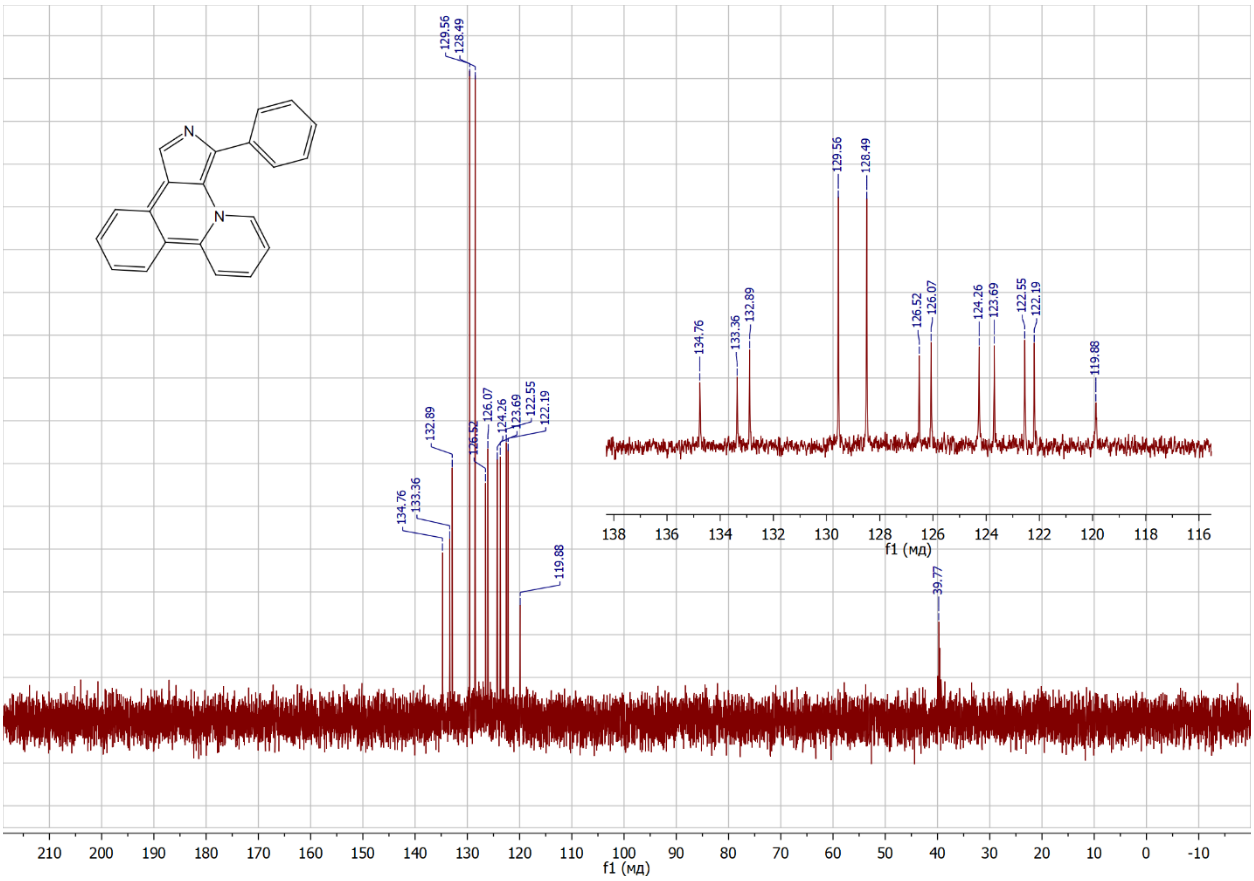

^{1}H NMR (400 MHz, DMSO-d_6) of 3-(4-chlorophenyl)pyrido[2,1-*a*]pyrrolo[3,4-*c*]isoquinoline (**6c**)

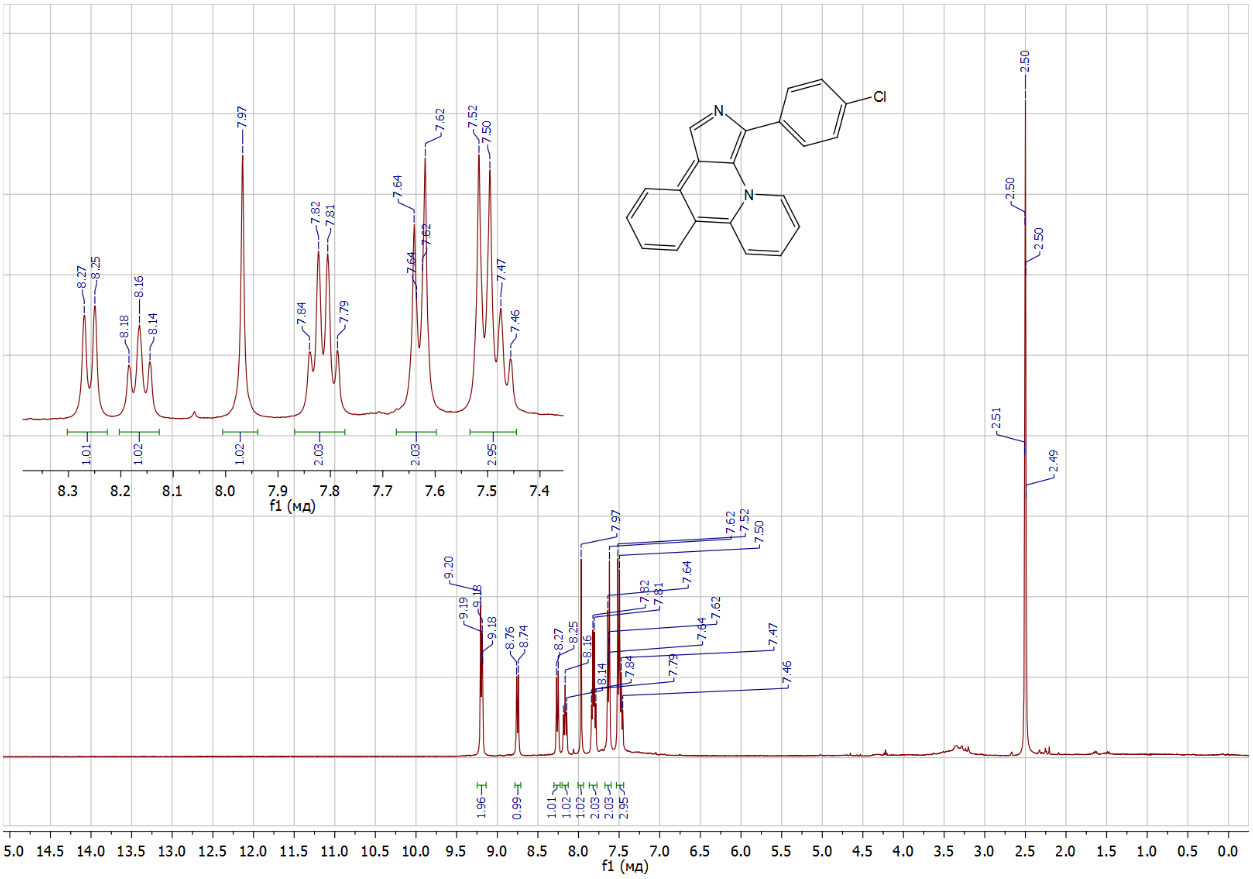

^{13}C{1H} NMR (101 MHz, DMSO-d_6) of 3-(4-chlorophenyl)pyrido[2,1-*a*]pyrrolo[3,4-*c*]isoquinoline (**6c**)

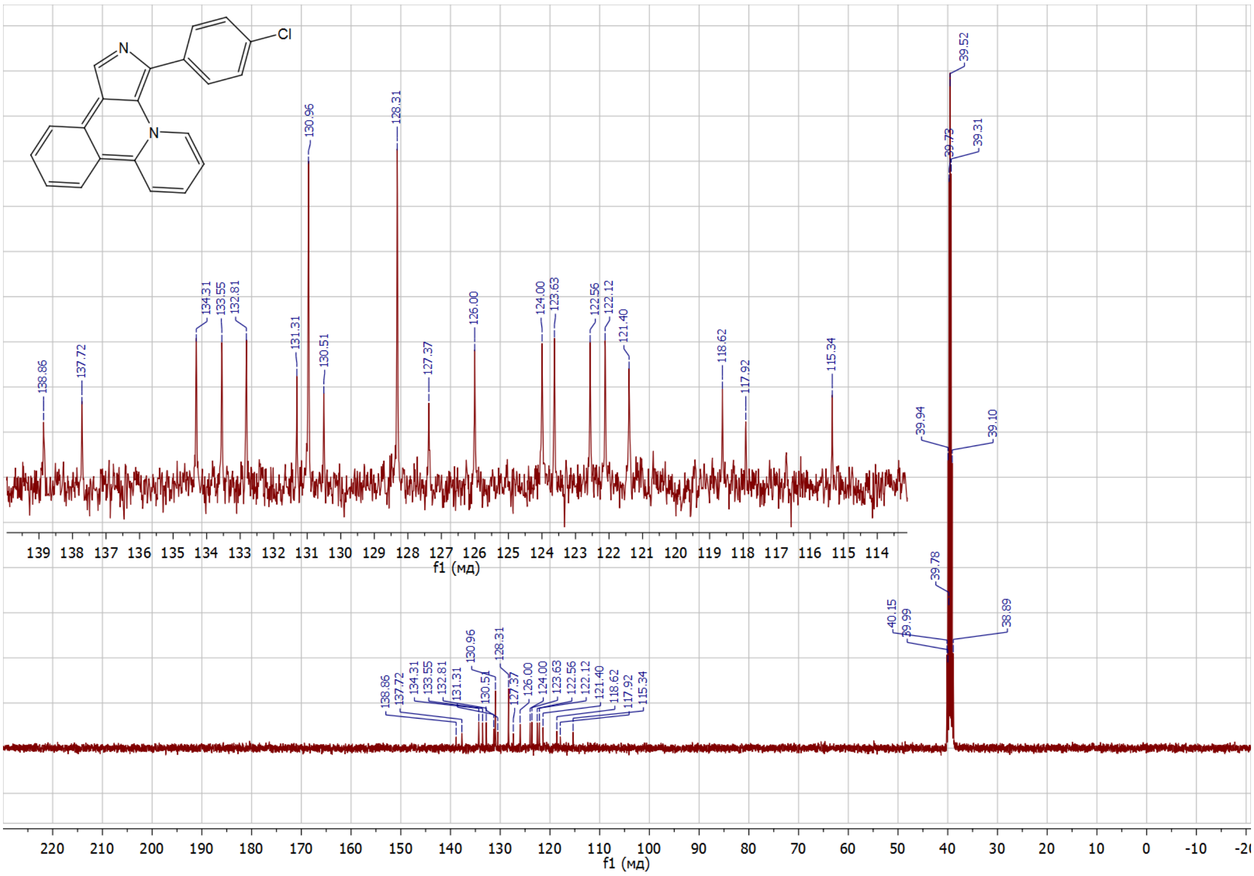

^{13}C DEPT135 NMR (101 MHz, DMSO-d_6) of 3-(4-chlorophenyl)pyrido[2,1-*a*]pyrrolo[3,4-*c*]isoquinoline (**6c**)

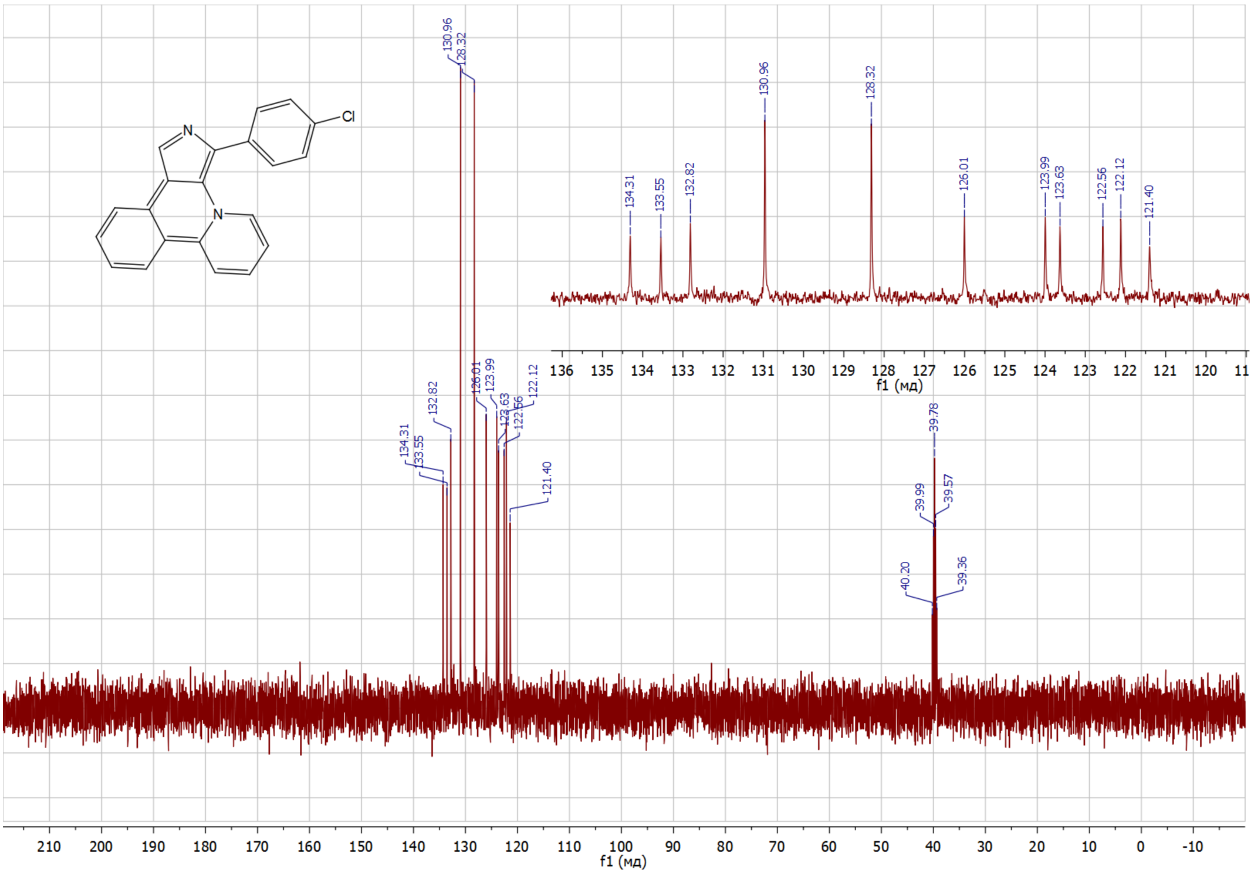

^{1}H NMR (400 MHz, DMSO-d_6) of 3-(3-methyl)pyrido[2,1-*a*]pyrrolo[3,4-*c*]isoquinoline (**6e**)

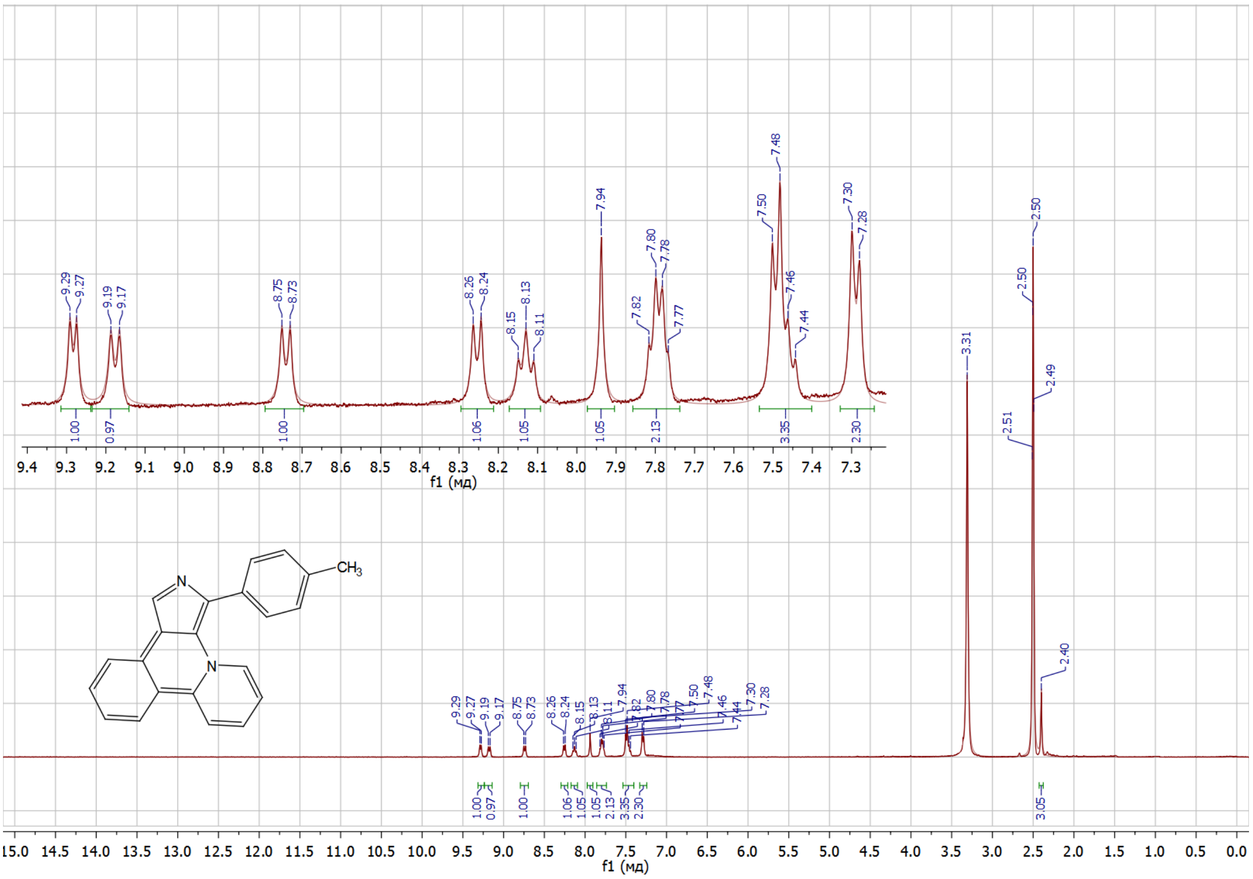

^{13}C{1H} NMR (101 MHz, DMSO-d_6) of 3-(3-methyl)pyrido[2,1-*a*]pyrrolo[3,4-*c*]isoquinoline (**6e**)

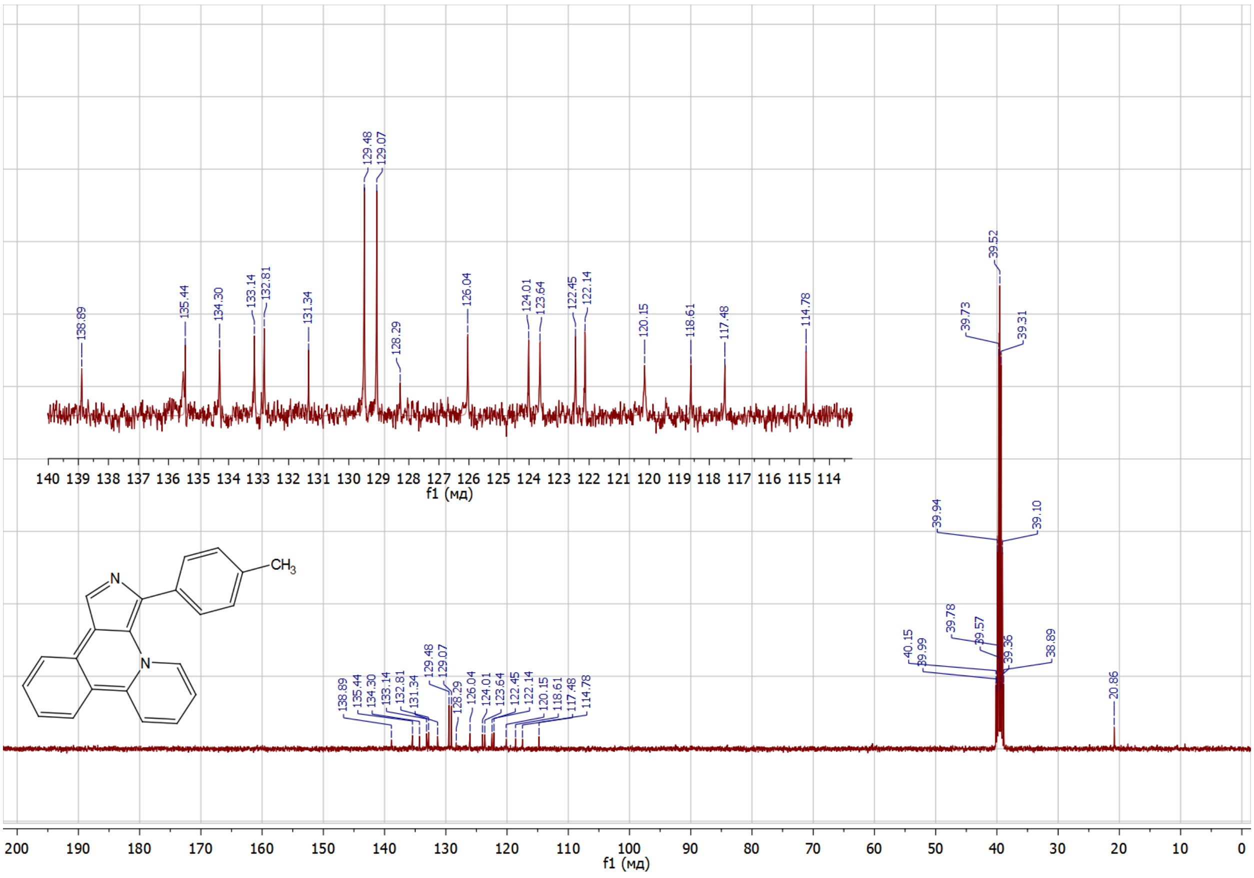

^{13}C DEPT135 NMR (101 MHz, DMSO-d_6) of 3-(3-methyl)pyrido[2,1-*a*]pyrrolo[3,4-*c*]isoquinoline (**6e**)

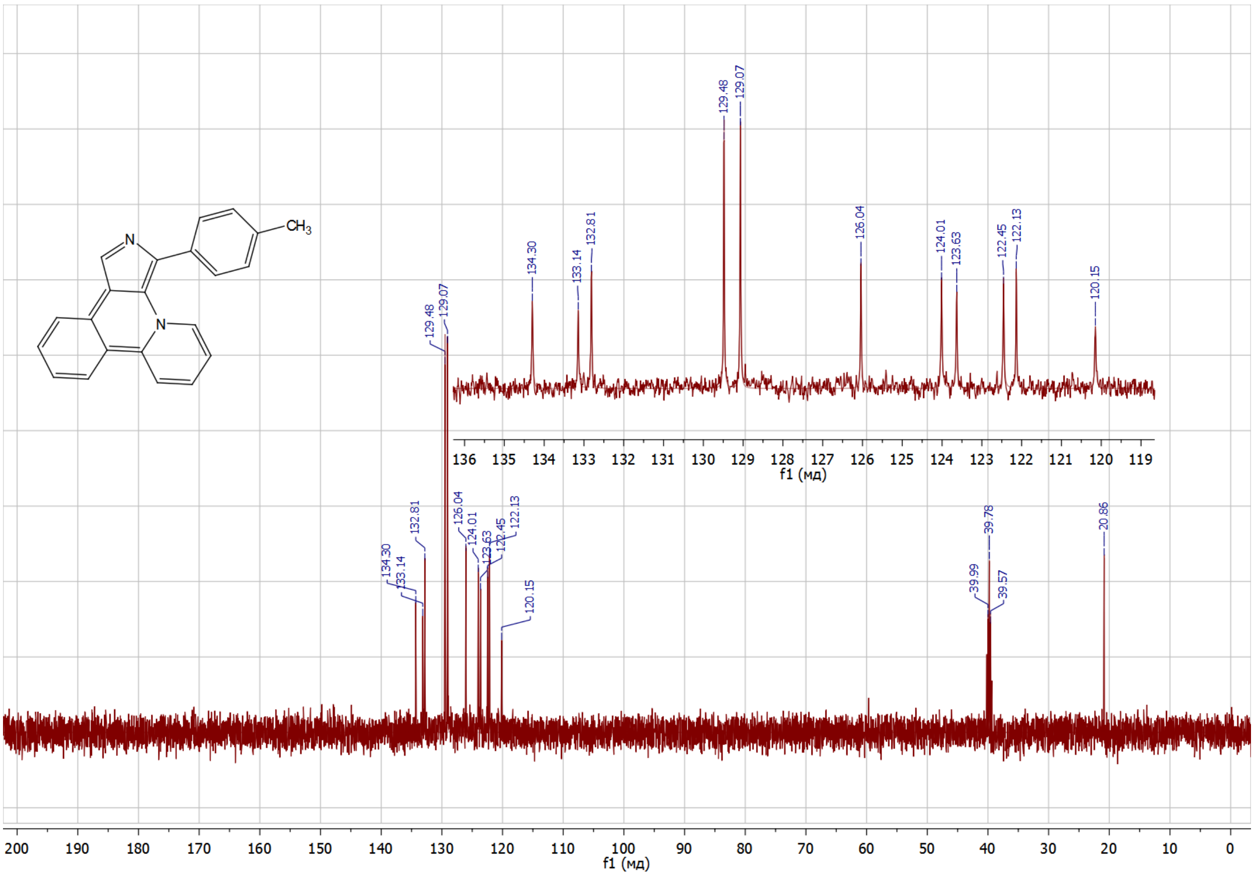

^{1}H NMR (400 MHz, DMSO-d_6) of 3-(4-methoxyphenyl)pyrido[2,1-*a*]pyrrolo[3,4-*c*]isoquinoline (**6f**)

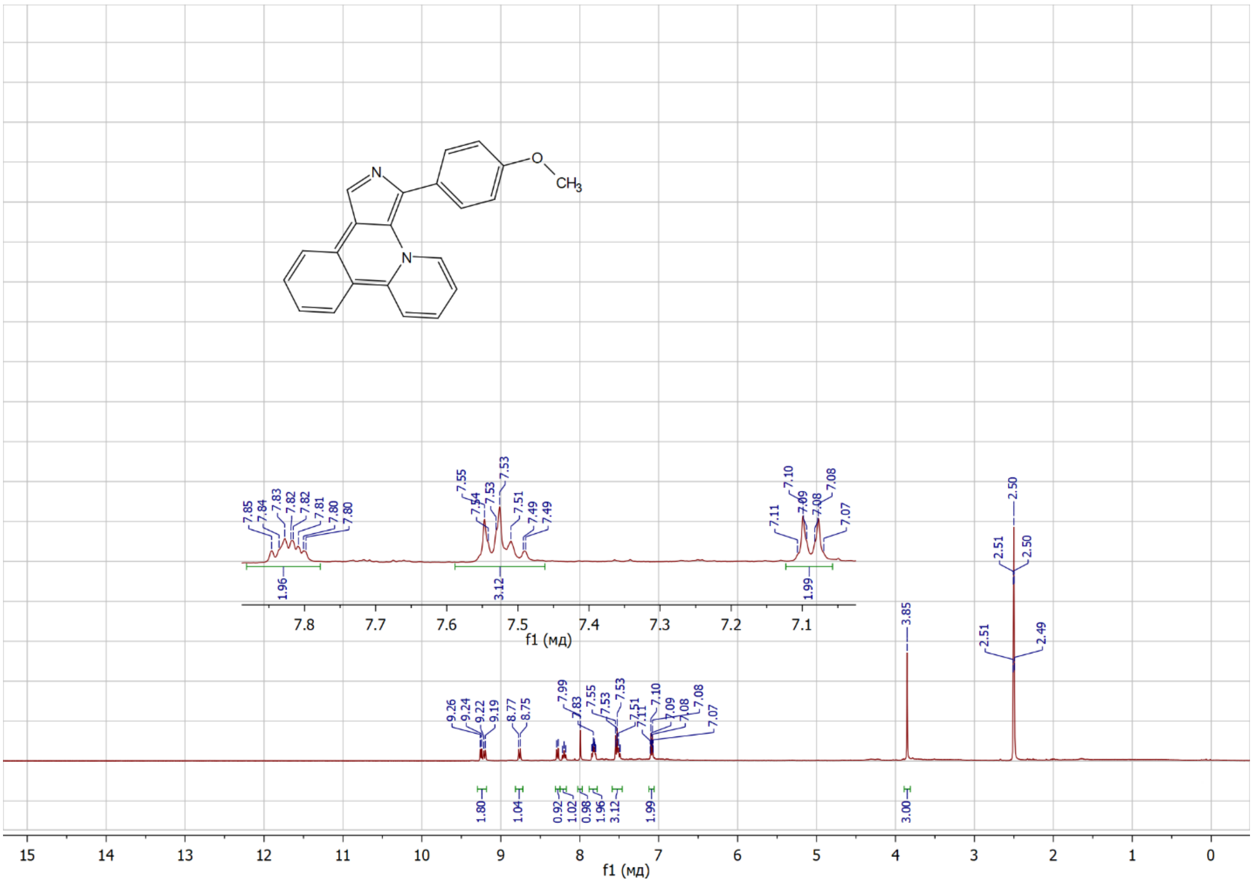

$^{13}C\{1H\}$ NMR (101 MHz, DMSO-d_6) of 3-(4-methoxyphenyl)pyrido[2,1-*a*]pyrrolo[3,4-*c*]isoquinoline (**6f**)

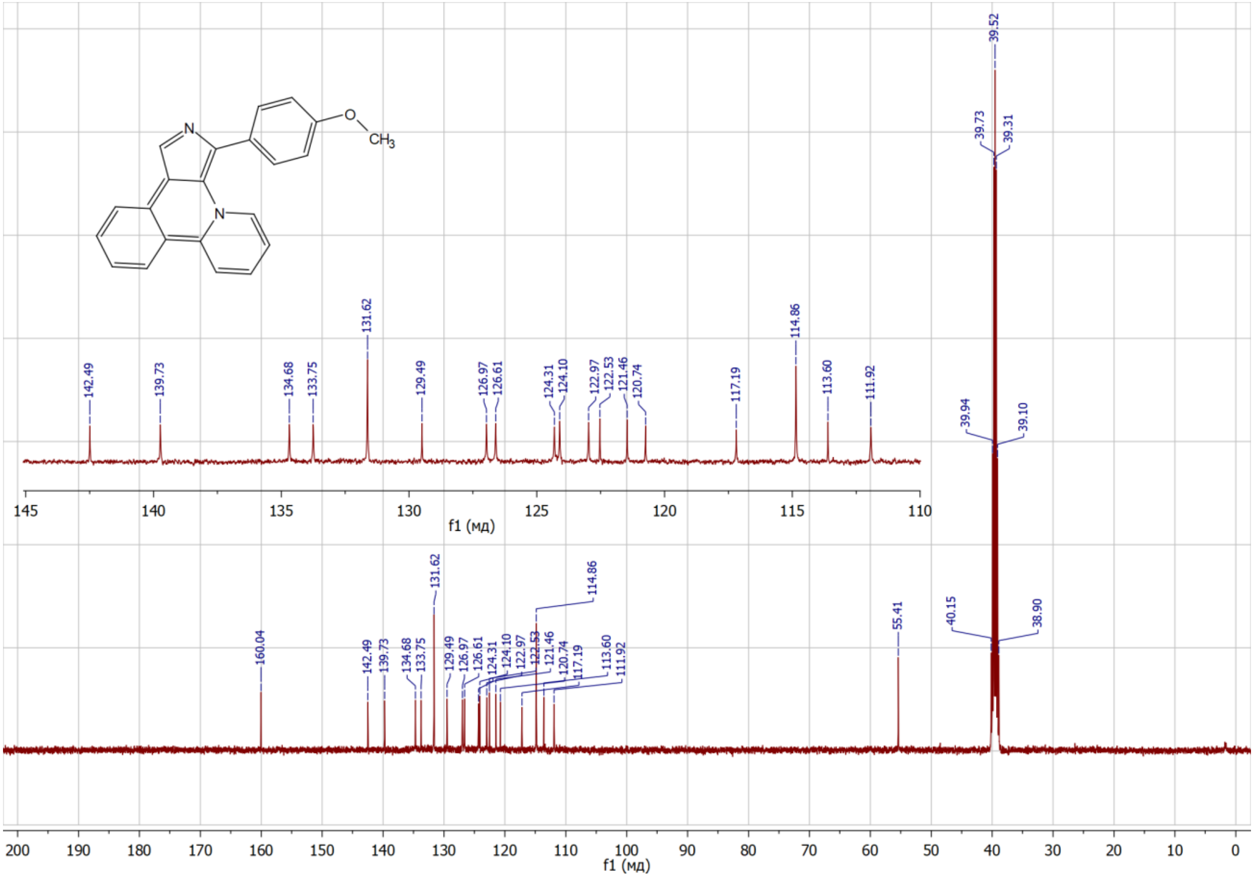

^{13}C DEPT135 NMR (101 MHz, DMSO-d_6) of 3-(4-methoxyphenyl)pyrido[2,1-*a*]pyrrolo[3,4-*c*]isoquinoline (**6f**)

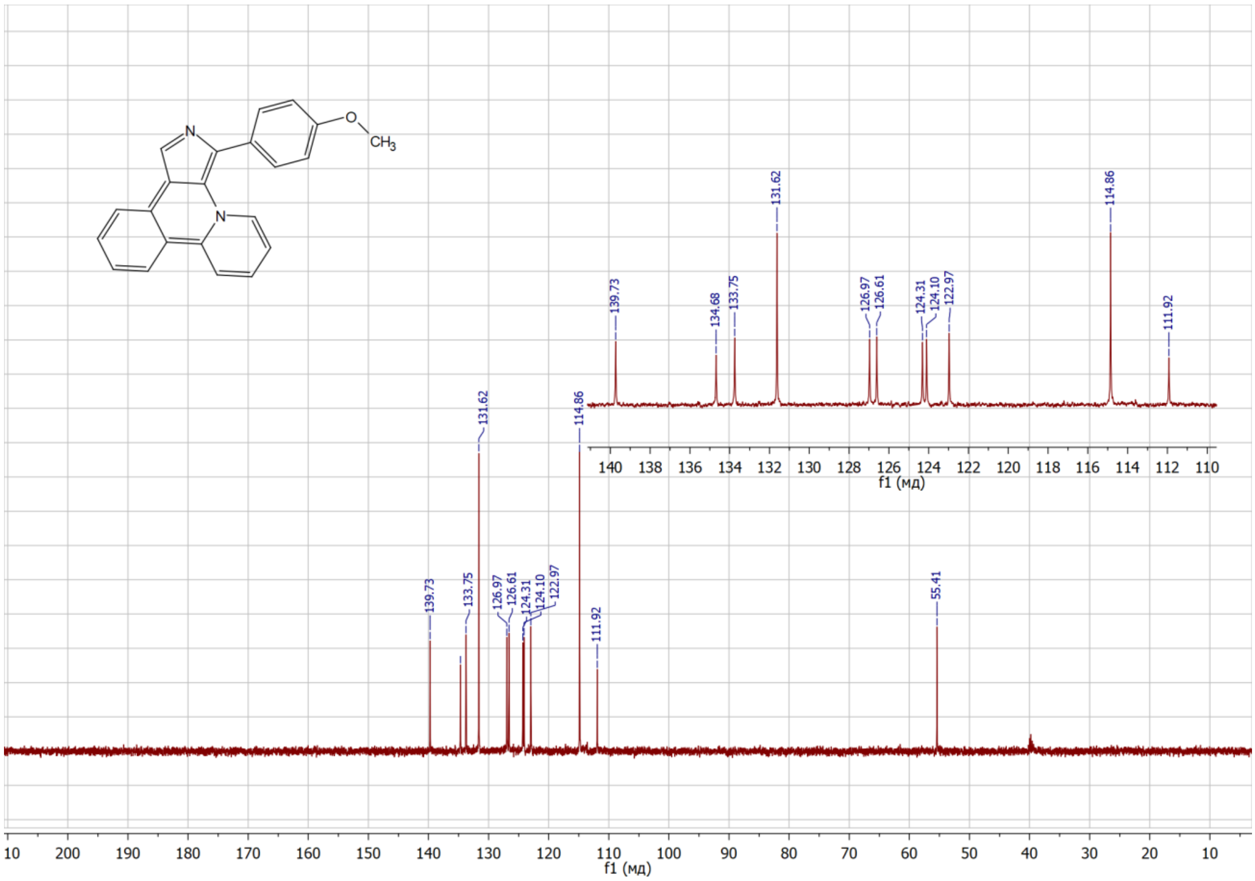

^{1}H NMR (400 MHz, DMSO-d_6) of 3-(2-methoxyphenyl)pyrido[2,1-*a*]pyrrolo[3,4-*c*]isoquinoline (**6g**)

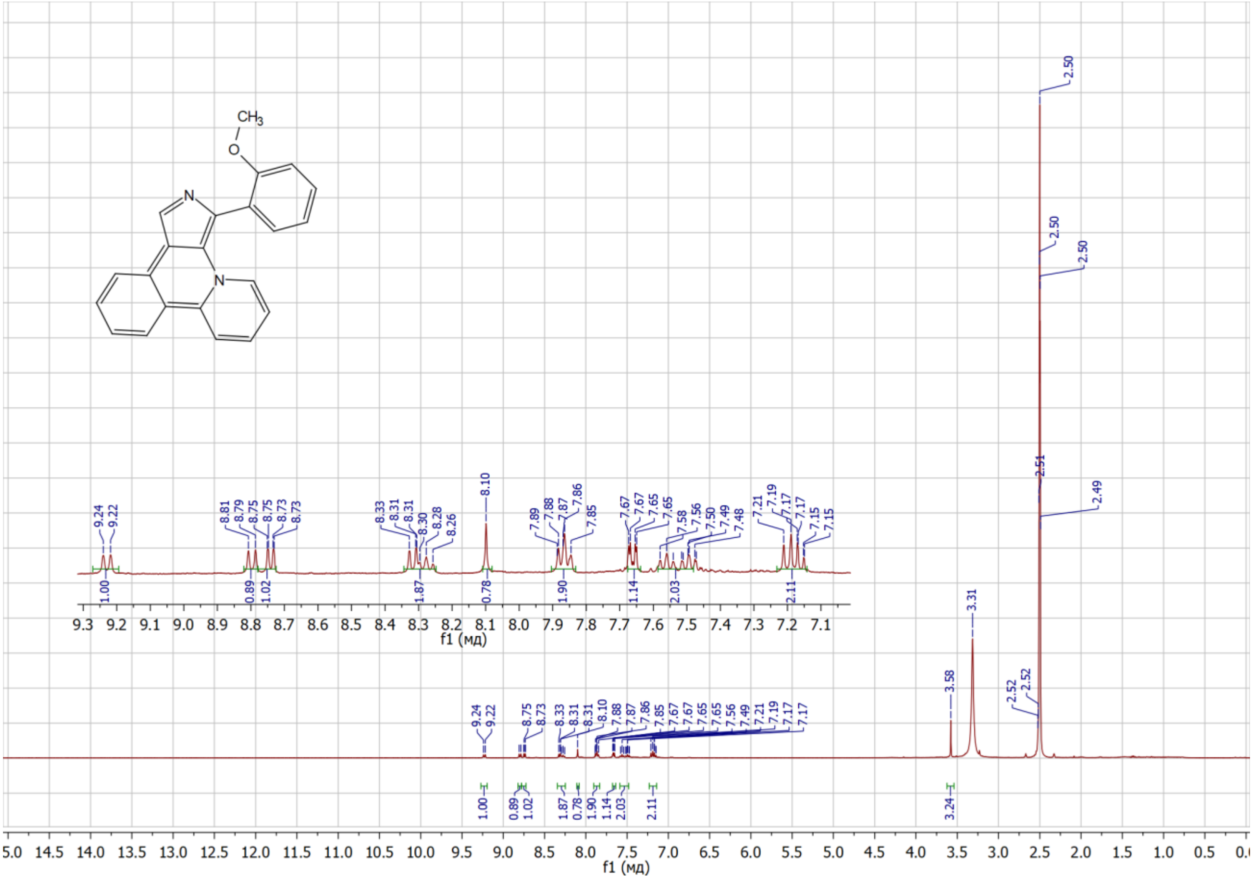

$^{13}C\{^{1}H\}$ NMR (101 MHz, DMSO-d_6) of 3-(2-methoxyphenyl)pyrido[2,1-*a*]pyrrolo[3,4-*c*]isoquinoline (**6g**)

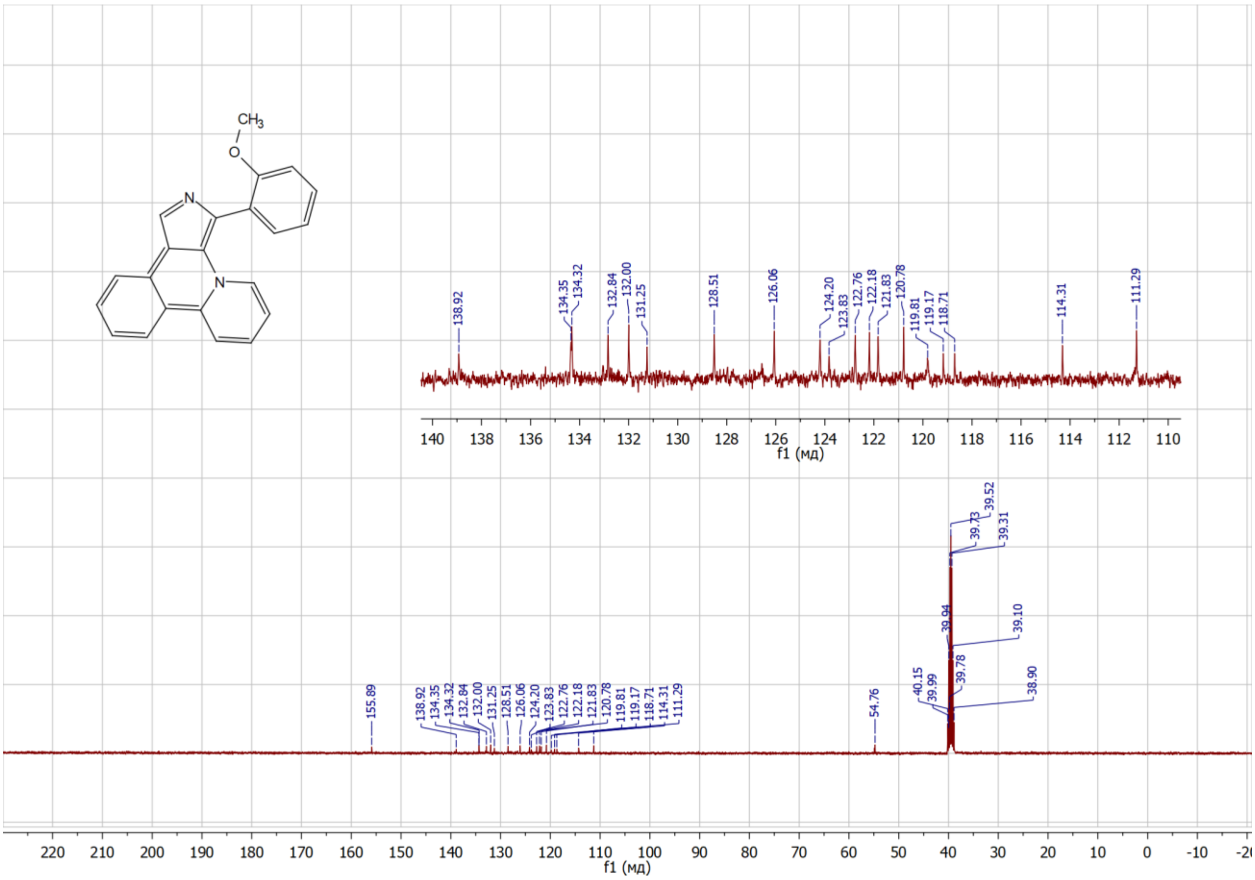

^{13}C DEPT135 NMR (101 MHz, DMSO-d_6) of 3-(2-methoxyphenyl)pyrido[2,1-*a*]pyrrolo[3,4-*c*]isoquinoline (**6g**)

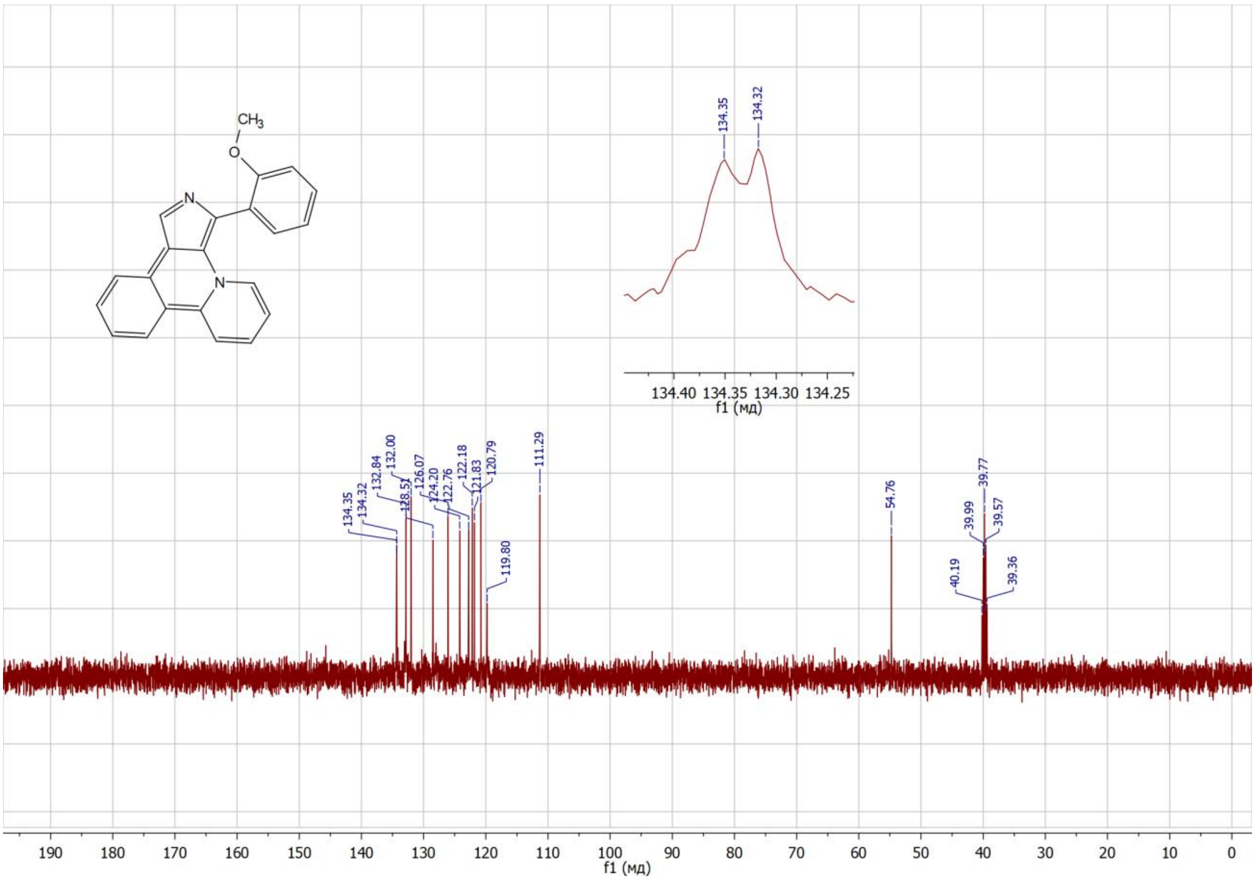

^{1}H NMR (400 MHz, DMSO-d_6) of 3-(3-methoxyphenyl)pyrido[2,1-*a*]pyrrolo[3,4-*c*]isoquinoline (**6h**)

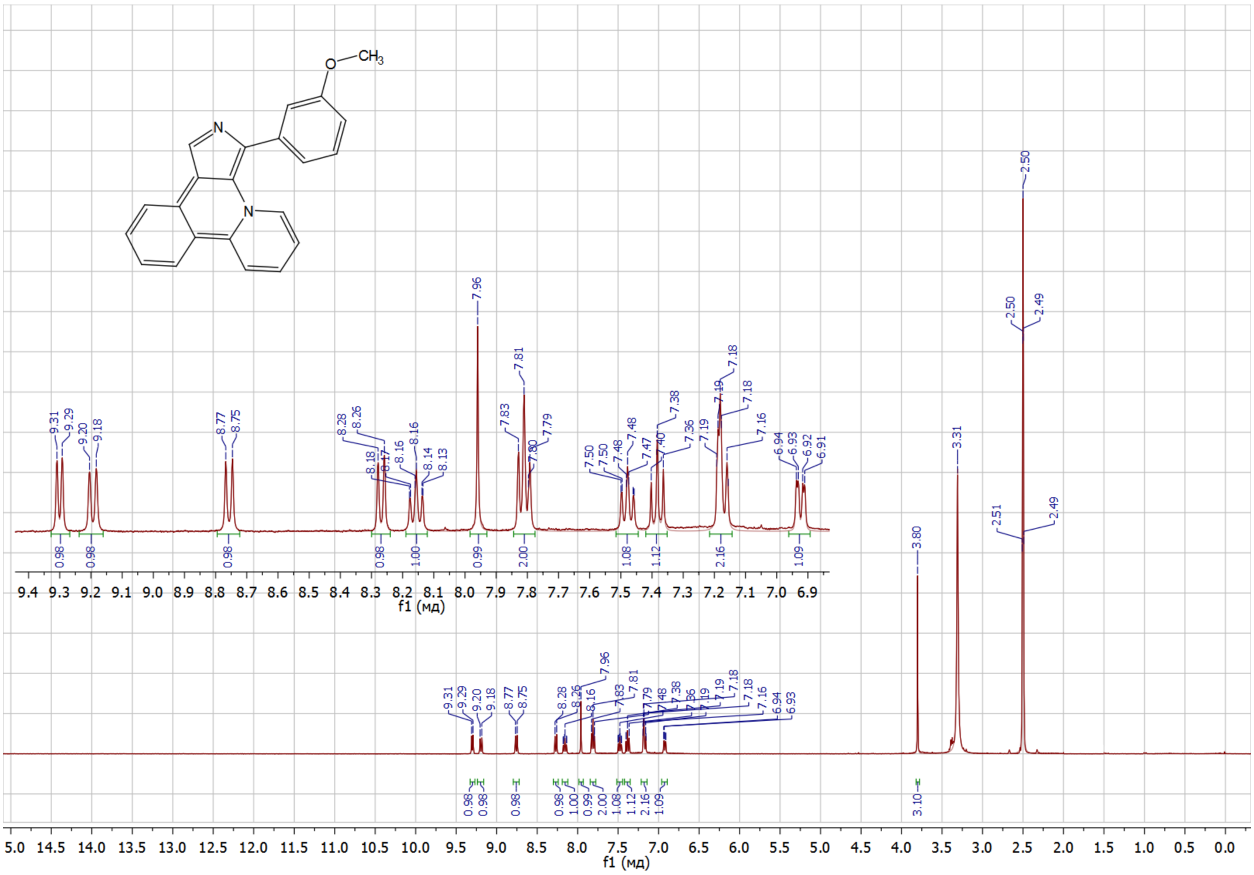

^{13}C{1H} NMR (101 MHz, DMSO-d_6) of 3-(3-methoxyphenyl)pyrido[2,1-*a*]pyrrolo[3,4-*c*]isoquinoline (**6h**)

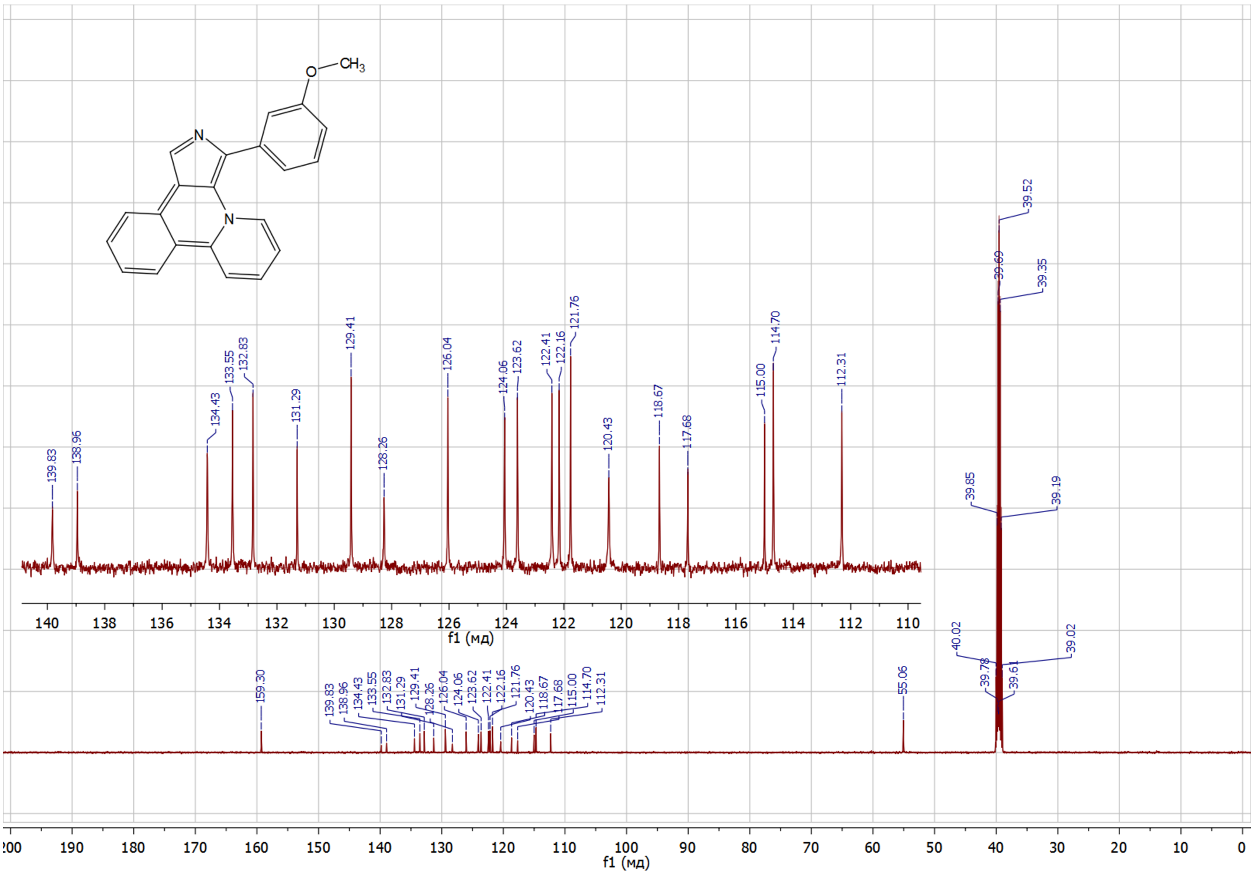

^{13}C DEPT135 NMR (101 MHz, DMSO-d_6) of 3-(3-methoxyphenyl)pyrido[2,1-*a*]pyrrolo[3,4-*c*]isoquinoline (**6h**)

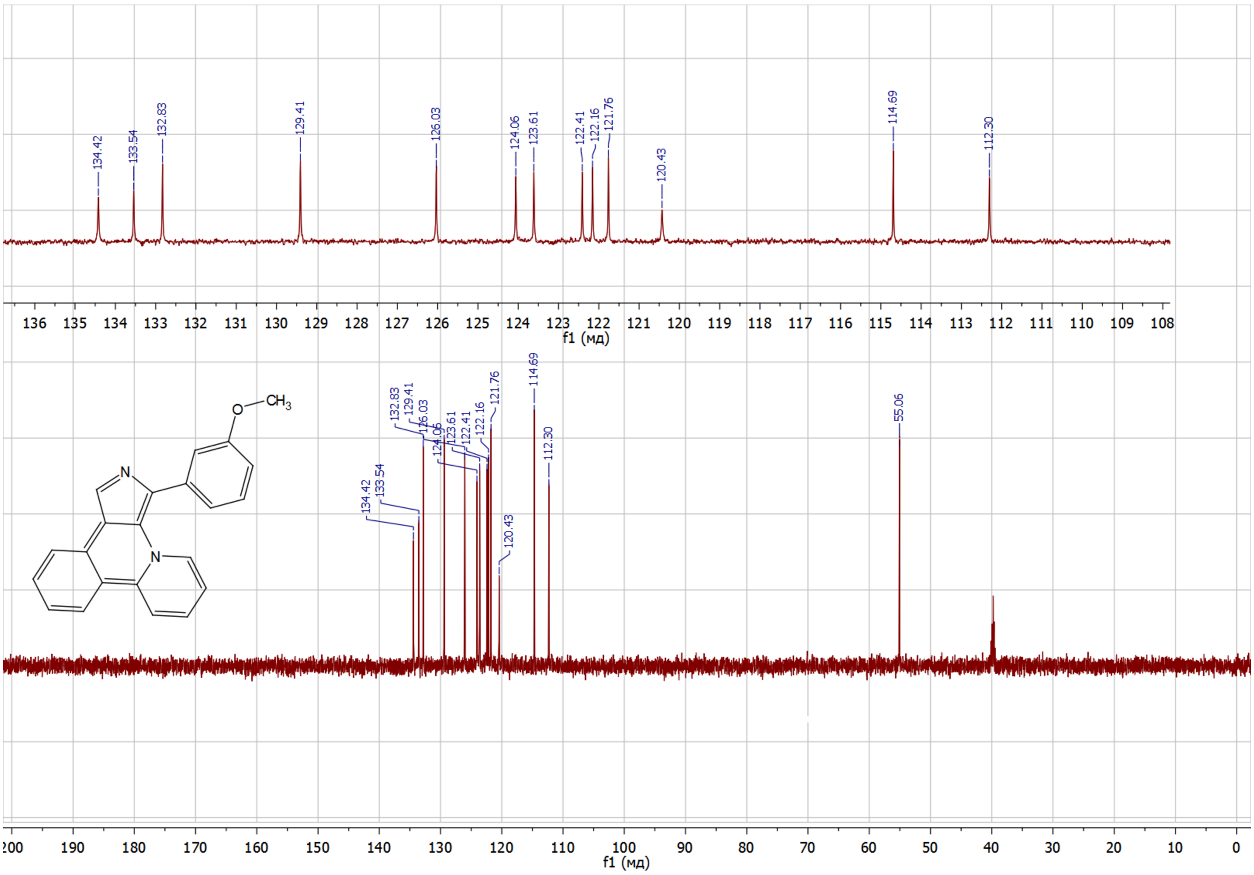

^{1}H NMR (400 MHz, DMSO-d_6) of 3-(2,4-dimethoxyphenyl)pyrido[2,1-*a*]pyrrolo[3,4-*c*]isoquinoline (**6i**)

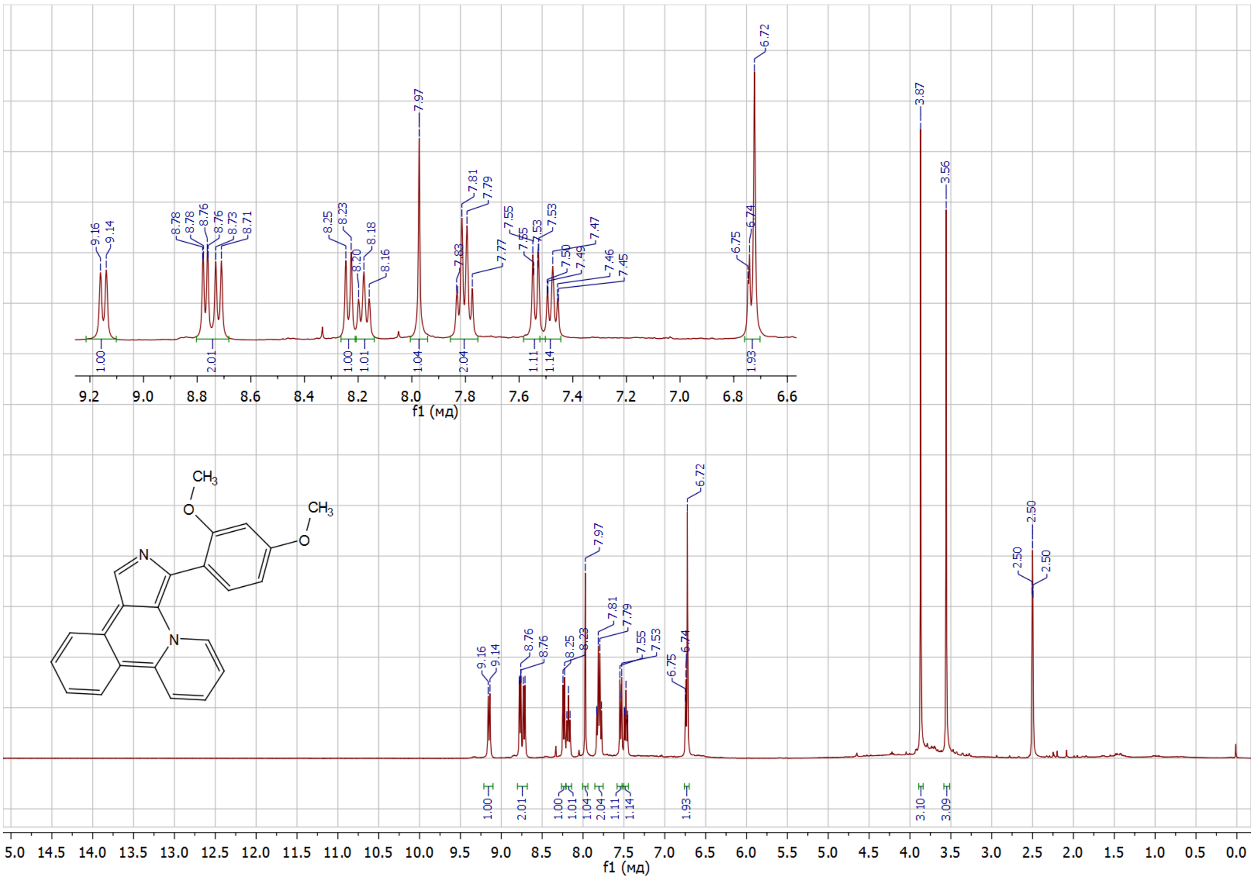

^{13}C{1H} NMR (101 MHz, DMSO-d_6) of 3-(2,4-dimethoxyphenyl)pyrido[2,1-*a*]pyrrolo[3,4-*c*]isoquinoline (**6i**)

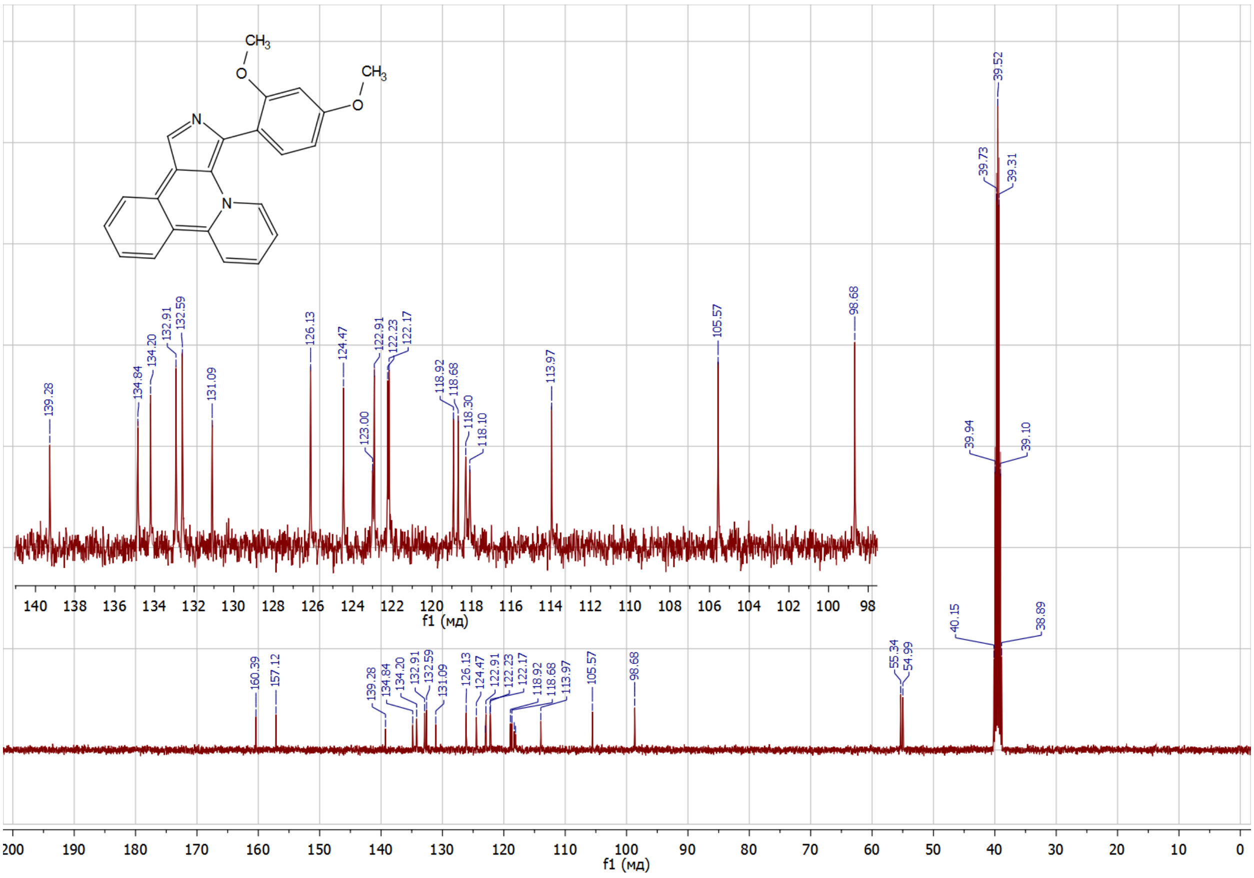

^{13}C DEPT135 NMR (101 MHz, DMSO-d_6) of 3-(2,4-dimethoxyphenyl)pyrido[2,1-*a*]pyrrolo[3,4-*c*]isoquinoline (**6i**)

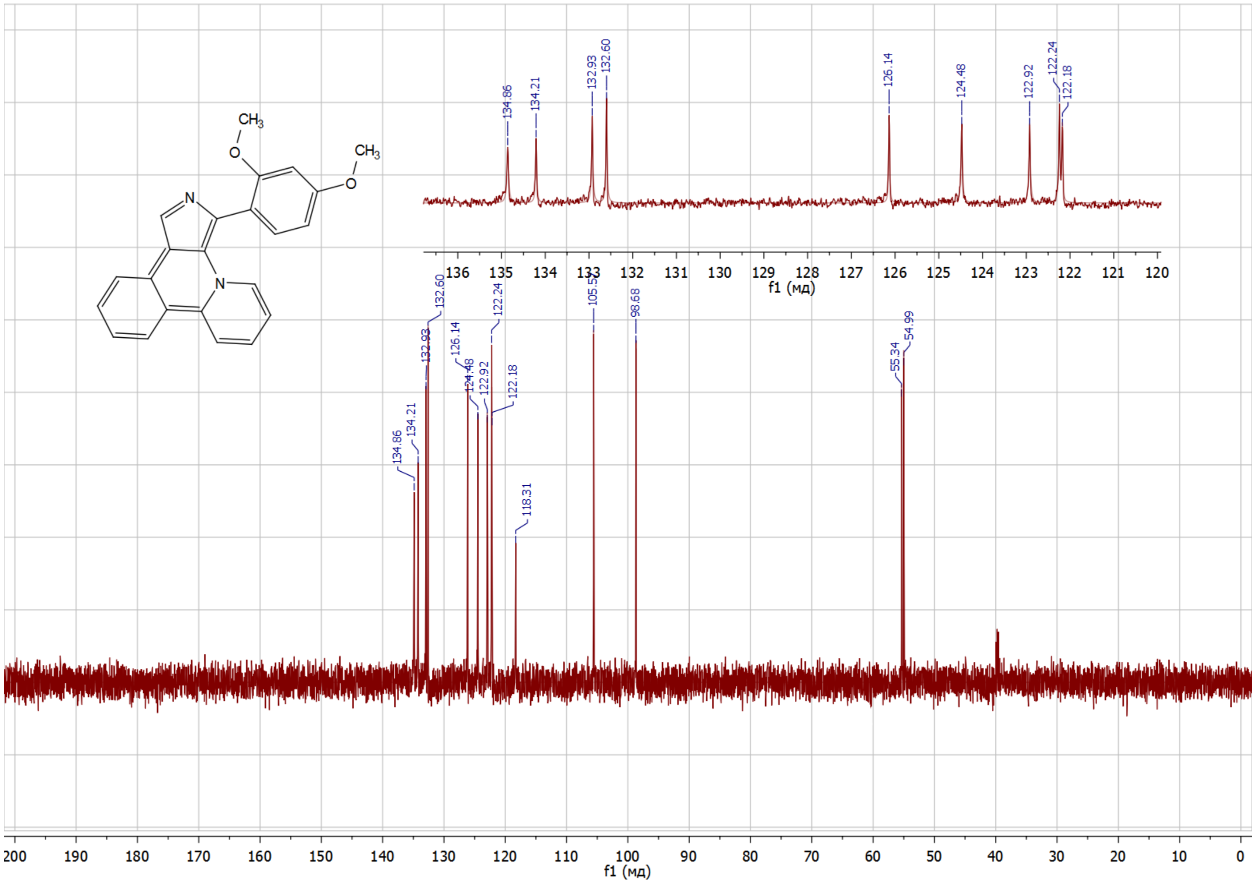

^{1}H NMR (400 MHz, DMSO-d_6) of 4-(pyrido[2,1-*a*]pyrrolo[3,4-*c*]isoquinolin-3-yl)benzonitrile (**6l**)

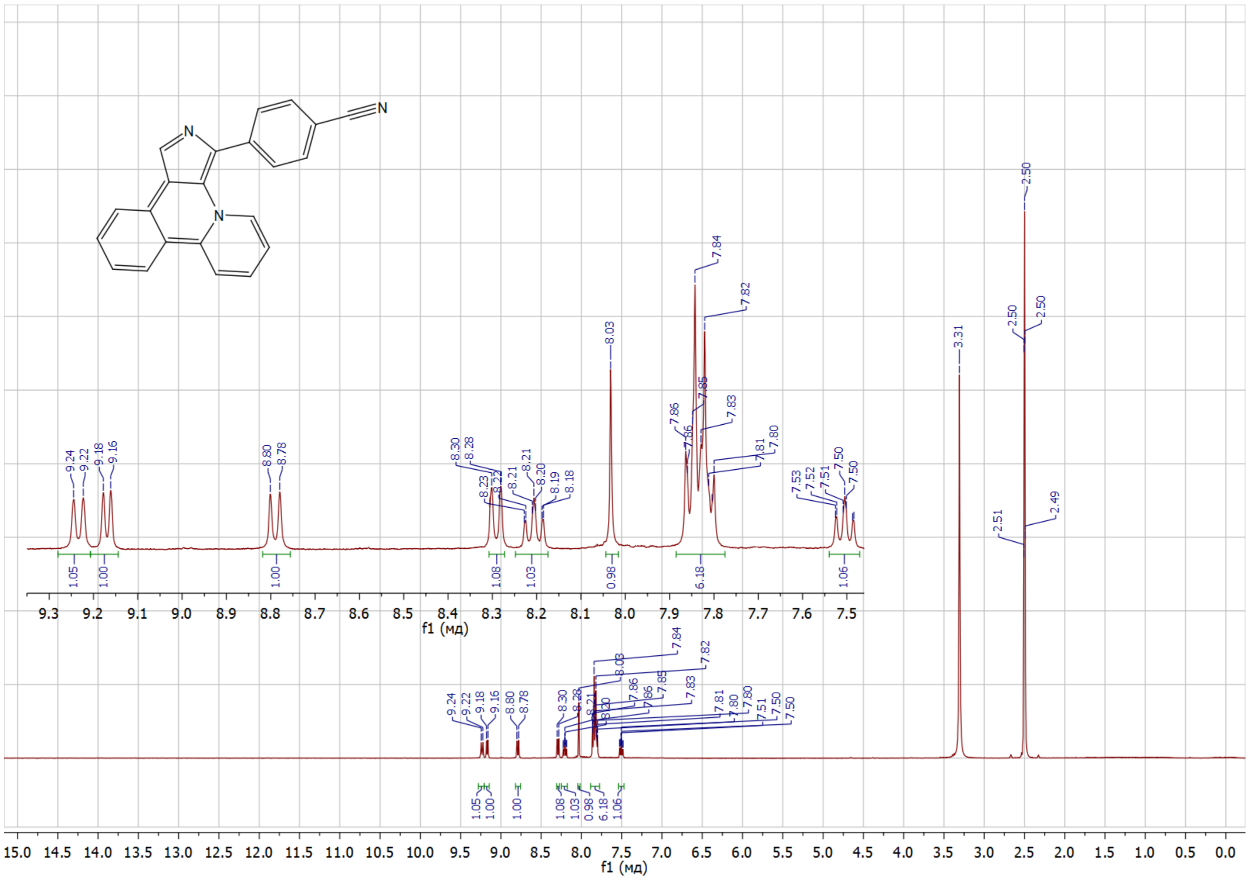

^{13}C{1H} NMR (101 MHz, DMSO-d_6) of 4-(pyrido[2,1-*a*]pyrrolo[3,4-*c*]isoquinolin-3-yl)benzonitrile (**6l**)

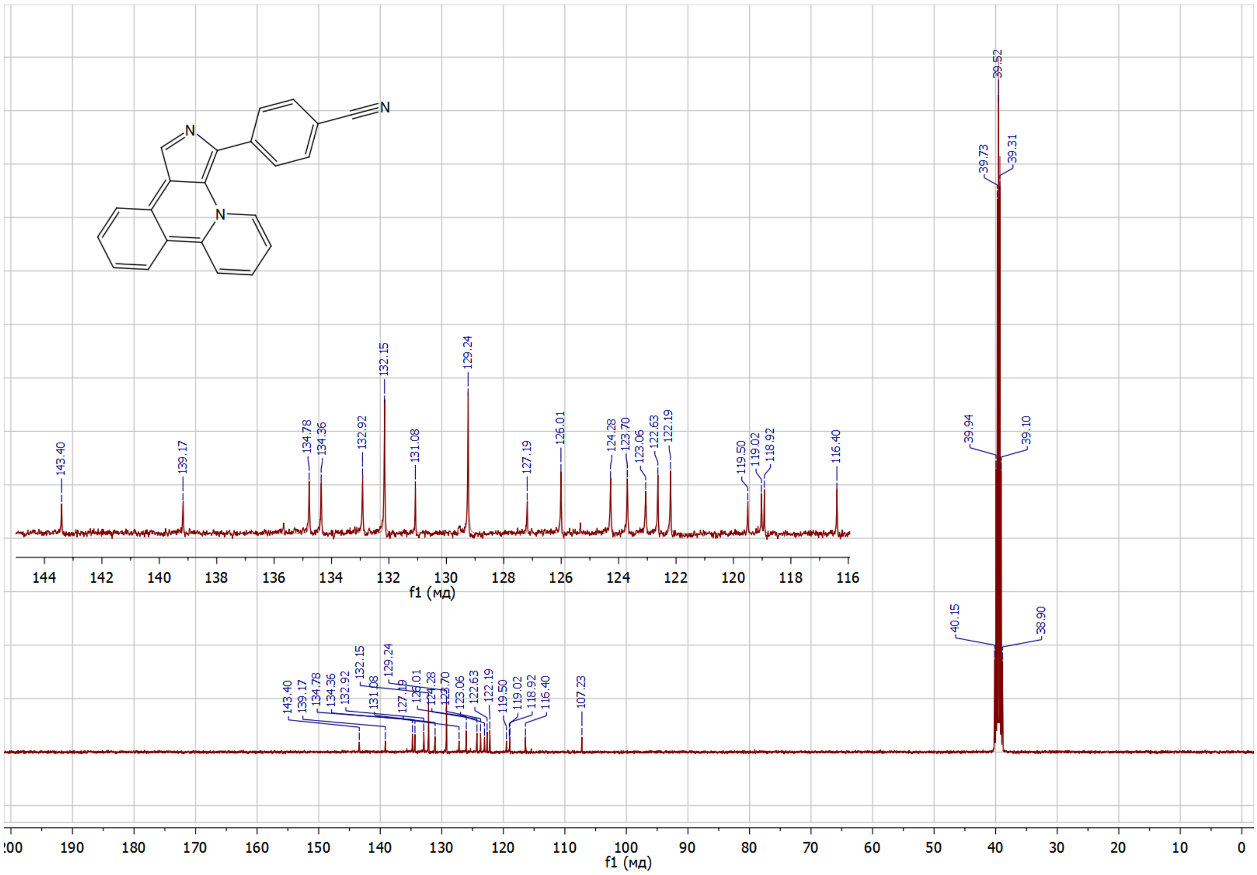

^{13}C DEPT135 NMR (101 MHz, DMSO-d_6) of 4-(pyrido[2,1-*a*]pyrrolo[3,4-*c*]isoquinolin-3-yl)benzonitrile (**6l**)

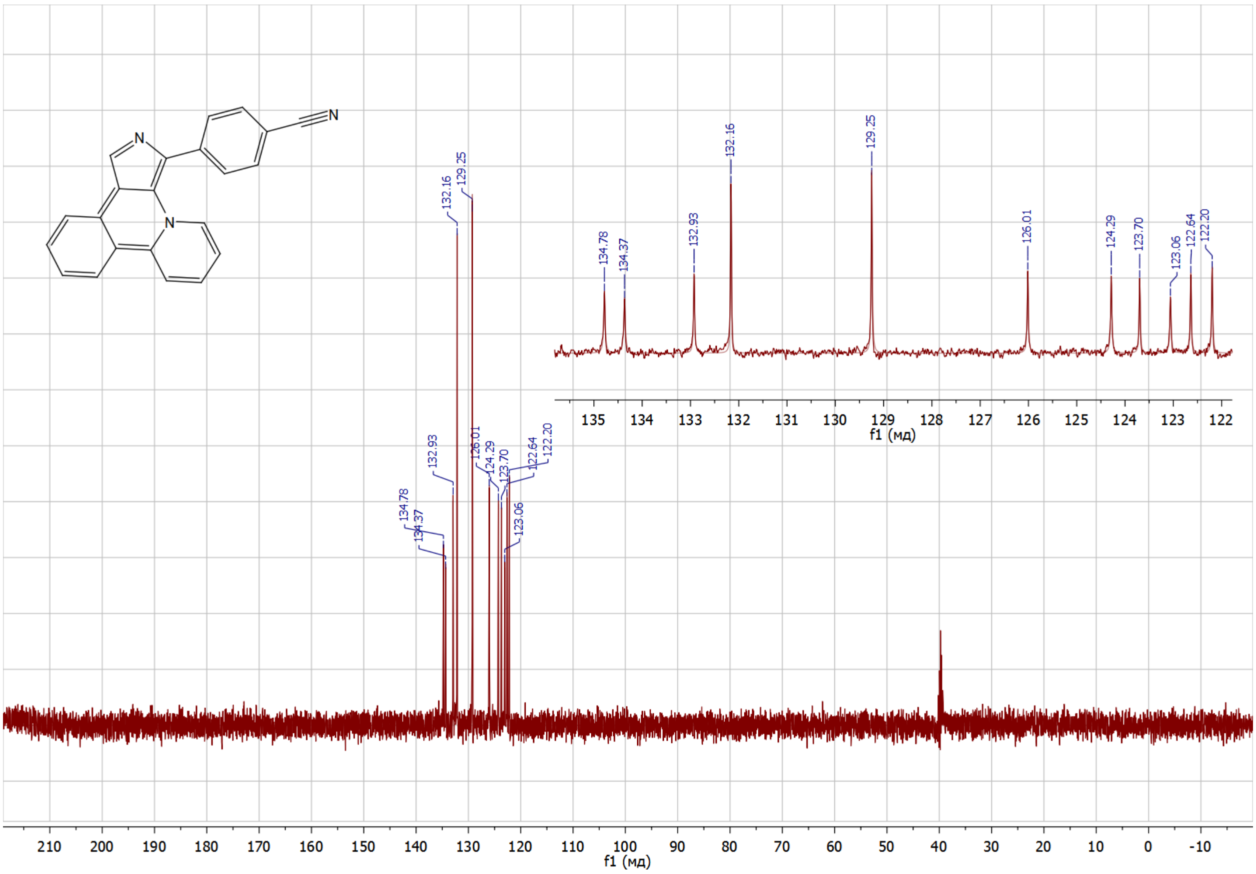

^{1}H NMR (400 MHz, DMSO-d_6) of 3-(pyridin-2-yl)pyrido[2,1-*a*]pyrrolo[3,4-*c*]isoquinoline (**6m**)

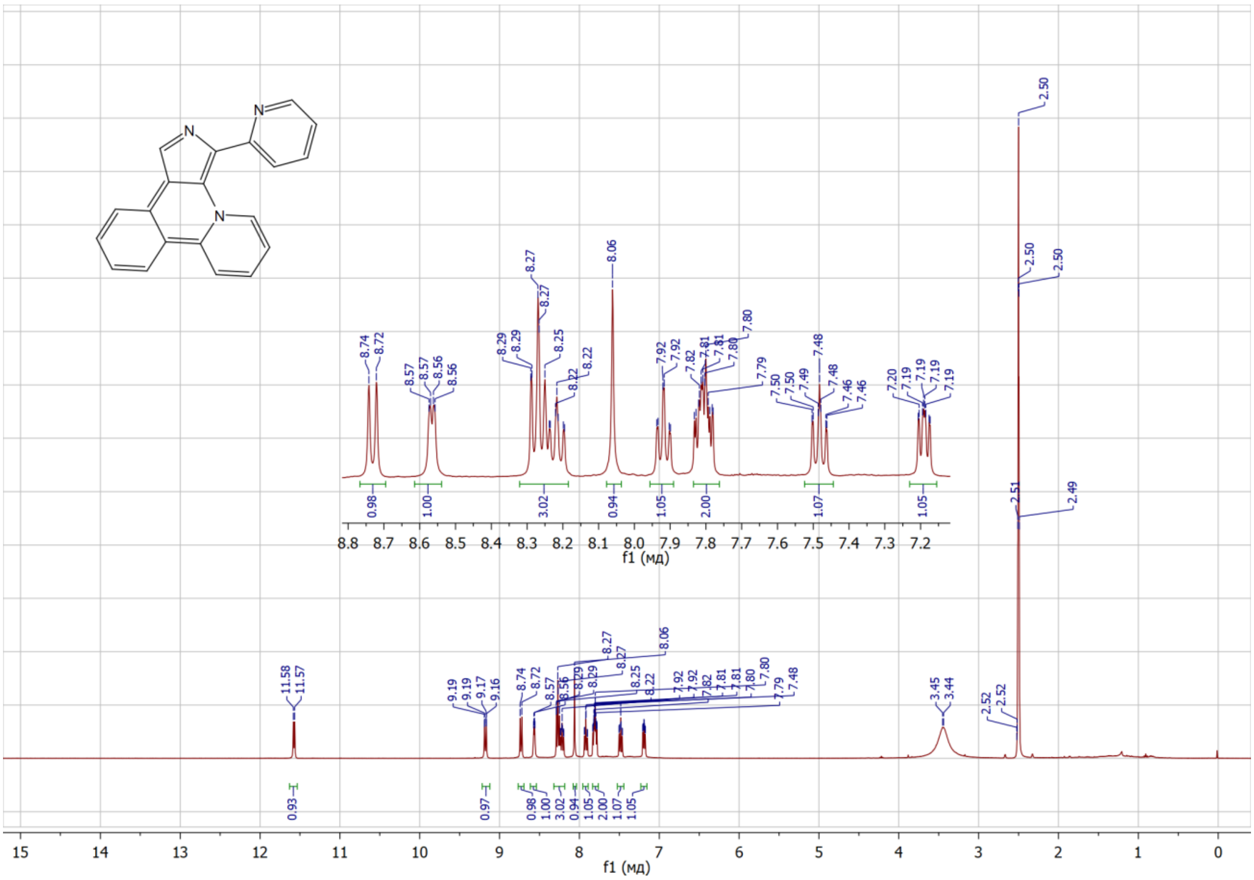

$^{13}C\{1H\}$ NMR (101 MHz, DMSO-d_6) of 3-(pyridin-2-yl)pyrido[2,1-*a*]pyrrolo[3,4-*c*]isoquinoline (**6m**)

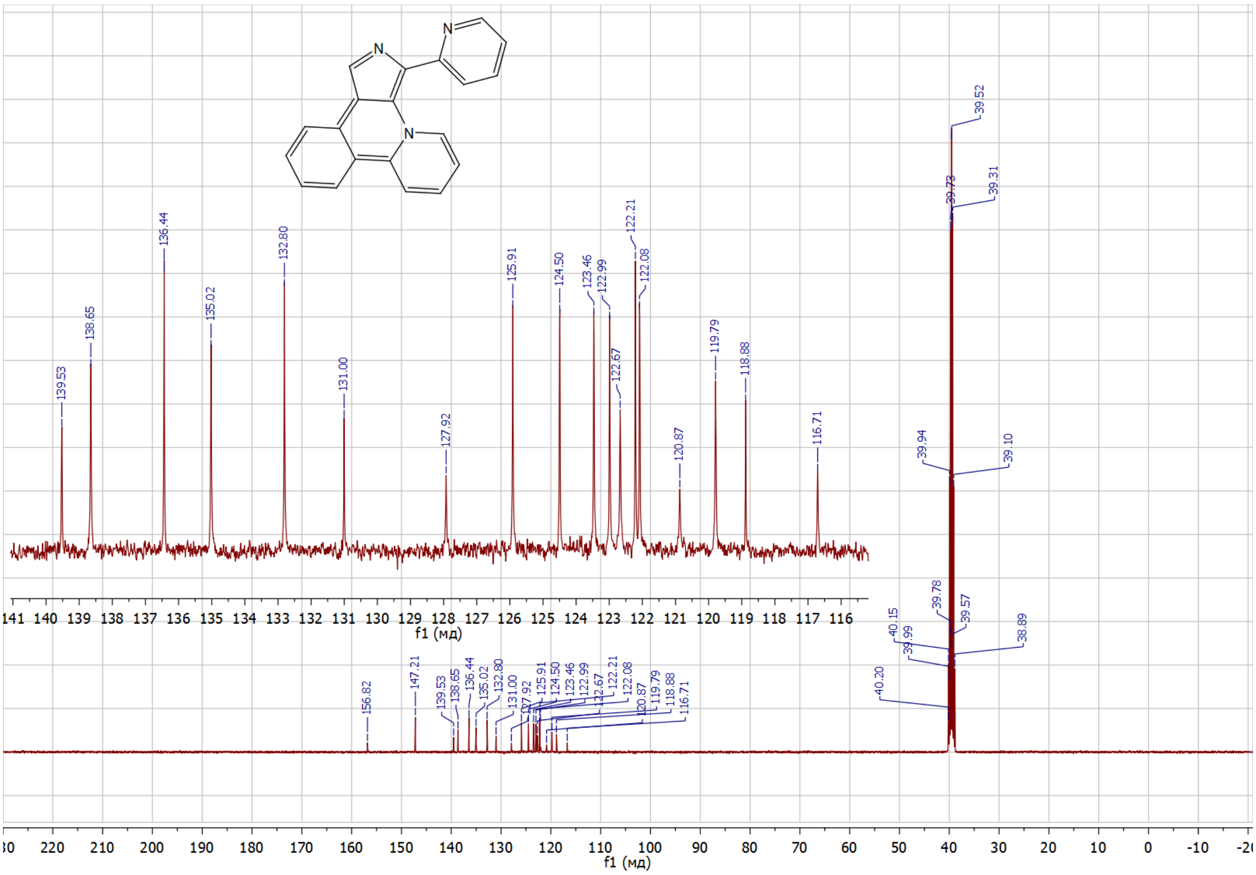

^{13}C DEPT135 NMR (101 MHz, DMSO-d_6) of 3-(pyridin-2-yl)pyrido[2,1-*a*]pyrrolo[3,4-*c*]isoquinoline (**6m**)

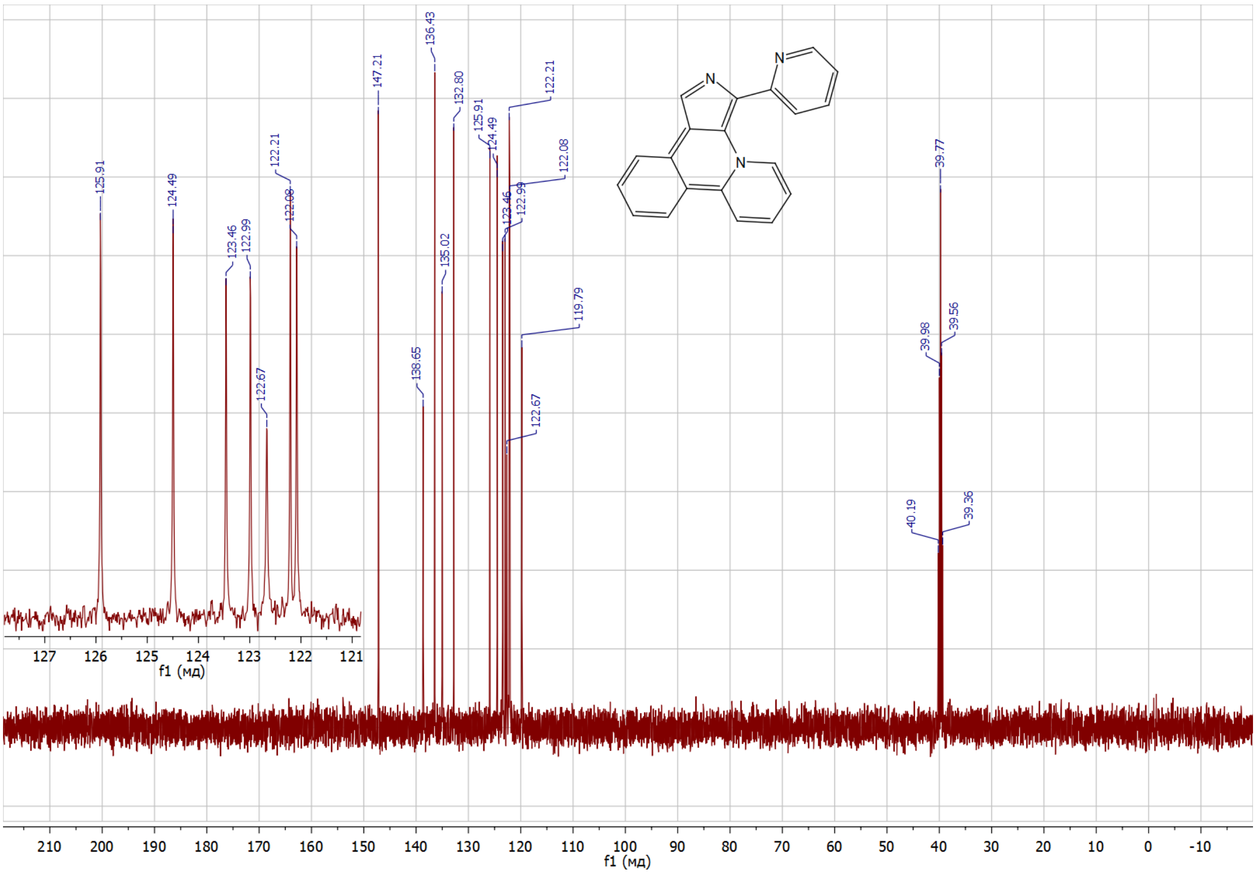

^{1}H NMR (400 MHz, DMSO-d_6) of 7-methyl-3-phenylpyrido[2,1-*a*]pyrrolo[3,4-*c*]isoquinoline (**6n**)

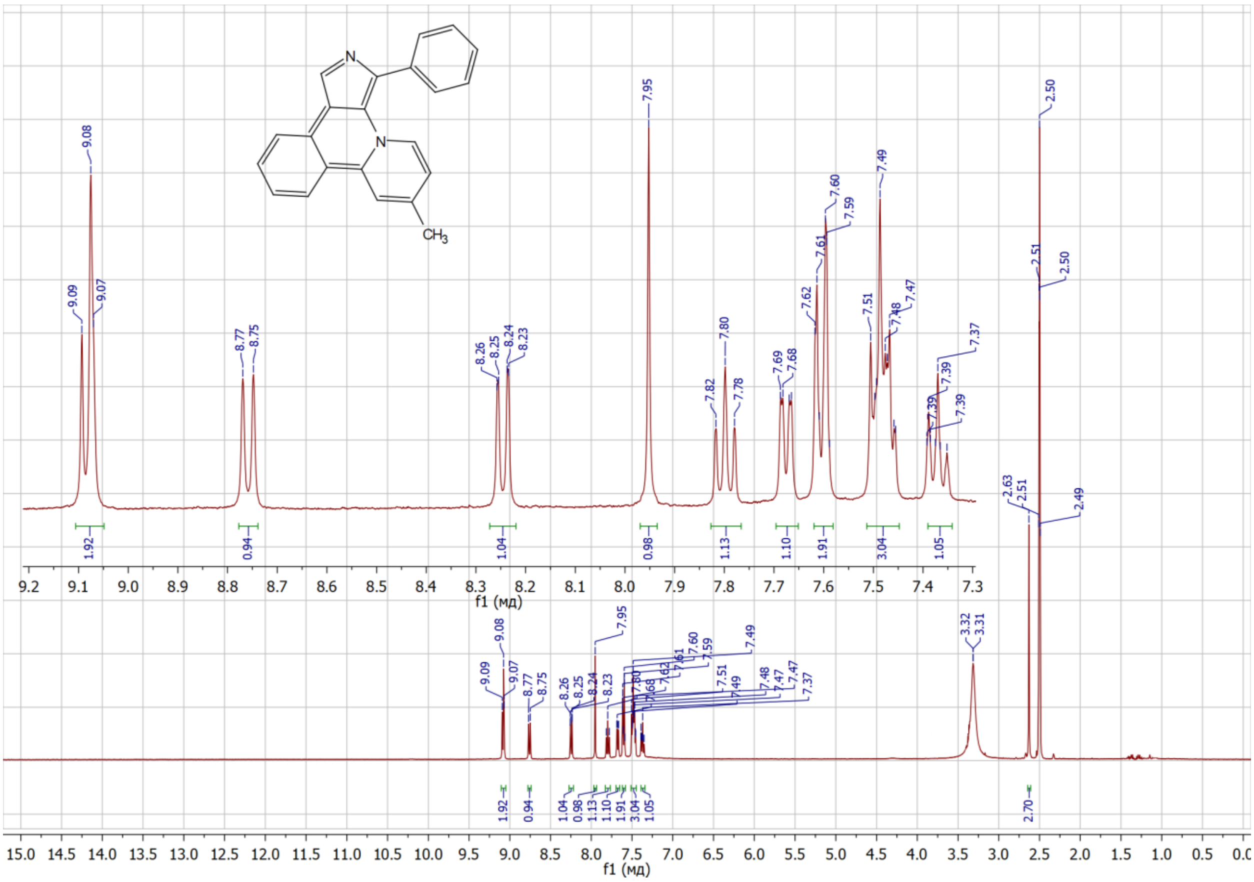

^{13}C{1H} NMR (101 MHz, DMSO-d_6) of 7-methyl-3-phenylpyrido[2,1-*a*]pyrrolo[3,4-*c*]isoquinoline (**6n**)

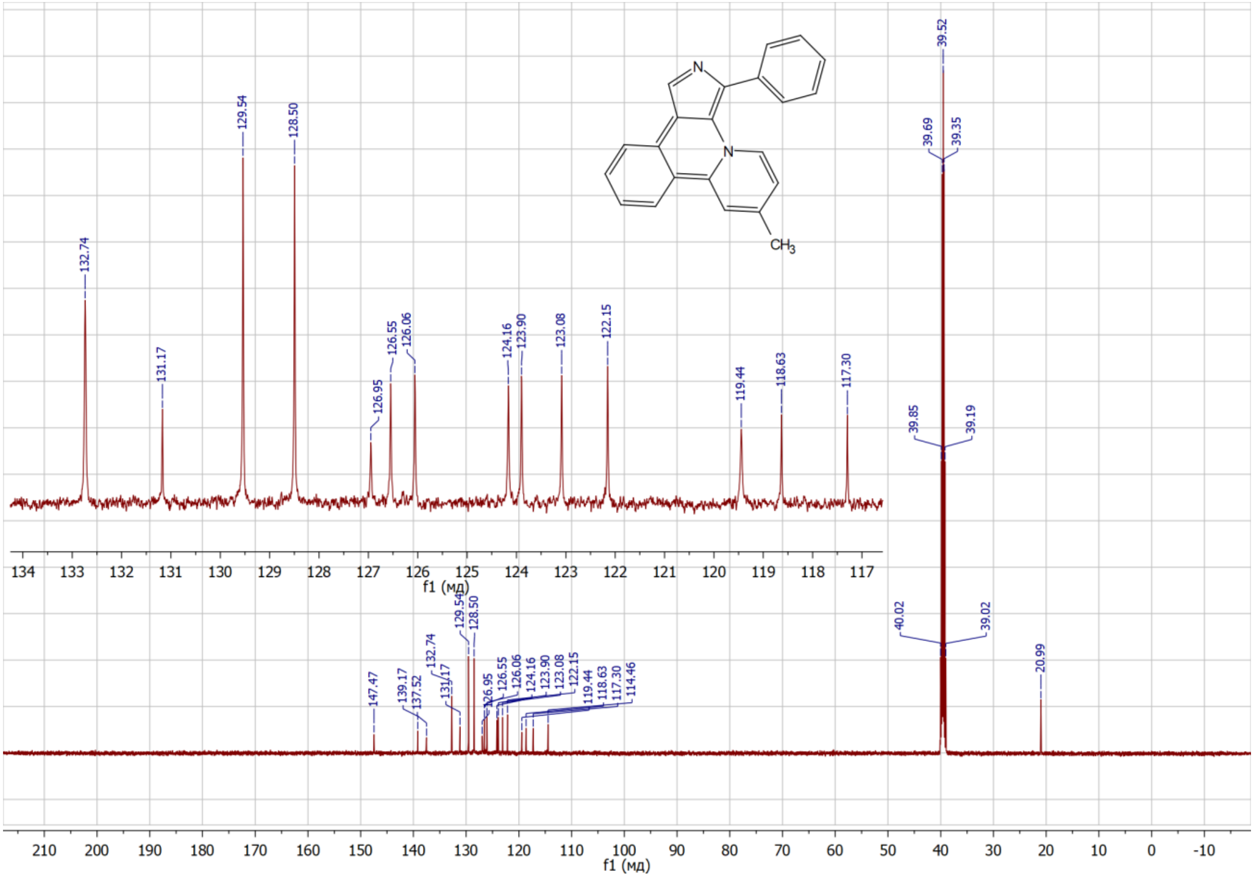

^{13}C DEPT135 NMR (101 MHz, DMSO-d_6) of 7-methyl-3-phenylpyrido[2,1-*a*]pyrrolo[3,4-*c*]isoquinoline (**6n**)

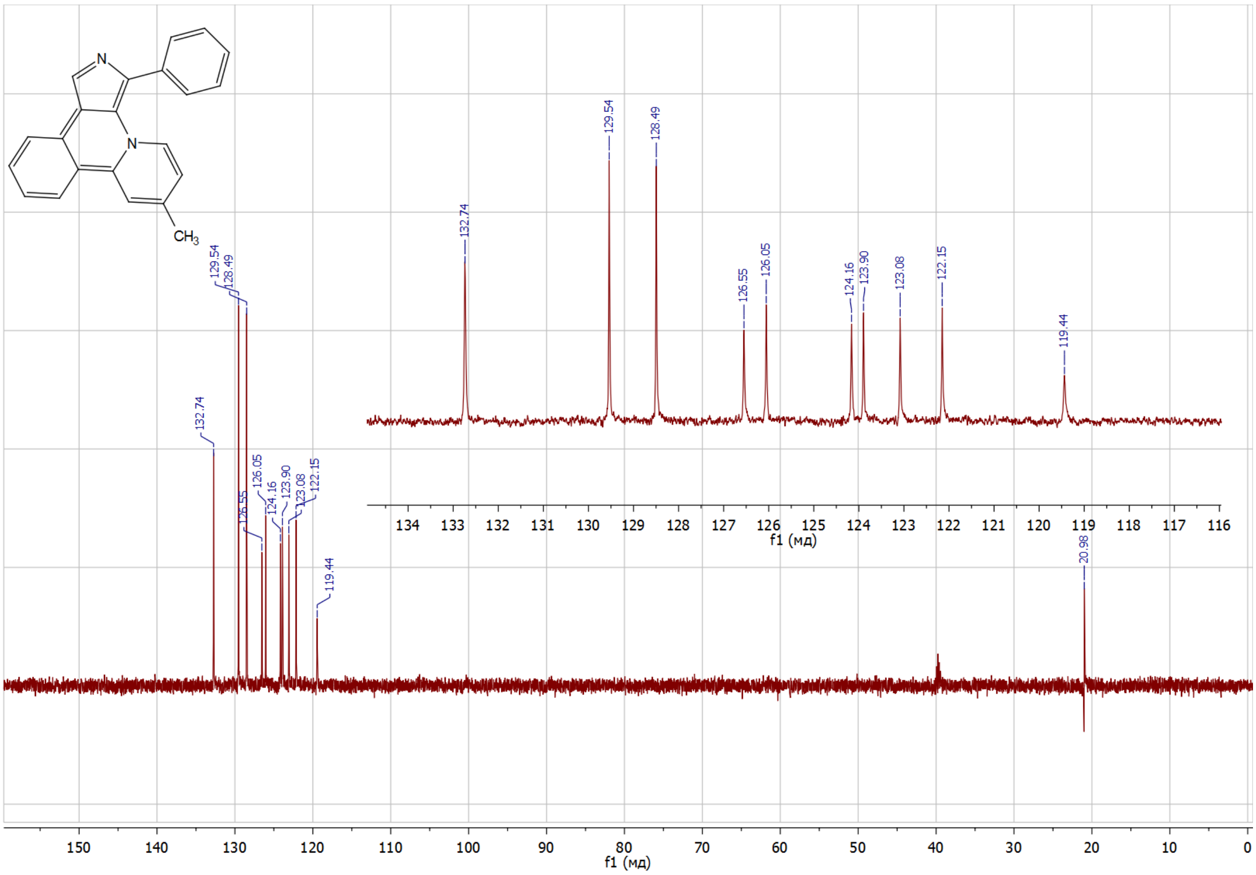

^{1}H NMR (400 MHz, DMSO-d_6) of 7-methoxy-3-phenylpyrido[2,1-*a*]pyrrolo[3,4-*c*]isoquinoline (**6o**)

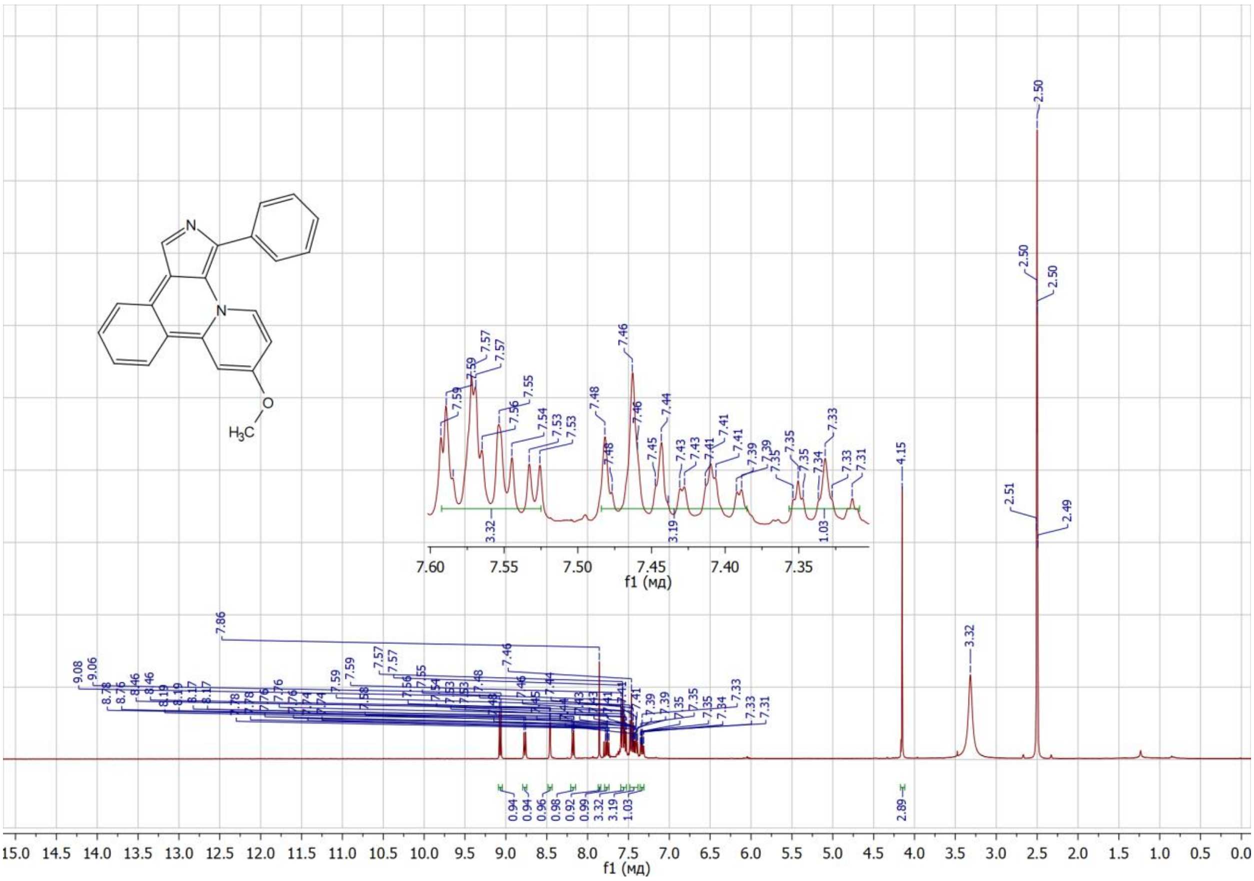

$^{13}C\{1H\}$ NMR (101 MHz, DMSO-d_6) of 7-methoxy-3-phenylpyrido[2,1-*a*]pyrrolo[3,4-*c*]isoquinoline (**6o**)

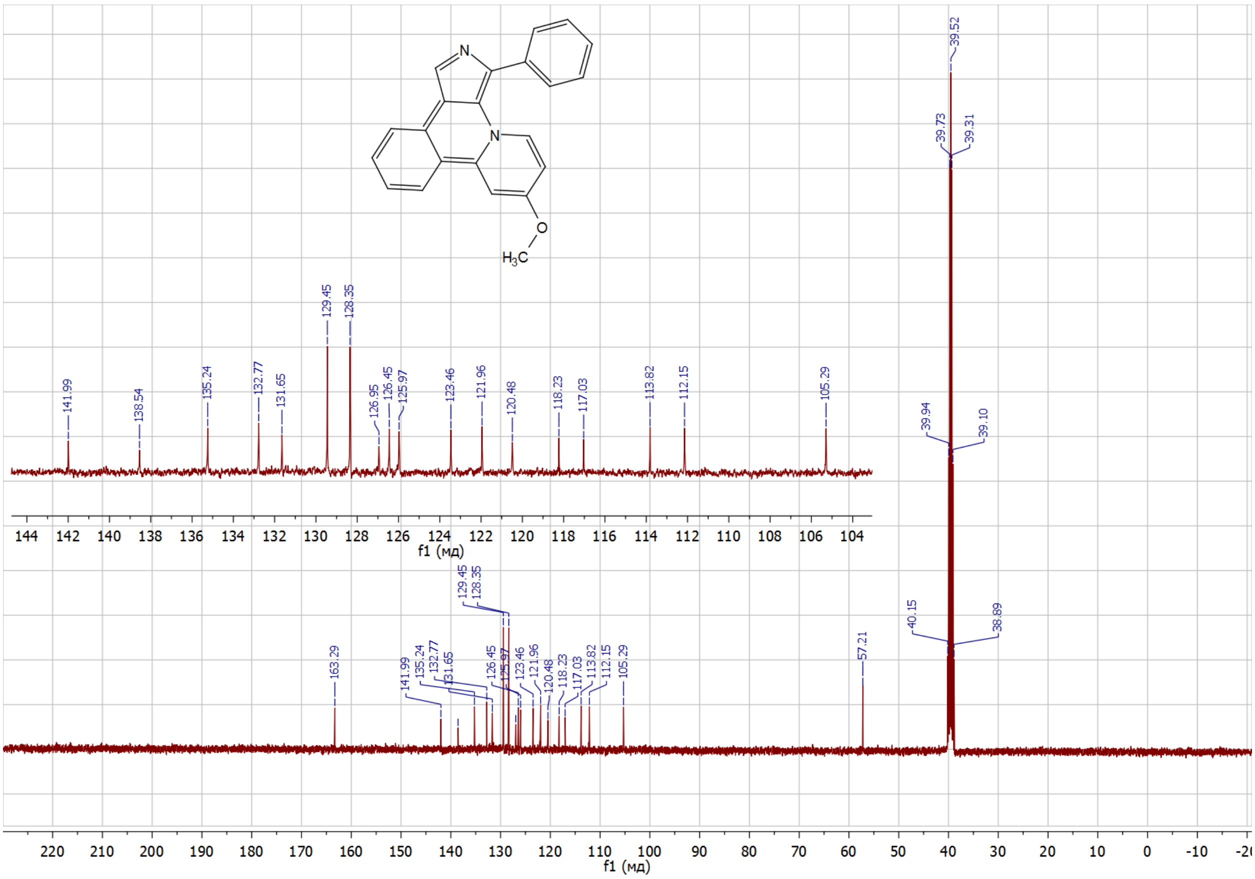

^{13}C DEPT135 NMR (101 MHz, DMSO-d_6) of 7-methoxy-3-phenylpyrido[2,1-*a*]pyrrolo[3,4-*c*]isoquinoline (**6o**)

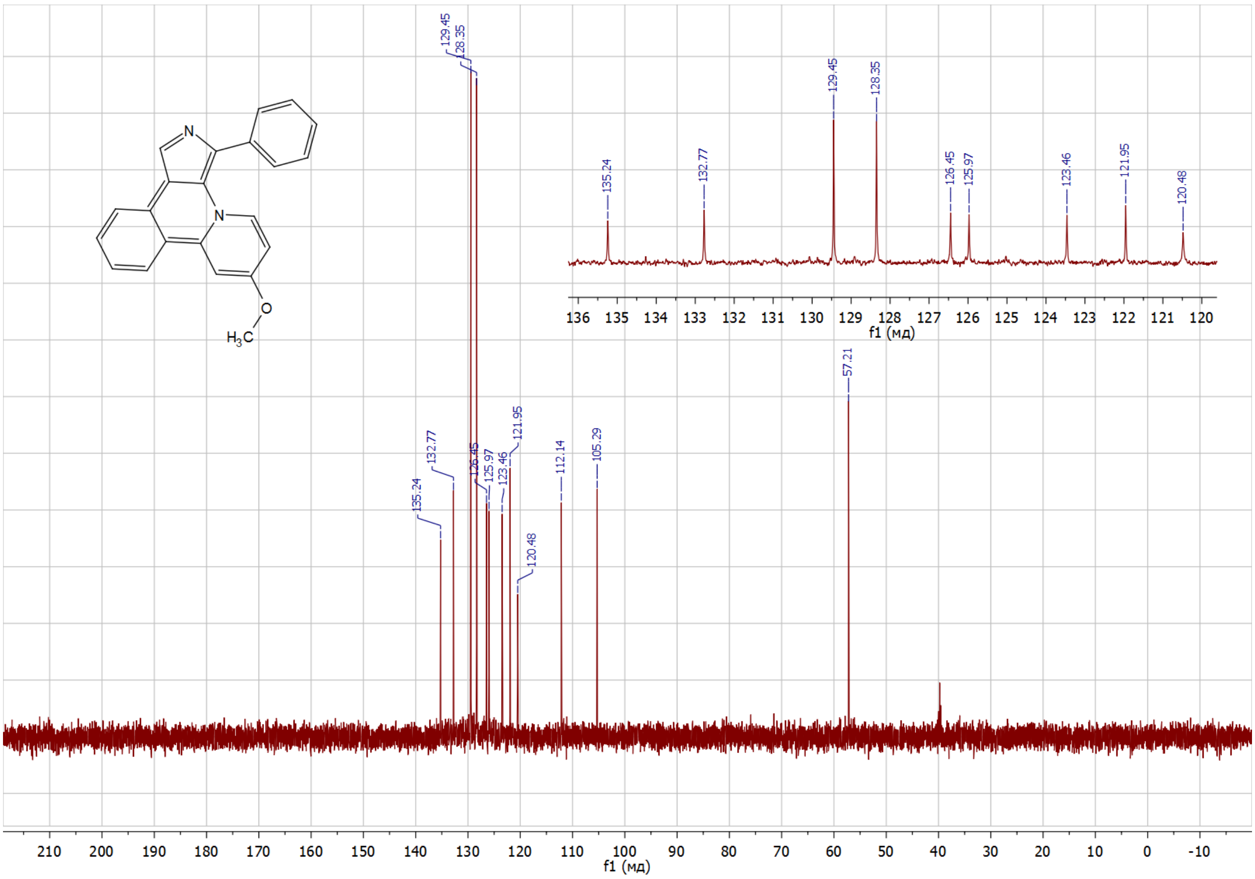

^{1}H NMR (400 MHz, DMSO-d_6) of N,N-dimethyl-3-phenylpyrido[2,1-*a*]pyrrolo[3,4-*c*]isoquinolin-7-amine (**6p**)

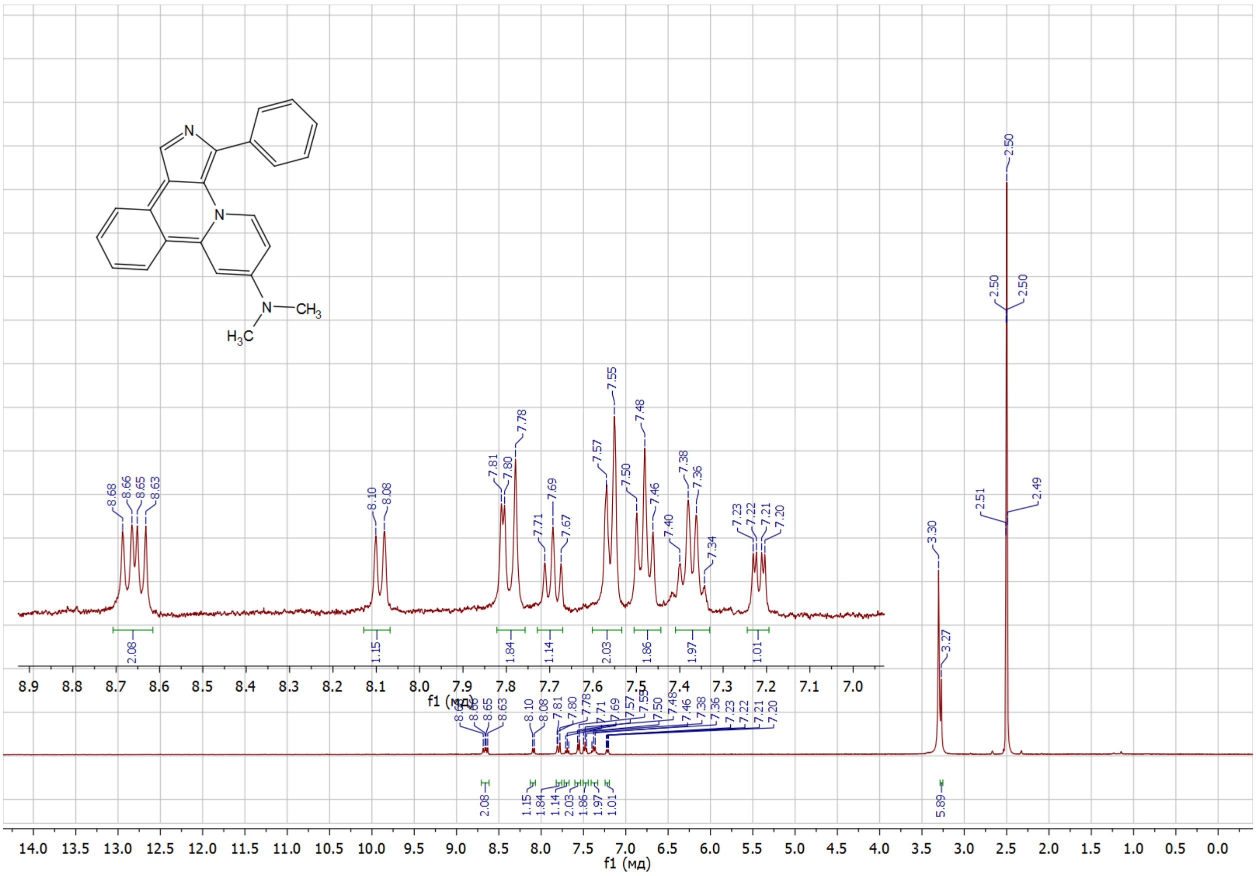

$^{13}C\{^{1}H\}$ NMR (101 MHz, DMSO-d_6) of N,N-dimethyl-3-phenylpyrido[2,1-*a*]pyrrolo[3,4-*c*]isoquinolin-7-amine (**6p**)

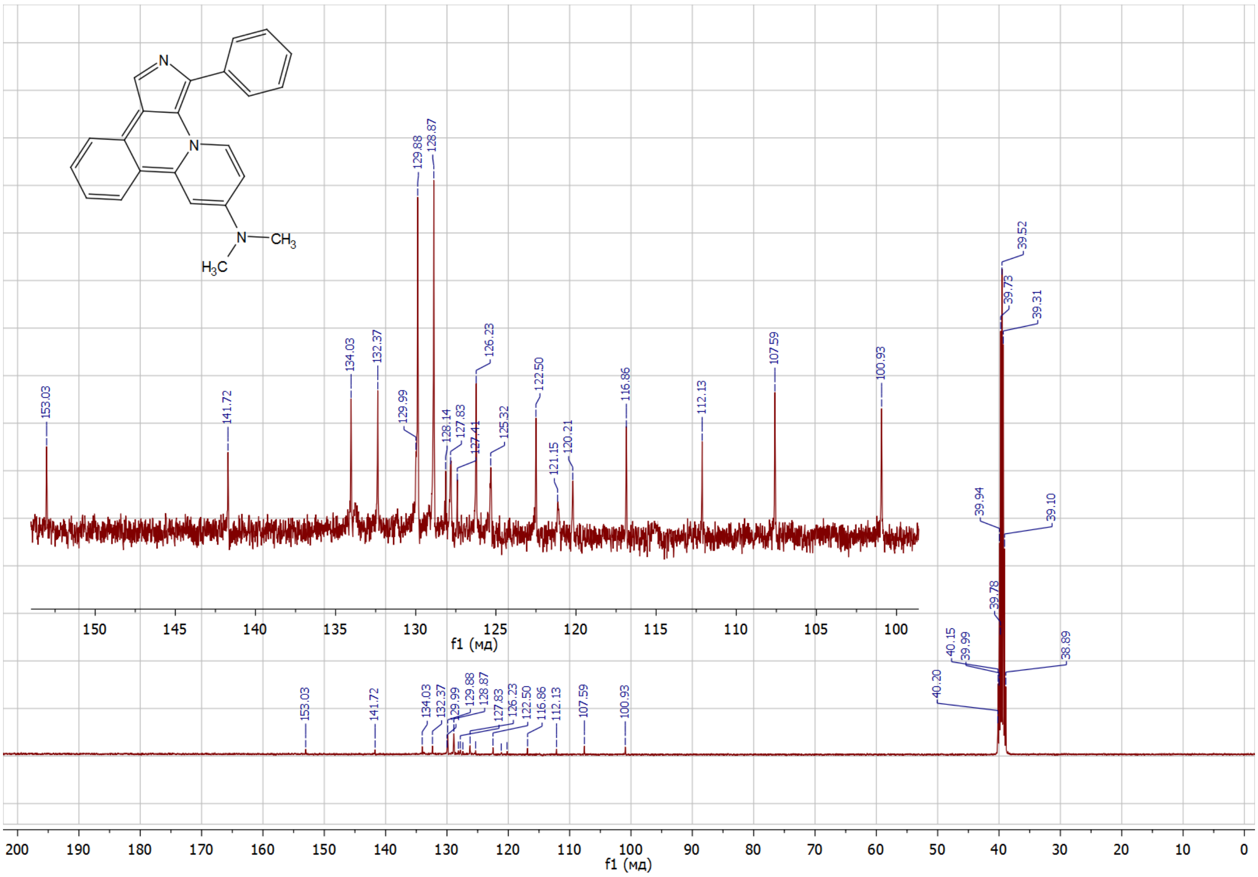

^{13}C DEPT135 NMR (101 MHz, DMSO-d_6) of N,N-dimethyl-3-phenylpyrido[2,1-*a*]pyrrolo[3,4-*c*]isoquinolin-7-amine (**6p**)

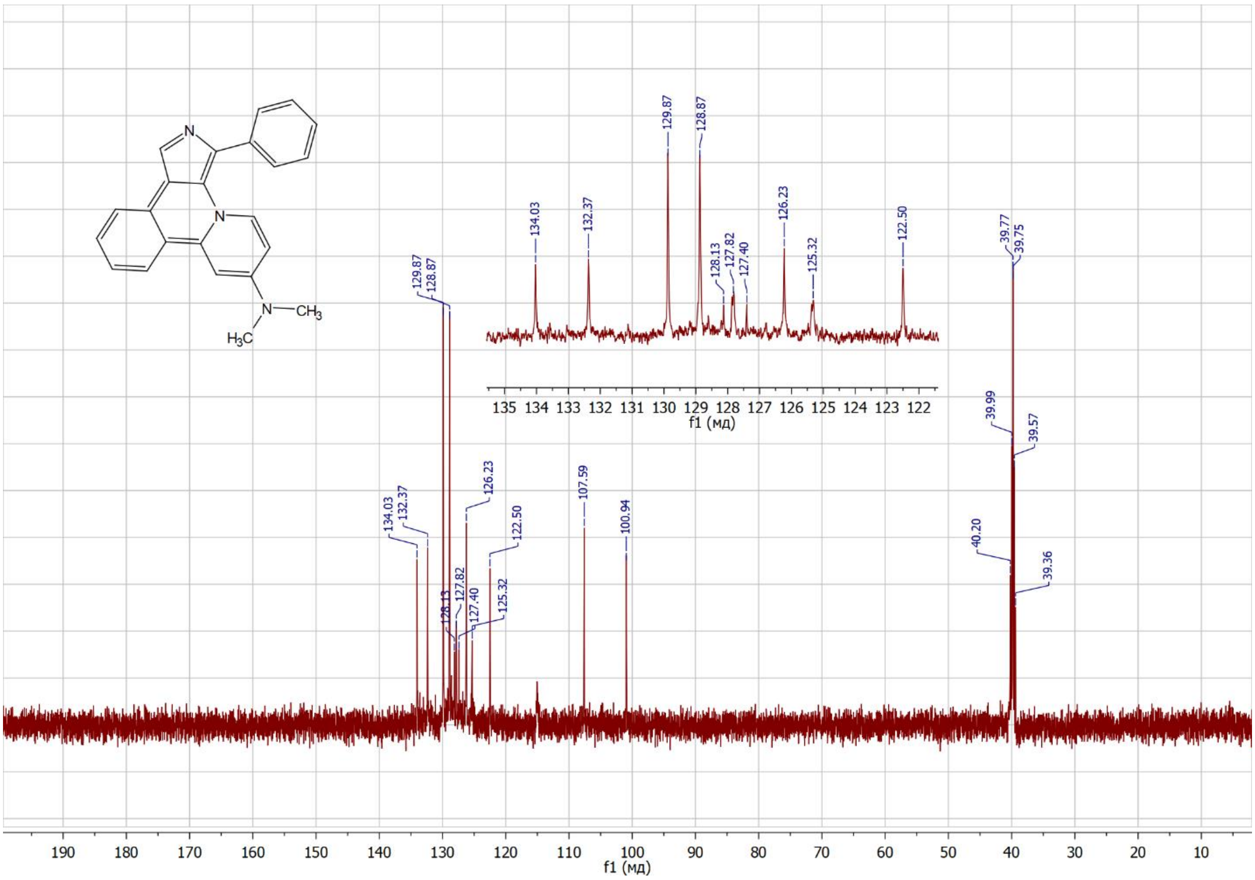

^{1}H NMR (400 MHz, DMSO-d_6) of 3,7-diphenylpyrido[2,1-*a*]pyrrolo[3,4-*c*]isoquinoline (**6q**)

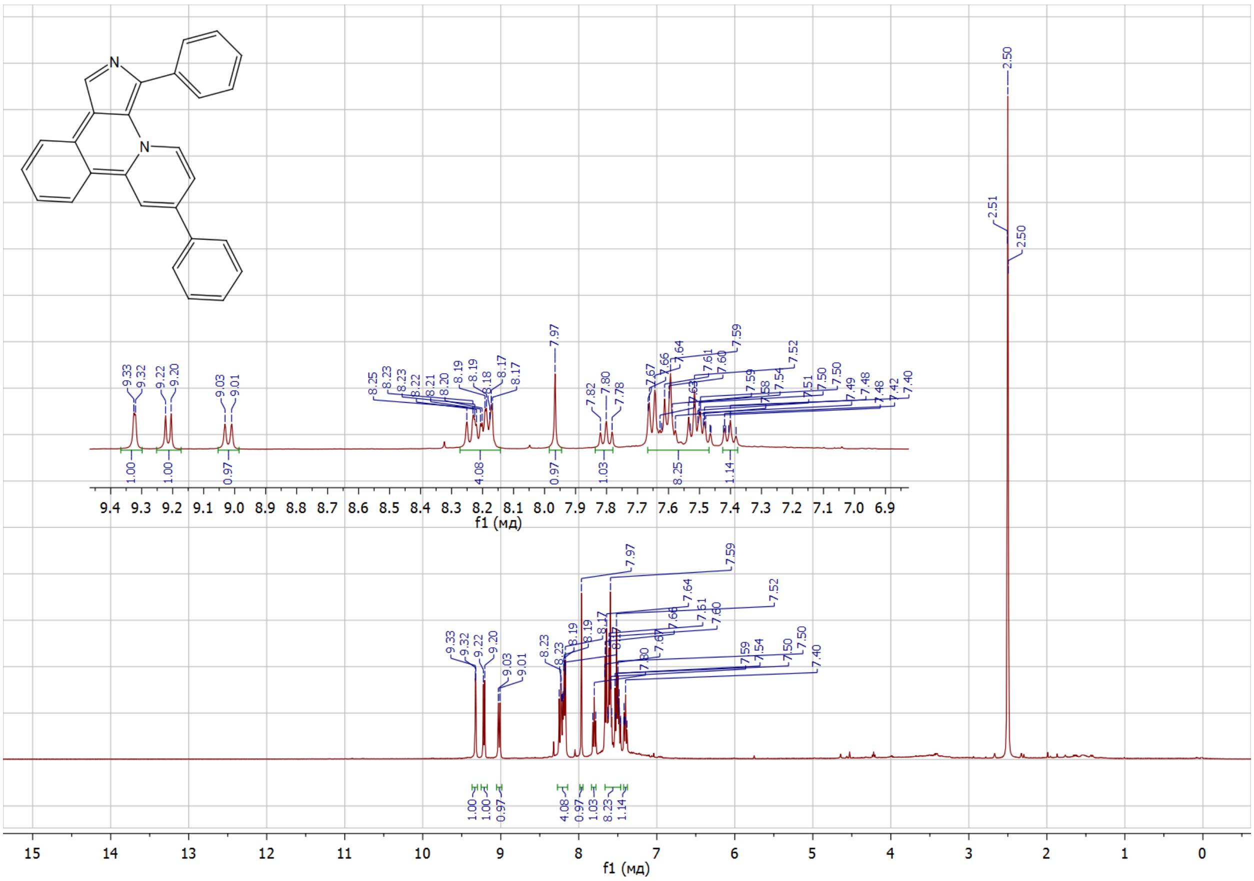

$^{13}C\{1H\}$ NMR (101 MHz, DMSO-d_6) of 3,7-diphenylpyrido[2,1-*a*]pyrrolo[3,4-*c*]isoquinoline (**6q**)

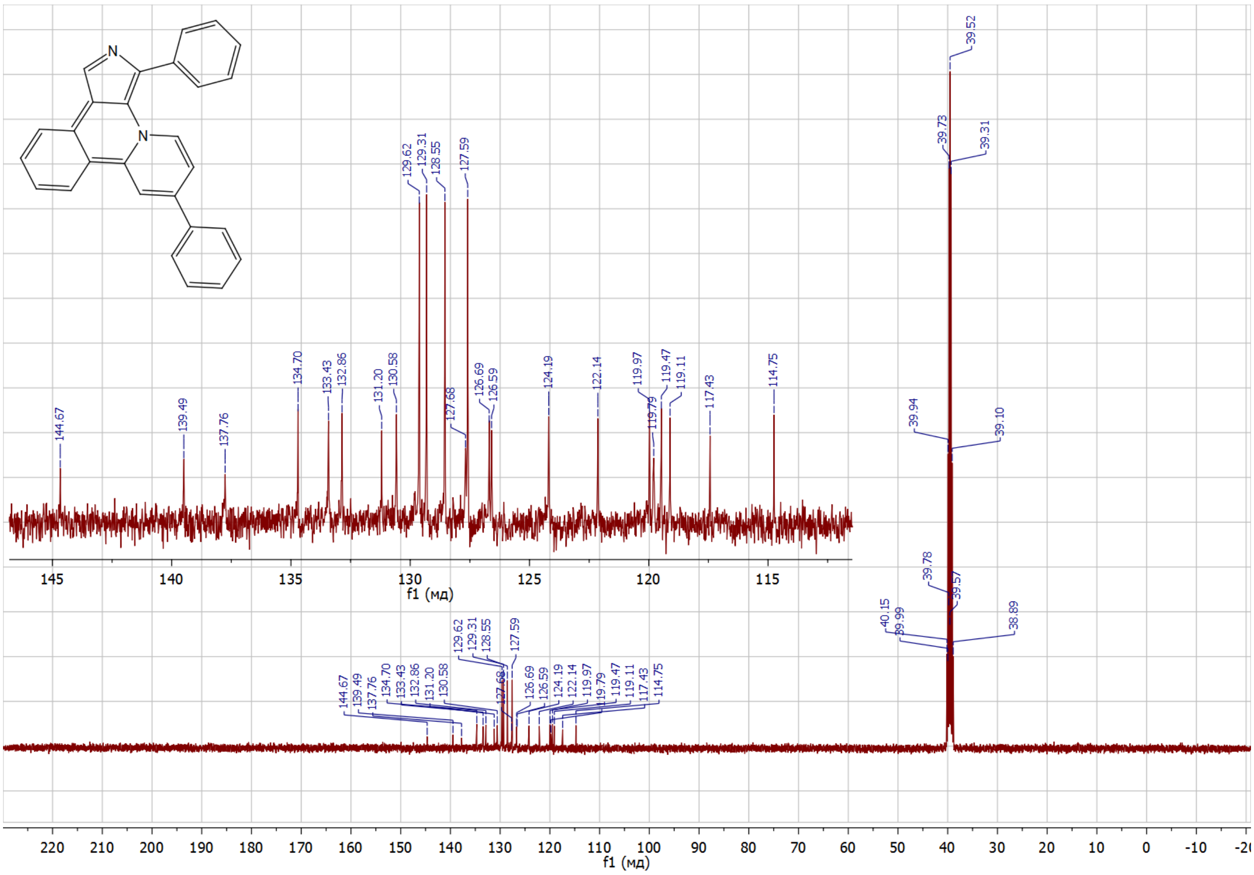

^{13}C DEPT135 NMR (101 MHz, DMSO-d_6) of 3,7-diphenylpyrido[2,1-*a*]pyrrolo[3,4-*c*]isoquinoline (**6q**)

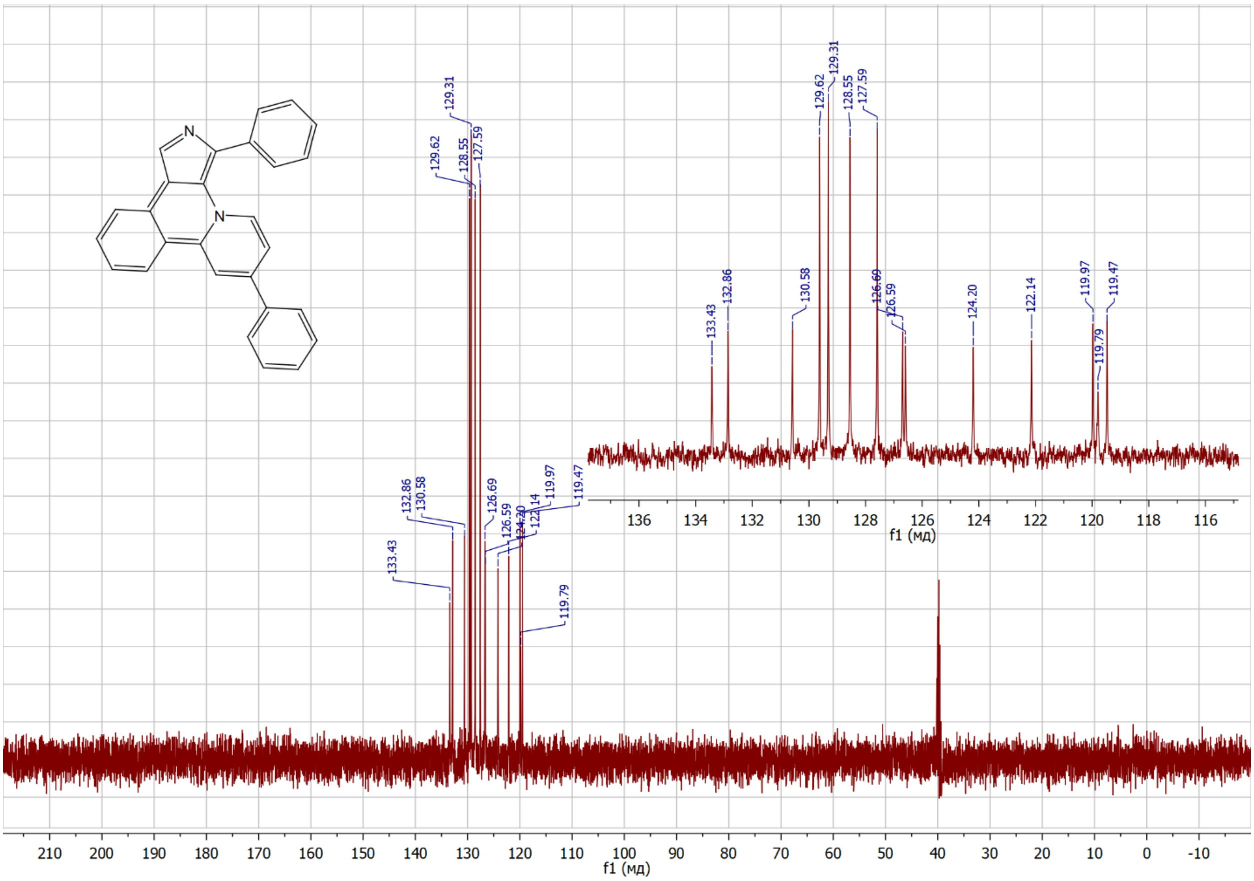

^{1}H NMR (400 MHz, DMSO-d_6) of 7-(4-methoxyphenyl)-3-phenylpyrido[2,1-*a*]pyrrolo[3,4-*c*]isoquinoline (**6r**)

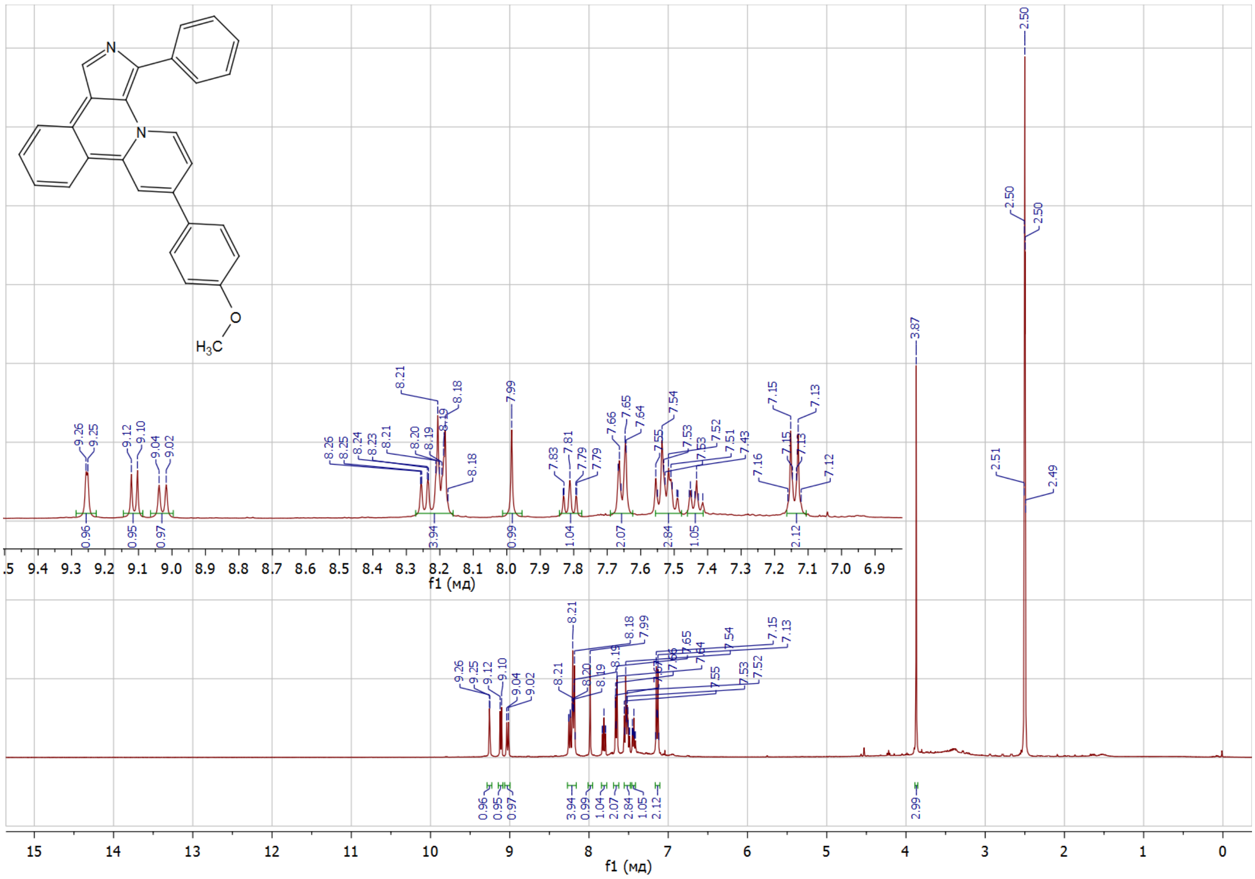

$^{13}C\{1H\}$ NMR (101 MHz, DMSO-d_6) of 7-(4-methoxyphenyl)-3-phenylpyrido[2,1-*a*]pyrrolo[3,4-*c*]isoquinoline (**6r**)

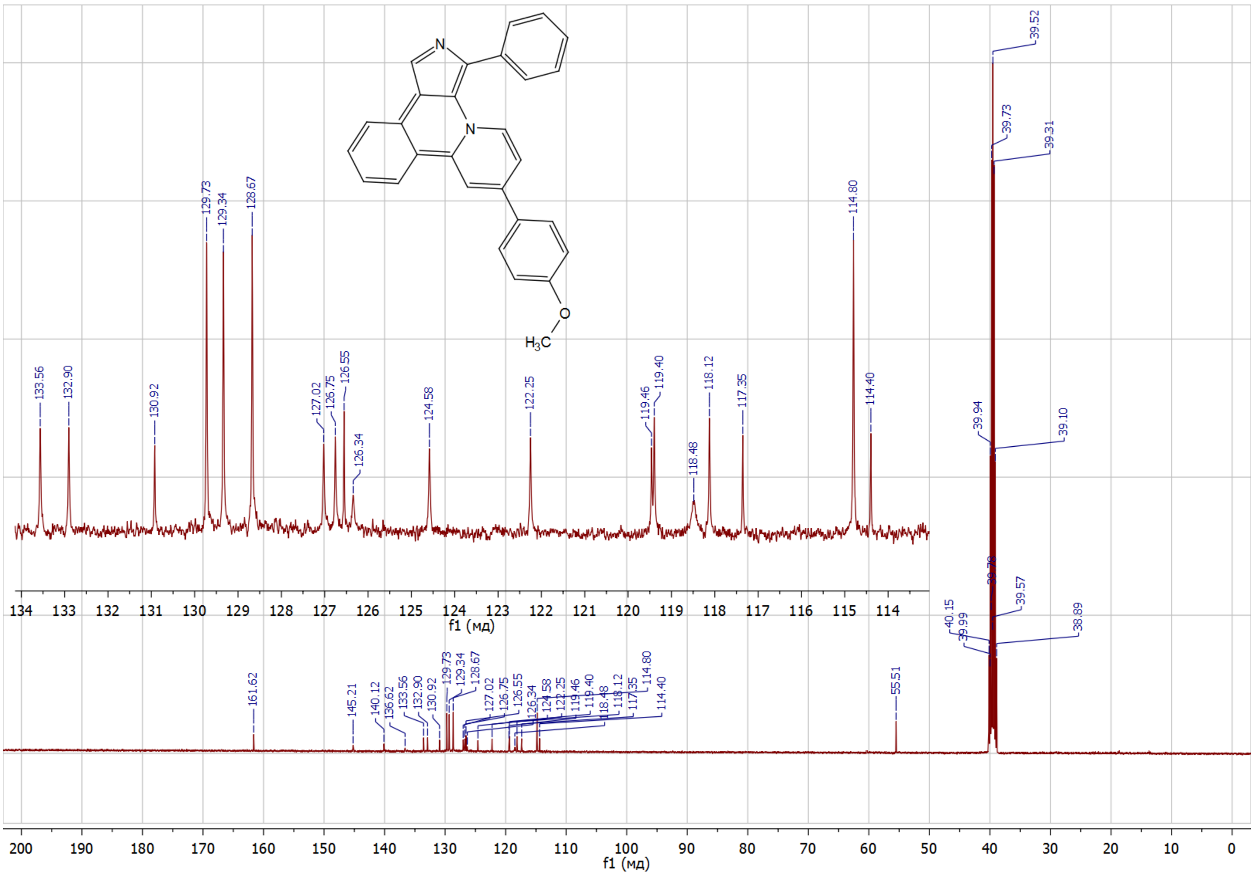

^{13}C DEPT135 NMR (101 MHz, DMSO-d_6) of 7-(4-methoxyphenyl)-3-phenylpyrido[2,1-*a*]pyrrolo[3,4-*c*]isoquinoline (**6r**)

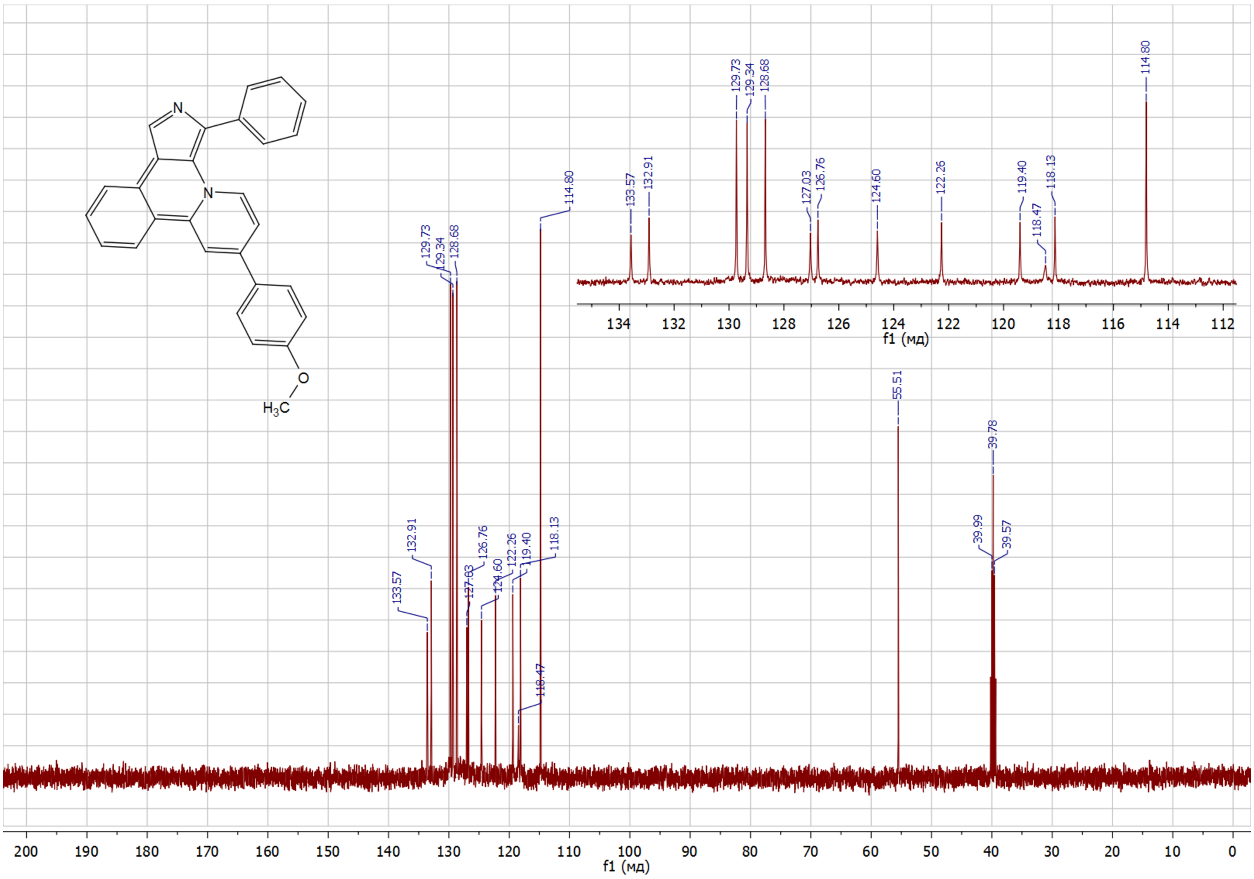

^{1}H NMR (400 MHz, DMSO-d_6) of 6,8-dimethyl-3-phenylpyrido[2,1-*a*]pyrrolo[3,4-*c*]isoquinoline (**6s**)

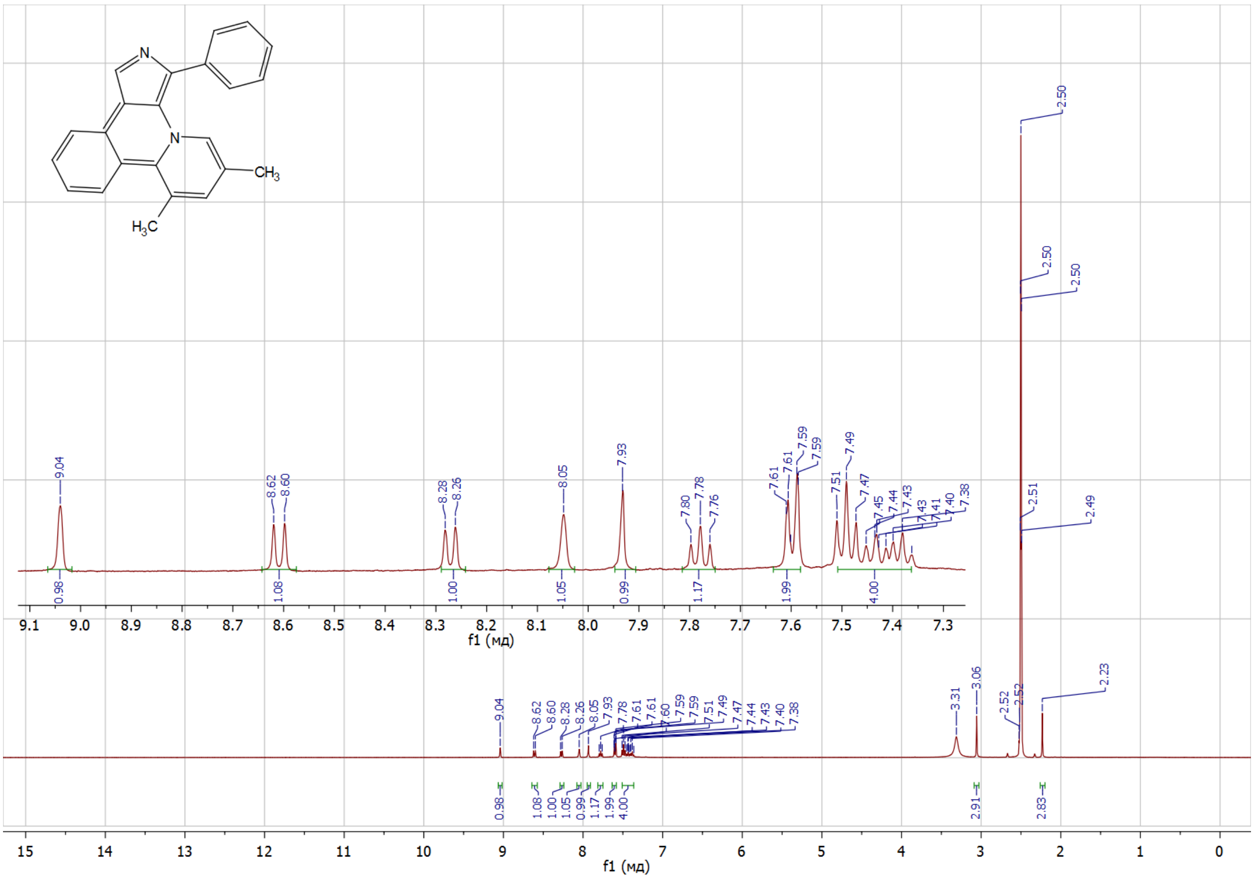

^{13}C{1H} NMR (101 MHz, DMSO-d_6) of 6,8-dimethyl-3-phenylpyrido[2,1-*a*]pyrrolo[3,4-*c*]isoquinoline (**6s**)

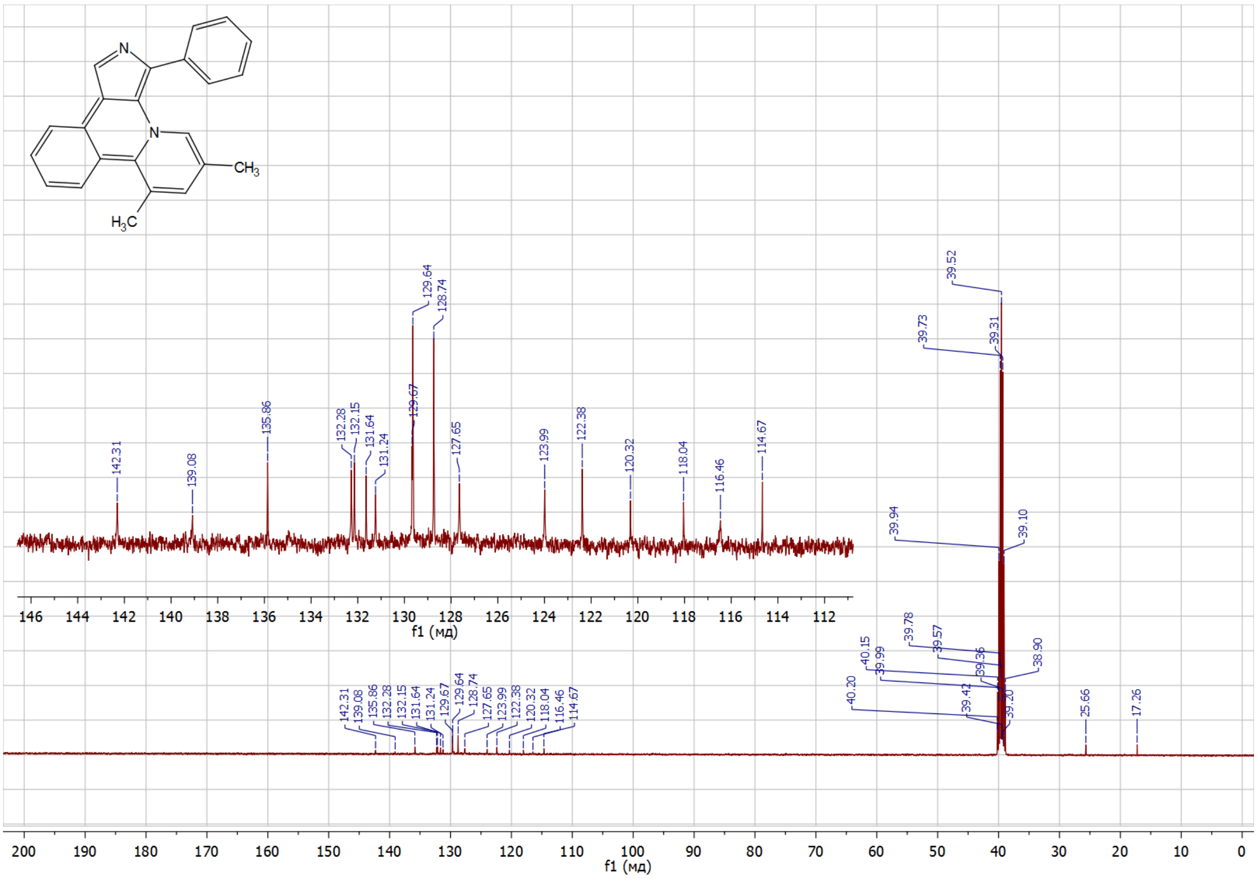

^{13}C DEPT135 NMR (101 MHz, DMSO-d_6) of 6,8-dimethyl-3-phenylpyrido[2,1-*a*]pyrrolo[3,4-*c*]isoquinoline (**6s**)

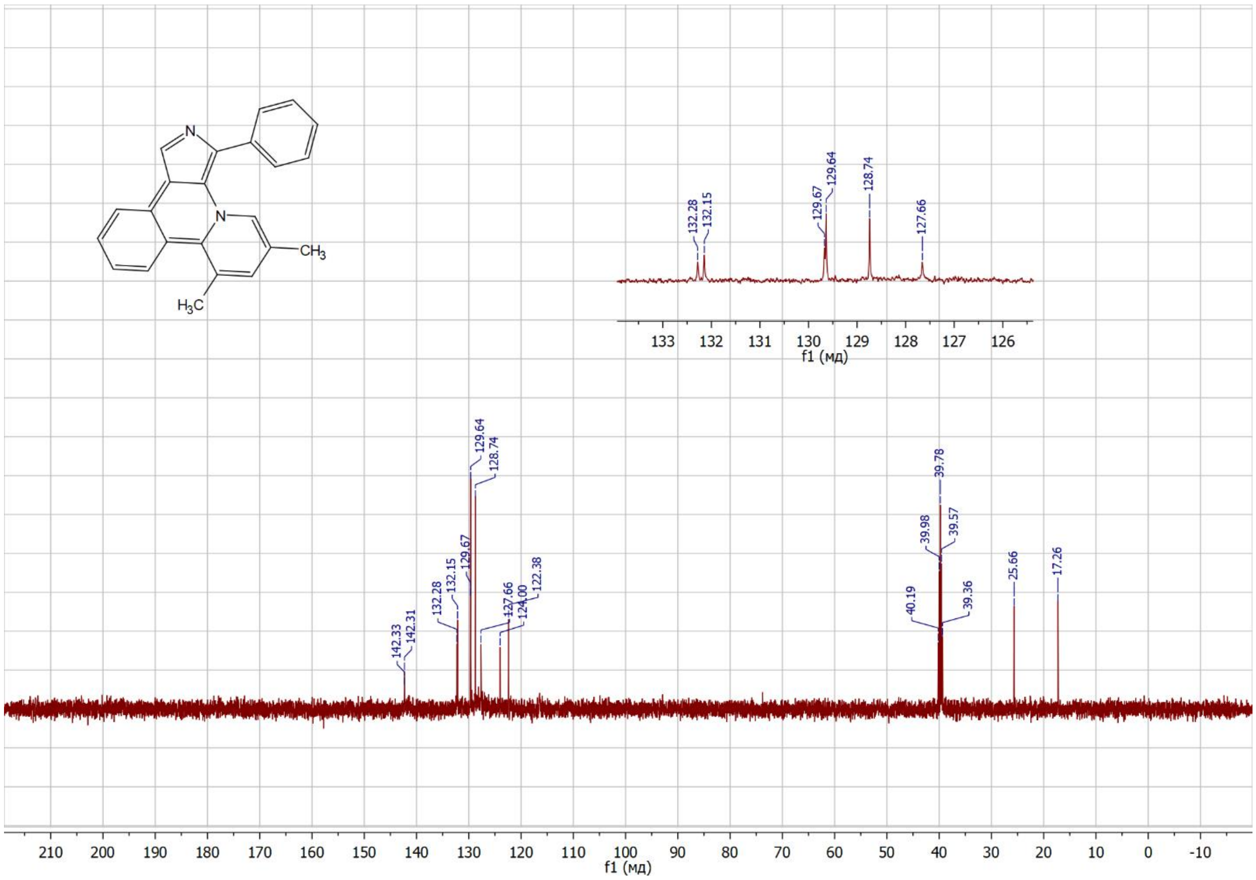

^{1}H NMR (400 MHz, DMSO-d_6) of 1-(4-(2-bromophenyl)-5-(*tert*-butoxycarbonyl)-2-phenyl-1*H*-pyrrol-3-yl)pyridin-1-ium bromide (**7**)

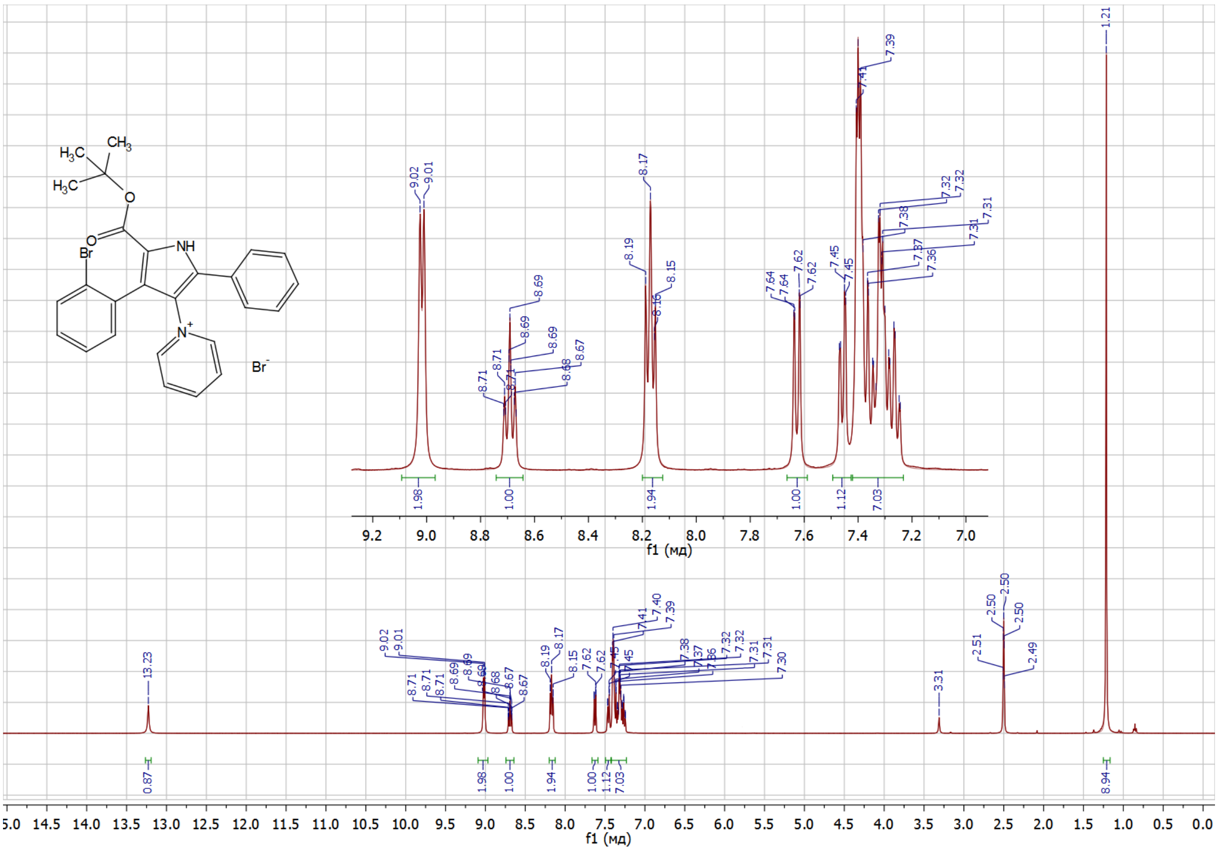

^{13}C{1H} NMR (101 MHz, DMSO-d_6) of 1-(4-(2-bromophenyl)-5-(*tert*-butoxycarbonyl)-2-phenyl-1*H*-pyrrol-3-yl)pyridin-1-ium bromide (**7**)

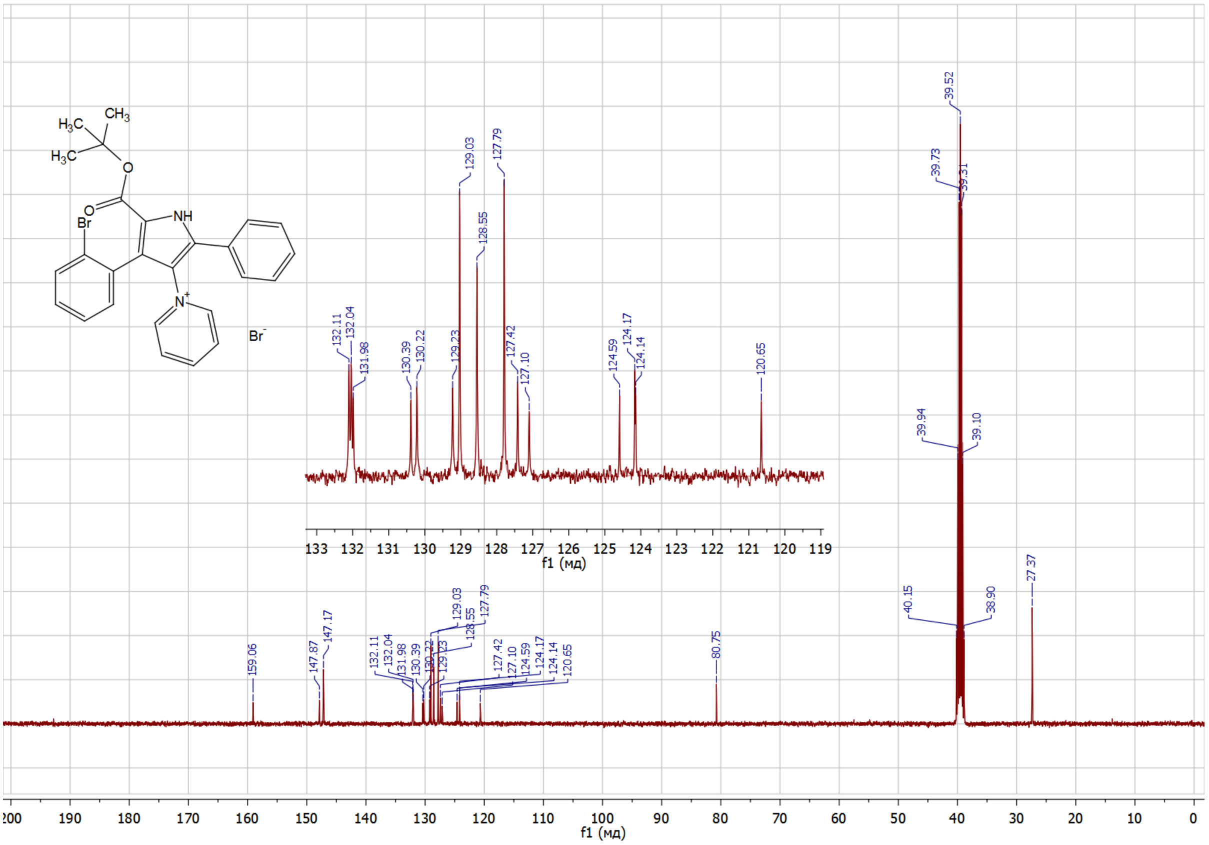

^{13}C DEPT135 NMR (101 MHz, DMSO-d_6) of 1-(4-(2-bromophenyl)-5-(*tert*-butoxycarbonyl)-2-phenyl-1*H*-pyrrol-3-yl)pyridin-1-ium bromide (**7**)

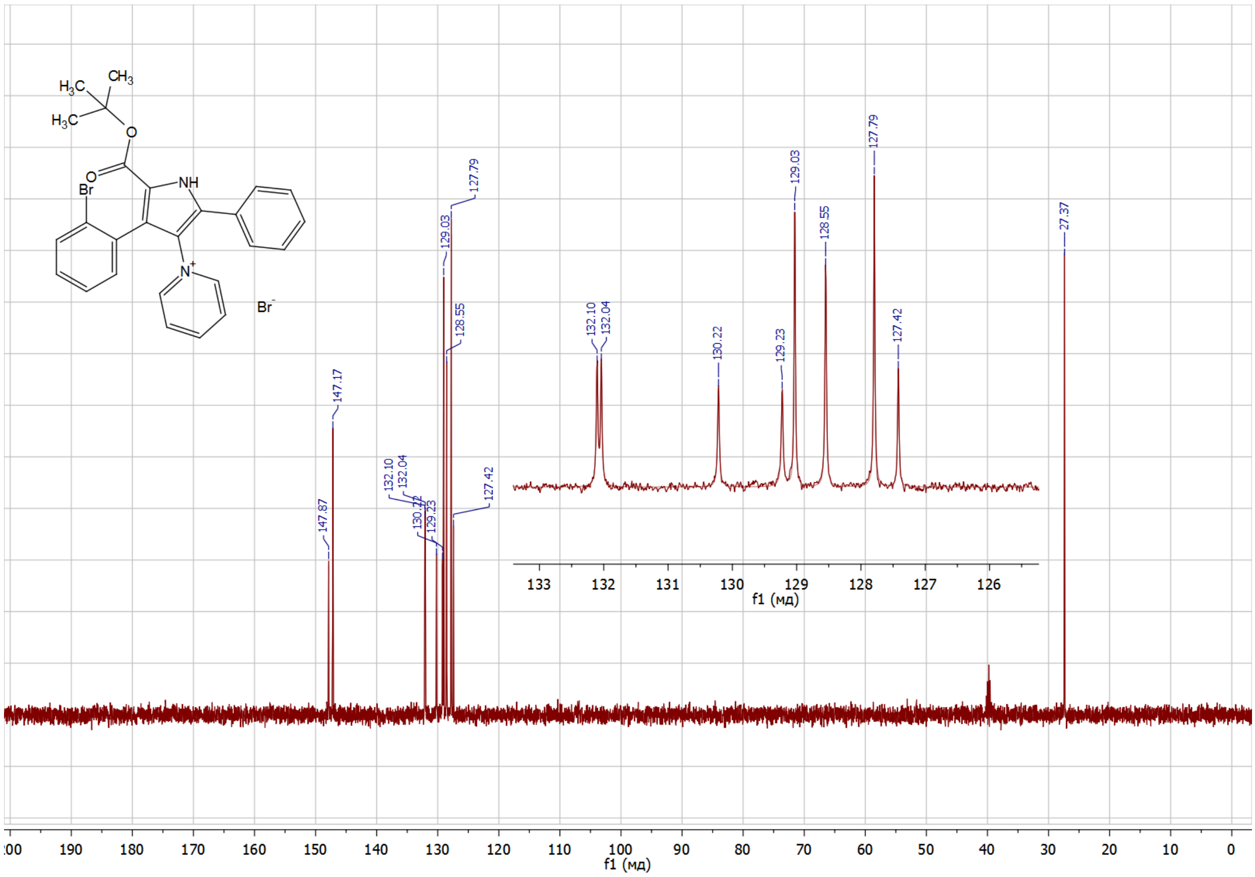

^{1}H NMR (400 MHz, DMSO-d_6) of 1-(*tert*-butoxycarbonyl)-3-phenyl-2*H*-pyrido[2,1-*a*]pyrrolo[3,4-*c*]isoquinolin-4-ium bromide (**8**)

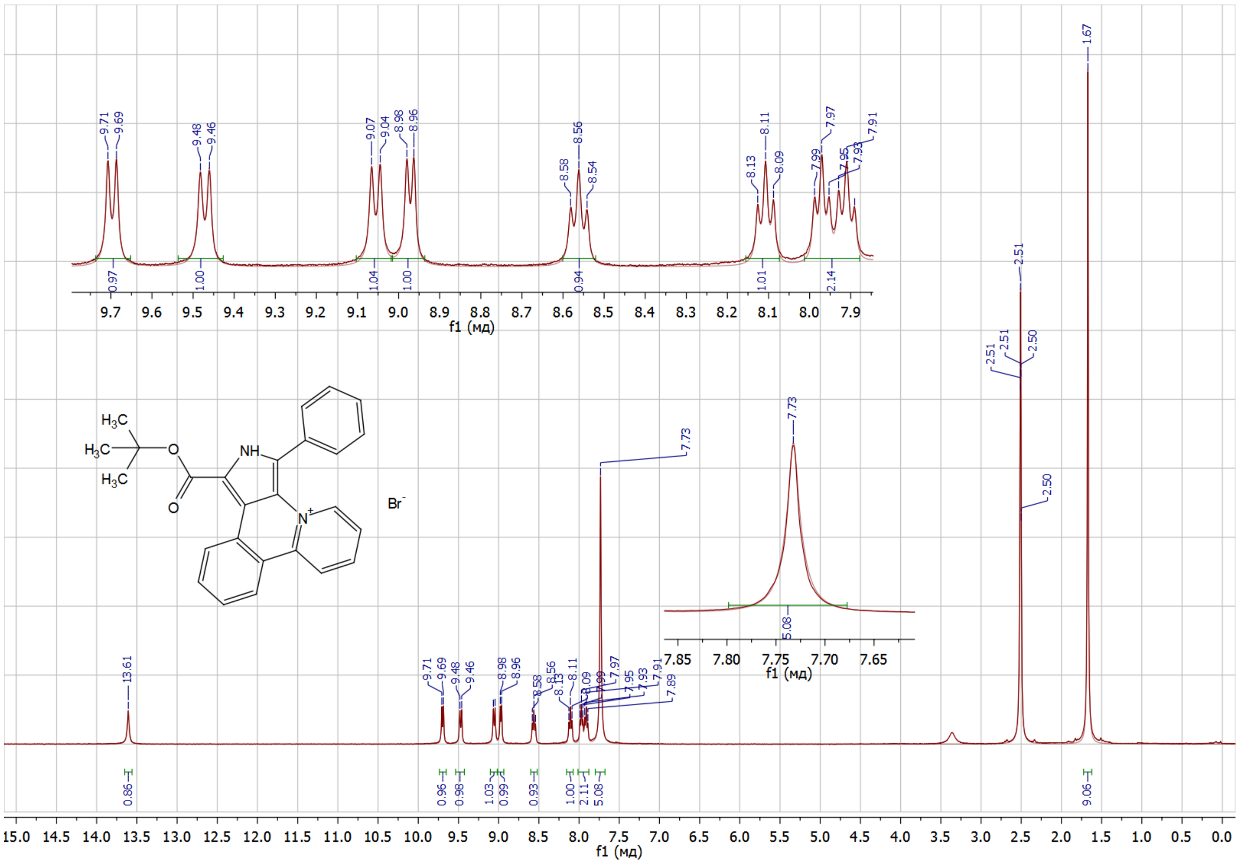

^{13}C{1H} NMR (101 MHz, DMSO-d_6) of 1-(*tert*-butoxycarbonyl)-3-phenyl-2*H*-pyrido[2,1-*a*]pyrrolo[3,4-*c*]isoquinolin-4-ium bromide (**8**)

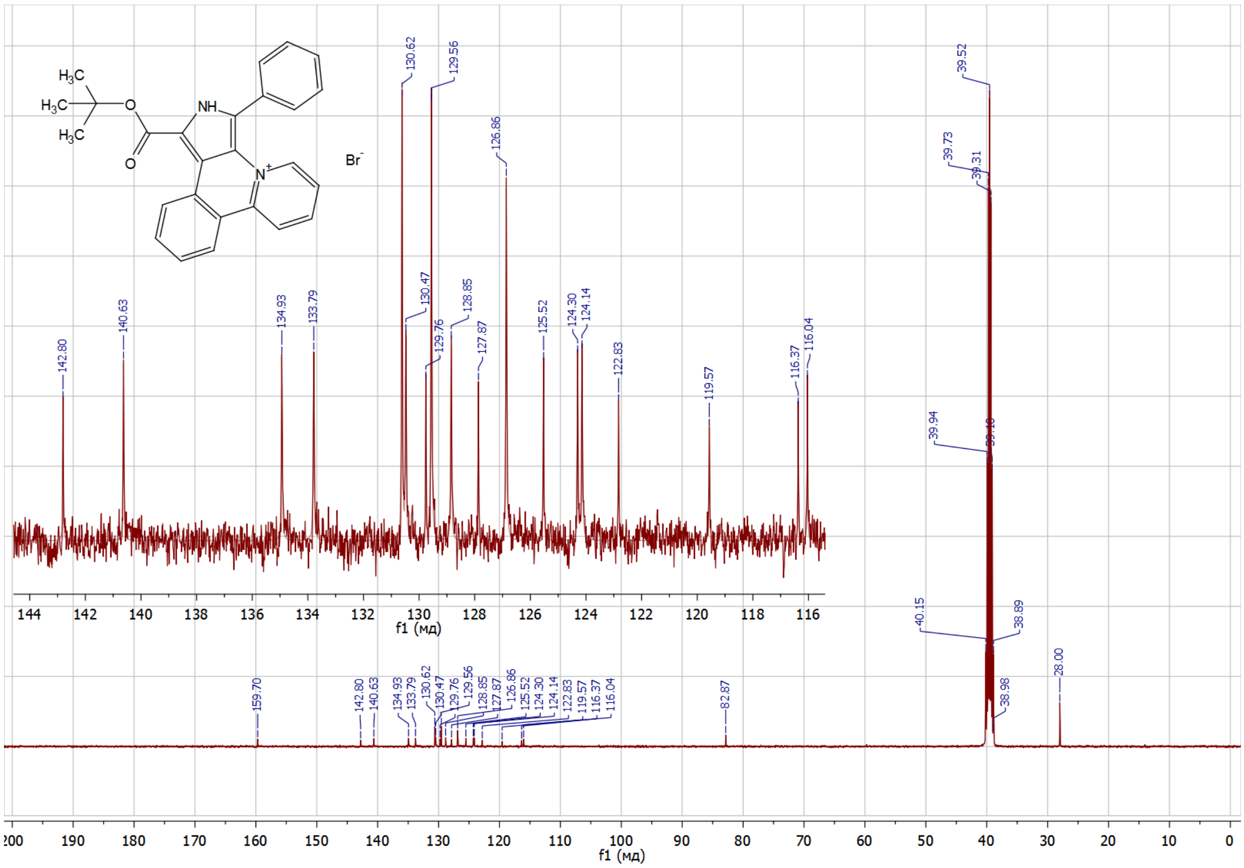

^{13}C DEPT135 NMR (101 MHz, DMSO-d_6) of 1-(*tert*-butoxycarbonyl)-3-phenyl-2*H*-pyrido[2,1-*a*]pyrrolo[3,4-*c*]isoquinolin-4-ium bromide (**8**)

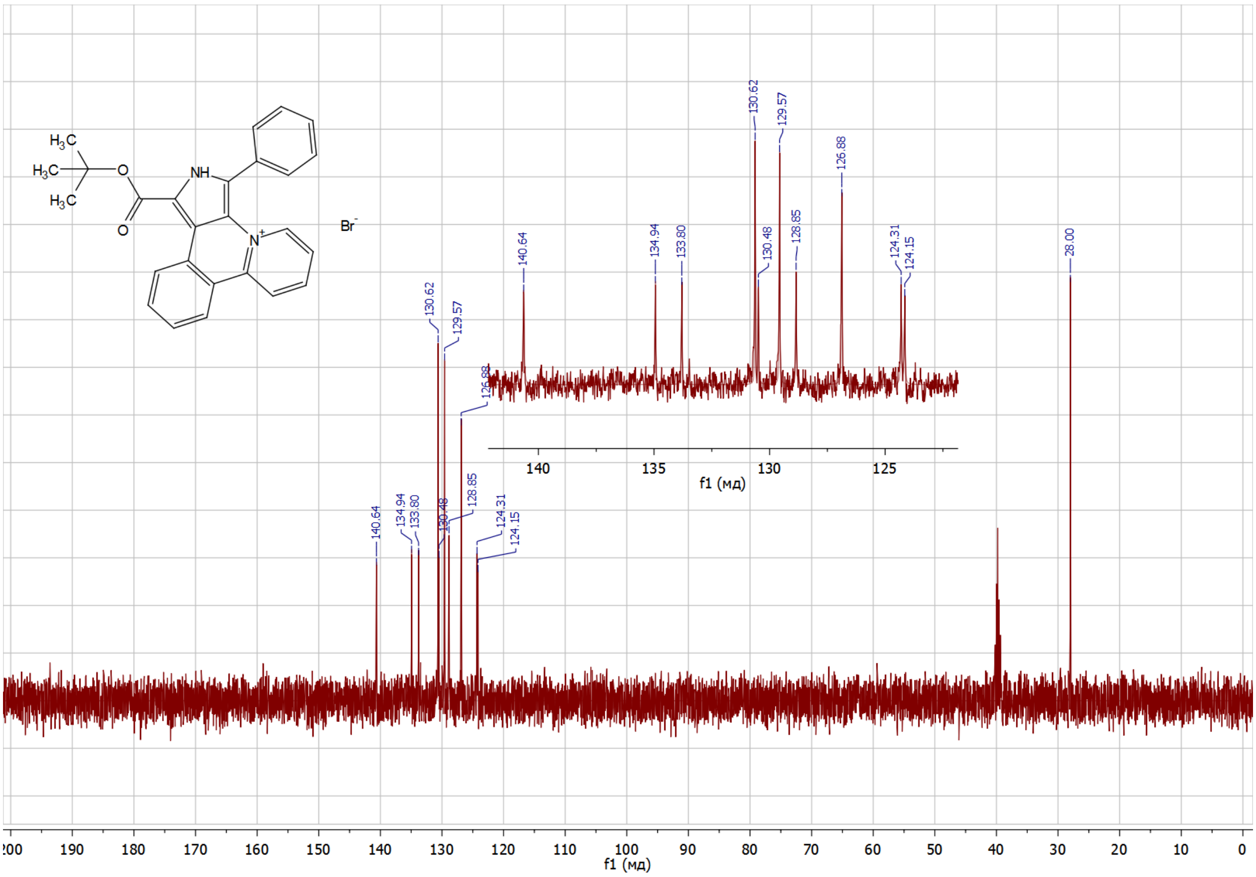

^{1}H NMR (400 MHz, DMSO-*d*$_6$) of 1-(2-(2-bromophenyl)-4-(2-iodophenyl)-1*H*-pyrrol-3-yl)pyridin-1-ium bromide (**9**)

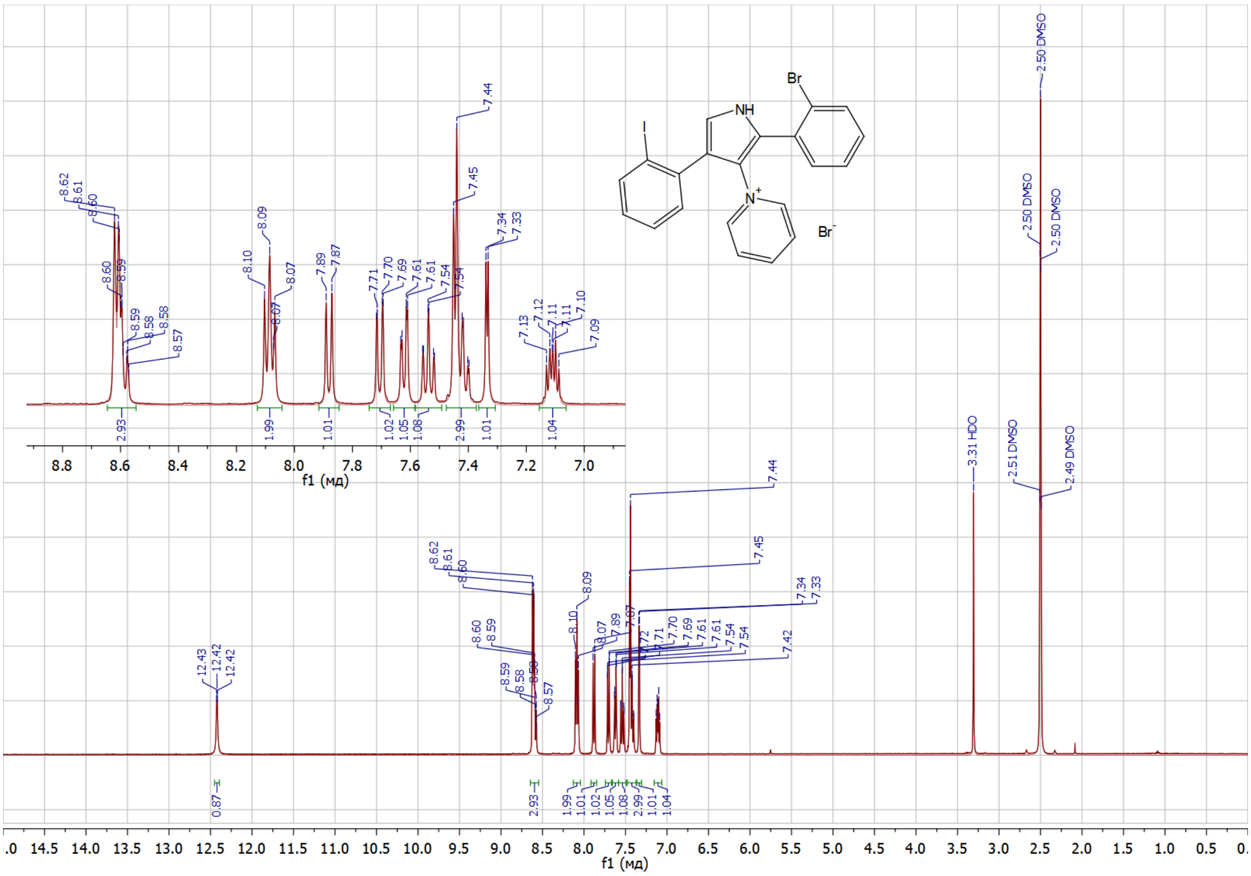

$^{13}C\{1H\}$ NMR (101 MHz, DMSO-*d*$_6$) of 1-(2-(2-bromophenyl)-4-(2-iodophenyl)-1*H*-pyrrol-3-yl)pyridin-1-ium bromide (**9**)

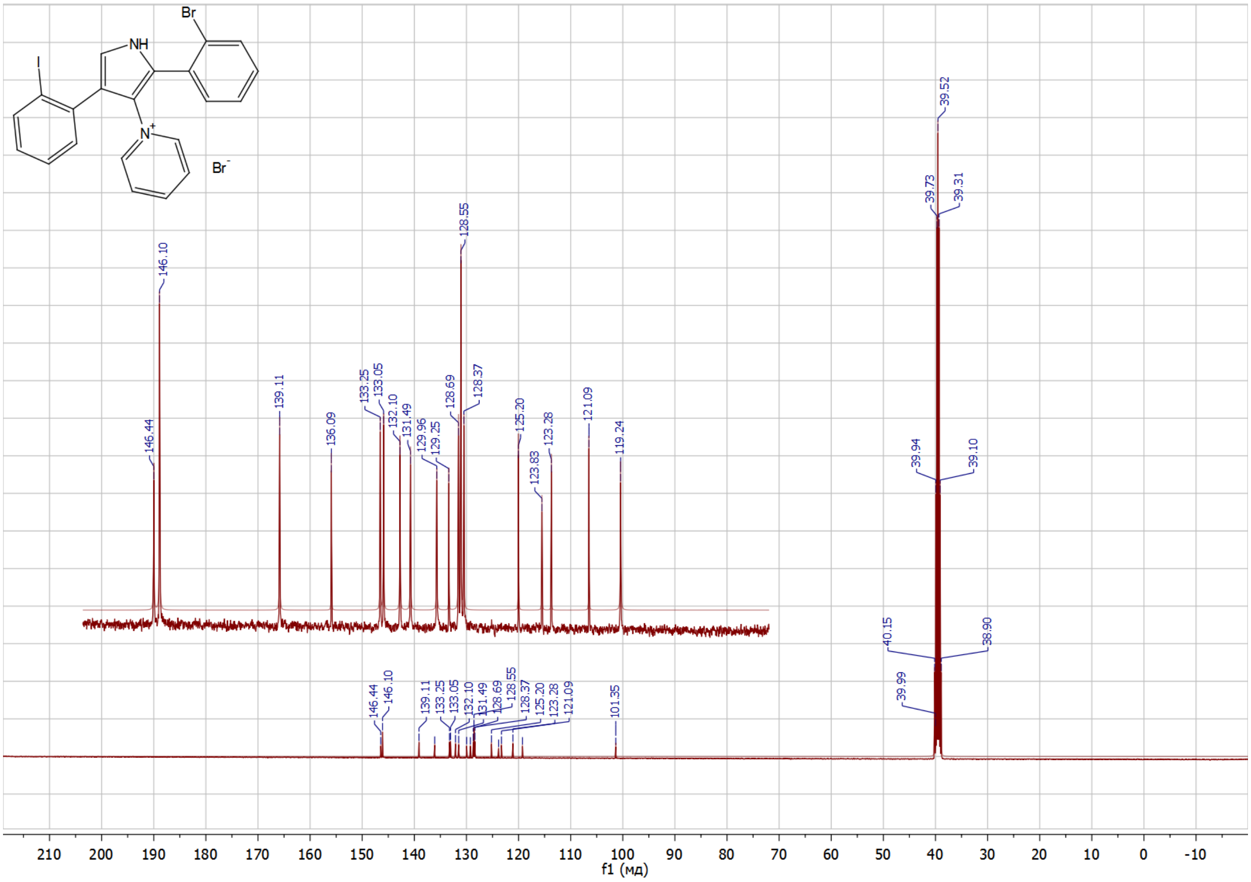

^{13}C DEPT135 NMR (101 MHz, DMSO-d_6) of 1-(2-(2-bromophenyl)-4-(2-iodophenyl)-1*H*-pyrrol-3-yl)pyridin-1-ium bromide (**9**)

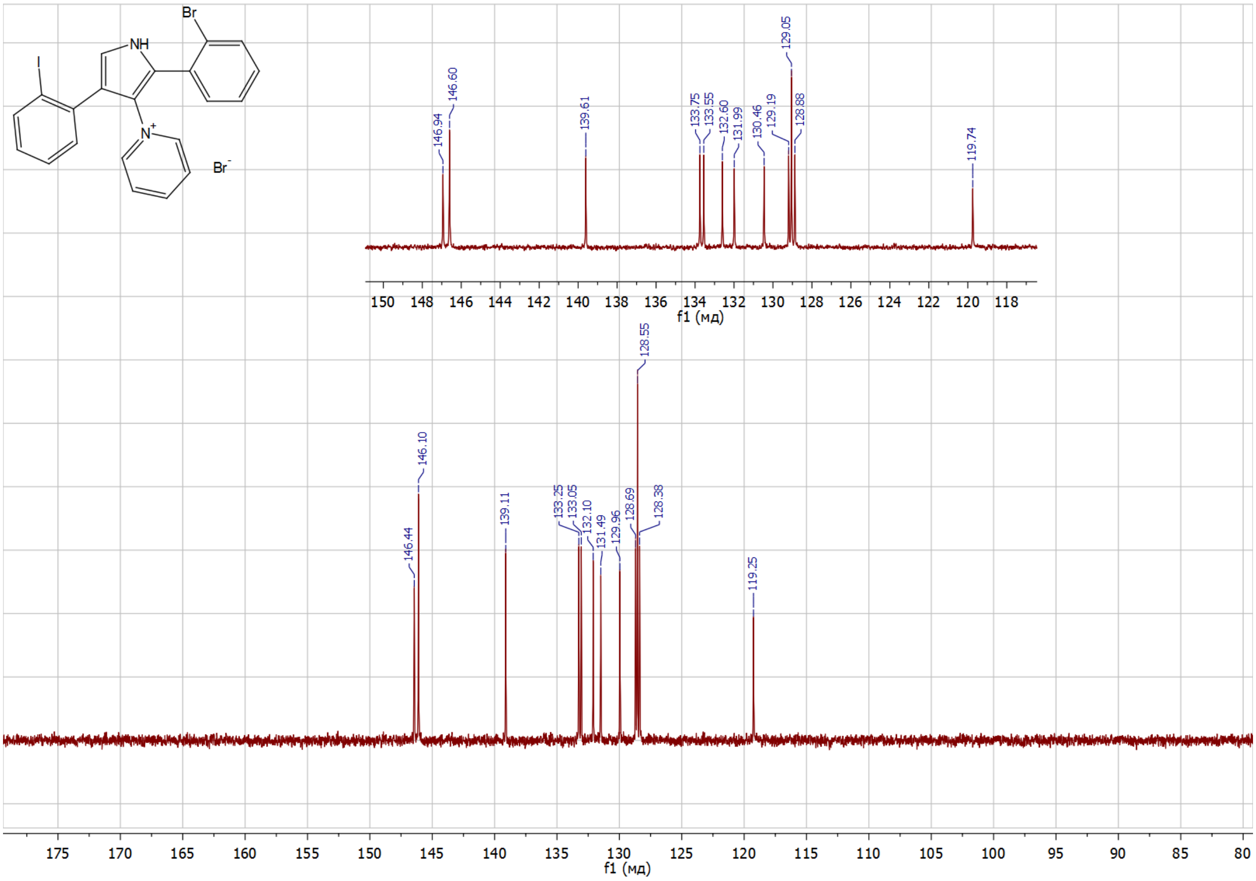

^{1}H NMR (400 MHz, DMSO-d_6) of 3-(2-bromophenyl)-2*H*-pyrido[2,1-*a*]pyrrolo[3,4-*c*]isoquinolin-4-ium bromide (**10**)

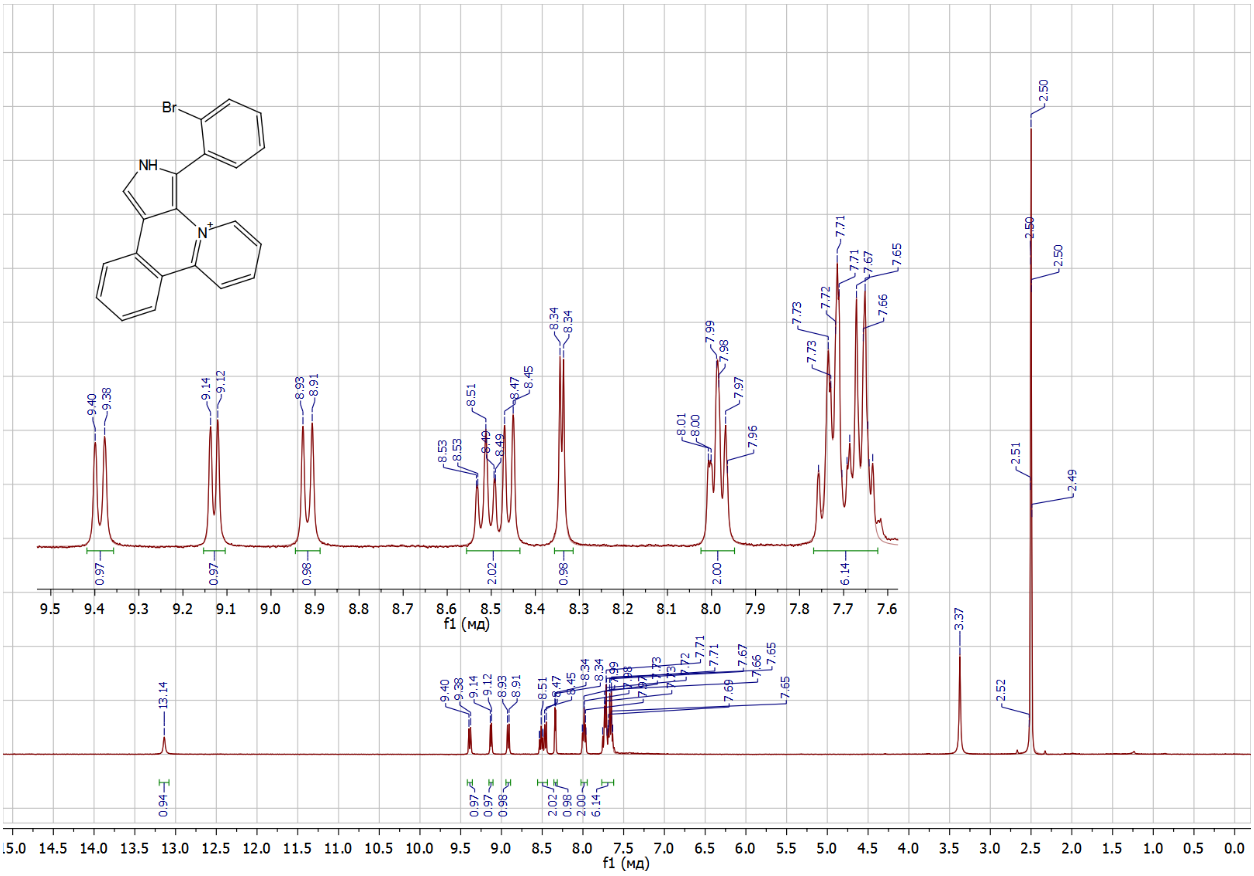

^{13}C{1H} NMR (101 MHz, DMSO-d_6) of 3-(2-bromophenyl)-2*H*-pyrido[2,1-*a*]pyrrolo[3,4-*c*]isoquinolin-4-ium bromide (**10**)

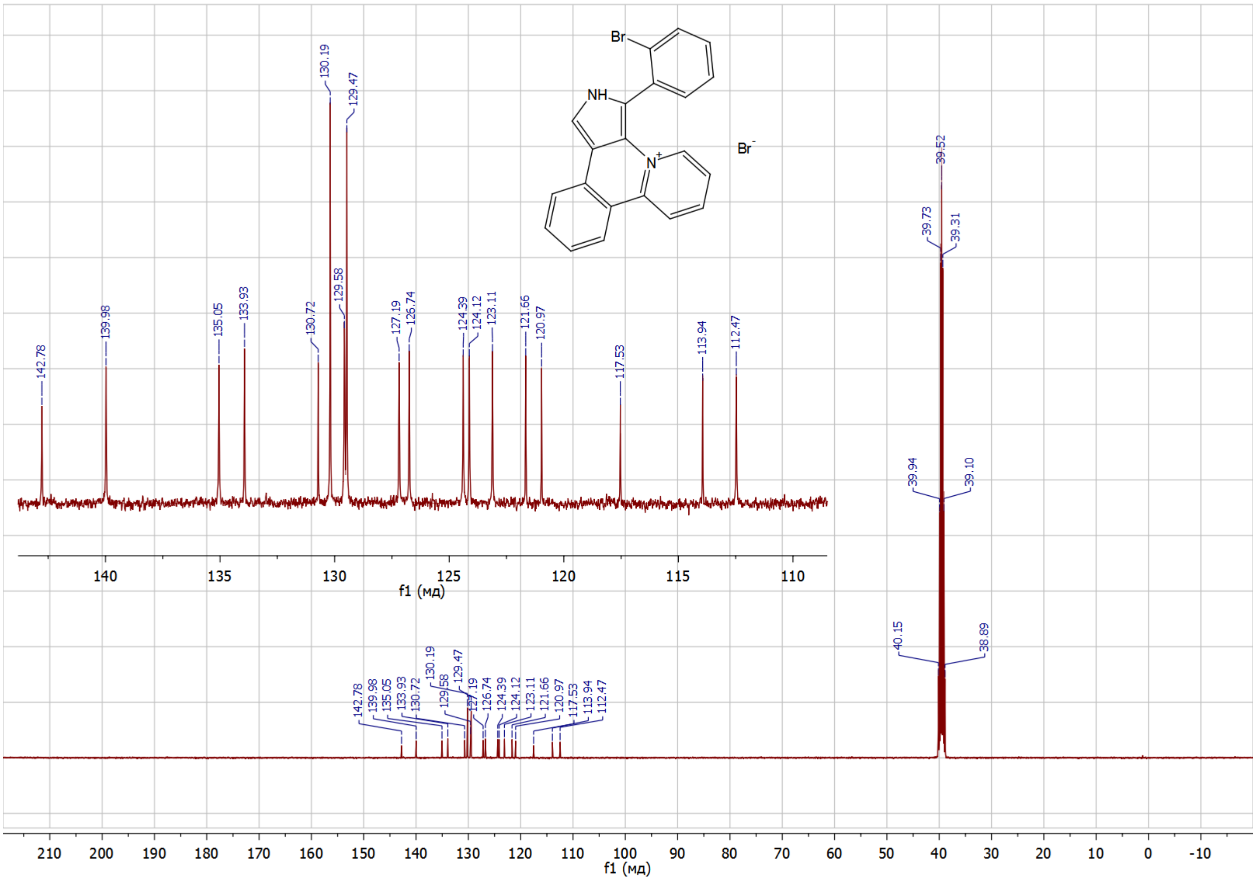

^{13}C DEPT135 NMR (101 MHz, DMSO-d_6) of 3-(2-bromophenyl)-2*H*-pyrido[2,1-*a*]pyrrolo[3,4-*c*]isoquinolin-4-ium bromide (**10**)

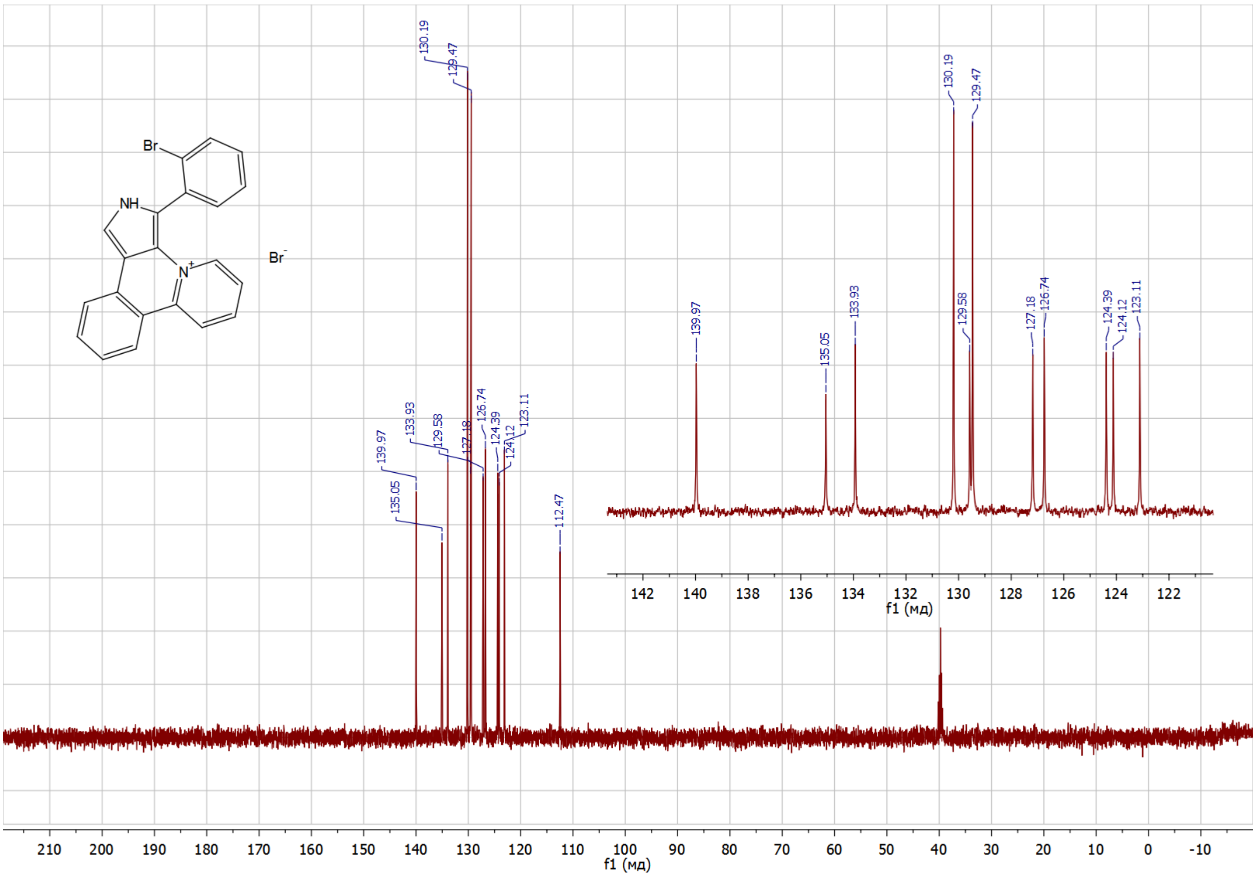

^{1}H NMR (400 MHz, DMSO-d_6) of 3-(2-bromophenyl)pyrido[2,1-*a*]pyrrolo[3,4-*c*]isoquinoline (**11**)

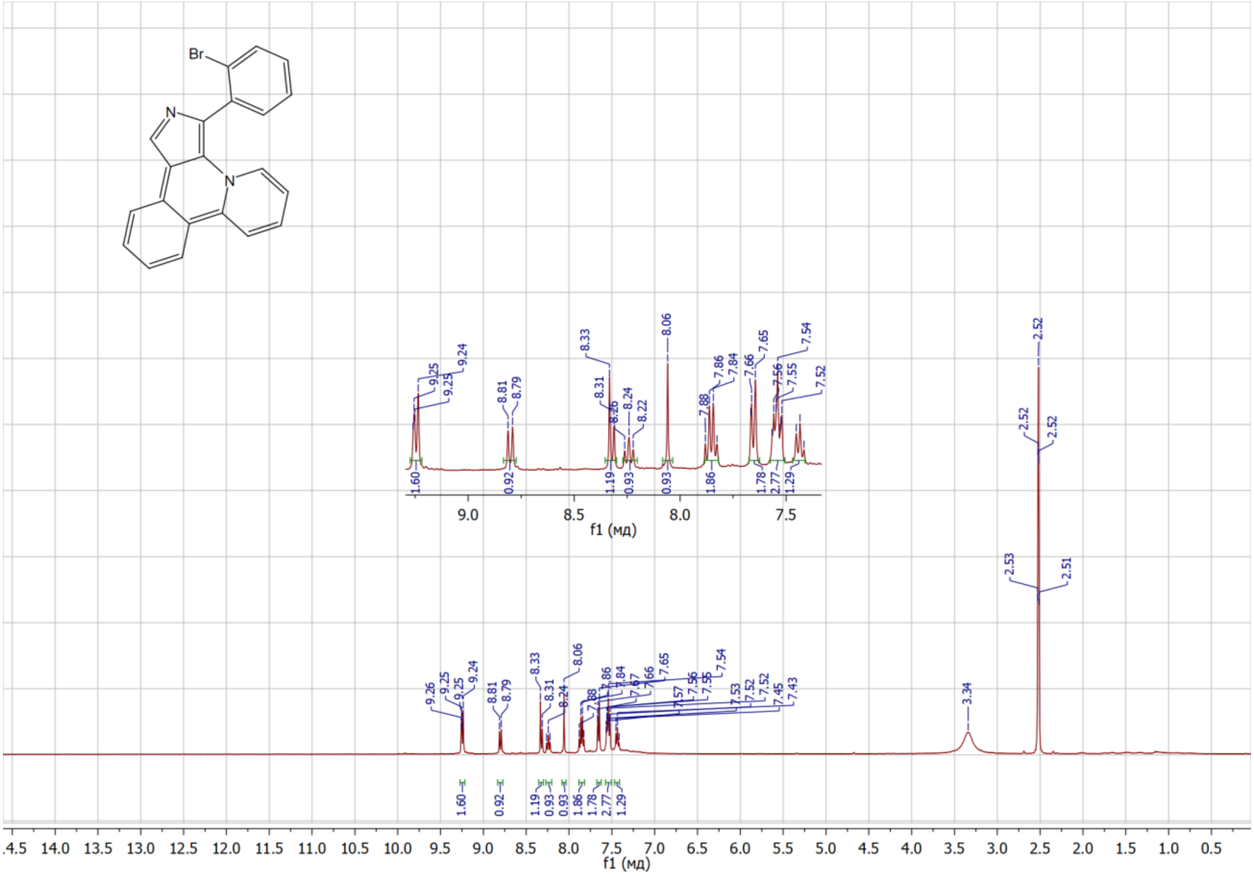

$^{13}C\{1H\}$ NMR (101 MHz, DMSO-d_6) of 3-(2-bromophenyl)pyrido[2,1-*a*]pyrrolo[3,4-*c*]isoquinoline (**11**)

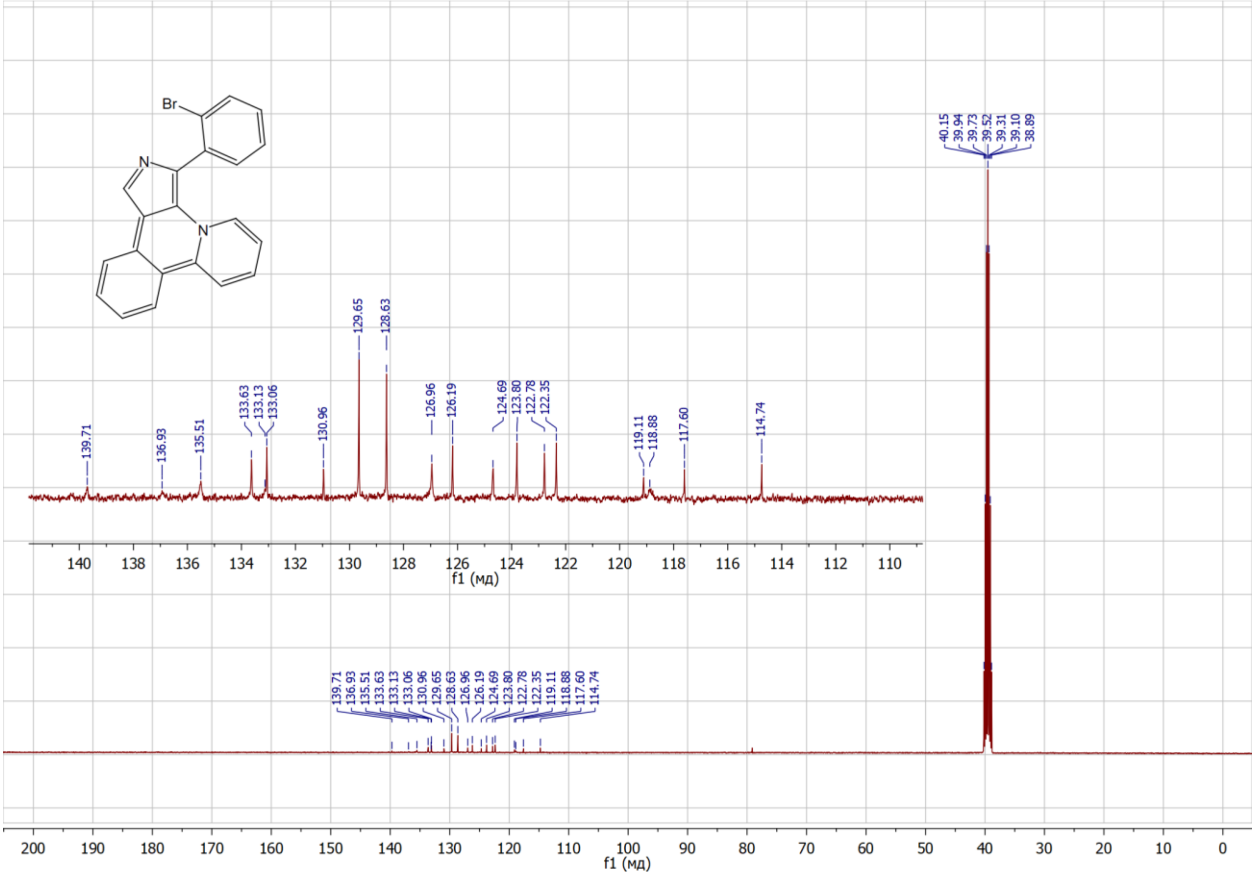

^{13}C DEPT135 NMR (101 MHz, DMSO-d_6) of 3-(2-bromophenyl)pyrido[2,1-*a*]pyrrolo[3,4-*c*]isoquinoline (**11**)

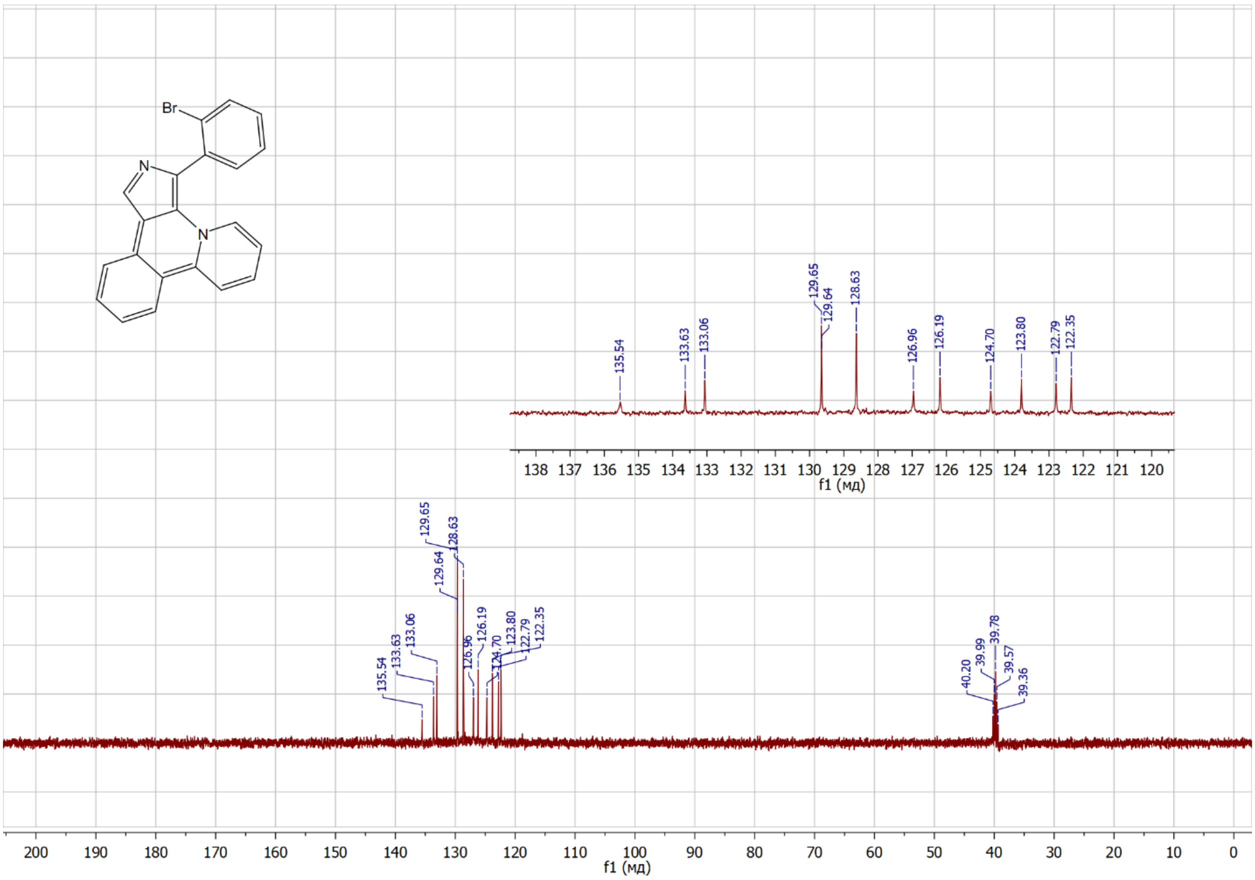

^{1}H NMR (400 MHz, DMSO-d_6) of 1-(2,4-bis(2-bromophenyl)-1*H*-pyrrol-3-yl)pyridin-1-ium bromide (**12**)

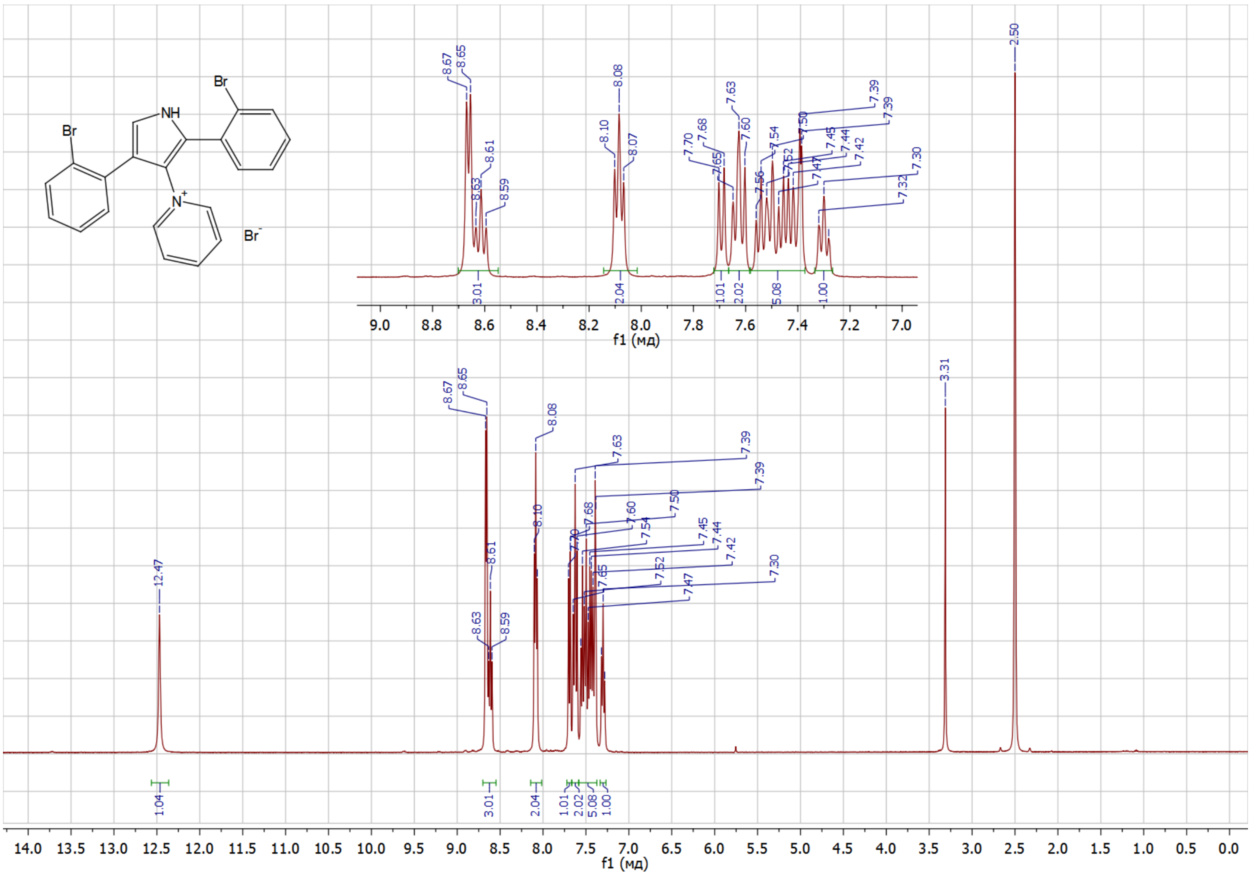

$^{13}C\{1H\}$ NMR (101 MHz, DMSO-d_6) of 1-(2,4-bis(2-bromophenyl)-1*H*-pyrrol-3-yl)pyridin-1-ium bromide (**12**)

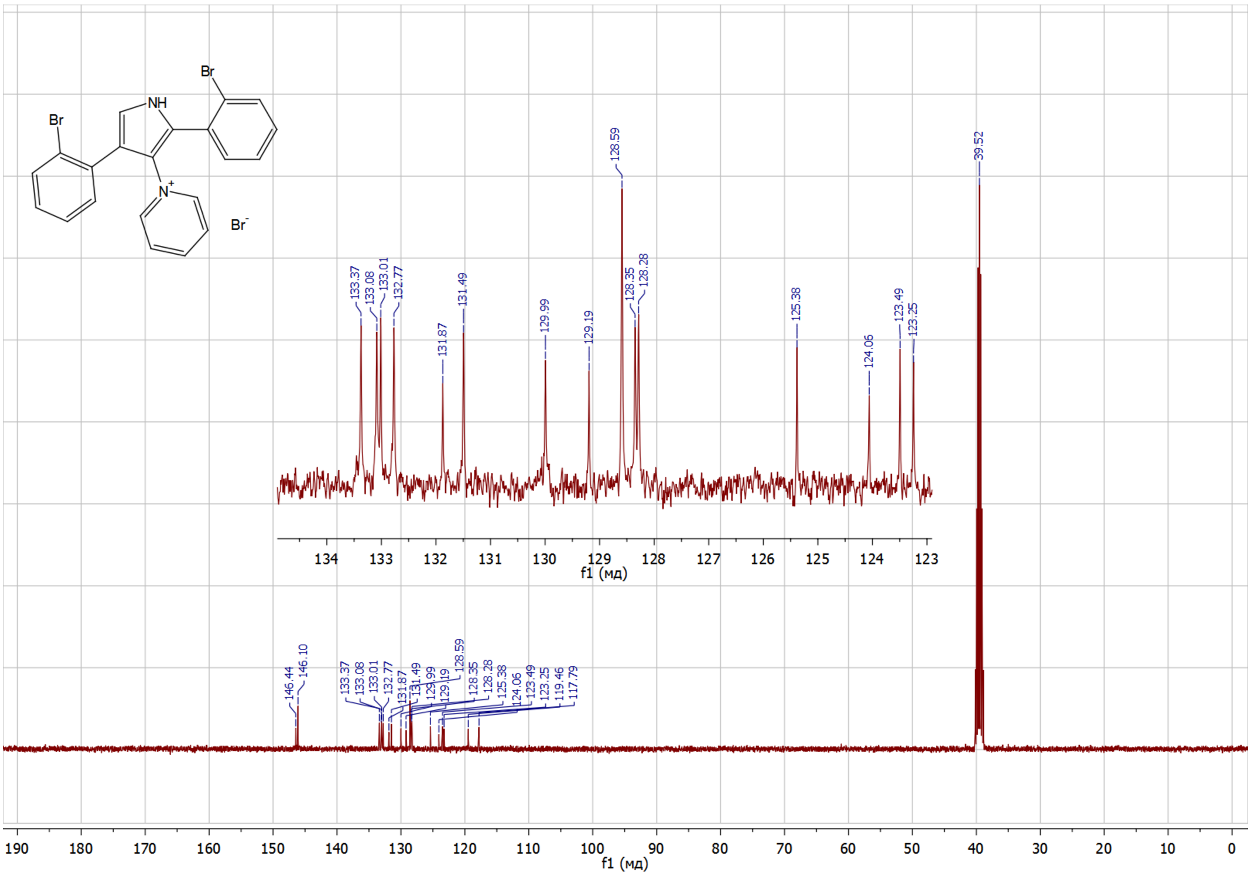

^{13}C DEPT135 NMR (101 MHz, DMSO-d_6) of 1-(2,4-bis(2-bromophenyl)-1*H*-pyrrol-3-yl)pyridin-1-ium bromide (**12**)

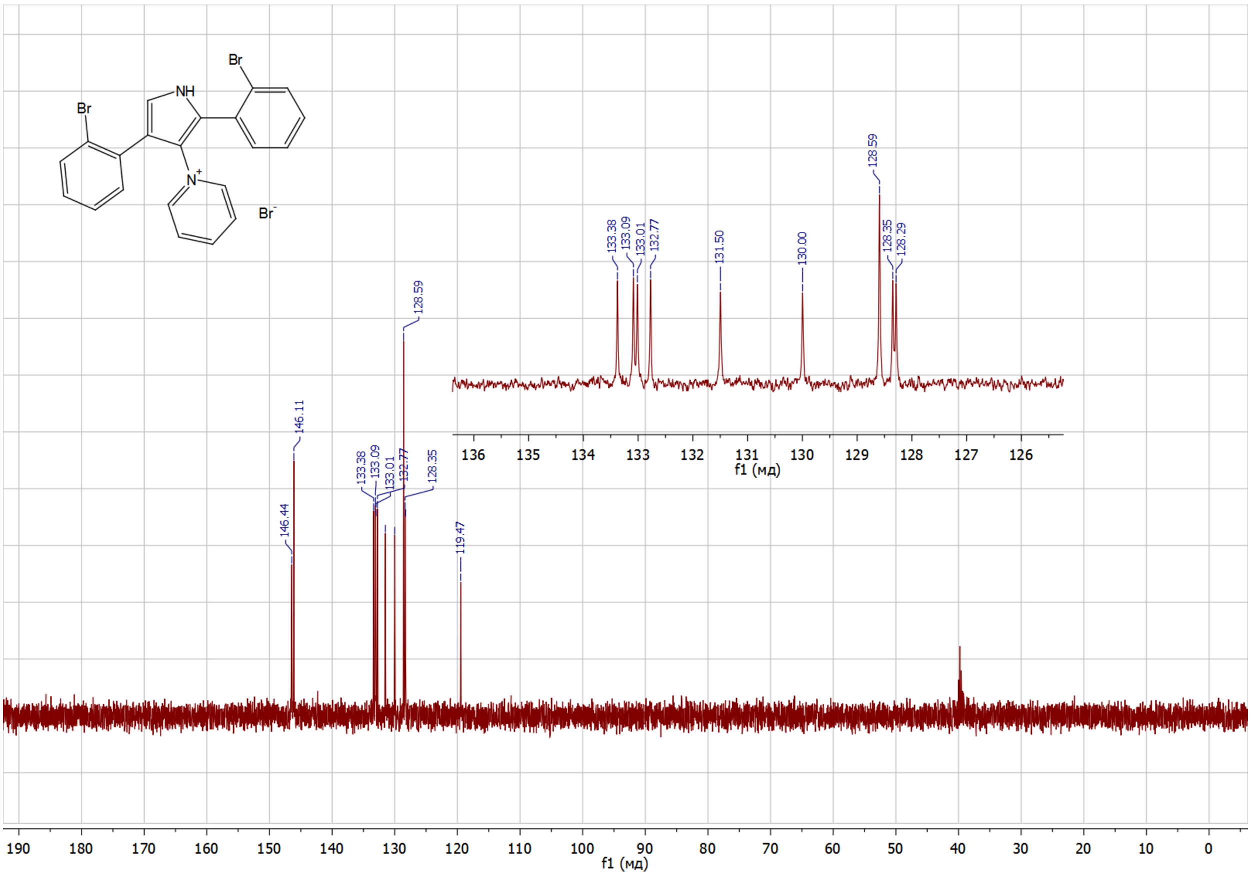

^{1}H NMR (500 MHz, DMSO-d_6) of dibenzo[b,g]pyrido[2,1,6-de]pyrrolo[2,3,4-ij]quinolizin-1-ium bromide (**13**)

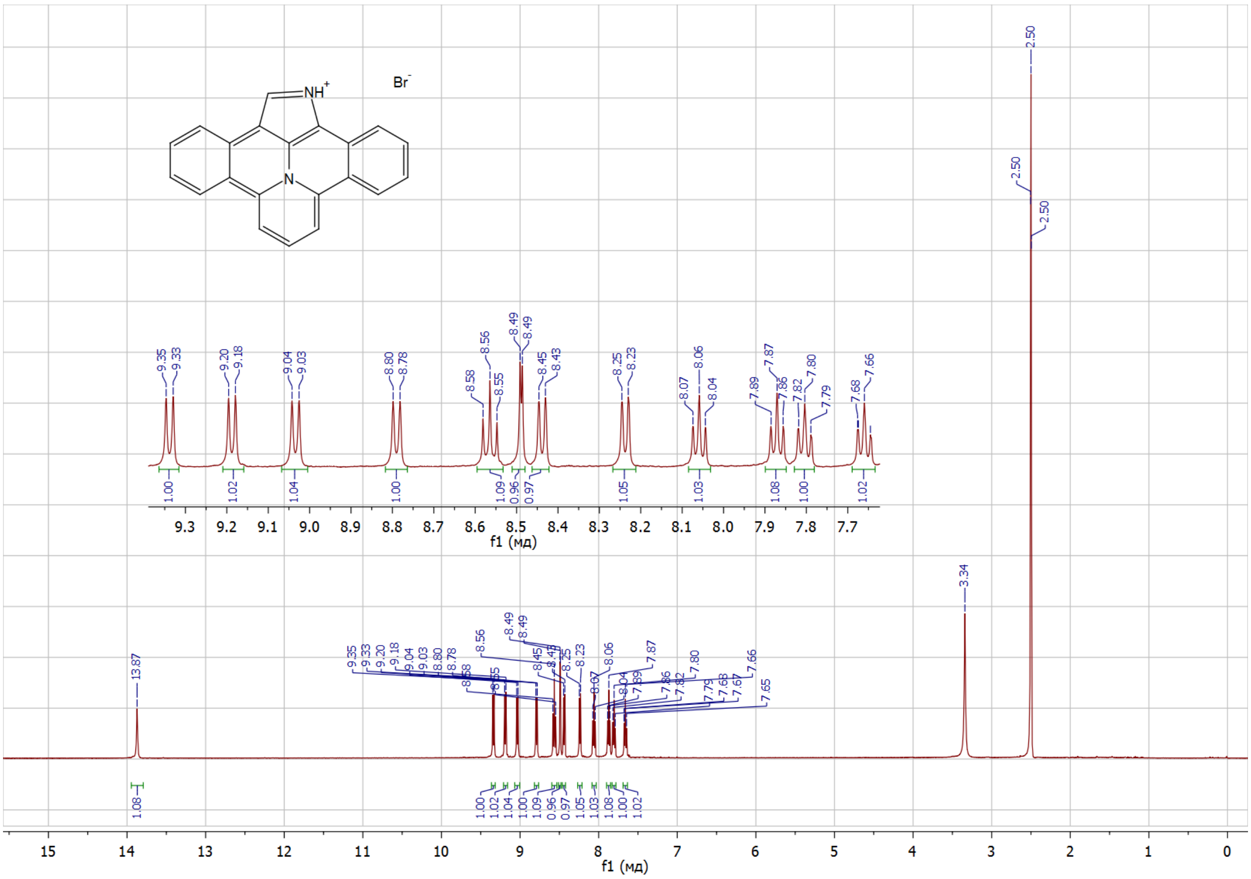

$^{13}C\{1H\}$ NMR (126 MHz, DMSO-d_6) of dibenzo[b,g]pyrido[2,1,6-de]pyrrolo[2,3,4-ij]quinolizin-1-ium bromide (**13**)

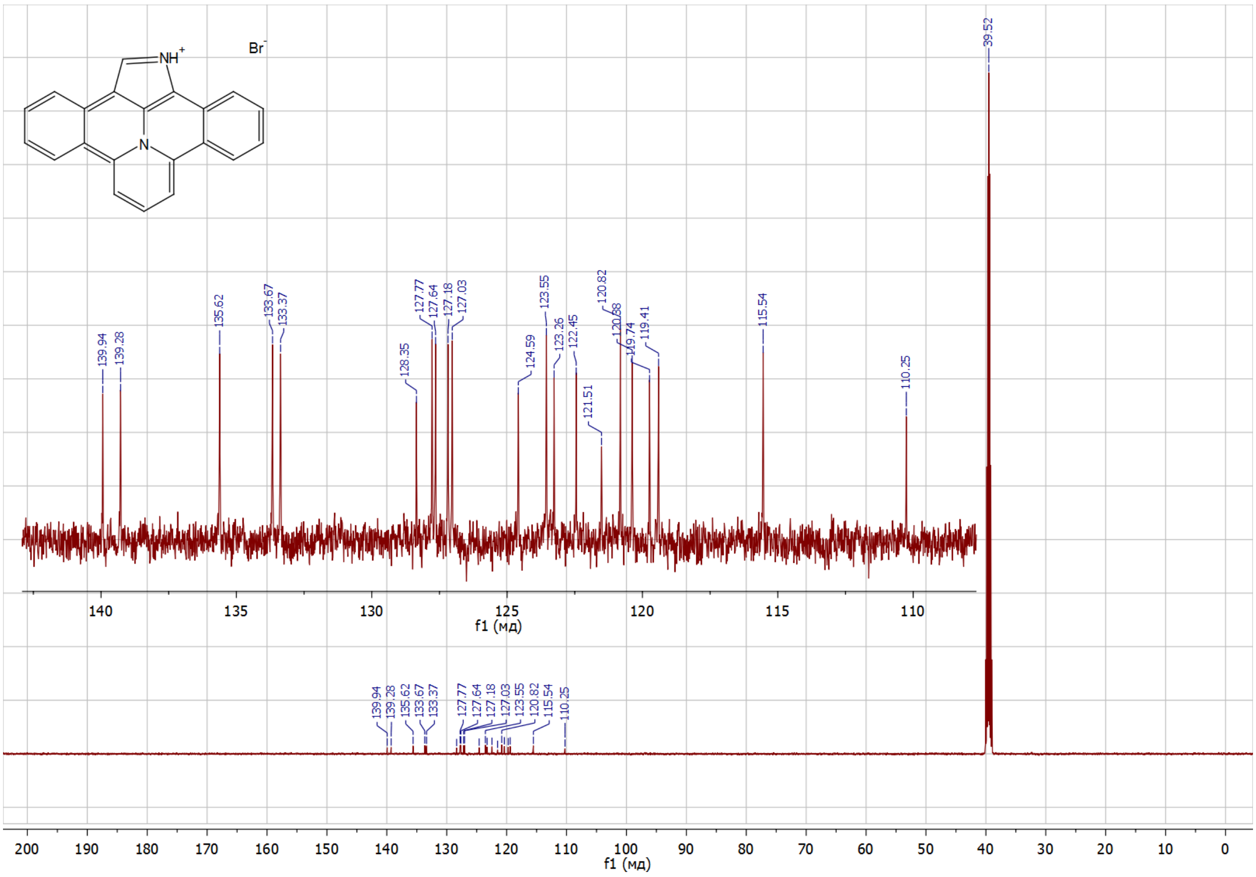

^{13}C DEPT135 NMR (126 MHz, DMSO-d_6) of dibenzo[b,g]pyrido[2,1,6-de]pyrrolo[2,3,4-ij]quinolizin-1-ium bromide (**13**)

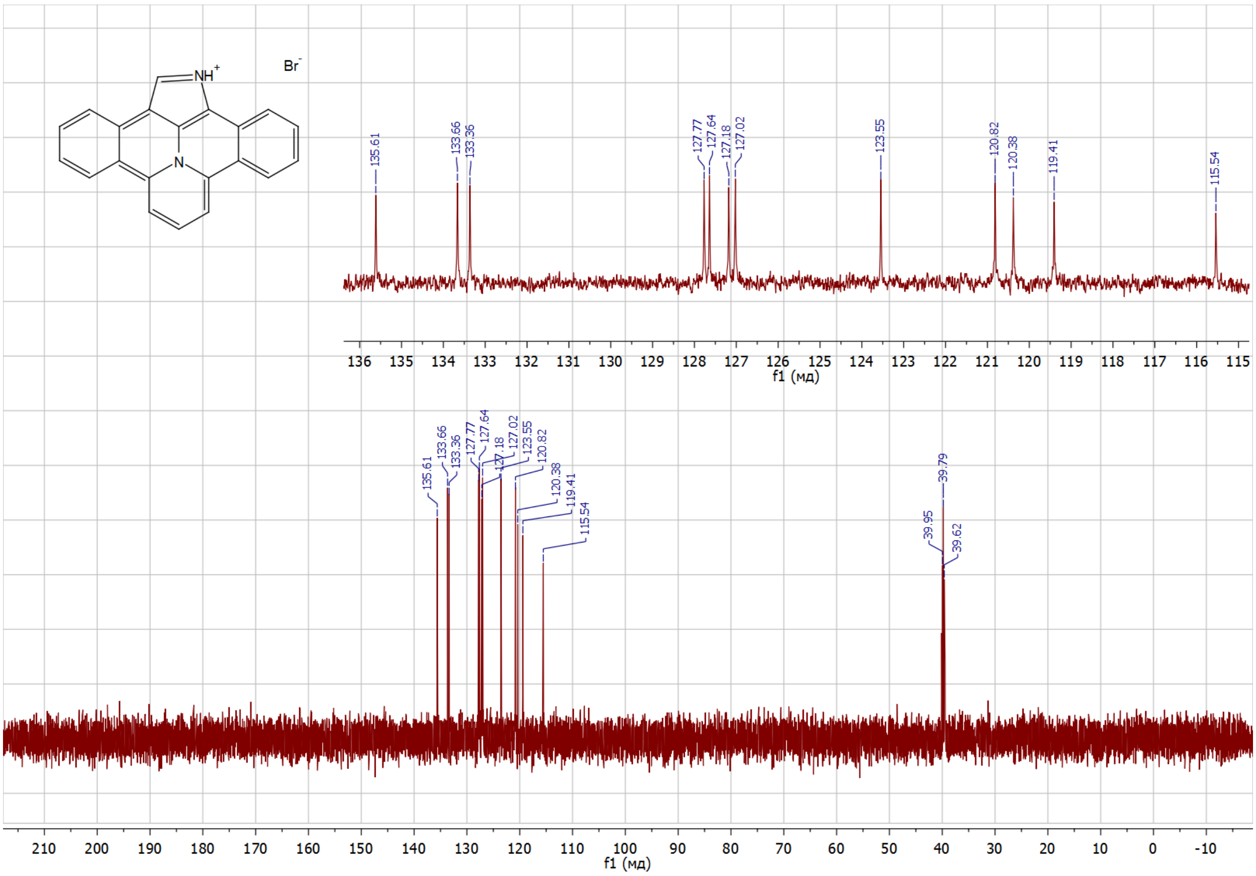

^{1}H NMR (400 MHz, DMSO-d_6) of dibenzo[b,g]pyrido[2,1,6-de]pyrrolo[2,3,4-ij]quinoline (**14**)

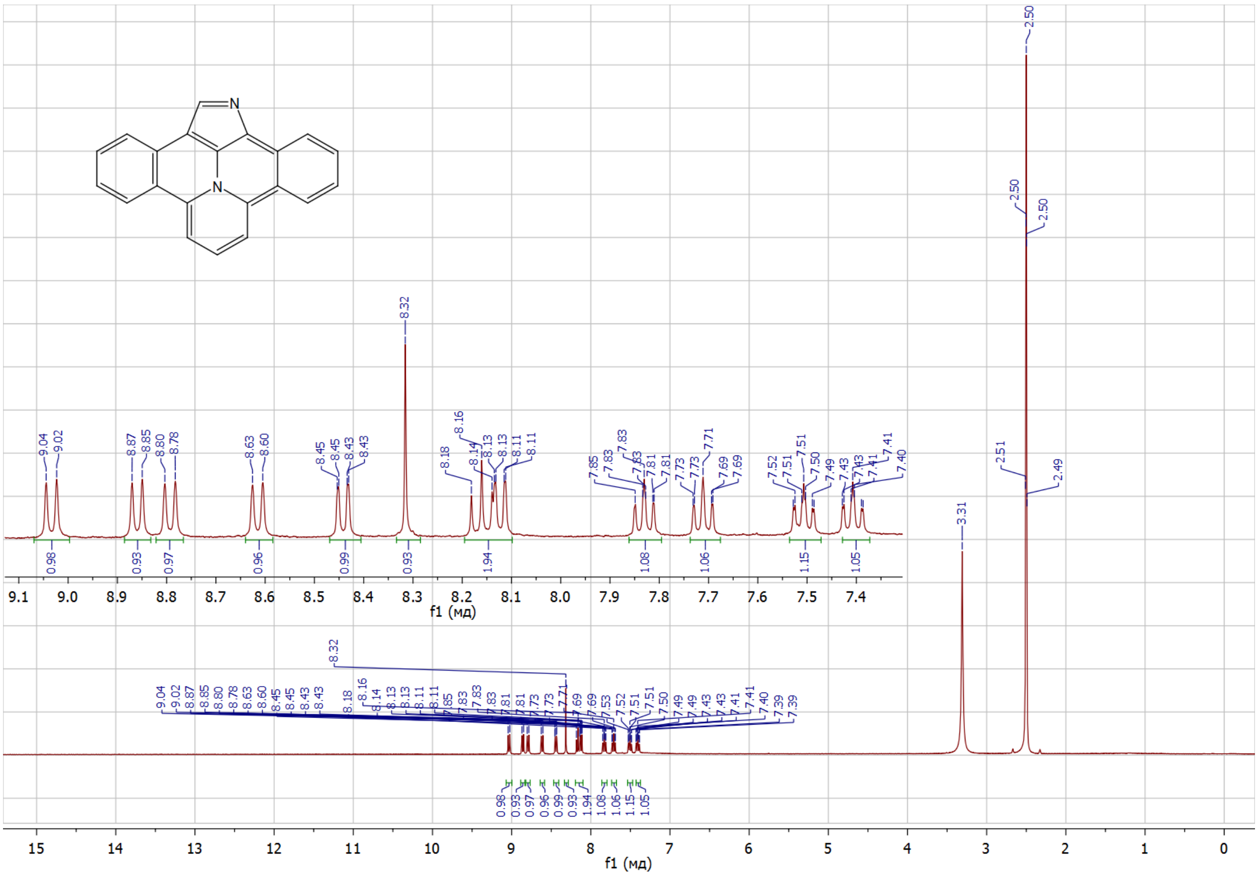

^{13}C{1H} NMR (101 MHz, DMSO-d_6) of dibenzo[b,g]pyrido[2,1,6-de]pyrrolo[2,3,4-ij]quinoline (**14**)

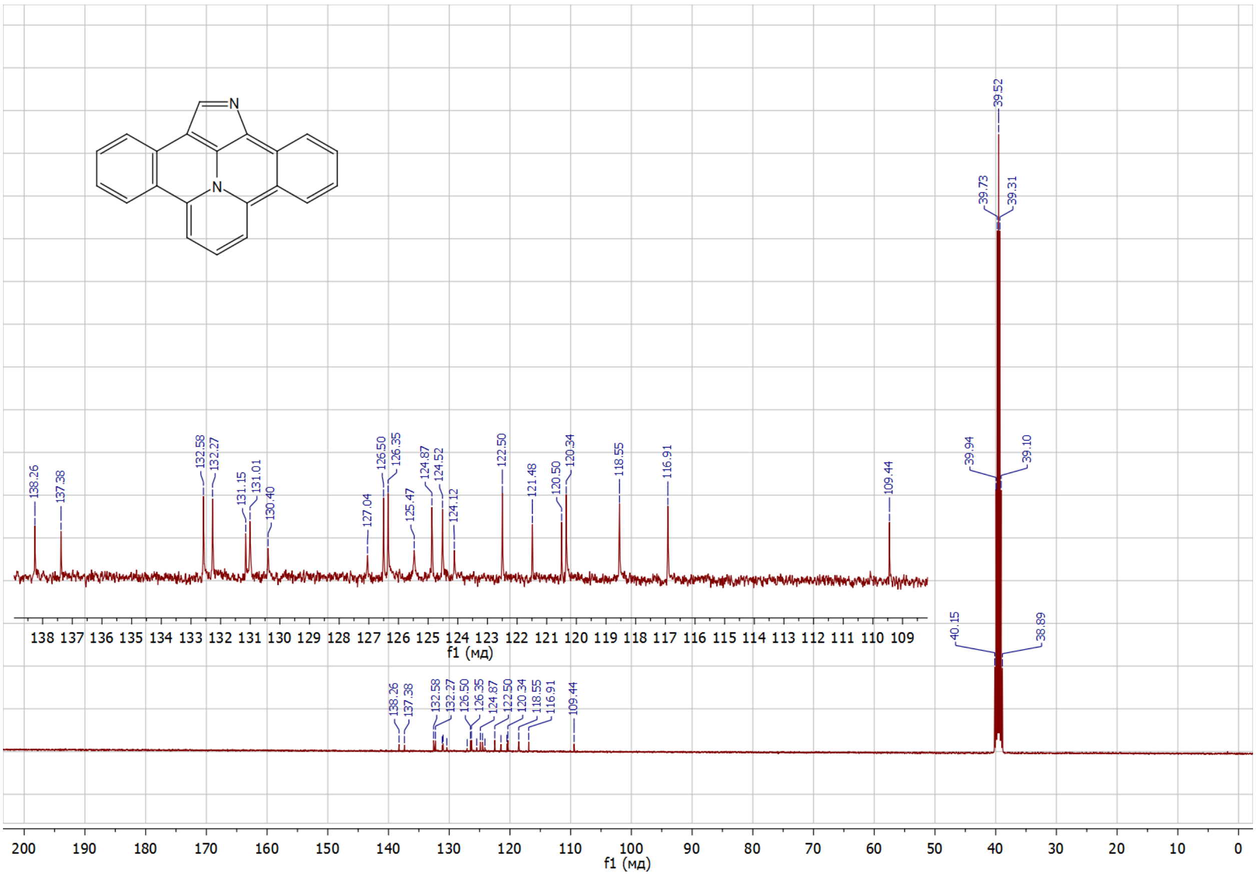

^{13}C DEPT135 NMR (101 MHz, DMSO-d_6) of dibenzo[b,g]pyrido[2,1,6-de]pyrrolo[2,3,4-ij]quinoline (**14**)

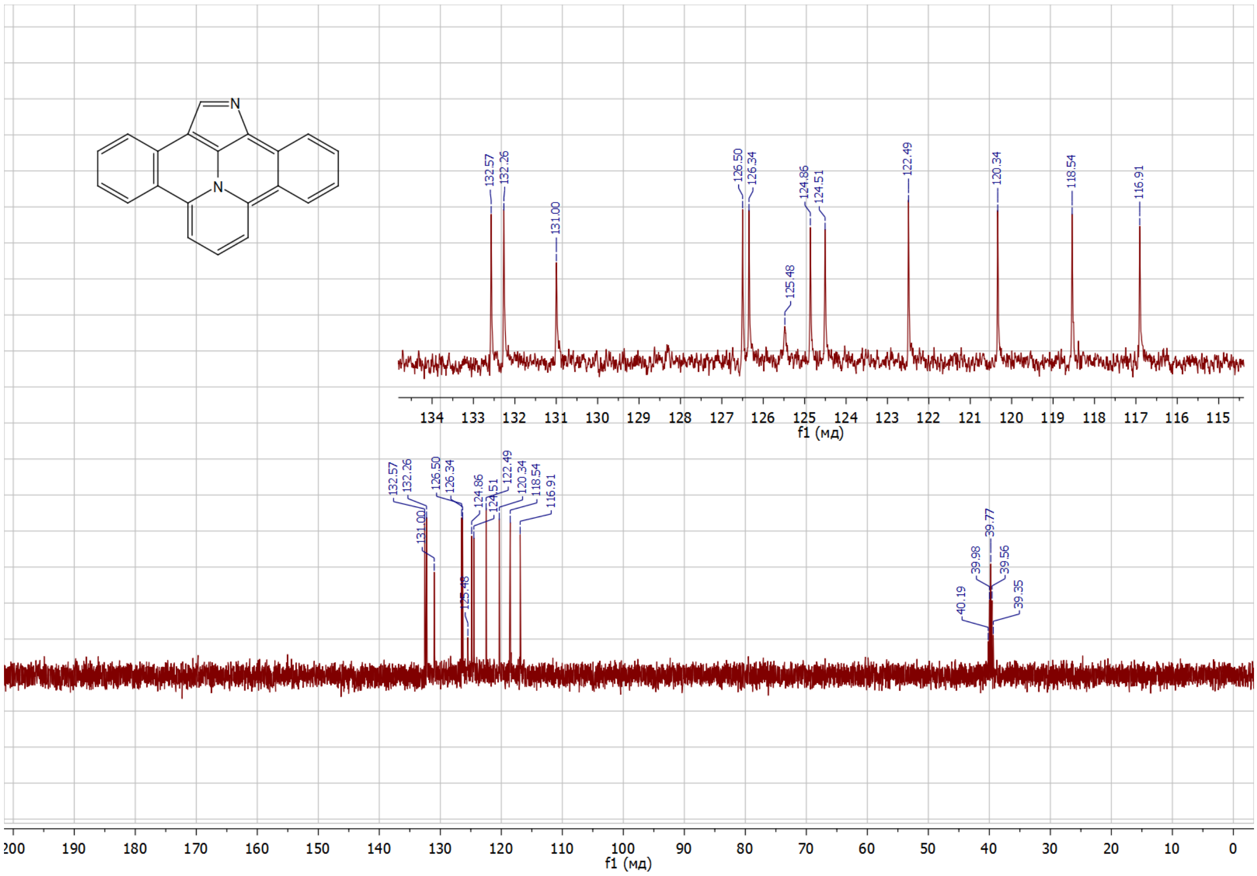

^{1}H NMR (400 MHz, DMSO-d_6) of 1-(2-(2-bromophenyl)-2-oxoethyl)-4-methylpyridin-1-ium bromide **(15b)**

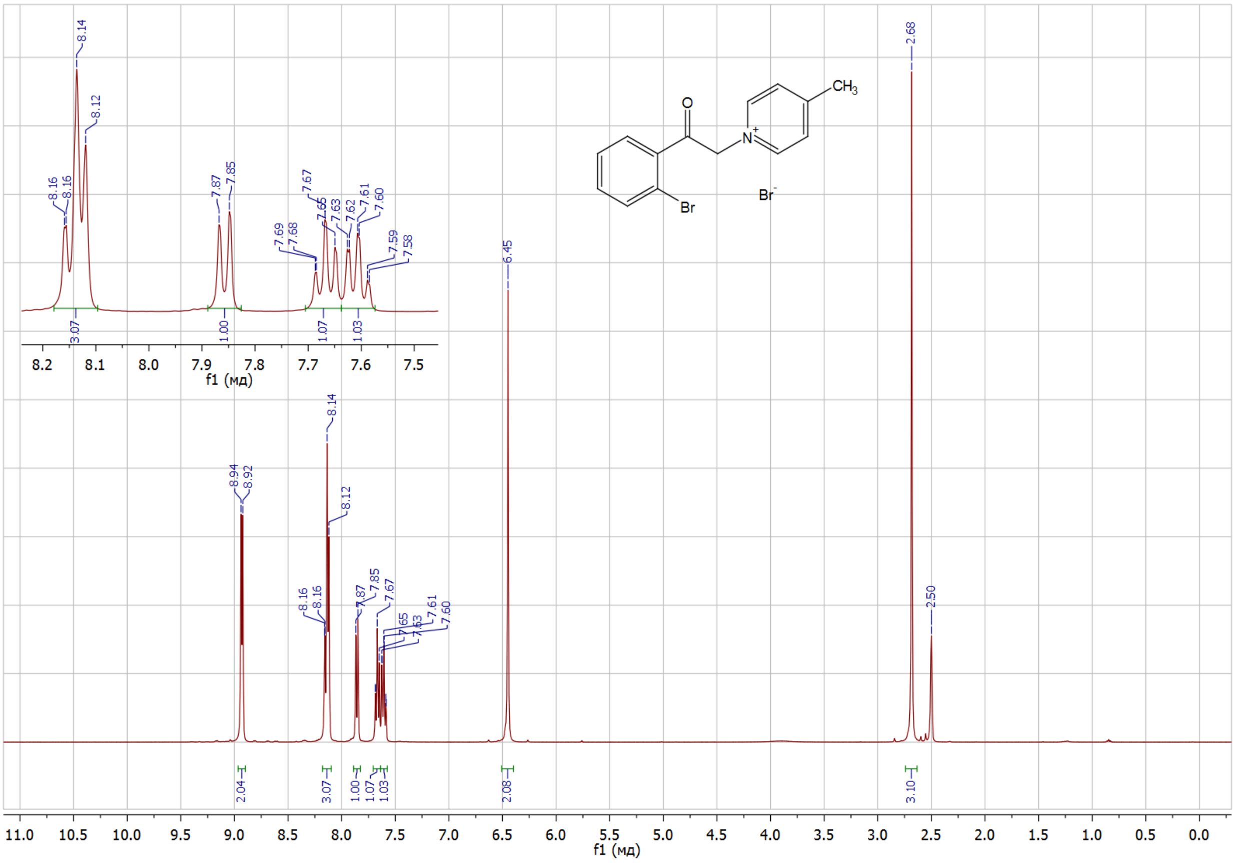

^{13}C{1H} NMR (101 MHz, DMSO-d_6) of 1-(2-(2-bromophenyl)-2-oxoethyl)-4-methylpyridin-1-ium bromide **(15b)**

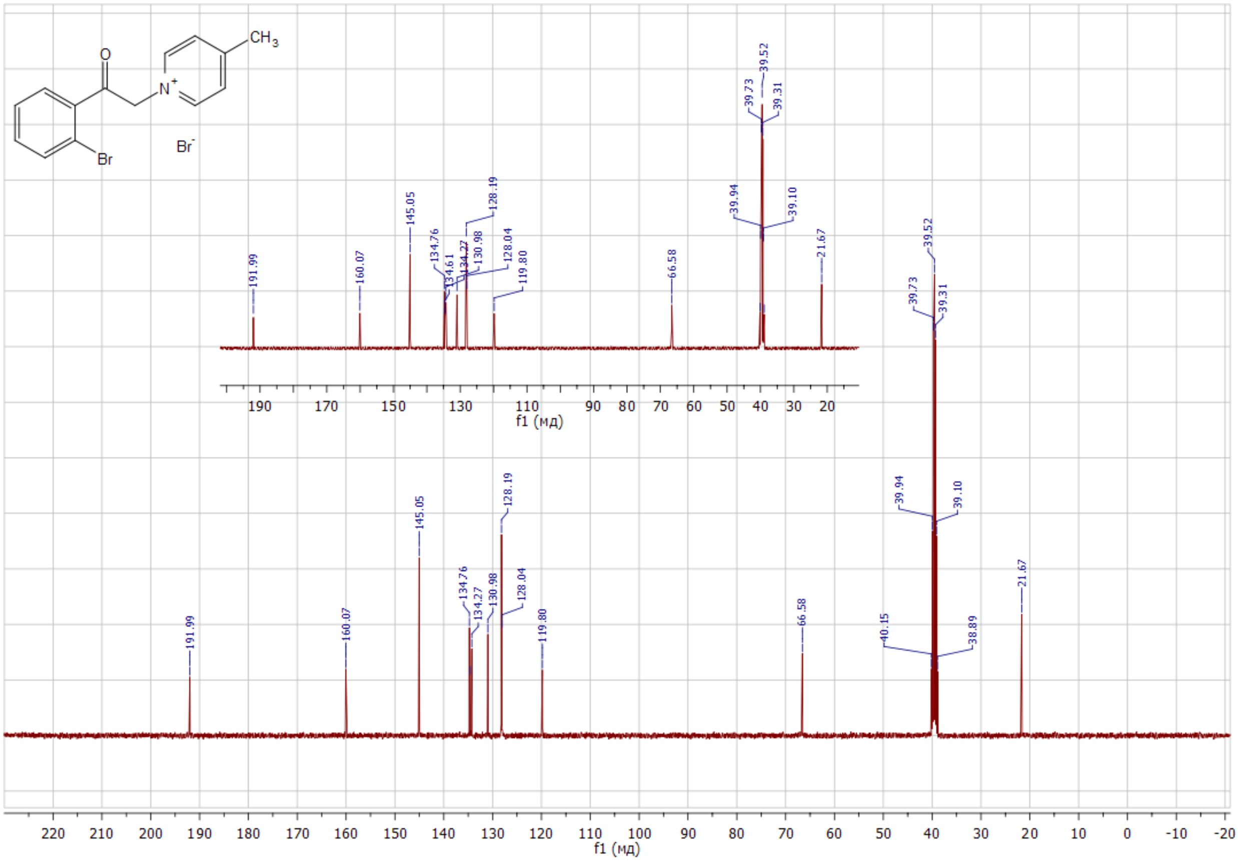

^{13}C DEPT135 NMR (101 MHz, DMSO-d_6) of 1-(2-(2-bromophenyl)-2-oxoethyl)-4-methylpyridin-1-ium bromide **(15b)**

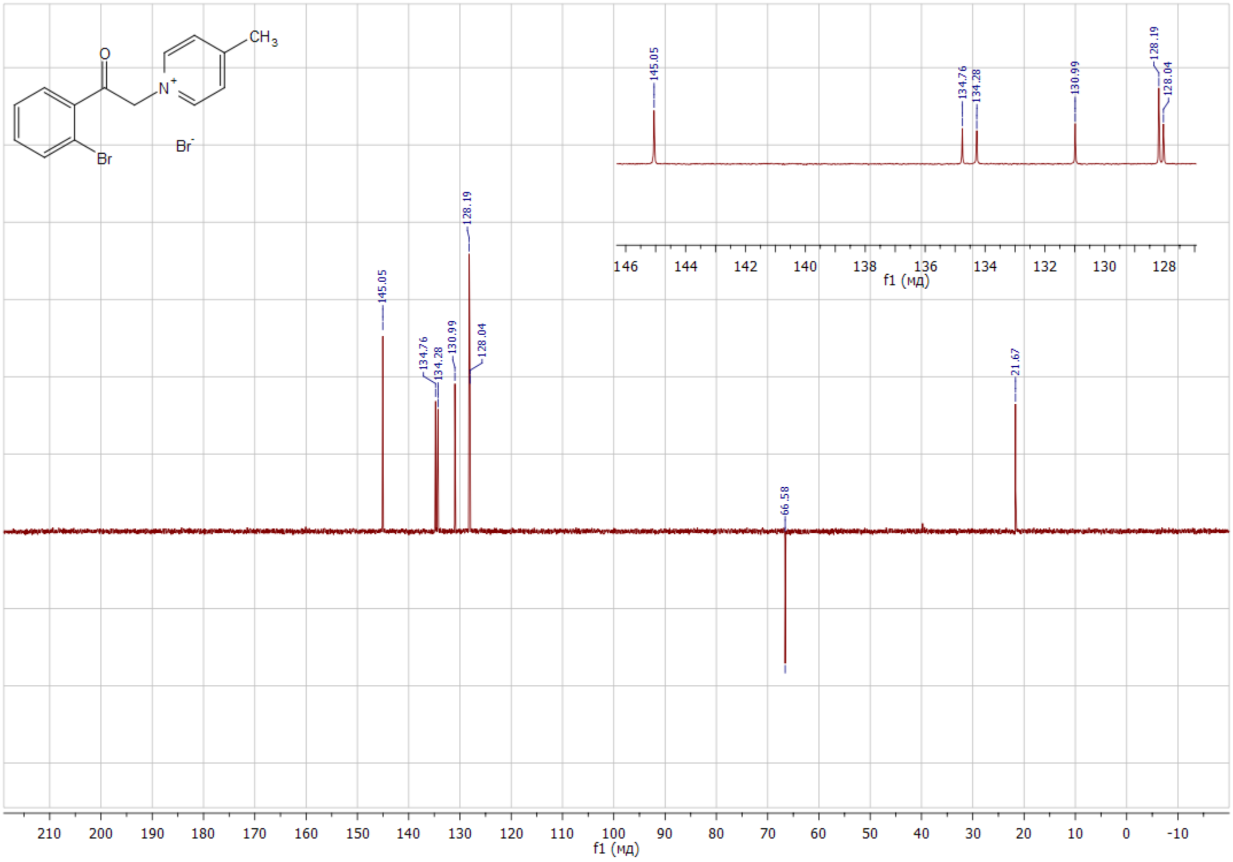

^{1}H NMR (400 MHz, DMSO-d_6) of 1-(2-(2-bromophenyl)-2-oxoethyl)-4-(4-methoxyphenyl)pyridin-1-ium bromide **(15e)**

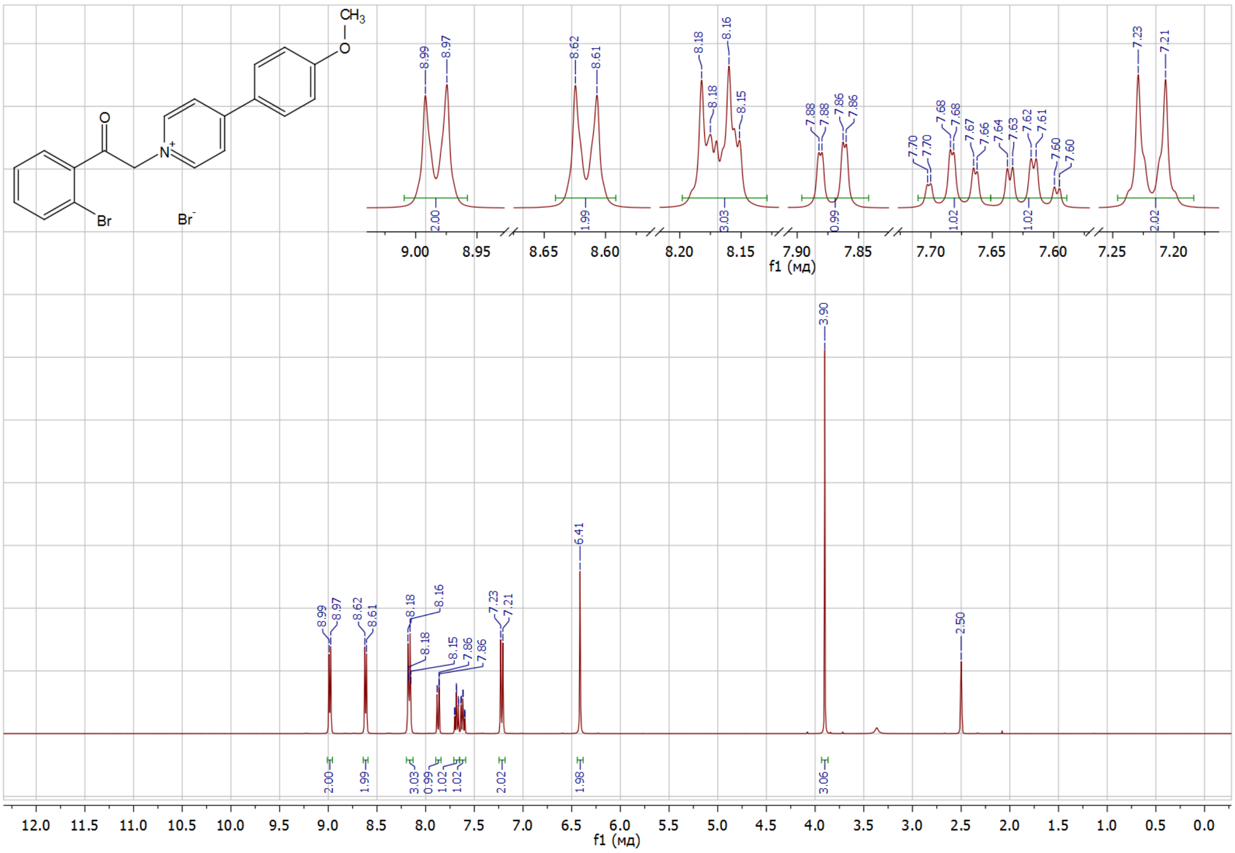

^{13}C{1H} NMR (101 MHz, DMSO-d_6) of 1-(2-(2-bromophenyl)-2-oxoethyl)-4-(4-methoxyphenyl)pyridin-1-ium bromide **(15e)**

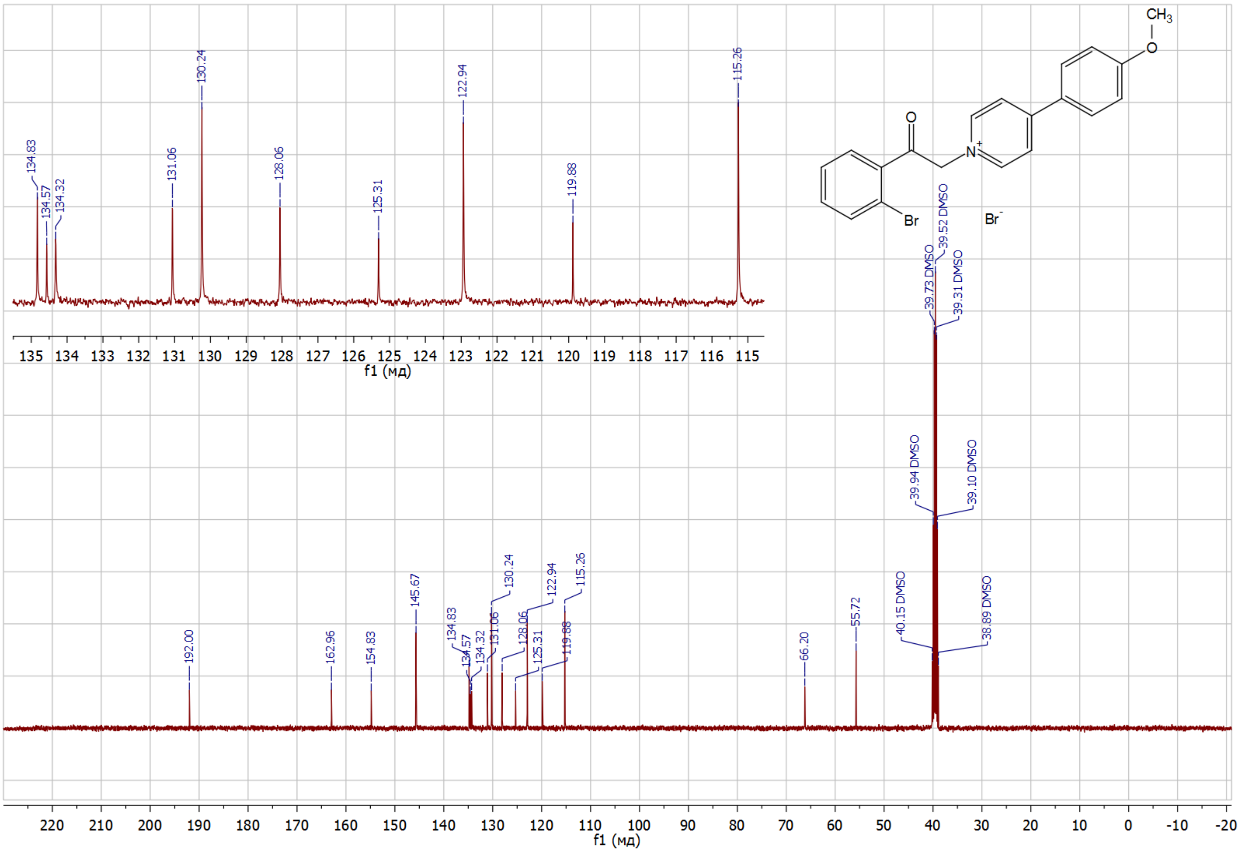

^{13}C DEPT135 NMR (101 MHz, DMSO-d_6) of 1-(2-(2-bromophenyl)-2-oxoethyl)-4-(4-methoxyphenyl)pyridin-1-ium bromide **(15e)**

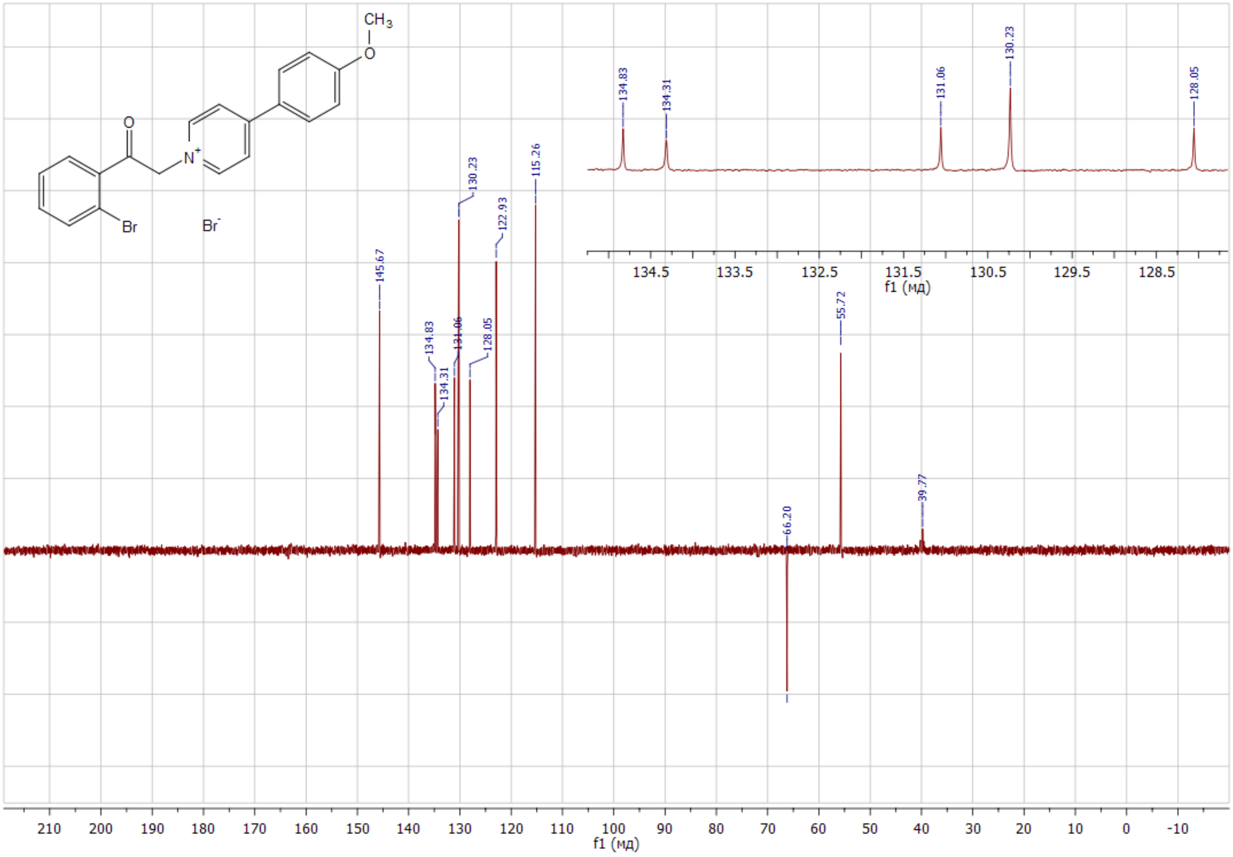

^{1}H NMR (400 MHz, DMSO-d_6) of 2-(2-(2-bromophenyl)-2-oxoethyl)isoquinolin-2-ium bromide **(15g)**

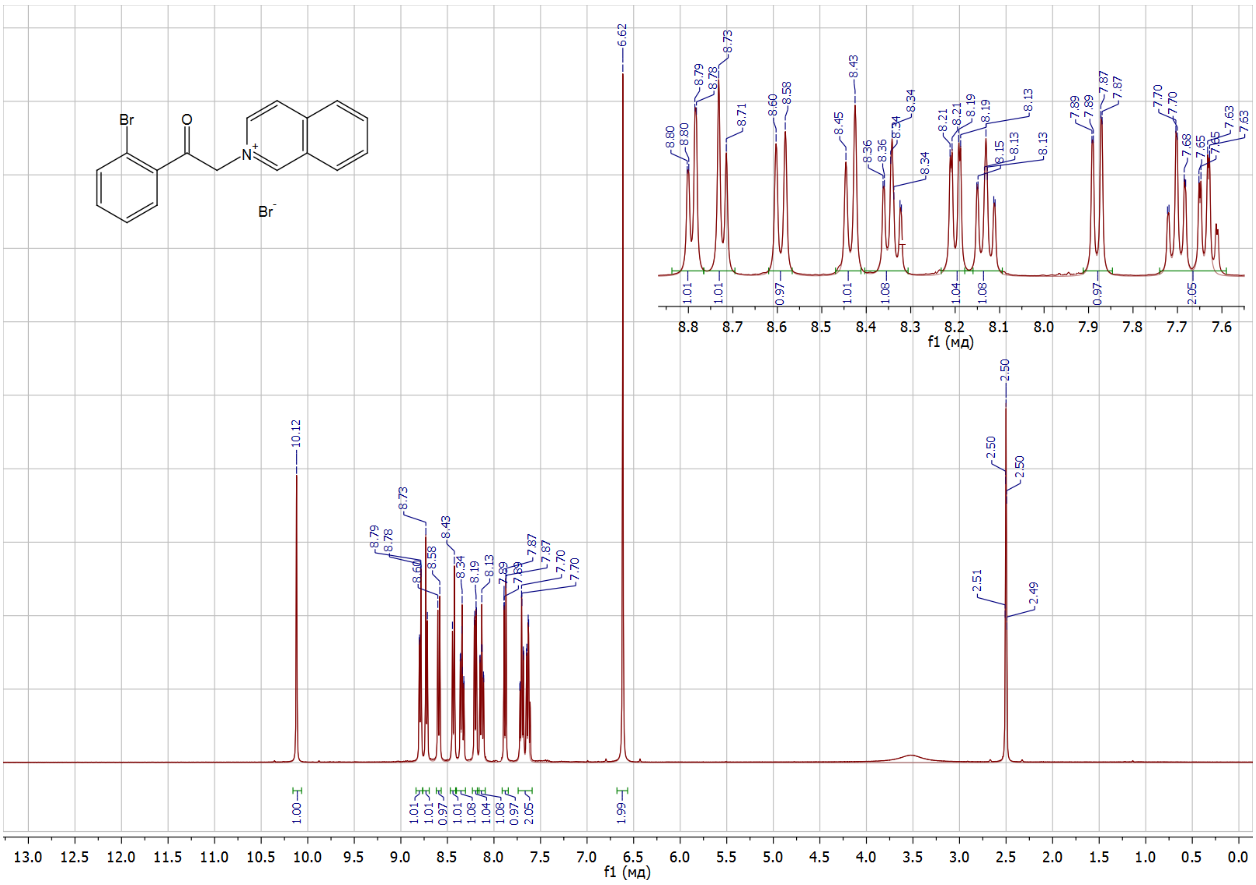

^{13}C{1H} NMR (101 MHz, DMSO-d_6) of 2-(2-(2-bromophenyl)-2-oxoethyl)isoquinolin-2-ium bromide **(15g)**

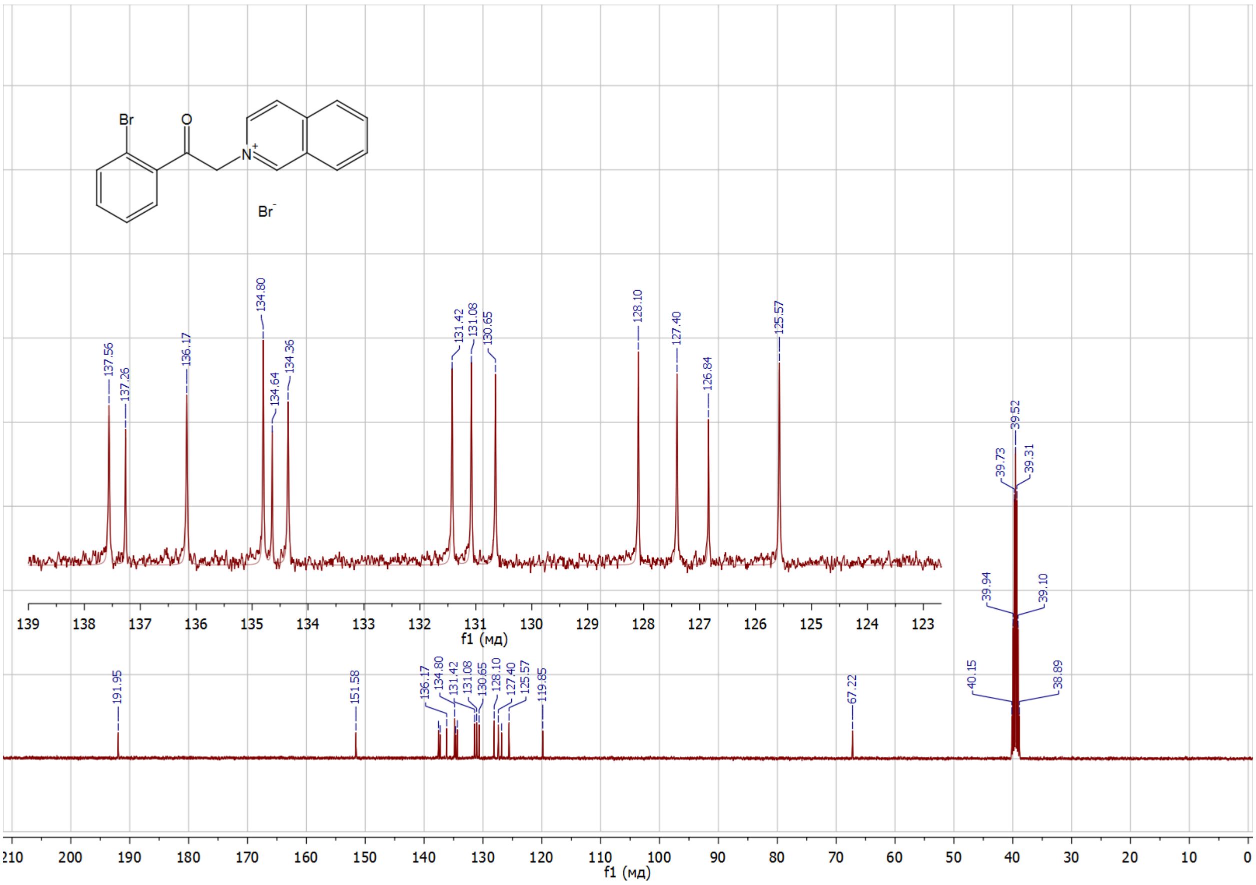

^{13}C DEPT135 NMR (101 MHz, DMSO-d_6) of 2-(2-(2-bromophenyl)-2-oxoethyl)isoquinolin-2-ium bromide **(15g)**

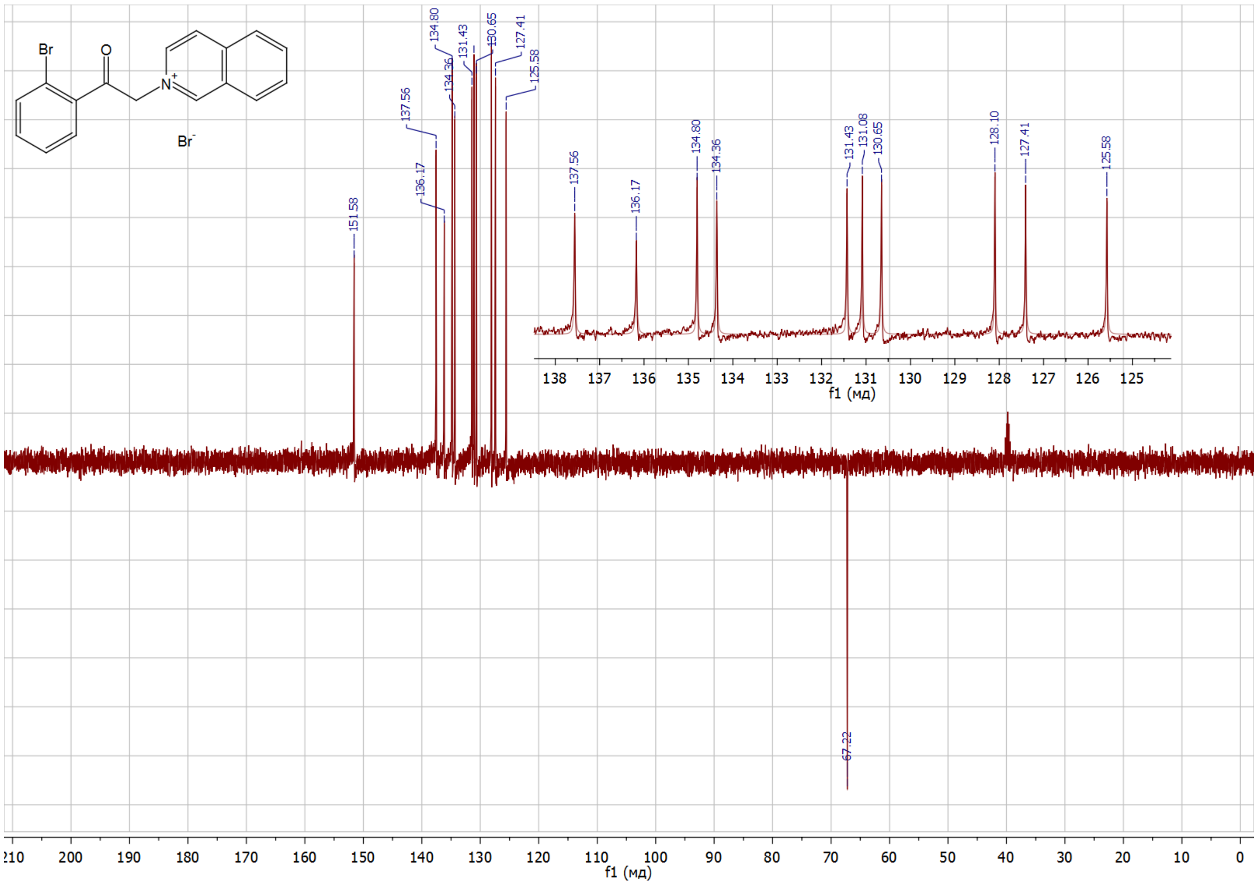

^{1}H NMR (400 MHz, DMSO-d_6) of 4-benzoyl-1-(2-(2-bromophenyl)-2-oxoethyl)pyridin-1-ium bromide **(15j)**

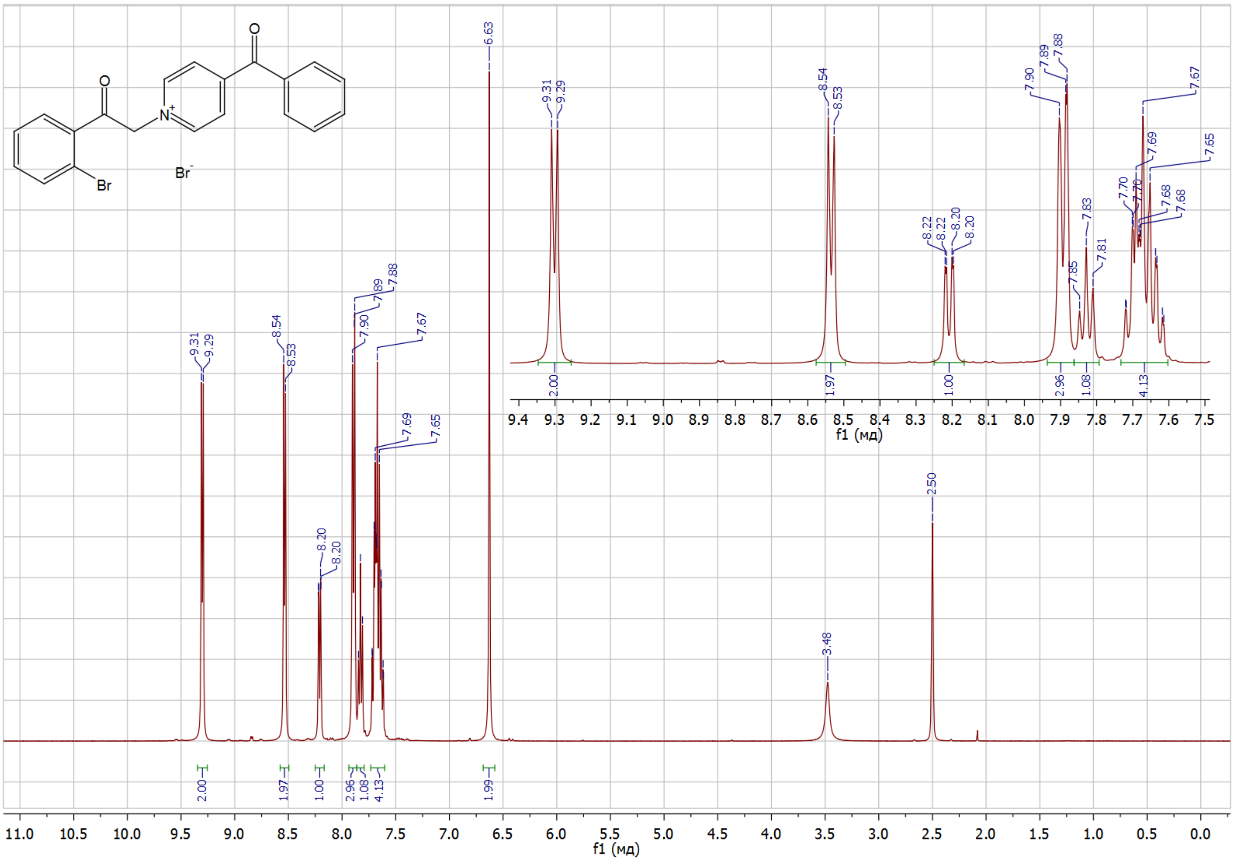

^{13}C{1H} NMR (101 MHz, DMSO-d_6) of 4-benzoyl-1-(2-(2-bromophenyl)-2-oxoethyl)pyridin-1-ium bromide **(15j)**

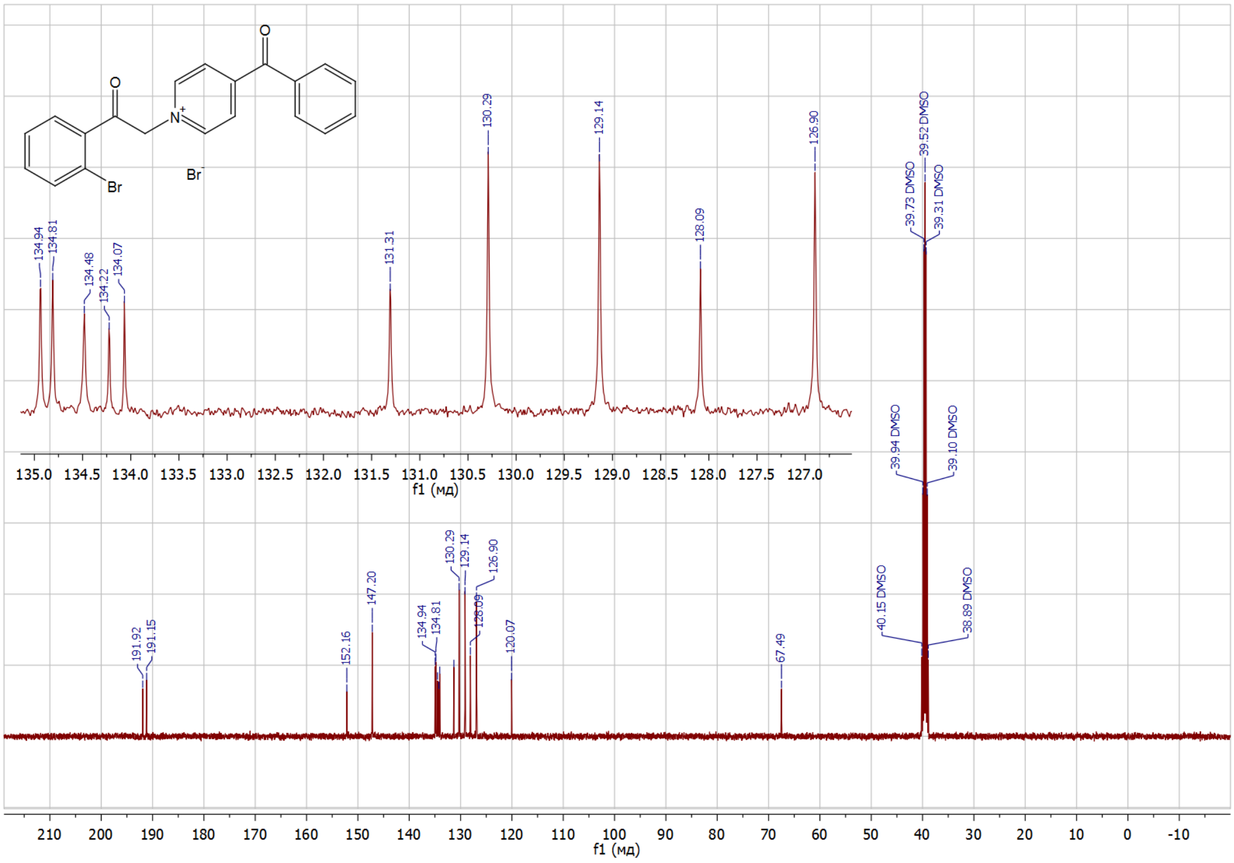

^{13}C DEPT135 NMR (101 MHz, DMSO-d_6) of 4-benzoyl-1-(2-(2-bromophenyl)-2-oxoethyl)pyridin-1-ium bromide **(15j)**

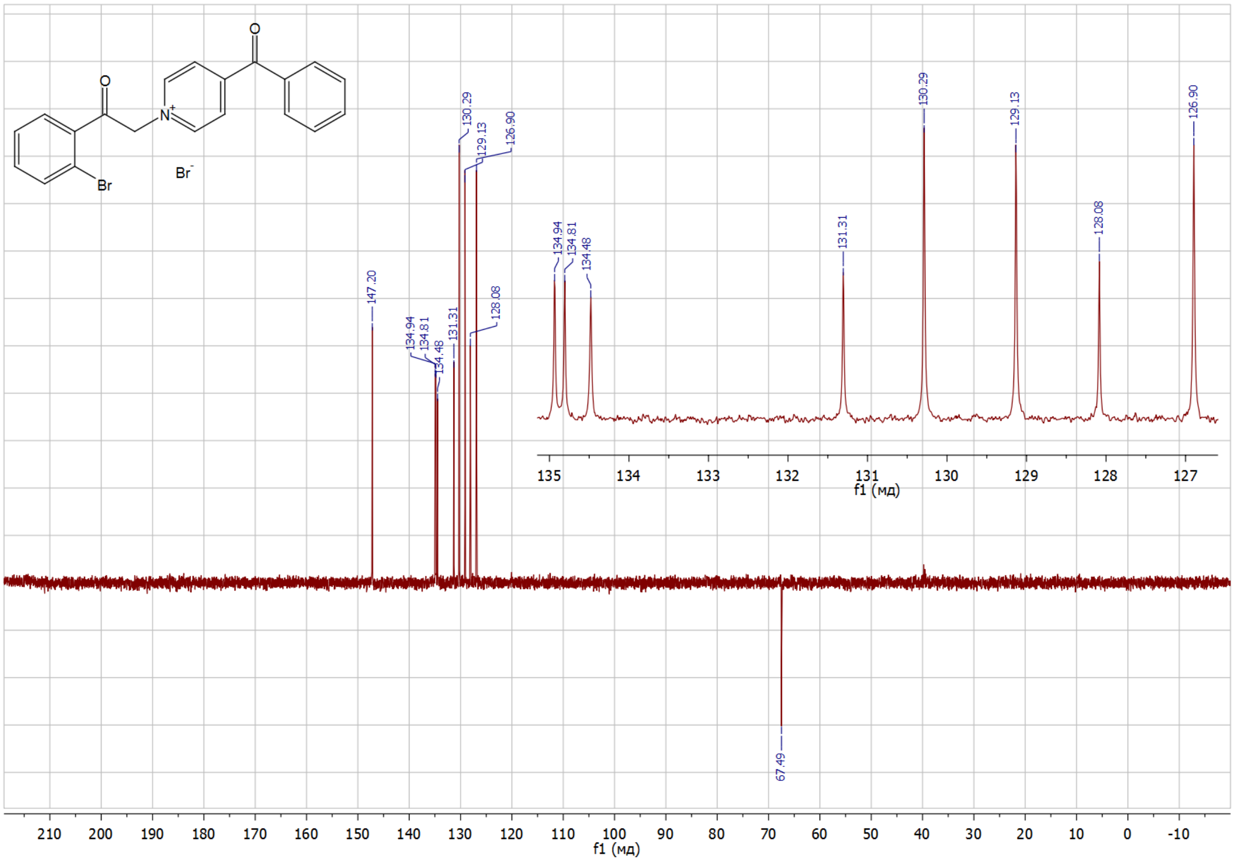

^{1}H NMR (400 MHz, DMSO-d_6) of 1-(2-(2-bromophenyl)-4-phenyl-1*H*-pyrrol-3-yl)-4-methylpyridin-1-ium bromide (**16b**)

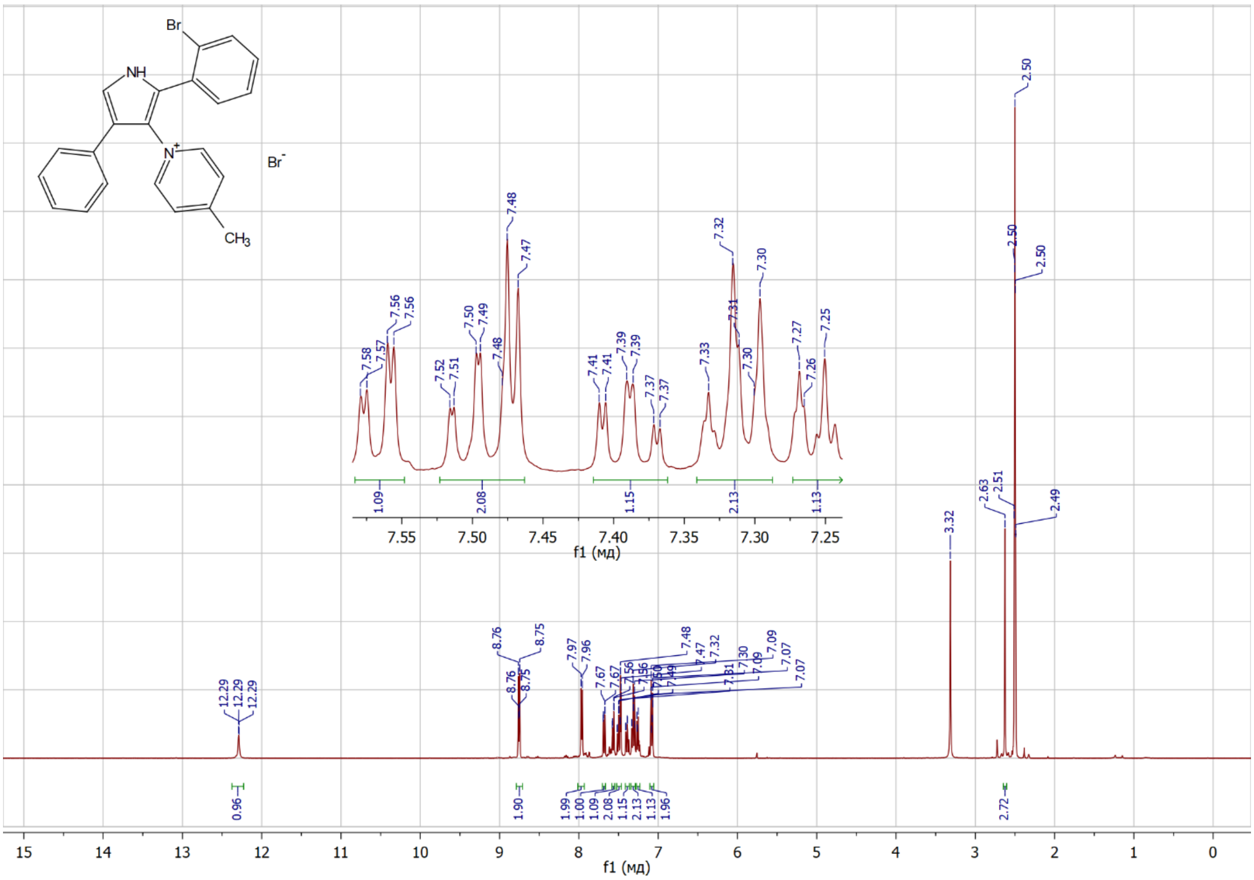

$^{13}C\{^{1}H\}$ NMR (101 MHz, DMSO-d_6) of 1-(2-(2-bromophenyl)-4-phenyl-1*H*-pyrrol-3-yl)-4-methylpyridin-1-ium bromide (**16b**)

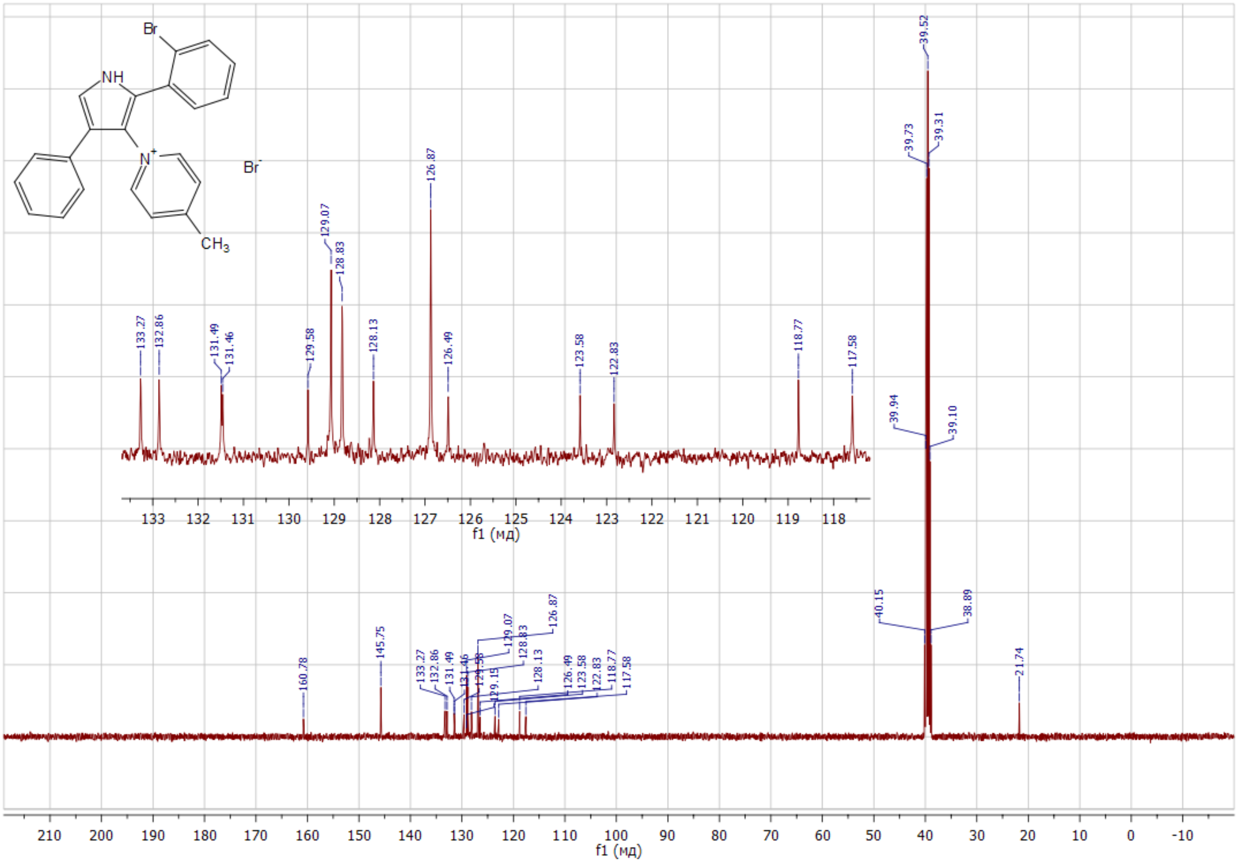

^{13}C DEPT135 NMR (101 MHz, DMSO-d_6) of 1-(2-(2-bromophenyl)-4-phenyl-1*H*-pyrrol-3-yl)-4-methylpyridin-1-ium bromide (**16b**)

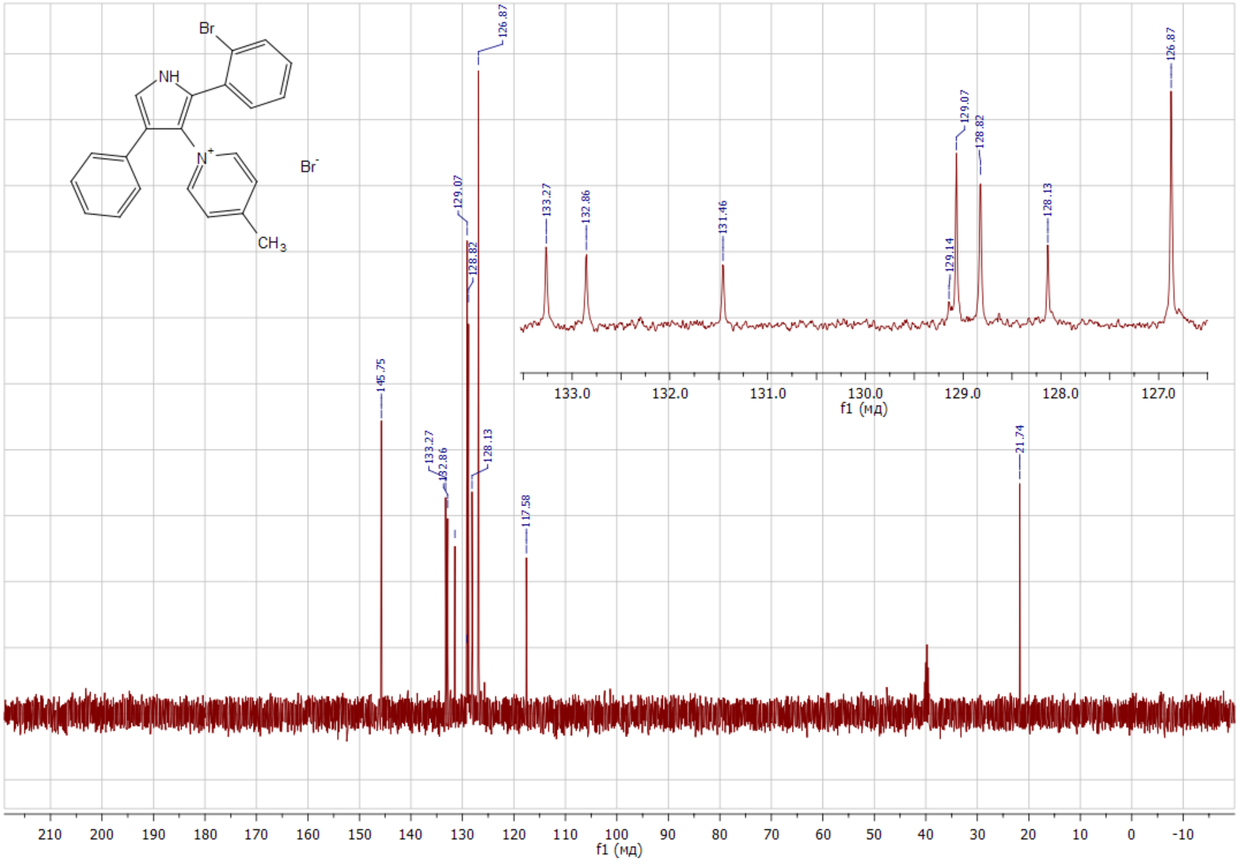

^{1}H NMR (500 MHz, DMSO-d_6) of 1-(2-(2-Bromophenyl)-4-phenyl-1*H*-pyrrol-3-yl)-4-methoxypyridin-1-ium bromide (**16c**)

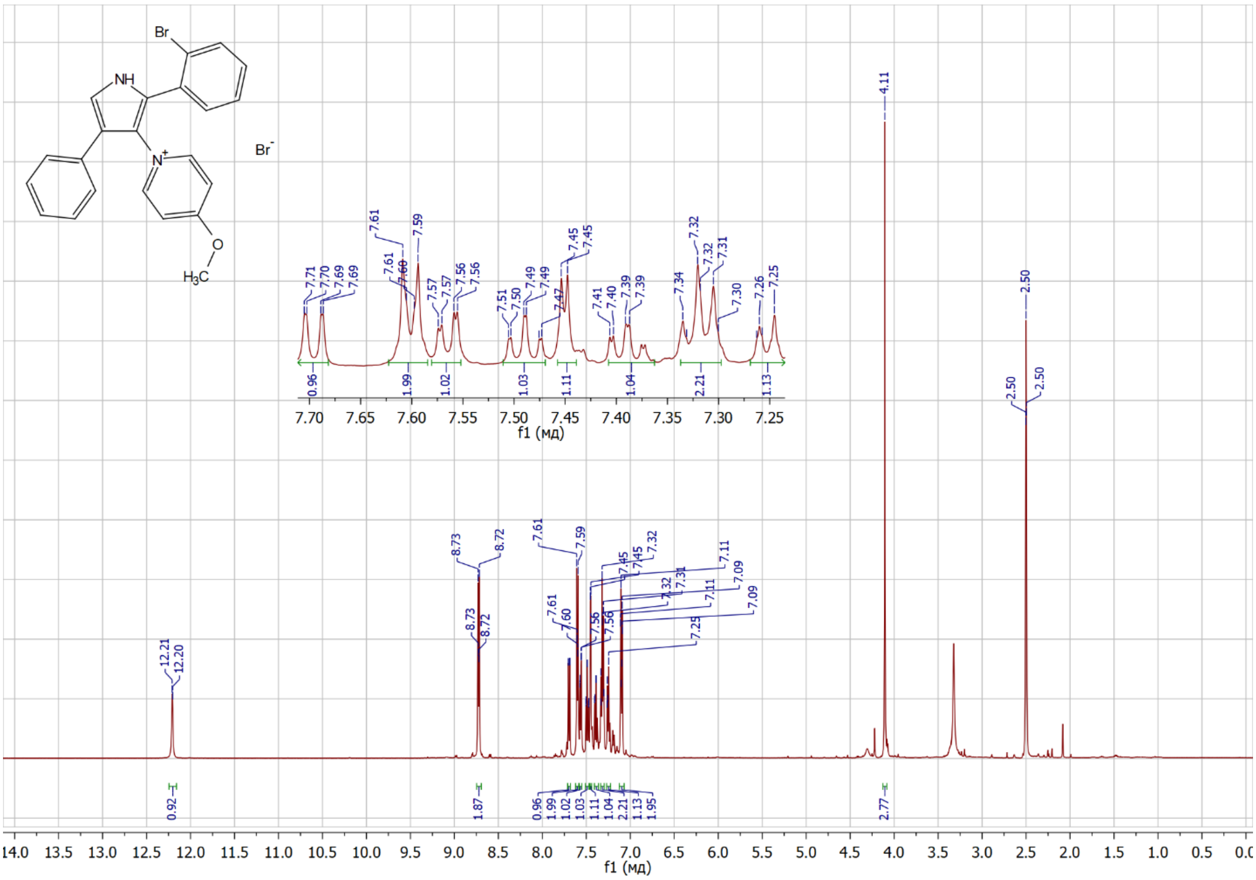

$^{13}C\{1H\}$ NMR (126 MHz, DMSO-d_6) of 1-(2-(2-Bromophenyl)-4-phenyl-1*H*-pyrrol-3-yl)-4-methoxypyridin-1-ium bromide (**16c**)

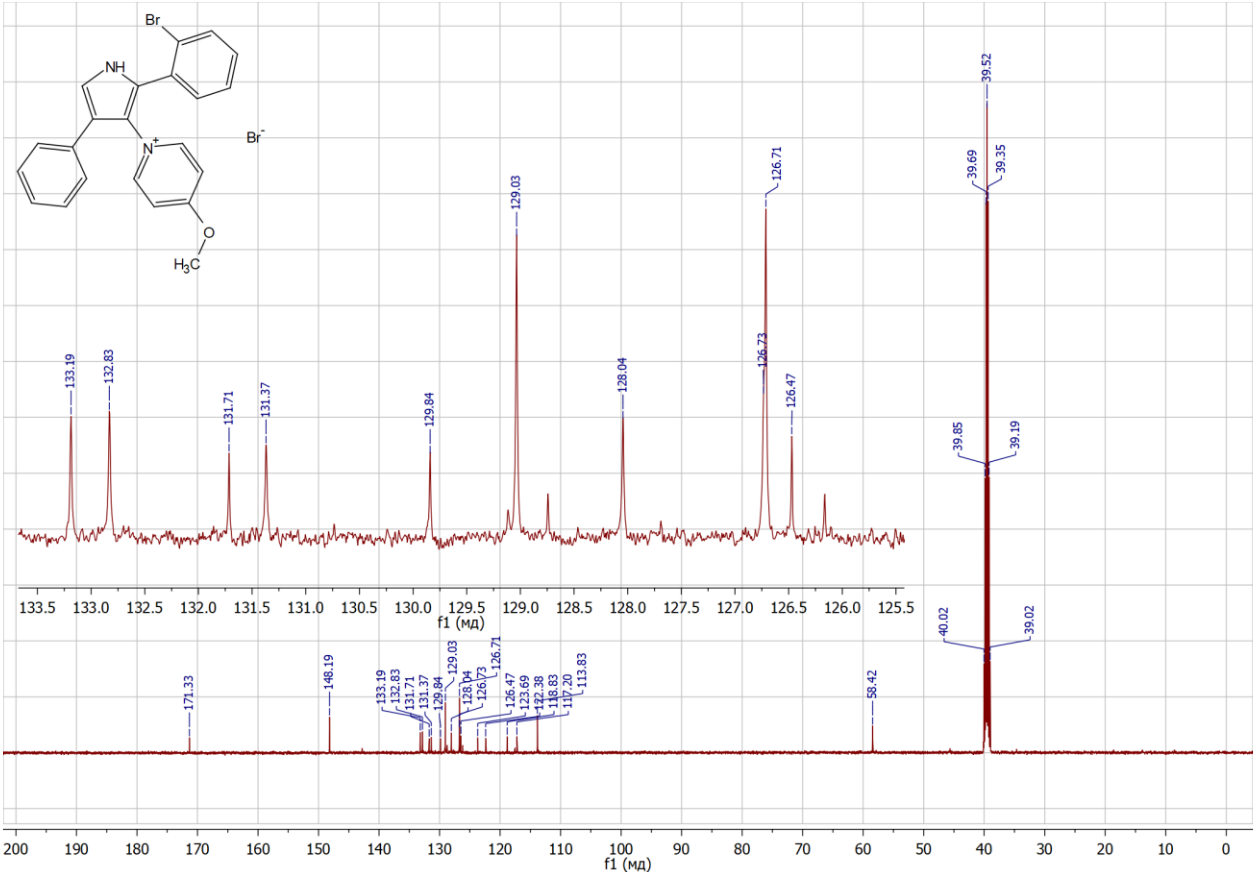

^{13}C DEPT135 NMR (126 MHz, DMSO-d_6) of 1-(2-(2-Bromophenyl)-4-phenyl-1*H*-pyrrol-3-yl)-4-methoxypyridin-1-ium bromide (**16c**)

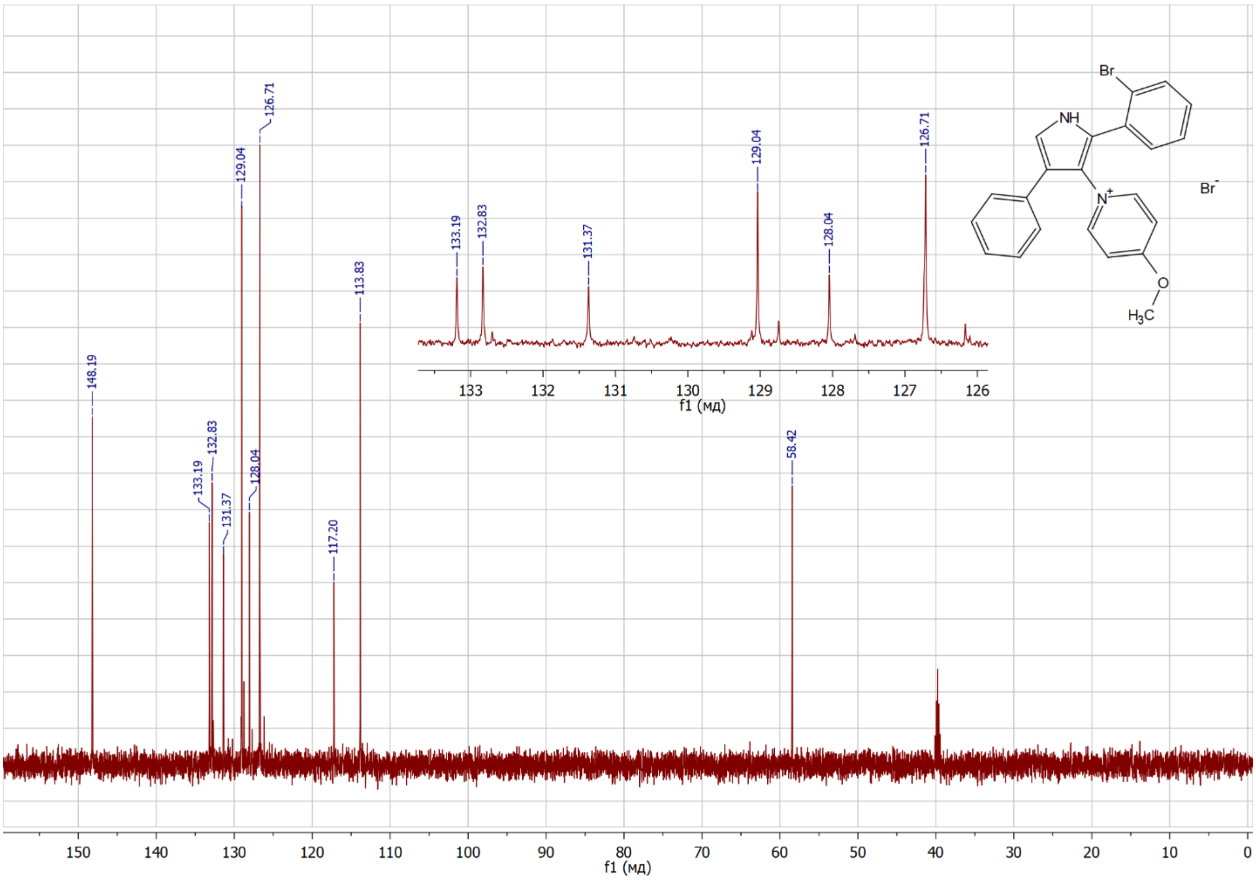

^{1}H NMR (400 MHz, DMSO-d_6) of 1-(2-(2-bromophenyl)-4-phenyl-1*H*-pyrrol-3-yl)-4-(4-methoxyphenyl)pyridin-1-ium bromide (**16e**)

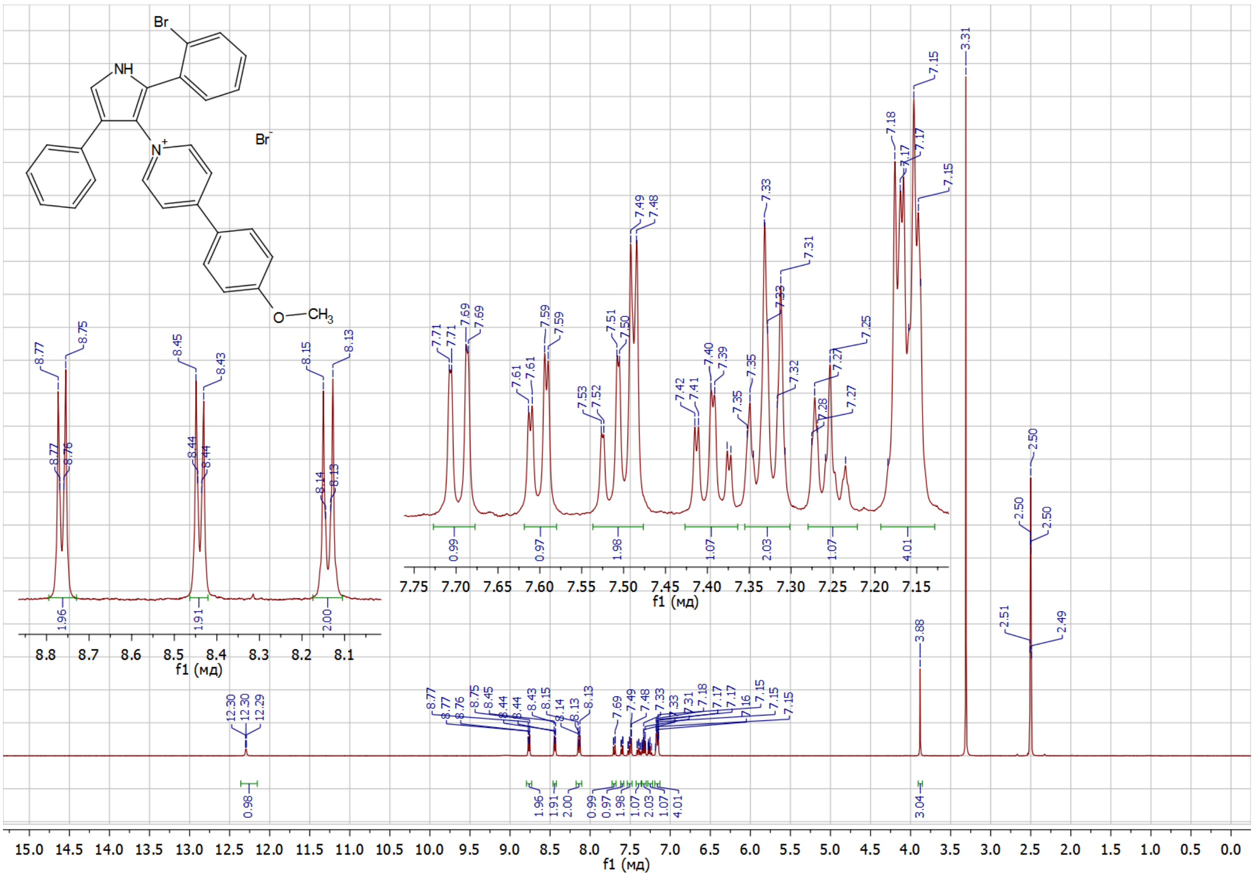

^{13}C{1H} NMR (101 MHz, DMSO-d_6) of 1-(2-(2-bromophenyl)-4-phenyl-1*H*-pyrrol-3-yl)-4-(4-methoxyphenyl)pyridin-1-ium bromide (**16e**)

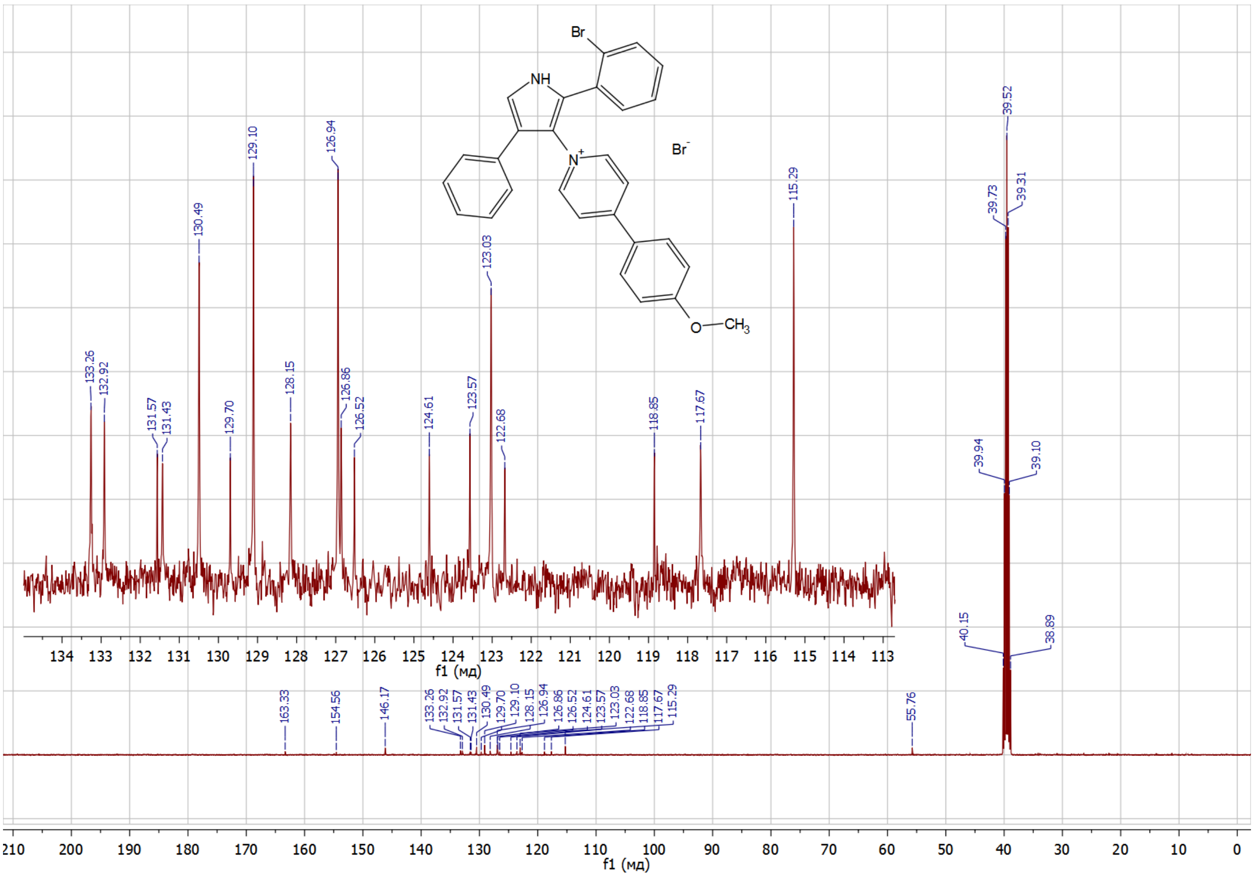

^{13}C DEPT135 NMR (101 MHz, DMSO-d_6) of 1-(2-(2-bromophenyl)-4-phenyl-1*H*-pyrrol-3-yl)-4-(4-methoxyphenyl)pyridin-1-ium bromide (**16e**)

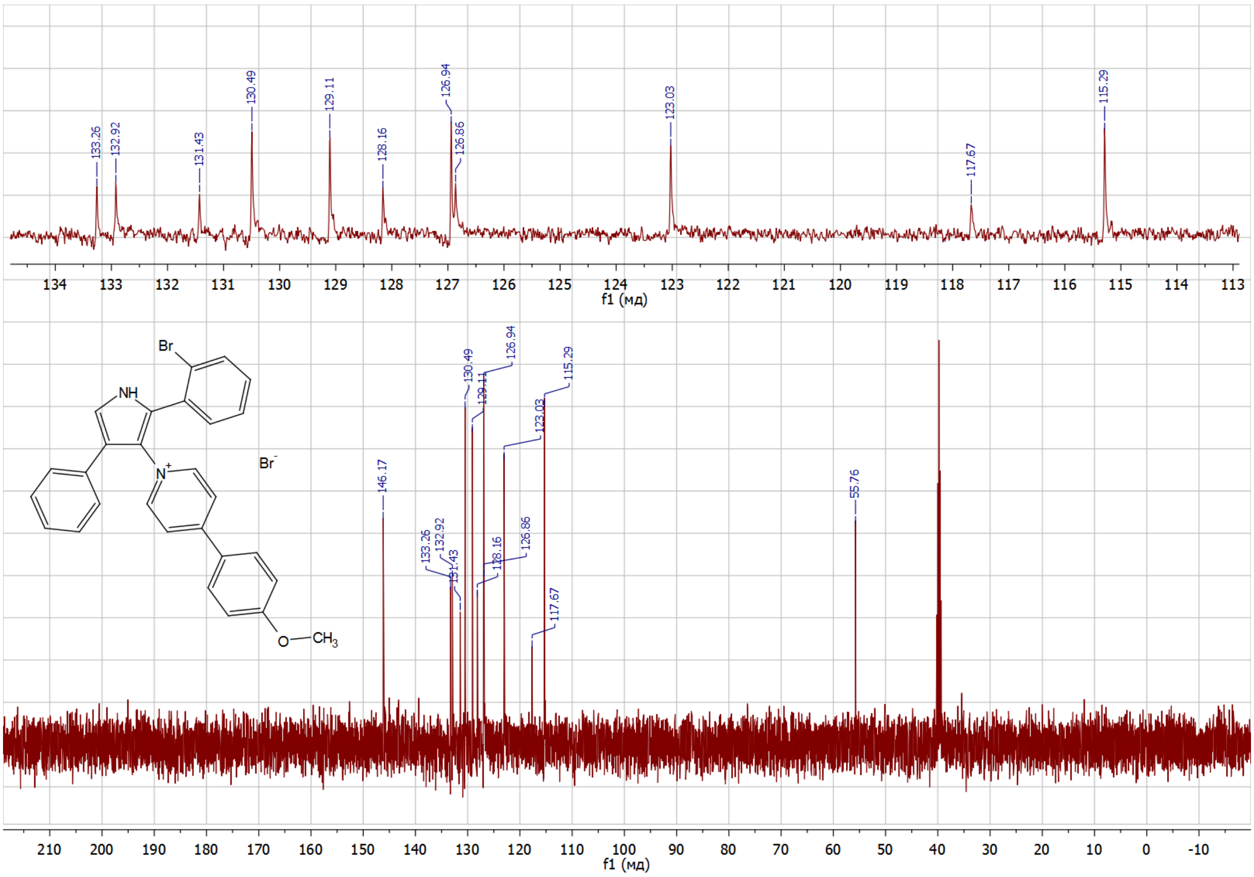

^{1}H NMR (400 MHz, DMSO-d_6) of 1-(2-(2-bromophenyl)-4-phenyl-1*H*-pyrrol-3-yl)-3,5-dimethylpyridin-1-ium bromide (**16f**)

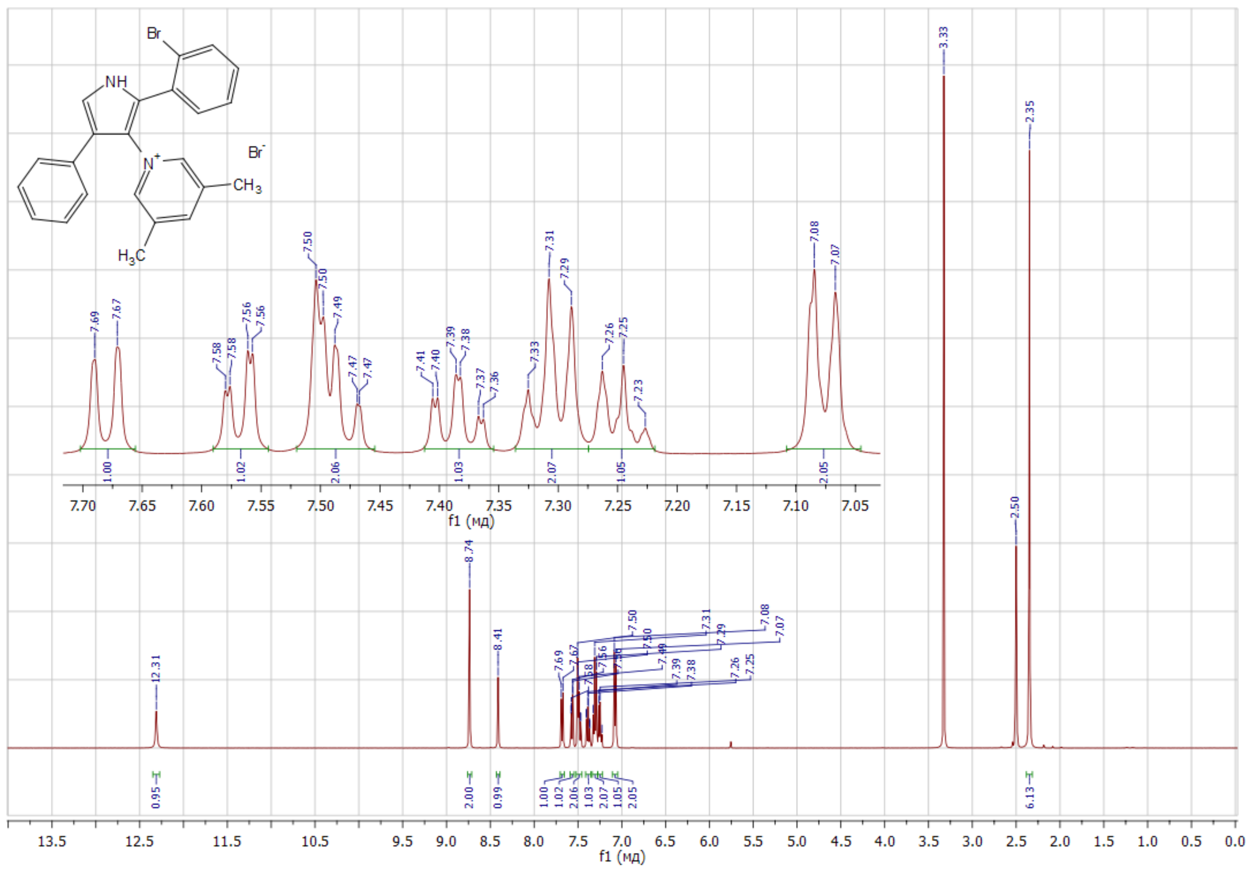

$^{13}C\{1H\}$ NMR (101 MHz, DMSO-d_6) of 1-(2-(2-bromophenyl)-4-phenyl-1*H*-pyrrol-3-yl)-3,5-dimethylpyridin-1-ium bromide (**16f**)

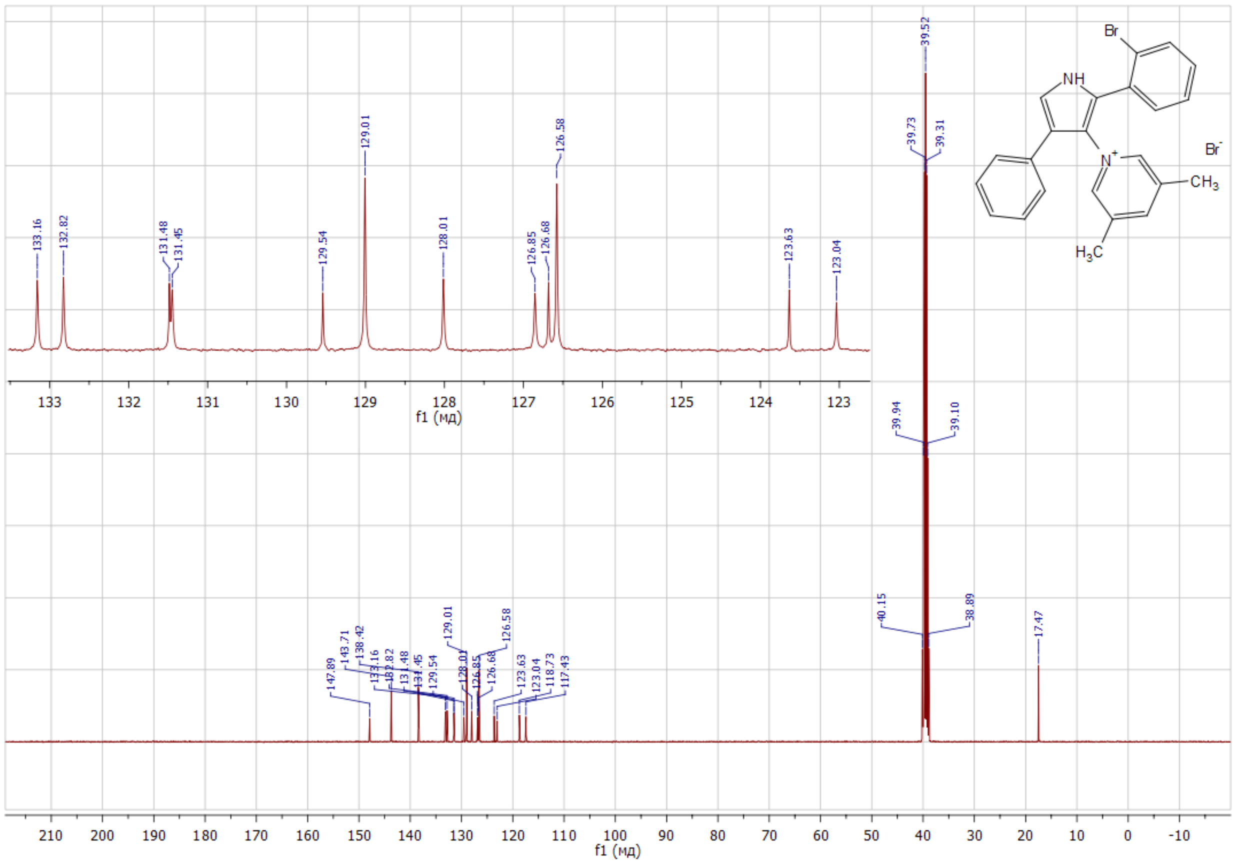

^{13}C DEPT135 NMR (101 MHz, DMSO-d_6) of 1-(2-(2-bromophenyl)-4-phenyl-1*H*-pyrrol-3-yl)-3,5-dimethylpyridin-1-ium bromide (**16f**)

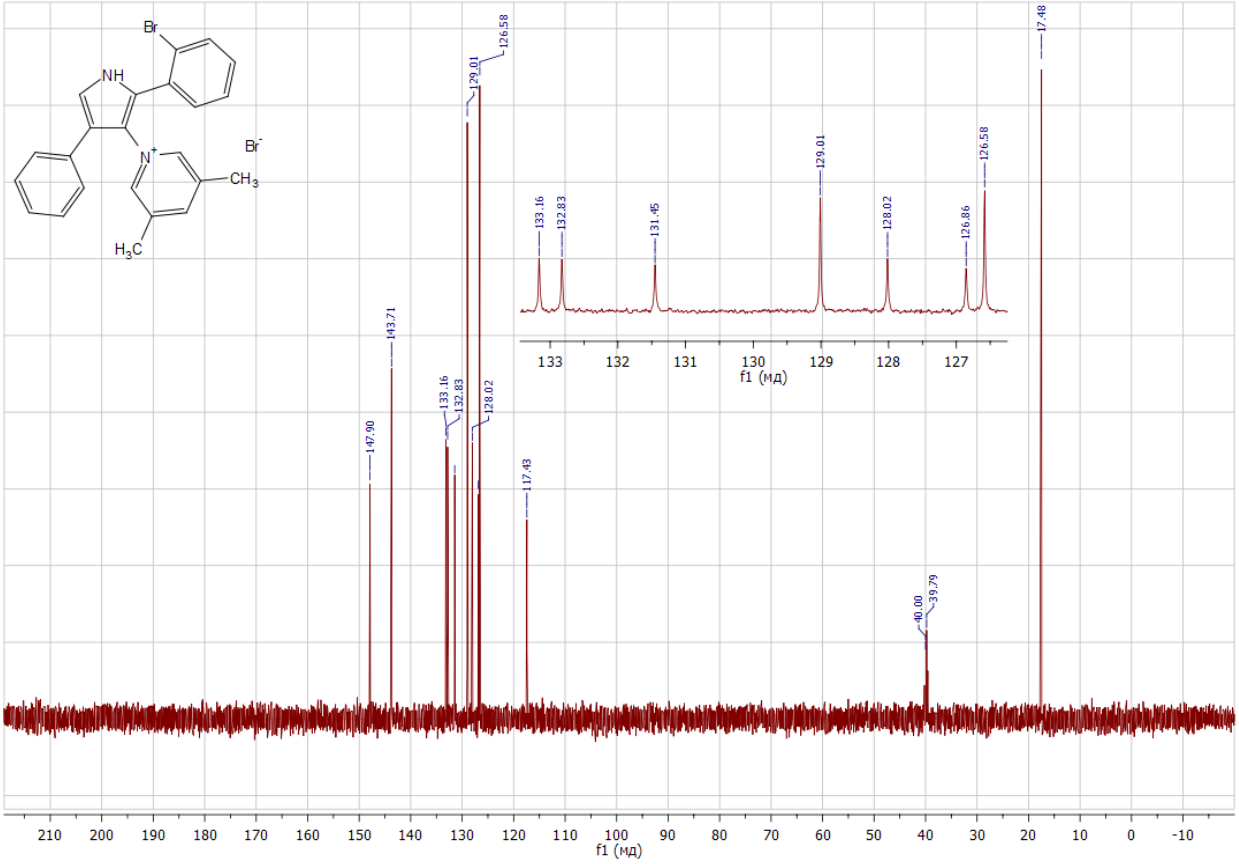

^{1}H NMR (500 MHz, DMSO-d_6) of 2-(2-(2-bromophenyl)-4-phenyl-1*H*-pyrrol-3-yl)isoquinolin-2-ium bromide (**16g**)

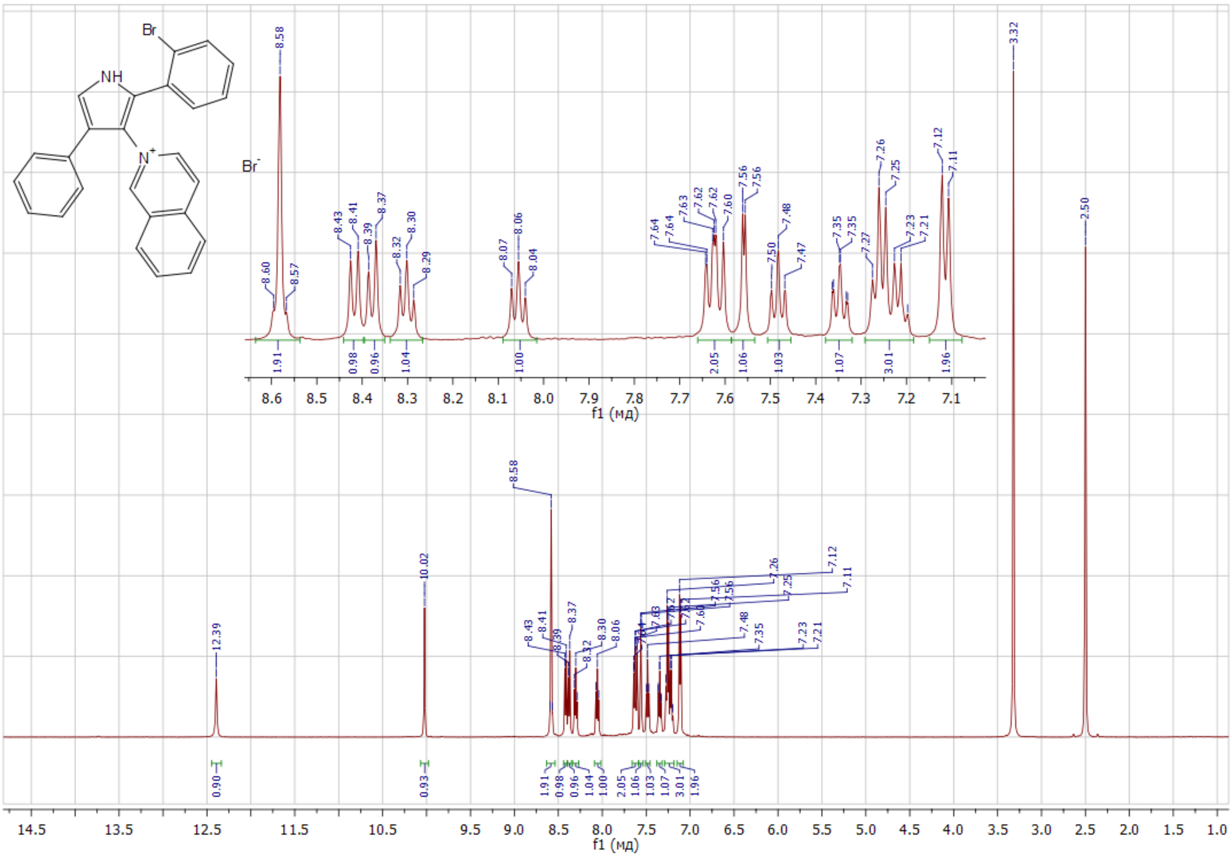

$^{13}C\{^1H\}$ NMR (126 MHz, DMSO-d_6) of 2-(2-(2-bromophenyl)-4-phenyl-1*H*-pyrrol-3-yl)isoquinolin-2-ium bromide (**16g**)

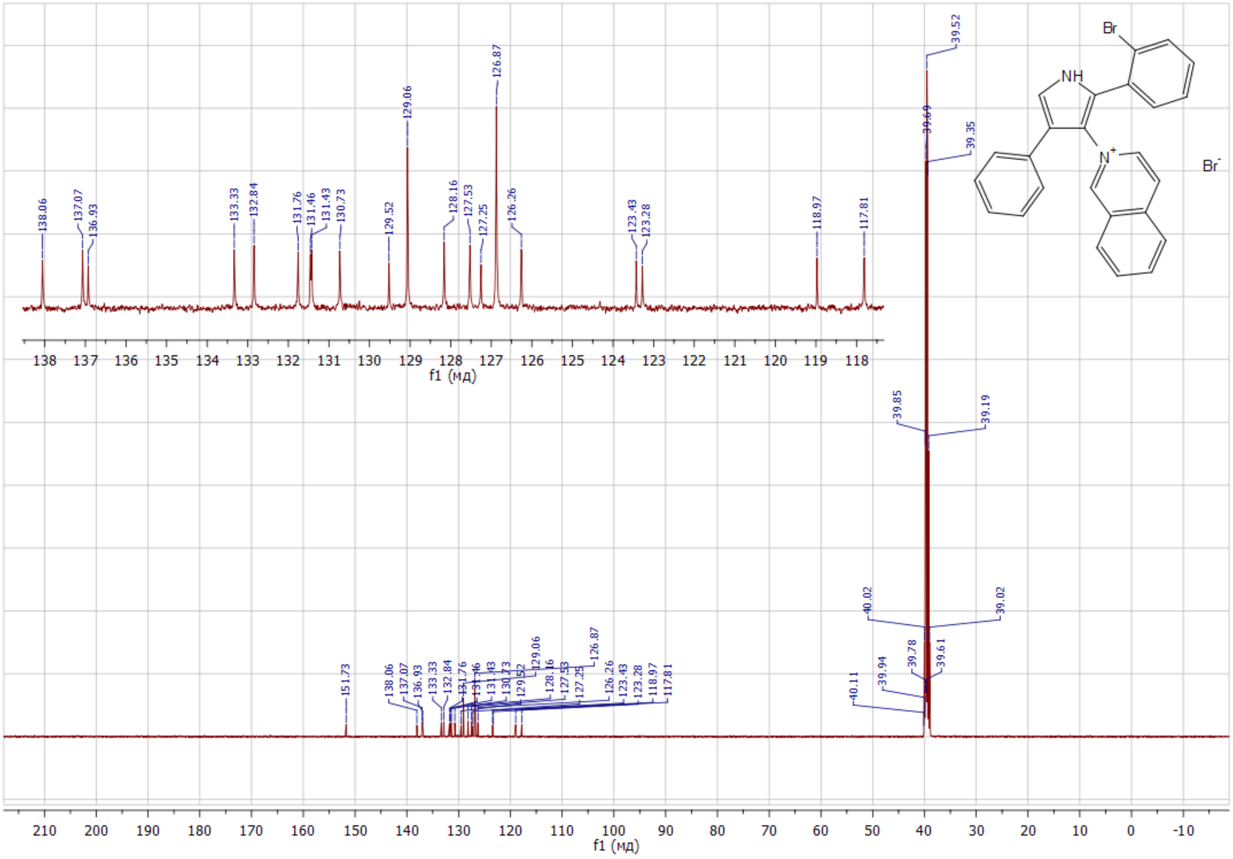

^{13}C DEPT135 NMR (126 MHz, DMSO-d_6) of 2-(2-(2-bromophenyl)-4-phenyl-1H-pyrrol-3-yl)isoquinolin-2-ium bromide (**16g**)

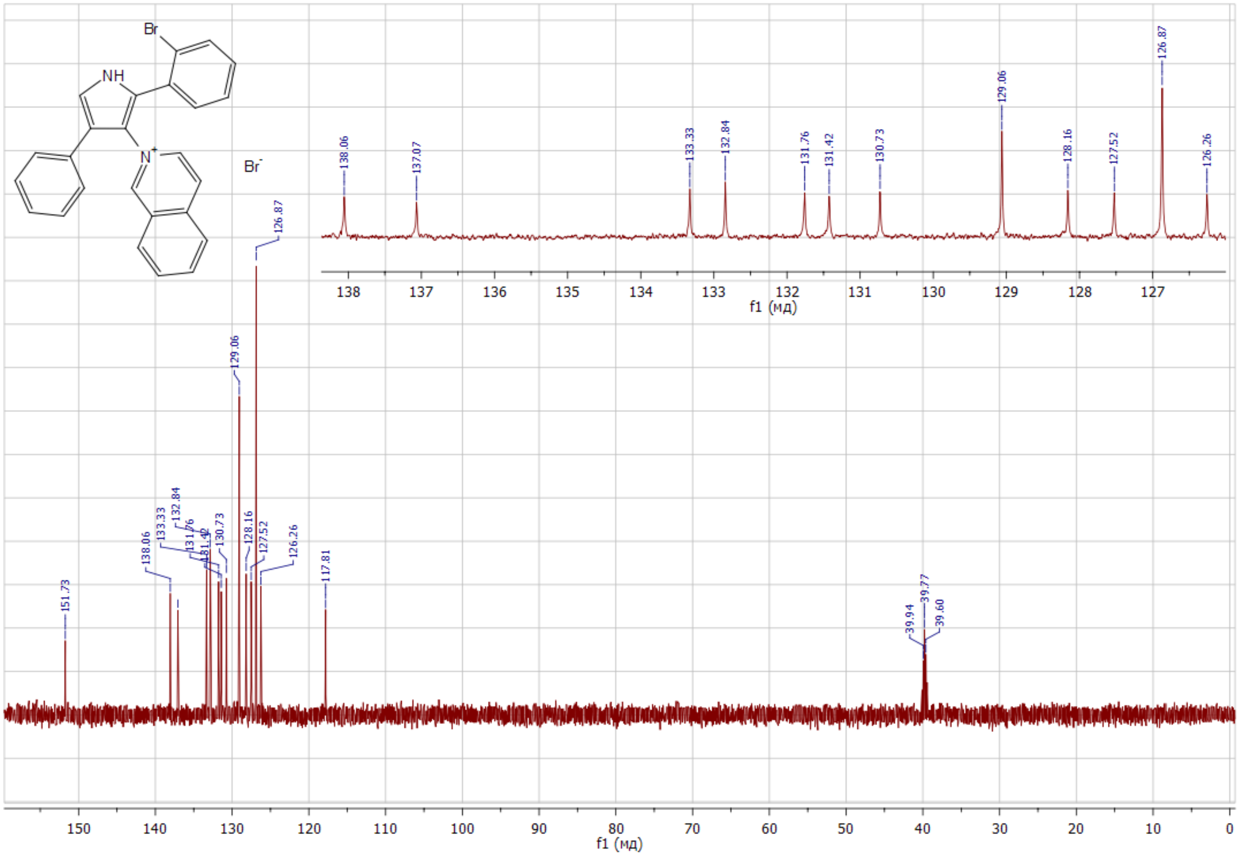

^{1}H NMR (400 MHz, DMSO-d_6) of 1-(2-(2-bromophenyl)-4-(4-methoxyphenyl)-1*H*-pyrrol-3-yl)pyridin-1-ium bromide (**16h**)

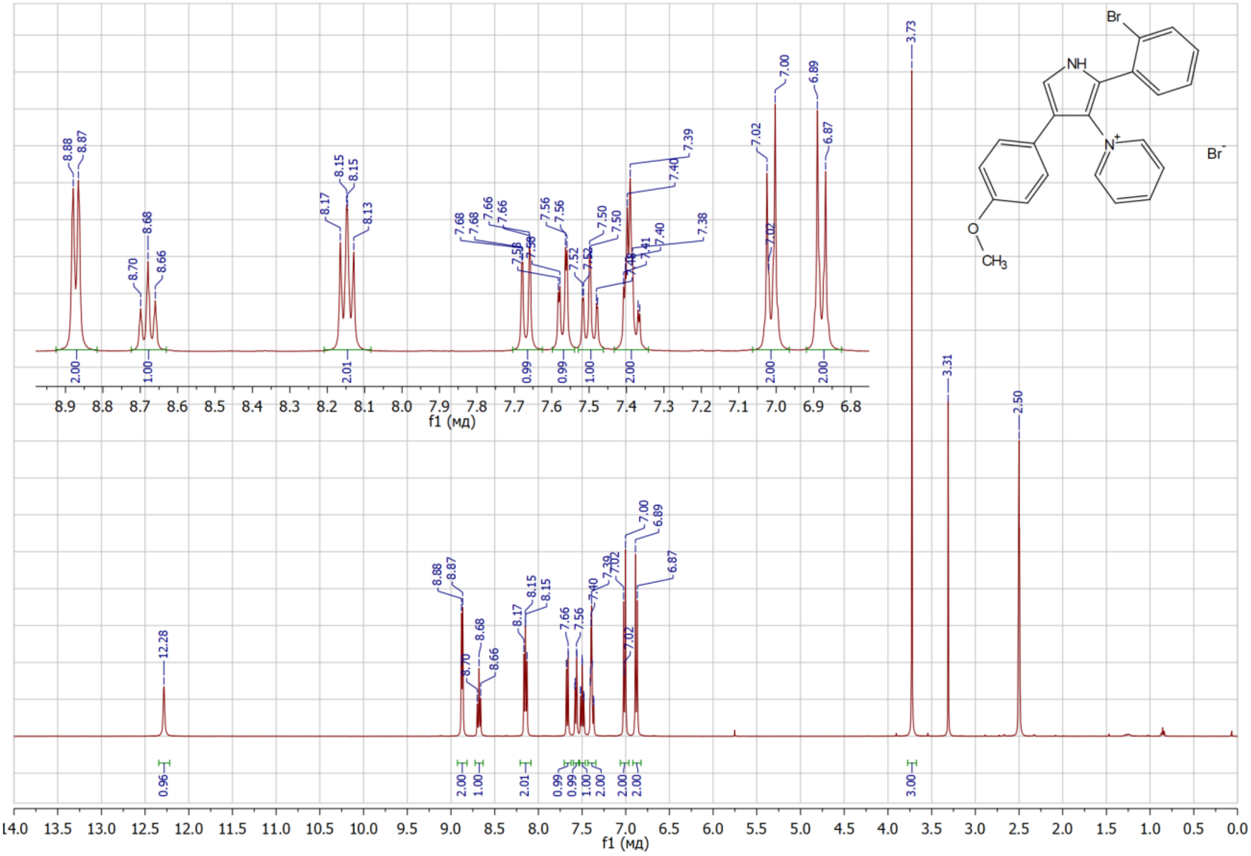

$^{13}C\{^{1}H\}$ NMR (101 MHz, DMSO-d_6) of 1-(2-(2-bromophenyl)-4-(4-methoxyphenyl)-1*H*-pyrrol-3-yl)pyridin-1-ium bromide (**16h**)

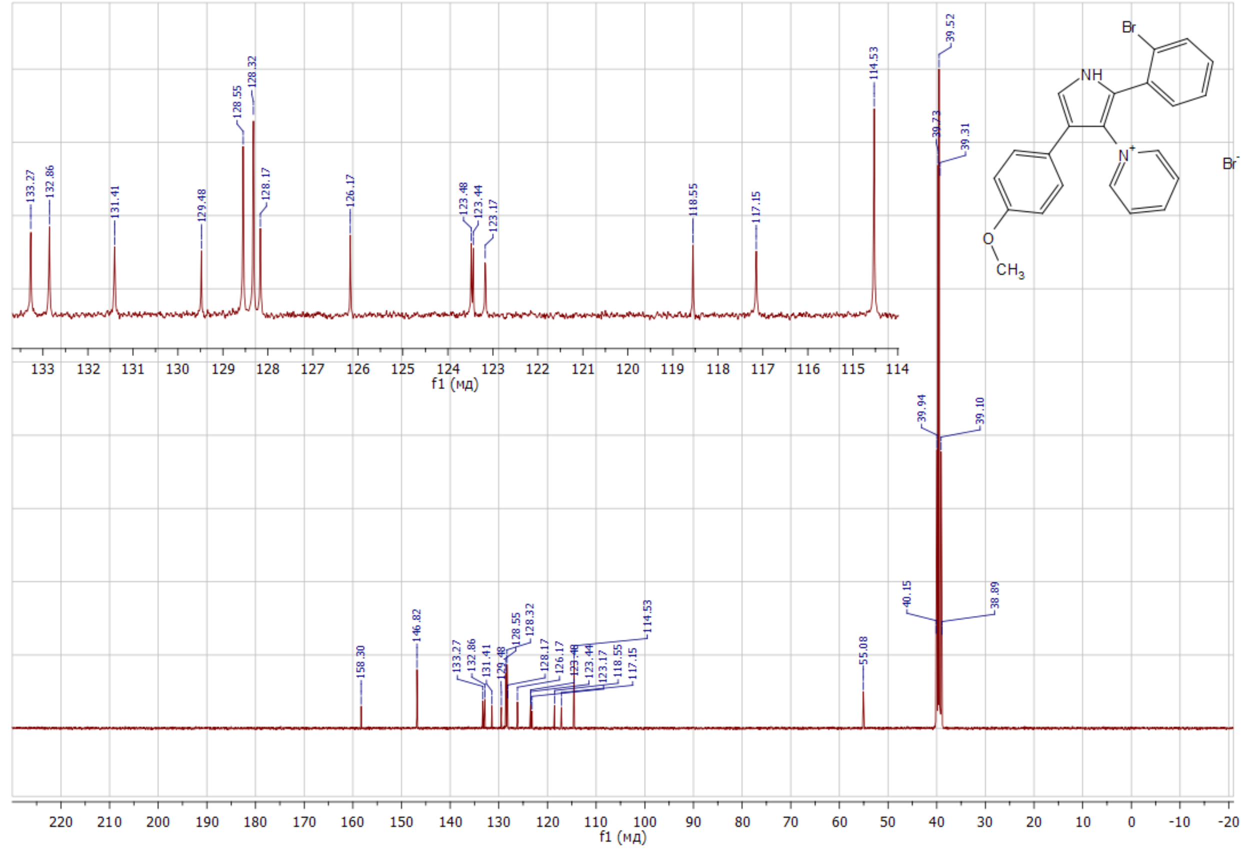

^{13}C DEPT135 NMR (101 MHz, DMSO-d_6) of 1-(2-(2-bromophenyl)-4-(4-methoxyphenyl)-1*H*-pyrrol-3-yl)pyridin-1-ium bromide (**16h**)

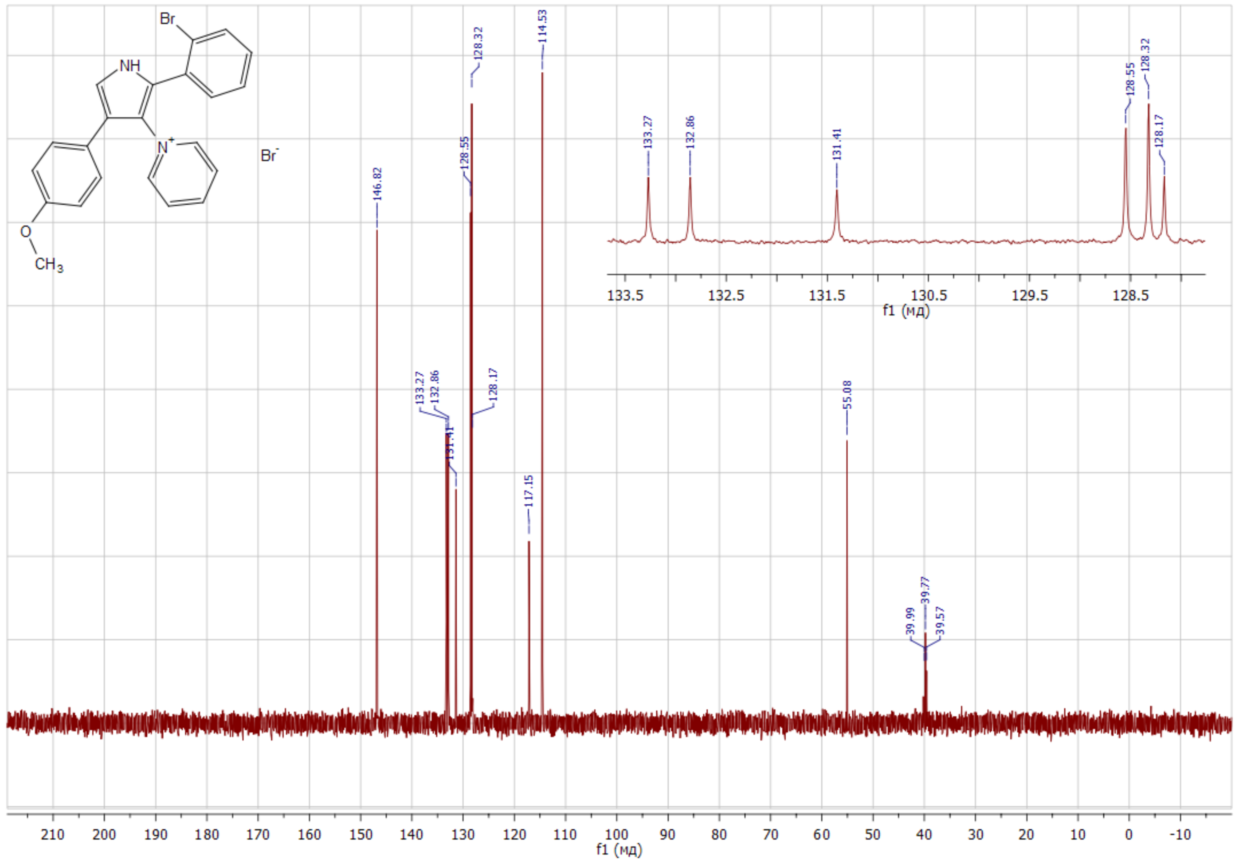

^{1}H NMR (400 MHz, DMSO-d_6) of 1-(2-(2-bromophenyl)-4-phenyl-1*H*-pyrrol-3-yl)-4-cyanopyridin-1-ium bromide (**16i**)

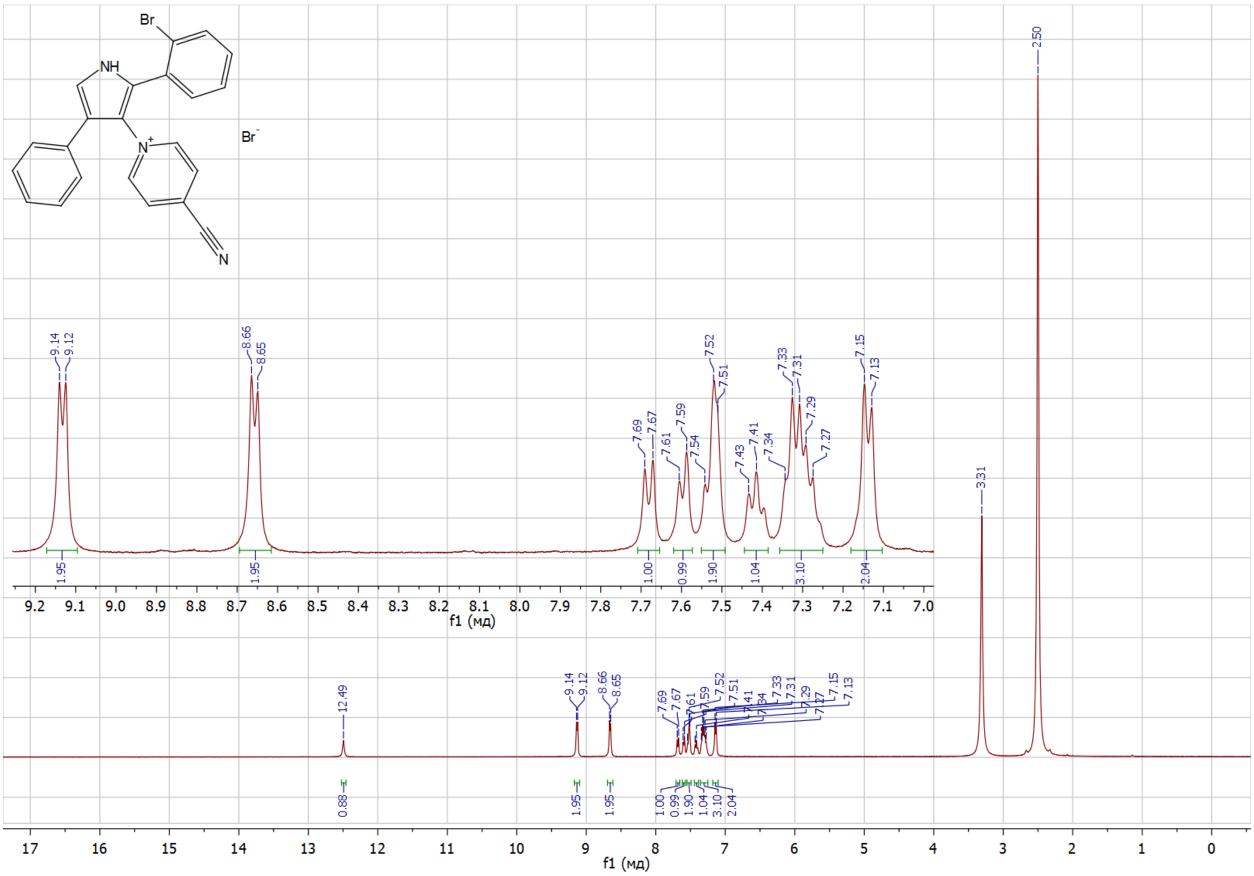

$^{13}C\{1H\}$ NMR (101 MHz, DMSO-d_6) of 1-(2-(2-bromophenyl)-4-phenyl-1*H*-pyrrol-3-yl)-4-cyanopyridin-1-ium bromide (**16i**)

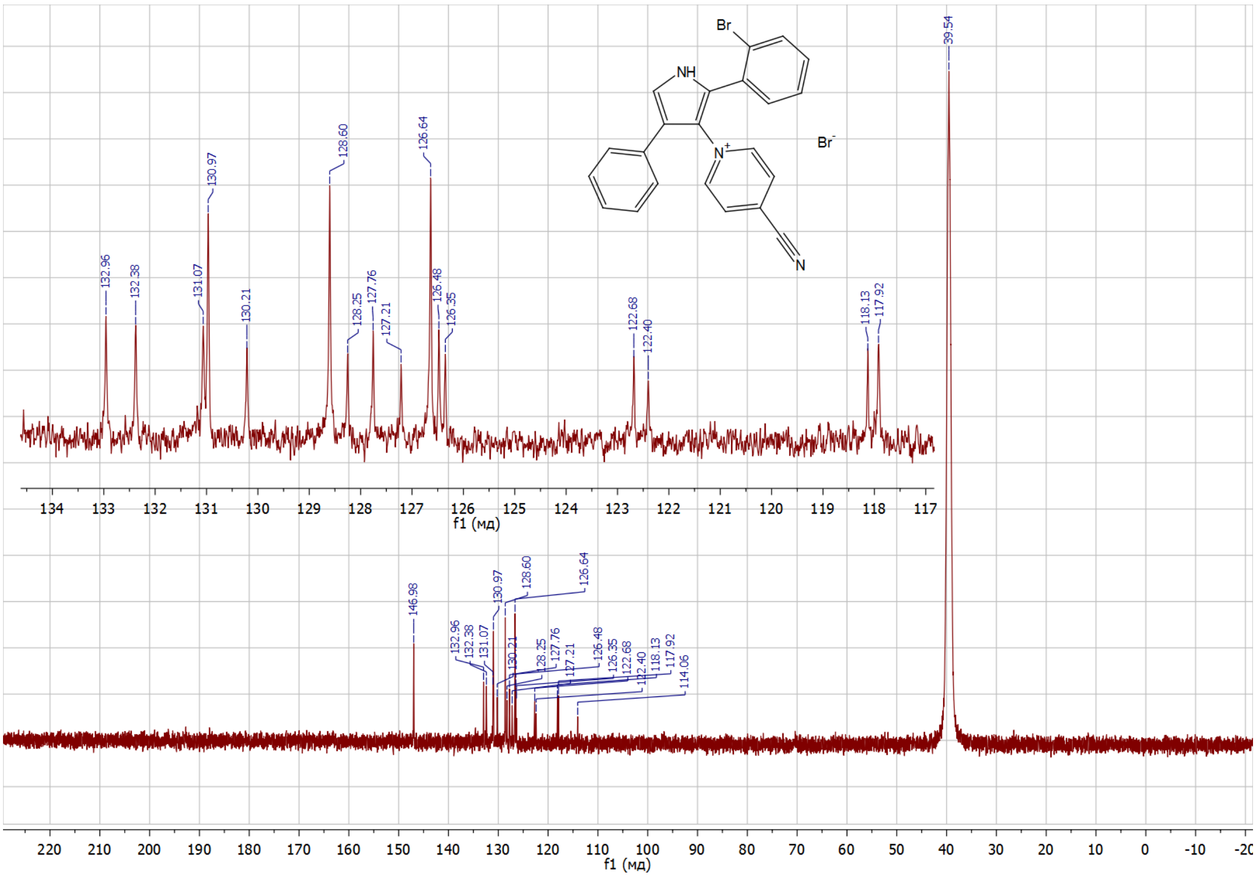

^{13}C DEPT135 NMR (101 MHz, DMSO-d_6) of 1-(2-(2-bromophenyl)-4-phenyl-1*H*-pyrrol-3-yl)-4-cyanopyridin-1-ium bromide (**16i**)

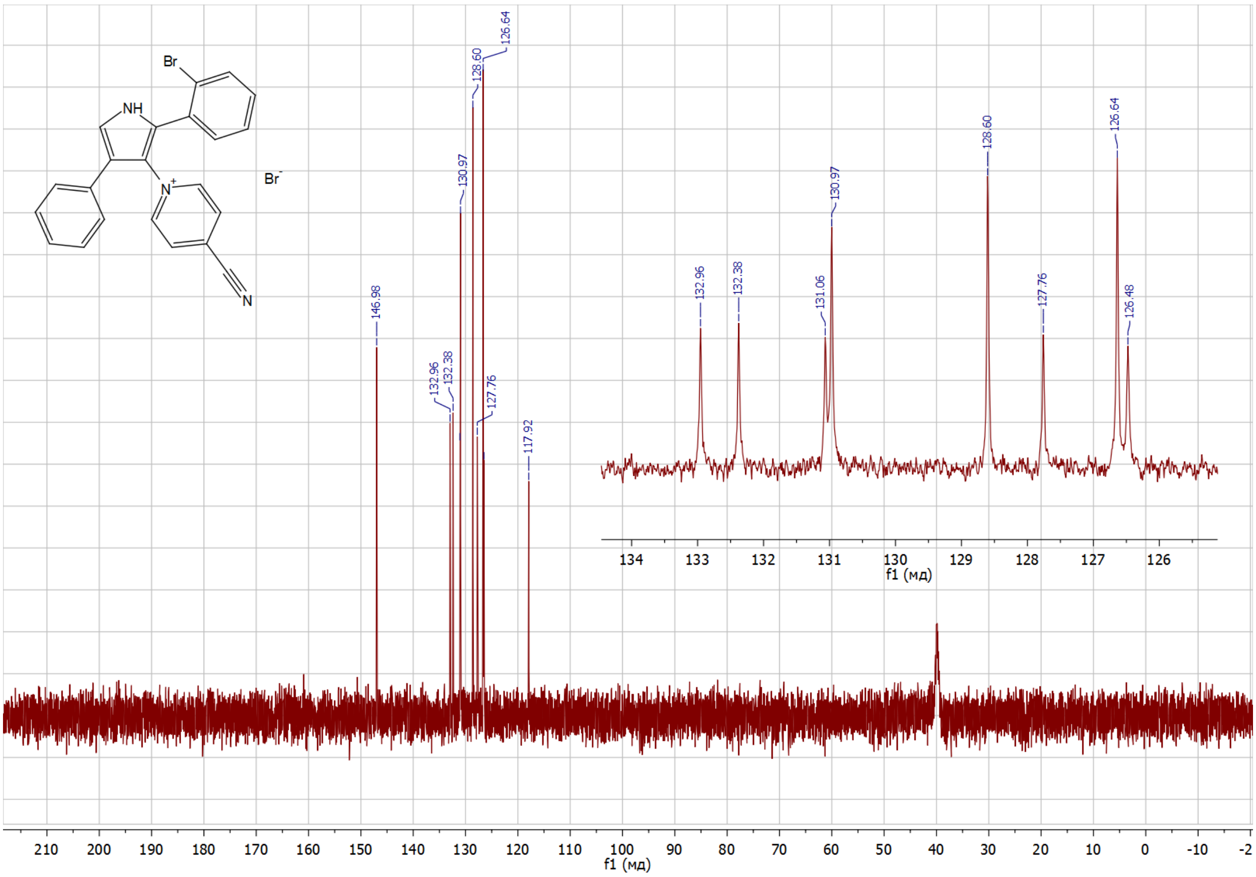

^{1}H NMR (400 MHz, DMSO-d_6) of 4-benzoyl-1-(2-(2-bromophenyl)-4-phenyl-1*H*-pyrrol-3-yl)pyridin-1-ium bromide (**16j**)

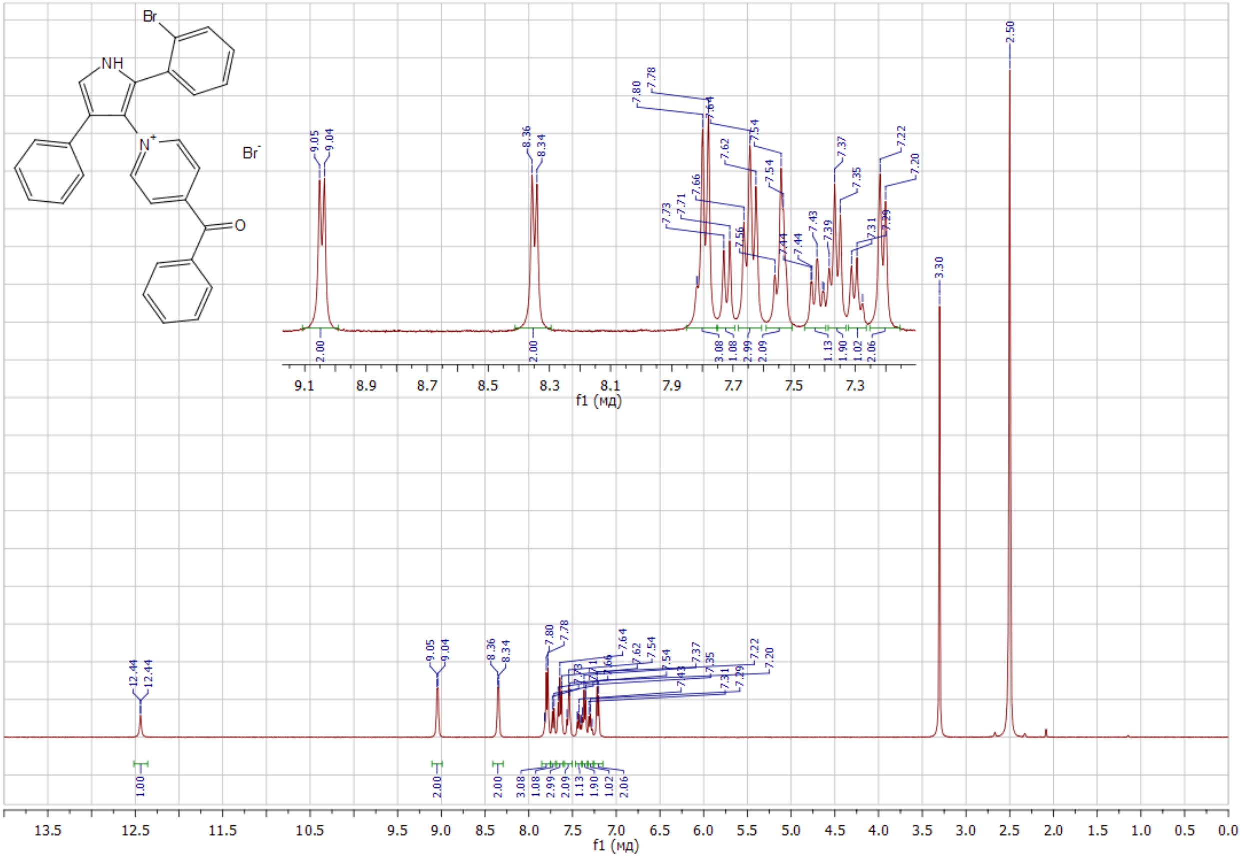

^{13}C{1H} NMR (101 MHz, DMSO-d_6) of 4-benzoyl-1-(2-(2-bromophenyl)-4-phenyl-1*H*-pyrrol-3-yl)pyridin-1-ium bromide (**16j**)

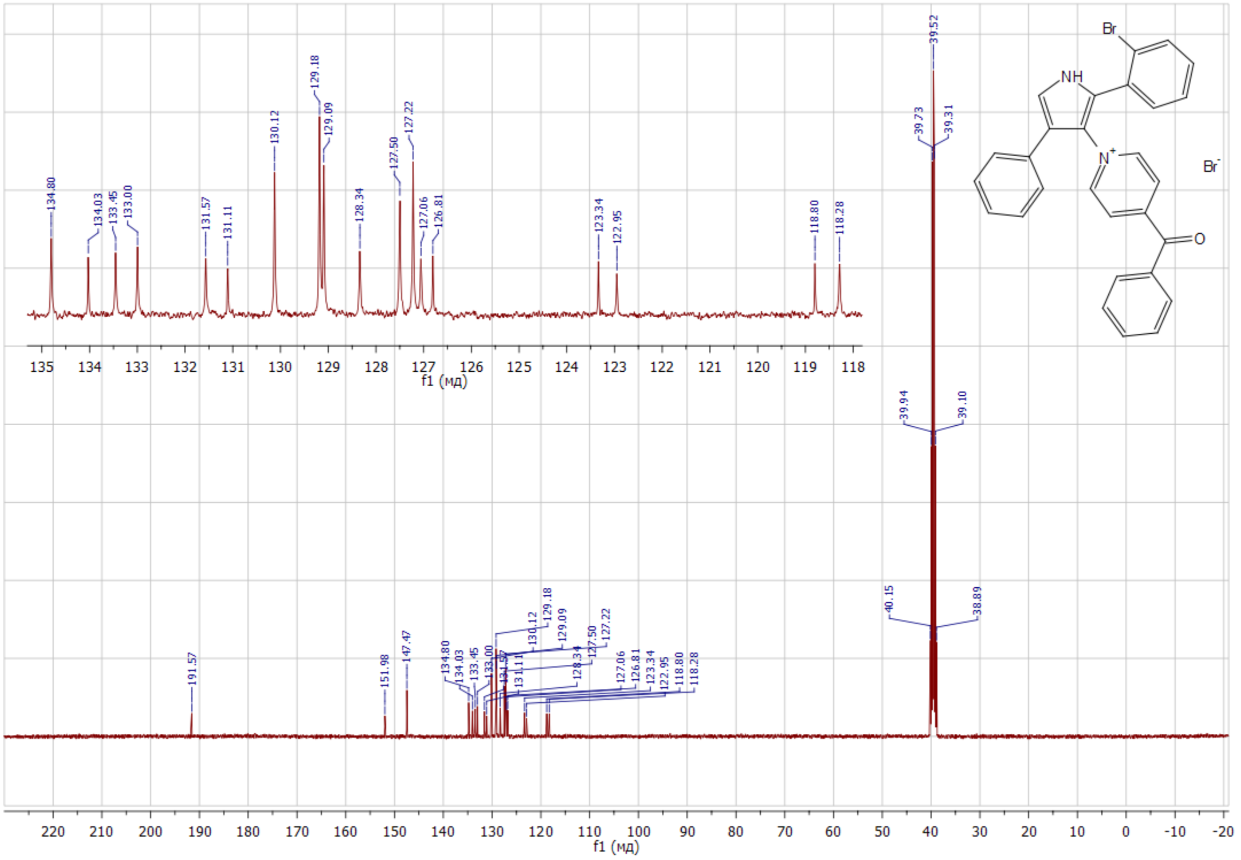

^{13}C DEPT135 NMR (101 MHz, DMSO-d_6) of 4-benzoyl-1-(2-(2-bromophenyl)-4-phenyl-1*H*-pyrrol-3-yl)pyridin-1-ium bromide (**16j**)

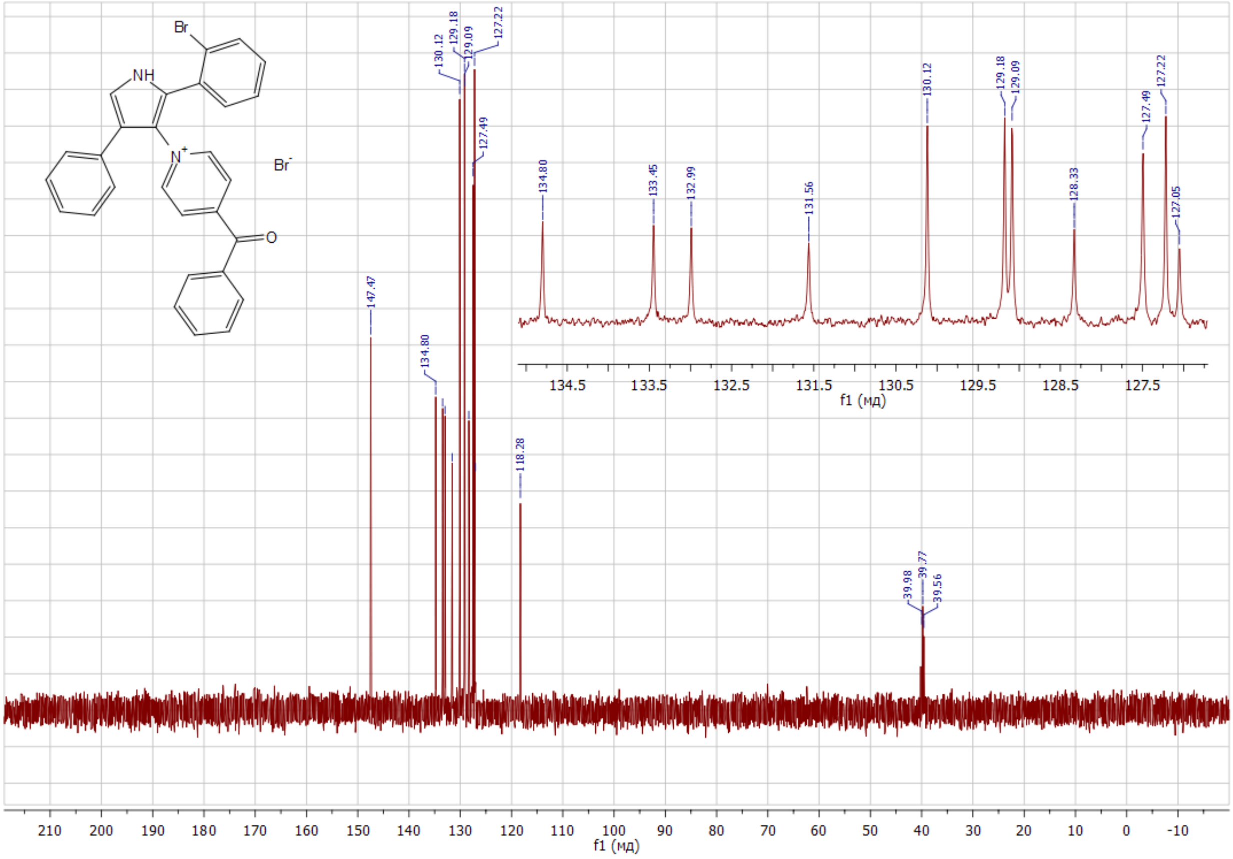

^{1}H NMR (400 MHz, DMSO-d_6) of 3-phenylpyrido[2,1-*a*]pyrrolo[3,2-*c*]isoquinolin-1-ium bromide (**17a**)

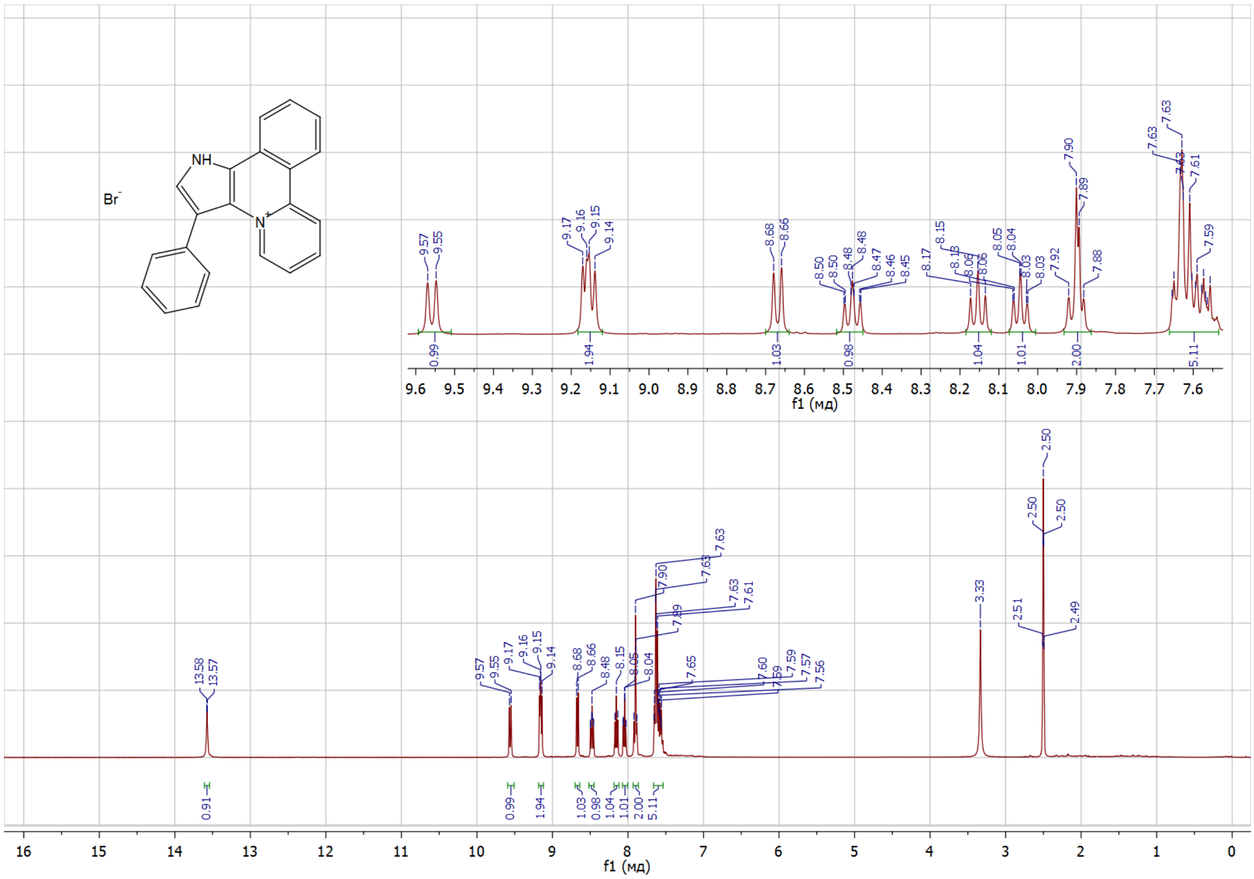

^{13}C{1H} NMR (101 MHz, DMSO-d_6) of 3-phenylpyrido[2,1-*a*]pyrrolo[3,2-*c*]isoquinolin-1-ium bromide (**17a**)

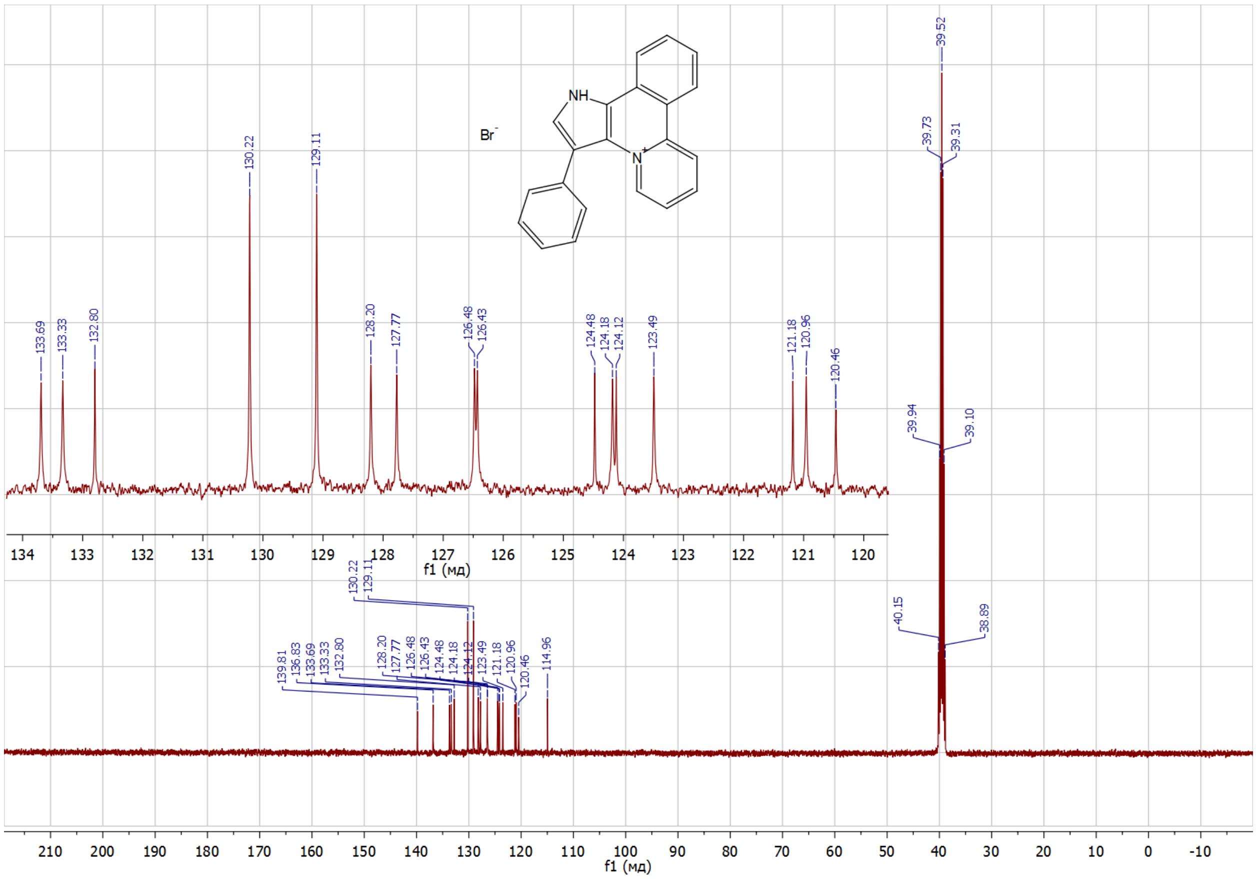

^{13}C DEPT135 NMR (101 MHz, DMSO-d_6) of 3-phenylpyrido[2,1-*a*]pyrrolo[3,2-*c*]isoquinolin-1-ium bromide (**17a**)

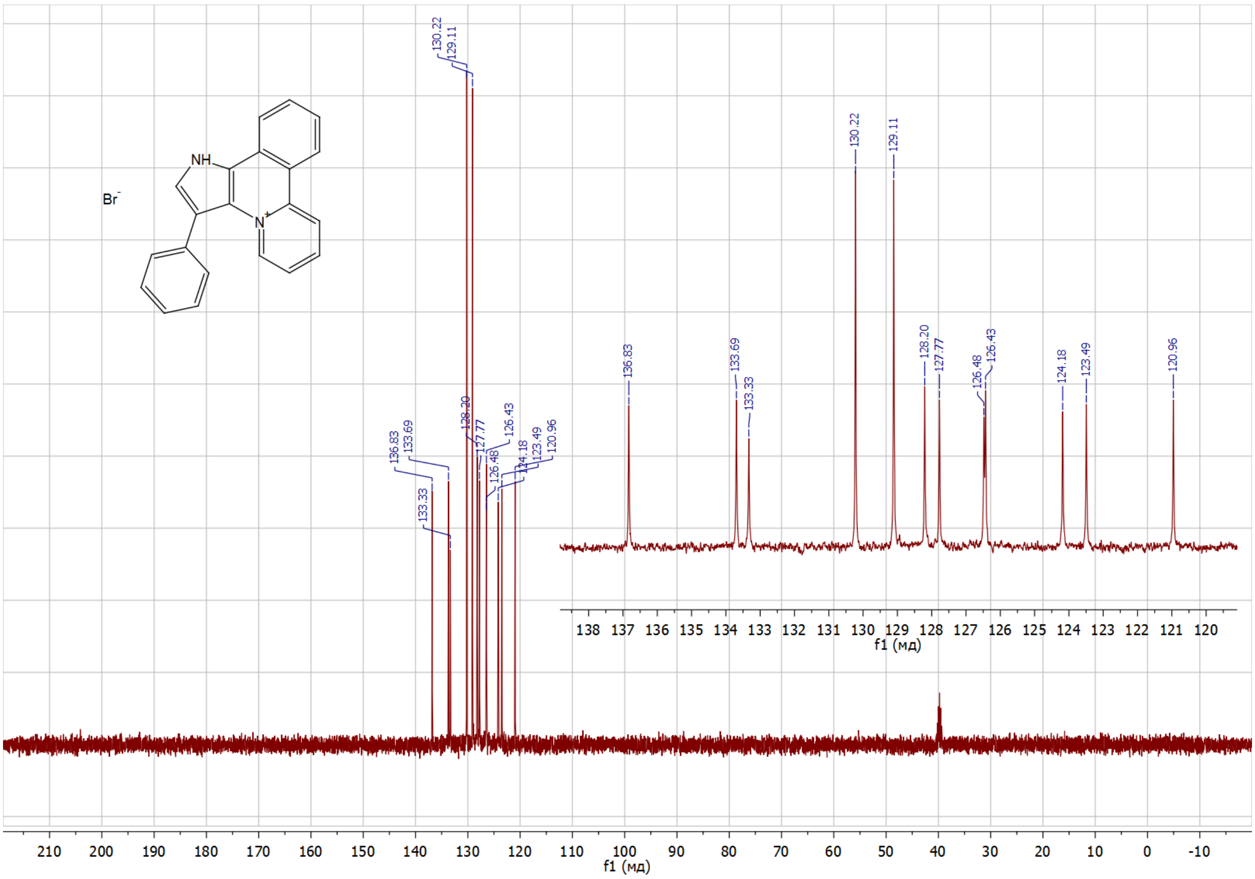

^{1}H NMR (400 MHz, DMSO-d_6) of 7-methyl-3-phenyl-1*H*-pyrido[2,1-*a*]pyrrolo[3,2-*c*]isoquinolin-4-ium bromide (**17b**)

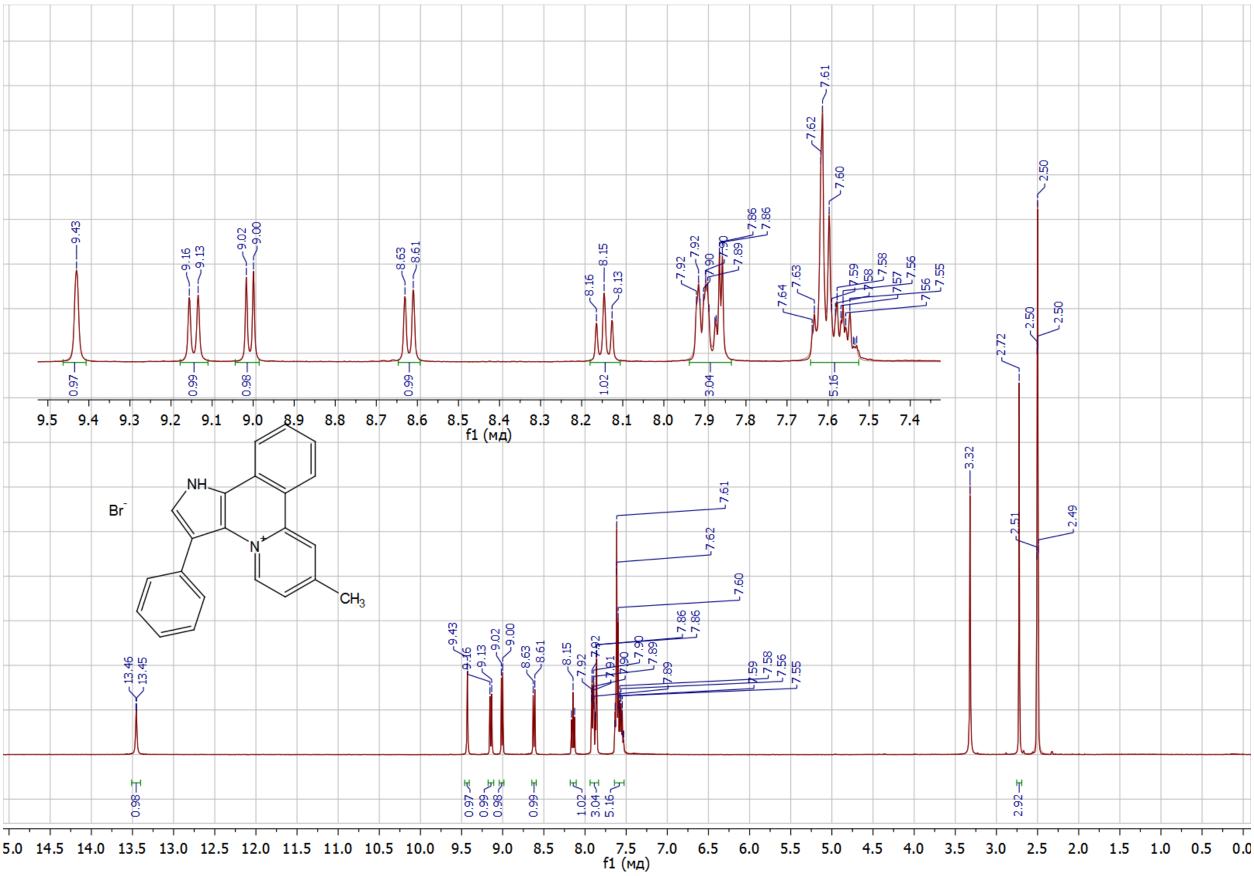

$^{13}C\{1H\}$ NMR (101 MHz, DMSO-d_6) of 7-methyl-3-phenyl-1*H*-pyrido[2,1-*a*]pyrrolo[3,2-*c*]isoquinolin-4-ium bromide (**17b**)

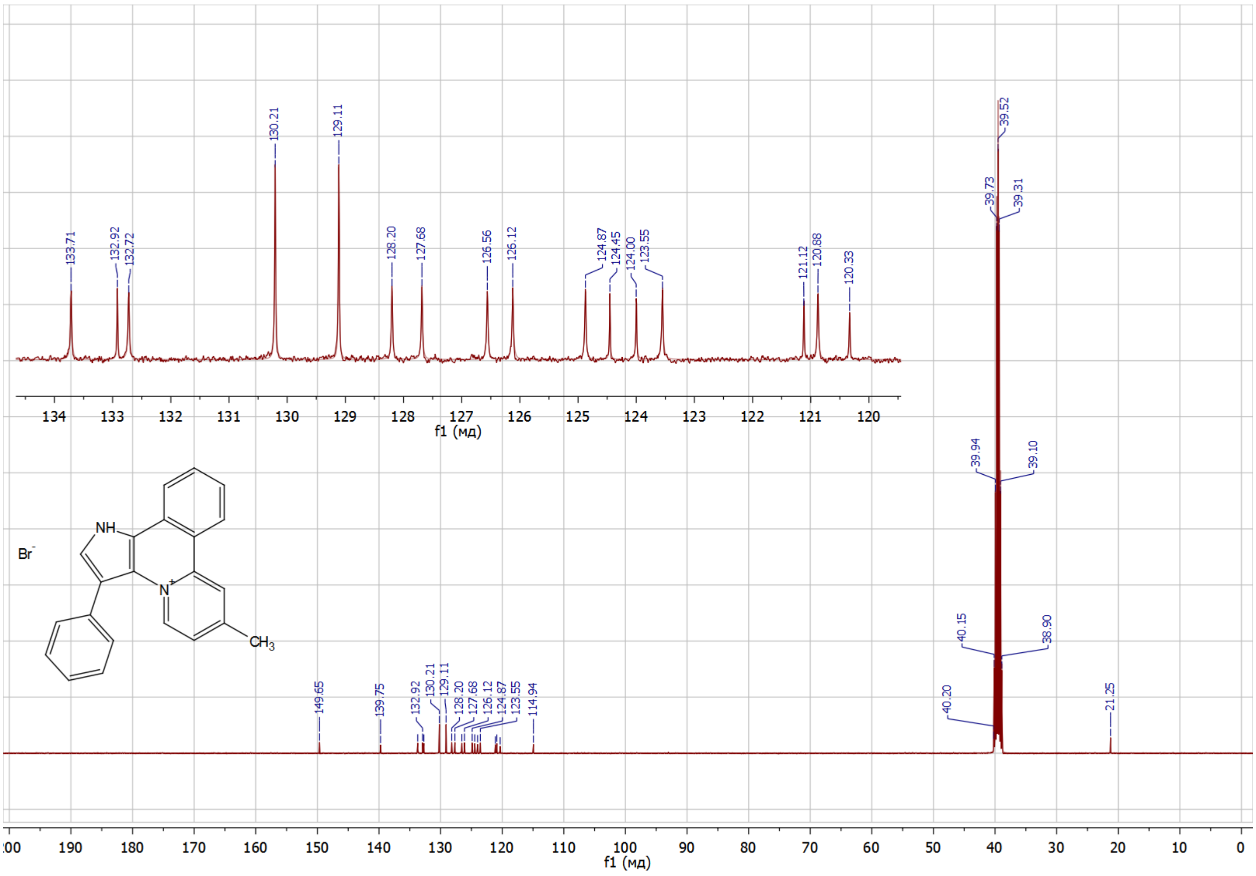

^{13}C DEPT135 NMR (101 MHz, DMSO-d_6) of 7-methyl-3-phenyl-1*H*-pyrido[2,1-*a*]pyrrolo[3,2-*c*]isoquinolin-4-ium bromide (**17b**)

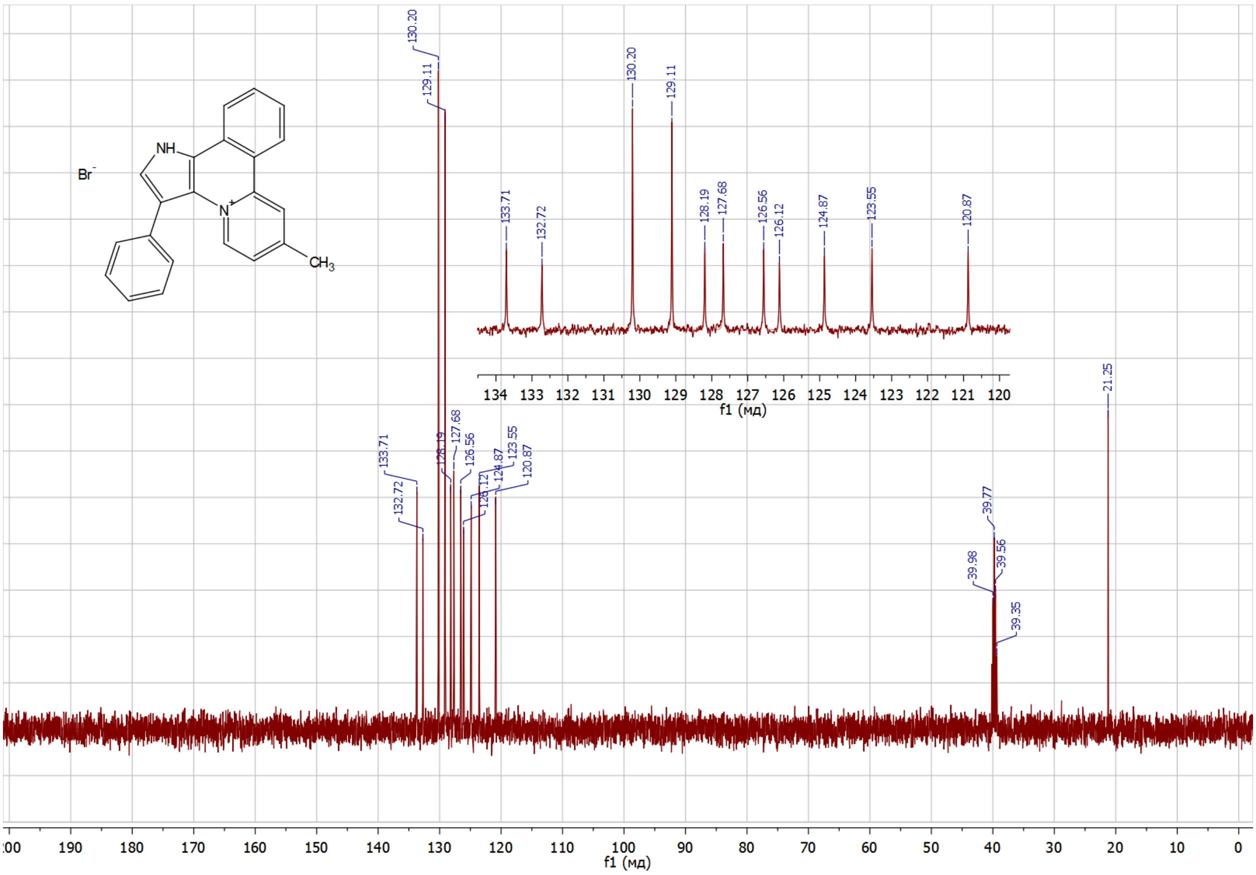

^{1}H NMR (400 MHz, DMSO-d_6) of 7-methoxy-3-phenyl-1*H*-pyrido[2,1-*a*]pyrrolo[3,2-*c*]isoquinolin-4-ium bromide (**17c**)

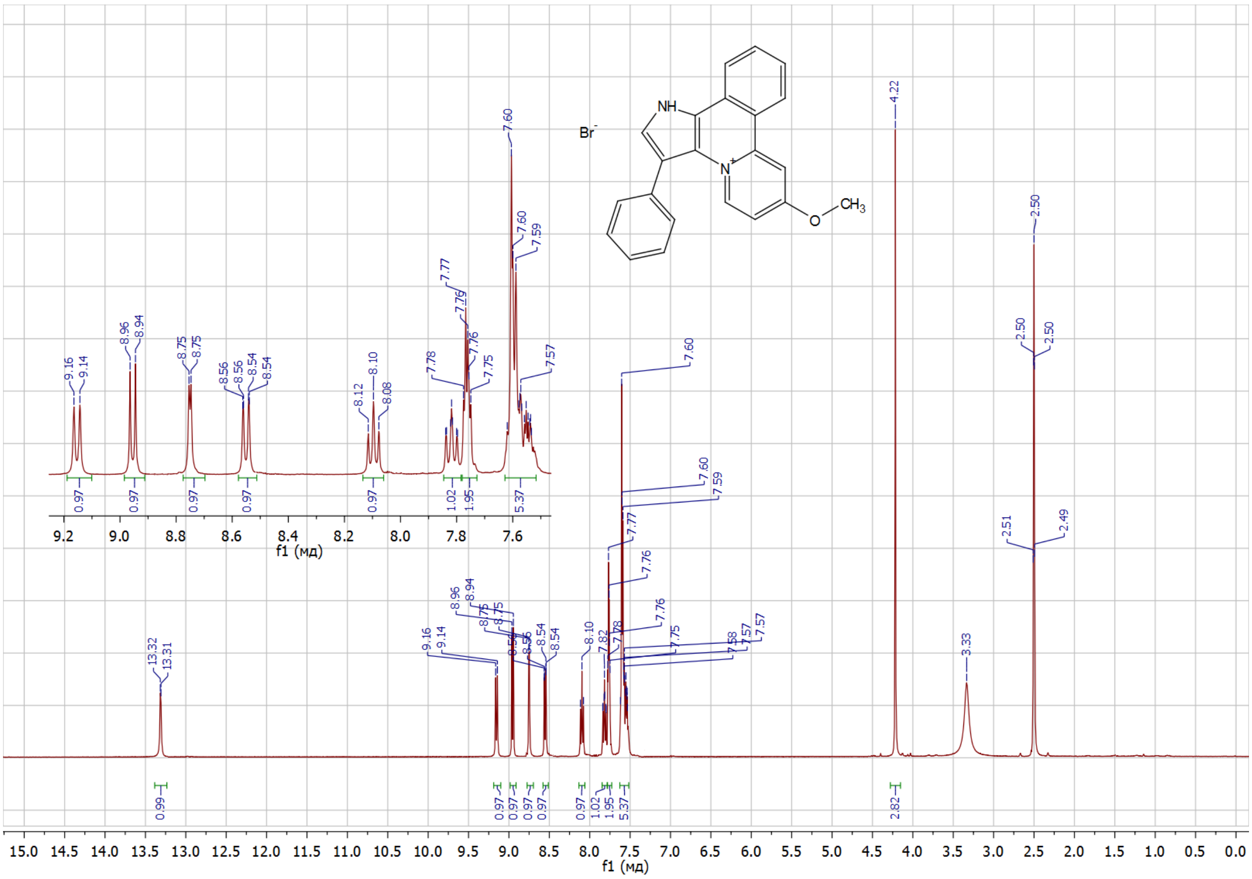

$^{13}C\{1H\}$ NMR (101 MHz, DMSO-d_6) of 7-methoxy-3-phenyl-1*H*-pyrido[2,1-*a*]pyrrolo[3,2-*c*]isoquinolin-4-ium bromide (**17c**)

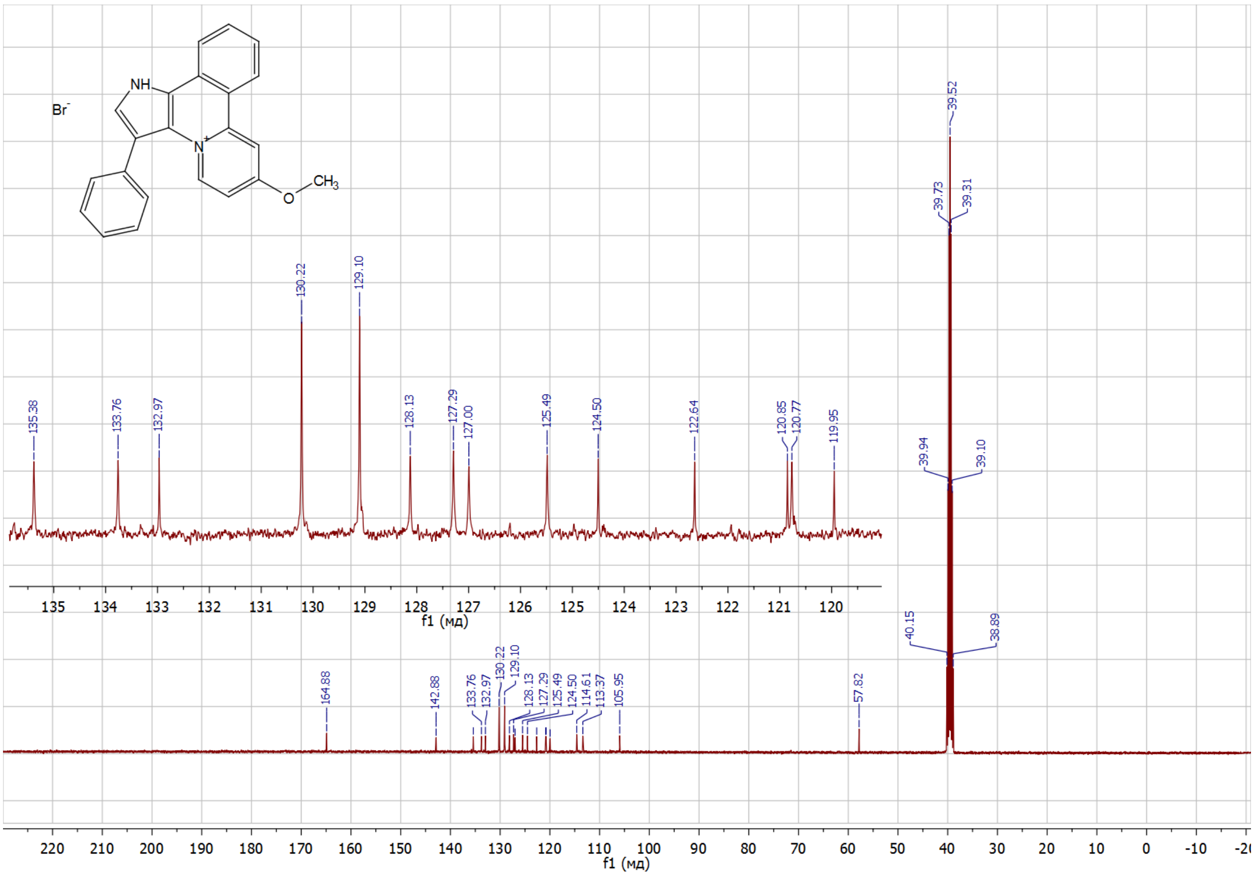

^{13}C DEPT135 NMR (101 MHz, DMSO-d_6) of 7-methoxy-3-phenyl-1*H*-pyrido[2,1-*a*]pyrrolo[3,2-*c*]isoquinolin-4-ium bromide (**17c**)

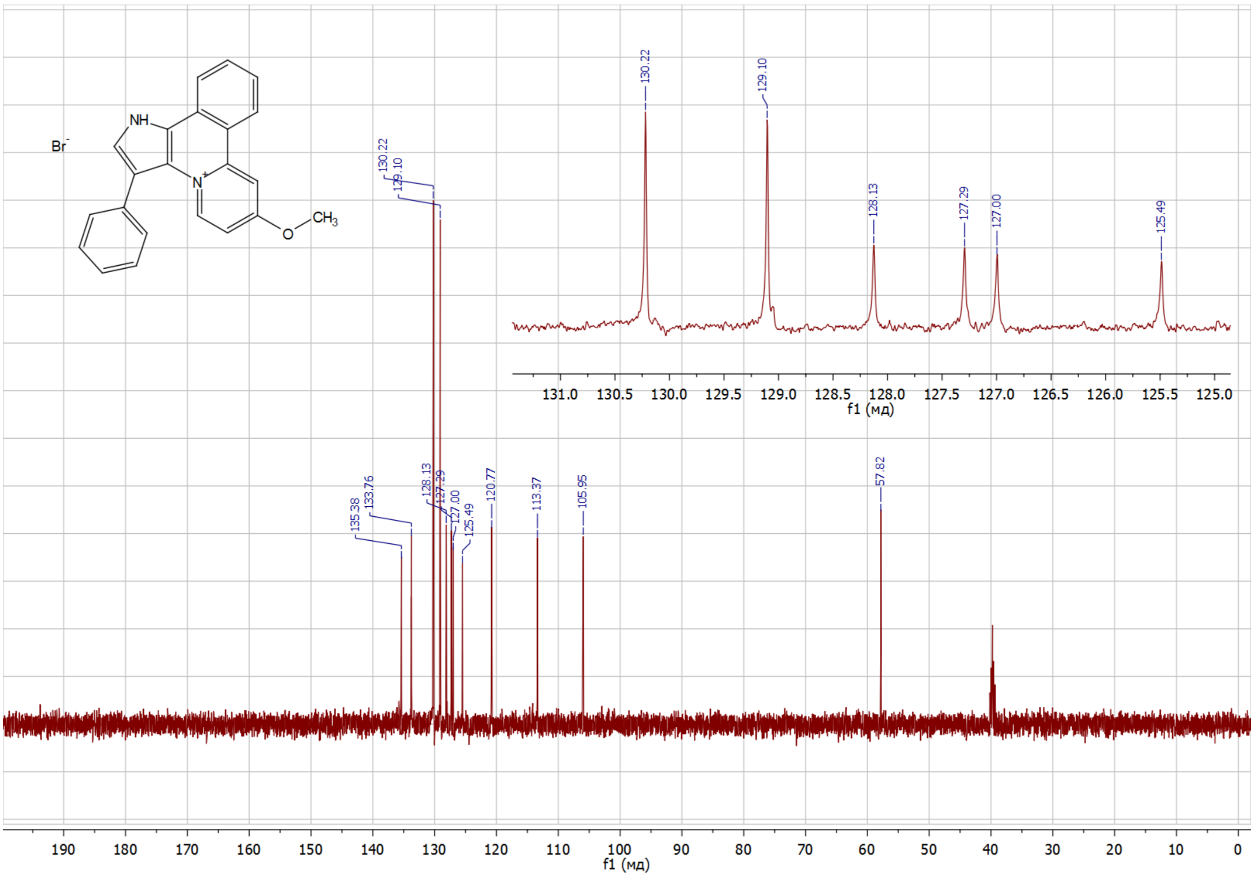

^{1}H NMR (400 MHz, DMSO-d_6) of 3,7-diphenylpyrido[2,1-*a*]pyrrolo[3,2-*c*]isoquinolin-1-ium bromide (**17d**)

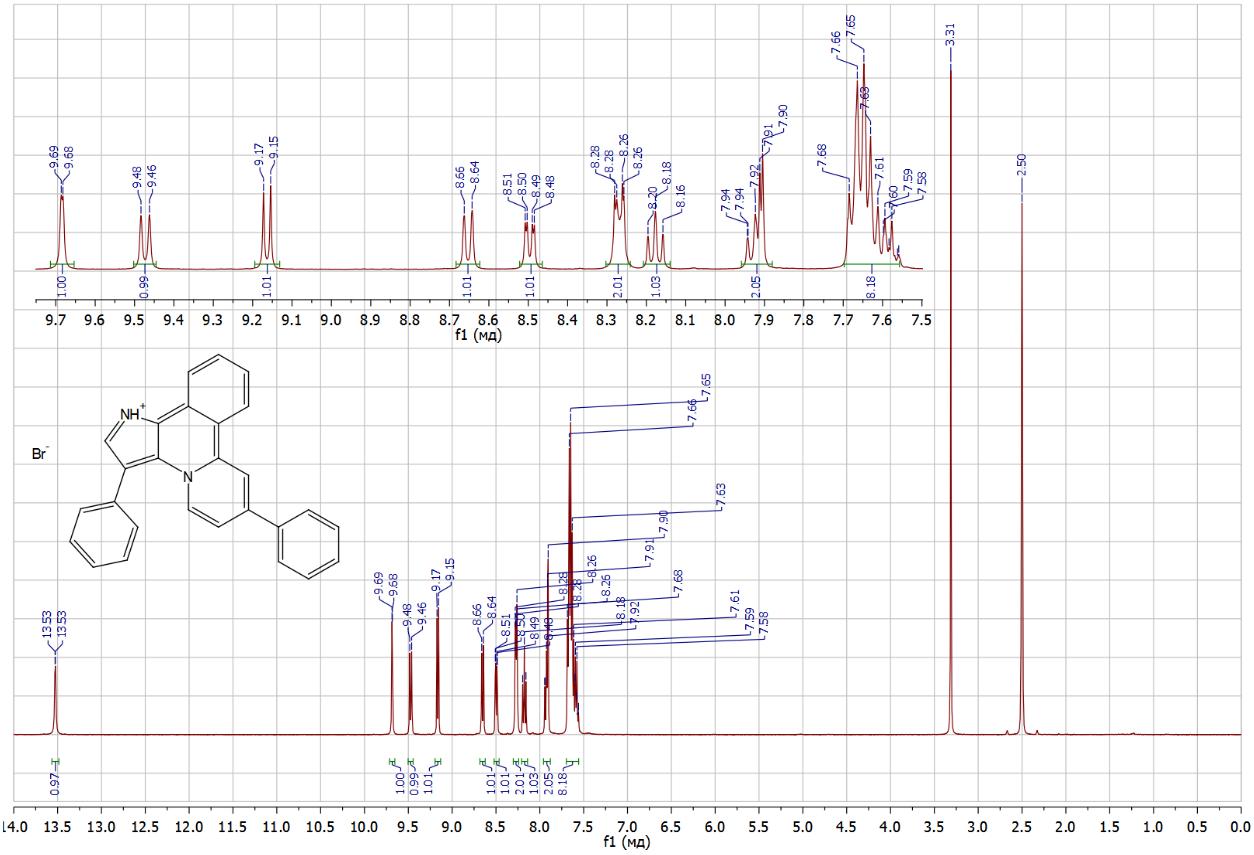

^{13}C{1H} NMR (101 MHz, DMSO-d_6) of 3,7-diphenylpyrido[2,1-*a*]pyrrolo[3,2-*c*]isoquinolin-1-ium bromide (**17d**)

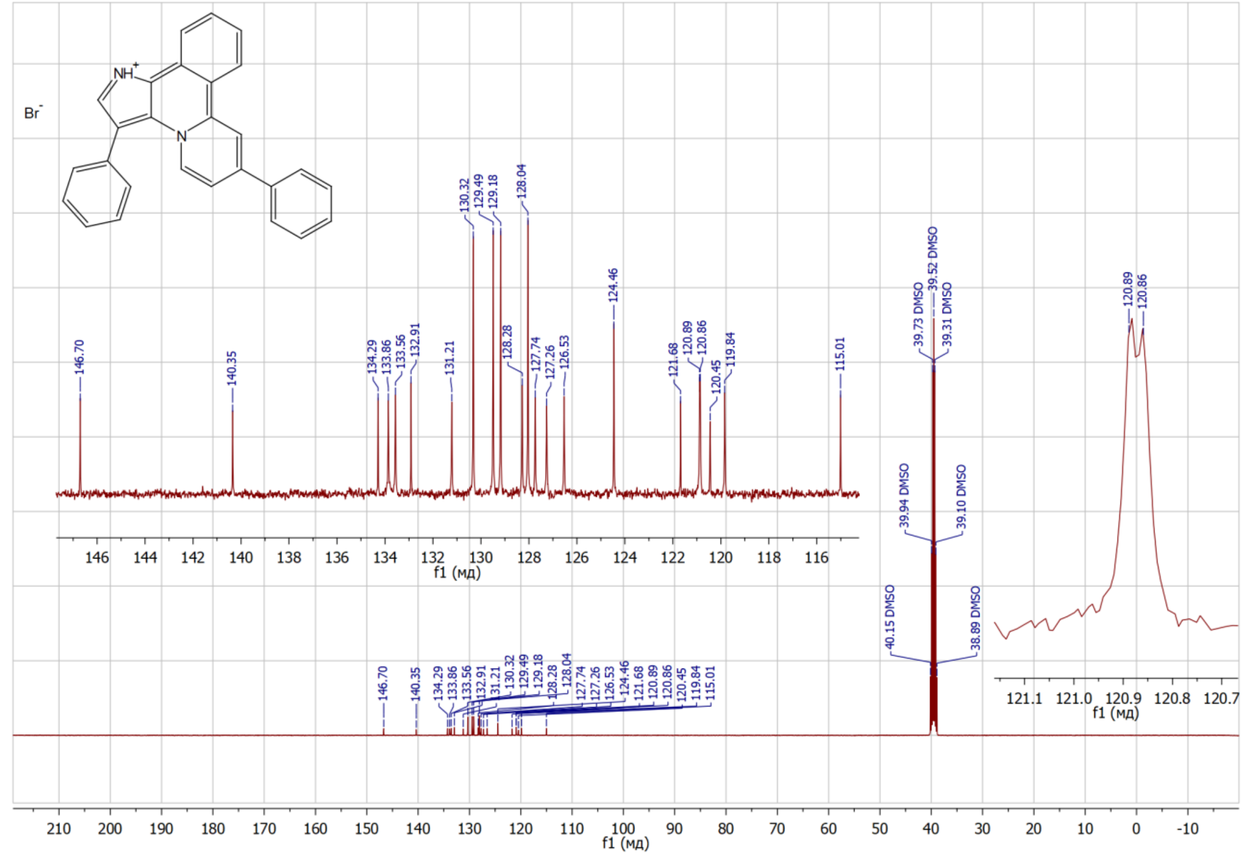

^{13}C DEPT135 NMR (101 MHz, DMSO-d_6) of 3,7-diphenylpyrido[2,1-*a*]pyrrolo[3,2-*c*]isoquinolin-1-ium bromide (**17d**)

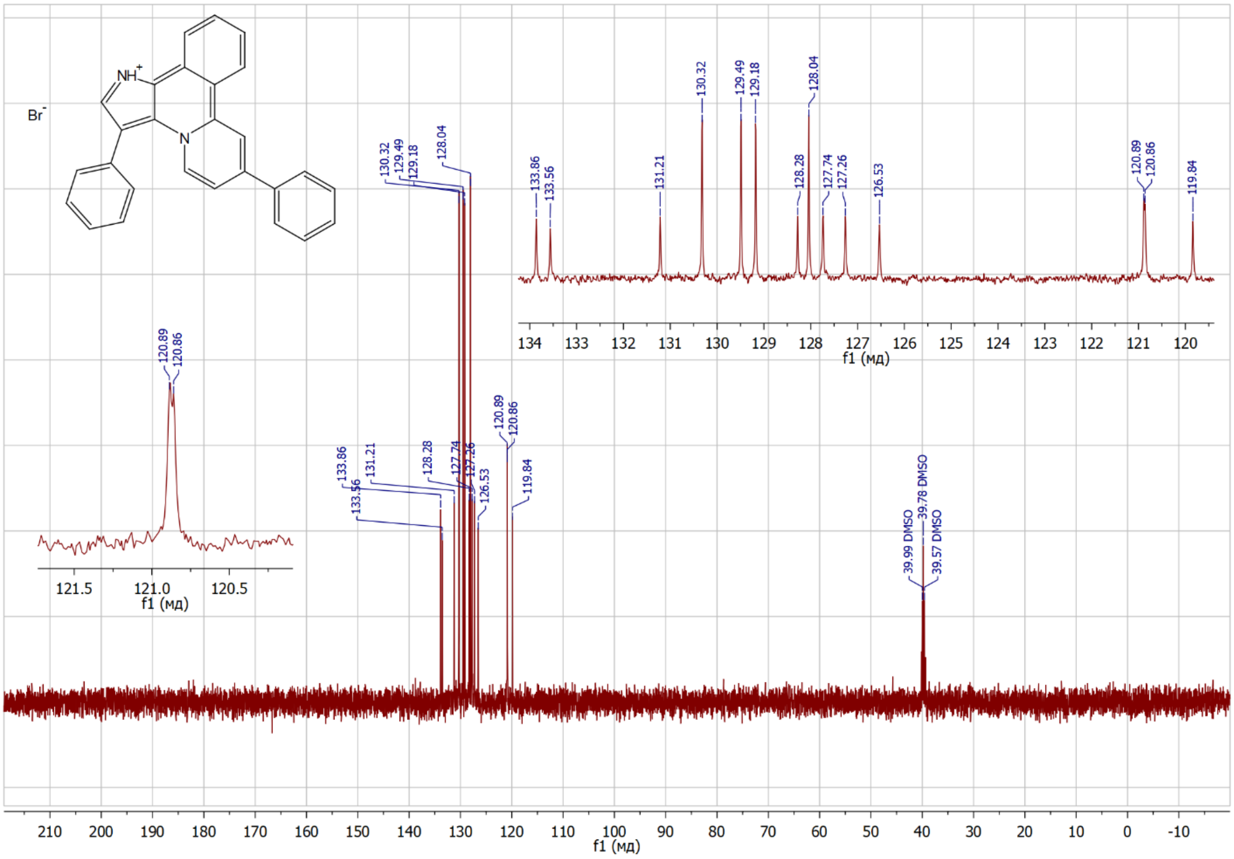

^{1}H NMR (400 MHz, DMSO-d_6) of 7-(4-methoxyphenyl)-3-phenyl-1*H*-pyrido[2,1-*a*]pyrrolo[3,2-*c*]isoquinolin-4-ium bromide (**17e**)

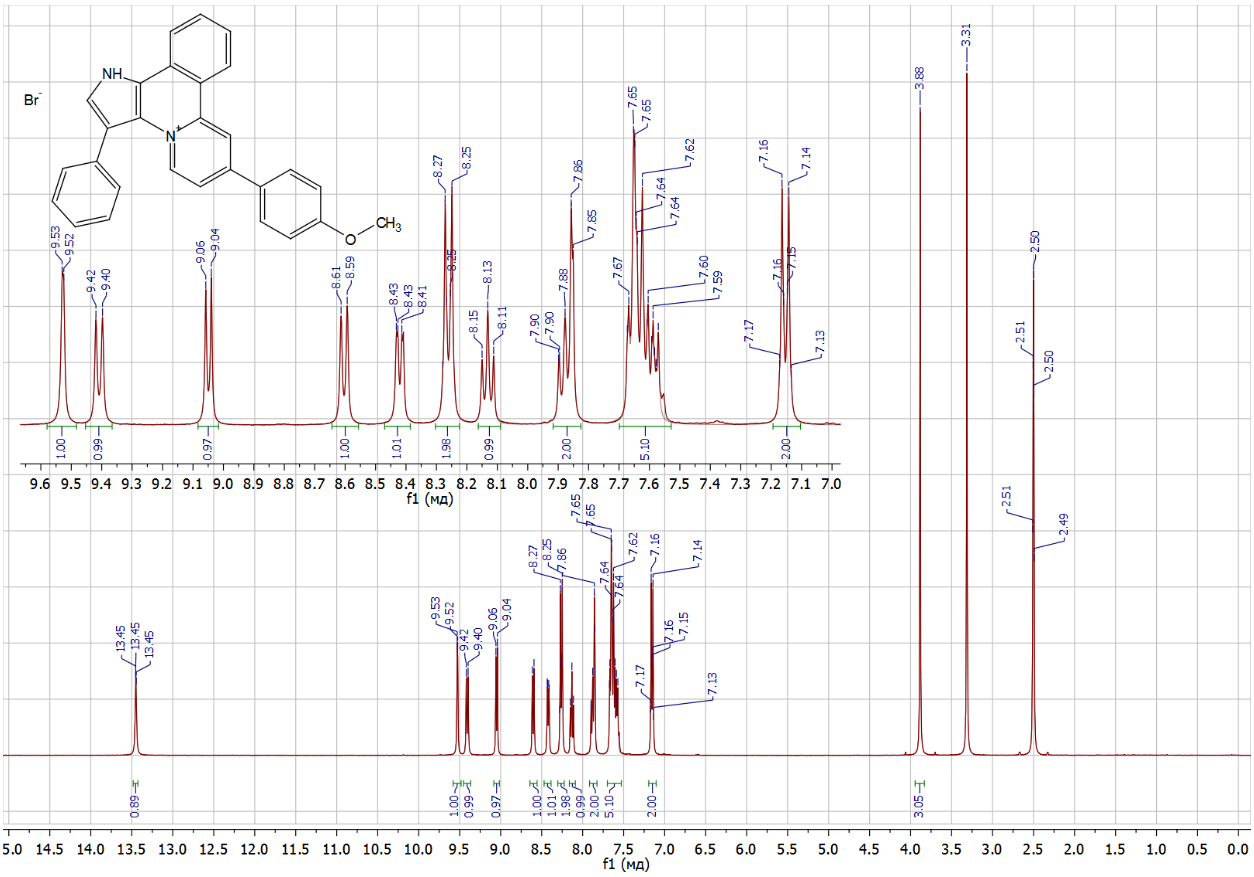

^{13}C{1H} NMR (101 MHz, DMSO-d_6) of 7-(4-methoxyphenyl)-3-phenyl-1*H*-pyrido[2,1-*a*]pyrrolo[3,2-*c*]isoquinolin-4-ium bromide (**17e**)

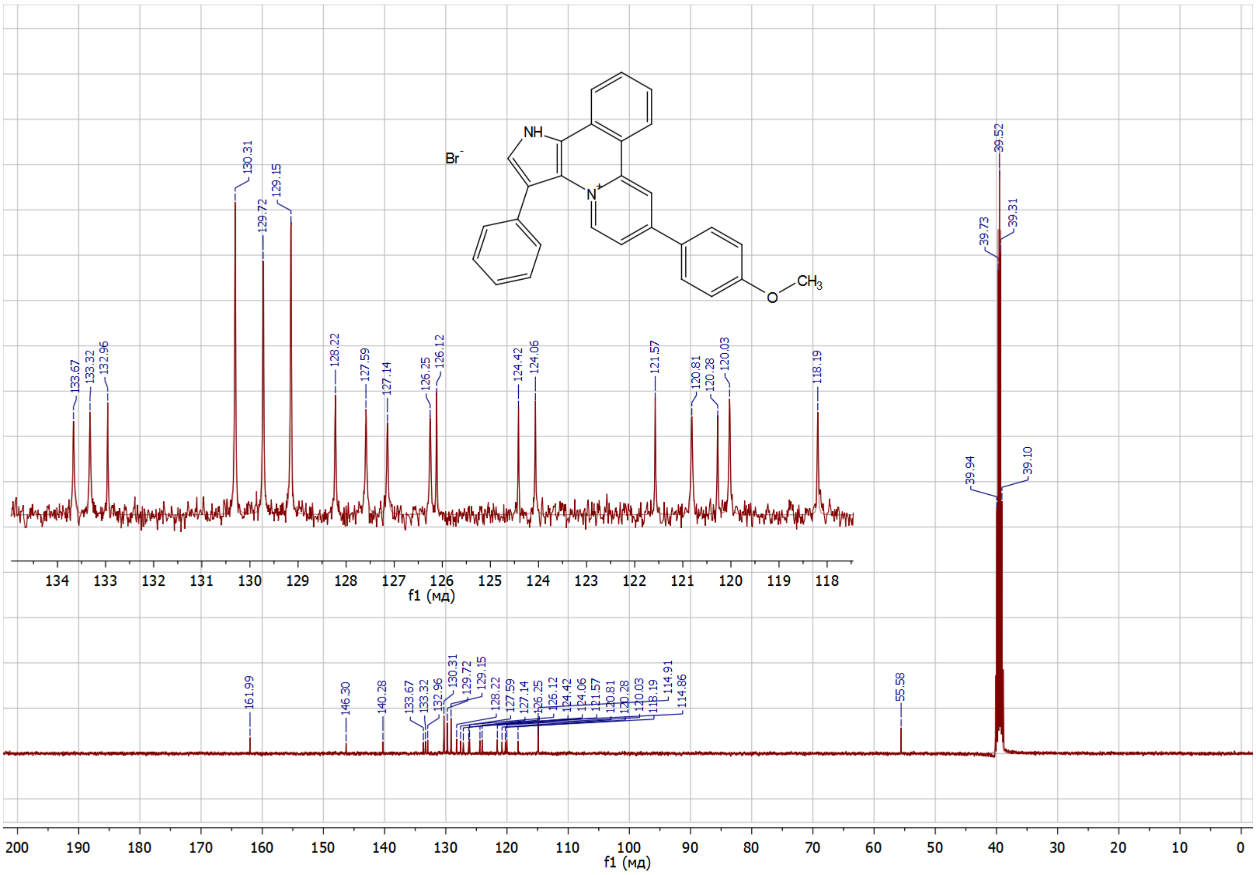

^{13}C DEPT135 NMR (101 MHz, DMSO-d_6) of 7-(4-methoxyphenyl)-3-phenyl-1*H*-pyrido[2,1-*a*]pyrrolo[3,2-*c*]isoquinolin-4-ium bromide (**17e**)

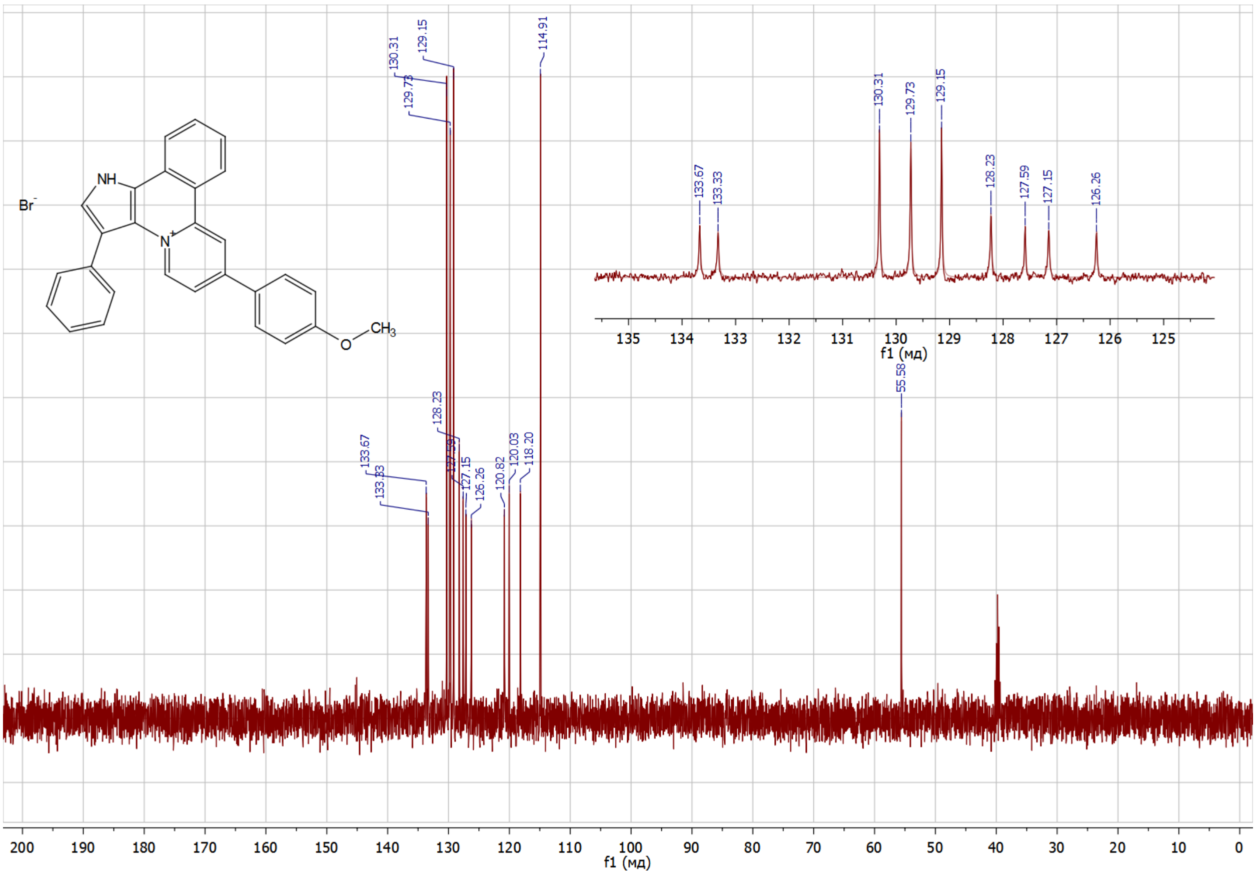

^{1}H NMR (400 MHz, DMSO-d_6) of 6,8-dimethyl-3-phenylpyrido[2,1-*a*]pyrrolo[3,2-*c*]isoquinolin-1-ium bromide (**17f**)

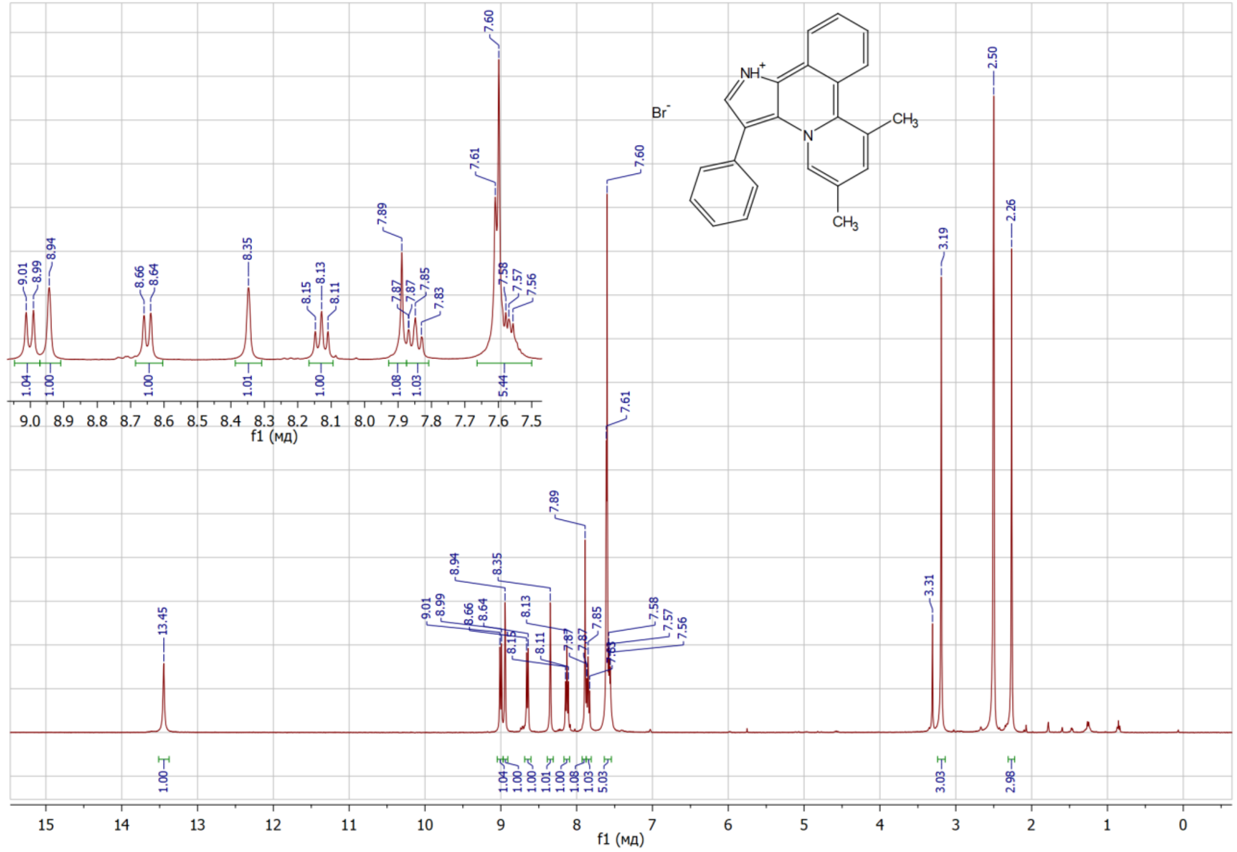

^{13}C{1H} NMR (101 MHz, DMSO-d_6) of 6,8-dimethyl-3-phenylpyrido[2,1-*a*]pyrrolo[3,2-*c*]isoquinolin-1-ium bromide (**17f**)

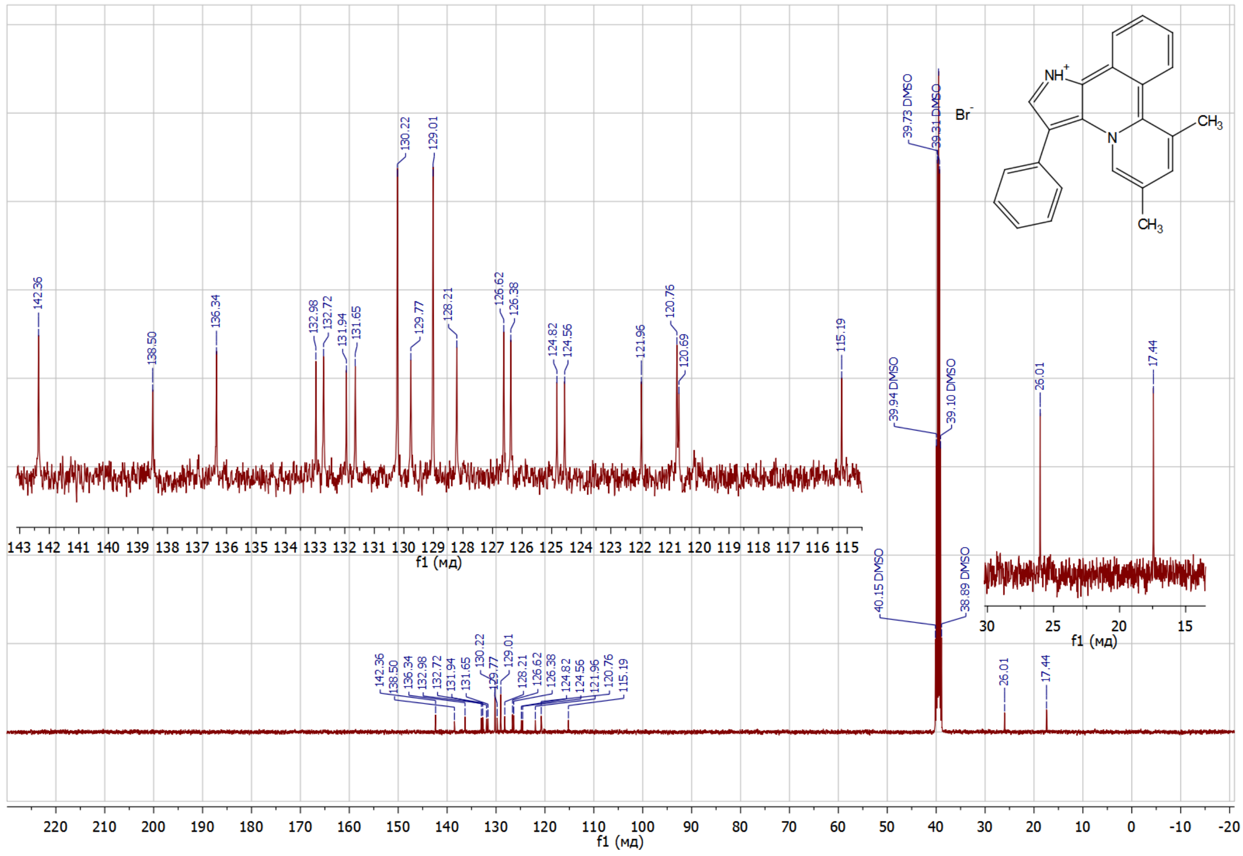

^{13}C DEPT135 NMR (101 MHz, DMSO-d_6) of 6,8-dimethyl-3-phenylpyrido[2,1-*a*]pyrrolo[3,2-*c*]isoquinolin-1-ium bromide (**17f**)

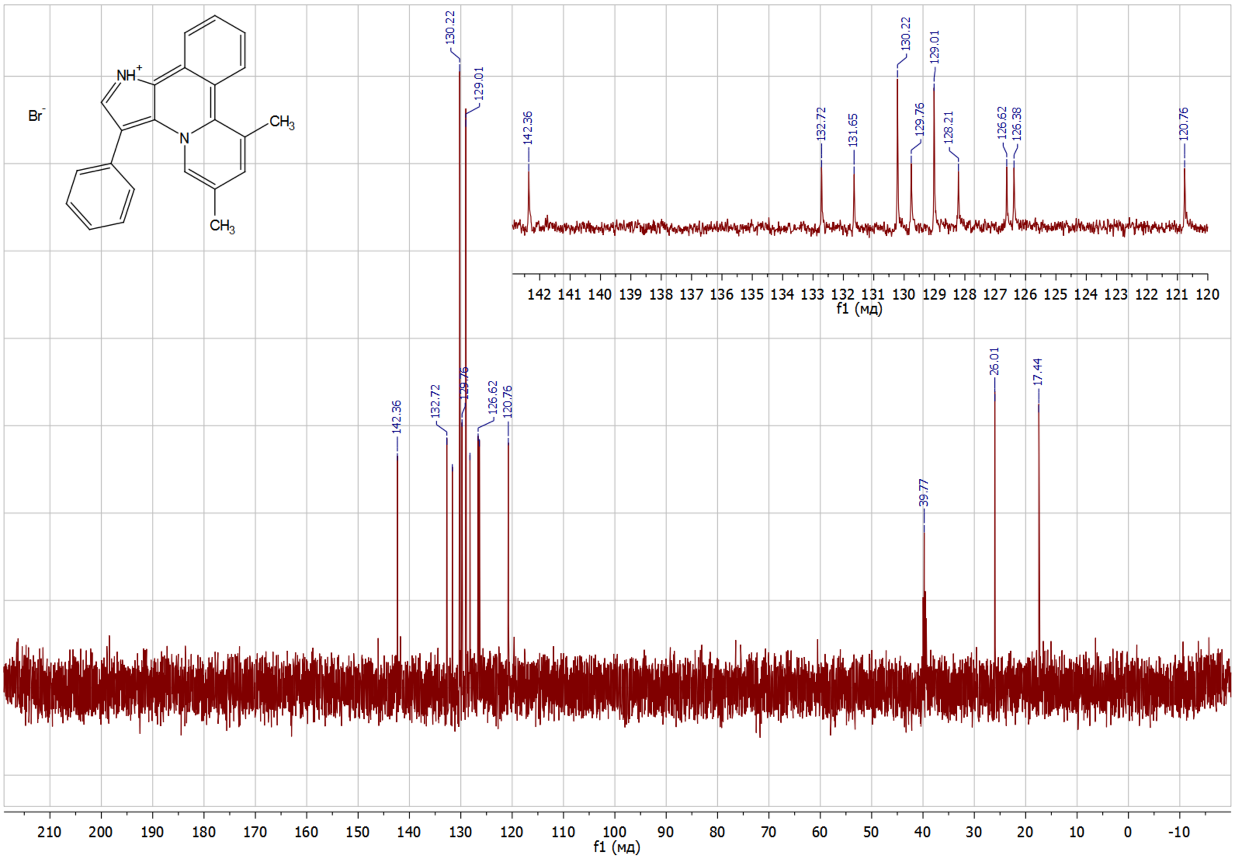

^{1}H NMR (400 MHz, DMSO-d_6) of 3-phenyl-1*H*-isoquinolino[1,2-*a*]pyrrolo[3,2-*c*]isoquinolin-4-ium bromide (**17g**)

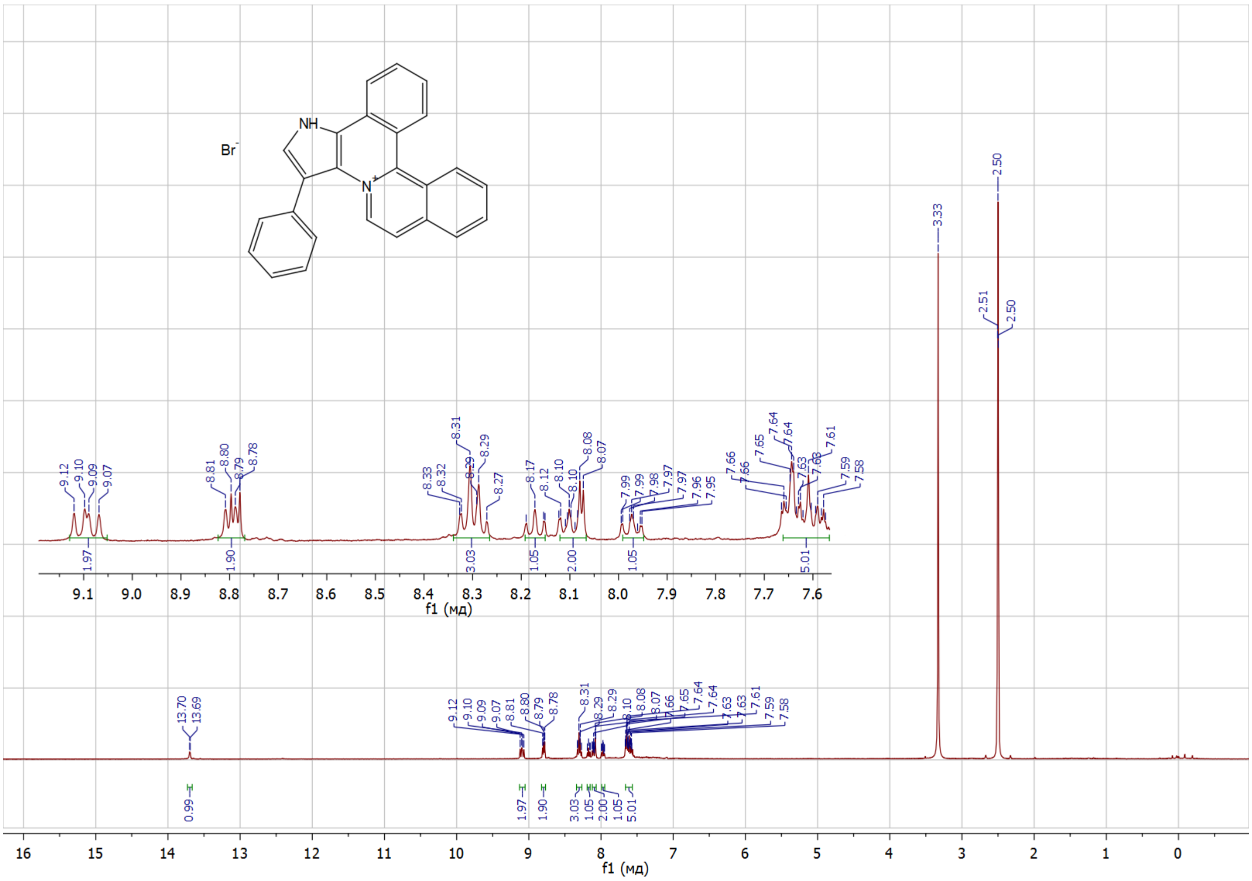

$^{13}C\{^{1}H\}$ NMR (101 MHz, DMSO-d_6) of 3-phenyl-1*H*-isoquinolino[1,2-*a*]pyrrolo[3,2-*c*]isoquinolin-4-ium bromide (**17g**)

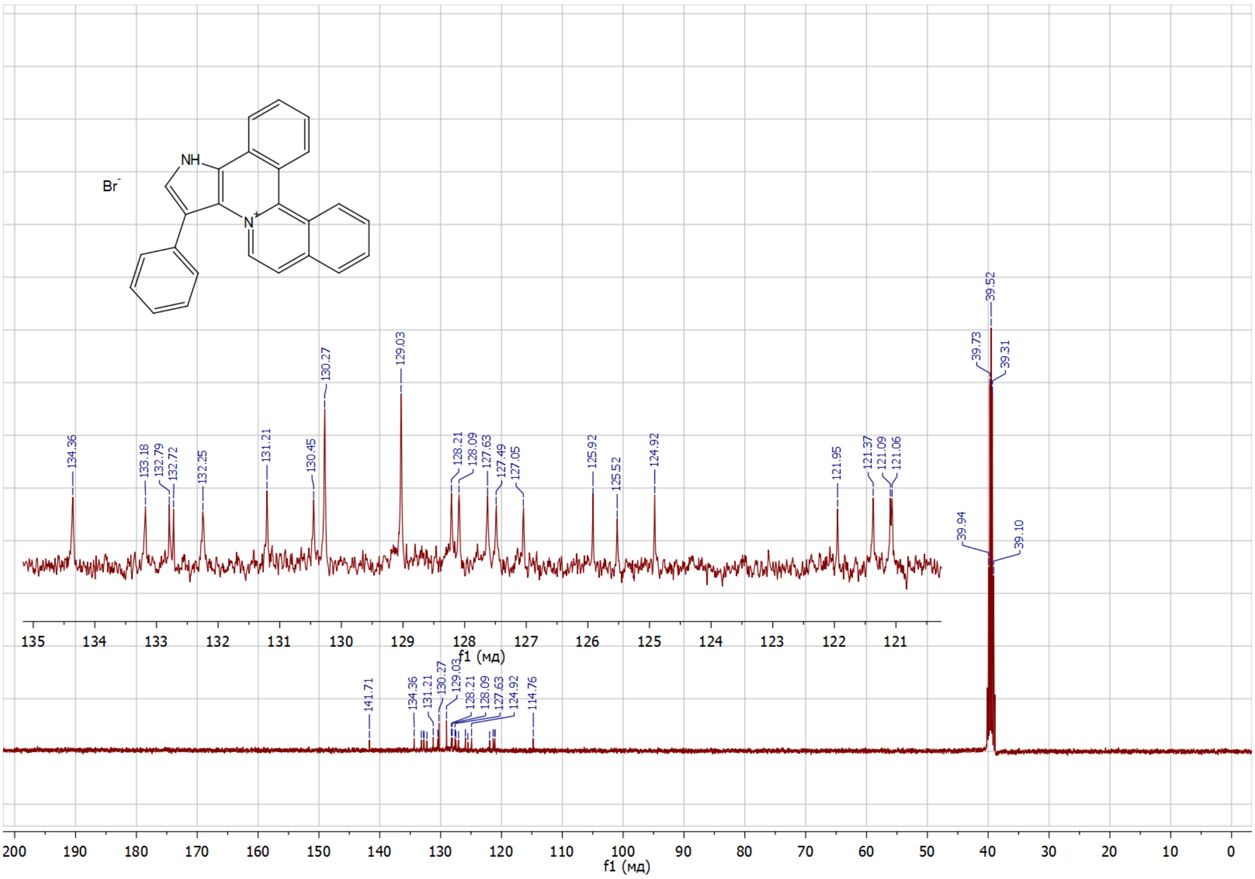

^{13}C DEPT135 NMR (101 MHz, DMSO-d_6) of 3-phenyl-1*H*-isoquinolino[1,2-*a*]pyrrolo[3,2-*c*]isoquinolin-4-ium bromide (**17g**)

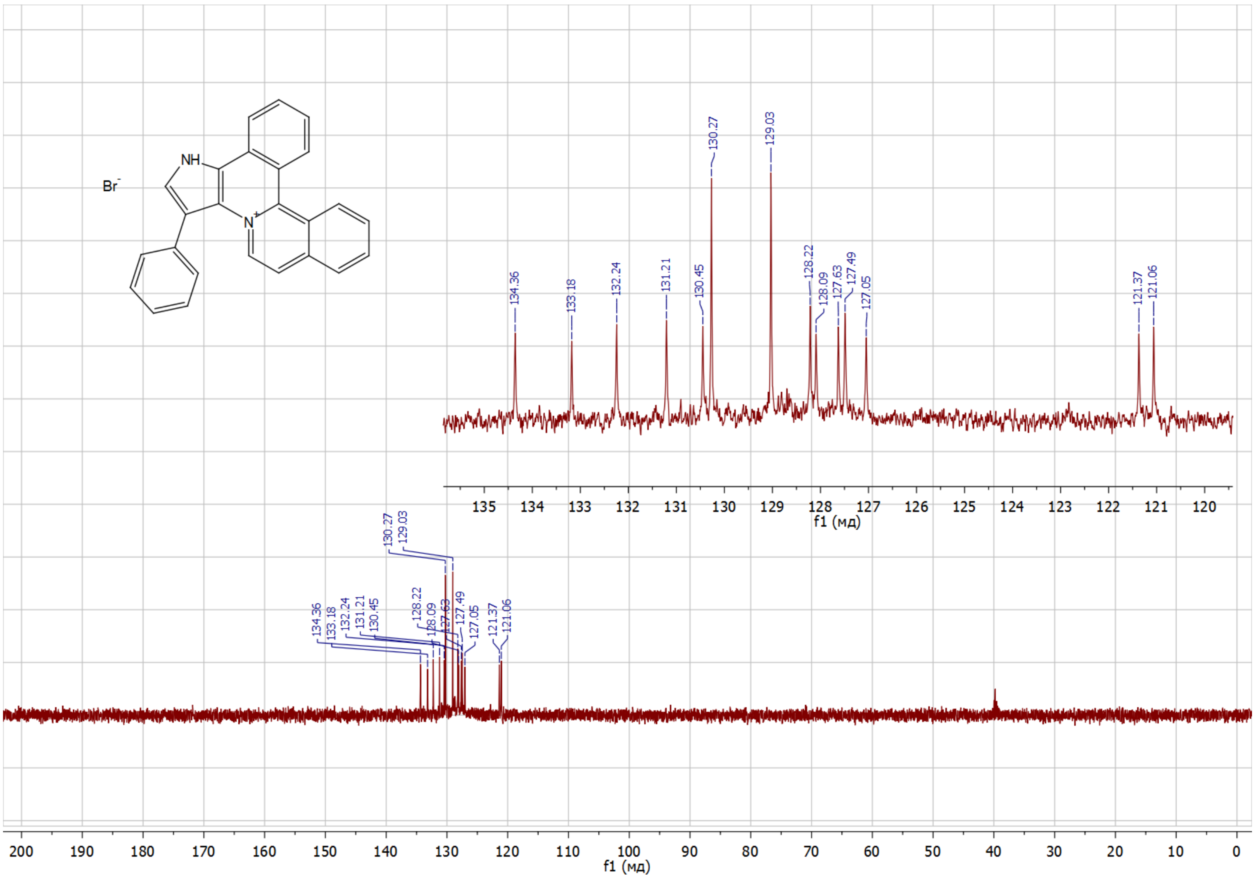

^{1}H NMR (400 MHz, DMSO-d_6) of 3-(4-methoxyphenyl)-1*H*-pyrido[2,1-*a*]pyrrolo[3,2-*c*]isoquinolin-4-ium bromide (**17h**)

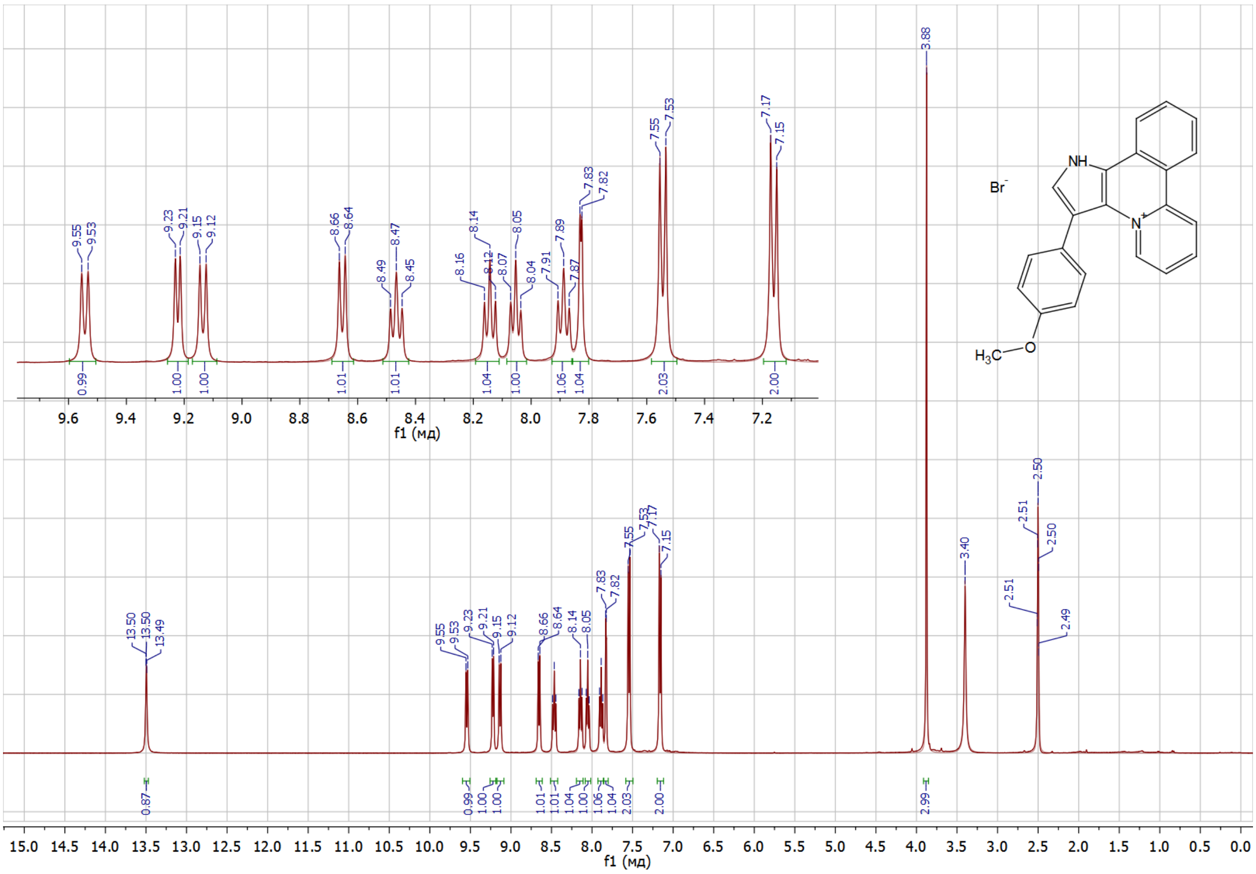

$^{13}C\{1H\}$ NMR (101 MHz, DMSO-d_6) of 3-(4-methoxyphenyl)-1*H*-pyrido[2,1-*a*]pyrrolo[3,2-*c*]isoquinolin-4-ium bromide (**17h**)

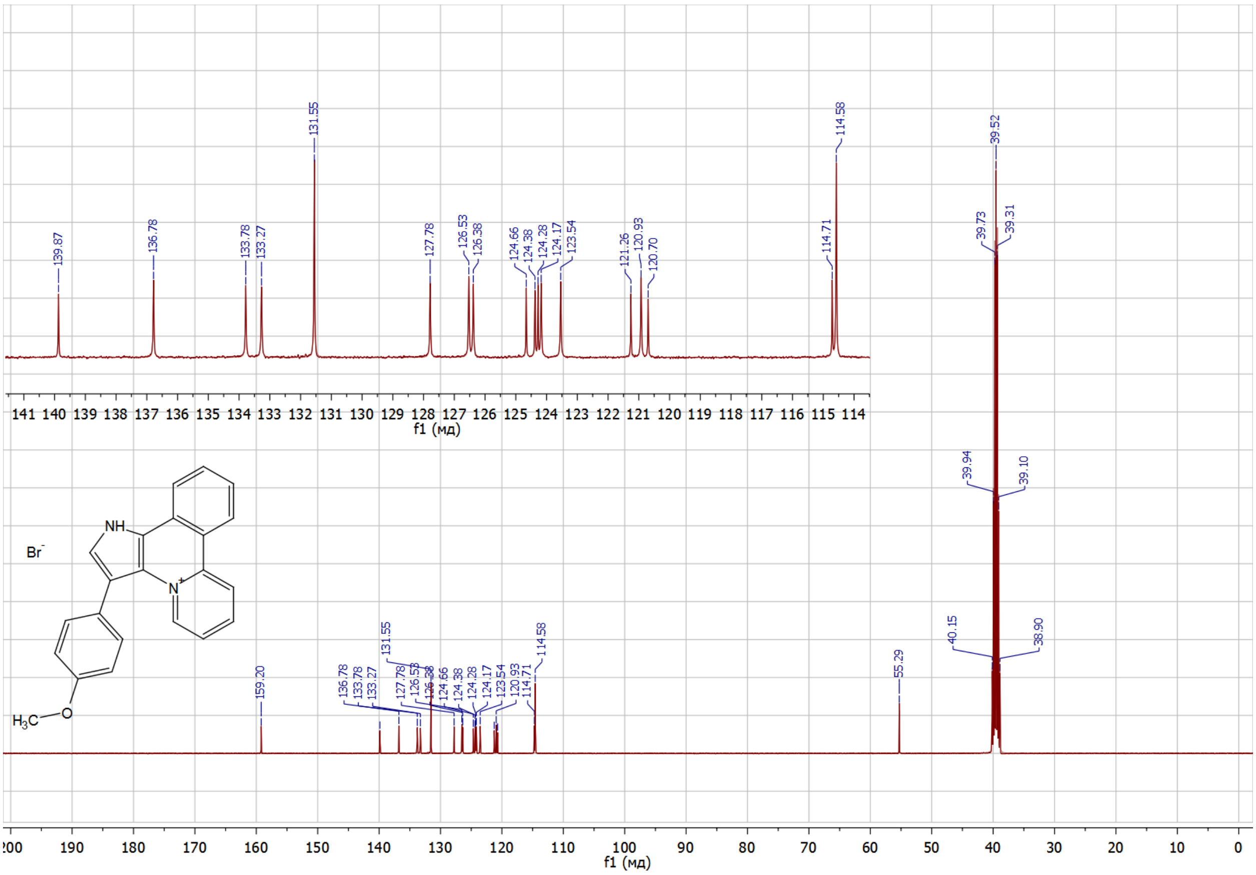

^{13}C DEPT135 NMR (101 MHz, DMSO-d_6) of 3-(4-methoxyphenyl)-1*H*-pyrido[2,1-*a*]pyrrolo[3,2-*c*]isoquinolin-4-ium bromide (**17h**)

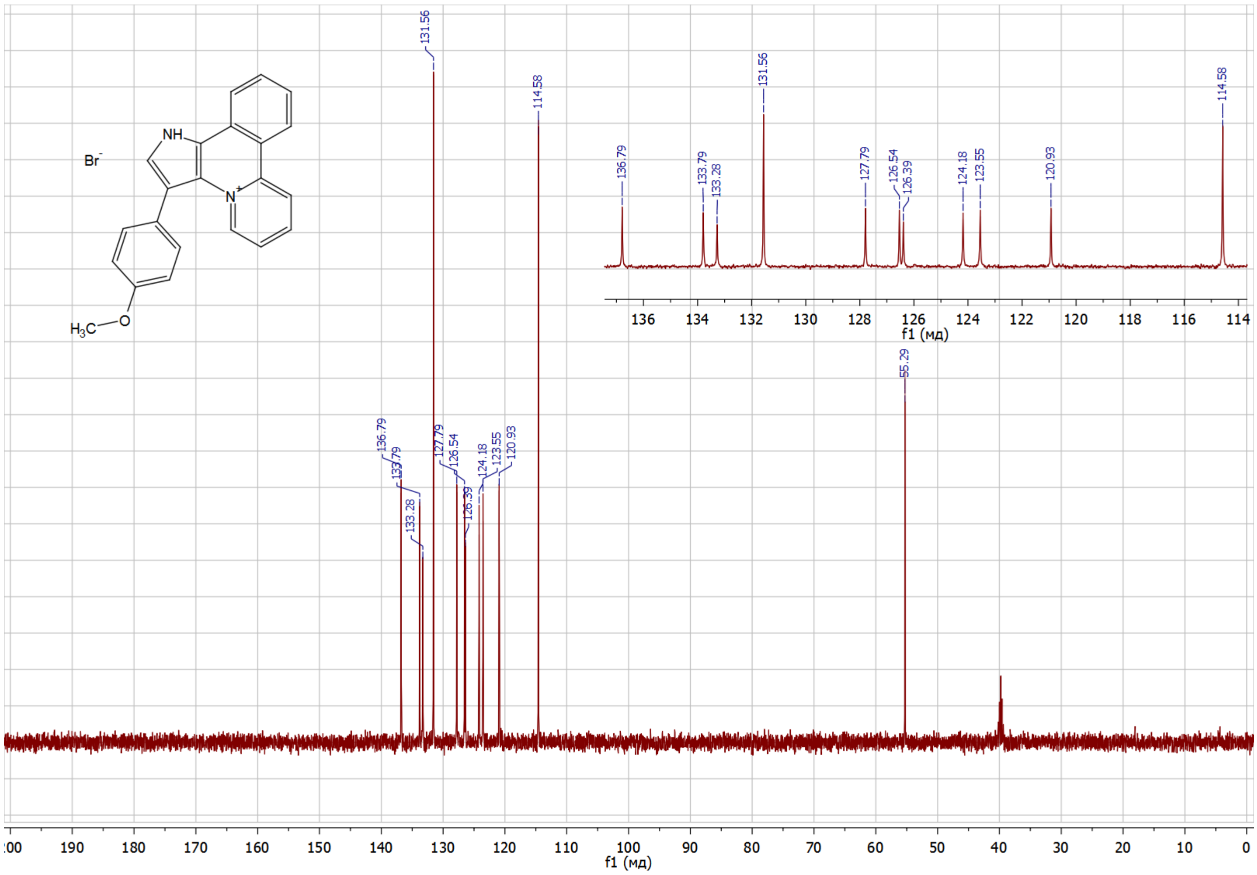

^{1}H NMR (400 MHz, DMSO-d_6) of 3-phenylpyrido[2,1-*a*]pyrrolo[3,2-*c*]isoquinoline (**18a**)

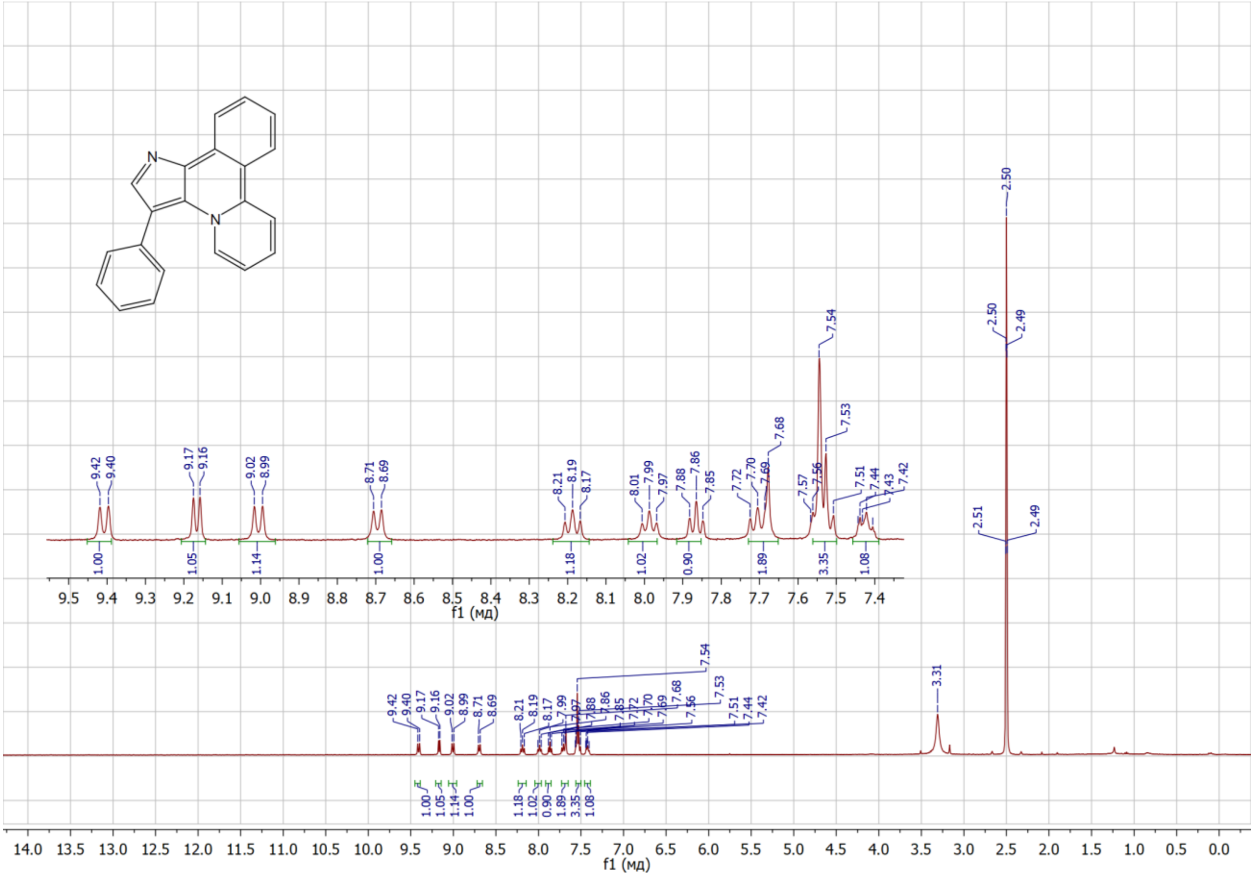

^{13}C{1H} NMR (101 MHz, DMSO-d_6) of 3-phenylpyrido[2,1-*a*]pyrrolo[3,2-*c*]isoquinoline (**18a**)

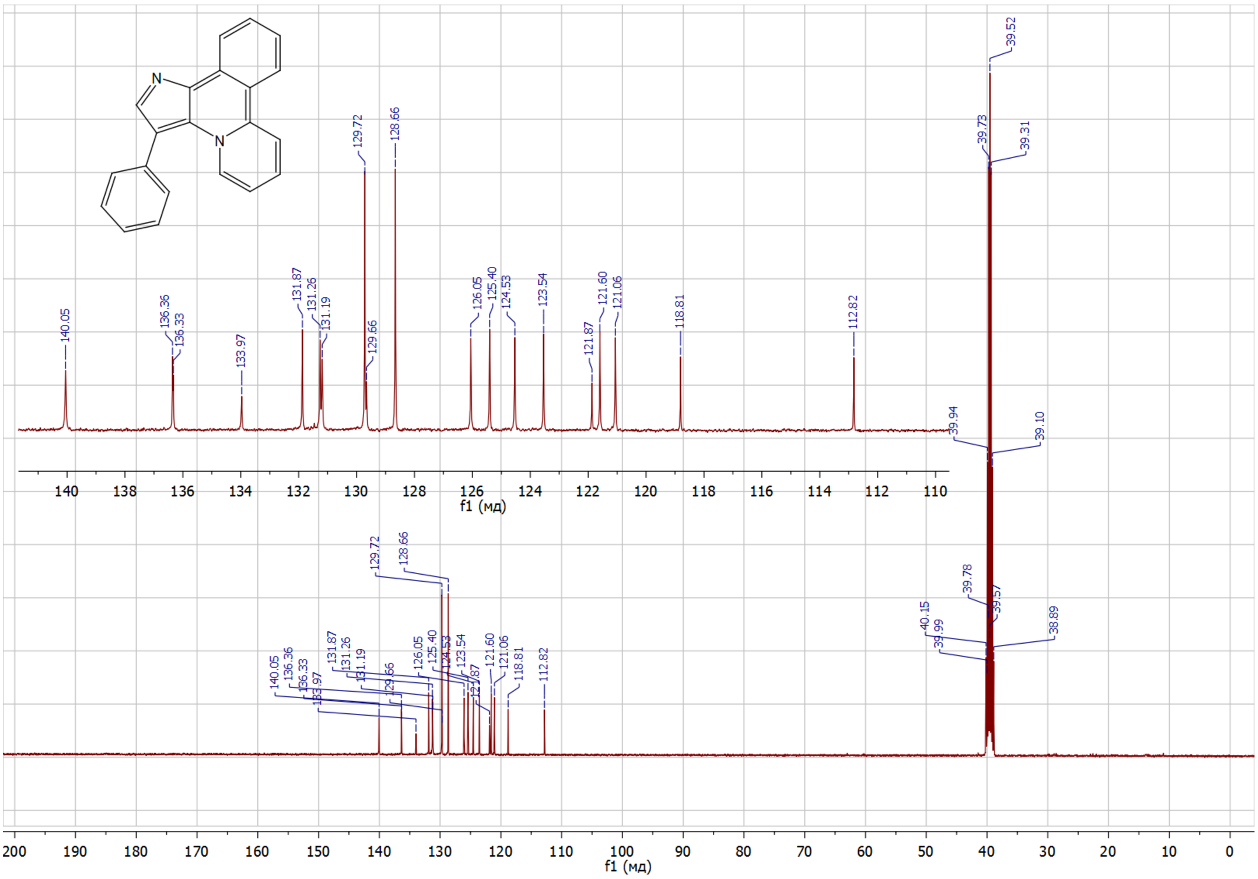

^{13}C DEPT135 NMR (101 MHz, DMSO-d_6) of 3-phenylpyrido[2,1-*a*]pyrrolo[3,2-*c*]isoquinoline (**18a**)

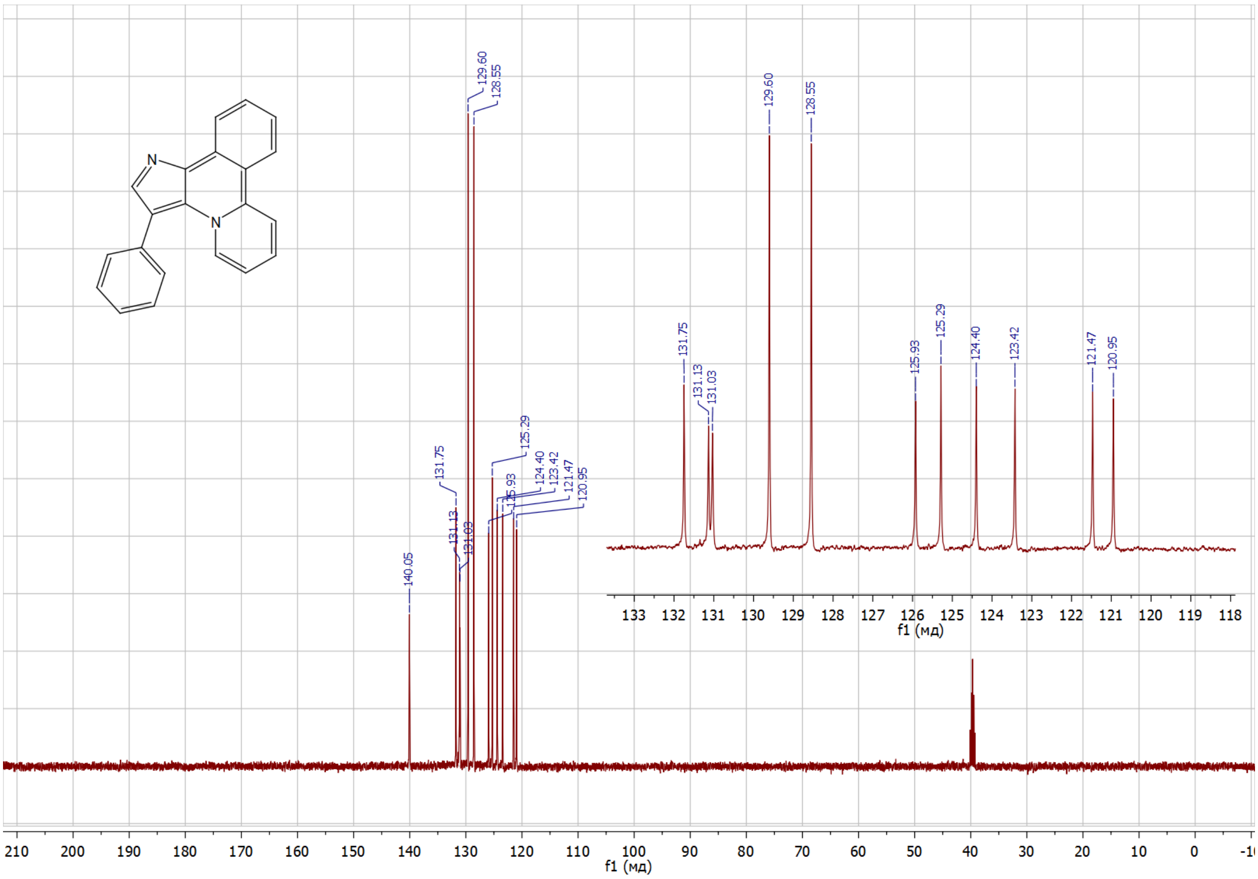

^{1}H NMR (400 MHz, DMSO-d_6) of 7-methoxy-3-phenylpyrido[2,1-*a*]pyrrolo[3,2-*c*]isoquinoline (**18c**)

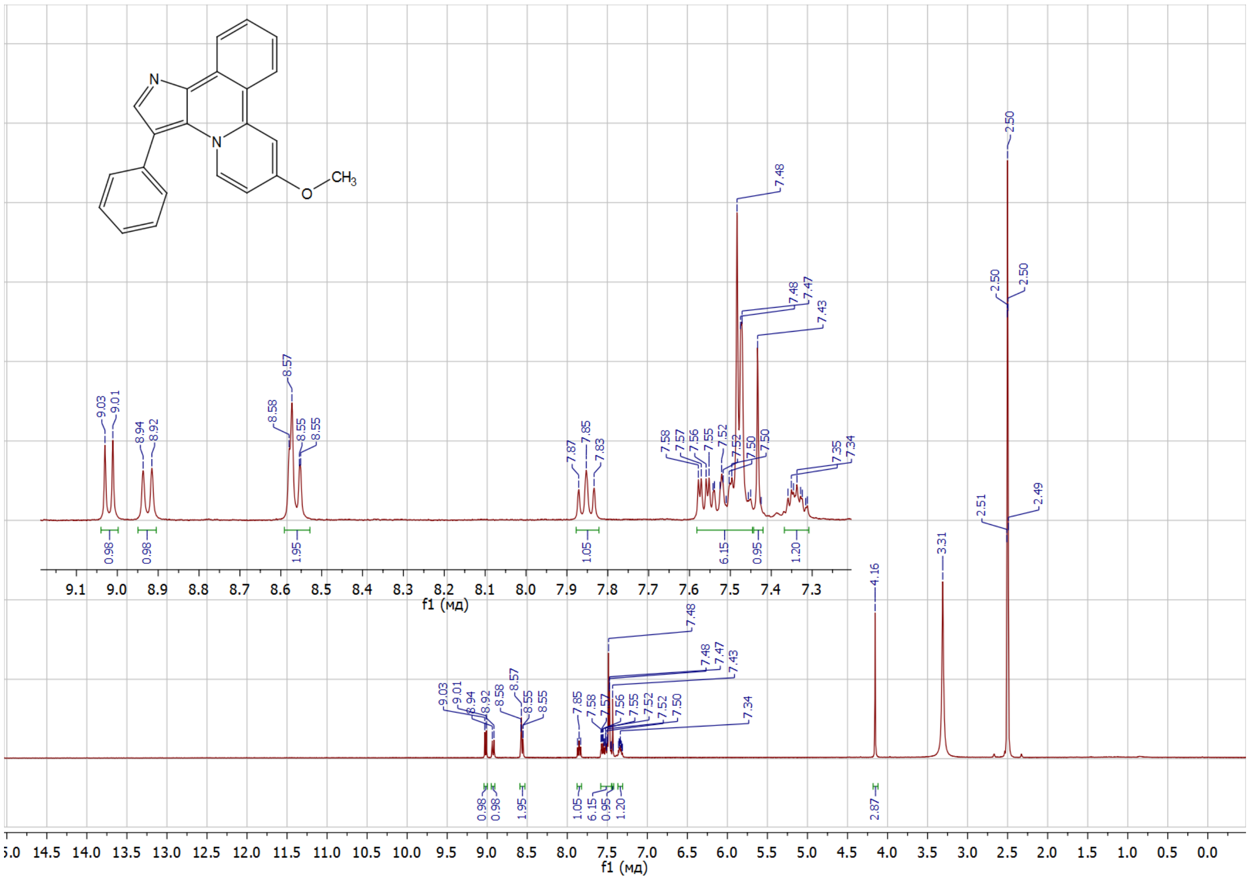

$^{13}C\{1H\}$ NMR (101 MHz, DMSO-d_6) of 7-methoxy-3-phenylpyrido[2,1-*a*]pyrrolo[3,2-*c*]isoquinoline (**18c**)

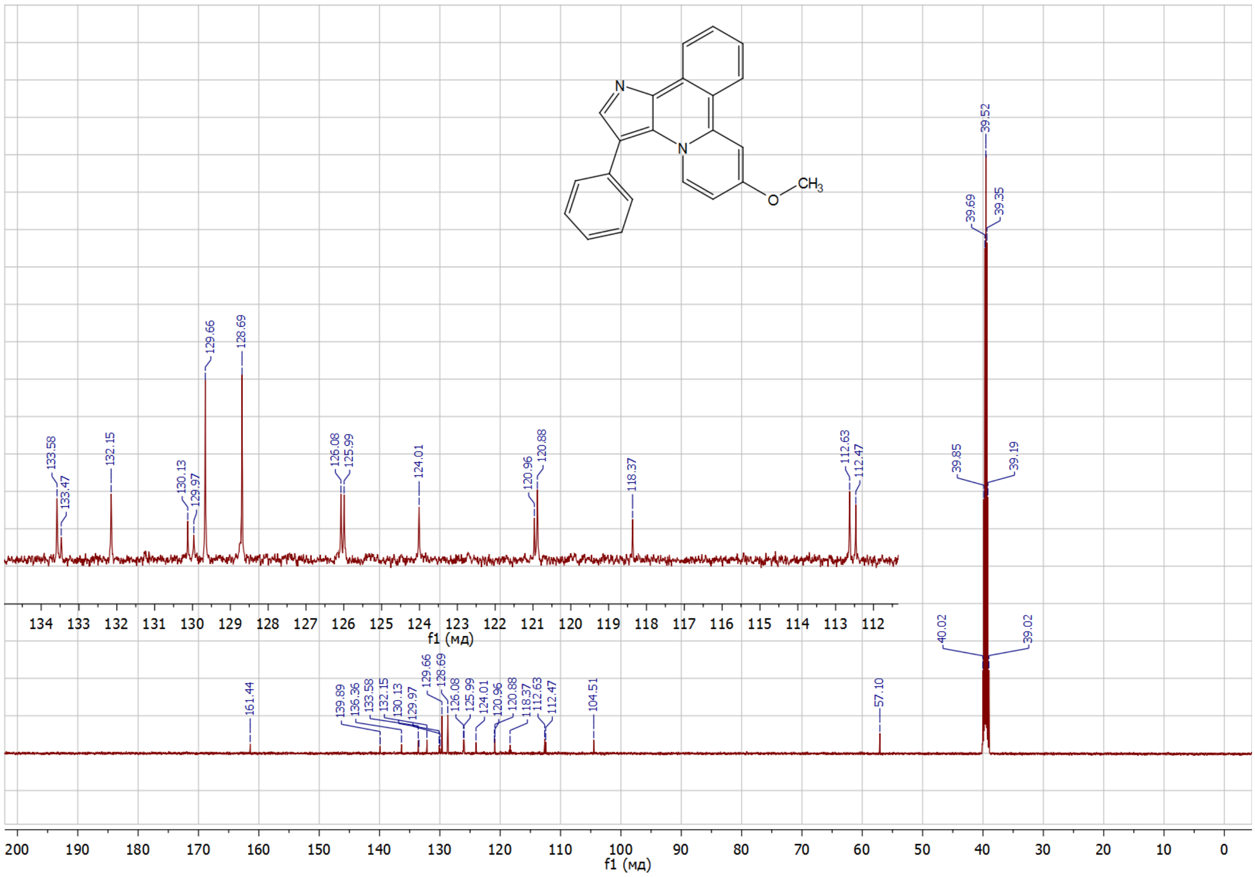

^{13}C DEPT135 NMR (101 MHz, DMSO-d_6) of 7-methoxy-3-phenylpyrido[2,1-*a*]pyrrolo[3,2-*c*]isoquinoline (**18c**)

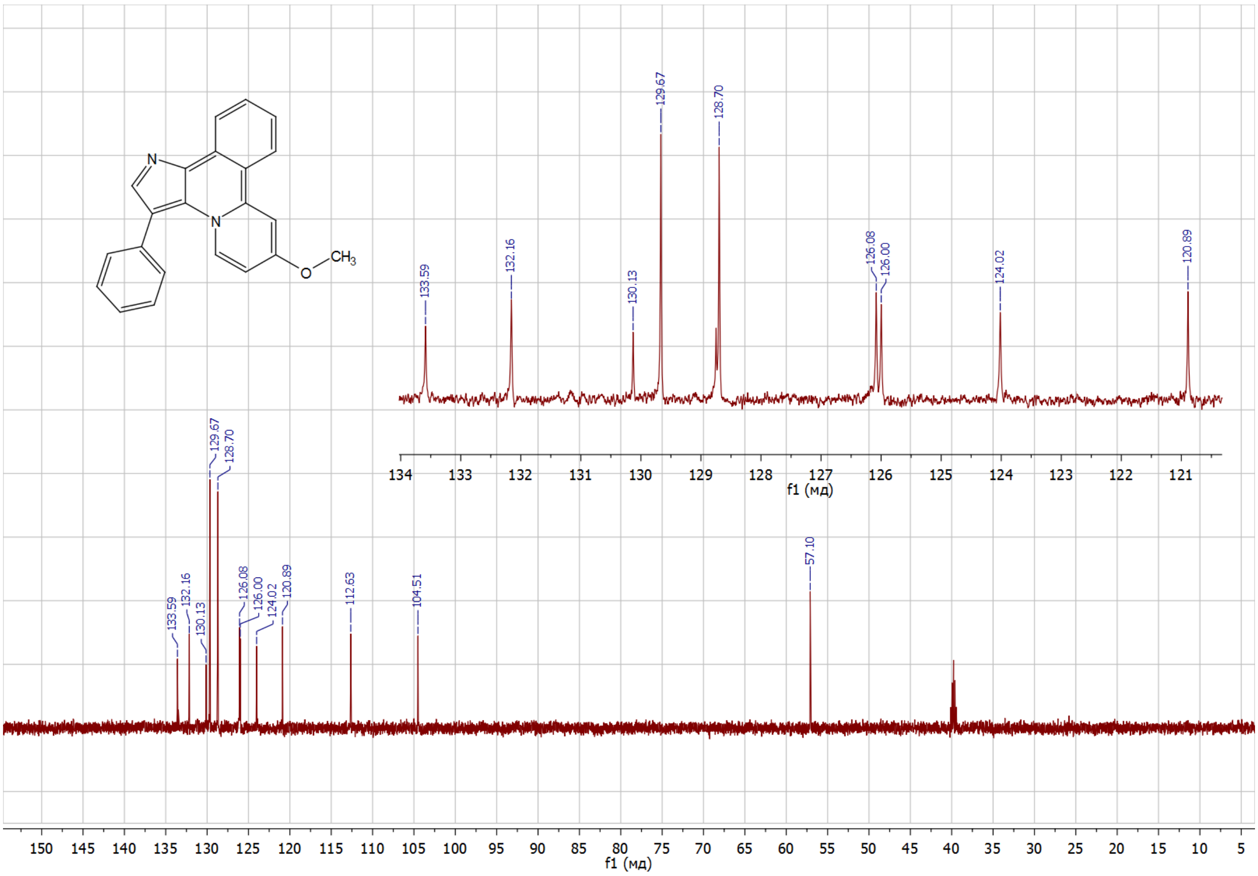

^{1}H NMR (400 MHz, DMSO-d_6) of 3,7-diphenylpyrido[2,1-*a*]pyrrolo[3,2-*c*]isoquinoline (**18d**)

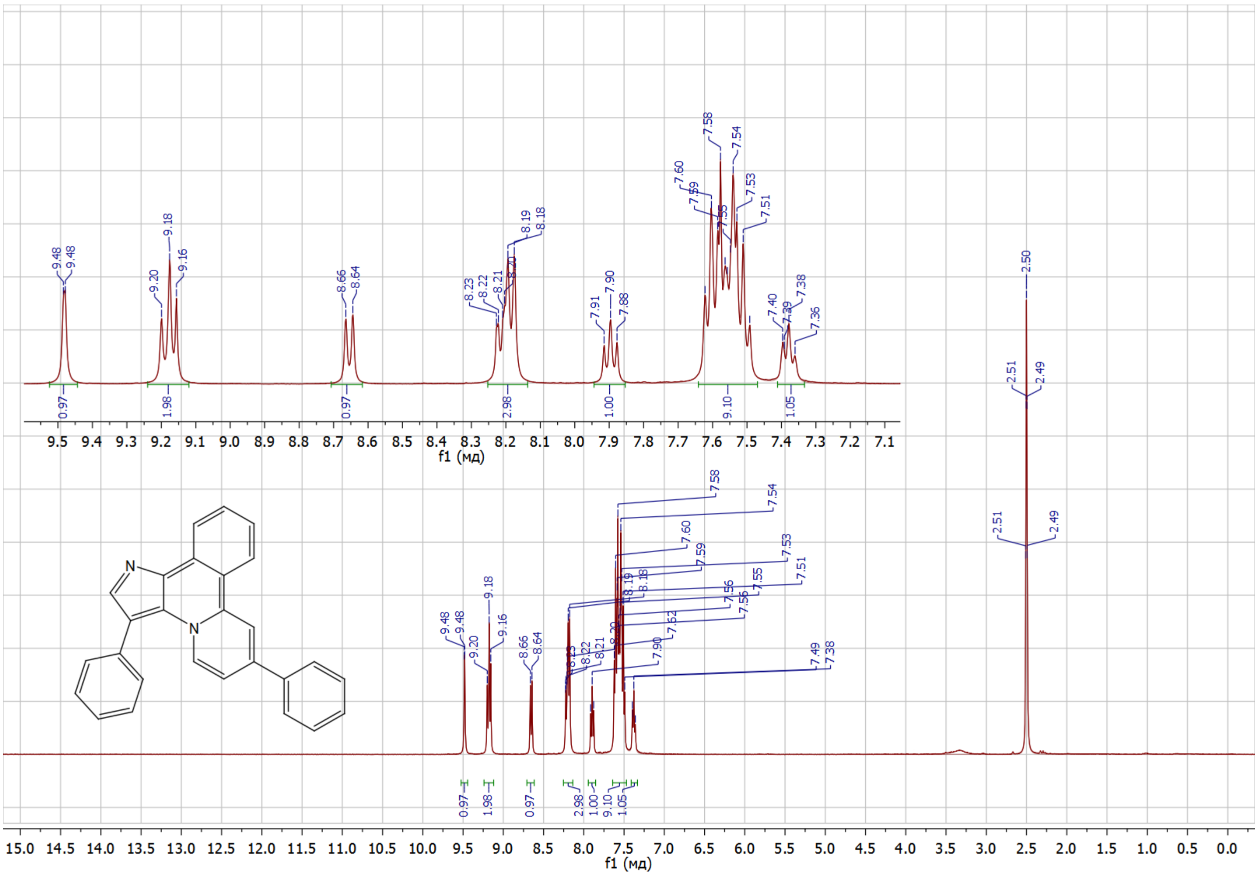

$^{13}C\{1H\}$ NMR (101 MHz, DMSO-d_6) of 3,7-diphenylpyrido[2,1-*a*]pyrrolo[3,2-*c*]isoquinoline (**18d**)

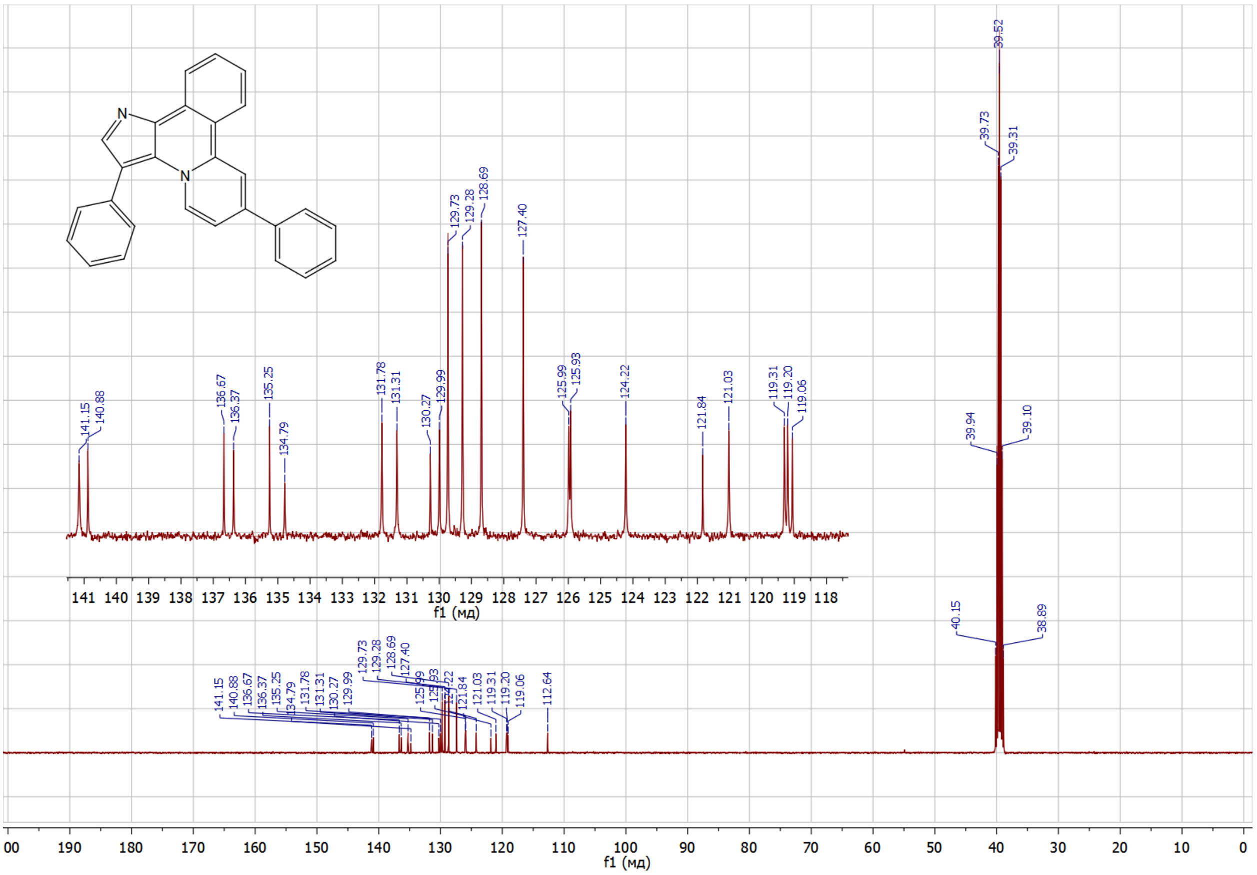

^{13}C DEPT135 NMR (101 MHz, DMSO-d_6) of 3,7-diphenylpyrido[2,1-*a*]pyrrolo[3,2-*c*]isoquinoline (**18d**)

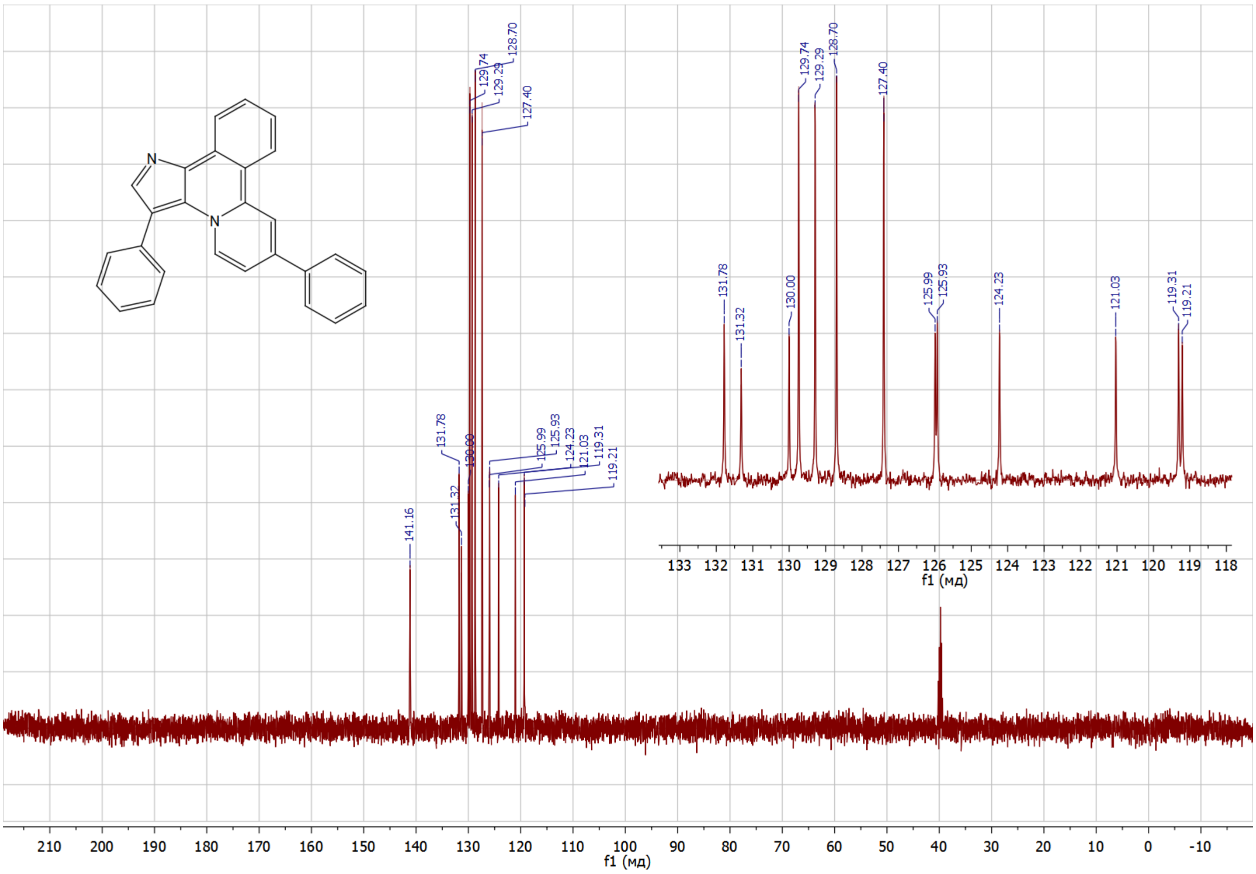

^{1}H NMR (400 MHz, DMSO-d_6) of 7-(4-methoxyphenyl)-3-phenylpyrido[2,1-*a*]pyrrolo[3,2-*c*]isoquinoline (**18e**)

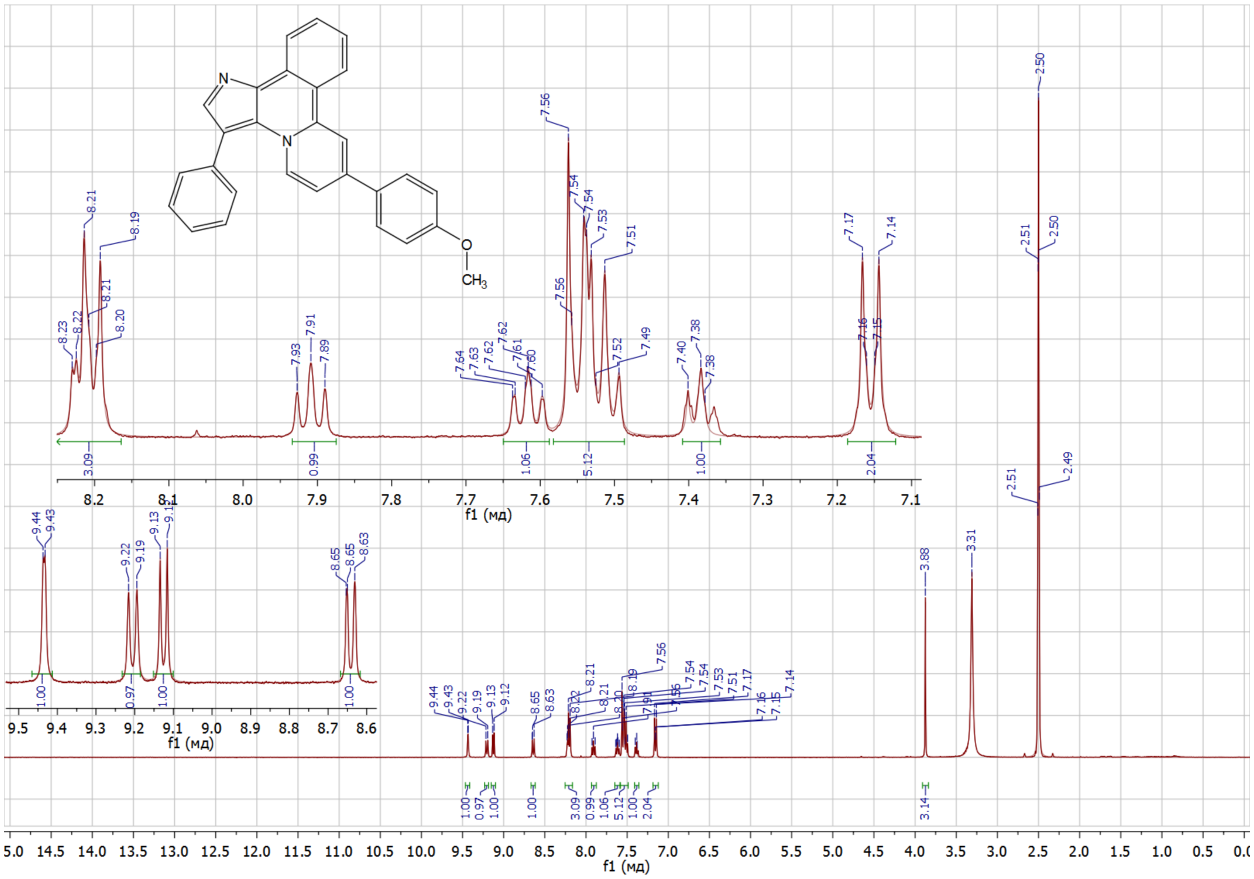

^{13}C{1H} NMR (101 MHz, DMSO-d_6) of 7-(4-methoxyphenyl)-3-phenylpyrido[2,1-*a*]pyrrolo[3,2-*c*]isoquinoline (**18e**)

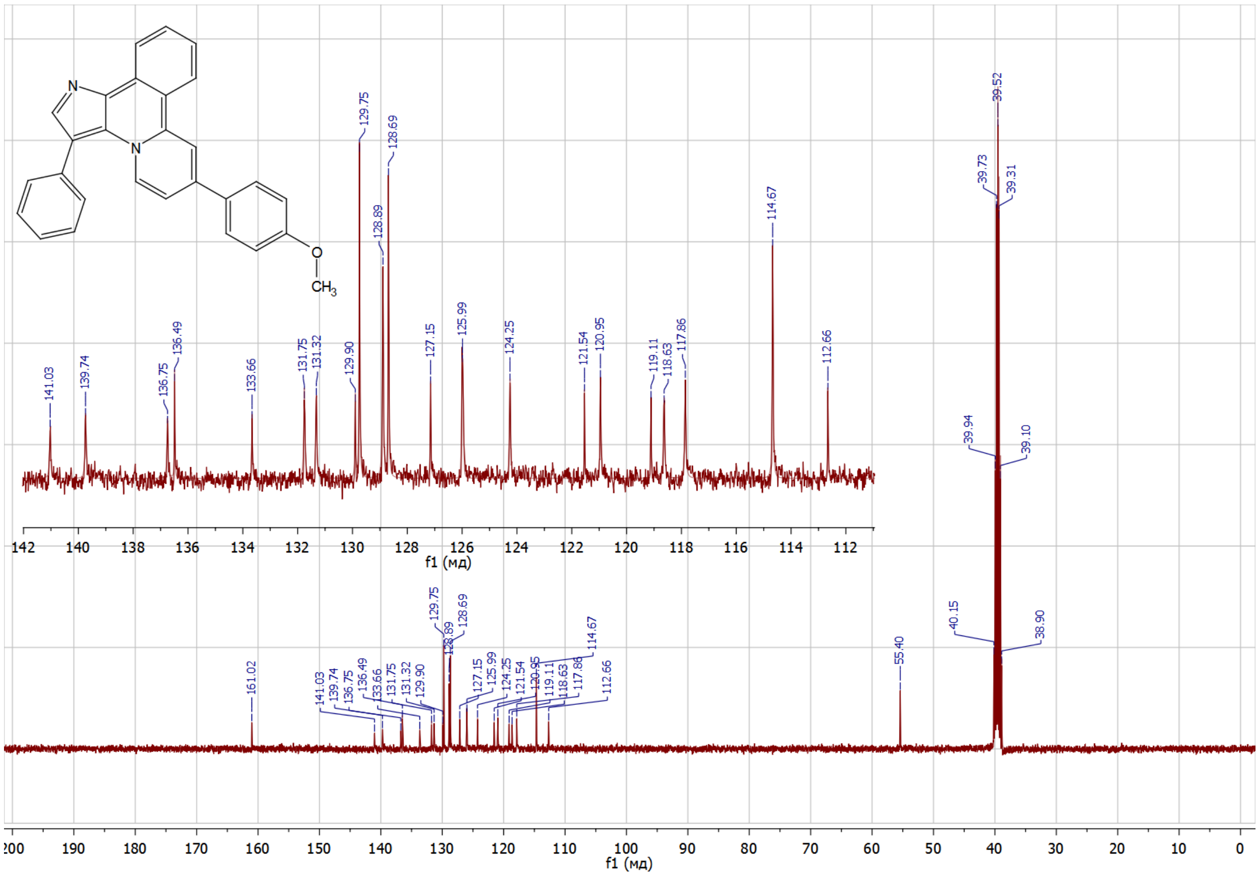

^{13}C DEPT135 NMR (101 MHz, DMSO-d_6) of 7-(4-methoxyphenyl)-3-phenylpyrido[2,1-*a*]pyrrolo[3,2-*c*]isoquinoline (**18e**)

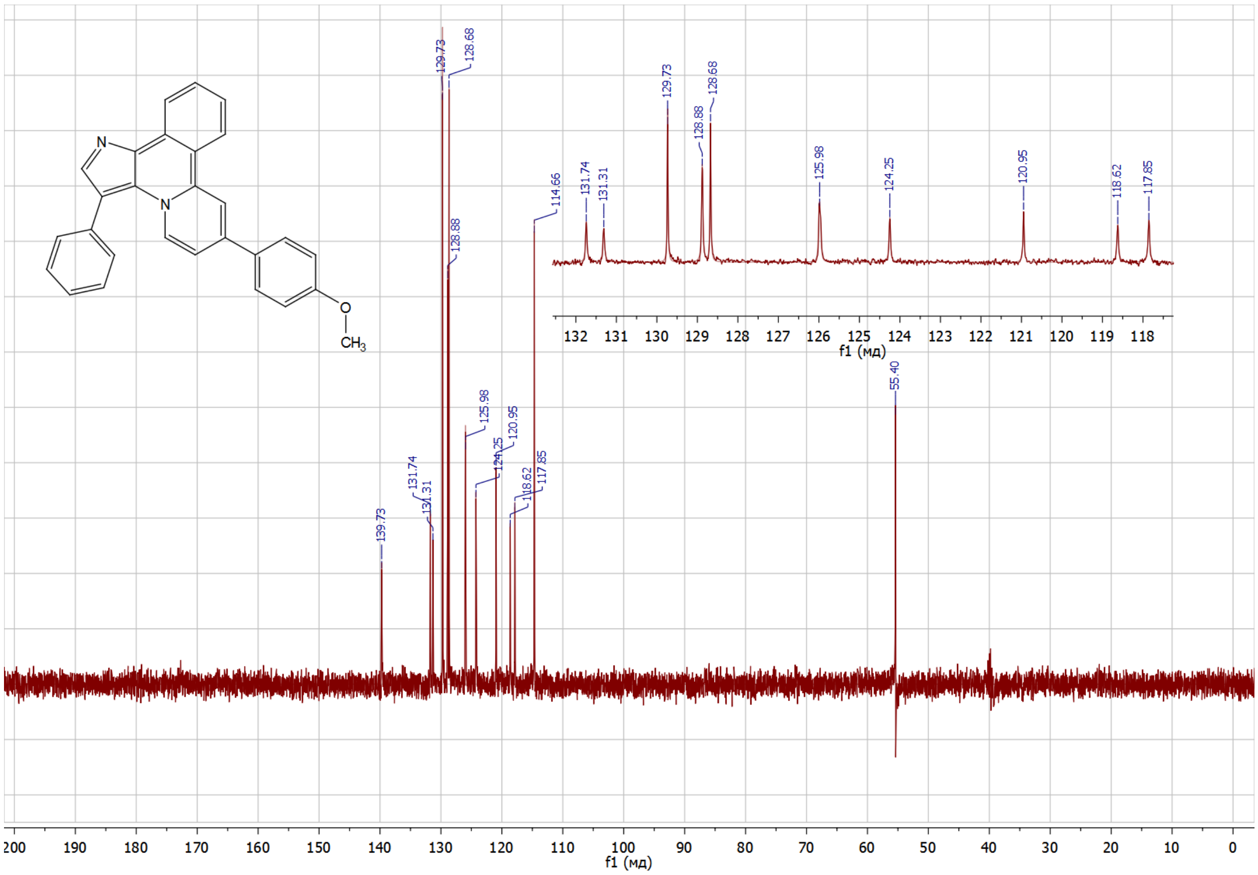

^{1}H NMR (400 MHz, DMSO-d_6) of 6,8-dimethyl-3-phenylpyrido[2,1-*a*]pyrrolo[3,2-*c*]isoquinoline (**18f**)

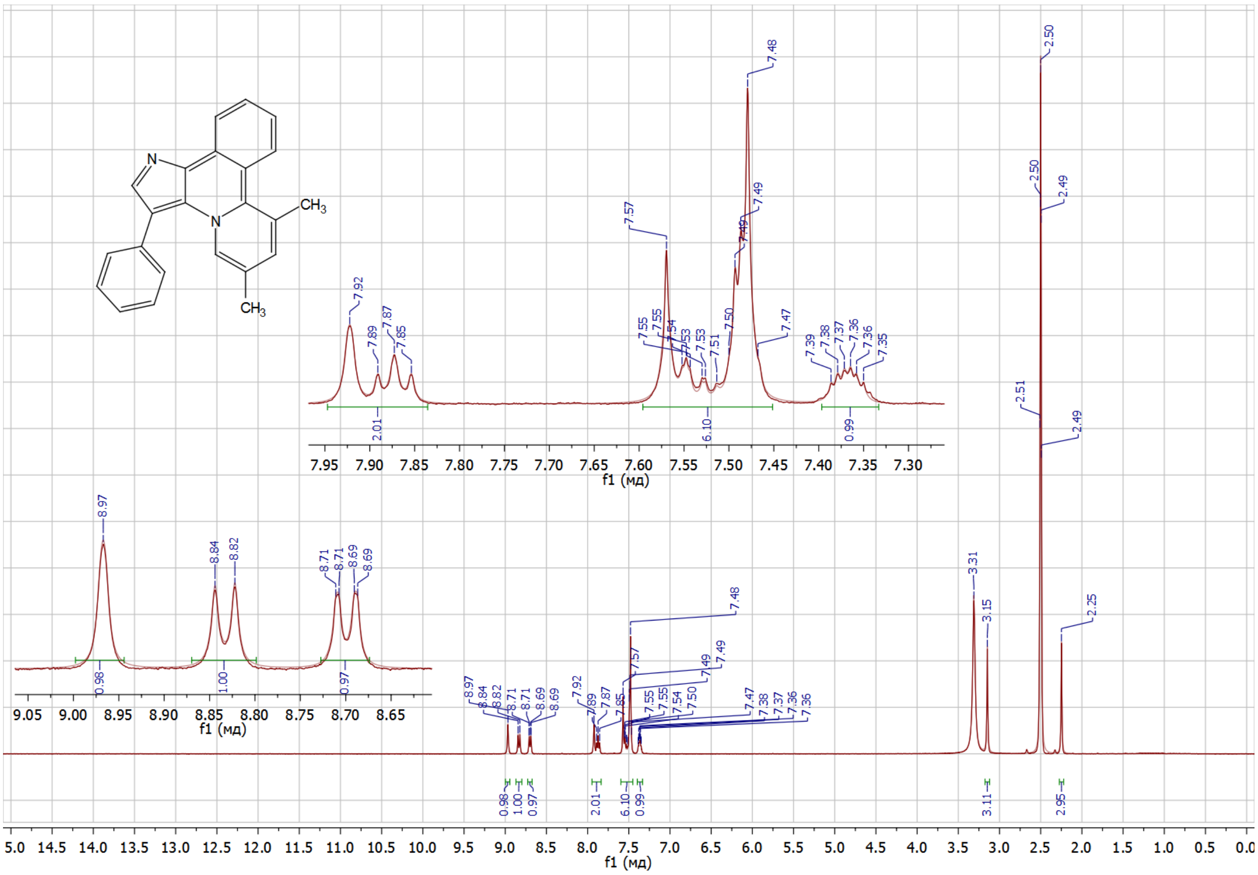

$^{13}C\{1H\}$ NMR (101 MHz, DMSO-d_6) of 6,8-dimethyl-3-phenylpyrido[2,1-*a*]pyrrolo[3,2-*c*]isoquinoline (**18f**)

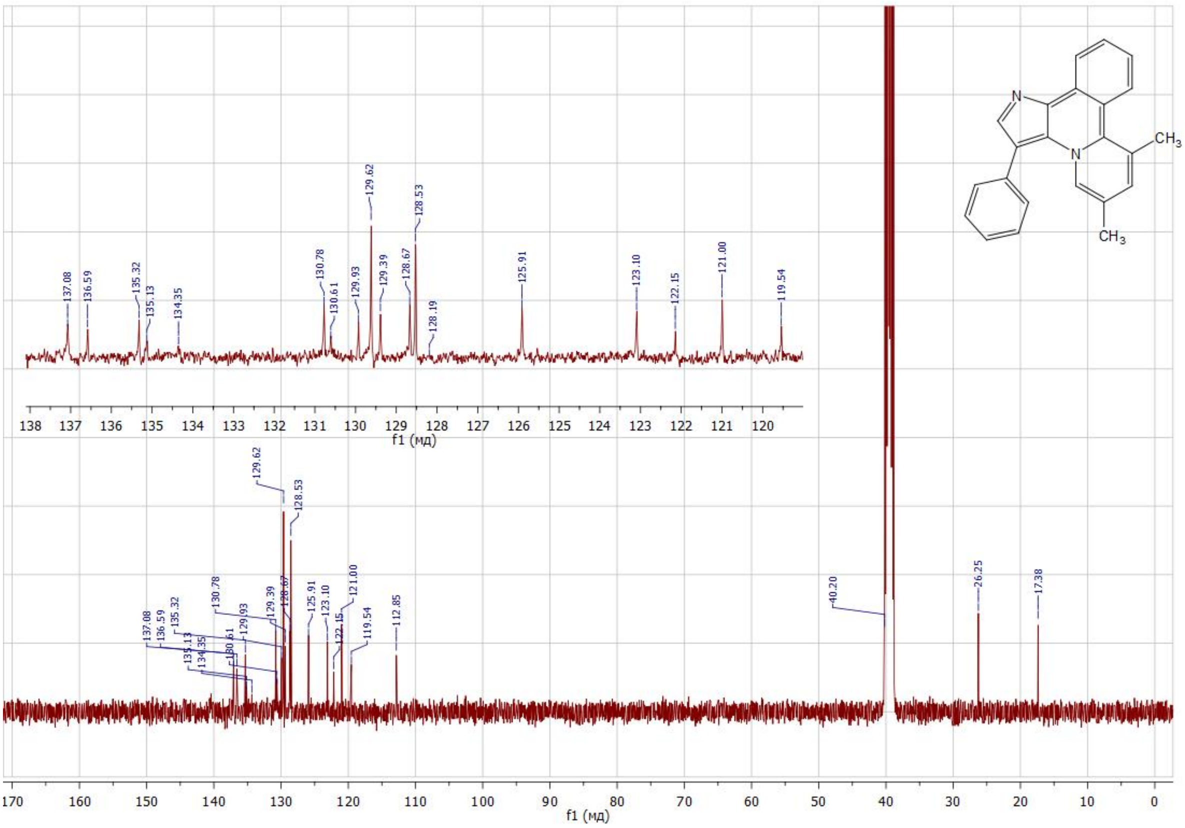

^{13}C DEPT135 NMR (101 MHz, DMSO-d_6) of 6,8-dimethyl-3-phenylpyrido[2,1-*a*]pyrrolo[3,2-*c*]isoquinoline (**18f**)

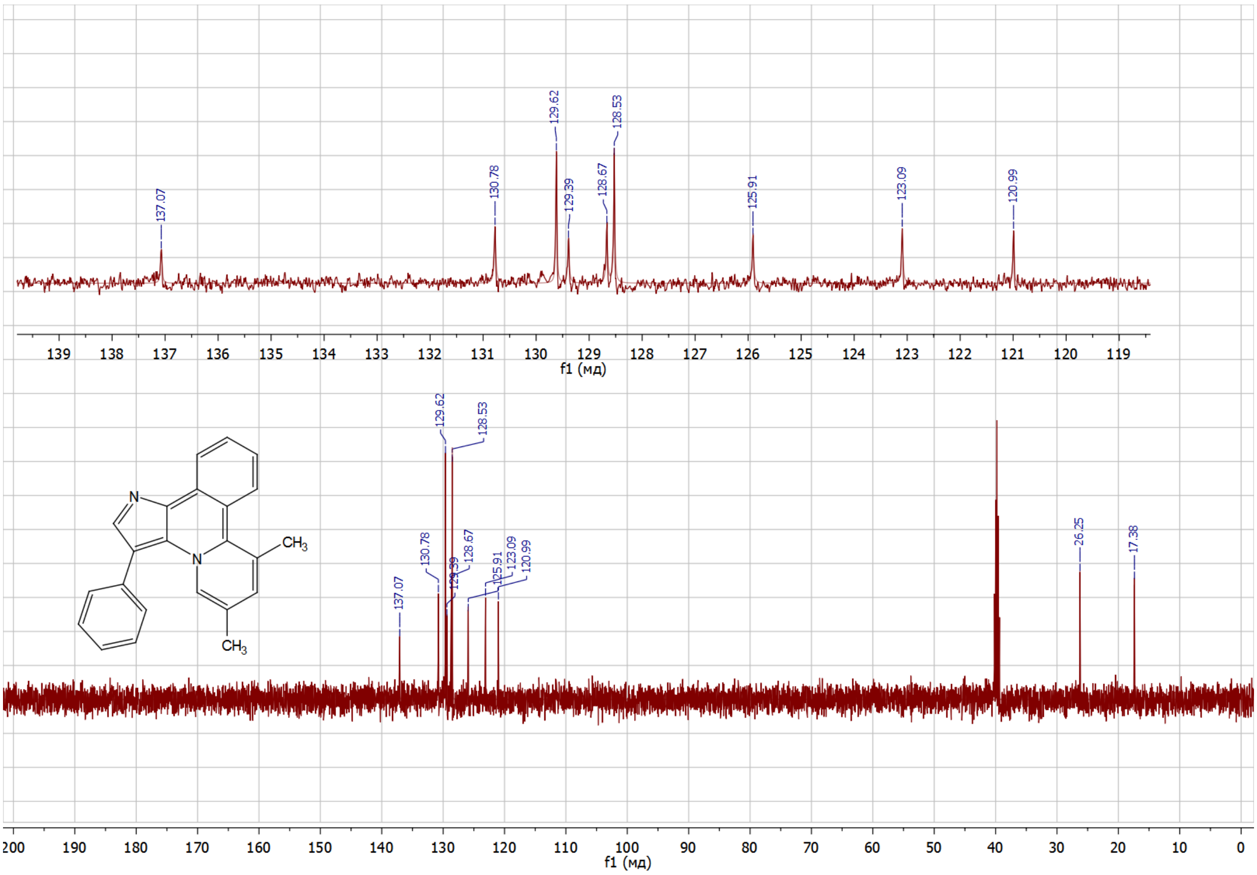

^{1}H NMR (400 MHz, DMSO-d_6) of 3-phenylisoquinolino[1,2-*a*]pyrrolo[3,2-*c*]isoquinoline (**18g**)

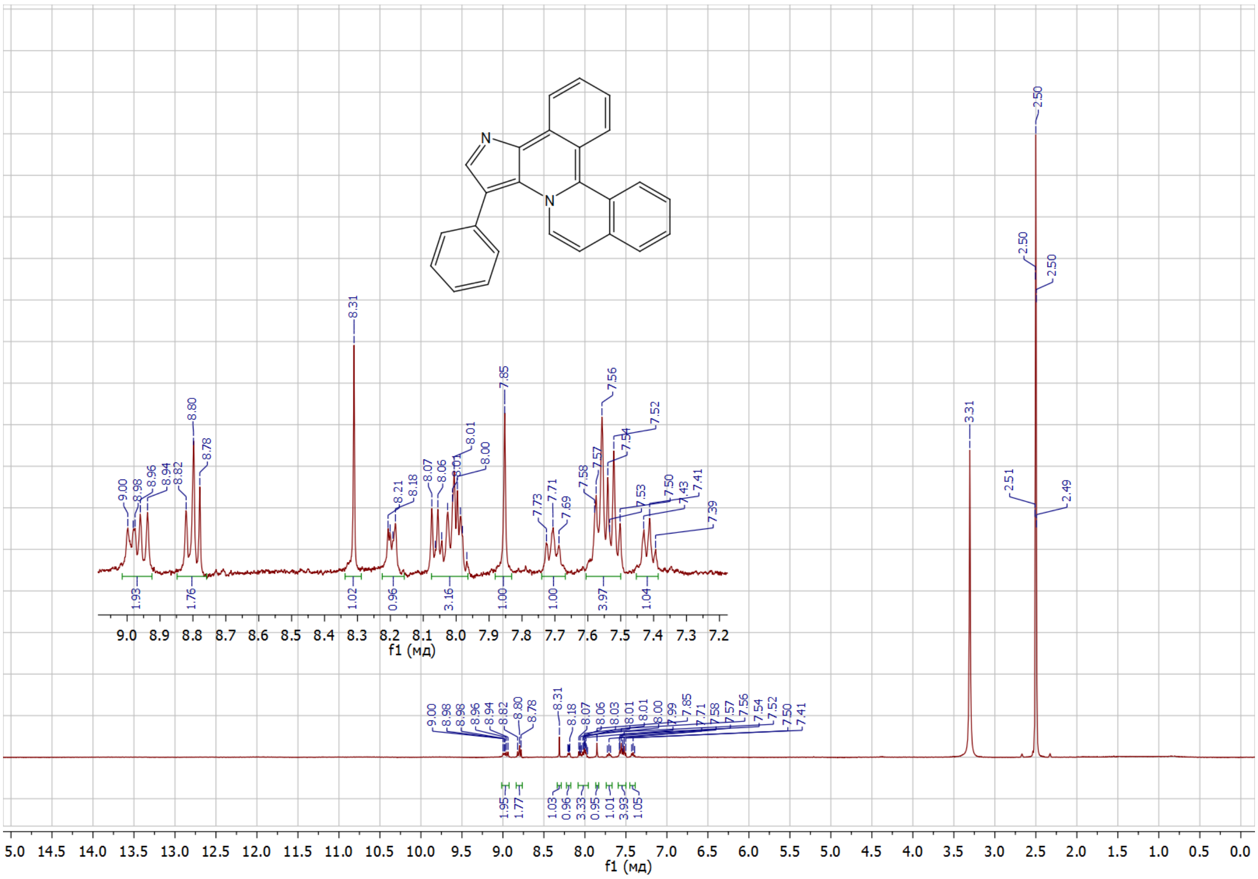

^{13}C{1H} NMR (101 MHz, DMSO-d_6) of 3-phenylisoquinolino[1,2-*a*]pyrrolo[3,2-*c*]isoquinoline (**18g**)

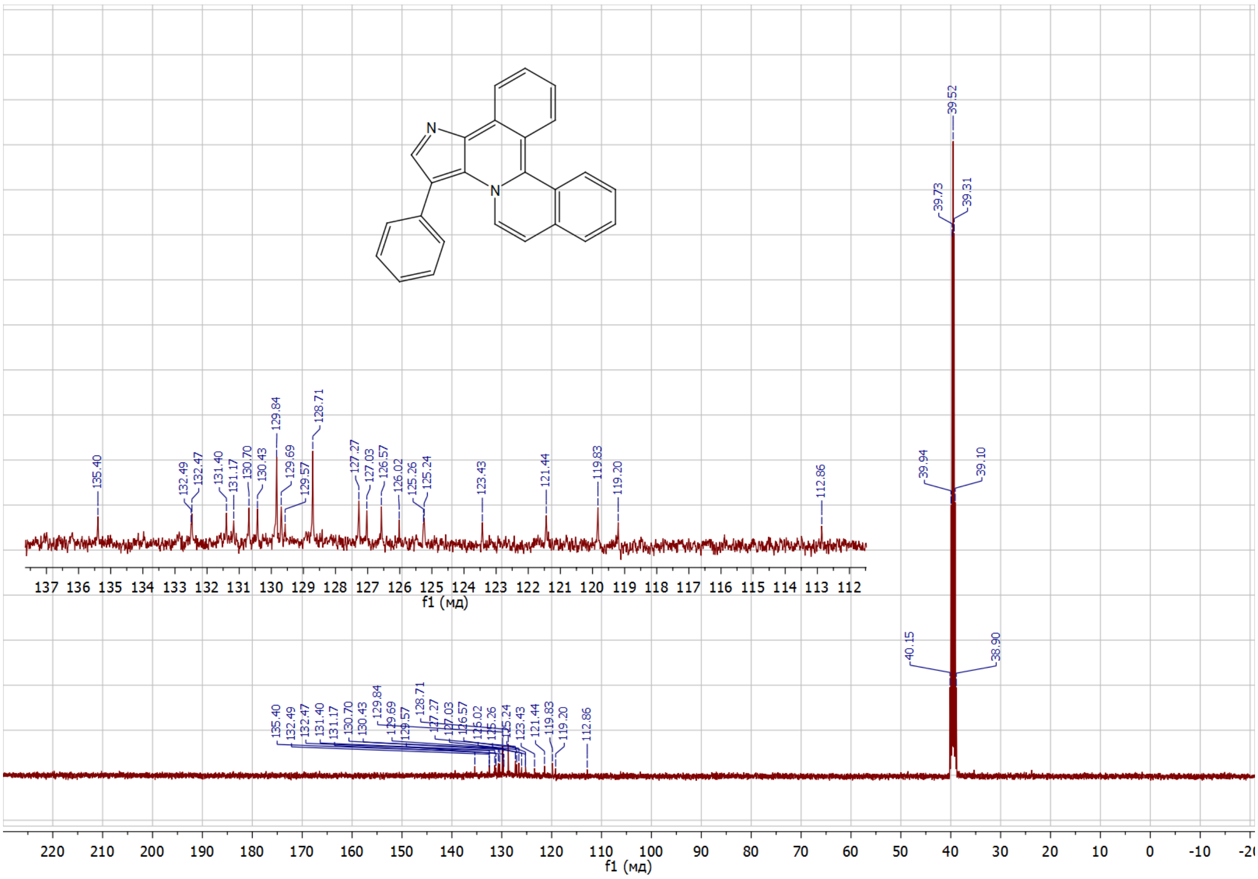

^{13}C DEPT135 NMR (101 MHz, DMSO-d_6) of 3-phenylisoquinolino[1,2-*a*]pyrrolo[3,2-*c*]isoquinoline (**18g**)

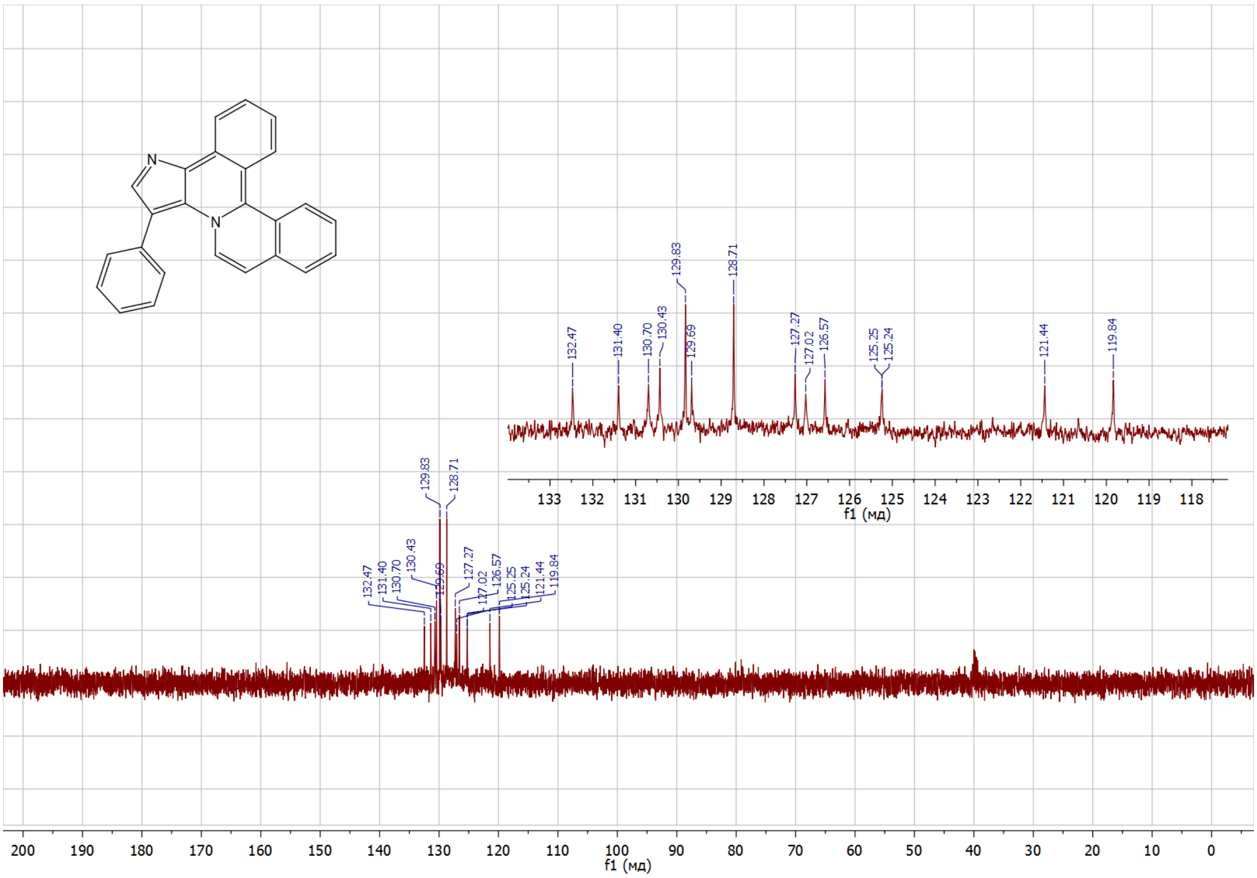

^{1}H NMR (400 MHz, DMSO-d_6) of 3-(4-methoxyphenyl)pyrido[2,1-*a*]pyrrolo[3,2-*c*]isoquinoline (**18h**)

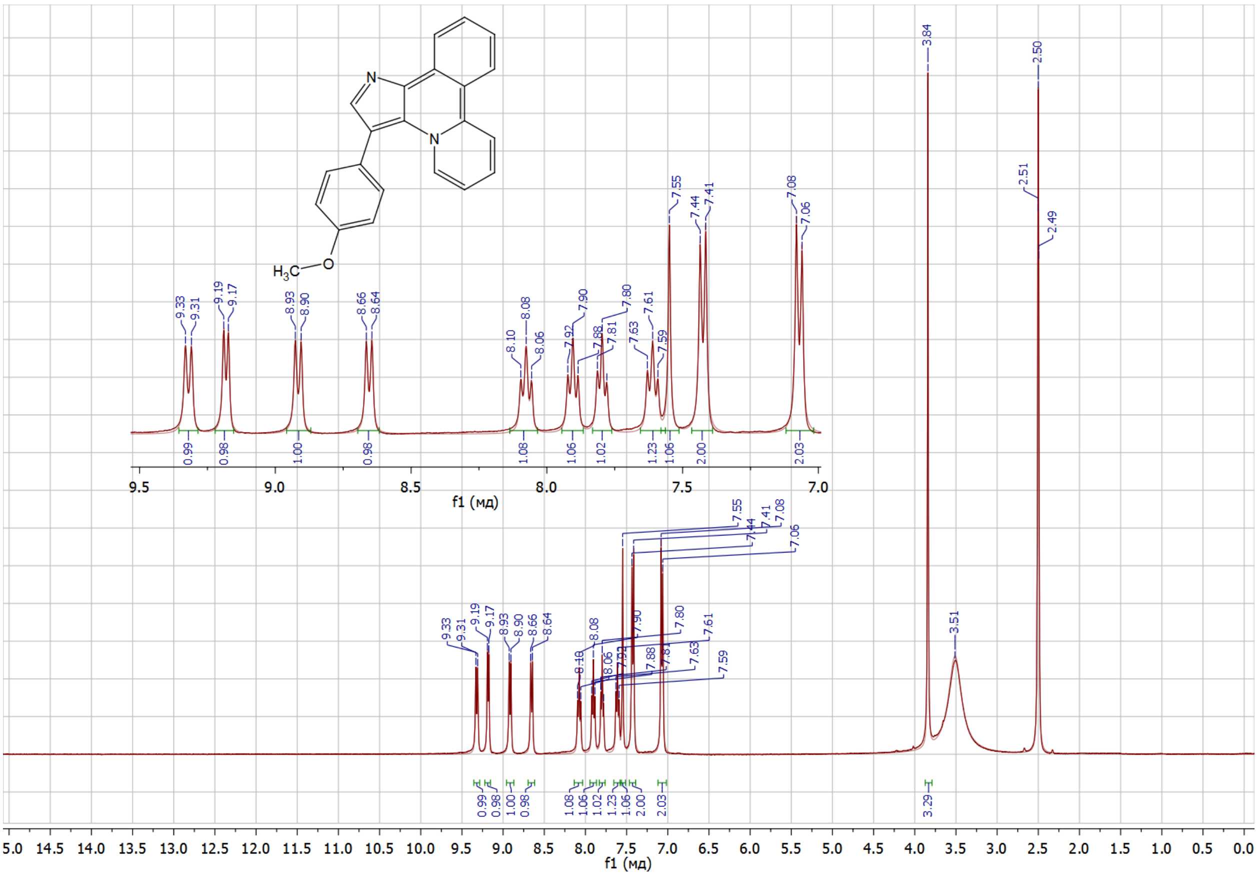

^{13}C{1H} NMR (101 MHz, DMSO-d_6) of 3-(4-methoxyphenyl)pyrido[2,1-*a*]pyrrolo[3,2-*c*]isoquinoline (**18h**)

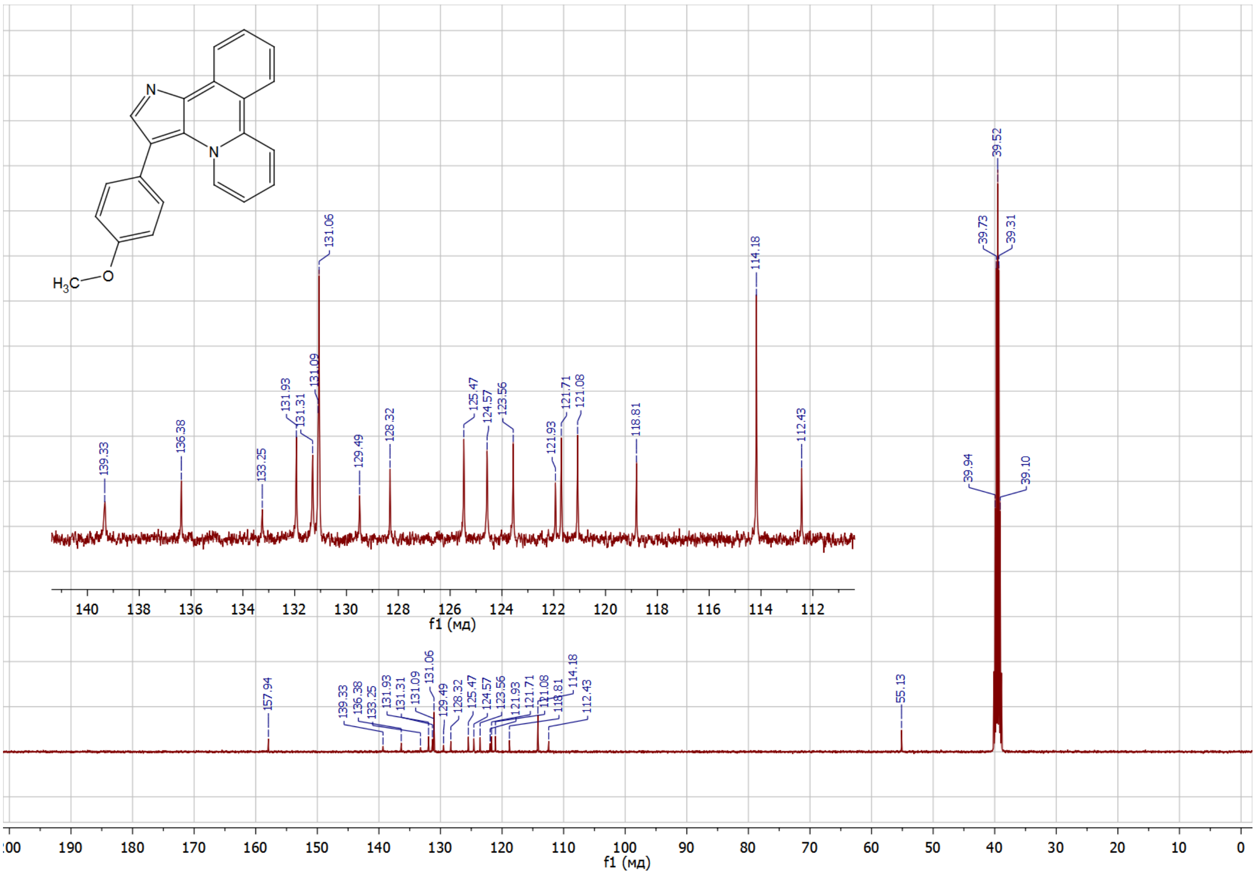

^{13}C DEPT135 NMR (101 MHz, DMSO-d_6) of 3-(4-methoxyphenyl)pyrido[2,1-*a*]pyrrolo[3,2-*c*]isoquinoline (**18h**)

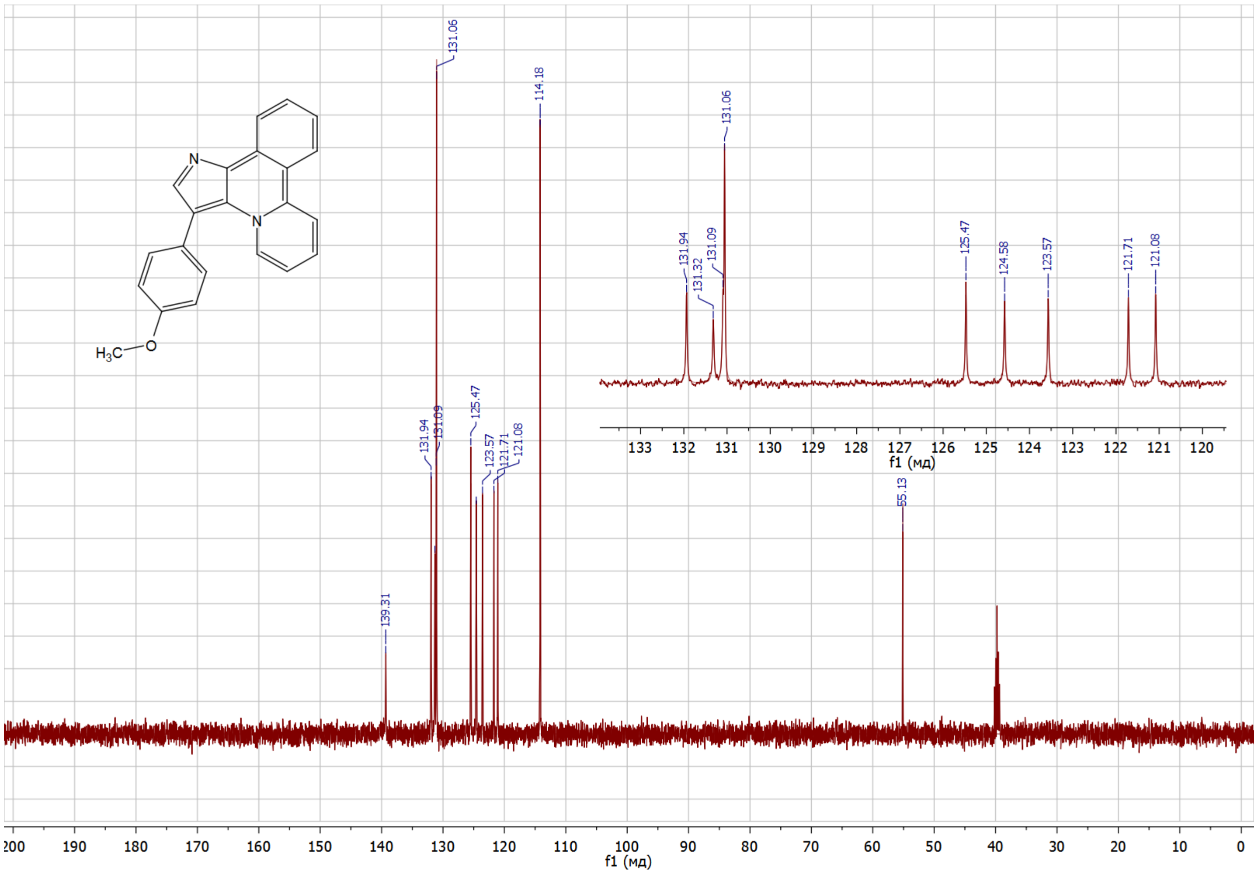

^{1}H NMR (400 MHz, DMSO-d_6) of 1-methyl-2-phenyl-1*H*-pyrido[2,1-*a*]pyrrolo[3,2-*c*]isoquinolin-4-ium iodide (**N-Me-19**)

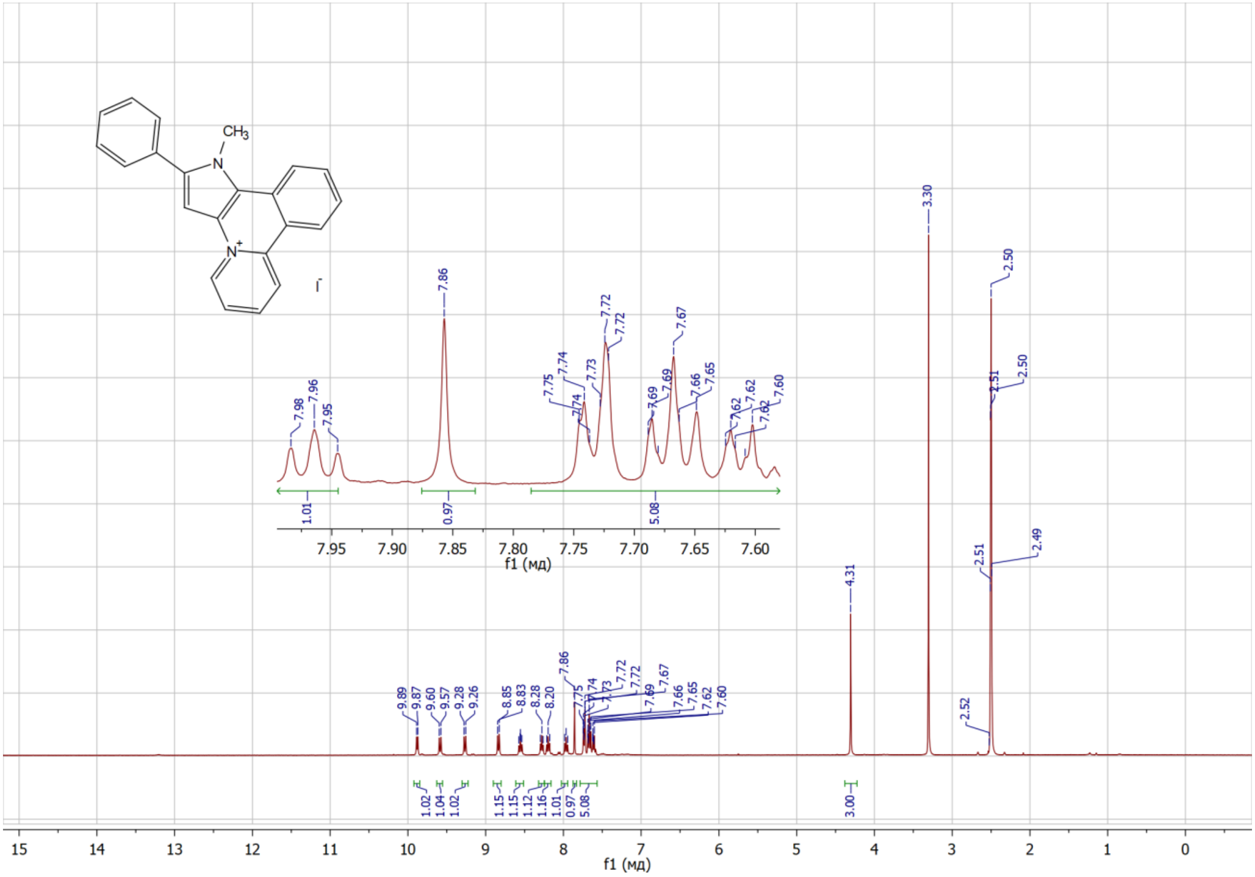

$^{13}C\{^{1}H\}$ NMR (101 MHz, DMSO-d_6) of 1-methyl-2-phenyl-1*H*-pyrido[2,1-*a*]pyrrolo[3,2-*c*]isoquinolin-4-ium iodide (**N-Me-19**)

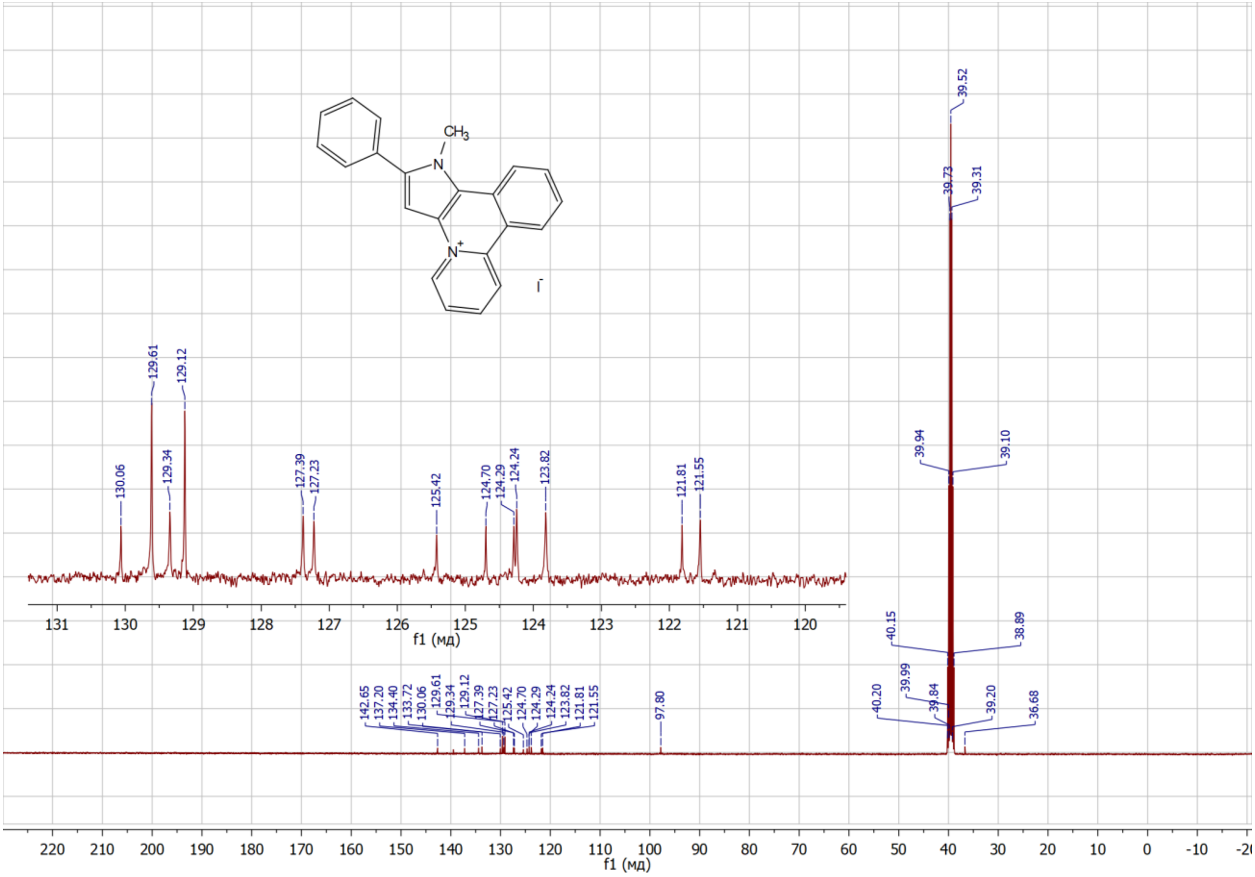

^{13}C DEPT135 NMR (101 MHz, DMSO-d_6) of 1-methyl-2-phenyl-1*H*-pyrido[2,1-*a*]pyrrolo[3,2-*c*]isoquinolin-4-ium iodide (**N-Me-19**)

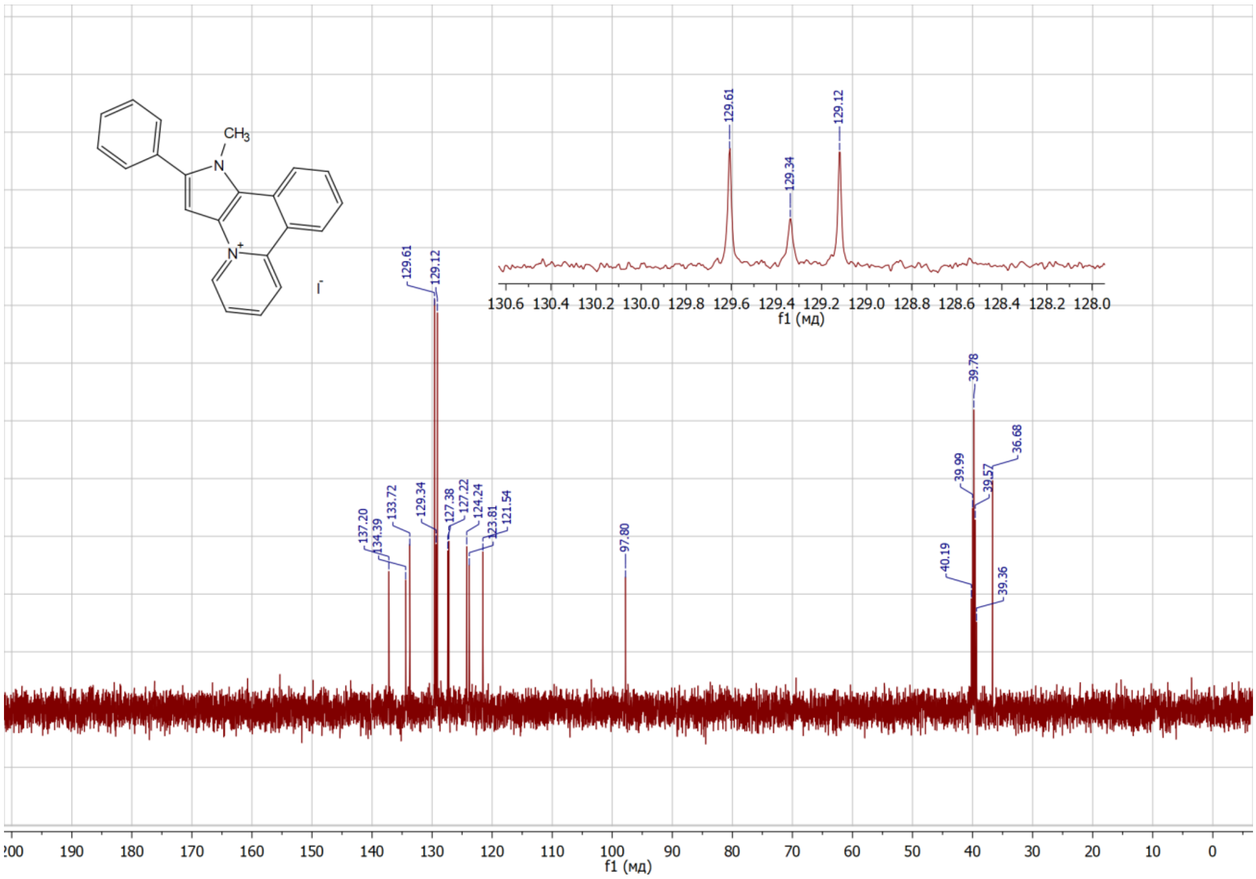

^{1}H NMR (400 MHz, $CDCl_3$) of 1-(1,2-dibromoethyl)-2-iodobenzene

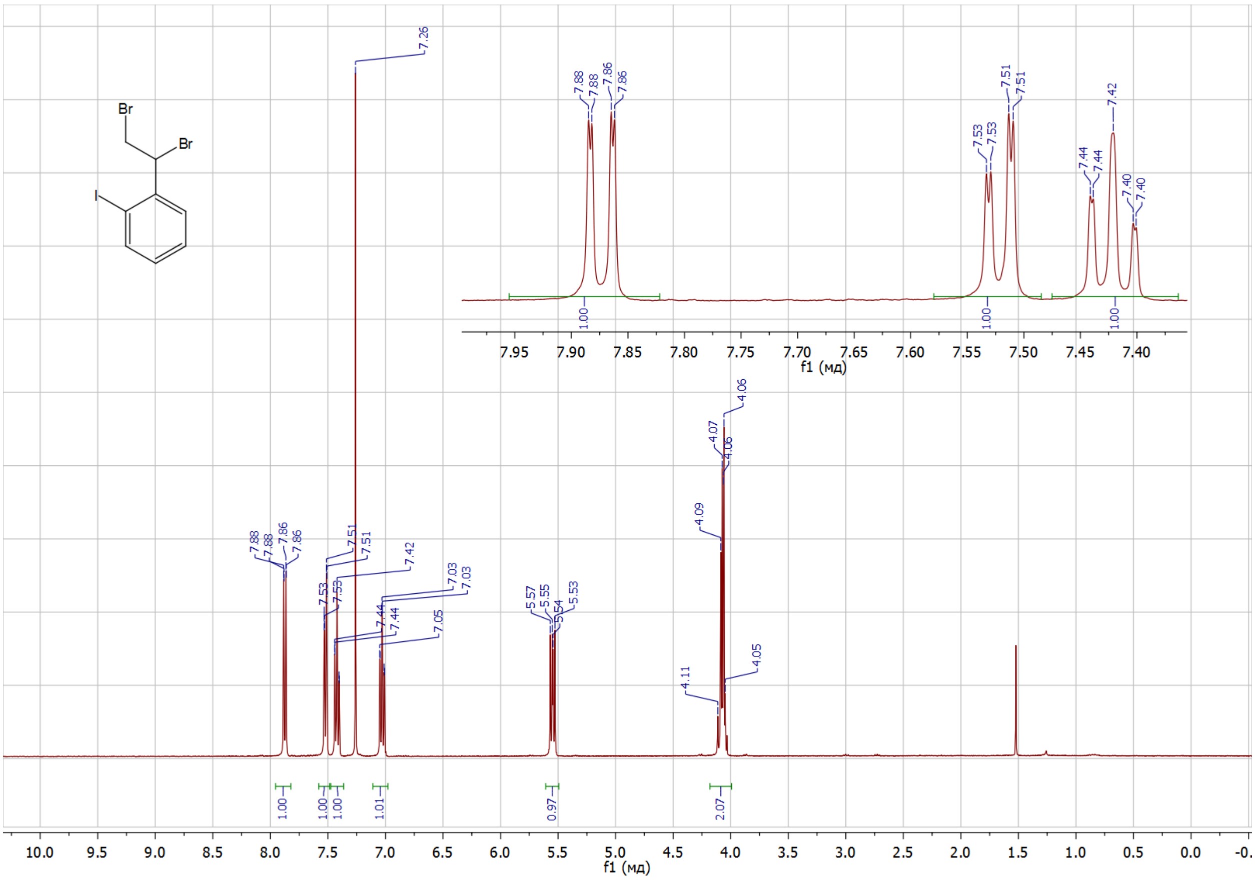

^{13}C{1H} NMR (101 MHz, $CDCl_3$) of 1-(1,2-dibromoethyl)-2-iodobenzene

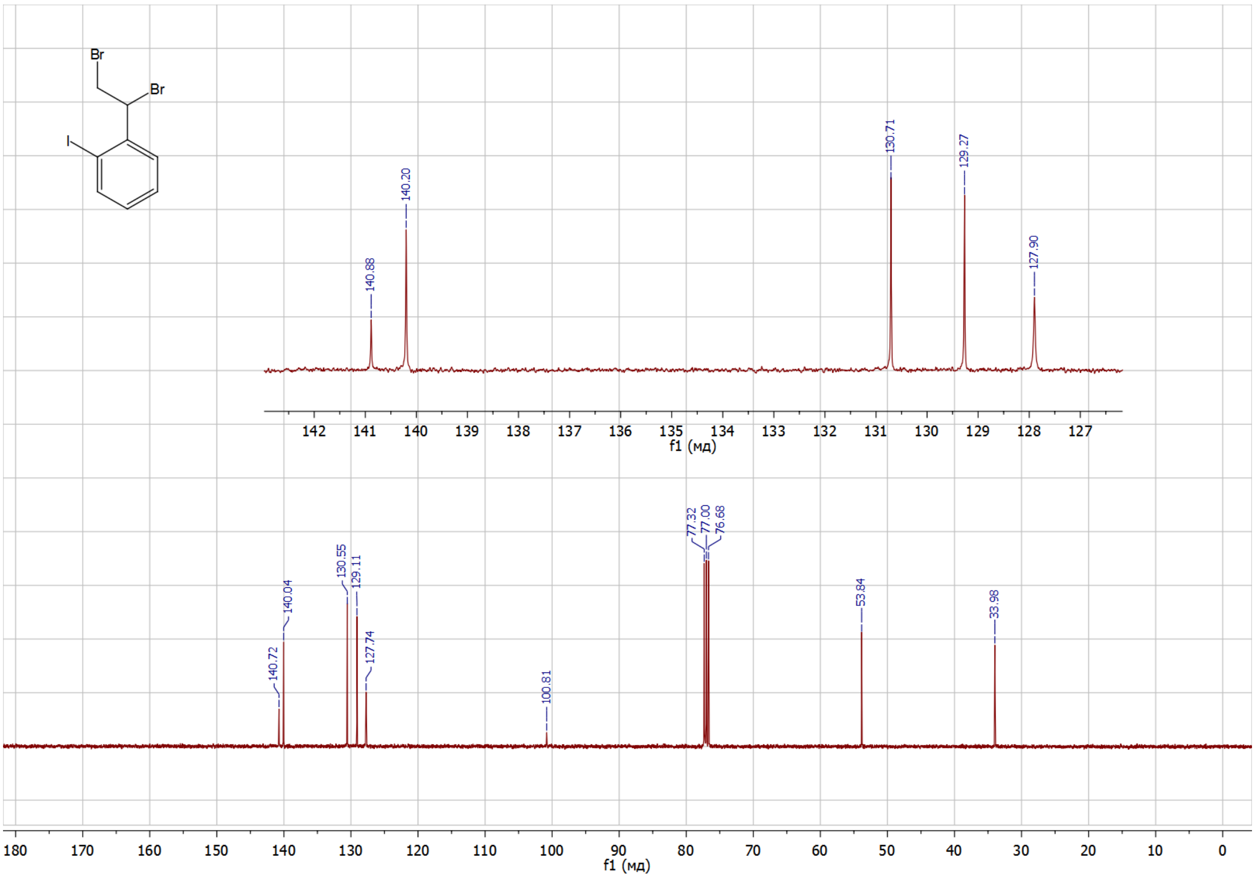

^{13}C DEPT135 NMR (101 MHz, $CDCl_3$) of 1-(1,2-dibromoethyl)-2-iodobenzene

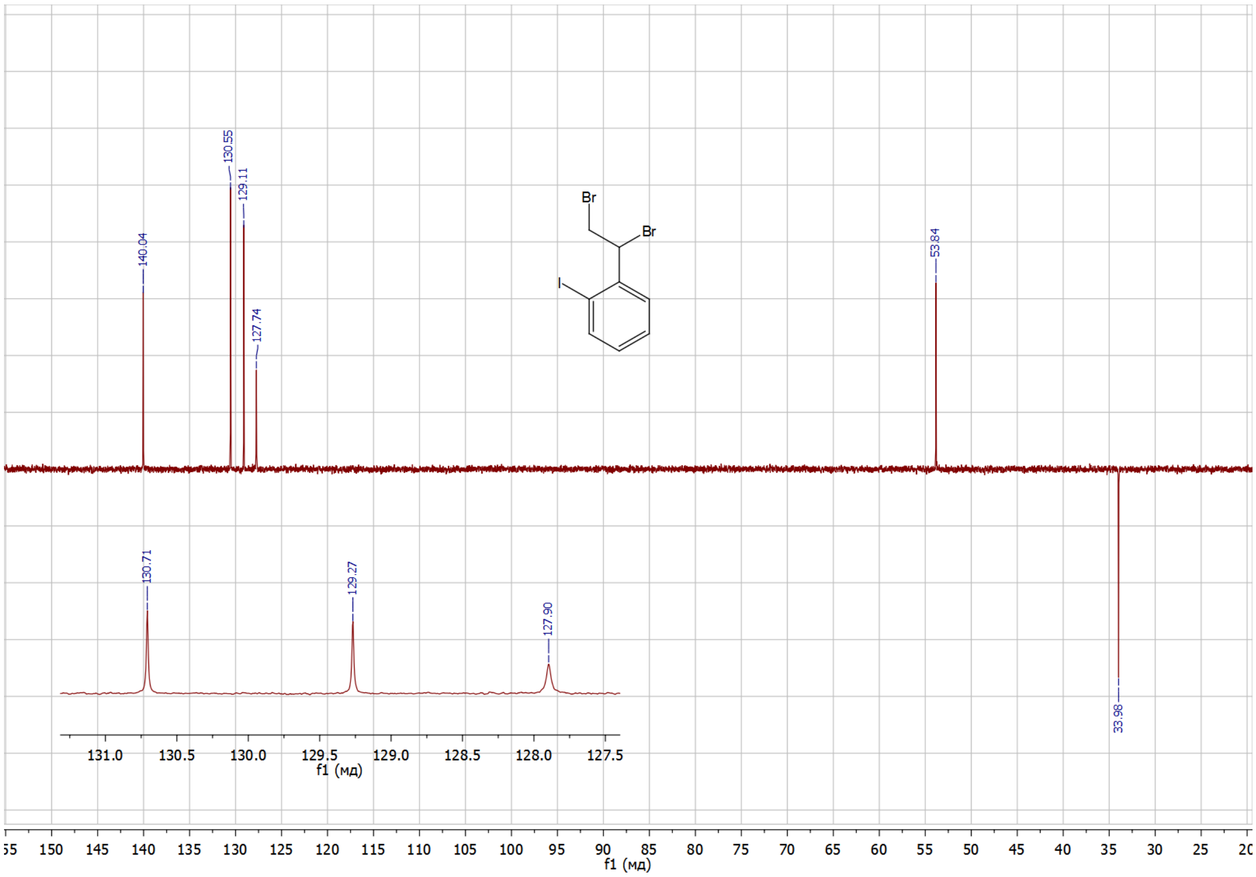

^{1}H NMR (400 MHz, $CDCl_3$) of 3-(2-iodophenyl)-2*H*-azirine

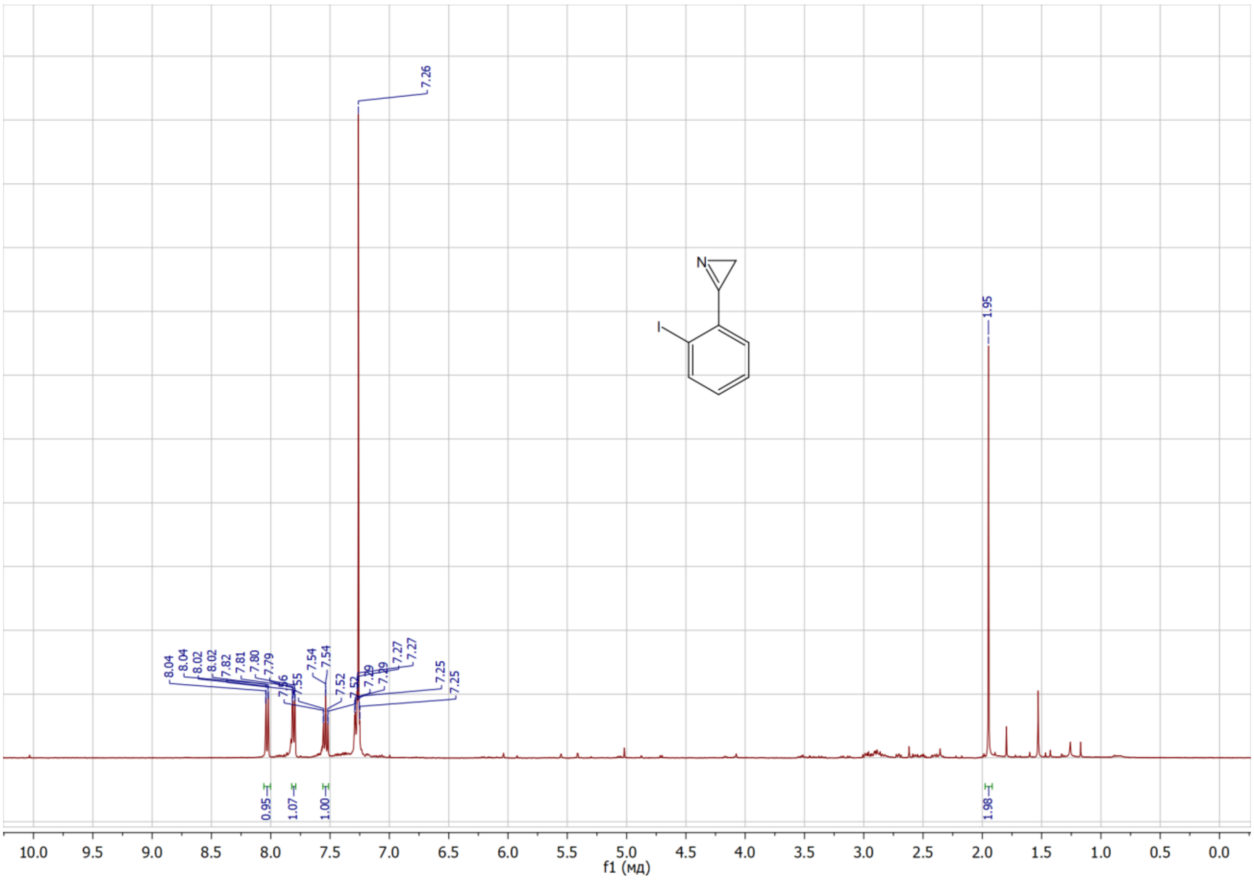

^{13}C{1H} NMR (101 MHz, $CDCl_3$) of 3-(2-iodophenyl)-2*H*-azirine

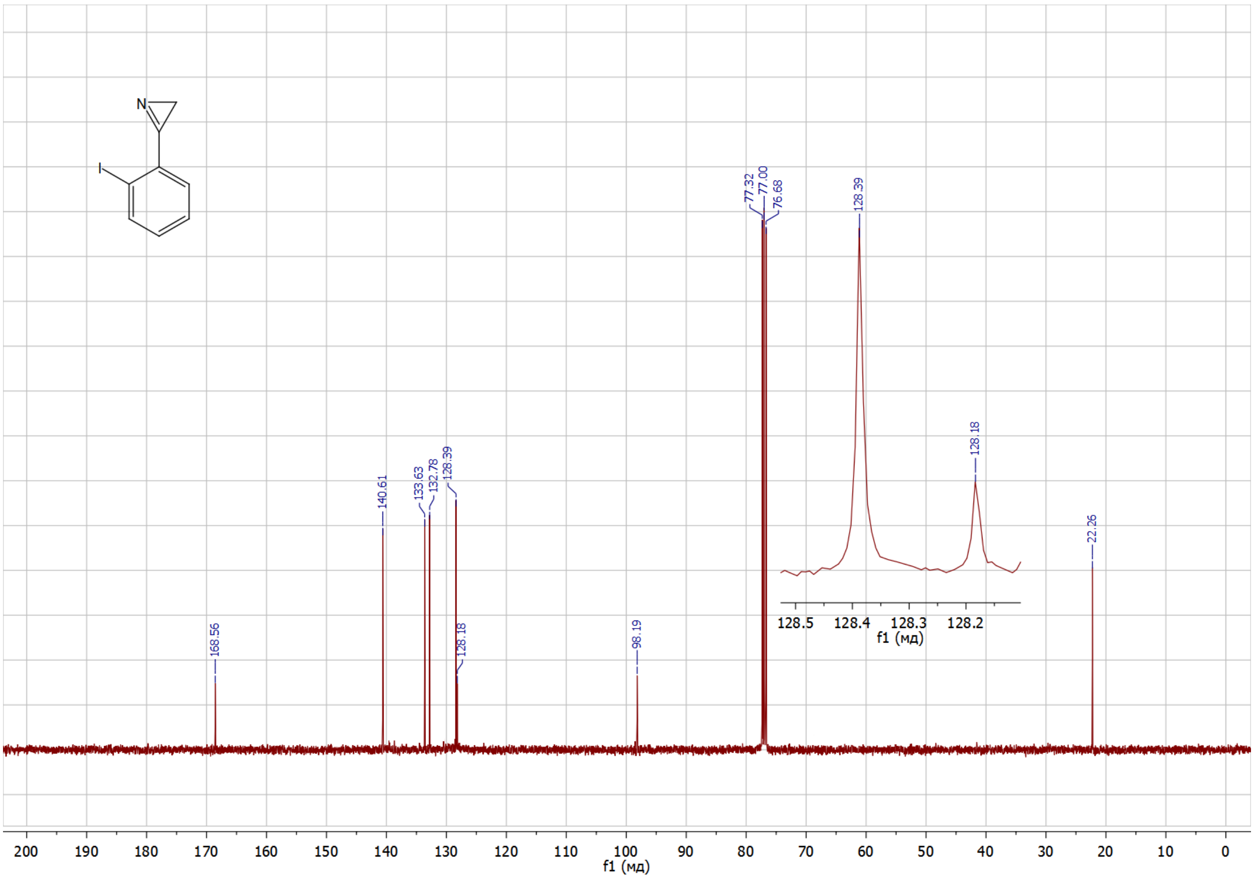

^{13}C DEPT135 NMR (101 MHz,CDCl3) of 3-(2-iodophenyl)-2*H*-azirine

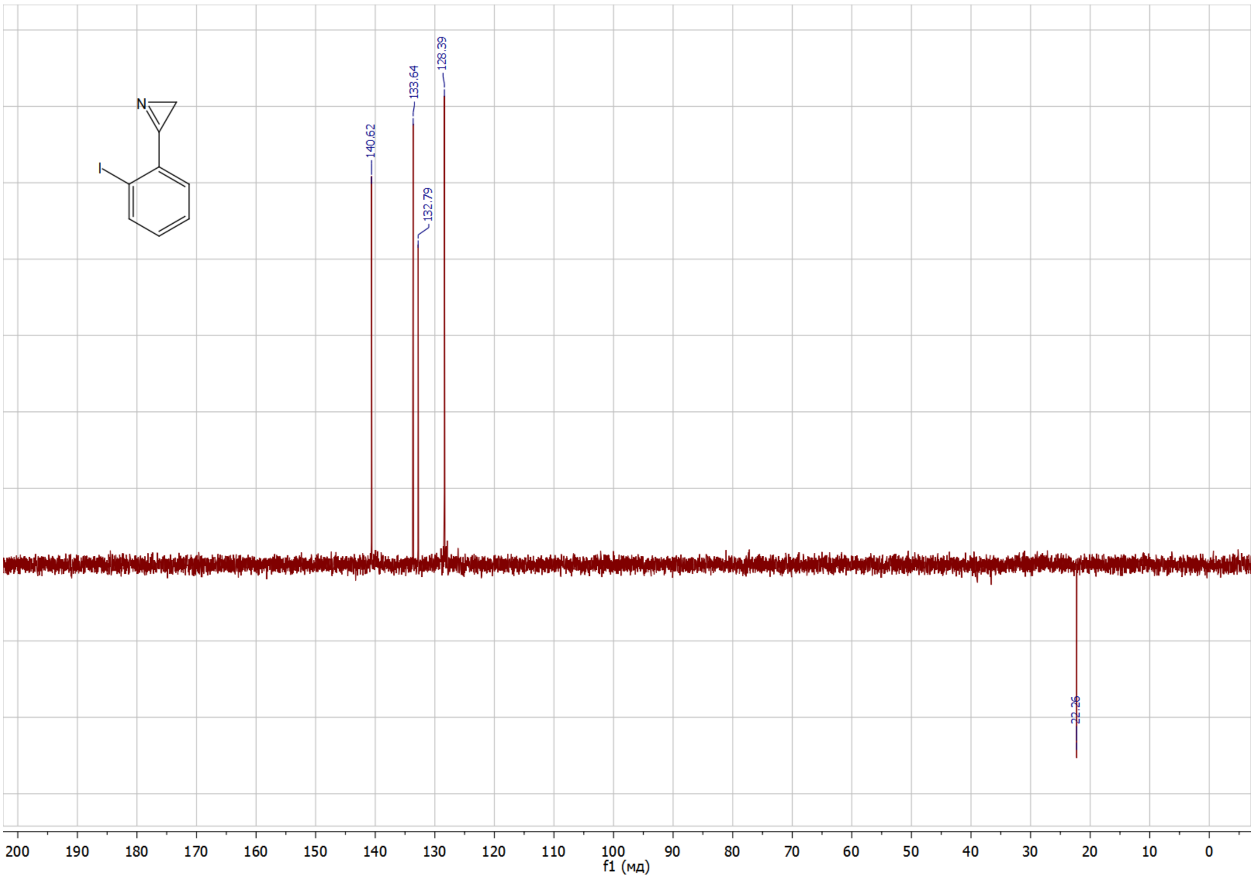

X-ray diffraction experiments

A suitable crystal was selected and studied on a Rigaku Oxford Diffraction «XtaLAB Supernova» with HyPix 3000 type detector. The crystal was kept at 100(2) K during data collection. The structure was solved using Olex2 [1] with SHELXT [2] structure solution program using Intrinsic Phasing. Structural refinement was carried out with SHELXL refinement package [2] using Least Squares minimisation.

[1] Dolomanov, O. V.; Bourhis, L. J.; Gildea, R. J.; Howard, J. A. K.; Puschmann, H., OLEX2: a complete structure solution, refinement and analysis program. *Journal of Applied Crystallography* **2009**, *42*, 339-341.

[2] Sheldrick, G., SHELXT - Integrated space-group and crystal-structure determination. *Acta Crystallographica Section A* **2015**, *71*, 3-8.

3-Phenylpyrido[2,1-*a*]pyrrolo[3,4-*c*]isoquinolin-2-ium bromide (**3a**).

Single crystals of **3a** were growth from methanol/diethyl ether at room temperature.

(CCDC 1974298).

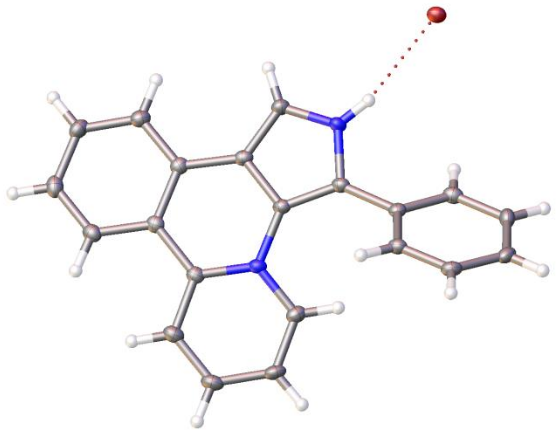

Crystal Data for $C_{21}H_{15}BrN_2$ (*M* =375.26 g/mol): monoclinic, space group P2$_1$/n (no. 14), *a* = 9.3373(3) Å, *b* = 9.3695(3) Å, *c* = 17.9629(5) Å, *β* = 97.580(3)°, *V* = 1557.77(8) Å^3, *Z* = 4, *T* = 100(2) K, μ(CuKα) = 3.606 mm^{-1}, *Dcalc* = 1.600 g/cm^3, 27246 reflections measured (9.936° ≤ 2Θ ≤ 152.942°), 3252 unique (R_{int} = 0.0785, R_{sigma} = 0.0343) which were used in all calculations. The final R_1 was 0.0326 (I≥2σ(I)) and wR_2 was 0.0847 (I≥2σ(I)).

Table S1 Crystal data and structure refinement for 3a.

Identificationcode	3a
Empiricalformula	$C_{21}H_{15}BrN_2$
Formulaweight	375.26
Temperature/K	100(2)
Crystalsystem	monoclinic
Spacegroup	P2$_1$/n
a/Å	9.3373(3)
b/Å	9.3695(3)

c/Å	17.9629(5)
α/°	90
β/°	97.580(3)
γ/°	90
Volume/Å^3	1557.77(8)
Z	4
D_{calc}g/cm^3	1.600
μ/mm^{-1}	3.606
F(000)	760.0
Crystalsize/mm^3	0.21 × 0.15 × 0.1
Radiation	CuKα (λ = 1.54184)
2Θ range for data collection/°	9.936 to 152.942
Indexranges	-11 ≤ h ≤ 11, -11 ≤ k ≤ 11, -22 ≤ l ≤ 22
Reflectionscollected	27246
Independentreflections	3252 [R_{int} = 0.0785, R_{sigma} = 0.0343]
Data/restraints/parameters	3252/0/217
Goodness-of-fit on F^2	1.063
Final R indexes [I≥2σ(I)]	R_1 = 0.0326, wR_2 = 0.0847
Final R indexes [all data]	R_1 = 0.0395, wR_2 = 0.0886
Largest diff. peak/hole / e Å^{-3}	0.51/-0.73

Table S2 Fractional Atomic Coordinates (×10^4) and Equivalent Isotropic Displacement Parameters (Å^2×10^3) for 3a. U_{eq} is defined as 1/3 of of the trace of the orthogonalised U_{IJ} tensor.

Atom	*x*	*y*	*z*	U(eq)
Br1	3820.3(2)	6148.2(3)	7966.3(2)	17.96(10)
N2	6410(2)	3028(2)	4998.6(11)	14.5(4)
N1	4699(2)	4463(2)	6511.3(11)	15.2(4)
C14	4149(2)	2988(2)	5554.1(13)	14.9(4)
C15	3620(3)	3649(2)	6150.8(13)	15.9(5)
C13	3519(2)	1969(2)	5008.1(13)	15.4(5)
C8	4421(3)	1415(2)	4504.6(13)	15.1(5)
C5	8092(3)	2148(3)	3929.5(14)	20.7(5)
C2	5600(2)	3457(3)	5575.0(13)	14.4(4)
C3	7696(3)	3689(3)	4937.8(14)	18.2(5)
C7	5878(3)	1980(3)	4496.4(13)	15.3(4)
C4	8551(3)	3262(3)	4416.5(14)	20.3(5)
C16	7283(2)	5029(3)	6558.4(13)	14.7(4)
C10	2453(3)	-74(3)	3948.4(14)	20.4(5)
C6	6765(3)	1531(3)	3968.3(14)	19.6(5)
C1	5952(2)	4354(3)	6189.3(13)	14.9(4)
C9	3862(3)	371(3)	3984.1(13)	18.0(5)
C20	9839(3)	4915(3)	7039.4(13)	18.5(5)
C18	8488(3)	7053(3)	7201.0(13)	19.0(5)
C17	7240(3)	6412(3)	6841.4(13)	17.6(5)
C11	1559(3)	485(3)	4444.9(14)	20.1(5)

C21	8596(3)	4275(3)	6677.1(13)	16.8(5)
C19	9792(3)	6309(3)	7297.9(14)	19.3(5)
C12	2086(3)	1487(3)	4972.4(14)	18.9(5)

Table S3 Anisotropic Displacement Parameters (Å^2×10^3) for 3a. The Anisotropic displacement factor exponent takes the form: $-2\pi^2[h^2a^{*2}U_{11}+2hka^*b^*U_{12}+\ldots]$.

Atom	U_{11}	U_{22}	U_{33}	U_{23}	U_{13}	U_{12}
Br1	15.96(14)	19.73(15)	19.01(15)	-1.14(9)	5.35(9)	1.28(8)
N2	13.7(9)	16.0(10)	14.5(9)	1.3(7)	4.7(7)	1.3(7)
N1	13.8(9)	18.0(10)	14.6(9)	-2.4(8)	4.3(7)	0.8(7)
C14	13.7(10)	15.0(11)	16.5(11)	2.8(9)	3.7(8)	2.1(8)
C15	12.9(10)	17.8(12)	17.1(11)	0.4(9)	2.9(9)	0.9(8)
C13	15.0(11)	15.0(11)	15.8(11)	2.7(9)	1.1(9)	1.2(8)
C8	16.7(11)	13.0(11)	15.8(11)	2.5(9)	2.3(9)	2.9(8)
C5	20.3(12)	24.4(13)	18.9(11)	1.1(10)	8.5(9)	4.8(10)
C2	13.6(10)	14.4(11)	16.1(11)	1.5(9)	4.6(8)	0.9(8)
C3	15.2(11)	21.0(12)	18.4(11)	3.6(9)	2.7(9)	-1.2(9)
C7	16.6(11)	14.8(11)	14.4(10)	1.8(8)	2.4(8)	2.2(8)
C4	14.9(11)	25.9(13)	21.5(12)	3.3(10)	7.4(9)	-1.1(9)
C16	14.1(11)	16.3(11)	14.5(10)	1.3(9)	4.4(8)	-0.7(8)
C10	25.5(13)	16.7(12)	18.0(12)	0.1(9)	-1.4(10)	-2.0(9)
C6	20.3(12)	22.5(12)	16.7(11)	-0.2(9)	5.4(9)	3.3(9)
C1	14.2(10)	14.6(11)	16.7(11)	1.3(9)	4.9(9)	2.3(8)
C9	21.5(12)	15.9(12)	16.9(11)	0.6(9)	4.0(9)	2.0(9)
C20	14.7(11)	22.8(12)	18.9(11)	2.6(10)	6.1(9)	1.6(9)
C18	22.7(12)	16.4(11)	17.9(11)	-2.9(9)	3.0(9)	-4.3(9)
C17	17.5(11)	18.5(12)	17.2(11)	2.4(9)	4.2(9)	4.0(9)
C11	16.1(11)	22.8(13)	20.9(12)	2.2(10)	0.4(9)	-4.2(9)
C21	16.7(11)	16.4(11)	18.4(11)	1.0(9)	6.2(9)	1.5(9)
C19	16.3(11)	24.5(13)	17.5(11)	0.6(9)	4.1(9)	-5.8(9)
C12	16.5(11)	21.5(12)	18.9(12)	1.9(10)	3.9(9)	-0.2(9)

Table S4 Bond Lengths for 3a.

Atom	Atom	Length/Å	Atom	Atom	Length/Å
N2	C2	1.418(3)	C2	C1	1.392(3)
N2	C3	1.369(3)	C3	C4	1.368(3)
N2	C7	1.381(3)	C7	C6	1.404(3)
N1	C15	1.358(3)	C16	C1	1.472(3)
N1	C1	1.376(3)	C16	C17	1.395(3)
C14	C15	1.384(3)	C16	C21	1.406(3)
C14	C13	1.438(3)	C10	C9	1.374(4)
C14	C2	1.420(3)	C10	C11	1.401(4)
C13	C8	1.414(3)	C20	C21	1.390(3)

C13	C12	1.405(3)	C20	C19	1.389(4)
C8	C7	1.461(3)	C18	C17	1.392(3)
C8	C9	1.405(3)	C18	C19	1.394(4)
C5	C4	1.393(4)	C11	C12	1.378(4)
C5	C6	1.377(4)			

Table S5 Bond Angles for 3a.

Atom	Atom	Atom	Angle/°	Atom	Atom	Atom	Angle/°
C3	N2	C2	119.2(2)	N2	C7	C6	117.4(2)
C3	N2	C7	120.9(2)	C6	C7	C8	122.1(2)
C7	N2	C2	119.86(19)	C3	C4	C5	119.4(2)
C15	N1	C1	112.1(2)	C17	C16	C1	120.0(2)
C15	C14	C13	132.0(2)	C17	C16	C21	118.6(2)
C15	C14	C2	106.1(2)	C21	C16	C1	121.3(2)
C2	C14	C13	121.8(2)	C9	C10	C11	120.3(2)
N1	C15	C14	107.6(2)	C5	C6	C7	121.6(2)
C8	C13	C14	117.1(2)	N1	C1	C2	104.7(2)
C12	C13	C14	123.1(2)	N1	C1	C16	119.5(2)
C12	C13	C8	119.8(2)	C2	C1	C16	135.5(2)
C13	C8	C7	120.5(2)	C10	C9	C8	120.8(2)
C9	C8	C13	118.7(2)	C19	C20	C21	120.1(2)
C9	C8	C7	120.6(2)	C17	C18	C19	120.4(2)
C6	C5	C4	119.2(2)	C18	C17	C16	120.5(2)
N2	C2	C14	119.4(2)	C12	C11	C10	120.2(2)
C1	C2	N2	131.1(2)	C20	C21	C16	120.8(2)
C1	C2	C14	109.4(2)	C20	C19	C18	119.6(2)
C4	C3	N2	121.3(2)	C11	C12	C13	120.1(2)
N2	C7	C8	120.4(2)				

Table S6 Hydrogen Atom Coordinates (Å×10[4]) and Isotropic Displacement Parameters (Å[2]×10[3]) for 3a.

Atom	*x*	*y*	*z*	U(eq)
H1	4607	4998	6905	18
H15	2674	3550	6284	19
H5	8686	1818	3575	25
H3	8002	4460	5264	22
H4	9448	3721	4387	24
H10	2084	-765	3586	25
H6	6443	784	3630	23
H9	4466	-31	3653	22
H20	10722	4398	7110	22
H18	8451	8003	7382	23
H17	6352	6921	6788	21

H11	586	171	4418	24
H21	8634	3317	6508	20
H19	10644	6753	7539	23
H12	1480	1853	5312	23

1*H*-dibenzo[*b*,*g*]pyrido[2,1,6-*de*]pyrrolo[2,3,4-*ij*]quinolizin-14-ium bromide (**13**).

Single crystals of **13** were growth from methanol/hexane at room temperature.

(CCDC 2049334).

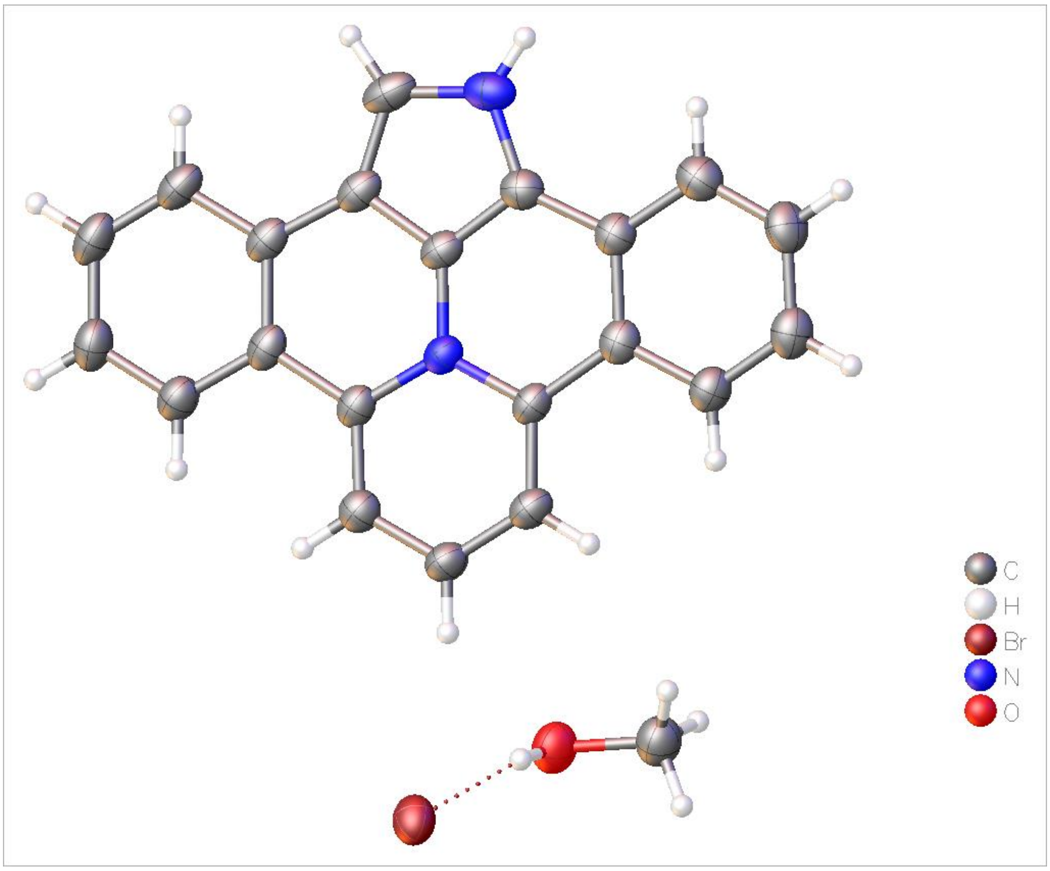

Crystal Datafor$C_{22}H_{17}BrN_2O$ (M = 405.28 g/mol):triclinic, space group P-1 (no. 2), a = 7.7049(3) Å, b = 9.5333(4) Å, c = 12.7349(3) Å, α = 111.349(3)°, β = 92.627(2)°,γ = 98.001(3)°, V = 858.01(6) Å^3, Z = 2, T = 100(2) K, μ(CuKα) = 3.366 mm^{-1},$Dcalc$ = 1.569 g/cm^3, 7090reflections measured (7.494° ≤ 2Θ ≤ 139.994°), 3243 unique (R_{int} = 0.0195, R_{sigma} = 0.0241) which were used in all calculations. The final R_1 was 0.0343 (I≥2σ(I)) and wR_2 was 0.0948(I≥2σ(I)).

Table S7 Crystal data and structure refinement for 13.

Identificationcode	13
Empiricalformula	$C_{22}H_{17}BrN_2O$
Formulaweight	405.28
Temperature/K	112.0(5)
Crystalsystem	triclinic

Spacegroup	P-1
a/Å	7.7049(3)
b/Å	9.5333(4)
c/Å	12.7349(3)
α/°	111.349(3)
β/°	92.627(2)
γ/°	98.001(3)
Volume/Å^3	858.01(6)
Z	2
D_{calc}g/cm^3	1.569
μ/mm^{-1}	3.366
F(000)	412.0
Crystalsize/mm^3	0.16 × 0.09 × 0.05
Radiation	CuKα (λ = 1.54184)
2Θ range for data collection/°	7.494 to 139.994
Indexranges	-9 ≤ h ≤ 9, -11 ≤ k ≤ 11, -12 ≤ l ≤ 15
Reflectionscollected	7090
Independentreflections	3243 [R_{int} = 0.0195, R_{sigma} = 0.0241]
Data/restraints/parameters	3243/0/238
Goodness-of-fit on F^2	1.084
Final R indexes [I>=2σ (I)]	R_1 = 0.0343, wR_2 = 0.0948
Final R indexes [all data]	R_1 = 0.0388, wR_2 = 0.0974
Largest diff. peak/hole / e Å^{-3}	0.91/-0.64

Table S8 Fractional Atomic Coordinates (×10^4) and Equivalent Isotropic Displacement Parameters (Å^2×10^3) for 13. U_{eq} is defined as 1/3 of of the trace of the orthogonalised U_{IJ} tensor.

**Atom**	***x***	***y***	***z***	**U(eq)**
Br01	581.9(4)	8833.5(3)	2505.1(2)	42.21(12)
O003	4827(3)	9252(2)	2517.6(16)	44.4(5)
C00Q	5360(4)	8753(4)	1406(2)	44.5(6)
N002	7380(2)	5512(2)	4502.4(16)	27.4(4)
N004	8791(3)	2013(3)	4138(2)	38.1(6)
N006	8479(3)	2531(3)	5290(2)	38.9(7)
C00H	7323(3)	4706(3)	7609(2)	36.8(6)
C00P	9034(4)	2216(3)	637(2)	39.9(6)
C00C	7356(3)	4943(3)	6581(2)	31.4(5)
C00B	6318(3)	7838(3)	5339(2)	32.3(5)
C00F	6823(3)	6560(3)	5449(2)	28.9(5)
C00G	6940(3)	7002(3)	3412(2)	32.2(5)
C00O	6761(3)	5753(4)	8538(2)	41.2(6)

C00J	6384(3)	8061(3)	4329(2)	32.8(5)
C00E	8495(3)	3163(3)	2606(2)	30.9(5)
C00D	8044(3)	4508(3)	2512(2)	31.1(5)
C00M	6223(4)	7054(4)	8467(2)	40.9(6)
C00N	8620(3)	3547(3)	544(2)	38.3(6)
C00A	6809(3)	6263(3)	6505(2)	31.1(5)
C00L	8993(3)	2030(3)	1661(2)	37.9(6)
C00I	8144(3)	4679(3)	1462(2)	34.1(5)
C00K	6245(3)	7313(3)	7468(2)	35.7(5)
C004	8791(3)	2013(3)	4138(2)	38.1(6)
C005	7876(3)	4218(3)	4584(2)	28.8(5)
C006	8479(3)	2531(3)	5290(2)	38.9(7)
C007	8413(3)	3065(3)	3702(2)	31.3(5)
C008	7453(3)	5698(3)	3466.2(19)	28.3(5)
C009	7906(3)	3897(3)	5570(2)	31.8(5)

Table S9 Anisotropic Displacement Parameters (Å^2×10^3) for 13. The Anisotropic displacement factor exponent takes the form: -2π^2[h^{2}a*^{2}U$_{11}$+2hka*b*U$_{12}$+…].

Atom	U_{11}	U_{22}	U_{33}	U_{23}	U_{13}	U_{12}
Br01	44.41(18)	39.32(18)	40.96(18)	13.79(12)	11.67(12)	1.84(12)
O003	45.2(11)	53.9(12)	35.7(10)	18.9(9)	3.4(8)	8.1(9)
C00Q	43.1(15)	53.2(18)	40.2(14)	20.3(13)	5.6(12)	9.4(13)
N002	23.2(9)	30.8(10)	30.2(10)	15.2(8)	0.7(7)	1.4(8)
N004	29.0(11)	31.4(12)	54.8(15)	17.8(11)	0.6(10)	4.7(9)
N006	28.7(11)	42.1(14)	54.1(15)	31.4(12)	-5.3(10)	-1.6(10)
C00H	31.5(12)	46.4(15)	39.0(13)	27.1(12)	-1.3(10)	-1.2(11)
C00P	38.8(14)	40.7(15)	31.7(13)	5.7(11)	3.0(10)	1.0(11)
C00C	23.1(10)	39.7(13)	34.5(12)	20.9(11)	-2.1(9)	-2.7(9)
C00B	27.3(11)	35.6(13)	34.2(12)	14.5(10)	1.5(9)	2.7(10)
C00F	22.3(10)	36.1(13)	29.6(11)	15.7(10)	0.2(8)	0.2(9)
C00G	29.2(11)	35.4(13)	35.0(12)	18.9(10)	-0.8(9)	1.2(10)
C00O	35.2(13)	58.9(18)	35.9(13)	28.2(13)	1.9(11)	0.1(12)
C00J	32.5(12)	35.1(13)	34.4(12)	17.7(10)	1.2(10)	5.3(10)
C00E	25.7(11)	32.4(12)	32.4(12)	11.9(10)	0.6(9)	-0.3(9)
C00D	24.6(11)	35.1(13)	31.1(12)	12.4(10)	-0.3(9)	-1.7(9)
C00M	37.6(13)	55.3(17)	31.8(13)	19.4(12)	6.2(10)	5.2(12)
C00N	32.7(12)	46.4(15)	31.6(12)	13.0(11)	-0.2(10)	-0.5(11)
C00A	23.0(11)	41.4(14)	30.8(12)	18.7(11)	0.2(9)	-1.9(10)
C00L	32.5(12)	36.0(14)	42.0(14)	11.8(11)	2.2(10)	3.0(10)
C00I	30.4(12)	39.7(14)	31.7(12)	14.5(11)	-0.1(10)	1.9(10)
C00K	32.2(12)	44.2(15)	35.2(13)	20.5(11)	3.0(10)	5.4(11)

C004	29.0(11)	31.4(12)	54.8(15)	17.8(11)	0.6(10)	4.7(9)
C005	22.7(10)	30.9(12)	35.1(12)	17.4(10)	-1.7(9)	-0.9(9)
C006	28.7(11)	42.1(14)	54.1(15)	31.4(12)	-5.3(10)	-1.6(10)
C007	25.6(11)	30.0(12)	37.0(12)	12.6(10)	0.1(9)	1.0(9)
C008	24.4(10)	33.1(12)	28.1(11)	15.1(10)	-1.2(9)	-1.3(9)
C009	24.2(10)	38.1(13)	36.9(12)	22.0(11)	-3.0(9)	-2.7(9)

Table S10 BondLengthsfor 13.

Atom	Atom	Length/Å	Atom	Atom	Length/Å
O003	C00Q	1.419(3)	C00F	C00A	1.471(3)
N002	C00F	1.388(3)	C00G	C00J	1.369(4)
N002	C005	1.378(3)	C00G	C008	1.379(3)
N002	C008	1.397(3)	C00O	C00M	1.393(4)
N004	C006	1.411(4)	C00E	C00D	1.417(4)
N004	C007	1.368(3)	C00E	C00L	1.405(4)
N006	C004	1.411(4)	C00E	C007	1.437(3)
N006	C009	1.361(4)	C00D	C00I	1.409(3)
C00H	C00C	1.408(3)	C00D	C008	1.471(4)
C00H	C00O	1.375(4)	C00M	C00K	1.382(4)
C00P	C00N	1.394(4)	C00N	C00I	1.377(4)
C00P	C00L	1.380(4)	C00A	C00K	1.404(4)
C00C	C00A	1.416(4)	C004	C007	1.368(3)
C00C	C009	1.436(4)	C005	C007	1.382(4)
C00B	C00F	1.381(3)	C005	C009	1.397(3)
C00B	C00J	1.380(3)	C006	C009	1.361(4)

Table S11 BondAnglesfor 13.

Atom	Atom	Atom	Angle/°	Atom	Atom	Atom	Angle/°
C00F	N002	C008	123.2(2)	C00C	C00A	C00F	121.8(2)
C005	N002	C00F	118.55(19)	C00K	C00A	C00C	118.7(2)
C005	N002	C008	118.2(2)	C00K	C00A	C00F	119.5(2)
C007	N004	C006	108.7(2)	C00P	C00L	C00E	120.2(3)
C009	N006	C004	108.9(2)	C00N	C00I	C00D	120.6(3)
C00O	C00H	C00C	120.2(3)	C00M	C00K	C00A	120.4(3)
C00L	C00P	C00N	120.0(3)	C007	C004	N006	108.7(2)
C00H	C00C	C00A	119.8(2)	N002	C005	C007	124.6(2)
C00H	C00C	C009	123.6(2)	N002	C005	C009	124.5(2)
C00A	C00C	C009	116.6(2)	C007	C005	C009	110.9(2)
C00F	C00B	C00J	121.0(2)	C009	C006	N004	108.9(2)
N002	C00F	C00A	119.0(2)	N004	C007	C00E	133.7(2)
C00B	C00F	N002	117.2(2)	N004	C007	C005	105.9(2)
C00B	C00F	C00A	123.8(2)	C004	C007	C00E	133.7(2)

C00J	C00G	C008	121.6(2)	C004	C007	C005	105.9(2)
C00H	C00O	C00M	120.2(2)	C005	C007	C00E	120.4(2)
C00G	C00J	C00B	120.2(2)	N002	C008	C00D	118.8(2)
C00D	C00E	C007	116.0(2)	C00G	C008	N002	116.8(2)
C00L	C00E	C00D	120.1(2)	C00G	C008	C00D	124.4(2)
C00L	C00E	C007	124.0(2)	N006	C009	C00C	134.7(2)
C00E	C00D	C008	121.9(2)	N006	C009	C005	105.7(2)
C00I	C00D	C00E	118.3(2)	C005	C009	C00C	119.6(2)
C00I	C00D	C008	119.8(2)	C006	C009	C00C	134.7(2)
C00K	C00M	C00O	120.7(3)	C006	C009	C005	105.7(2)
C00I	C00N	C00P	120.8(2)				

Table S12 TorsionAnglesfor 13.

A	B	C	D	Angle/°	A	B	C	D	Angle/°
N002	C00F	C00A	C00C	0.3(3)	C00D	C00E	C007	C004	-177.4(2)
N002	C00F	C00A	C00K	-179.9(2)	C00D	C00E	C007	C005	1.6(3)
N002	C005	C007	N004	179.7(2)	C00N	C00P	C00L	C00E	1.5(4)
N002	C005	C007	C00E	0.4(4)	C00A	C00C	C009	N006	-178.7(2)
N002	C005	C007	C004	179.7(2)	C00A	C00C	C009	C005	-0.1(3)
N002	C005	C009	N006	-179.6(2)	C00A	C00C	C009	C006	-178.7(2)
N002	C005	C009	C00C	1.4(3)	C00L	C00P	C00N	C00I	-0.9(4)
N002	C005	C009	C006	-179.6(2)	C00L	C00E	C00D	C00I	-1.2(3)
N004	C006	C009	C00C	178.5(3)	C00L	C00E	C00D	C008	177.7(2)
N004	C006	C009	C005	-0.2(3)	C00L	C00E	C007	N004	2.3(4)
N006	C004	C007	C00E	179.2(3)	C00L	C00E	C007	C004	2.3(4)
N006	C004	C007	C005	0.1(3)	C00L	C00E	C007	C005	-178.7(2)
C00H	C00C	C00A	C00F	179.9(2)	C00I	C00D	C008	N002	-179.4(2)
C00H	C00C	C00A	C00K	0.1(3)	C00I	C00D	C008	C00G	1.2(4)
C00H	C00C	C009	N006	0.7(4)	C004	N006	C009	C00C	178.5(3)
C00H	C00C	C009	C005	179.3(2)	C004	N006	C009	C005	-0.2(3)
C00H	C00C	C009	C006	0.7(4)	C005	N002	C00F	C00B	-179.4(2)
C00H	C00O	C00M	C00K	0.0(4)	C005	N002	C00F	C00A	0.9(3)
C00P	C00N	C00I	C00D	-0.7(4)	C005	N002	C008	C00G	179.7(2)
C00C	C00H	C00O	C00M	-0.1(4)	C005	N002	C008	C00D	0.3(3)
C00C	C00A	C00K	C00M	-0.2(4)	C006	N004	C007	C00E	179.2(3)
C00B	C00F	C00A	C00C	-179.3(2)	C006	N004	C007	C005	0.1(3)
C00B	C00F	C00A	C00K	0.5(3)	C007	N004	C006	C009	0.1(3)
C00F	N002	C005	C007	178.3(2)	C007	C00E	C00D	C00I	178.5(2)
C00F	N002	C005	C009	-1.8(3)	C007	C00E	C00D	C008	-2.6(3)
C00F	N002	C008	C00G	0.1(3)	C007	C00E	C00L	C00P	179.9(2)
C00F	N002	C008	C00D	-179.3(2)	C007	C005	C009	N006	0.3(3)

C00F	C00B	C00J	C00G	0.6(4)	C007	C005	C009	C00C	-178.7(2)
C00F	C00A	C00K	C00M	-180.0(2)	C007	C005	C009	C006	0.3(3)
C00O	C00H	C00C	C00A	0.1(4)	C008	N002	C00F	C00B	0.2(3)
C00O	C00H	C00C	C009	-179.3(2)	C008	N002	C00F	C00A	-179.50(19)
C00O	C00M	C00K	C00A	0.1(4)	C008	N002	C005	C007	-1.3(3)
C00J	C00B	C00F	N002	-0.6(3)	C008	N002	C005	C009	178.6(2)
C00J	C00B	C00F	C00A	179.1(2)	C008	C00G	C00J	C00B	-0.2(4)
C00J	C00G	C008	N002	-0.1(3)	C008	C00D	C00I	C00N	-177.2(2)
C00J	C00G	C008	C00D	179.3(2)	C009	N006	C004	C007	0.1(3)
C00E	C00D	C00I	C00N	1.7(3)	C009	C00C	C00A	C00F	-0.7(3)
C00E	C00D	C008	N002	1.7(3)	C009	C00C	C00A	C00K	179.5(2)
C00E	C00D	C008	C00G	-177.7(2)	C009	C005	C007	N004	-0.3(3)
C00D	C00E	C00L	C00P	-0.4(4)	C009	C005	C007	C00E	-179.5(2)
C00D	C00E	C007	N004	-177.4(2)	C009	C005	C007	C004	-0.3(3)

Table S13 Hydrogen Atom Coordinates (Å×10^4) and Isotropic Displacement Parameters (Å^2×10^3) for 13.

**Atom**	***x***	***y***	***z***	**U(eq)**
H003	3752.5	9032.3	2482.23	67
H00D	4896.57	9313.06	997.4	67
H00E	4920.03	7682.7	1020.21	67
H00F	6623.2	8927.46	1450.17	67
H004	9158.4	1175.53	3771.54	46
H006	8626.05	2060.89	5741.18	47
H00H	7683.37	3839.71	7660.48	44
H00P	9337.84	1452.9	8.09	48
H00B	5928.25	8559.55	5954.95	39
H00G	6973.53	7165.33	2735.78	39
H00O	6739.76	5592.22	9215.53	49
H00J	6049.93	8932.5	4270.66	39
H00M	5844.31	7756.86	9099.45	49
H00N	8667.13	3671.61	-146.04	46
H00L	9295.56	1152.06	1726.18	46
H00I	7884.53	5564.29	1387.45	41
H00K	5883.84	8188.08	7433.24	43
H00A	9188.3	1107.34	3741.68	46
H00C	8638.03	2022.61	5777.89	47

Table S14 AtomicOccupancyfor 13.

**Atom**	***Occupancy***	**Atom**	***Occupancy***	**Atom**	***Occupancy***

N004 0.56(3)	H004 0.56(3)	N006 0.44(3)
H006 0.44(3)	C004 0.44(3)	H00A 0.44(3)
C006 0.56(3)	H00C 0.56(3)	

3-Phenylpyrido[2,1-*a*]pyrrolo[3,2-*c*]isoquinolin-4-ium bromide (**17a**).

Single crystals of **17a** were growth from methanol/diethyl ether at room temperature.

(CCDC 2049337).

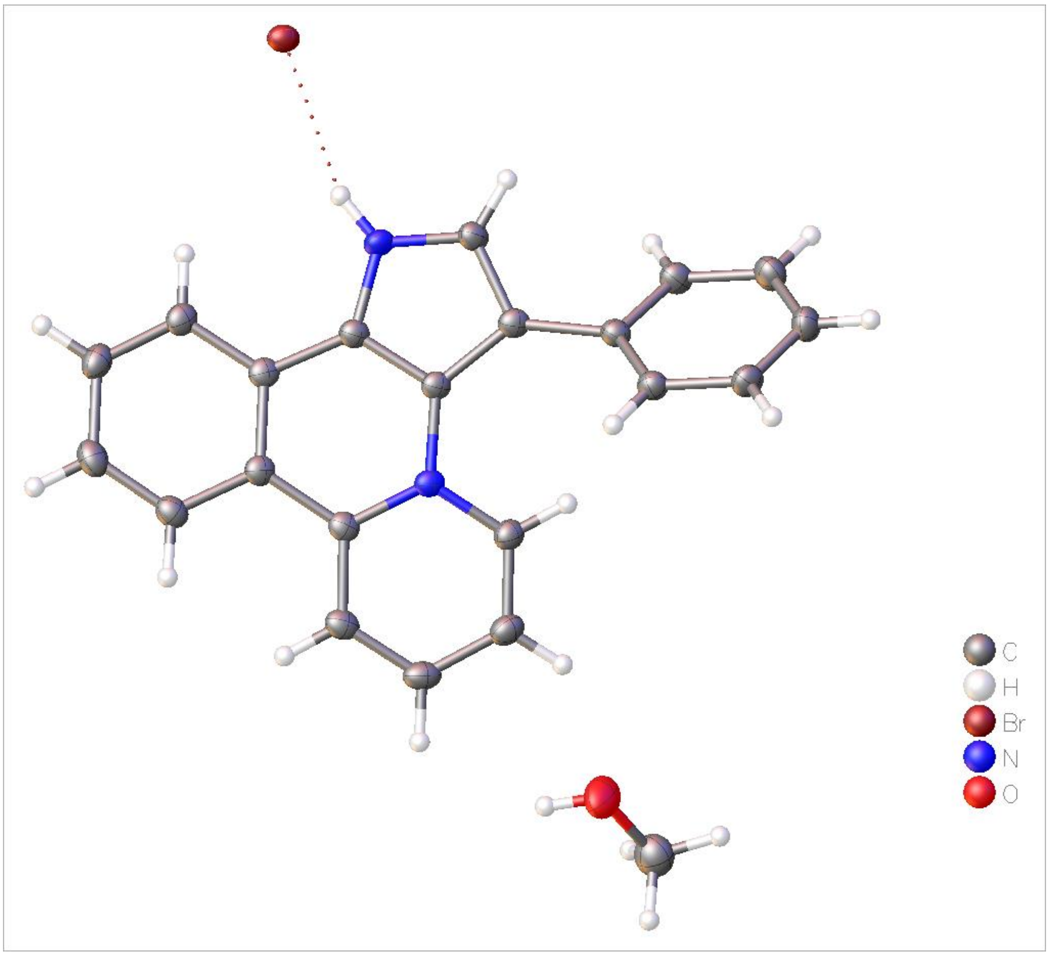

Crystal Data for $C_{22}H_{19}BrN_2O$ (M = 407.30g/mol): monoclinic, space group C2/c (no. 15), a = 25.8474(4) Å, b = 10.7503(2) Å, c = 13.1889(3) Å, β = 99.922(2)°, V = 3609.95(12) Å^3, Z = 8, T = 100(2) K, μ(CuKα) = 3.200 mm^{-1}, $Dcalc$ =1.499 g/cm^3, 9610 reflections measured (6.944° ≤ 2Θ ≤ 139.99°), 3421 unique (R_{int} = 0.0304, R_{sigma} = 0.0325) which were used in all calculations. The final R_1 was 0.0299 (I≥2σ(I)) and wR_2 was 0.0784 (I≥2σ(I)).

Table S15 Crystal data and structure refinement for 17a.

Identificationcode	17a
Empiricalformula	$C_{22}H_{19}BrN_2O$
Formulaweight	407.30
Temperature/K	100.0(2)
Crystalsystem	monoclinic

Spacegroup	C2/c
a/Å	25.8474(4)
b/Å	10.7503(2)
c/Å	13.1889(3)
α/°	90
β/°	99.922(2)
γ/°	90
Volume/Å^3	3609.95(12)
Z	8
D_{calc}g/cm^3	1.499
μ/mm^{-1}	3.200
F(000)	1664.0
Crystalsize/mm^3	0.15 × 0.11 × 0.02
Radiation	CuKα (λ = 1.54184)
2Θ range for data collection/°	6.944 to 139.99
Indexranges	$-26 \leq h \leq 31$, $-12 \leq k \leq 13$, $-16 \leq l \leq 14$
Reflectionscollected	9610
Independentreflections	3421 [R_{int} = 0.0304, R_{sigma} = 0.0325]
Data/restraints/parameters	3421/0/237
Goodness-of-fit on F^2	1.103
Final R indexes [I≥2σ (I)]	R_1 = 0.0299, wR_2 = 0.0784
Final R indexes [all data]	R_1 = 0.0327, wR_2 = 0.0799
Largest diff. peak/hole / e Å^{-3}	0.41/-0.62

Table S16 Fractional Atomic Coordinates (×10^4) and Equivalent Isotropic Displacement Parameters (Å^2×10^3) for 17a. U_{eq} is defined as 1/3 of of the trace of the orthogonalised U_{IJ} tensor.

Atom	*x*	*y*	*z*	U(eq)
Br01	4210.0(2)	7762.4(2)	3507.3(2)	20.88(9)
O003	6912.8(7)	-975.4(19)	2506.9(15)	41.7(5)
C00Q	7255.5(10)	-1513(3)	1911(2)	38.0(6)
N002	5004.2(7)	5404.0(16)	3718.9(13)	19.7(4)
N004	5598.7(7)	2438.6(16)	3853.1(13)	17.5(3)
C00K	7183.0(9)	6240(2)	4156.0(18)	27.5(5)
C00F	6208.4(9)	792(2)	3748.3(17)	24.5(5)
C00N	3732.6(9)	1677(2)	3773.9(17)	26.3(5)
C00O	7235.3(8)	4672(2)	5465.5(17)	24.1(5)
C00M	6716.7(8)	4332(2)	5101.8(16)	21.4(4)
C00J	7467.4(8)	5636(2)	5004.0(17)	26.6(5)
C00C	4548.7(8)	3346(2)	3705.0(15)	18.7(4)
C00L	4233.9(8)	1233(2)	3834.4(16)	22.9(4)
C00D	5806.7(9)	-75(2)	3742.2(17)	24.3(5)

C00G	6666.7(8)	5903(2)	3785.8(17)	23.7(4)
C00E	5189.1(8)	1599.5(19)	3828.9(15)	18.9(4)
C00A	5306.5(9)	327(2)	3777.7(15)	22.0(4)
C00H	4657.4(8)	2057(2)	3800.8(15)	19.2(4)
C00I	4028.4(8)	3779(2)	3616.0(15)	21.7(4)
C00B	6097.0(8)	2029(2)	3805.5(16)	20.9(4)
C00P	3625.2(9)	2953(2)	3662.6(17)	25.0(5)
C005	5511.3(8)	5784(2)	3847.7(15)	20.5(4)
C006	4988.9(8)	4142.5(19)	3747.2(15)	18.9(4)
C007	5506.4(8)	3724.5(19)	3890.9(15)	17.8(4)
C008	5849.9(8)	4779(2)	3970.3(15)	19.3(4)
C009	6424.0(8)	4959.8(19)	4265.6(16)	19.6(4)

Table S17 Anisotropic Displacement Parameters (Å^2×10^3) for 17a. The Anisotropic displacement factor exponent takes the form: $-2\pi^2[h^2a^{*2}U_{11}+2hka^*b^*U_{12}+\ldots]$.

Atom	U_{11}	U_{22}	U_{33}	U_{23}	U_{13}	U_{12}
Br01	22.69(13)	17.21(14)	20.89(14)	0.45(7)	-1.47(9)	1.78(8)
O003	28.7(9)	42.9(11)	49.2(11)	-19.1(9)	-5.3(8)	-2.1(8)
C00Q	36.9(14)	39.1(14)	37.4(14)	3.1(11)	4.7(11)	-2.7(11)
N002	19.4(8)	16.7(8)	21.4(9)	1.4(7)	-1.4(7)	3.4(7)
N004	19.7(9)	17.4(8)	13.8(8)	-0.2(6)	-1.7(6)	2.2(7)
C00K	25.4(11)	25.2(11)	31.5(12)	4.2(9)	3.4(9)	-3.6(9)
C00F	23.7(10)	24.6(11)	23.5(11)	-0.6(9)	-0.3(8)	5.5(9)
C00N	25.3(11)	29.2(12)	23.7(11)	-4.2(9)	2.3(8)	-7.4(9)
C00O	21.1(10)	24.3(11)	24.6(11)	3.1(9)	-2.8(8)	2.4(9)
C00M	21.3(10)	19.3(10)	22.8(10)	2.0(8)	1.5(8)	0.4(8)
C00J	19.6(10)	27.3(11)	31.0(12)	0.7(10)	-1.2(9)	-2.6(9)
C00C	20.9(10)	21.1(10)	12.8(9)	-1.3(8)	-0.8(7)	0.7(8)
C00L	25.9(11)	21.4(11)	20.1(10)	-0.1(8)	0.8(8)	-3.0(9)
C00D	31.3(12)	19.1(10)	20.5(10)	-1.4(8)	-1.2(9)	4.2(9)
C00G	24.3(11)	22.3(10)	22.4(11)	2.9(8)	-2.2(8)	0.0(9)
C00E	22.1(10)	19.8(10)	13.4(9)	-0.6(8)	-0.9(7)	-1.1(8)
C00A	26.6(11)	20.0(10)	17.3(10)	-1.2(8)	-2.2(8)	-1.2(9)
C00H	20.5(10)	21.3(10)	14.2(10)	-1.0(8)	-1.7(8)	0.3(8)
C00I	20.9(10)	25.1(11)	17.3(10)	-1.2(8)	-2.0(8)	1.6(9)
C00B	19.5(10)	22.3(10)	19.6(10)	-1.5(8)	-0.3(8)	1.6(8)
C00P	19.5(10)	31.1(12)	23.1(11)	-4.0(9)	0.0(8)	2.5(9)
C005	20.7(10)	18.3(10)	20.4(10)	0.3(8)	-2.4(8)	-0.7(8)
C006	20.5(10)	18.5(10)	16.4(10)	0.6(8)	-0.7(7)	0.5(8)
C007	19.4(10)	18.0(10)	14.4(9)	1.0(7)	-1.4(7)	1.0(8)
C008	21.5(10)	19.8(10)	15.2(10)	1.1(8)	-0.9(8)	1.5(8)

C009	20.9(10)	17.1(10)	19.9(10)	-0.3(8)	0.7(8)	-0.3(8)

Table S18 Bond Lengths for 17a.

Atom	Atom	Length/Å	Atom	Atom	Length/Å
O003	C00Q	1.406(3)	C00M	C009	1.398(3)
N002	C005	1.355(3)	C00C	C00H	1.416(3)
N002	C006	1.357(3)	C00C	C00I	1.409(3)
N004	C00E	1.387(3)	C00C	C006	1.417(3)
N004	C00B	1.373(3)	C00L	C00H	1.415(3)
N004	C007	1.405(3)	C00D	C00A	1.372(3)
C00K	C00J	1.389(3)	C00G	C009	1.400(3)
C00K	C00G	1.388(3)	C00E	C00A	1.406(3)
C00F	C00D	1.394(3)	C00E	C00H	1.454(3)
C00F	C00B	1.365(3)	C00I	C00P	1.378(3)
C00N	C00L	1.370(3)	C005	C008	1.382(3)
C00N	C00P	1.403(3)	C006	C007	1.393(3)
C00O	C00M	1.392(3)	C007	C008	1.433(3)
C00O	C00J	1.389(3)	C008	C009	1.481(3)

Table S19 Bond Angles for 17a.

Atom	Atom	Atom	Angle/°	Atom	Atom	Atom	Angle/°
C005	N002	C006	109.25(17)	C00C	C00H	C00E	120.35(19)
C00E	N004	C007	120.47(17)	C00L	C00H	C00C	118.23(19)
C00B	N004	C00E	120.57(18)	C00L	C00H	C00E	121.40(19)
C00B	N004	C007	118.93(18)	C00P	C00I	C00C	120.2(2)
C00G	C00K	C00J	120.3(2)	C00F	C00B	N004	121.6(2)
C00B	C00F	C00D	119.2(2)	C00I	C00P	C00N	119.8(2)
C00L	C00N	C00P	121.0(2)	N002	C005	C008	111.00(19)
C00J	C00O	C00M	120.5(2)	N002	C006	C00C	129.00(19)
C00O	C00M	C009	120.2(2)	N002	C006	C007	107.11(18)
C00O	C00J	C00K	119.6(2)	C007	C006	C00C	123.80(19)
C00H	C00C	C006	116.25(18)	N004	C007	C008	132.39(19)
C00I	C00C	C00H	120.2(2)	C006	C007	N004	118.53(18)
C00I	C00C	C006	123.5(2)	C006	C007	C008	108.86(18)
C00N	C00L	C00H	120.6(2)	C005	C008	C007	103.76(18)
C00A	C00D	C00F	119.7(2)	C005	C008	C009	120.93(19)
C00K	C00G	C009	120.6(2)	C007	C008	C009	134.71(19)
N004	C00E	C00A	117.64(19)	C00M	C009	C00G	118.83(19)
N004	C00E	C00H	119.68(18)	C00M	C009	C008	121.71(19)

C00A	C00E	C00H	122.59(19)	C00G	C009	C008	118.88(19)
C00D	C00A	C00E	121.4(2)				

Table S20 Torsion Angles for 17a.

A	B	C	D	Angle/°	A	B	C	D	Angle/°
N002	C005	C008	C007	0.8(2)	C00H	C00C	C00I	C00P	2.6(3)
N002	C005	C008	C009	-171.52(18)	C00H	C00C	C006	N002	-177.1(2)
N002	C006	C007	N004	-174.22(17)	C00H	C00C	C006	C007	-1.1(3)
N002	C006	C007	C008	1.0(2)	C00H	C00E	C00A	C00D	175.95(19)
N004	C00E	C00A	C00D	-0.6(3)	C00I	C00C	C00H	C00L	-1.8(3)
N004	C00E	C00H	C00C	5.7(3)	C00I	C00C	C00H	C00E	176.19(18)
N004	C00E	C00H	C00L	-176.37(18)	C00I	C00C	C006	N002	0.4(3)
N004	C007	C008	C005	173.2(2)	C00I	C00C	C006	C007	176.39(19)
N004	C007	C008	C009	-16.0(4)	C00B	N004	C00E	C00A	1.4(3)
C00K	C00G	C009	C00M	-2.3(3)	C00B	N004	C00E	C00H	-175.32(18)
C00K	C00G	C009	C008	169.2(2)	C00B	N004	C007	C006	168.27(18)
C00F	C00D	C00A	C00E	-0.5(3)	C00B	N004	C007	C008	-5.6(3)
C00N	C00L	C00H	C00C	0.1(3)	C00B	C00F	C00D	C00A	1.0(3)
C00N	C00L	C00H	C00E	-177.89(19)	C00P	C00N	C00L	C00H	0.9(3)
C00O	C00M	C009	C00G	1.9(3)	C005	N002	C006	C00C	176.1(2)
C00O	C00M	C009	C008	-169.3(2)	C005	N002	C006	C007	-0.5(2)
C00M	C00O	C00J	C00K	-1.8(3)	C005	C008	C009	C00M	127.5(2)
C00J	C00K	C00G	C009	0.6(4)	C005	C008	C009	C00G	-43.7(3)
C00J	C00O	C00M	C009	0.1(3)	C006	N002	C005	C008	-0.2(2)
C00C	C00I	C00P	C00N	-1.5(3)	C006	C00C	C00H	C00L	175.72(18)
C00C	C006	C007	N004	9.0(3)	C006	C00C	C00H	C00E	-6.3(3)
C00C	C006	C007	C008	-175.75(18)	C006	C00C	C00I	C00P	-174.79(19)
C00L	C00N	C00P	C00I	-0.2(3)	C006	C007	C008	C005	-1.1(2)
C00D	C00F	C00B	N004	-0.3(3)	C006	C007	C008	C009	169.6(2)
C00G	C00K	C00J	C00O	1.4(4)	C007	N004	C00E	C00A	179.17(17)
C00E	N004	C00B	C00F	-1.0(3)	C007	N004	C00E	C00H	2.5(3)
C00E	N004	C007	C006	-9.6(3)	C007	N004	C00B	C00F	-178.78(19)
C00E	N004	C007	C008	176.6(2)	C007	C008	C009	C00M	-42.0(3)
C00A	C00E	C00H	C00C	-170.85(19)	C007	C008	C009	C00G	146.8(2)
C00A	C00E	C00H	C00L	7.1(3)					

Table S21 Hydrogen Atom Coordinates (Å×10^4) and Isotropic Displacement Parameters (Å^2×10^3) for 17a.

Atom	*x*	*y*	*z*	U(eq)
H003	6608.71	-1259.77	2319.19	63

H00C	7097.87	-1467.18	1180.97	57
H00E	7317.66	-2385.63	2110.67	57
H00H	7589.79	-1061.31	2026.09	57
H002	4728.41	5897.33	3630.75	24
H00K	7342.62	6887.42	3828.23	33
H00F	6555.28	525.84	3713.07	29
H00N	3453.6	1111.52	3807.82	32
H00O	7431.9	4240.95	6033.41	29
H00M	6561.09	3672.7	5422.67	26
H00J	7818.15	5879.51	5266.35	32
H00L	4297.61	363.86	3899.6	27
H00D	5879.11	-939.31	3713.58	29
H00G	6476.3	6316.27	3201.61	28
H00A	5033.66	-267.64	3767.41	26
H00I	3955.21	4641.25	3523.64	26
H00B	6370.84	2619.74	3812.52	25
H00P	3275.58	3248.04	3619.42	30
H005	5619.61	6628.67	3853.05	25
